# Counterion-Mediated Enantioconvergent Synthesis of Axially Chiral Medium Rings

**DOI:** 10.1021/jacs.2c05485

**Published:** 2022-08-03

**Authors:** Ji-Yuan Du, Tudor Balan, Tim D. W. Claridge, Martin D. Smith

**Affiliations:** † Chemistry Research Laboratory, University of Oxford, 12 Mansfield Road, Oxford OX1 3TA, United Kingdom; ‡ College of Chemistry and Chemical Engineering, Liaocheng University, Liaocheng, Shandong 252059, China

## Abstract

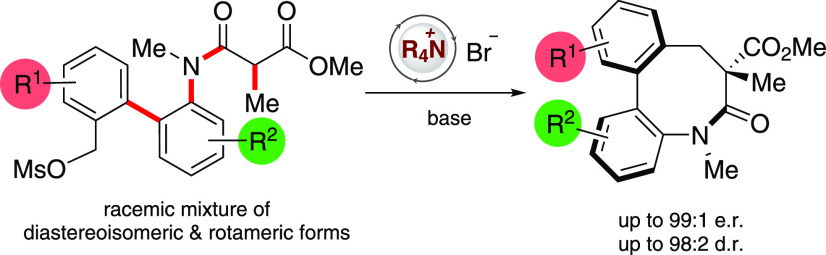

There are few enantioconvergent reactions in which racemic substrates bearing multiple stereochemical features are converted into products with high levels of diastereo- and enantiocontrol. Here, we disclose a process for the highly enantio- and diastereoselective syntheses of medium ring lactams via an intramolecular counterion-directed *C*-alkylation reaction. The treatment of racemic biaryl anilides that exist as a complex mixture of enantiomers and diastereoisomeric conformers by virtue of multiple axes of restricted rotation with a quinidine-derived ammonium salt under basic conditions affords medium ring lactams bearing elements of both axial and point chirality via an enolate-driven configurational relaxation process. Thermal equilibration of the *syn*- and *anti*-product diasteroisomers has demonstrated that the barriers to bowl inversion are >124 kJ mol^-1^. We propose that the chiral ammonium salt differentiates between a complex and rapidly equilibrating mixture of enolate and rotational isomers, ultimately leading to highly enantioselective alkylative ring closure. This dynamic and enantioconvergent process offers an operationally simple approach to the synthesis of valuable chiral medium ring lactams for which there are few catalytic and enantioselective approaches.

## Introduction

**Figure 1:**
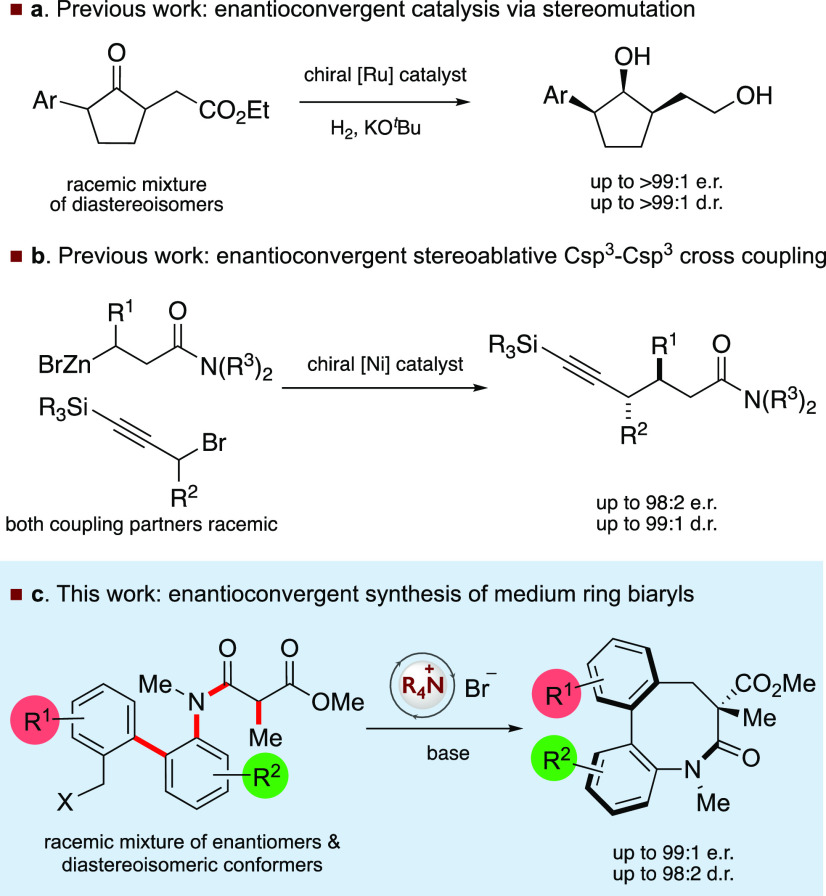
Enantioconvergent syntheses from substrates bearing multiple stereochemical features. (a) Enantioconvergent catalysis via stereomutation. (b) Enantioconvergent catalysis via stereoablation. (c) This work: A counterion mediated approach to enantioenriched dibenzolactams via an enantioconvergent *C*-alkylation.

Enantioconvergent catalytic reactions are those in which a racemic starting material is converted directly into an enantioenriched product. There are numerous examples of such transformations that convert racemic substrates bearing a single stereochemical element into the products with high levels of enantiocontrol.^1,2^ However, there are relatively few examples in which racemic substrates bearing multiple stereochemical features are converted into complex products with diastereo- and enantiocontrol. Zhou and co-workers have demonstrated that a racemic and diastereoisomeric mixture of α,α′-substituted cyclopentanones can be hydrogenated to a single product with high e.r. and d.r. in the presence of a chiral ruthenium complex (Figure 1a).^3^ Zhao and co-workers employed a chiral iridium complex in conjunction with a chiral phosphoric acid catalyst to enable a hydrogen borrowing protocol for the dynamic enantioselective amination of an isomeric mixture of secondary alcohols with almost complete stereocontrol.^4^ Kalek and Fu^5^ have shown a chiral nickel catalyst can enable enantioconvergent sp^3^–sp^3^ cross coupling from two racemic coupling partners (Figure 1b),^6^ and Jiang and co-workers have demonstrated enantioselective photoredox radical–radical coupling.^7^ We were interested in whether we could apply an enantioconvergent approach to the synthesis of biaryls bearing restricted rotation about the aryl–aryl axis. With the recognition of the increasing importance of the field of axial chirality, there has been an explosion of interest in enantioselective methods for the synthesis of these molecules.^8−12^ However, few of these methods are applicable to the synthesis of biaryls that are embedded in rings, despite these motifs being observed in potent bioactive natural products and medicinally relevant compounds.^13−16^

This may reflect the inherent challenges of constructing rotationally restricted biaryls, coupled with the difficulties of forming medium ring compounds with their transannular, large angle, and torsional strain.^17−21^ In addition, while progress has been made in the enantioselective synthesis of medium ring heterocycles,^22−25^ there are few enantioselective catalytic methods for the synthesis of medium ring biaryl compounds.^26−28^ We reasoned that a conceptually interesting approach to this class of compounds would be via the base mediated ring-closing *C*-alkylation of biaryl anilides (Figure 1c). This poses a number of challenges, as anilides are known to possess significant barriers to rotation about the *N*-aryl bond when appropriately substituted.^29−31^ In addition, the potential for restricted rotation about the biaryl and amide bonds means that these materials exist as a complex mixture of enantiomers and diastereoisomeric conformers. Curran and co-workers^33^ have elegantly demonstrated that the barrier to rotation of anilides is very significantly reduced upon enolate formation^32^ due to an increased facility for nitrogen pyramidalization in the cross conjugated amide enolate. We postulated that exploitation of this effect could enable rapid conformational and enolate equilibration of our substrates prior to ring closure and that a chiral counterion could differentiate between members of this dynamic ensemble, leading to an enantioconvergent ring closure with control of the axial and point chirality elements. We have previously demonstrated that a chiral counterion is able to differentiate between equilibrating anions in enantioselective *O*-alkylation reactions,^34^ and this tenet extends that observation.^35^

## Results and Discussion

A model anilide substrate **1** was constructed in a five-step procedure from *N*-methyl iodoaniline that included a palladium mediated cross coupling of 2-hydroxybenzene boronic acid hemiester with palladium acetate, *N*-acylation to introduce the β-keto amide functionality and transformation of a primary alcohol into a bromide leaving group (see Supporting Information p 9). Model substrate **1** is point chiral and racemic and exists as a complex mixture of rotameric forms, as demonstrated by ^1^H NMR spectroscopy. With a model substrate in hand, we commenced our investigations of the cyclization reaction by examining the base catalyzed *C*-alkylation process (Table 1). When the substrate was stirred with stoichiometric LiHMDS at RT, a single diastereoisomer of (racemic) product **2** was produced in 66% yield. In contrast, we observed that treatment with tetrabutylammonium ammonium bromide and KOH led to an effective cyclization but favored a different diastereoisomer (d.r. 4:1). This change in diastereoselectivity is likely a consequence of deprotonation and cyclization being faster than conformational equilibration in the presence of stoichiometric LiHMDS, so the diastereoselectivity more closely reflects the geometry attained from the kinetic and irreversible deprotonation. In contrast, with a reversible equilibrium deprotonation in the presence of the ammonium salt, equilibration may be faster than cyclization, and so, the diastereoselectivity reflects the relative rates of cyclization of the (rotameric) transition states, leading to different diastereoisomers.

**Table 1:**
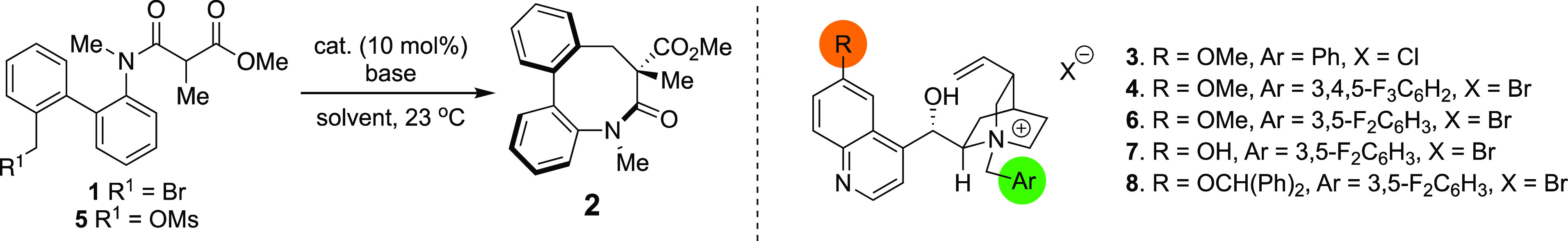
Optimization of the Ring Closing Reaction^a^

entry	catalyst	base	solvent	substrate	yield (%)^b^	d.r.^c^	e.r.^d^
1		LiHMDS	THF	**1**	66	<1:20	
2	Bu_4_NBr	KOH (aq.)	toluene	**1**	78	4:1	
3	**3**	K_3_PO_4_ (aq.)	toluene	**1**	11	75:25	73:27
4	**3**	Cs_2_CO_3_ (aq.)	toluene	**1**	29	85:15	90:10
5	**4**	Cs_2_CO_3_ (aq.)	toluene	**1**	19	95:5	92:8
6	**4**	Cs_2_CO_3_ (aq.)	benzene	**1**	18	93:7	92:8
7	**4**	Cs_2_CO_3_ (aq.)	Et_2_O	**1**	10	88:12	90:10
8	**4**	Cs_2_CO_3_ (aq.)	TBME	**1**	11	91:9	91:9
9	**4**	Cs_2_CO_3_ (aq.)	CPME	**1**	25	95:5	92:8
10	**4**	KOH (aq.)	CPME	**1**	>99	95:5	90:10
11	**4**	KOH (aq.)	CPME	**5**	92	92:8	93:7
12	**6**	KOH (aq.)	CPME	**5**	86	95:5	93:7
13	**7**	KOH (aq.)	CPME	**5**	90	94:6	93:7
14	**8**	KOH (aq.)	CPME	**5**	92	97:3	97:3

a Reaction conditions: substrate **1**/**5** (0.05 mmol, 1.0 equiv), catalyst (0.1 equiv), and base (50% w/w aq.) in 1 mL of solvent stirred for 12–17 h at 23 °C. CPME = cyclopentyl methyl ether; TBME = *tert*-butylmethyl ether.

b Yields determined by ^1^H NMR spectroscopy using 1,3,5-trimethoxybenzene as internal standard.

c e.r. of major diastereoisomer determined by stationary phase HPLC.

d d.r. determined by ^1^H NMR spectroscopy.

Subsequently, we turned our attention to the enantioselective process and were pleased to observe that treatment with *N*-benzylquinidinium chloride **3** in the presence of potassium phosphate affected the desired cyclization, albeit in poor yield and selectivity (Table 1, entry 3). A change to cesium carbonate as base (entry 4) led to an encouraging increase in selectivity (85:15 d.r.; 90:10 e.r.) with a modest 29% yield. Selectivity was further improved with the exploration of a different *N*-benzylic group in **4** at the expense of yield. The exploration of a range of different solvents with catalyst **4** was mostly ineffective (entries 5–9), but a switch to potassium hydroxide in cyclopentyl methyl ether led to a striking increase in yield (to >99%) without significantly compromising selectivity (95:5 d.r.; 90:10 e.r., entry 10). At this stage, we probed how the leaving group could also affect reaction efficacy, recognizing that the relative rates of the ring closure and the interconversion of the rotameric forms of the substrate could have an impact on selectivity.

The change from a bromide leaving group to a methanesulfonate in **5** lead to a small increase in enantioselectivity (to 93:7 e.r., entry 11), and hence, we subsequently explored how the nature of the *N*-pendant group on the catalyst could influence enantioselectivity with a focus on this substrate. We observed that changing from *N*-3,4,5-trifluorobenzyl (in **4**) to *N*-3,5-difluorobenzyl (in **6**) led to an incremental increase in diastereoselectivity (to 95:5 d.r.), and the application of catalysts with a free phenolic group (as in **7**) gave very similar results (entries 11–13).

Finally, we observed that catalyst **8** in which the quinoline was functionalized with an *O*-diphenylmethyl group led to a further increase in selectivity (to 97:3 e.r. and 97:3 d.r., entry 14). In all cases, the minor diastereoisomer was produced in a significantly lower e.r. The absolute configuration of **2** was unambiguously determined by X-ray crystallography (see CCDC 2071682 for the crystal data).We were confident we had discovered the best catalyst for this transformation, and hence, we explored the substrate scope by initially examining the substitution on the aryl ring distal to the lactam nitrogen (Table 2). Electron-donating groups such as 4-methyl and 4-methoxy were well tolerated, and enantioenriched lactams **9** and **10** were obtained in excellent yields and selectivities (**9**: 95.5:4.5 e.r. and 93:7 d.r.; **10**: 95.5:4.5 e.r. and 95:5 d.r). Substrates with halogens in this position, such as fluorine and chlorine, were also transformed smoothly and selectively into the desired lactam products **11** (98:2 e.r.; 98:2 d.r.) and **12** (97.5:2.5 e.r.; 96:4 d.r.). Substituents in the 5-position such as fluorine and bromine have minimal impact on the broad, high selectivity observed; the corresponding medium ring products **13** (97.5:2.5 e.r.; 96:4 d.r.) and **14** (97:3 e.r.; 97:3 d.r.) were isolated in good yields with excellent e.r. and d.r. We also observed good stereocontrol with the cyclization of a substrate bearing fluorine in the 3-position of the distal arene chain to afford product **15** (95.5:4.5 e.r.; 92:8 d.r.). We next examined the impact of the introduction of substituents on the arene proximal to the lactam nitrogen and were able to demonstrate that both electron-rich and electron-deficient groups were well tolerated. In general, the yield and stereoselectivity of the cyclization was insensitive to the identity of the substituent in the 4′-position. We observed that electron-rich substituents including methoxy (**16**, 98:2 e.r.; 97:3 d.r.; 86% yield) and methyl (**17**, 97.5:2.5 e.r.; 97:3 d.r.; 74% yield) were tolerated with high stereoselectivity and yields. Substrates containing fluorine (**18**, 98.5:1.5 e.r.; 94:6 d.r.; 90% yield) or chlorine (**19**, 97:3 e.r.; 92:8 d.r.; 98% yield) also led to successful and selective cyclizations. The cyclization is tolerant of substitution in the 5′-position; trifluoromethoxy **20** (98:2 e.r.; 93:7 d.r.; 63% yield), methyl **21** (95.5:4.5 e.r.; 97:3 d.r.; 95% yield), fluorine **22** (97:3 e.r.; 86:14 d.r.; 84% yield), and chlorine **23** (97.5:2.5 e.r.; 93:7 d.r.; 99% yield) containing-substrates were all successfully transformed in this catalytic transformation with high levels of enantio- and diastereoselectivity. The introduction of strong conjugating 5′-electron withdrawing groups such as nitro and cyano was also investigated; substrates containing these groups cyclized in moderate yields and high levels of enantioselectivity to afford **24** (37:63 d.r.; 55% yield; 97.5:2.5 e.r. for minor diastereoisomer; 44:56 e.r. for major diastereoisomer) and **25** (70:30 d.r.; 62% yield; 96.5:3.5 e.r. for major diastereoisomer). These substrates also cyclized with significantly diminished d.r., which may reflect modulation of the starting substrate rotational barriers. It has been demonstrated that conjugating electron withdrawing groups *para* to the nitrogen increase the barriers to N–C rotation in axially chiral anilines.^36,37^ In anilides, *para*-electron withdrawing groups have been shown to reduce the barrier to amide N–CO rotation very significantly.^38,39^ In **24**, the barrier to N–Ar rotation is likely to be much higher than in a compound such as **2**. This is a consequence of ground state stabilization through delocalization of the (anilide) nitrogen with the conjugating nitro group. This will have an impact on the N–Ar rotational barrier in the reactive enolate intermediate that will likely slow down conformational relaxation relative to the rate of ring closure, resulting in the lower observed diastereoselectivity. Additionally, the installation of substituents on both aryl rings is also possible: **26** (99:1 e.r., 95:5 d.r., and 77% yield) and **27** (98.5:1.5 e.r., 95:5 d.r., and 86% yield) were obtained successfully with excellent stereoselectivities and good yields.

**Table 2:**
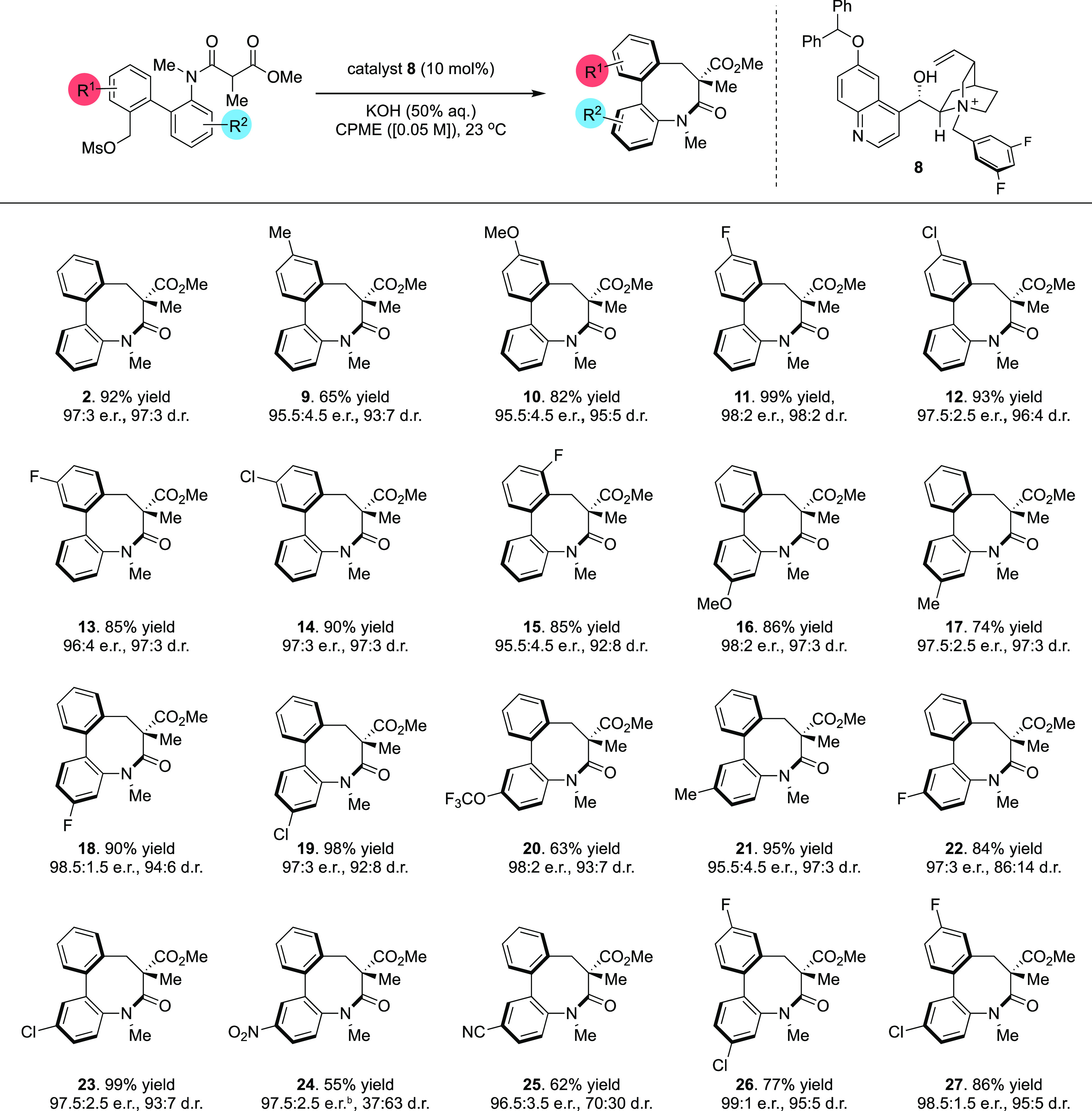
Scope of Enantioconvergent Ring Closing Reaction^a^

a Reaction conditions: substrate (0.1 mmol), catalyst **8** (0.1 mmol), KOH (50% aq. (w/w) 0.4 mmol), CPME (2 mL), 12–17 h, 23 °C. Yields are for isolated and purified material. The e.r. of the major diastereoisomer was determined by chiral stationary phase HPLC. The d.r. was determined by ^1^H NMR spectroscopy.

b The e.r. and structure shown are for the minor diastereoisomer (the e.r. for the major diastereoisomer = 44:56).

**Figure 2:**
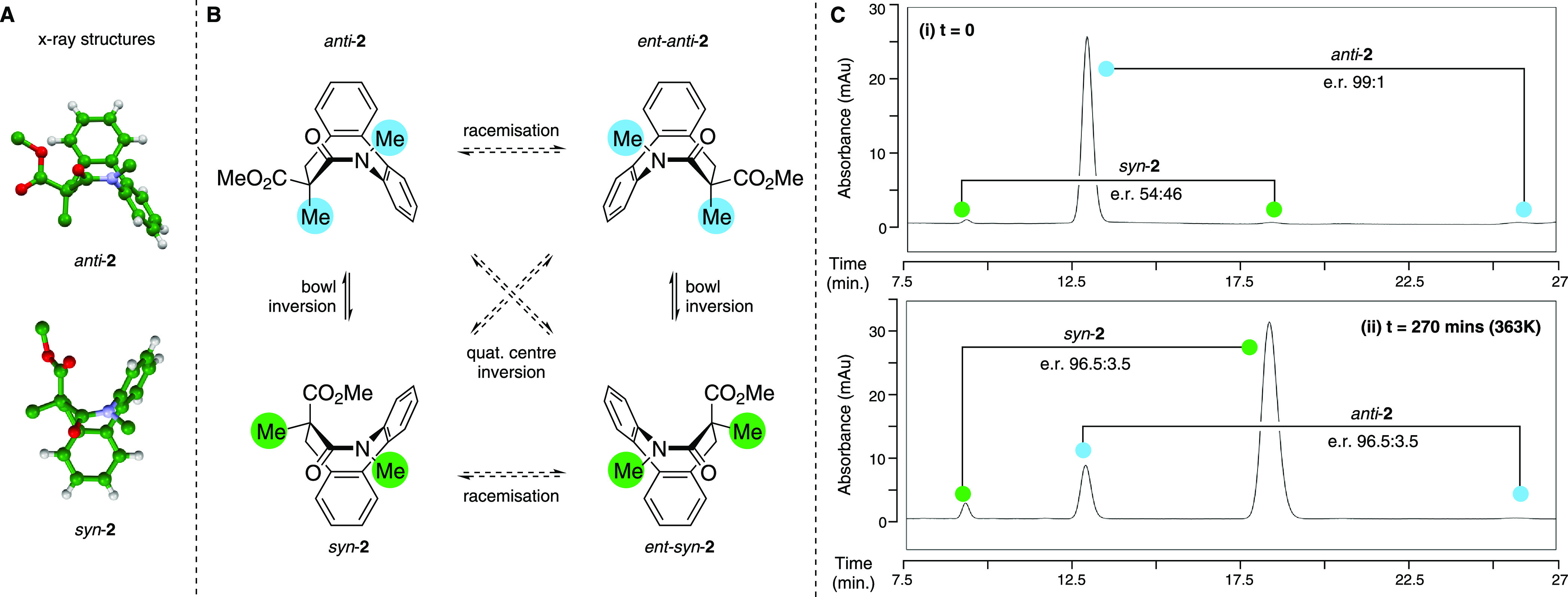
Conformation and configuration of dibenzolactam products. (A) Structures of *anti*-**2** (CCDC 2071682) and *syn*-**2** (CCDC 2071683) determined by X-ray diffraction. (B) Stereochemical relationships between all isomers of cyclic products; dotted lines indicate processes that do not occur under thermal conditions. (C) Chiral stationary phase HPLC traces: (i) enantioenriched products from a chiral catalyst mediated reaction (*t* = 0), 98:2 d.r. (*anti*/*syn*), 99:1 e.r. (*anti*), and 54:46 e.r. (*syn*); (ii) ratio of isomers after thermal equilibration (270 min at 363 K), 15:85 d.r. (*anti*/*syn*), 96.5:3.5 e.r. (*anti*), and 96.5:3.5 e.r. (*syn*).

The configurational stability of the related dibenzolactam compounds^35^ has been demonstrated by Natsugari and co-workers,^41^ and hence, we were confident that the products of our cyclization would be unlikely to change relative or absolute configuration under the reaction conditions. This is consistent with the observation that there was no change in e.r. or d.r. for **2** in toluene solution at 298 K for several weeks. The X-ray crystal structure (Figure 2A) of *anti*-**2** demonstrates that the biaryl lactam adopts a deep bowl-like arrangement in which the two aryl rings are twisted out of conjugation (torsion angle of 59°) and the *N*-methyl amide populates an *E*-configuration. The smaller C7 methyl group occupies a pseudoaxial position with the methyl ester substituent in an equatorial arrangement. The X-ray crystal structure of *syn*-**2** is relatively similar with the torsion between the aryl rings being slightly larger (62.8°). The amide is also *E*-configured by virtue of the overall bowl geometry, which is slightly twisted from an optimal boat conformation, likely to minimize eclipsing strain between the two sp^3^ carbons in the ring. Although **2** is, in principle, a two-axis system by virtue of the *N*-aryl and biaryl–biaryl bonds, we anticipated that inversion of the bowl shape of the eight membered ring would occur via a concerted process; this is consistent with the mechanism proposed for a related system by a detailed computational study.^40^ We assumed that the all-carbon quaternary stereocenter adjacent to the lactam carbonyl would be invariant under thermal conditions, which precludes the enantiomerization processes pictured (Figure 2B). Thus, we anticipated that we would observe the (independent) interconversion of two pairs of diastereoisomers under thermal equilibration.

To probe the magnitude of the barriers to rotation, we heated an enantio- and diastereoenriched *m*-xylene solution of **2** (starting composition: 98:2 d.r. (*anti*/*syn*); 99:1 e.r. (*anti*); 56:44 e.r. (*syn*)) to 90 °C and followed the thermal interconversion of these compounds over time with chiral stationary phase HPLC.^42−44^ The 98:2 (*anti*/*syn*) ratio of diastereoisomers was converted to an equilibrium mixture of 15:85 (*anti*/*syn*) in 270 min, and from this, we can calculate the barriers to bowl inversion to be Δ*G*_363 K_^‡^ = 124.1 kJ mol^–1^ (for the *anti*- to *syn*-conversion) and Δ*G*_363 K_^‡^ = 129.8 kJ mol^–1^ (for the *syn*- to *anti*-conversion).^45^ This appears to be broadly consistent with our original observation; barriers of this magnitude mean that interconversion at room temperature is practically nonexistent. The *syn*-isomer in which the methyl group populates a pseudoequatorial position is the most stable, which is also consistent with other observations of the relative size of a methyl ester vs a methyl group.^46^ A consequence of this equilibration is an increase in the population of *syn*-**2** (and a decrease in that of *anti*-**2**); while this is reflected in the diastereoisomeric ratio above, *syn*-**2** is also enantiomeric with *ent*-*syn*-**2**, which means that the e.r. of the *syn*-isomer increases from 54:46 to 96.5:3.5 (Figure 2C). As the total enantioselectivity in the system remains constant throughout the equilibration process, we observe a compensatory decrease in the e.r. of *anti*-**2** from the 99:1 starting point to the same enantiomeric ratio of 96.5:3.5.

**Figure 3:**
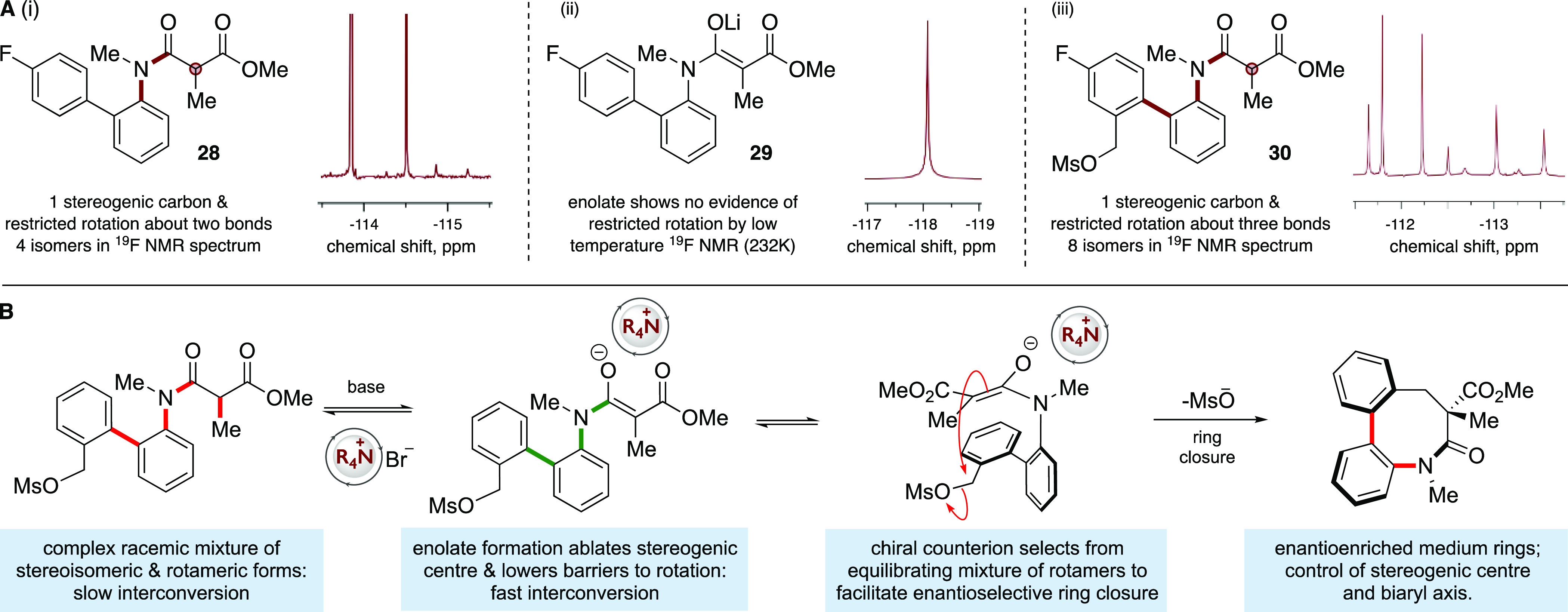
Proposed mechanism of enantioconvergent ring closure. (A) Partial proton-decoupled ^19^F NMR spectra of (i) model substrate **28** showing 4 peaks; (ii) model lithium enolate **29** derived from **28** demonstrating how the rotational profile changes upon deprotonation; (iii) cyclization precursor **30** showing 8 peaks. (B) Proposed mechanism of equilibrium enolate-driven configurational relaxation to enable rapid conformational exchange and subsequent counterion-mediated enantioselective cyclization.

To gain some insight into the mechanism of this enantioselective transformation, we probed the rotameric preferences of a model system **28** and the cyclization precursor mesylate **30** (Figure 3). Model compound **28** incorporates a diagnostic *para*-fluoro substituent on the biaryl ring but does not possess the activated *ortho*-hydroxymethyl substituent needed for cyclization. **28** has 4 signals in the proton decoupled ^19^F NMR spectrum, consistent with the anticipated slow rotation of the *N*-aryl and the amide N–CO bonds. Of the four rotameric species, two existed with very low population (>20:1) and were barely visible in the ^19^F spectrum, suggestive of high energy barriers to reach these conformational states. The NOE studies performed at 253 K where interconversion between the rotamers could not be detected indicated the two major species correlated with conformers in which the amide C=O was *anti*- to the aryl ring,^47,48^ and hence, the minor forms are anticipated to correspond to the carbonyl being *syn* to the aryl group. We employed variable temperature ^19^F 1D selective exchange spectroscopy (EXSY) NMR experiments to estimate the barrier to rotation of the *N*-aryl bond as 79.6 kJ mol^–1^ (see Supporting Information p 73 for details); this corresponds to a half-life of approximately 11 s at 298 K.

Upon formation of the enolate of **29** with stoichiometric LiHMDS, the spectrum simplifies to a single signal at −118 ppm. Low temperature ^19^F NMR experiments did not lead to decoalesence of the signal at temperatures as low as 223 K, indicating a very substantial decrease of the barriers to interconversion. The real system is significantly more complex: there are 8 signals in the proton decoupled ^19^F NMR of mesylate **30** consistent with the presence of 8 isomers (plus their mirror images); this is what would be expected from the restricted rotation about three bonds (*N*-aryl, aryl–aryl, and the amide N–CO) plus the presence of the stereogenic center. The complexity of this system made accurate determination of all of three rotational energy barriers intractable. A relatively low-energy interconversion could be monitored by 2D ^19^F EXSY experiments carried out at 253–303 K that indicated barriers Δ*G*_298_^‡^ to be in the range of 63–69 kJ mol^–1^. This is likely to be the aryl–aryl rotation with the increase in this specific barrier in **30** vs **28**, a consequence of the introduction of the *ortho*-substituent on the biaryl system. This *ortho*-substitution is likely to also increase the barrier of the *N*-aryl rotation, but higher temperature studies via ^19^F NMR to probe this presented a complex pattern of peak broadening without complete signal coalescence at temperatures up to 358 K. This suggests that the remaining two interconversion processes are of much higher energy, although precise barriers could not be determined (see Supporting Information p 77 for details).

From these data, we are able to suggest a plausible mechanism for the cyclization reaction (Figure 3B). The mesylate **5** exists as a racemic mixture of enantiomers and enantiomeric conformers. In the presence of base and the ammonium salt, reversible deprotonation of the 1,3-dicarbonyl ablates the stereogenic center; formation of this enolate disrupts the conjugation in the amide, enabling pyramidalization of the aniline nitrogen and lowering the barrier to rotation about the *N*-aryl bond very significantly, leading to rapid and reversible configurational and conformational equilibration under the reaction conditions. We postulate that the chiral ammonium counterion is able to select from this rapidly equilibrating ensemble to enable highly diastereo- and enantioselective cyclization, leading to the observed medium ring products.

## Conclusion

We have demonstrated that the highly enantio- and diastereoselective synthesis of axially chiral medium rings can be accomplished through an enantioconvergent counterion mediated cyclization. In this stereodynamic process, the formation of an enolate enables rapid interconversion of multiple isomeric forms of the starting substrate, between which the chiral counterion differentiates in the cyclization step. This process offers a general approach to ring-constrained biaryls and will likely find an application in materials and medicinal chemistry.

## Supporting Information Available

Experimental procedures and characterization data (PDF)
