# Supplementary material for: Counterion-Mediated Enantioconvergent Synthesis of Axially Chiral Medium Rings
Source: J Am Chem Soc. 2022 Aug 3;144(32):14790–7. doi: 10.1021/jacs.2c05485 (PMC9394463; doi:10.1021/jacs.2c05485)
Supplement: Supplementary file 1 — ja2c05485_si_001.pdf [file ja2c05485_si_001.pdf]

Supporting information

Counterion-mediated enantioconvergent synthesis of axially chiral medium rings

Ji-Yuan Du, [+a,b] Tudor Balan,[+a] Tim D. W. Claridge [a] & Martin D. Smith[a]*

[a]Chemistry Research Laboratory, University of Oxford, 12 Mansfield Road, Oxford, OX1 3TA, UK.

[b]College of Chemistry and Chemical Engineering, Liaocheng University, Liaocheng, Shandong 252059, China.

[+] These two authors contributed equally.

Contents

1. General Information. 2

2. Extended optimization for the synthesis of axially chiral dibenzolactams. 3

3. General procedure for the synthesis of substrates. 7

4. General Procedure for the Counterion Mediated Cyclization (2, 9-34). 53

5. Thermal Equilibration Studies 70

6. Absolute Configuration assignment of *anti*-2. 73

7. Relative Configuration assignment of *syn*-2. 74

8. NMR studies of rotational barriers of 28 and 30 75

9. NMR spectra 82

10. HPLC spectra 187

11. References. 233

1. General Information.

Reactions requiring moisture-sensitive reagents were carried out in flame-dried glassware, under an atmosphere of argon (balloon pressure). Dry dichloromethane and tetrahydrofuran were purified by filtration through activated alumina columns employing the method of Grubbs et al. Water was purified by an Elix® UV-10 system. Reagents were used directly as supplied by major chemical suppliers. Petrol refers to the fraction of petroleum ether which boils in the range 40 – 60 °C. Brine refers to a saturated aqueous solution of sodium chloride.

Silica gel chromatography was carried out using Merck Geduran® Silicagel (40–63 µm particle size). Thin layer chromatography (TLC) was carried out using pre-coated, aluminium backed plates (Merck Kieselgel 60 F254). Visualisation was achieved with ultraviolet irradiation (254 nm) and staining with permanganate.

NMR spectroscopy was carried out using Bruker Avance spectrometers in the deuterated solvent stated, using the residual non-deuterated solvent signal as an internal reference (^{1} H NMR: $CDCl_3$ (7.26), $(CD_3)_2SO$ (2.50); ^{13}C NMR: $CDCl_3$ (77.16), $(CD_3)_2SO$ (39.52); ^{19}F NMR: $CFCl_3$ (0.00)). Chemical shifts are quoted in ppm, based on appearance rather than interpretation. Signal patterns are indicated as: s, singlet; d, doublet; t, triplet; q, quartet; m, multiplet. Coupling constants, J, are quoted to the nearest 0.1 Hz and are presented as observed.

Infrared spectra were prepared as a neat film and were recorded using a Bruker Tensor 27 FTIR spectrometer using an ATR module. HRMS was carried out using Bruker MicroTDF and Micromass GCT spectrometers under electrospray ionization (ESI) or ammonia chemical ionization (CI)/electron ionization (EI) conditions respectively. Analytical chiral HPLC was carried out on a Dionex UltiMate 3000 HPLC system comprising a Dionex LPG-3400A pump, WPS-3000SL autosampler and TCC-3000SD column compartment, and a Daicel Chiralpak column (0.46 cm × 25 cm), equipped with an appropriate guard column (0.4 cm × 1 cm).

Melting points were determined using a Reichert melting point apparatus and are uncorrected.

Optical rotations were recorded on a Schmidt-Haensch Unipol L2000 polarimeter and values are quoted [mL g^{-1} dm^{-1}]. Concentrations are quoted in g/100 mL.

2. Extended optimization for the synthesis of axially chiral dibenzolactams.

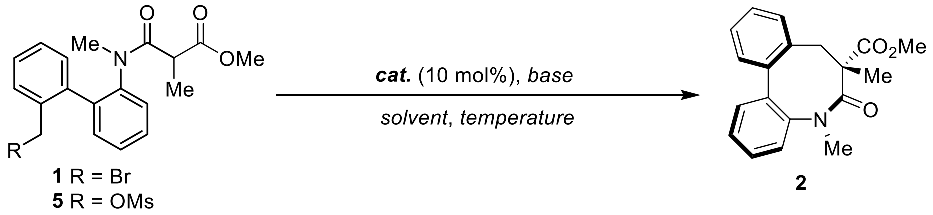

The anilide **1** or **5** (0.05 mmol, 1.0 equiv.) was suspended in the appropriate solvent and catalyst (0.1 equiv.) was added. The mixture was vigorously stirred before the base was added. After an appropriate time, the reaction was quenched with 1.0 M HCl aqueous (1.0 equiv.) and extracted with ethyl acetate. The organic phase was dried with anhydrous sodium sulfate, filtered and concentrated in vacuo. The crude mixture was purified by column chromatography to give the corresponding dibenzolactam **2**.

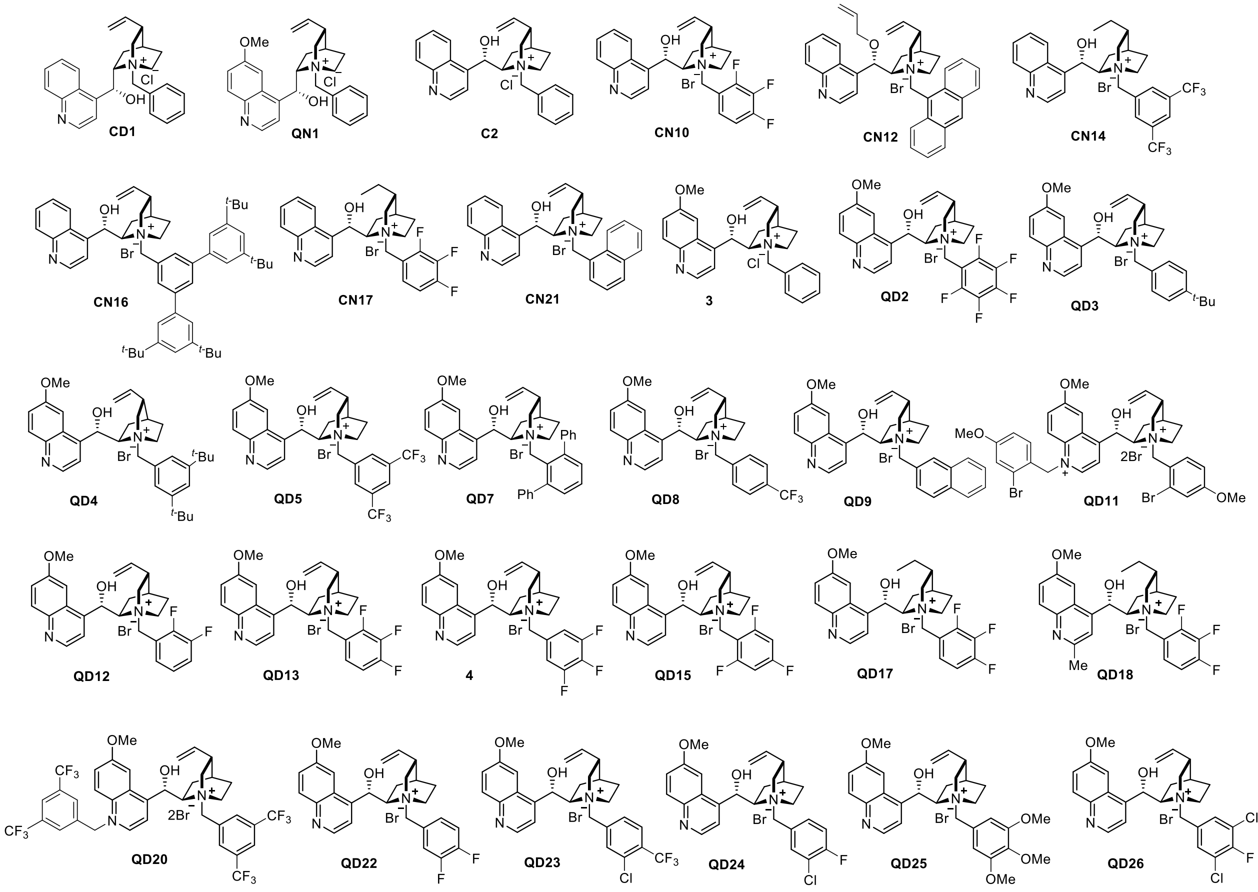

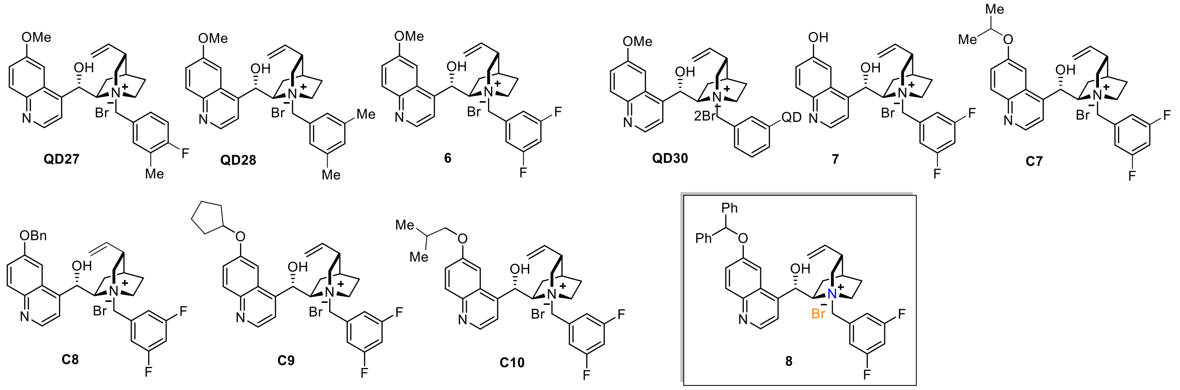

Figure S1. Screened catalysts.

Table S1: Extended optimization data.

Entry	R^1	Cat. (0.1 eq)	Base (equiv.)	Solvent (conc.)	Time (h)	T/ °C	Yield	d.r.[a]	e.e.[b]
1	Br	CD1	K_3PO_4 (solid, 5)	$PhCH_3$ (0.1 M)	72	23	31	71:29	30:70
2	Br	CN1	K_3PO_4 (solid, 5)	$PhCH_3$ (0.1 M)	72	23	56	86:14	54:45
3	Br	**3**	K_3PO_4 (solid, 5)	$PhCH_3$ (0.1 M)	72	23	11	75:25	73:27
4	Br	QN1	K_3PO_4 (solid, 5)	$PhCH_3$ (0.1 M)	72	23	18	72:28	36:64
5	Br	CN10	K_3PO_4 (solid, 5)	$PhCH_3$ (0.1 M)	72	23	35	94:6	58:42
6	Br	CN12	K_3PO_4 (solid, 5)	$PhCH_3$ (0.1 M)	72	23	58	87:13	50:50
7	Br	CN14	K_3PO_4 (solid, 5)	$PhCH_3$ (0.1 M)	72	23	43	94:6	53:47
8	Br	CN16	K_3PO_4 (solid, 5)	$PhCH_3$ (0.1 M)	72	23	42	86:14	55:45
9	Br	CN17	K_3PO_4 (solid, 5)	$PhCH_3$ (0.1 M)	72	23	65	97:3	59:41
10	Br	CN21	K_3PO_4 (solid, 5)	$PhCH_3$ (0.1 M)	72	23	54	86:14	57:43
11	Br	QD1	K_3PO_4 (sat. aq., 5)	$PhCH_3$ (0.1 M)	72	23	34	84:16	90:10
12	Br	QD1	Cs_2CO_3 (50% aq., 5)	$PhCH_3$ (0.1 M)	66	23	29	85:15	90:10
13	Br	QD1	Cs_2CO_3 (solid, 5)	$PhCH_3$ (0.1 M)	66	23	54	74:26	75:25
14	Br	QD1	KOH (solid, 2)	$PhCH_3$ (0.1 M)	66	23	82	58:42	68:32
15	Br	QD1	KOH (20%, aq., 2)	$PhCH_3$ (0.1 M)	66	23	40	77:23	85:15
16	Br	QD2	Cs_2CO_3 (50% aq., 5)	$PhCH_3$ (0.1 M)	72	23	3	77:23	59:41
17	Br	QD3	Cs_2CO_3 (50% aq., 5)	$PhCH_3$ (0.1 M)	72	23	28	69:31	81:19
18	Br	QD4	Cs_2CO_3 (50% aq., 5)	$PhCH_3$ (0.1 M)	72	23	17	59:41	69:31
19	Br	QD5	Cs_2CO_3 (50% aq., 5)	$PhCH_3$ (0.1 M)	72	23	17	90:10	75:25

20	Br	QD6	Cs_2CO_3 (50% aq., 5)	$PhCH_3$ (0.1 M)	72	23	8	73:27	66:34
21	Br	QD7	Cs_2CO_3 (50% aq., 5)	$PhCH_3$ (0.1 M)	72	23	9	67:33	65:35
22	Br	QD8	Cs_2CO_3 (50% aq., 5)	$PhCH_3$ (0.1 M)	72	23	15	86:14	86:14
23	Br	QD9	Cs_2CO_3 (50% aq., 5)	$PhCH_3$ (0.1 M)	72	23	14	85:15	88:12
24	Br	QD11	Cs_2CO_3 (50% aq., 5)	$PhCH_3$ (0.1 M)	72	23	5	74:26	80:20
25	Br	QD12	Cs_2CO_3 (50% aq., 5)	$PhCH_3$ (0.1 M)	72	23	14	89:11	88:12
26	Br	QD13	Cs_2CO_3 (50% aq., 5)	$PhCH_3$ (0.1 M)	72	23	9	90:10	89:11
27	Br	**4**	Cs_2CO_3 (50% aq., 5)	$PhCH_3$ (0.1 M)	72	23	19	95:5	92:8
28	Br	QD15	Cs_2CO_3 (50% aq., 5)	$PhCH_3$ (0.1 M)	72	23	10	55:45	56:44
29	Br	QD17	Cs_2CO_3 (50% aq., 5)	$PhCH_3$ (0.1 M)	72	23	11	85:15	84:16
30	Br	QD18	Cs_2CO_3 (50% aq., 5)	$PhCH_3$ (0.1 M)	72	23	17	88:12	87:13
31	Br	QD20	Cs_2CO_3 (50% aq., 5)	$PhCH_3$ (0.1 M)	72	23	N/A	N/A	N/A
32	Br	**4**	Cs_2CO_3 (50% aq., 5)	PhH (0.1 M)	54	23	18	93:7	92:8
33	Br	**4**	Cs_2CO_3 (50% aq., 5)	CH_2Cl_2 (0.1 M)	54	23	10	77:23	82:18
34	Br	**4**	Cs_2CO_3 (50% aq., 5)	$CHCl_3$ (0.1 M)	54	23	6	88:12	76:24
35	Br	**4**	Cs_2CO_3 (50% aq., 5)	$^{i}Pr_2O$ (0.1 M)	54	23	6	63:37	77:23
36	Br	**4**	Cs_2CO_3 (50% aq., 5)	CPME (0.1 M)	54	23	25	95:5	92:8
37	Br	**4**	Cs_2CO_3 (50% aq., 5)	Et_2O (0.1 M)	54	23	10	88:12	90:10
38	Br	**4**	Cs_2CO_3 (50% aq., 5)	TBME (0.1 M)	54	23	11	91:9	91:9
39	Br	**4**	Cs_2CO_3 (50% aq., 5)	DCE (0.1 M)	54	23	N/A	N/A	N/A
40	Br	**4**	Cs_2CO_3 (sat. aq., 5)	$PhCH_3$ (0.1 M)	54	23	35	94:6	91:9
41	Br	**4**	K_3PO_4 (sat. aq., 5)	PhH (0.1 M)	54	23	35	95:5	92:8
42	Br	**4**	NaOH (50% aq., 2)	PhH (0.1 M)	43	23	quant	95:5	76:24
43	Br	**4**	KOH (50% aq., 2)	PhH (0.1 M)	42	23	quant	93:7	87:13
44	Br	**4**	KOH (50% aq., 2)	CPME (0.1 M)	21	23	quant	95:5	90:10
45	Br	**4**	KOH (50% aq., 1.2)	CPME (0.1 M)	24	23	92	95:5	89:11
46	Br	**4**	KOH (50% aq., 1.2)	CPME (0.05 M)	24	23	N/A	95:5	90:10
47	Br	**4**	KOH (50% aq., 1.2)	CPME (0.025 M)	24	23	quant	96:4	89:11
48	Br	**4**	KOH (50% aq., 1.2)	CPME (0.1 M)	24	23	98	96:4	89:11
49	Br	**4**	CsOH (50% aq., 1.2)	CPME (0.1 M)	24	23	76	93:7	89:11

50	Br	**4**	CsOH (50% aq., 1.2)	CPME (0.1 M)	24	0	34	93:7	92:8
51	Br	**4**	KOH (50% aq., 1.2)	CPME (0.1 M)	24	10	87	96:4	91:9
52	Br	QD22	KOH (50% aq., 1.2)	CPME (0.05 M)	24	23	N/A	95:5	88:12
53	Br	QD23	KOH (50% aq., 1.2)	CPME (0.05 M)	24	23	N/A	91:9	83:17
54	Br	QD24	KOH (50% aq., 1.2)	CPME (0.05 M)	24	23	N/A	94:6	88:12
55	Br	QD25	KOH (50% aq., 1.2)	CPME (0.05 M)	24	23	N/A	72:28	73:27
56	Br	QD26	KOH (50% aq., 1.2)	CPME (0.05 M)	24	23	N/A	95:5	87:13
57	Br	QD27	KOH (50% aq., 1.2)	CPME (0.05 M)	24	23	N/A	92:8	88:12
58	Br	QD28	KOH (50% aq., 1.2)	CPME (0.05 M)	24	23	N/A	86:14	87:13
59	Br	**6**	KOH (50% aq., 1.2)	CPME (0.05 M)	24	23	N/A	95:5	90:10
60	Br	QD30	KOH (50% aq., 1.2)	CPME (0.05 M)	24	23	N/A	80:20	51:49
61	OMs	**4**	KOH (50% aq., 1.2)	CPME (0.05 M)	12	23	92	92:8	93:7
62	OMs	QD8	KOH (50% aq., 1.2)	CPME (0.05 M)	17	23	91	86:14	74
63	OMs	QD9	KOH (50% aq., 1.2)	CPME (0.05 M)	17	23	92	84:16	73
64	OMs	QD12	KOH (50% aq., 1.2)	CPME (0.05 M)	17	23	85	85:15	66
65	OMs	QD13	KOH (50% aq., 1.2)	CPME (0.05 M)	17	23	79	85:15	47
66	OMs	QD22	KOH (50% aq., 1.2)	CPME (0.05 M)	17	23	85	92:8	85
67	OMs	QD24	KOH (50% aq., 1.2)	CPME (0.05 M)	17	23	82	93:7	85
68	OMs	QD26	KOH (50% aq., 1.2)	CPME (0.05 M)	17	23	85	93:7	81
69	OMs	QD27	KOH (50% aq., 1.2)	CPME (0.05 M)	17	23	79	89:11	83
70	OMs	**6**	KOH (50% aq., 1.2)	CPME (0.05 M)	17	23	86	95:5	93:7
71	OMs	**7**	KOH (50% aq., 1.2)	CPME (0.05 M)	17	23	90	94:6	93:7
72	OMs	C7	KOH (50% aq., 1.2)	CPME (0.05 M)	17	23	89	94:6	92:8
73	OMs	**8**	KOH (50% aq., 1.2)	CPME (0.05 M)	17	23	80	97:3	96:4
74	OMs	C9	KOH (50% aq., 1.2)	CPME (0.05 M)	17	23	86	95:5	93:7
75	OMs	C8	KOH (50% aq., 1.2)	CPME (0.05 M)	17	23	83	96:4	94:6
76	OMs	C10	KOH (50% aq., 1.2)	CPME (0.05 M)	17	23	89	94:6	93:7
**77**	**OMs**	**8**	**KOH (50% aq., 4.0)**	**CPME (0.05 M)**	**17**	**23**	**92**	**97:3**	**97:3**
78	OMs	**8**	KOH (50% aq., 1.2)	CPME (0.05 M)	17	0	82	98:2	97:3

[a] e.e. determined by chiral stationary phase HPLC. [b] determined by chiral stationary phase HPLC.

2. General procedure for the synthesis of catalysts.

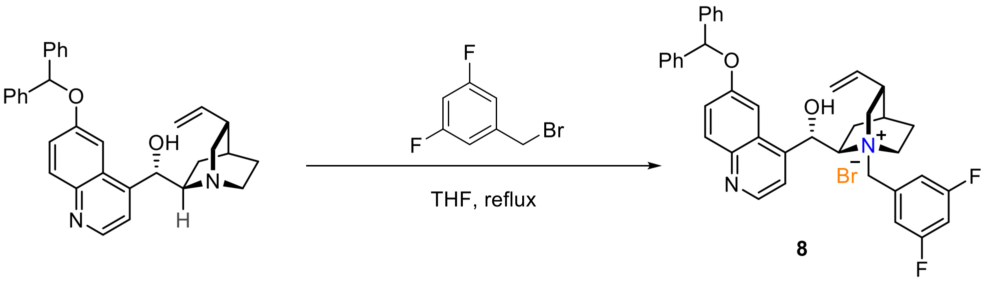

To a suspension of quinidine derivative[1] (843.0 mg, 1.82 mmol, 1.0 equiv.) in tetrahydrofuran (18.0 mL) was added 3, 5-trifluorobenzyl bromide (0.234 mL, 1.82 mmol, 1.0 equiv.). The mixture was refluxed overnight and cooled to room temperature to give a white precipitate. The solids were collected in a funnel and washed successively with cold tetrahydrofuran (20 mL), ethyl acetate (50 mL) and diethyl ether (50 mL). The resulting powder was dried under vacuum to give the catalyst (878.0 mg, 70.7% yield) as a white solid.

^{1}H NMR (400 MHz, DMSO) δ = 8.80 (d, J = 4.5 Hz, 1H), 8.02 (d, J = 9.2 Hz, 1H), 7.75 – 7.69 (m, 3H), 7.66 (dd, J = 9.3, 2.2 Hz, 1H), 7.63 – 7.57 (m, 3H), 7.57 – 7.51 (m, 2H), 7.46 (s, 1H), 7.40 (t, J = 7.5 Hz, 2H), 7.29 – 7.22 (m, 4H), 6.74 (s, 1H), 6.11 – 5.79 (m, 2H), 5.39 – 5.10 (m, 2H), 4.99 – 4.83 (m, 1H), 4.32 – 4.16 (m, 1H), 4.01 (s, 1H), 3.75 (s, 1H), 3.56 (t, J = 11.3 Hz, 1H), 3.06 (q, J = 9.9 Hz, 1H), 2.73 – 2.60 (m, 1H), 2.35 (t, J = 11.3 Hz, 1H), 1.90 (s, 1H), 1.83 – 1.63 (m, 2H), 1.25 – 1.06 (m, 1H), 0.97 – 0.74 ppm (m, 1H).

^{13}C NMR (126 MHz, DMSO) δ = 163.8 (d, J = 13.3 Hz), 161.8 (d, J = 13.3 Hz), 156.0, 148.1, 144.1, 143.5, 141.6 (d, J = 17.4 Hz), 137.5, 132.0, 131.8 (t, J = 9.9 Hz), 129.0 (d, J = 7.1 Hz), 128.1 (d, J = 14.7 Hz), 127.1, 126.9, 125.7, 122.6, 121.1, 117.6 (d, J = 5.8 Hz), 117.4 (d, J = 9.6 Hz), 106.5 (t, J = 25.7 Hz), 105.7, 80.4, 67.8, 65.0, 61.4, 56.5, 54.7, 37.1, 26.5, 23.4, 21.2 ppm.

HRMS (ESI$^+$) $C_{39}H_{37}F_2N_2O_2^+$ [M-Br]$^+$ requires 603.2818, found 603.2811.

3. General procedure for the synthesis of substrates.

N-Me-2-iodoaniline (or bromoaniline) derivatives[2] and benzeneboronic acid hemiesters[3] were synthesised according to literature procedures. The general scheme for the five-step synthesis of substrates from benzeneboronic acid hemiesters and 2-iodo (or bromo)-*N*-methylanilines is shown below.

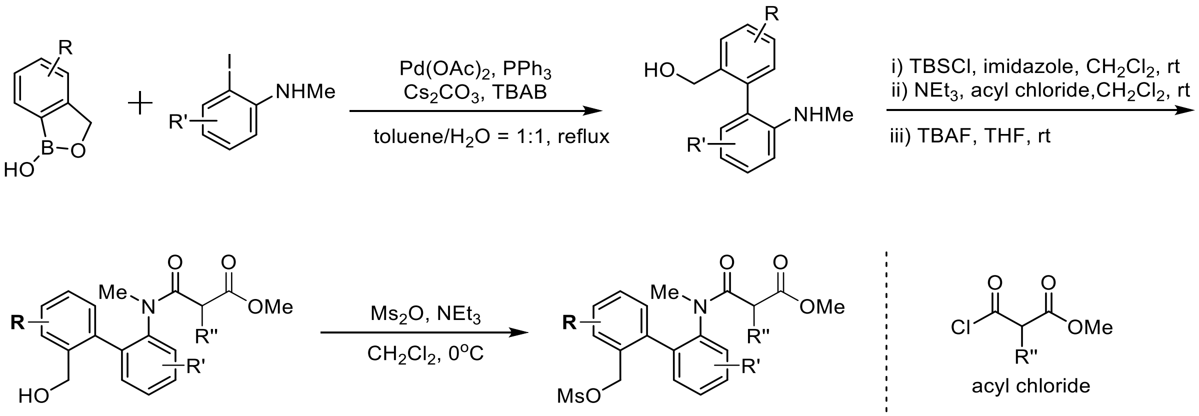

General Procedure A: Suzuki coupling of benzeneboronic acid hemiesters and 2-iodo-*N*-methylanilines.

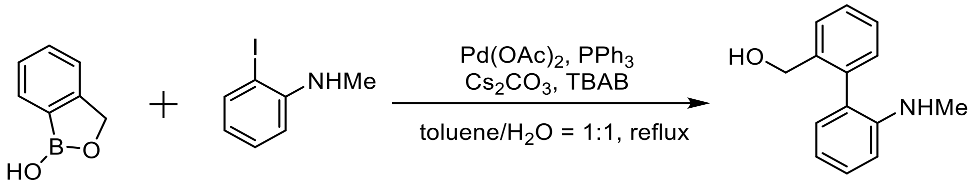

S1-1

To a solution of 2-iodo-*N*-methylaniline (3.0 g, 12.93 mmol, 1.2 equiv.) in toluene/H_2O (v/v = 1:1) were added sequentially benzeneboronic acid hemiester (1.44 g, 10.77 mmol, 1.0 equiv.), $Pd(OAc)_2$ (241.7 mg, 1.07 mmol, 0.1 equiv.), PPh_3 (1.13 g, 4.31 mmol, 0.4 equiv.), tetrabutylammonium bromide (344.8 mg, 1.07 mmol, 0.1 equiv.), and Cs_2CO_3 (8.77 g, 26.92 mmol, 2.5 equiv.). The resulting mixture was degassed and heated to reflux for 24 h. The solvent was removed under reduced pressure and the residue was purified by flash column chromatography (silica gel, 10% to 25% EtOAc in petroleum ether) to afford biaryl compound **S1-1** (1.73 g, 75.5% yield) as an oil.

^{1}H NMR (400 MHz, $CDCl_3$), δ = 7.65 – 7.55 (m, 1H), 7.48 – 7.39 (m, 2H), 7.36 (ddd, *J* = 8.2, 7.3, 1.5 Hz, 1H), 7.29 – 7.22 (m, 1H), 7.06 (dd, *J* = 7.4, 1.6 Hz, 1H), 6.88 (td, *J* = 7.4, 1.1 Hz, 1H), 6.80 (dd, *J* = 8.2, 1.1 Hz, 1H), 4.65 – 3.99 (m, 2H), 3.35 (s, 1H), 2.76 (s, 3H) ppm; **^{13}C NMR** (101 MHz, $CDCl_3$), δ = 146.2, 140.0, 137.6, 130.2, 129.5, 129.3, 128.9, 128.3, 128.3, 127.4, 117.9, 110.7, 63.6, 30.6 ppm; **FTIR (neat) v/cm^{-1}** 2980.67, 2360.62, 1577.80, 1288.63, 1002.89, 751.05; **HRMS** (ESI$^+$) $C_{14}H_{16}NO$ $[M+H]^+$ requires 214.1226; found 214.1227.

General Procedure B: Synthesis of amides.

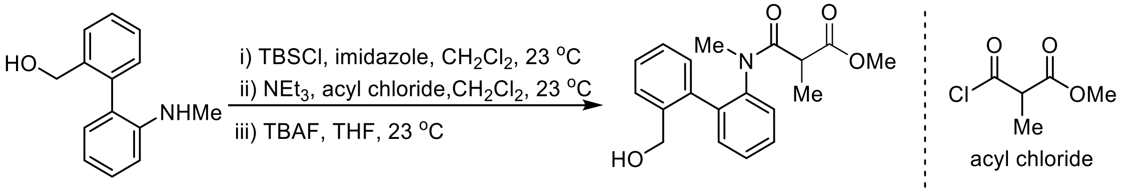

TBSCl (1.19 g, 7.94 mmol, 1.3 equiv.) and imidazole (581.4 mg, 8.55 mmol, 1.4 equiv.) were added to a solution of compound **S1-1** (1.31 g, 6.11 mmol, 1.0 equiv.) in THF (5 mL/mmol). Stirred at room temperature for 10 h. Then water was added, the aqueous phase was extracted with dichloromethane (2 x 20 mL). The combined organic layers were dried with Na_2SO_4, filtered and concentrated. The crude product was used for the next step without further purification.

Oxalyl chloride (1.68 g, 13.44 mmol, 2.2 equiv.) was added dropwise to a solution of corresponding acid (1.61 g, 12.22 mmol, 2.0 equiv.) and drops of DMF in dry dichloromethane at 0 °C. After 10 min the resulting mixture was warmed to room temperature and stirred for another 30 min. The remaining oxalyl and solvent were removed under reduced pressure to afford corresponding acyl chloride. To the solution of TBS-protected biaryl compound in dry dichloromethane, NEt_3 and freshly prepared oxalyl chloride were added sequentially, the reaction mixture was stirred for 2 h at room temperature. The reaction was quenched with water, the aqueous phase was extracted with dichloromethane (20 mL x 2). The combined organic layers were washed with brine, dried over Na_2SO_4, concentrated and the crude product TBS-amide was used for the next step without further purification.

To the solution of TBS-amide in THF was added TBAF (6.11 mL, 6.11 mmol, 1.0 equiv., 1.0 M in THF) at room temperature, the mixture was stirred for 2 h. The reaction mixture was concentrated and the residue was purified by flash column chromatography (silica gel, 20% to 50% EtOAc in petroleum, gradient elution) to afford compound **S1-2** (725.0 mg, 36.2% in 3 steps) as colorless oil.

^{1}H NMR (400 MHz, $CDCl_3$), δ = 7.53 – 7.39 (m, 1H), 7.28 – 7.05 (m, 6H), 7.03 – 6.78 (m, 1H), 4.54 – 3.88 (m, 2H), 3.78 – 3.37 (m, 3H), 3.34 – 2.92 (m, 2H), 2.90 – 2.42 (m, 2H), 1.62 – 0.44 (m, 3H) ppm; **^{13}C NMR** (101 MHz, $CDCl_3$), δ = 170.6, 170.4, 170.1, 169.8, 169.5, 169.3, 169.0, 141.4, 141.1, 140.7, 140.4, 140.2, 139.5, 138.8, 138.4, 138.3, 138.2, 137.6, 137.3, 137.1, 136.6, 135.8, 135.5, 135.4, 131.7, 131.5, 131.2, 131.1, 130.7, 130.5, 129.2, 129.0, 128.6, 128.3, 128.0,

127.8, 127.6, 127.6, 127.5, 127.3, 126.7, 126.6, 126.5, 126.2, 126.1, 125.7, 61.2, 61.1, 51.7, 51.6, 51.5, 43.0, 42.9, 42.4, 42.2, 40.0, 39.6, 39.4, 38.7, 35.6, 35.3, 35.2, 14.5, 13.9, 12.9, 11.9, 11.9 ppm**; FTIR (neat) ν/cm^{-1}** 2980.66, 2360.54, 1636.50, 1386.79, 1155.61, 955.04;

HRMS (ESI$^+$) $C_{19}H_{22}NO_4$ $[M+H]^+$ requires 328.1543; found 328.1544.

General Procedure C: Bromination or Mesylation of benzylic alcohol.

Compound 1:

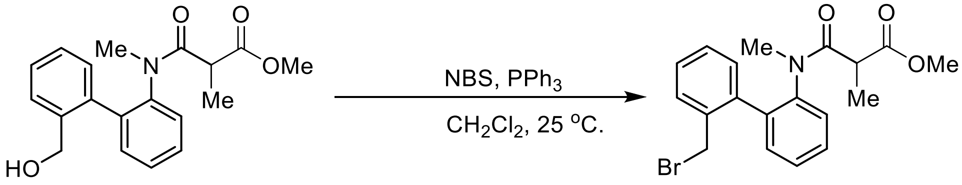

S1-2 **1**

To the solution of **S1-2** (490.5 mg, 1.50 mmol) in dichloromethane was added PPh_3 (473.0 mg, 1.80 mmol) and NBS (400.0 mg, 2.25 mmol) at 25°C. After 2 h the reaction was quenched with water. The aqueous layer was extracted with dichloromethane and the organic layer was washed with water, brine and dried over Na_2SO_4, then filtered and concentrated under reduced pressure. The crude material was purified by column chromatography to yield compound **1** as a white foam (439.3 mg, 75.3% yield).

^{1}H NMR (500 MHz, acetone - *d*^{6}) δ = 7.71 - 7.64 (m, 1H), 7.59 - 7.51 (m, 3H), 7.48 – 7.34 (m, 3H), 7.34 – 7.07 (m, 1H), 4.76 - 4.28 (m, 2H), 3.88 – 3.60 (m, 2H), 3.59 – 3.17 (m, 3H), 3.15 – 2.82 (m, 1H), 2.79 - 2.69 (m, 1H), 1.66 – 1.04 (m, 2H), 0.91 – 0.63 (m, 1H) ppm; **^{13}C NMR** (126 MHz, acetone - *d*^{6}) δ = 171.0, 170.8, 170.9, 170.5, 170.5, 170.4, 170.2, 170.1, 170.0, 169.5, 169.3, 168.9, 168.3, 142.7, 142.3, 142.1, 141.6, 141.6, 139.1, 139.0, 138.6, 138.5, 138.4, 138.1, 137.9, 137.7, 137.6, 137.5, 137.4, 137.3, 137.2, 137.1, 137.0, 136.5, 135.9, 135.8, 135.5, 135.4, 132.1, 131.6, 131.5, 131.5, 131.4, 131.3, 131.1, 130.9, 130.8, 130.7, 130.4, 130.2, 130.1, 130.0, 129.9, 129.8, 129.7, 129.7, 129.6, 129.6, 129.5, 129.4, 129.4, 129.2, 129.2, 129.1, 129.0, 128.9, 128.9, 128.8, 128.7, 128.6, 128.5, 128.4, 128.3, 128.2, 128.2, 128.1, 128.0, 127.9, 127.8, 127.7, 127.3, 127.2, 127.0, 126.9, 51.8, 51.8, 51.6, 51.6, 51.5, 51.1, 43.4, 43.4, 43.1, 42.6, 42.4, 42.4, 39.3, 38.8, 38.6, 38.6, 38.0, 37.9, 35.8, 35.4, 33.3, 33.0, 32.9, 32.0, 31.8, 31.7, 14.6, 14.3, 13.6, 13.3, 13.2, 13.1, 12.8, 12.3 ppm; **FTIR (neat) ν/cm^{-1}** 2980.85, 2360.56, 1745.94, 1655.63, 1476.17, 1383.65, 1207.69, 1006.74, 769.05;

HRMS (ESI$^+$) $C_{19}H_{21}BrNO_3$ $[M+H]^+$ requires 390.0699; 392.0679; found 390.0699; 392.0678.

Compound 5:

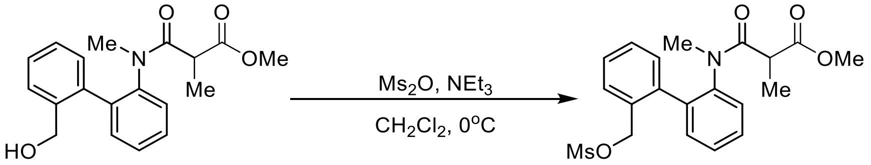

S1-2 **5**

A dichloromethane solution of methanesulfonic anhydride (446.8 mg, 2.56 mmol, 1.2 equiv.) was added dropwise to a solution of **S1-2** (700.0 mg, 2.14 mmol, 1.0 equiv.) and Et_3N (0.45 mL, 3.21 mmol, 1.5 equiv.) in dichloromethane at 0°C. After 2 h the reaction was quenched with water. The aqueous layer was extracted with dichloromethane. The organic layer was washed with water, brine and dried over Na_2SO_4, then filtered and concentrated under reduced pressure. The crude material was purified by column chromatography to afford 721.0 mg of compound **5** in 83.1% yield as a colourless oil.

^{1}H NMR (400 MHz, $CDCl_3$), δ = 7.57-7.51 (m, 1H), 7.37-7.31 (m, 5H), 7.24 – 6.89 (m, 2H), 5.52 – 4.47 (m, 2H), 4.08 – 3.38 (m, 3H), 3.37 – 2.94 (m, 2H), 2.97 – 2.14 (m, 5H), 1.39 – 1.25 (m, 2H), 0.71-0.73 (m, 1H) ppm; **^{13}C NMR** (101 MHz, $CDCl_3$), δ = 170.6, 170.3, 170.1, 170.0, 169.2, 168.4, 141.5, 141.4, 141.0, 140.8, 138.3, 137.7, 137.5, 137.4, 136.5, 136.2, 136.1, 132.3, 132.0, 131.5, 131.4, 131.2, 130.7, 130.6, 130.3, 130.1, 130.0, 129.4, 129.3, 129.1, 128.9, 128.9, 128.7, 128.5, 128.2, 128.0, 127.9, 127.9, 127.6, 127.2, 126.9, 126.8, 69.9, 68.7, 68.5, 68.1, 51.8, 51.8, 51.7, 43.2, 43.0, 42.7, 42.5, 42.4, 39.1, 38.9, 38.7, 37.0, 36.9, 36.8, 36.0, 35.8, 35.3, 14.6, 14.1, 13.8, 13.2, 12.3 ppm; **FTIR (neat) ν/cm^{-1}** 2980.76, 2360.72, 1653.13, 1354.16, 1207.45, 934.61; **HRMS** (ESI$^+$) $C_{20}H_{24}NO_6S$ $[M+H]^+$ requires 406.1319; found 406.1321.

Compound S9-1:

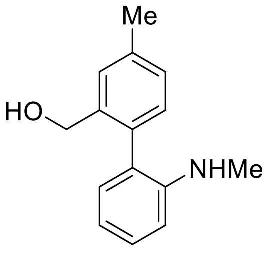

S9-1

Prepared according to General Procedure A with 2-iodo-*N*-methylaniline (814.0 mg, 3.49 mmol),

5-methylbenzo[c][1,2]oxaborol-1(3H)-ol (430.0 mg, 2.90 mmol), palladium acetate (65.3 mg, 0.29 mmol), triphenylphosphine (307.0 mg, 1.17 mmol), cesium carbonate (2.37 g, 7.26 mmol), and TBAB (94.0 mg, 0.29 mmol) in the mixed solvent of toluene and water (v/v = 1:1). Purification by column chromatography, eluting with 10% ethyl acetate in petrol, yielded intermediate **S9-1** as an oil (465.0 mg, 70.6% yield).

^{1}H NMR (400 MHz, $CDCl_3$), δ = 7.27 (s, 1H), 7.24 – 7.17 (m, 1H), 7.10 (d, J = 7.7 Hz, 1H), 7.01 (d, J = 7.6 Hz, 1H), 6.91 (dd, J = 7.4, 1.6 Hz, 1H), 6.74 (td, J = 7.4, 1.0 Hz, 1H), 6.66 (d, J = 8.1 Hz, 1H), 4.23 (dd, J = 61.8, 38.2 Hz, 2H), 3.12 (s, 2H), 2.62 (s, 3H), 2.32 (s, 3H) ppm; **^{13}C NMR** (101 MHz, $CDCl_3$), δ = 146.4, 139.8, 138.1, 134.6, 130.1, 130.1, 129.7, 129.0, 128.8, 123.9, 118.0, 110.7, 63.7, 30.6, 21.1 ppm; **FTIR (neat) ν/cm^{-1}** 2980.39, 2360.45, 1477.61, 1253.57, 1120.33, 790.05;

HRMS (ESI^+) $C_{15}H_{18}NO$ $[M+H]^+$ requires 228.1383; found 228.1382.

Compound S9-2:

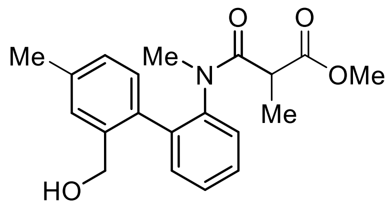

S9-2

Prepared according to General Procedure B with **S9-1** (450.0 mg, 1.98 mmol). Purification by column chromatography, gradient eluting with ethyl acetate in petrol (25% to 50%), yielded intermediate **S9-2** as an oil (569 mg, 84.3% yield in 3 steps).

^{1}H NMR (600 MHz, $CDCl_3$), δ = 7.39 - 7.36 (m, 2H), 7.32 - 7.29 (m, 1H), 7.26 - 7.20 (m, 1H), 7.19 – 7.04 (m, 2H), 7.02 – 6.80 (m, 1H), 4.66 – 4.06 (m, 1H), 3.79 -3.62 (m, 1H), 3.57 - 3.37 (m, 1H), 3.58 – 3.36 (m, 2H), 3.34 – 3.01 (m, 1H), 2.76 - 2.66 (m, 2H), 2.36 – 2.24 (m, 1H), 1.37 – 1.07 (m, 2H), 0.82 - 0.74 (m, 1H) ppm; **^{13}C NMR** (151 MHz, $CDCl_3$), δ = 172.3, 170.5, 167.9, 139.9, 139.7, 137.3, 137.2, 136.1, 135.9, 135.7, 134.8, 133.3, 133.0, 132.8, 132.6, 132.0, 131.3, 130.0, 129.5, 129.4, 129.2, 129.0, 128.6, 127.3, 127.0, 126.7, 126.6, 126.6, 126.4, 126.3, 126.1, 126.0, 125.8, 125.8, 125.5, 125.5, 125.3, 124.8, 124.7, 124.5, 124.4, 61.9, 60.3, 59.7, 59.0, 49.9, 49.8, 45.6, 41.3, 41.1, 40.6, 37.7, 37.6, 35.2, 33.8, 33.1, 18.7, 18.6, 18.6, 18.5, 18.5, 18.5, 11.1, 10.2, 9.5, 6.8 ppm; **FTIR (neat) ν/cm^{-1}** 2980.66, 2360.45, 1698.29, 1436.28, 1240.77, 1009.48;

HRMS (ESI$^+$) $C_{20}H_{24}NO_4$ [M+H]$^+$ requires 342.1711; found 342.1703.

Compound S9:

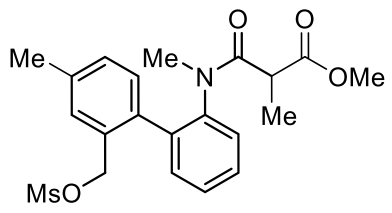

S9

Prepared according to General Procedure C with **S9-2** (569.0 mg, 1.67 mmol), methanesulfonic anhydride (350.0 mg, 2.00 mmol) and triethylamine (0.36 mL, 2.51 mmol). Purification by column chromatography, gradient eluting with ethyl acetate in petrol (25% to 50%), yielded product **S9** as an oil (180.0 mg, 25.7% yield).

^{1}H NMR (400 MHz, $CDCl_3$), δ = 7.54 – 7.23 (m, 5H), 7.18 – 6.68 (m, 2H), 5.34 – 4.62 (m, 2H), 3.67 - 3.61 (m, 2H), 3.57 – 3.26 (m, 2H), 3.30 – 2.53 (m, 7H), 2.47 – 2.16 (m, 4H), 1.62 – 1.05 (m, 2H), 0.82 – 0.77(m, 1H) ppm; **^{13}C NMR** (101 MHz, $CDCl_3$), δ = 171.0, 170.7, 170.5, 170.2, 169.8, 169.6, 168.7, 141.9, 141.5, 141.3, 138.6, 138.4, 137.9, 137.0, 136.7, 135.8, 135.6, 135.3, 135.1, 134.9, 132.3, 132.1, 131.7, 131.3, 131.0, 130.5, 130.3, 130.0, 129.6, 129.3, 129.2, 129.0, 128.8, 128.4, 128.2, 128.0, 127.6, 127.3, 127.2, 77.3, 77.0, 76.6, 70.4, 68.9, 68.7, 68.4, 64.2, 52.3, 52.1, 43.6, 43.4, 43.1, 43.0, 40.2, 39.3, 37.5, 37.4, 36.2, 35.8, 35.4, 20.9, 20.9, 20.8, 15.1, 14.4, 13.5, 12.4 ppm; **FTIR (neat) ν/cm^{-1}** 2980.74, 2360.37, 1653.68, 1353.77, 1174.10, 942.36;
HRMS (ESI$^+$) $C_{21}H_{26}NO_6S$ [M+H]$^+$ requires 420.1475; found 420.1477.

Compound S10-1:

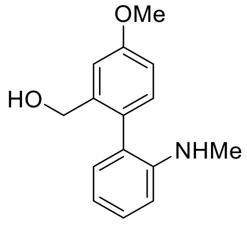

S10-1

Prepared according to General Procedure A with 2-iodo-*N*-methylaniline (1.03 g, 4.40 mmol), 5-methoxybenzo[c][1,2]oxaborol-1(3H)-ol (600.0 mg, 3.67 mmol), palladium acetate (83.0 mg, 0.36 mmol), triphenylphosphine (386.0 mg, 1.47 mmol), cesium carbonate (3.00 g, 9.18 mmol), and

TBAB (119.0 mg, 0.36 mmol) in the mixed solvent of toluene and water (v/v = 1:1). Purification by column chromatography, eluting with 10% ethyl acetate in petrol, yielded intermediate **S10-1** as an oil (300.0 mg, 37.0% yield).

^{1}H NMR (400 MHz, $CDCl_3$), δ = 7.21 (td, J = 7.9, 1.6 Hz, 1H), 7.07 – 6.96 (m, 2H), 6.91 (dd, J = 7.4, 1.6 Hz, 1H), 6.83 (dd, J = 8.3, 2.8 Hz, 1H), 6.73 (td, J = 7.4, 0.9 Hz, 1H), 6.65 (d, J = 8.1 Hz, 1H), 4.24 (q, J = 12.4 Hz, 2H), 3.77 (s, 3H), 3.19 (s, 2H), 2.64 (s, 3H) ppm; **^{13}C NMR** (101 MHz, $CDCl_3$), δ = 159.6, 146.7, 141.5, 131.3, 130.0, 129.6, 128.8, 127.1, 117.8, 114.3, 113.9, 110.5, 63.8, 55.2, 30.7 ppm; **FTIR (neat) ν/cm^{-1}** 2980.64, 2360.27, 1491.71, 1287.55, 1121.42, 750.08;

HRMS (ESI$^+$) $C_{15}H_{18}NO_2$ $[M+H]^+$ requires 244.1332; found 244.1333.

Compound S10-2:

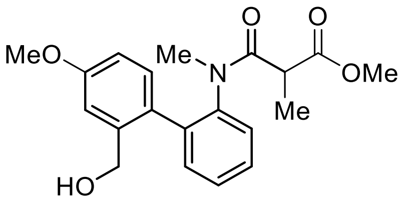

S10-2

Prepared according to General Procedure B with **S10-1** (300.0 mg, 1.23 mmol). Purification by column chromatography, gradient eluting with ethyl acetate in petrol (25% to 50%), yielded intermediate **S10-2** as an oil (350.0 mg, 75.2% yield in 3 steps).

^{1}H NMR (400 MHz, $CDCl_3$), δ = 7.40 – 7.28 (m, 3H), 7.27 – 7.19 (m, 1H), 7.19 – 6.89 (m, 2H), 6.77 (d, J = 6.0 Hz, 1H), 4.55 – 4.11 (m, 2H), 3.76 (d, J = 6.8 Hz, 3H), 3.68 – 3.45 (m, 3H), 3.39 – 2.98 (m, 3H), 2.70 (d, J = 40.0 Hz, 1H), 1.57 – 1.07 (m, 2H), 0.95 – 0.51 (m, 1H) ppm; **^{13}C NMR** (101 MHz, $CDCl_3$), δ = 171.0, 170.6, 170.4, 170.2, 169.6, 159.2, 159.2, 159.0, 141.9, 141.6, 141.2, 140.1, 138.4, 137.5, 132.4, 132.0, 131.6, 131.3, 130.6, 129.6, 129.3, 129.2, 128.6, 128.5, 128.1, 127.9, 127.8, 127.0, 126.7, 113.8, 113.0, 112.6, 112.3, 61.9, 61.8, 55.0, 54.9, 52.1, 52.1, 51.9, 43.4, 43.2, 43.0, 42.7, 39.8, 39.7, 38.9, 38.2, 36.0, 35.8, 14.9, 14.3, 13.8, 13.3, 12.5 ppm; **FTIR (neat) ν/cm^{-1}** 2980.66, 2360.22, 1609.71, 1256.90, 1101.65, 798.22;

HRMS (ESI$^+$) $C_{20}H_{23}NO_5Na$ $[M+Na]^+$ requires 380.1468; found 380.1472.

Compound S10:

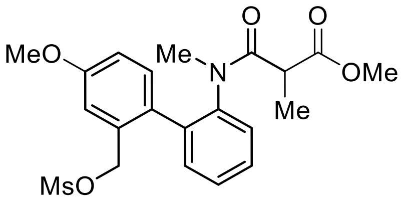

S10

Prepared according to General Procedure C with **S10-2** (350.0 mg, 1.02 mmol), methanesulfonic anhydride (214.0 mg, 1.23 mmol) and triethylamine (0.21 mL, 1.54 mmol). Purification by column chromatography, gradient eluting with ethyl acetate in petrol (25% to 50%), yielded product **S1** as an oil (440.0 mg, 99.1% yield).

^{1}H NMR (400 MHz, $CDCl_3$), δ = 7.56 – 7.35 (m, 2H), 7.35 – 7.28 (m, 1H), 7.28 – 7.18 (m, 1H), 7.18 – 6.96 (m, 2H), 6.93 – 6.80 (m, 1H), 4.97 (m, 5.06 – 4.87, 2H), 3.83 – 3.73 (m, 3H), 3.70 – 3.56 (m, 3H), 3.54 – 3.22 (m, 3H), 3.20 – 3.02 (m, 1H), 2.93 (d, J = 13.5 Hz, 1H), 2.79 (d, J = 25.7 Hz, 2H), 2.65 (s, 1H), 1.49 – 1.17 (m, 2H), 0.83 (dd, J = 18.3, 6.0 Hz, 1H) ppm; **^{13}C NMR** (101 MHz, $CDCl_3$), δ = 170.8, 170.5, 170.3, 169.4, 168.6, 159.2, 159.1, 158.9, 142.0, 141.9, 141.3, 141.2, 137.3, 136.6, 136.2, 136.1, 133.7, 132.1, 131.8, 131.3, 130.8, 130.5, 130.2, 130.0, 129.7, 129.1, 128.8, 128.6, 128.0, 127.9, 127.4, 127.1, 127.0, 115.4, 115.3, 114.5, 114.3, 113.9, 113.6, 70.0, 68.7, 68.5, 68.2, 59.9, 55.0, 55.0, 52.0, 52.0, 43.4, 43.2, 42.9, 42.7, 39.1, 39.0, 37.1, 36.1, 35.9, 35.5, 14.8, 14.3, 13.8, 13.4, 12.7 ppm; **FTIR (neat) ν/cm^{-1}** 2980.88, 2360.64, 1737.00, 1457.70, 1174.28, 939.45;

HRMS (ESI^{+}) $C_{21}H_{26}NO_7S$ $[M+H]^{+}$ requires 436.1424; found 436.1424.

Compound S11-1:

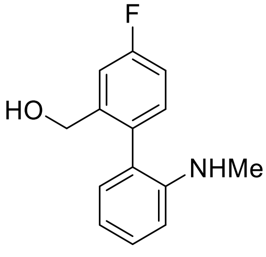

S11-1

Prepared according to General Procedure A with 2-iodo-*N*-methylaniline (920.0 mg, 3.95 mmol), benzo[c][1,2]oxaborol-1(3H)-ol (500.0 mg, 3.29 mmol), palladium acetate (74.0 mg, 0.33 mmol),

triphenylphosphine (345.0 mg, 1.32 mmol), cesium carbonate (2.70 g, 8.22 mmol), and TBAB (106.0 mg, 0.33 mmol) in the mixed solvent of toluene and water (v/v = 1:1). Purification by column chromatography, eluting with 10% ethyl acetate in petrol, yielded intermediate **S11-1** as an oil (700.0 mg, 92.1% yield).

^{1}H NMR (400 MHz, $CDCl_3$), δ = 7.21 (td, J = 8.1, 1.6 Hz, 1H), 7.17 (dd, J = 9.5, 2.7 Hz, 1H), 7.07 (dd, J = 8.4, 5.8 Hz, 1H), 6.96 (td, J = 8.3, 2.7 Hz, 1H), 6.87 (dd, J = 7.4, 1.6 Hz, 1H), 6.73 (td, J = 7.4, 1.0 Hz, 1H), 6.65 (d, J = 8.1 Hz, 1H), 4.47 – 3.72 (m, 2H), 3.19 (brs, 2H), 2.63 (s, 3H) ppm; **^{13}C NMR** (101 MHz, $CDCl_3$), δ = 162.6 (d, J = 247.0 Hz), 146.3, 142.5 (d, J = 7.0 Hz), 133.0 (d, J = 3.3 Hz), 131.8 (d, J = 7.9 Hz), 129.8, 129.1, 126.1, 117.9, 115.5 (d, J = 21.7 Hz), 114.8 (d, J = 21.1 Hz), 110.6, 63.0, 30.6 ppm; **^{19}F NMR** (377 MHz, $CDCl_3$), δ = -113.81 ppm; **FTIR (neat) ν/cm^{-1}** 2980.67, 2360.68, 1636.75, 1424.80, 1205.53, 1093.68;

HRMS (ESI^+) $C_{14}H_{15}FNO$ $[M+H]^+$ requires 232.1132; found 232.1133.

Compound S11-2:

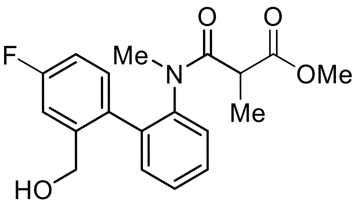

S11-2

Prepared according to General Procedure B with **S11-1** (700.0 mg, 3.03 mmol). Purification by column chromatography, gradient eluting with ethyl acetate in petrol (25% to 50%), yielded intermediate **S11-2** as an oil (550.0 mg, 47.8% yield in 3 steps).

^{1}H NMR (400 MHz, $CDCl_3$), δ = 7.42 – 7.33 (m, 2H), 7.30 – 7.23 (m, 2H), 7.17 – 7.11 (m, 1H), 6.95 – 6.87 (m, 1H), 4.58 – 3.98 (m, 2H), 3.74 – 3.55 (m, 2H), 3.55 – 3.36 (m, 2H), 3.33 – 2.96 (m, 2H), 2.87 – 2.60 (m, 1H), 2.01 (brs, 1H), 1.65 – 1.13 (m, 2H), 1.00 – 0.67 (m, 1H) ppm; **^{13}C NMR** (126 MHz, $CDCl_3$), δ = 171.2, 170.9, 170.7, 170.6, 170.3, 170.1, 169.8, 169.1, 163.7, 163.7, 163.4, 163.4, 161.8, 161.7, 161.5, 161.4, 143.5, 143.5, 143.4, 142.3, 142.1, 142.0, 141.5, 141.1, 140.8, 138.3, 138.2, 137.9, 137.4, 137.0, 133.2, 132.7, 132.3, 132.1, 132.1, 131.9, 131.7, 131.5, 131.0, 130.6, 130.2, 130.1, 129.8, 129.8, 129.4, 129.2, 129.0, 128.9, 128.6, 128.3, 128.3, 128.1, 127.5, 127.4, 127.2, 127.1, 116.7, 116.6, 116.4, 116.2, 115.5, 115.1, 114.9, 114.8, 114.7, 114.5, 114.3, 114.2, 114.0, 113.4, 113.2, 62.4, 62.3, 62.0, 52.6, 52.5, 52.4, 43.9, 43.6, 43.5, 43.3, 43.1,

40.1, 39.9, 39.5, 39.3, 36.8, 36.0, 15.2, 15.0, 14.6, 13.9, 13.7, 13.5, 12.8 ppm; **^{19}F NMR** (377 MHz, $CDCl_3$), δ = -112.9, -112.9, -113.0, -113.0, -113.0, -113.0, -113.0, -113.0, -113.0, -113.0, -113.0, -113.0, -113.1, -113.1, -113.2, -113.3, -113.3, -113.6, -113.6, -113.6, -113.8, -113.8, -113.9, -113.9, -114.0, -114.0, -114.1, -114.1, -114.5, -114.6, -114.6, -114.6 ppm; **FTIR (neat) ν/cm^{-1}** 2980.74, 2360.24, 1717.42, 1507.40, 1436.14, 1146.98;

HRMS (ESI$^+$) $C_{19}H_{21}FNO_4$ $[M+H]^+$ requires 346.1449; found 346.1451.

Compound 30:

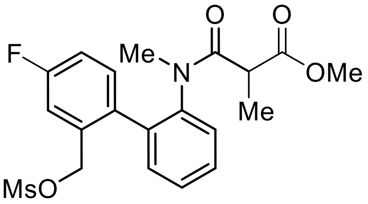

30

Prepared according to General Procedure C with **S11-2** (550.0 mg, 1.60 mmol), methanesulfonic anhydride (337.0 mg, 1.93 mmol) and triethylamine (0.34 mL, 2.42 mmol). Purification by column chromatography, gradient eluting with ethyl acetate in petrol (25% to 50%), yielded product **30** as an oil (640.0 mg, 94.5% yield).

^{1}H NMR (400 MHz, $CDCl_3$), δ = 7.64 – 7.33 (m, 2H), 7.32 – 7.16 (m, 3H), 7.16 – 6.89 (m, 2H), 5.19 – 4.56 (m, 2H), 3.90 – 3.55 (m, 2H), 3.55 – 3.28 (m, 2H), 3.26 – 3.01 (m, 1H), 2.97 – 2.88 (m, 1H), 2.84 (d, J = 2.6 Hz, 1H), 2.79 (s, 1H), 2.73 (s, 1H), 2.63 (s, 1H), 1.25 (ddd, J = 61.3, 39.0, 6.5 Hz, 2H), 0.79 (d, J = 6.3 Hz, 1H) ppm; **^{13}C NMR** (101 MHz, $CDCl_3$), δ = 171.0, 170.8, 170.6, 170.5, 170.2, 169.7, 168.9, 163.4, 163.3, 161.0, 160.9, 142.2, 142.1, 141.8, 141.6, 141.4, 141.3, 136.9, 136.5, 136.1, 135.8, 135.7, 135.5, 134.2, 133.9, 133.8, 133.6, 133.5, 132.5, 132.4, 132.1, 131.9, 131.8, 131.4, 131.2, 131.1, 130.9, 130.1, 129.9, 129.6, 129.5, 129.1, 128.6, 128.5, 128.4, 127.9, 127.6, 127.5, 117.2, 116.9, 116.8, 116.7, 116.6, 116.3, 116.2, 116.1, 115.9, 115.7, 115.5, 115.0, 114.8, 69.6, 69.4, 68.1, 67.9, 67.7, 52.4, 52.3, 52.2, 51.9, 43.8, 43.5, 43.3, 42.9, 39.5, 39.3, 37.5, 37.5, 37.4, 36.7, 36.4, 36.3, 35.9, 15.1, 14.6, 14.2, 13.7, 13.6, 12.9 ppm; **^{19}F NMR** (377 MHz, $CDCl_3$), δ = -112.02 – -112.21 (m), -112.26 (q, J = 8.9 Hz), -112.44 – -112.86 (m), -112.94 (q, J = 8.2 Hz), -113.36 (q, J = 8.2 Hz), -113.69 – -114.07 (m) ppm; **FTIR (neat) ν/cm^{-1}** 2980.82, 2360.71, 1654.47, 1355.38, 1175.04, 955.78;

HRMS (ESI$^+$) $C_{20}H_{23}FNO_6S$ $[M+H]^+$ requires 424.1225; found 424.1225.

Compound S12-1:

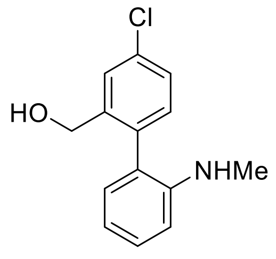

S12-1

Prepared according to General Procedure A with 2-iodo-*N*-methylaniline (1.0 g, 4.29 mmol), 5-chlorobenzo[c][1,2]oxaborol-1(3H)-ol (600.0 mg, 3.57 mmol), palladium acetate (80.9 mg, 0.36 mmol), triphenylphosphine (376.0 mg, 1.43 mmol), cesium carbonate (2.91 g, 8.43 mmol), and TBAB (117.0 mg, 0.36 mmol) in the mixed solvent of toluene and water (v/v = 1:1). Purification by column chromatography, eluting with 10% ethyl acetate in petrol, yielded intermediate **S12-1** as an oil (360.0 mg, 40.8% yield).

^{1}H NMR (400 MHz, $CDCl_3$), δ = 7.57 (d, J = 2.3 Hz, 1H), 7.40 – 7.28 (m, 2H), 7.15 (d, J = 8.1 Hz, 1H), 6.98 (dd, J = 7.4, 1.7 Hz, 1H), 6.85 (td, J = 7.4, 1.1 Hz, 1H), 6.78 (dd, J = 8.2, 1.1 Hz, 1H), 4.40 – 4.28 (m, 2H), 3.25 (brs, 2H), 2.75 (s, 3H) ppm; **^{13}C NMR** (101 MHz, $CDCl_3$), δ = 146.0, 142.0, 135.9, 134.2, 131.7, 129.6, 129.3, 129.2, 128.3, 126.2, 118.2, 111.0, 63.3, 30.8 ppm;

FTIR (neat) ν/cm^{-1} 2980.65, 2360.48, 1604.59, 1249.45, 1090.04, 955.03;

HRMS (ESI$^+$) $C_{14}H_{15}ClNO$ $[M+H]^+$ requires 248.0837; found 248.0837.

Compound S12-2:

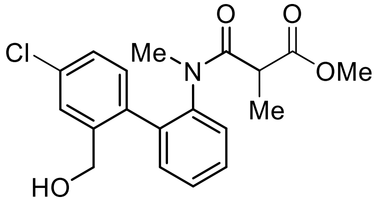

S12-2

Prepared according to General Procedure B with **S12-1** (330.0 mg, 1.34 mmol). Purification by column chromatography, gradient eluting with ethyl acetate in petrol (25% to 50%), yielded intermediate **S12-2** as an oil (219.0 mg, 45.2% yield in 3 steps).

^{1}H NMR (400 MHz, $CDCl_3$), δ = 7.68 – 7.49 (m, 1H), 7.42 – 7.32 (m, 2H), 7.31 – 7.24 (m, 1H), 7.24 – 7.14 (m, 2H), 7.13 – 6.80 (m, 1H), 4.50 – 4.09 (m, 2H), 3.73 – 3.37 (m, 4H), 3.33 – 2.97

(m, 2H), 2.72 (d, J = 39.2 Hz, 1H), 1.35 – 1.12 (m, 2H), 0.80 (d, J = 7.1 Hz, 1H) ppm; **^{13}C NMR** (101 MHz, $CDCl_3$), δ = 170.7, 170.4, 170.0, 169.7, 169.6, 142.2, 142.1, 141.3, 140.9, 140.6, 137.6, 137.4, 137.0, 136.4, 135.3, 134.2, 134.0, 133.9, 133.8, 133.4, 133.2, 131.9, 131.4, 130.9, 130.8, 130.3, 129.7, 129.5, 129.3, 129.1, 128.8, 128.4, 128.0, 127.9, 127.6, 127.2, 127.1, 126.8, 126.6, 126.1, 61.3, 61.2, 43.8, 43.2, 42.8, 42.7, 39.7, 39.1, 38.4, 36.0, 35.7, 14.8, 14.2, 13.8, 13.4, 13.3, 12.4 ppm; **FTIR (neat) ν/cm^{-1}** 2980.75, 2360.75, 1594.00, 1488.96, 1249.44, 955.85;
HRMS (ESI$^+$) $C_{19}H_{21}ClNO_4$ $[M+H]^+$ requires 362.1154; found 362.1155.

Compound S12:

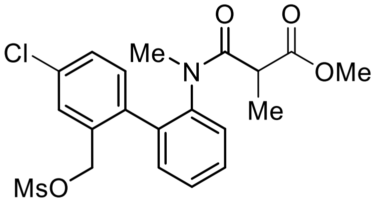

S12

Prepared according to General Procedure C with **S12-2** (219.0 mg, 0.60 mmol), methanesulfonic anhydride (127.0 mg, 0.72 mmol) and triethylamine (0.13 mL, 0.90 mmol). Purification by column chromatography, gradient eluting with ethyl acetate in petrol (25% to 50%), yielded product **S12** as an oil (225.0 mg, 84.5% yield).
^{1}H NMR (400 MHz, $CDCl_3$), δ = 7.57 – 7.44 (m, 1H), 7.44 – 7.34 (m, 2H), 7.33 – 7.23 (m, 2H), 7.23 – 7.16 (m, 1H), 7.12 – 6.88 (m, 1H), 5.43 – 4.67 (m, 2H), 3.73 – 3.56 (m, 2H), 3.55 – 3.29 (m, 2H), 3.23 – 3.10 (m, 1H), 2.93 (d, J = 12.8 Hz, 2H), 2.86 – 2.76 (m, 2H), 2.68 (d, J = 38.6 Hz, 1H), 1.48 – 1.11 (m, 2H), 0.81 (d, J = 6.6 Hz, 1H) ppm; **^{13}C NMR** (101 MHz, $CDCl_3$), δ = 170.7, 170.6, 170.2, 169.5, 168.7, 160.7, 141.7, 141.7, 141.2, 141.1, 141.0, 136.7, 136.3, 136.1, 135.9, 135.4, 135.3, 134.8, 134.6, 134.3, 134.2, 133.9, 133.8, 133.5, 133.1, 133.0, 132.8, 132.7, 132.1, 131.7, 131.6, 131.3, 131.0, 130.7, 130.5, 130.2, 129.8, 129.7, 129.5, 129.4, 129.0, 128.9, 128.6, 128.4, 128.3, 128.2, 128.0, 127.8, 127.6, 127.4, 127.2, 105.9, 69.2, 69.1, 67.6, 67.3, 67.1, 61.9, 52.1, 52.1, 52.0, 51.7, 48.3, 43.6, 43.3, 42.8, 39.2, 37.4, 37.3, 36.9, 36.2, 35.8, 29.3, 14.9, 14.4, 14.0, 13.6, 13.4, 12.7ppm; **FTIR (neat) ν/cm^{-1}** 2981.12, 2360.70, 1746.04, 1355.57, 1175.00, 938.15;
HRMS (ESI$^+$) $C_{20}H_{23}ClNO_6S$ $[M+H]^+$ requires 440.0929; found 440.0929.

Compound S13-1:

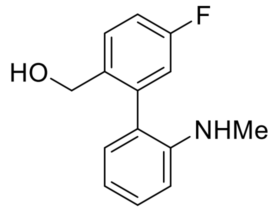

S13-1

Prepared according to General Procedure A with 2-iodo-*N*-methylaniline (1.1 g, 4.73 mmol), 6-fluorobenzo[c][1,2]oxaborol-1(3H)-ol (600.0 mg, 3.94 mmol), palladium acetate (89.0 mg, 0.39 mmol), triphenylphosphine (415.0 mg, 1.58 mmol), cesium carbonate (3.2 g, 9.85 mmol), and TBAB (127.3 mg, 0.39 mmol) in the mixed solvent of toluene and water (v/v = 1:1). Purification by column chromatography, eluting with 10% ethyl acetate in petrol, yielded intermediate **S13-1** as an oil (632.0 mg, 69.4% yield).

^{1}H NMR (400 MHz, $CDCl_3$), δ = 7.52 (dd, J = 8.4, 5.9 Hz, 1H), 7.38 – 7.29 (m, 1H), 7.10 (td, J = 8.4, 2.8 Hz, 1H), 7.01 (dd, J = 7.5, 1.6 Hz, 1H), 6.94 (dd, J = 9.2, 2.7 Hz, 1H), 6.85 (td, J = 7.5, 1.0 Hz, 1H), 6.77 (d, J = 8.2 Hz, 1H), 4.38 – 4.26 (m, 2H), 3.24 (brs, 1H), 2.75 (s, 3H) ppm; **^{13}C NMR** (101 MHz, $CDCl_3$), δ = 163.6, 161.1, 146.0, 139.8 (d, J = 7.9 Hz), 136.0 (d, J = 3.6 Hz), 131.2 (d, J = 8.7 Hz), 129.3, 126.3, 118.1, 117.1 (d, J = 21.1 Hz), 115.1 (d, J = 21.1 Hz), 110.9, 63.0, 30.7 ppm; **^{19}F NMR** (377 MHz, $CDCl_3$), δ = -113.9 (td, J = 9.0, 6.0 Hz) ppm; **FTIR (neat) v/cm^{-1}** 2980.75, 2360.50, 1602.94, 1289.12, 1046.81, 826.98;

HRMS (ESI^{+}) $C_{14}H_{15}FNO$ [M+H]$^{+}$ requires 232.1132; found 232.1134.

Compound S13-2:

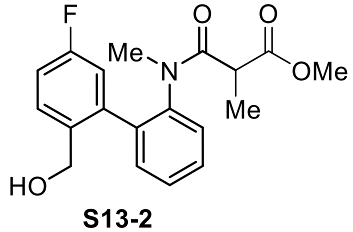

S13-2

Prepared according to General Procedure B with **S13-1** (600.0 mg, 2.59 mmol). Purification by column chromatography, gradient eluting with ethyl acetate in petrol (25% to 50%), yielded intermediate **S13-2** as an oil (488.0 mg, 54.4% yield in 3 steps).

^{1}H NMR (400 MHz, $CDCl_3$), δ = 7.49 (dd, J = 8.4, 5.9 Hz, 1H), 7.43 – 7.32 (m, 2H), 7.27 (dt, J = 14.5, 7.6 Hz, 1H), 7.19 – 7.12 (m, 1H), 7.09 – 6.59 (m, 2H), 4.58 – 4.02 (m, 2H), 3.71 - 3.62 (m, 2H), 3.57 – 3.34 (m, 2H), 3.38 - 3.21 (m, 2H), 2.80 - 2.15 (d, J = 42.4 Hz, 1H), 1.43 – 1.16 (m, 2H), 0.91 – 0.71 (m, 1H) ppm; **^{13}C NMR** (101 MHz, $CDCl_3$), δ = 170.6, 170.4, 170.2, 169.9, 169.6, 169.5, 162.2, 161.9, 161.7, 159.7, 159.5, 159.1, 141.1, 140.4, 138.7, 137.8, 137.7, 137.6, 137.2, 136.3, 136.2, 135.9, 134.6, 134.3, 131.7, 131.4, 131.2, 130.7, 130.5, 130.0, 129.8, 129.0, 128.7, 128.2, 127.8, 127.7, 127.0, 127.0, 126.7, 116.2, 116.0, 115.3, 114.6, 114.4, 114.1, 60.9, 60.9, 60.8, 59.9, 51.9, 51.9, 51.6, 51.6, 43.6, 42.6, 42.5, 39.7, 38.9, 38.1, 35.9, 35.5, 14.6, 14.0, 13.6, 13.2, 13.1, 12.2 ppm; **^{19}F NMR** (377 MHz, $CDCl_3$), δ = -114.73, -115.11, -115.53, -115.55, -115.65, -115.91, -115.93 ppm; **FTIR (neat) ν/cm^{-1}** 2980.74, 2360.87, 1750.01, 1456.42, 1180.25, 955.93;
HRMS (ESI$^+$) $C_{19}H_{21}FNO_4$ $[M+H]^+$ requires 346.1449; found 346.1451.

Compound S13:

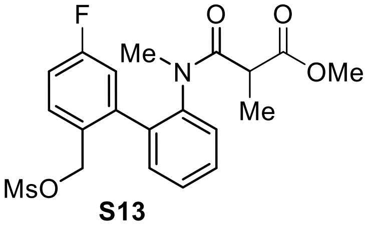

S13

Prepared according to General Procedure C with **S13-2** (488.0 mg, 1.41 mmol), methanesulfonic anhydride (297.0 mg, 1.70 mmol) and triethylamine (0.30 mL, 2.21 mmol). Purification by column chromatography, gradient eluting with ethyl acetate in petrol (25% to 50%), yielded product **S13** as an oil (477.0 mg, 79.9% yield).
^{1}H NMR (400 MHz, $CDCl_3$), δ = 7.55 – 7.45 (m, 1H), 7.45 – 7.34 (m, 2H), 7.33 – 7.20 (m, 2H), 7.14 – 7.02 (m, 1H), 6.91 – 6.66 (m, 1H), 5.12 – 4.67 (m, 2H), 3.79 – 3.61 (m, 2H), 3.58 – 3.31 (m, 2H), 3.28 – 3.12 (m, 1H), 2.92 (d, J = 12.0 Hz, 2H), 2.88 – 2.61 (m, 4H), 1.45 – 1.18 (m, 2H), 0.85 (d, J = 6.4 Hz, 1H) ppm; **^{13}C NMR** (101 MHz, $CDCl_3$), δ = 170.6, 170.5, 170.1, 170.1, 169.3, 168.7, 163.5, 163.3, 162.9, 161.1, 160.8, 141.5, 141.0, 140.8, 140.1, 140.1, 136.4, 135.7, 135.3, 135.2, 133.0, 132.6, 132.5, 132.4, 131.5, 131.1, 130.9, 130.5, 129.8, 129.5, 129.3, 128.8, 128.2, 128.2, 127.8, 127.6, 127.4, 127.1, 127.0, 126.7, 126.7, 117.3, 117.1, 116.6, 116.3, 116.1, 115.5, 115.4, 115.3, 115.2, 114.8, 114.6, 69.3, 68.0, 67.7, 67.5, 52.0, 51.9, 51.7, 43.3, 43.1, 42.9, 42.6, 39.2, 39.0, 37.2, 37.1, 37.0, 36.3, 36.0, 35.5, 14.6, 14.1, 13.4, 13.2, 12.5 ppm; **^{19}F NMR** (377 MHz,

$CDCl_3$), δ = -108.70 – -114.17 (m) ppm; **FTIR (neat) ν/cm^{-1}** 2981.05, 2360.84, 1746.12, 1354.39, 1174.02, 968.40;

HRMS (ESI$^+$) $C_{20}H_{23}FNO_6S$ [M+H]$^+$ requires 424.1225; found 424.1227.

Compound S14-1:

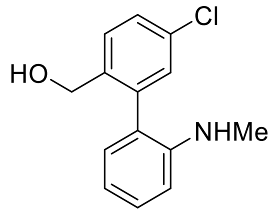

S14-1

Prepared according to General Procedure A with 2-iodo-*N*-methylaniline (1.33 g, 5.71 mmol), 6-chlorobenzo[c][1,2]oxaborol-1(3H)-ol (800.0 mg, 4.76 mmol), palladium acetate (107.0 mg, 0.47 mmol), triphenylphosphine (500.0 mg, 1.90 mmol), cesium carbonate (3.88 g, 11.90 mmol), and TBAB (154.0 mg, 0.47 mmol) in the mixed solvent of toluene and water (v/v = 1:1). Purification by column chromatography, eluting with 10% ethyl acetate in petrol, yielded intermediate **S14-1** as an oil (783.0 mg, 66.5% yield). **^{1}H NMR** (400 MHz, $CDCl_3$), δ = 7.51 (d, *J* = 8.2 Hz, 1H), 7.41 (dd, *J* = 8.3, 2.3 Hz, 1H), 7.35 – 7.32 (m, 1H), 7.25 (d, *J* = 2.2 Hz, 1H), 7.02 (dd, *J* = 7.5, 1.5 Hz, 1H), 6.88 (td, *J* = 7.4, 1.0 Hz, 1H), 6.83 – 6.78 (m, 2H), 4.56 – 3.97 (m, 2H), 3.26 (s, 0H), 2.77 (s, 3H) ppm; **^{13}C NMR** (101 MHz, $CDCl_3$), δ = 145.9, 139.4, 138.6, 133.9, 130.8, 130.2, 129.5, 129.4, 128.5, 126.2, 118.3, 111.1, 63.1, 30.8 ppm; **FTIR (neat) ν/cm^{-1}** 2980.69, 2360.36, 1558.46, 1473.56, 1250.31, 954.61;

HRMS (ESI$^+$) $C_{14}H_{15}ClNO$ [M+H]$^+$ requires 248.0837; found 248.0838.

Compound S14-2:

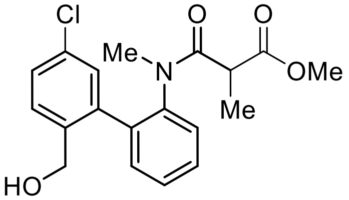

S14-2

Prepared according to General Procedure B with **S14-1** (770.0 mg, 3.11 mmol). Purification by column chromatography, gradient eluting with ethyl acetate in petrol (25% to 50%), yielded intermediate **S14-2** as an oil (546.0 mg, 48.6% yield in 3 steps).

^{1}H NMR (400 MHz, $CDCl_3$), δ = 7.56 - 7.45 (m, 1H), 7.41 - 7.33 (m, 2H), 7.31 - 7.23 (m, 2H), 7.20 – 7.08 (m, 1H), 7.07 – 6.82 (m, 1H), 4.48 – 4.11 (m, 2H), 3.78 – 3.59 (m, 2H), 3.57 – 3.38 (m, 2H), 3.34 – 3.11 (m, 2H), 2.82 – 2.64 (m, 1H), 2.38 (s, 1H), 1.48 – 1.16 (m, 2H), 0.93 – 0.71 (m, 1H) ppm; **^{13}C NMR** (101 MHz, $CDCl_3$), δ = 170.6, 170.4, 170.3, 169.9, 169.5, 141.1, 140.6, 140.3, 138.4, 137.5, 137.3, 137.0, 136.3, 136.0, 132.5, 131.8, 131.3, 131.1, 130.8, 130.5, 130.2, 129.4, 129.2, 129.1, 128.8, 128.6, 128.3, 127.9, 127.6, 127.5, 127.1, 127.0, 126.7, 61.0, 60.9, 60.9, 52.6, 52.1, 52.0, 43.1, 42.6, 39.8, 39.6, 38.9, 37.7, 36.0, 35.6, 14.9, 14.0, 13.4, 13.2, 13.2, 12.2 ppm; **FTIR (neat) ν/cm^{-1}** 2980.69, 2360.37, 1716.85, 1386.95, 1154.38, 955.21;
HRMS (ESI^{+}) $C_{19}H_{21}ClNO_4$ $[M+H]^{+}$ requires 362.1154; found 362.1154.

Compound S14:

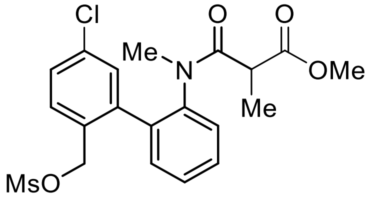

S14

Prepared according to General Procedure C with **S14-2** (521.0 mg, 1.44 mmol), methanesulfonic anhydride (302.0 mg, 1.73 mmol) and triethylamine (0.31 mL, 2.16 mmol). Purification by column chromatography, gradient eluting with ethyl acetate in petrol (25% to 50%), yielded product **S14** as an oil (531.0 mg, 83.9% yield). **^{1}H NMR** (400 MHz, $CDCl_3$), δ = 7.57 – 7.43 (m, 1H), 7.43 – 7.35 (m, 2H), 7.35 – 7.26 (m, 2H), 7.26 – 6.93 (m, 2H), 4.99 - 4.82 (m, 2H), 3.74 – 3.22 (m, 4H), 3.21 – 3.01 (m, 1H), 2.88 (d, J = 12.0 Hz, 1H), 2.83 – 2.52 (m, 4H), 1.56 – 1.08 (m, 2H), 0.91 – 0.64 (m, 1H) ppm; **^{13}C NMR** (101 MHz, $CDCl_3$), δ = 170.5, 170.4, 170.2, 170.1, 170.0, 170.0,

169.8, 169.2, 168.5, 141.4, 141.0, 140.8, 140.6, 140.1, 139.4, 139.2, 136.2, 135.6, 135.2, 134.9, 134.7, 134.5, 133.3, 131.9, 131.6, 131.6, 131.5, 131.3, 131.0, 130.8, 130.4, 129.8, 129.6, 129.5, 129.5, 129.3, 129.2, 129.0, 128.8, 128.4, 128.3, 128.2, 128.1, 127.6, 127.1, 69.6, 67.8, 67.5, 67.3, 52.2, 52.0, 51.9, 51.9, 51.8, 43.1, 42.9, 42.5, 39.1, 38.6, 37.2, 37.1, 37.0, 36.0, 35.5, 15.3, 14.1, 13.5, 13.2, 12.4 ppm; **FTIR (neat) ν/cm^{-1}** 2981.09, 2360.56, 1745.50, 1384.24, 1207.41, 930.44; **HRMS** (ESI$^+$) $C_{20}H_{23}ClNO_6S$ [M+H]$^+$ requires 440.0929; found 440.0931.

Compound S15-1:

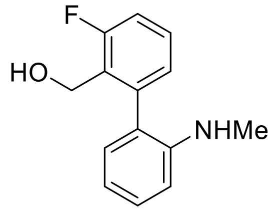

S15-1

Prepared according to General Procedure A with 2-iodo-*N*-methylaniline (920.0 mg, 3.95 mmol), 4-fluorobenzo[c][1,2]oxaborol-1(3H)-ol (500.0 mg, 3.29 mmol), palladium acetate (74.0 mg, 0.32 mmol), triphenylphosphine (345.0 mg, 1.32 mmol), cesium carbonate (2.70 g, 8.22 mmol), and TBAB (106.0 mg, 0.32 mmol) in the mixed solvent of toluene and water (v/v = 1:1). Purification by column chromatography, eluting with 10% ethyl acetate in petrol, yielded intermediate **S15-1** as an oil (560.0 mg, 73.5% yield).

^{1}H NMR (400 MHz, $CDCl_3$), δ = 7.45 – 7.32 (m, 2H), 7.21 – 7.11 (m, 1H), 7.06 (dd, J = 7.5, 1.4 Hz, 1H), 6.91 (td, J = 7.4, 1.1 Hz, 1H), 6.83 (d, J = 8.1 Hz, 1H), 4.62 (dd, J = 11.8, 1.3 Hz, 1H), 4.28 (dd, J = 11.8, 2.5 Hz, 1H), 3.44 (s, 2H), 2.78 (s, 3H) ppm; **^{13}C NMR** (101 MHz, $CDCl_3$), δ = 163.0, 160.5, 146.2, 140.7, 140.7, 130.0, 129.9, 129.7, 129.3, 127.5, 127.3, 126.7, 126.7, 125.9, 125.9, 118.4, 115.4, 115.2, 111.2, 77.3, 77.0, 76.6, 56.9, 56.9, 30.8 ppm; **^{19}F NMR** (377 MHz, $CDCl_3$), δ = -117.08 ppm; **FTIR (neat) ν/cm^{-1}** 2980.71, 2360.32, 1577.66, 1456.67, 1239.10, 795.61;

HRMS (ESI$^+$) $C_{14}H_{15}FNO$ [M+H]$^+$ requires 232.1132; found 232.1133.

Compound S15-2:

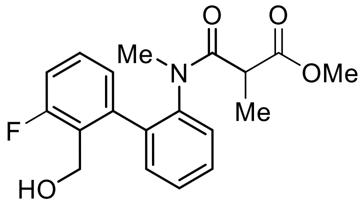

S15-2

Prepared according to General Procedure B with **S15-1** (560.0 mg, 2.42 mmol). Purification by column chromatography, gradient eluting with ethyl acetate in petrol (25% to 50%), yielded intermediate **S15-2** as an oil (340 mg, 41.2% in 3 steps).

^{1}H NMR (400 MHz, $CDCl_3$), δ = 7.62 – 7.36 (m, 2H), 7.36 – 7.19 (m, 3H), 7.17 – 7.02 (m, 1H), 7.02 – 6.73 (m, 1H), 4.82 – 4.05 (m, 2H), 3.77 – 3.45 (m, 3H), 3.44 – 3.30 (m, 2H), 3.24 (d, J = 7.6 Hz, 1H), 2.86 – 2.56 (m, 1H), 1.59 – 1.07 (m, 2H), 0.80 (d, J = 6.8 Hz, 1H) ppm; **^{13}C NMR** (101 MHz, $CDCl_3$), δ = 170.8, 170.5, 170.4, 170.3, 170.0, 169.9, 169.5, 169.4, 169.0, 163.2, 163.0, 160.7, 160.5, 141.8, 141.4, 141.4, 141.1, 140.8, 140.8, 139.9, 139.7, 139.4, 139.4, 137.7, 137.4, 136.8, 136.4, 136.2, 132.5, 132.2, 132.1, 131.7, 131.0, 130.8, 129.2, 129.0, 128.9, 128.6, 128.5, 128.2, 128.1, 127.8, 127.7, 127.7, 127.5, 127.3, 127.0, 126.9, 126.7, 126.0, 125.8, 125.6, 125.0, 124.5, 124.3, 124.0, 114.8, 114.7, 114.6, 114.5, 114.4, 114.1, 55.7, 55.5, 52.0, 51.8, 51.8, 51.4, 43.4, 43.1, 43.0, 42.8, 42.7, 42.5, 40.0, 39.7, 39.6, 38.9, 36.1, 35.8, 35.4, 14.8, 14.2, 13.7, 13.2, 13.0, 12.3 ppm; **^{19}F NMR** (377 MHz, $CDCl_3$), δ = -114.82 – -115.55 (m), -115.69 – -115.99 (m), -116.10 – -116.31 (m), -116.29 – -116.60 (m), -116.86 – -117.20 (m), -117.22 – -117.43 (m) ppm; **FTIR (neat) ν/cm^{-1}** 2980.66, 2360.45, 1683.76, 1489.16, 1249.77, 955.12;

HRMS (ESI^+) $C_{19}H_{21}FNO_4$ $[M+H]^+$ requires 346.1449; found 346.1451.

Compound S15:

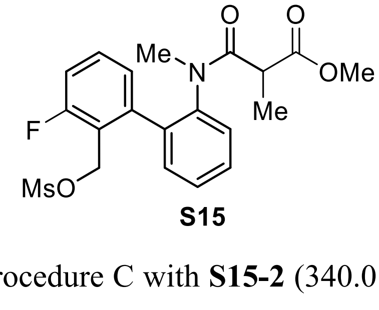

S15

Prepared according to General Procedure C with **S15-2** (340.0 mg, 0.99 mmol), methanesulfonic anhydride (209.1mg, 1.20 mmol) and triethylamine (0.22 mL, 1.50 mmol). Purification by column

chromatography, gradient eluting with ethyl acetate in petrol (25% to 50%), yielded product **S15** as an oil (320.0 mg, 76.4% yield).

^{1}H NMR (400 MHz, $CDCl_3$), δ = 7.48 – 7.17 (m, 5H), 7.10 – 7.04 (m, 1H), 7.02 – 6.74 (m, 1H), 5.36 – 5.09 (m, 1H), 5.01 – 4.56 (m, 1H), 3.63 (d, J = 1.9 Hz, 1H), 3.58 – 3.28 (m, 3H), 3.24 – 3.12 (m, 1H), 3.10 – 2.79 (m, 3H), 2.76 – 2.48 (m, 2H), 1.51 – 1.00 (m, 2H), 0.81 – 0.72 (q, J = 7.3, 6.8 Hz, 1H) ppm; **^{13}C NMR** (101 MHz, $CDCl_3$), δ = 170.7, 170.5, 170.2, 169.9, 169.7, 169.4, 168.7, 163.5, 163.4, 163.1, 163.0, 162.7, 161.0, 160.6, 160.6, 160.2, 142.2, 141.7, 141.4, 141.2, 141.0, 140.6, 136.4, 136.0, 135.5, 135.1, 135.0, 132.1, 131.8, 131.7, 131.4, 131.3, 131.1, 131.0, 130.9, 130.8, 130.7, 130.6, 130.0, 129.9, 129.8, 129.5, 129.4, 128.8, 128.5, 128.3, 128.2, 128.1, 128.1, 127.7, 127.1, 126.9, 126.1, 125.5, 125.0, 124.7, 120.0, 119.1, 118.7, 118.5, 118.4, 118.3, 118.1, 115.3, 115.1, 115.1, 114.9, 114.7, 114.5, 63.9, 63.5, 62.9, 62.5, 62.5, 52.1, 51.9, 51.9, 51.6, 45.8, 43.4, 43.2, 42.9, 42.7, 42.5, 40.5, 39.2, 39.1, 39.0, 37.0, 36.9, 36.7, 36.7, 36.2, 36.0, 35.8, 35.5, 33.4, 28.0, 23.5, 20.4, 17.1, 16.8, 14.8, 14.3, 13.9, 13.3, 12.6 ppm; **^{19}F NMR** (377 MHz, $CDCl_3$), δ = -113.20 – -113.58 (m), -113.83 (dd, J = 10.2, 6.7 Hz), -114.18 – -114.62 (m), -114.64 – -114.92 (m), -115.14, -115.48 – -116.09 (m) ppm; **FTIR (neat) ν/cm^{-1}** 2980.93, 2360.28, 1654.60, 1355.57, 1205.94, 932.66;

HRMS (ESI$^+$) $C_{20}H_{23}FNO_6S$ $[M+H]^+$ requires 424.1225; found 424.1226.

Compound S16-1:

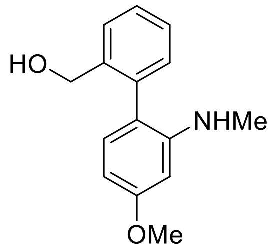

S16-1

Prepared according to General Procedure A with 5-methoxyl-2-bromo-*N*-methylaniline (1.35 g, 6.27 mmol), benzo[c][1,2]oxaborol-1(3H)-ol (700.0 mg, 5.22 mmol), palladium acetate (117.2 mg, 0.52 mmol), triphenylphosphine (549.0 mg, 2.09 mmol), cesium carbonate (4.26 g, 13.05 mmol), and TBAB (169.0 mg, 0.52 mmol) in the mixed solvent of toluene and water (v/v = 1:1). Purification by column chromatography, eluting with 10% ethyl acetate in petrol, yielded intermediate **S16-1** as an oil (799.0 mg, 62.9 % yield). **^{1}H NMR** (400 MHz, $CDCl_3$), δ = 7.54 (dd,

J = 7.2, 1.7 Hz, 1H), 7.39 (pd, J = 7.4, 1.7 Hz, 2H), 7.20 (dd, J = 7.2, 1.6 Hz, 1H), 6.94 (d, J = 8.2 Hz, 1H), 6.39 (dd, J = 8.2, 2.4 Hz, 1H), 6.32 (d, J = 2.4 Hz, 1H), 4.54 – 4.24 (m, 2H), 3.85 (s, 3H), 2.73 (s, 3H) ppm; **^{13}C NMR** (101 MHz, $CDCl_3$), δ = 160.6, 147.5, 140.5, 137.4, 130.8, 130.2, 129.3, 128.3, 128.3, 120.2, 101.9, 97.7, 63.8, 55.1, 30.6 ppm; **FTIR (neat) ν/cm^{-1}** 2980.61, 2360.42, 1519.92, 1339.23, 1171.77, 767.13;

HRMS (ESI$^+$) $C_{15}H_{18}NO_2$ $[M+H]^+$ requires 244.1332; found 244.1332.

Compound S16-2:

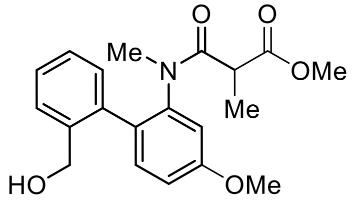

S16-2

Prepared according to General Procedure B with **S16-1** (779.0mg, 3.20 mmol). Purification by column chromatography, gradient eluting with ethyl acetate in petrol (25% to 50%), yielded intermediate **S16-2** as an oil (530.0 mg, 46.3% yield in 3 steps). **^{1}H NMR** (400 MHz, $CDCl_3$), δ = 7.51 (t, J = 10.0 Hz, 1H), 7.35 – 7.14 (m, 3H), 7.11 – 6.95 (m, 1H), 6.99 – 6.66 (m, 2H), 5.04 – 4.10 (m, 2H), 3.87 – 3.74 (m, 3H), 3.70 – 3.43 (m, 4H), 3.40 – 3.05 (m, 2H), 2.84 – 2.47 (m, 1H), 1.55 – 1.08 (m, 2H), 1.00 – 0.46 (m, 1H) ppm; **^{13}C NMR** (101 MHz, $CDCl_3$), δ = 171.0, 170.5, 170.2, 170.1, 169.5, 159.6, 159.3, 142.3, 142.2, 141.6, 140.3, 138.8, 138.6, 136.7, 135.8, 135.6, 132.8, 132.2, 131.8, 131.5, 130.9, 130.7, 129.8, 129.5, 128.9, 128.5, 128.0, 127.8, 127.6, 127.5, 127.4, 126.7, 126.5, 126.0, 113.8, 113.5, 113.0, 112.7, 112.1, 61.9, 61.8, 55.1, 55.1, 52.2, 52.0, 51.9, 43.4, 43.2, 42.9, 42.6, 39.8, 38.8, 35.7, 25.3, 14.9, 14.2, 13.3, 12.2 ppm; **FTIR (neat) ν/cm^{-1}** 2980.66, 2360.42, 1652.87, 1488.59, 1249.31, 954.86;

HRMS (ESI$^+$) $C_{20}H_{24}NO_5$ $[M+H]^+$ requires 358.1649; found 358.1650.

Compound S16:

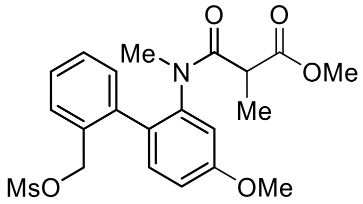

S16

Prepared according to General Procedure C with **S16-2** (530 mg, 1.48 mmol), methanesulfonic anhydride (310.0 mg, 1.78 mmol) and triethylamine (0.32 mL, 2.22 mmol). Purification by column chromatography, gradient eluting with ethyl acetate in petrol (25% to 50%), yielded product **S16** as an oil (500.0 mg, 77.6% yield). **^{1}H NMR** (400 MHz, $CDCl_3$), δ = 7.57 – 7.37 (m, 1H), 7.36 – 7.26 (m, 2H), 7.24 – 7.01 (m, 2H), 6.95 - 6.89 (m, 1H), 6.87 – 6.67 (m, 1H), 5.08 - 4.88 (m, 2H) 3.73 (dd, J = 12.4, 3.9 Hz, 3H), 3.66 – 3.42 (m,3H), 3.39 – 3.01 (m, 2H), 2.99 – 2.52 (m, 5H), 1.49 – 1.09 (m, 2H), 0.81 - 0.69 (m, 1H) ppm; **^{13}C NMR** (101 MHz, $CDCl_3$), δ = 170.7, 170.2, 170.1, 169.8, 169.2, 168.5, 159.8, 159.7, 159.5, 159.4, 142.5, 142.4, 142.1, 141.7, 138.1, 137.6, 137.4, 132.8, 132.4, 132.2, 132.0, 131.3, 131.0, 130.7, 130.5, 130.1, 130.0, 129.8, 129.5, 128.9, 128.7, 128.1, 128.0, 127.8, 127.5, 114.2, 113.9, 113.7, 113.6, 113.1, 112.9, 112.6, 70.0, 68.8, 68.6, 68.3, 67.6, 55.1, 55.0, 55.0, 51.9, 51.8, 51.7, 43.3, 43.1, 42.7, 42.6, 42.4, 39.1, 38.7, 37.0, 36.2, 35.6, 35.3, 14.8, 14.2, 13.2, 12.3 ppm; **FTIR (neat) ν/cm^{-1}** 2980.73, 2360.88, 1655.35, 1353.49, 1173.88, 886.46;

HRMS (ESI^{+}) $C_{21}H_{26}NO_7S$ $[M+H]^+$ requires 436.1424; found 436.1427.

Compound S17-1:

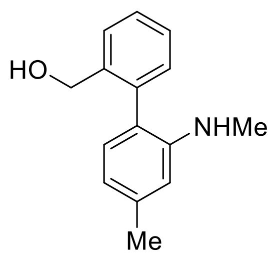

S17-1

Prepared according to General Procedure A with 5-methyl-2-iodo-*N*-methylaniline (1.48 g, 6.00 mmol), benzo[c][1,2]oxaborol-1(3H)-ol (670.0 mg, 5.0 mmol), palladium acetate (112.3 mg, 0.50 mmol), triphenylphosphine (525.0 mg, 2.00 mmol), cesium carbonate (4.08g, 12.50 mmol), and TBAB (162.0 mg, 0.50 mmol) in the mixed solvent of toluene and water (v/v = 1:1). Purification

by column chromatography, eluting with 10% ethyl acetate in petrol, yielded intermediate **S17-1** as an oil (711.0 mg, 62.6% yield). **^{1}H NMR** (400 MHz, $CDCl_3$), δ = 7.44 (dd, J = 7.2, 1.7 Hz, 1H), 7.34 – 7.26 (m, 2H), 7.11 (dd, J = 7.1, 1.7 Hz, 1H), 6.82 (d, J = 7.5 Hz, 1H), 6.59 (ddd, J = 7.5, 1.4, 0.6 Hz, 1H), 6.50 (s, 1H), 4.34 – 4.16 (m, 2H), 3.17 (s, 2H), 2.63 (s, 3H), 2.31 (s, 3H) ppm; **^{13}C NMR** (101 MHz, $CDCl_3$), δ = 146.1, 140.2, 138.8, 137.7, 130.5, 129.4, 129.4, 128.3, 128.3, 124.8, 118.7, 111.6, 63.8, 30.7, 21.6 ppm; **FTIR (neat) ν/cm^{-1}** 2980.81, 2360.58, 1575.89, 1457.18, 1429.68, 954.48;

HRMS (ESI$^+$) $C_{15}H_{18}NO$ $[M+H]^+$ requires 228.1383; found 228.1384.

Compound S17-2:

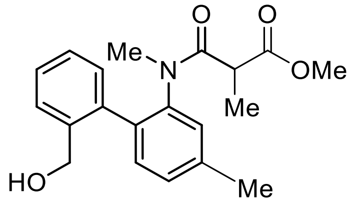

S17-2

Prepared according to General Procedure B with **S17-1** (670.0 mg, 2.95 mmol). Purification by column chromatography, gradient eluting with ethyl acetate in petrol (25% to 50%), yielded intermediate **S17-2** as an oil (490.0 mg, 48.7% yield in 3 steps).

^{1}H NMR (400 MHz, $CDCl_3$), δ = 7.59 - 7.41 (m, 1H), 7.34 - 7.26 (m, 1H), 7.26 – 7.00 (m, 4H), 7.03 – 6.82 (m, 1H), 4.53 – 4.14 (m, 2H), 3.67 - 3.61 (m, 1H), 3.54 - 3.47 (m, 1H), 3.45 – 3.23 (m, 2H), 3.19 - 2.96 (m, 1H), 2.83 - 2.66 (m, 1H), 2.36 – 2.33 (m, 3H), 2.10 (brs, 1H), 1.54 – 1.01 (m, 2H), 0.92 – 0.48 (m, 1H) ppm; **^{13}C NMR** (101 MHz, $CDCl_3$), δ = 170.9, 170.7, 170.5, 170.2, 170.0, 169.7, 169.3, 141.2, 140.4, 140.0, 139.0, 136.9, 135.8, 135.7, 135.4, 134.7, 134.3, 132.4, 131.5, 131.2, 130.8, 130.5, 129.6, 129.3, 128.7, 128.4, 128.2, 127.7, 127.6, 127.6, 127.5, 127.2, 127.1, 126.5, 126.3, 125.8, 61.6, 61.5, 52.6, 51.9, 51.7, 44.5, 42.8, 42.5, 39.8, 39.6, 38.8, 38.2, 35.8, 35.5, 20.5, 20.5, 20.5, 14.7, 14.2, 13.1, 12.1 ppm; **FTIR (neat) ν/cm^{-1}** 2980.83, 2360.36, 1745.64, 1382.22, 1208.83, 954.72;

HRMS (ESI$^+$) $C_{20}H_{24}NO_4$ $[M+H]^+$ requires 342.1700; found 342.1699.

Compound S17:

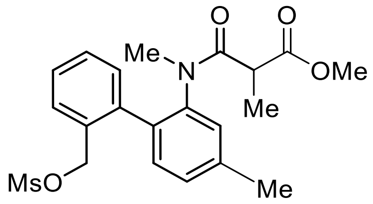

S17

Prepared according to General Procedure C with **S17-2** (450.0 mg, 1.31 mmol), methanesulfonic anhydride (276.0 mg, 1.59 mmol) and triethylamine (0.28 mL, 1.96 mmol). Purification by column chromatography, gradient eluting with ethyl acetate in petrol (25% to 50%), yielded product **S17** as an oil (501.0 mg, 91.2% yield).

^{1}H NMR (400 MHz, $CDCl_3$), δ = 7.58 – 7.42 (m, 1H), 7.36 – 7.25 (m, 2H), 7.18 - 6.96 (m, 4H), 5.23 – 4.72 (m, 2H), 3.72 – 3.43 (m, 3H), 3.39 – 3.01 (m, 2H), 2.97 – 2.54 (m, 5H), 2.41 – 2.24 (m, 3H), 1.51 – 1.10 (m, 2H), 0.81 – 0.66 (m, 1H) ppm; **^{13}C NMR** (101 MHz, $CDCl_3$), δ = 170.7, 170.3, 170.1, 169.9, 169.3, 169.1, 168.5, 141.3, 141.2, 140.8, 140.6, 139.5, 139.3, 139.0, 138.9, 138.7, 138.3, 137.7, 137.6, 134.4, 133.5, 133.1, 132.5, 131.8, 131.4, 131.2, 131.0, 130.8, 130.6, 130.5, 130.4, 130.2, 130.0, 129.9, 129.6, 129.5, 129.1, 129.1, 128.8, 128.7, 128.6, 128.5, 128.0, 127.9, 127.8, 127.6, 127.5, 70.0, 68.7, 68.5, 68.2, 51.8, 51.7, 51.7, 51.6, 51.4, 43.3, 43.1, 42.8, 42.5, 42.4, 39.1, 38.8, 37.1, 37.0, 36.1, 35.7, 35.4, 20.5, 20.4, 14.7, 14.2, 13.8, 13.2, 12.3 ppm;

FTIR (neat) ν/cm^{-1} 2980.78, 2360.67, 1698.50, 1380.87, 1174.08, 928.95;

HRMS (ESI^+) $C_{21}H_{26}NO_6S$ $[M+H]^+$ requires 420.1475; found 420.1477.

Compound S18-1:

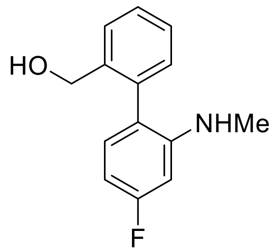

S18-1

Prepared according to General Procedure A with 5-fluoro-2-iodo-N-methylaniline (1.30 g, 5.20 mmol), benzo[c][1,2]oxaborol-1(3H)-ol (576.2 mg, 4.30 mmol), palladium acetate (97.0 mg, 0.43 mmol), triphenylphosphine (452.0 mg, 1.72 mmol), cesium carbonate (3.51 g, 10.75 mmol), and TBAB (139.0 mg, 0.43 mmol) in the mixed solvent of toluene and water (v/v = 1:1). Purification

by column chromatography, eluting with 10% ethyl acetate in petrol, yielded intermediate **S18-1** as an oil (481.0 mg, 48.4% yield). **^{1}H NMR** (400 MHz, $CDCl_3$), δ = 7.55 (dd, J = 7.3, 1.3 Hz, 1H), 7.47 – 7.33 (m, 2H), 7.18 (dd, J = 7.3, 1.5 Hz, 1H), 6.94 (dd, J = 8.2, 6.6 Hz, 1H), 6.50 (td, J = 8.4, 2.6 Hz, 1H), 6.43 (dd, J = 11.5, 2.4 Hz, 1H), 4.76 – 4.03 (m, 2H), 3.48 (brs, 1H), 2.72 (s, 3H) ppm; **^{13}C NMR** (101 MHz, $CDCl_3$), δ = 165.0, 162.6, 148.1 (d, J = 10.7 Hz), 140.1, 136.6, 130.6, 130.5 (d, J = 9.9 Hz), 129.0, 128.4 (d, J = 20.3 Hz), 122.4 (d, J = 2.8 Hz), 103.5 (d, J = 21.5 Hz), 97.8 (d, J = 26.2 Hz), 63.3, 30.5 ppm; **^{19}F NMR** (377 MHz, $CDCl_3$), δ = -112.85 (ddd, J = 11.5, 8.5, 6.5 Hz) ppm; **FTIR (neat) ν/cm^{-1}** 2980.64, 2360.46, 1614.25, 1381.35, 1172.60, 956.92;
HRMS (ESI$^+$) $C_{14}H_{15}FNO$ $[M+H]^+$ requires 232.1132; found 232.1134.

Compound S18-2:

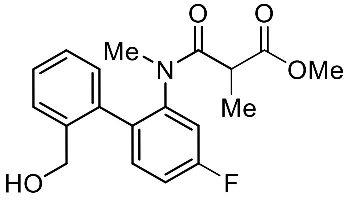

S18-2

Prepared according to General Procedure B with **S18-1** (440.0mg, 1.90 mmol). Purification by column chromatography, gradient eluting with ethyl acetate in petrol (25% to 50%), yielded intermediate **S18-2** as an oil (303.0 mg, 45.9% yield in 3 steps). **^{1}H NMR** (400 MHz, $CDCl_3$), δ = 7.58 – 7.47 (m, 1H), 7.44 – 7.28 (m, 2H), 7.25 -7.20 (m, J = 6.5 Hz, 1H), 7.18 – 6.83 (m, 3H), 4.44 - 4.24 (m, 2H), 3.89 – 3.46 (m, 4H), 3.40 – 3.03 (m, 2H), 2.67 (d, J = 40.3 Hz, 1H), 1.74 – 1.17 (m, 2H), 0.76 (t, J = 16.7 Hz, 1H) ppm; **^{13}C NMR** (101 MHz, $CDCl_3$), δ = 170.8, 170.4, 170.0, 169.4, 163.1, 160.6, 142.7, 142.7, 142.6, 142.1, 139.8, 138.7, 138.4, 136.0, 135.2, 135.1, 134.3, 133.9, 133.5, 132.9, 132.2, 131.5, 130.2, 129.3, 128.7, 128.4, 128.3, 128.1, 127.9, 127.8, 126.9, 126.2, 115.7, 115.5, 115.1, 114.9, 114.4, 114.3, 114.2, 114.1, 61.9, 61.7, 61.7, 52.2, 52.1, 52.0, 43.3, 42.9, 42.7, 39.6, 38.92, 35.9, 35.5, 14.8, 14.1, 13.2, 13.1, 12.3 ppm; **^{19}F NMR** (376 MHz, $CDCl_3$), δ = -61.41 – -144.62 (m) ppm; **FTIR (neat) ν/cm^{-1}** 2980.76, 2360.84, 1746.99, 1382.36, 1129.66, 948.12;
HRMS (ESI$^+$) $C_{19}H_{21}FNO_4$ $[M+H]^+$ requires 346.1449; found 346.1452.

Compound S18:

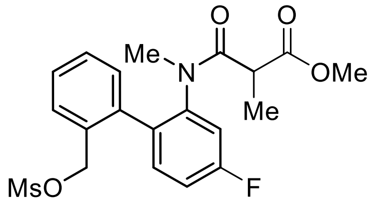

S18

Prepared according to General Procedure C with **S18-2** (283.0 mg, 0.82 mmol), methanesulfonic anhydride (172.0 mg, 0.99 mmol) and triethylamine (0.21 mL, 1.49 mmol). Purification by column chromatography, gradient eluting with ethyl acetate in petrol (25% to 50%), yielded product **S18** as an oil (206.0 mg, 59.3% yield). **^{1}H NMR** (400 MHz, $CDCl_3$), δ = 7.53 -7.41 (m, 1H), 7.44 – 7.20 (m, 3H), 7.18 – 7.10 (m, 1H), 7.04 (ddt, J = 11.1, 5.6, 2.8 Hz, 1H), 7.01 – 6.90 (m, 1H), 5.15 – 4.77 (m, 2H), 3.69 (s, 1H), 3.66 – 3.46 (m, 2H), 3.46 – 2.97 (m, 3H), 2.91 (d, J = 31.8 Hz, 2H), 2.83 – 2.60 (m, 2H), 1.54 – 1.12 (m, 2H), 0.79 (d, J = 6.8 Hz, 1H) ppm; **^{13}C NMR** (101 MHz, $CDCl_3$), δ = 170.6, 170.2, 170.1, 169.2, 168.5, 163.4, 163.3, 160.9, 160.8, 160.5, 143.1, 143.0, 142.9, 142.7, 142.5, 142.4, 137.6, 137.5, 137.0, 136.8, 133.7, 133.3, 133.2, 133.0, 132.8, 132.7, 132.1, 132.1, 131.1, 130.9, 130.7, 130.4, 129.8, 129.4, 129.1, 128.6, 128.5, 128.2, 128.1, 116.4, 116.2, 115.9, 115.6, 115.4, 115.3, 115.2, 114.5, 114.3, 114.1, 69.8, 68.6, 68.4, 68.1, 52.2, 52.1, 52.0, 43.5, 43.3, 43.0, 42.8, 42.7, 38.9, 37.3, 36.6, 35.9, 35.5, 14.8, 14.2, 13.3, 13.2, 12.6 ppm; **^{19}F NMR** (376 MHz, $CDCl_3$), δ = -84.92 – -129.41 (m) ppm; **FTIR (neat) ν/cm^{-1}** 2981.36, 2360.67, 1745.54, 1353.96, 1174.26, 926.52;

HRMS (ESI$^+$) $C_{20}H_{23}FNO_6S$ $[M+H]^+$ requires 424.1225; found 424.1226.

Compound S19-1:

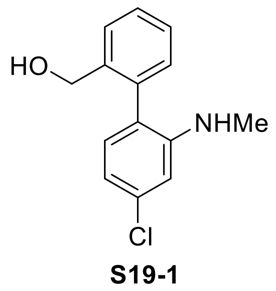

S19-1

Prepared according to General Procedure A with 5-chloro-2-iodo-*N*-methylaniline (1.20 g, 4.48 mmol), benzo[c][1,2]oxaborol-1(3H)-ol (500.0 mg, 3.73 mmol), palladium acetate (84.0 mg, 0.37 mmol), triphenylphosphine (393.0 mg, 1.50 mmol), cesium carbonate (3.04 g, 9.33 mmol), and

TBAB (121.0 mg, 0.37 mmol) in the mixed solvent of toluene and water (v/v = 1:1). Purification by column chromatography, eluting with 10% ethyl acetate in petrol, yielded intermediate **S19-1** as an oil (790.0 mg, 85.7% yield).

^{1}H NMR (400 MHz, $CDCl_3$), δ = 7.45 (dd, J = 7.4, 1.3 Hz, 1H), 7.30 (dtd, J = 16.7, 7.4, 1.5 Hz, 2H), 7.07 (dd, J = 7.3, 1.5 Hz, 1H), 6.81 (d, J = 7.9 Hz, 1H), 6.68 (dd, J = 7.9, 2.0 Hz, 1H), 6.60 (d, J = 2.0 Hz, 1H), 4.60 – 4.10 (m, 2H), 2.61 (s, 3H) ppm; **^{13}C NMR** (101 MHz, $CDCl_3$), δ = 147.4, 139.9, 136.3, 134.6, 130.4, 130.3, 129.0, 128.6, 128.3, 125.1, 117.2, 110.3, 63.2, 30.4 ppm; **FTIR (neat) ν/cm^{-1}** 2980.17, 22360.49, 1636.20, 1488.52, 1399.25, 1011.49;

HRMS (ESI$^+$) $C_{14}H_{15}ClNO$ $[M+H]^+$ requires 248.0837; found 248.0837.

Compound S19-2:

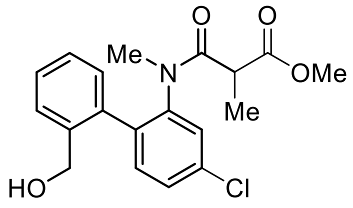

S19-2

Prepared according to General Procedure B with **S19-1** (782.0 mg, 3.16 mmol). Purification by column chromatography, gradient eluting with ethyl acetate in petrol (25% to 50%), yielded intermediate **S19-2** as an oil (578.0 mg, 44.3% yield in 3 steps).

^{1}H NMR (400 MHz, $CDCl_3$), δ = 7.51 (dd, J = 7.1, 3.8 Hz, 1H), 7.41 – 7.16 (m, 5H), 7.02 (dd, J = 73.4, 7.8 Hz, 1H), 5.01 – 4.01 (m, 2H), 3.99 – 3.50 (m, 2H), 3.50 – 3.03 (m, 3H), 2.70 (m, 1H), 1.89 (brs, 2H), 1.57 – 1.10 (m, 2H), 0.83-0.69 (m, 1H) ppm; **^{13}C NMR** (101 MHz, $CDCl_3$), δ = 170.7, 170.4, 169.9, 169.2, 142.5, 142.4, 141.7, 139.9, 138.5, 137.3, 136.2, 135.7, 135.0, 134.8, 133.7, 132.6, 131.9, 129.7, 129.3, 128.6, 128.3, 128.2, 128.0, 127.3, 127.2, 126.9, 126.1, 67.5, 61.6, 52.2, 52.1, 52.0, 43.4, 42.9, 42.6, 39.7, 38.9, 35.8, 25.2, 14.7, 13.8, 13.1, 12.2 ppm; **FTIR (neat) ν/cm^{-1}** 2980.79, 2360.38, 1652.97, 1457.47, 1240.3, 1024.57;

HRMS (ESI$^+$) $C_{19}H_{21}ClNO_4$ $[M+H]^+$ requires 362.1154; found 362.1157.

Compound S19:

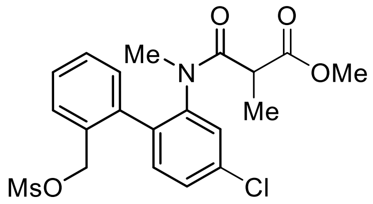

S19

Prepared according to General Procedure C with **S19-2** (570.0 mg, 1.58 mmol), methanesulfonic anhydride (331.0 mg, 1.90 mmol) and triethylamine (0.34 mL, 2.37 mmol). Purification by column chromatography, gradient eluting with ethyl acetate in petrol (25% to 50%), yielded product **S19** as an oil (603.0 mg, 86.9% yield).

^{1}H NMR (400 MHz, $CDCl_3$), δ = 7.53-7.49 (m, 1H), 7.49 – 7.20 (m, 5H), 7.19 – 6.81 (m, 1H), 5.57 – 4.61 (m, 2H), 3.84 – 3.60 (m, 2H), 3.59 – 3.47 (m, 1H), 3.45 – 3.28 (m, 1H), 3.21- 3.05 (m, 1H), 3.05 – 2.62 (m, 5H), 1.49 – 1.15 (m, 2H), 0.82-0.75 (m, 1H) ppm; **^{13}C NMR** (101 MHz, $CDCl_3$), δ = 170.5, 170.1, 169.9, 169.7, 169.0, 168.3, 167.9, 142.7, 142.6, 142.2, 141.9, 140.2, 137.2, 136.7, 136.5, 136.2, 135.4, 135.0, 134.9, 134.5, 134.3, 134.0, 133.8, 133.2, 132.8, 132.6, 132.4, 131.8, 130.9, 130.6, 130.5, 130.4, 130.3, 130.1, 129.6, 129.4, 129.3, 129.0, 128.7, 128.5, 128.2, 128.1, 127.6, 127.3, 127.2, 69.7, 68.5, 68.3, 68.0, 52.1, 52.0, 52.0, 45.8, 43.4, 43.4, 43.1, 42.6, 41.5, 38.9, 37.2, 36.5, 35.8, 35.4, 14.7, 14.5, 14.2, 13.7, 13.2 ppm; **FTIR (neat) ν/cm^{-1}** 2980.97, 2360.46, 1743.36, 13543.06, 1174.11, 924.74;

HRMS (ESI$^+$) $C_{20}H_{23}ClNO_6S$ $[M+H]^+$ requires 440.0929; found 440.0931.

Compound S20-1:

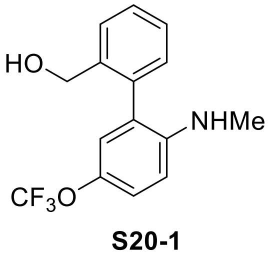

S20-1

Prepared according to General Procedure A with 2-bromo-N-methyl-4-(trifluoromethoxy)aniline (1.20 g, 4.48 mmol), benzo[c][1,2]oxaborol-1(3H)-ol (500.0 mg, 3.73 mmol), palladium acetate (84.0 mg, 0.37 mmol), triphenylphosphine (392.0 mg, 1.49 mmol), cesium carbonate (3.04 g, 9.32 mmol), and TBAB (120.0 mg, 0.37 mmol) in the mixed solvent of toluene and water (v/v = 1:1).

Purification by column chromatography, eluting with 10% ethyl acetate in petrol, yielded intermediate **S20-1** as an oil (709.0 mg, 64.0% yield). **^{1}H NMR** (400 MHz, $CDCl_3$), δ = 7.58 (dd, J = 7.5, 1.3 Hz, 1H), 7.43 (dtd, J = 18.8, 7.5, 1.5 Hz, 2H), 7.22 – 7.15 (m, 2H), 6.93 (d, J = 2.1 Hz, 1H), 6.70 (d, J = 8.8 Hz, 1H), 4.91 – 3.69 (m, 2H), 3.17 (s, 1H), 2.74 (s, 3H); **^{13}C NMR** (101 MHz, $CDCl_3$), δ = 145.1, 140.6, 140.6, 139.8, 136.1, 130.1, 129.4, 128.9, 128.5, 127.9, 124.2, 122.8, 121.9, 121.7, 119.8, 116.8, 110.8, 63.5, 30.8 ppm; **^{19}F NMR** (377 MHz, $CDCl_3$), δ = -58.40 ppm; **FTIR (neat) ν/cm^{-1}** 2981.03, 2360.46, 1614.36, 1255.06, 1159.39, 764.17;
HRMS (ESI$^+$) $C_{15}H_{15}F_3NO_2$ $[M+H]^+$ requires 298.1049; found 298.1049.

Compound S20-2:

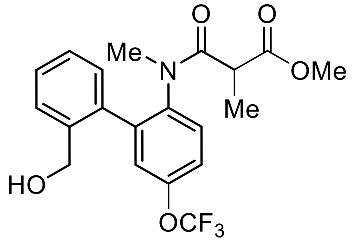

S20-2

Prepared according to General Procedure B with **S20-1** (700.0 mg, 2.35 mmol). Purification by column chromatography, gradient eluting with ethyl acetate in petrol (25% to 50%), yielded intermediate **S20-2** as an oil (272.0 mg, 28.1% yield in 3 steps). **^{1}H NMR** (400 MHz, $CDCl_3$), δ = 7.52 (d, J = 7.7 Hz, 1H), 7.46 – 7.27 (m, 2H), 7.26 – 7.09 (m, 3H), 7.07 – 6.88 (m, 1H), 4.50 – 4.15 (m, 2H), 3.78 – 3.54 (m, 2H), 3.53 – 2.98 (m, 4H), 2.83 – 2.50 (m, 1H), 1.45 – 1.09 (m, 2H), 0.90 – 0.64 (m, 1H) ppm; **^{13}C NMR** (101 MHz, $CDCl_3$), δ = 170.8, 170.6, 170.1, 169.6, 147.8, 147.3, 147.3, 140.8, 140.2, 140.1, 139.5, 138.4, 135.6, 134.9, 134.7, 130.1, 129.4, 128.7, 128.6, 128.5, 128.4, 127.9, 127.1, 126.3, 124.4, 124.0, 123.8, 123.4, 121.4, 121.4, 121.1, 120.9, 118.9, 118.8, 116.3, 116.3, 62.0, 61.8, 61.7, 52.3, 52.2, 52.0, 43.5, 43.4, 43.1, 42.8, 39.8, 39.1, 36.1, 35.7, 14.9, 14.2, 13.2, 12.3 ppm; **^{19}F NMR** (377 MHz, $CDCl_3$), δ = -57.86, -57.88, -57.90, -57.94, -57.94, -57.96 ppm; **FTIR (neat) ν/cm^{-1}** 2980.74, 2360.56, 1652.77, 1218.72, 1167.66, 954.76;
HRMS (ESI$^+$) $C_{20}H_{21}F_3NO_5$ $[M+H]^+$ requires 412.1366; found 412.1367.

Compound S20:

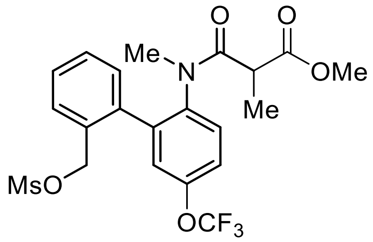

S20

Prepared according to General Procedure C with **S20-2** (300.0 mg, 0.73 mmol), methanesulfonic anhydride (154.0 mg, 0.88 mmol) and triethylamine (0.16 mL, 1.09 mmol). Purification by column chromatography, gradient eluting with ethyl acetate in petrol (25% to 50%), yielded product **S20** as an oil (282.0 mg, 78.9% yield). **^{1}H NMR** (400 MHz, $CDCl_3$), δ = 7.60 – 7.43 (m, 1H), 7.41 – 7.31 (m, 2H), 7.29 – 7.14 (m, 3H), 7.14 – 6.93 (m, 1H), 5.21 – 4.78 (m, 2H), 3.65 (d, J = 7.7 Hz, 1H), 3.60 – 3.23 (m, 3H), 3.22 – 3.08 (m, 1H), 3.06 – 2.55 (m, 5H), 1.55 – 1.02 (m, 2H), 0.89 – 0.65 (m, 1H); **^{13}C NMR** (101 MHz, $CDCl_3$), δ = 170.7, 170.5, 170.3, 170.2, 169.9, 169.5, 168.7, 148.0, 147.9, 147.8, 147.4, 147.3, 141.0, 140.3, 139.9, 139.7, 139.0, 138.6, 138.5, 137.6, 136.6, 136.6, 136.5, 132.6, 131.3, 131.1, 130.9, 130.7, 130.7, 130.6, 130.5, 130.2, 129.9, 129.7, 129.6, 129.3, 129.1, 129.0, 128.7, 128.5, 128.4, 128.2, 124.4, 124.0, 124.0, 123.8, 123.6, 123.3, 121.6, 121.4, 121.4, 121.3, 121.1, 118.8, 118.8, 116.3, 70.2, 68.3, 68.1, 67.8, 52.3, 52.2, 52.1, 43.6, 43.3, 43.2, 42.9, 42.7, 40.2, 37.4, 37.3, 36.6, 36.2, 35.7, 14.9, 14.3, 13.4, 12.6 ppm; **^{19}F NMR** (377 MHz, $CDCl_3$), δ = -57.74, -57.75, -57.82, -57.85, -57.92 ppm; **FTIR (neat) ν/cm^{-1}** 2981.00, 2360.68, 1656.09, 1252.46, 1173.70, 928.38;

HRMS (ESI^{+}) $C_{21}H_{23}F_3NO_7S$ $[M+H]^{+}$ requires 490.1142; found 490.1141.

Compound S21-1:

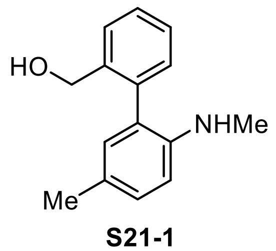

S21-1

Prepared according to General Procedure A with 4-methyl-2-iodo-*N*-methylaniline (995.0 mg, 4.02 mmol), benzo[c][1,2]oxaborol-1(3H)-ol (450.0 mg, 3.36 mmol), palladium acetate (75.4 mg, 0.33 mmol), triphenylphosphine (352.0 mg, 1.34 mmol), cesium carbonate (2.74 g, 8.40 mmol),

and TBAB (108.0 mg, 0.33 mmol) in the mixed solvent of toluene and water (v/v = 1:1). Purification by column chromatography, eluting with 10% ethyl acetate in petrol, yielded intermediate **S21-1** as an oil (680.0 mg, 89.4% yield).

^{1}H NMR (400 MHz, $CDCl_3$), δ = 7.45 (dd, J = 7.2, 1.7 Hz, 1H), 7.39 – 7.25 (m, 2H), 7.12 (dd, J = 7.1, 1.8 Hz, 1H), 7.05 (dd, J = 8.2, 1.6 Hz, 1H), 6.78 (d, J = 1.9 Hz, 1H), 6.62 (d, J = 8.2 Hz, 1H), 4.27 (dd, J = 12.6 Hz, 2H), 2.62 (s, 3H), 2.21 (s, 3H) ppm; **^{13}C NMR** (101 MHz, $CDCl_3$), δ = 143.8, 140.1, 137.9, 130.3, 130.2, 129.6, 129.3, 128.4, 128.3, 128.1, 127.6, 111.2, 64.0, 31.0, 20.3 ppm; **FTIR (neat) ν/cm^{-1}** 2980.69, 2360.45, 1648.91, 1473.41, 1224.18, 880.05;
HRMS (ESI$^+$) $C_{15}H_{18}NO$ [M+H]$^+$ requires 228.1383; found 228.1384.

Compound S21-2:

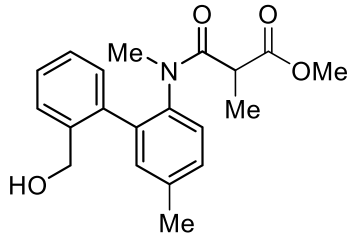

S21-2

Prepared according to General Procedure B with **S21-1** (620.0 mg, 2.73 mmol). Purification by column chromatography, gradient eluting with ethyl acetate in petrol (25% to 50%), yielded intermediate **S21-2** as an oil (560 mg, 60.1% yield in 3 steps).

^{1}H NMR (400 MHz, $CDCl_3$), δ = 7.62 – 7.41 (m, 1H), 7.40 – 7.27 (m, 1H), 7.26 – 7.10 (m, 3H), 7.11 – 6.86 (m, 2H), 5.46 – 4.60 (m, 1H), 4.57 – 4.13 (m, 2H), 4.00 – 3.45 (m, 2H), 3.46 – 3.06 (m, 2H), 2.78 (s, 1H), 2.68 (s, 1H), 2.47 – 2.30 (m, 3H), 1.64 – 0.69 (m, 3H) ppm; **^{13}C NMR** (101 MHz, $CDCl_3$), δ = 174.8, 173.6, 171.1, 170.9, 170.6, 170.4, 170.2, 169.7, 140.1, 139.3, 139.0, 139.0, 138.9, 138.5, 138.4, 138.1, 138.0, 137.9, 137.8, 137.4, 136.8, 136.2, 136.1, 135.5, 132.6, 132.5, 132.0, 131.4, 131.0, 130.8, 130.0, 129.8, 129.7, 129.5, 129.3, 129.2, 128.5, 128.3, 128.0, 127.9, 127.8, 127.5, 127.4, 127.2, 127.0, 126.7, 126.5, 126.0, 64.1, 62.0, 61.9, 60.9, 60.1, 52.1, 51.9, 43.4, 43.2, 42.7, 40.1, 39.1, 37.3, 36.1, 35.8, 35.6, 35.4, 32.9, 20.7, 20.7, 14.9, 14.2, 13.9, 13.5, 13.3, 12.3 ppm; **FTIR (neat) ν/cm^{-1}** 2980.66, 2360.52, 1698.42, 1541.31, 1386.97, 1071.23;
HRMS (ESI$^+$) $C_{20}H_{24}NO_4$ [M+H]$^+$ requires 342.1700; found 342.1700.

Compound S21:

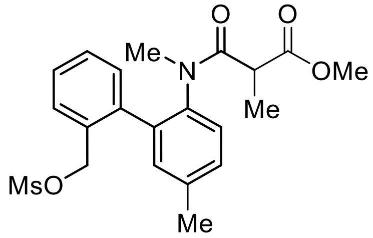

S21

Prepared according to General Procedure C with **S21-2** (555.0 mg, 1.63 mmol), methanesulfonic anhydride (340.0 mg, 1.95 mmol) and triethylamine (0.35 mL, 2.44 mmol). Purification by column chromatography, gradient eluting with ethyl acetate in petrol (25% to 50%), yielded product **S21** as an oil (465.0 mg, 68.0% yield).

^{1}H NMR (400 MHz, $CDCl_3$), δ = 7.68 – 7.47 (m, 1H), 7.48 – 7.27 (m, 2H), 7.25 – 6.84 (m, 4H), 5.45 – 4.81 (m, 2H), 3.77 – 3.45 (m, 3H), 3.42 – 3.04 (m, 2H), 3.01 – 2.91 (m, 1H), 2.91 – 2.74 (m, 3H), 2.71 – 2.63 (m, 1H), 2.47 – 2.24 (m, 3H), 1.56 – 1.10 (m, 2H), 0.79 (d, J = 6.9 Hz, 1H) ppm; **^{13}C NMR** (101 MHz, $CDCl_3$), δ = 170.9, 170.7, 170.4, 170.4, 170.1, 169.5, 168.9, 162.7, 139.0, 138.7, 138.5, 138.4, 138.4, 138.2, 138.0, 138.0, 137.9, 137.5, 137.0, 136.9, 136.4, 136.1, 136.0, 132.8, 132.3, 132.1, 131.9, 131.9, 131.4, 130.8, 130.5, 130.4, 130.2, 130.2, 130.1, 129.9, 129.7, 129.6, 129.6, 129.1, 128.9, 128.4, 128.3, 128.2, 128.2, 127.9, 127.8, 127.1, 126.9, 70.1, 68.7, 68.5, 68.3, 68.2, 52.1, 52.0, 52.0, 51.9, 51.6, 43.4, 43.2, 42.8, 39.1, 39.0, 37.4, 37.3, 37.2, 37.2, 36.1, 35.7, 32.8, 20.7, 20.6, 20.6, 14.9, 14.3, 14.0, 13.8, 13.4, 12.6 ppm; **FTIR (neat) ν/cm^{-1}** 2942.72, 2361.04, 1655.00, 1353.42, 1173.99, 927.11;

HRMS (ESI$^+$) $C_{21}H_{26}NO_6S$ $[M+H]^+$ requires 420.1475; found 420.1475.

Compound S22-1:

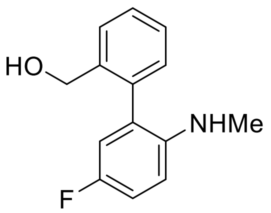

S22-1

Prepared according to General Procedure A with 4-fluoro-2-iodo-*N*-methylaniline (998.5 mg, 3.98 mmol), benzo[c][1,2]oxaborol-1(3H)-ol (445.0 mg, 3.32 mmol), palladium acetate (75.0 mg, 0.33 mmol), triphenylphosphine (349.0 mg, 1.33 mmol), cesium carbonate (2.90 g, 8.80 mmol), and

TBAB (108.0 mg, 0.33 mmol) in the mixed solvent of toluene and water (v/v = 1:1). Purification by column chromatography, eluting with 10% ethyl acetate in petrol, yielded intermediate **S22-1** as an oil (600.0 mg, 78.2% yield).

^{1}H NMR (400 MHz, $CDCl_3$), δ = 7.47 (dd, J = 7.4, 1.5 Hz, 1H), 7.33 (dtd, J = 16.4, 7.4, 1.6 Hz, 2H), 7.10 (dd, J = 7.3, 1.5 Hz, 1H), 6.99 – 6.82 (m, 1H), 6.71 (dd, J = 8.7, 3.0 Hz, 1H), 6.64 (dd, J = 8.9, 4.7 Hz, 1H), 4.33 – 4.21 (m, 2H), 3.39 (s, 1H), 2.62 (s, 2H) ppm; **^{13}C NMR** (101 MHz, $CDCl_3$), δ = 157.3, 154.9, 142.3, 139.8, 136.5, 129.9, 129.7, 129.1, 128.8, 128.4, 116.7, 116.5, 115.0, 114.8, 112.0, 112.0, 63.6, 31.3 ppm; **^{19}F NMR** (377 MHz, $CDCl_3$), δ = -127.16 (td, J = 8.8, 4.7 Hz) ppm; **FTIR (neat) ν/cm^{-1}** 2980.63, 2360.43, 1509.62, 1408.34, 1175.77, 808.28;

HRMS (ESI$^+$) $C_{14}H_{15}FNO$ $[M+H]^+$ requires 232.1132; found 232.1131.

Compound S22-2:

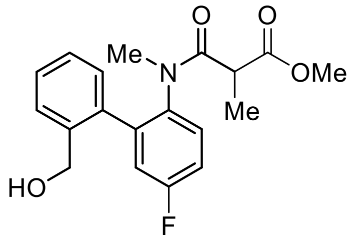

S22-2

Prepared according to General Procedure B with **S22-1** (600.0 mg, 2.59 mmol). Purification by column chromatography, gradient eluting with ethyl acetate in petrol (25% to 50%), yielded intermediate **S22-2** as an oil (169.0 mg, 18.9% yield in 3 steps).

^{1}H NMR (400 MHz, $CDCl_3$), δ = 7.60 – 7.44 (m, 1H), 7.38 - 7.33 (m, 1H), 7.29 – 7.17 (m, 2H), 7.13 – 7.03 (m, 2H), 6.969 - 6.85 (m, 1H), 4.76 – 4.06 (m, 2H), 3.64 (d, J = 17.3 Hz, 2H), 3.53 – 3.38 (m, 1H), 3.38 – 2.93 (m, 3H), 2.84 – 2.47 (m, 1H), 1.98 (brs, 1H), 1.69 – 1.13 (m, 2H), 0.95 – 0.63 (m, 2H) ppm; **^{13}C NMR** (101 MHz, $CDCl_3$), δ = 170.8, 170.7, 170.1, 169.7, 162.3, 162.0, 161.9, 159.8, 159.5, 159.4, 140.1, 137.7, 136.6, 136.0, 135.3, 130.1, 129.8, 129.4, 128.6, 128.5, 128.3, 128.1, 127.9, 127.2, 126.2, 118.6, 118.4, 118.2, 117.9, 117.7, 115.8, 115.6, 62.1, 61.9, 61.8, 52.4, 52.2, 52.1, 43.5, 43.3, 43.2, 42.8, 40.1, 39.8, 39.3, 36.3, 35.9, 15.0, 14.3, 13.4, 12.4 ppm; **^{19}F NMR** (377 MHz, $CDCl_3$), δ = -112.43, -114.01 ppm; **FTIR (neat) ν/cm^{-1}** 2980.69, 2360.59, 1698.28, 1435.98, 1200.34, 955.29;

HRMS (ESI$^+$) $C_{19}H_{21}FNO_4$ $[M+H]^+$ requires 346.1449; found 346.1451.

Compound S22:

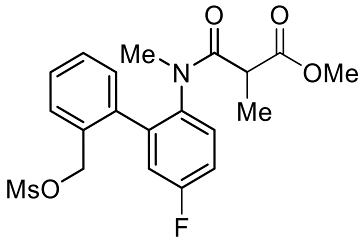

S22

Prepared according to General Procedure C with **S22-2** (165.0 mg, 0.48 mmol), methanesulfonic anhydride (102.0 mg, 0.58 mmol) and triethylamine (0.11 mL, 0.73 mmol). Purification by column chromatography, gradient eluting with ethyl acetate in petrol (25% to 50%), yielded product **S22** as an oil (180.0 mg, 88.6% yield).

^{1}H NMR (400 MHz, $CDCl_3$), δ = 7.62 – 7.46 (m, 1H), 7.44 – 7.26 (m, 2H), 7.27 – 7.02 (m, 3H), 7.02 – 6.80 (m, 1H), 5.22 – 4.77 (m, 2H), 3.80 – 3.56 (m, 2H), 3.55 – 3.26 (m, 2H), 3.24 – 3.00 (m, 2H), 2.93 – 2.55 (m, 4H), 1.49 – 1.08 (m, 2H), 0.84 – 0.64 (m, 1H) ppm; **^{13}C NMR** (101 MHz, $CDCl_3$), δ = 170.8, 170.7, 170.4, 170.3, 169.6, 168.9, 162.5, 162.0, 162.0, 159.6, 159.5, 138.7, 137.9, 137.4, 136.9, 132.7, 131.2, 130.8, 130.7, 130.6, 130.2, 129.6, 129.3, 129.0, 128.9, 128.8, 128.4, 128.1, 128.0, 125.1, 118.9, 118.5, 118.3, 117.9, 117.7, 116.4, 116.1, 115.9, 69.9, 68.4, 68.1, 68.0, 52.3, 52.2, 45.9, 43.6, 43.3, 43.1, 43.0, 42.8, 39.4, 37.6, 36.7, 36.3, 35.9, 15.0, 14.3, 13.5, 12.7 ppm; **^{19}F NMR** (377 MHz, $CDCl_3$), δ = -111.09 – -112.40 (m), -113.14 – -114.21 (m) ppm;
FTIR (neat) ν/cm^{-1} 2981.03, 2360.55, 1655.34, 13543.03, 1174.06, 967.62;
HRMS (ESI$^+$) $C_{20}H_{23}FNO_6S$ $[M+H]^+$ requires 424.1225; found 424.1228.

Compound S23-1:

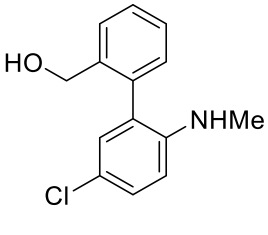

S23-1

Prepared according to General Procedure A with 4-chloro-2-iodo-*N*-methylaniline (1.20 g, 4.48 mmol), benzo[c][1,2]oxaborol-1(3H)-ol (500.0 mg, 3.73 mmol), palladium acetate (84.0 mg, 0.37 mmol), triphenylphosphine (393.0 mg, 1.50 mmol), cesium carbonate (3.04 g, 9.33 mmol), and

TBAB (121.0 mg, 0.37 mmol) in the mixed solvent of toluene and water (v/v = 1:1). Purification by column chromatography, eluting with 10% ethyl acetate in petrol, yielded intermediate **S23-1** as an oil (790.0 mg, 71.3% yield).

^{1}H NMR (400 MHz, $CDCl_3$), δ = 7.57 (dd, J = 7.4, 1.3 Hz, 1H), 7.42 (dtd, J = 16.3, 7.4, 1.6 Hz, 2H), 7.28 (dd, J = 8.7, 2.5 Hz, 1H), 7.19 (dd, J = 7.3, 1.5 Hz, 1H), 7.02 (d, J = 2.5 Hz, 1H), 6.68 (d, J = 8.7 Hz, 1H), 5.21 – 4.02 (m, 2H), 3.32 (s, 2H), 2.72 (s, 3H) ppm; **^{13}C NMR** (101 MHz, $CDCl_3$), δ = 145.1, 139.9, 136.2, 130.1, 129.4, 129.2, 128.8, 128.6, 128.6, 128.4, 122.4, 111.6, 63.2, 30.8 ppm; **FTIR (neat) ν/cm^{-1}** 2980.79, 2360.64, 1508.32, 1397.00, 1165.99, 952.85; **HRMS** (ESI^+) $C_{14}H_{15}ClNO$ $[M+H]^+$ requires 248.0837; found 248.0838.

Compound S23-2:

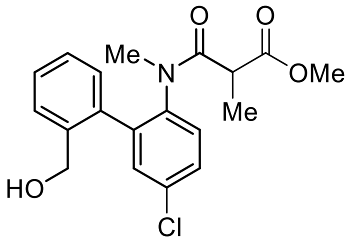

S23-2

Prepared according to General Procedure B with **S23-1** (790.0 mg, 3.20 mmol). Purification by column chromatography, gradient eluting with ethyl acetate in petrol (25% to 50%), yielded intermediate **S23-2** as an oil (546.0 mg, 47.2% yield in 3 steps).

^{1}H NMR (400 MHz, $CDCl_3$), δ = ^{1}H NMR (400 MHz, $CDCl_3$) 7.62 – 7.39 (m, 1H), 7.39 – 7.29 (m, 2H), 7.28 – 7.09 (m, 3H), 7.08 – 6.78 (m, 1H), 4.57 – 4.08 (m, 2H), 3.76 – 3.08 (m, 6H), 2.84 – 2.50 (m, 1H), 1.53 – 1.10 (m, 2H), 0.91 – 0.59 (m, 1H).ppm; **^{13}C NMR** (126 MHz, $CDCl_3$), δ = 171.0, 170.6, 170.2, 170.0, 169.7, 169.2, 141.0, 140.8, 140.5, 140.4, 140.0, 139.6, 138.1, 137.8, 136.1, 136.0, 135.4, 133.8, 133.0, 132.9, 132.1, 131.9, 131.6, 131.4, 131.2, 130.6, 130.4, 130.2, 130.1, 129.9, 129.8, 129.4, 129.2, 129.1, 129.0, 128.9, 128.7, 128.6, 128.5, 128.3, 128.1, 127.7, 126.3, 64.9, 64.7, 62.7, 62.6, 62.1, 52.6, 52.5, 45.9, 45.9, 43.8, 43.6, 43.2, 43.1, 40.0, 39.8, 39.3, 36.4, 36.0, 30.9, 15.2, 14.8, 14.5, 13.8, 13.6, 13.6, 13.2, 12.7 ppm; **FTIR (neat) ν/cm^{-1}** 2980.73, 2360.85, 1698.51, 1436.01, 1249.26, 1021.43;

HRMS (ESI^+) $C_{19}H_{21}ClNO_4$ $[M+H]^+$ requires 362.1154; found 362.1155.

Compound S23:

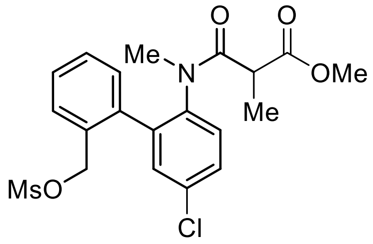

S23

Prepared according to General Procedure C with **S23-2** (540.0 mg, 1.50 mmol), methanesulfonic anhydride (314.0 mg, 1.80 mmol) and triethylamine (0.32 mL, 2.25 mmol). Purification by column chromatography, gradient eluting with ethyl acetate in petrol (25% to 50%), yielded product **S23** as an oil (561.0 mg, 85.2% yield).

^{1}H NMR (400 MHz, $CDCl_3$), δ = 7.58 – 7.43 (m, 1H), 7.41 – 7.27 (m, 4H), 7.26 – 6.86 (m, 2H), 5.90 – 4.43 (m, 2H), 3.64 (d, J = 7.4 Hz, 1H), 3.58 – 3.26 (m, 3H), 3.20 – 2.89 (m, 2H), 2.91 – 2.49 (m, 4H), 1.54 – 0.98 (m, 2H), 0.75 - 0.73 (m, 1H) ppm; **^{13}C NMR** (101 MHz, $CDCl_3$), δ = 170.8, 170.6, 170.4, 170.4, 169.6, 168.9, 140.8, 140.7, 140.3, 140.0, 139.9, 139.7, 138.8, 138.5, 138.4, 137.5, 137.0, 136.8, 133.9, 133.8, 133.6, 132.7, 132.6, 131.9, 131.5, 131.4, 131.1, 131.0, 130.9, 130.9, 130.8, 130.5, 130.4, 129.9, 129.9, 129.8, 129.7, 129.4, 129.3, 129.2, 129.0, 128.6, 70.1, 68.9, 68.6, 68.4, 52.4, 52.4, 52.3, 51.9, 43.7, 43.5, 43.2, 43.0, 39.3, 37.6, 37.5, 37.4, 36.8, 36.3, 35.8, 15.1, 14.5, 13.6, 12.8 ppm; **FTIR (neat) ν/cm^{-1}** 2980.90, 2360.70, 1655.73, 1354.62, 1174.15, 967.18;

HRMS (ESI$^+$) $C_{20}H_{23}ClNO_6S$ $[M+H]^+$ requires 440.0929; found 440.0929.

Compound S24-1:

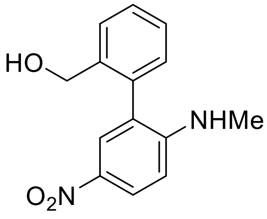

S24-1

Prepared according to General Procedure A with 4-nitro-2-iodo-*N*-methylaniline (1.25 g, 4.48 mmol), benzo[c][1,2]oxaborol-1(3H)-ol (500.0 mg, 3.73 mmol), palladium acetate (83.7 mg, 0.37 mmol), triphenylphosphine (393.0 mg, 1.50 mmol), cesium carbonate (3.19 g, 9.32 mmol), and TBAB (120.2 mg, 0.37 mmol) in the mixed solvent of toluene and water (v/v = 1:1). Purification

by column chromatography, eluting with 10% ethyl acetate in petrol, yielded intermediate **S24-1** as an oil (650.0 mg, 67.2% yield).

^{1}H NMR (400 MHz, $CDCl_3$), δ = 8.09 (dd, J = 9.1, 2.7 Hz, 1H), 7.81 (d, J = 2.7 Hz, 1H), 7.51 (d, J = 7.5 Hz, 1H), 7.42 – 7.24 (m, 2H), 7.09 (dd, J = 7.3, 1.3 Hz, 1H), 6.54 (d, J = 9.1 Hz, 1H), 4.39 – 4.26 (m, 2H), 2.77 (s, 3H) ppm; **^{13}C NMR** (101 MHz, $CDCl_3$), δ = 151.8, 139.6, 137.3, 134.4, 130.3, 129.1, 128.6, 128.4, 126.1, 125.9, 124.9, 108.0, 62.4, 30.1 ppm; **FTIR (neat) ν/cm^{-1}** 2980.67, 2360.62, 1579.03, 1325.33, 1151.42, 954.15;

HRMS (ESI^{+}) $C_{14}H_{15}N_2O_3$ $[M+H]^{+}$ requires 259.1077; found 259.1077.

Compound S24-2:

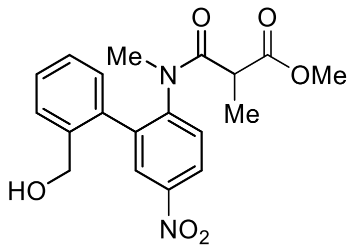

S24-2

Prepared according to General Procedure B with **S24-1** (650.0 mg, 2.51 mmol). Purification by column chromatography, gradient eluting with ethyl acetate in petrol (25% to 50%), yielded intermediate **S24-2** as an oil (379.0 mg, 40.5% yield in 3 steps).

^{1}H NMR (400 MHz, $CDCl_3$), δ = 8.36 – 8.00 (m, 2H), 7.55 (d, J = 7.8 Hz, 1H), 7.49 – 7.34 (m, 2H), 7.33 – 7.21 (m, 1H), 7.19 - 6.96 (m, 1H), 4.74 – 4.28 (m, 2H), 3.85 – 3.49 (m, 3H), 3.47 – 3.08 (m, 4H), 2.92 – 2.53 (m, 1H), 2.15 – 1.72 (m, 2H), 1.42 – 1.13 (m, 2H), 0.78 (s, 1H) ppm; **^{13}C NMR** (101 MHz, $CDCl_3$), δ = 170.4, 169.8, 169.0, 147.5, 147.4, 146.8, 146.6, 146.3, 146.0, 145.9, 139.9, 139.4, 138.3, 134.9, 134.9, 134.3, 129.7, 129.1, 129.0, 128.7, 128.6, 127.5, 126.7, 126.3, 126.1, 125.8, 125.4, 123.6, 123.5, 121.8, 62.1, 61.9, 61.8, 60.1, 53.0, 52.4, 52.3, 43.63, .0, 42.8, 38.9, 36.0, 14.8, 14.2, 13.2, 12.6 ppm; **FTIR (neat) ν/cm^{-1}** 2980.64, 2360.57, 1698.50, 1497.61, 1250.68, 954.89;

HRMS (ESI^{+}) $C_{19}H_{21}N_2O_6$ $[M+H]^{+}$ requires 373.1394; found 373.1395.

Compound S24:

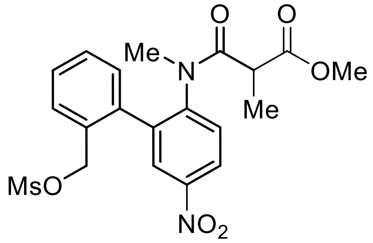

S24

Prepared according to General Procedure C with **S24-2** (260 mg, 0.70 mmol), methanesulfonic anhydride (147.0 mg, 0.84 mmol) and triethylamine (0.15 mL, 1.05 mmol). Purification by column chromatography, gradient eluting with ethyl acetate in petrol (25% to 50%), yielded product **S24** as an oil (271 mg, 86.0% yield).

^{1}H NMR (400 MHz, $CDCl_3$), δ = 8.38 – 7.90 (m, 2H), 7.73 – 7.28 (m, 4H), 7.17 – 6.94 (m, 1H), 5.66 – 4.63 (m, 2H), 4.06 – 3.33 (m, 4H), 3.27 – 2.94 (m, 2H), 2.92 – 2.49 (m, 4H), 1.54 – 1.13 (m, 2H), 0.82 – 0.72 (m, 1H) ppm; **^{13}C NMR** (101 MHz, $CDCl_3$), δ = 170.3, 170.2, 169.9, 169.5, 169.2, 147.7, 147.6, 147.0, 146.8, 146.6, 146.4, 146.0, 145.9, 137.9, 137.3, 136.6, 136.3, 136.1, 134.4, 131.8, 131.2, 130.8, 130.5, 130.1, 129.6, 129.4, 129.3, 129.1, 128.9, 127.2, 126.8, 126.3, 126.1, 124.3, 124.1, 123.9, 69.0, 67.9, 67.7, 67.2, 52.4, 52.3, 52.2, 43.6, 43.3, 42.9, 40.6, 40.4, 39.1, 37.5, 37.0, 36.0, 35.5, 33.5, 28.1, 23.5, 22.1, 20.5, 17.2, 16.9, 14.8, 14.3, 14.3, 13.2, 12.8, 10.8 ppm; **FTIR (neat) ν/cm^{-1}** 2980.74, 2360.59, 1747.16, 1351.74, 1174.06, 928.12;
HRMS (ESI$^+$) $C_{20}H_{23}N_2O_8S$ $[M+H]^+$ requires 451.1170; found 451.1168.

Compound S25-1:

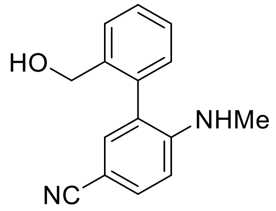

S25-1

Prepared according to General Procedure A with 3-iodo-4-(methylamino)benzonitrile (1.16 g, 4.48 mmol), benzo[c][1,2]oxaborol-1(3H)-ol (500.0 mg, 3.73 mmol), palladium acetate (84.0 mg, 0.37 mmol), triphenylphosphine (391.0 mg, 1.50 mmol), cesium carbonate (3.10 g, 9.33 mmol), and TBAB (120.0 mg, 0.37 mmol) in the mixed solvent of toluene and water (v/v = 1:1). Purification

by column chromatography, eluting with 10% ethyl acetate in petrol, yielded intermediate **S25-1** as an oil (870.0 mg, 98.0% yield).

^{1}H NMR (400 MHz, $CDCl_3$), δ = 7.63 – 7.57 (m, 1H), 7.54 (dd, J = 8.5, 2.0 Hz, 1H), 7.45 (td, J = 7.5, 1.5 Hz, 1H), 7.39 (td, J = 7.5, 1.4 Hz, 1H), 7.22 (d, J = 2.0 Hz, 1H), 7.15 (dd, J = 7.5, 1.3 Hz, 1H), 6.66 (d, J = 8.6 Hz, 2H), 4.38 (s, 1H), 2.79 (s, 3H) ppm; **^{13}C NMR** (101 MHz, $CDCl_3$), δ = 149.7, 139.6, 134.7, 133.6, 133.1, 130.2, 129.0, 128.7, 128.4, 126.1, 120.2, 109.4, 98.5, 62.5, 30.0 ppm; **FTIR (neat) ν/cm^{-1}** 2980.70, 2360.43, 1525.73, 1385.91, 1166.97, 953.54;

HRMS (ESI$^+$) $C_{15}H_{15}N_2O$ $[M+H]^+$ requires 239.1179; found 239.1180.

Compound S25-2:

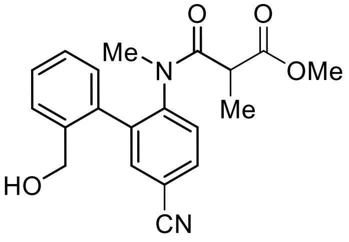

S25-2

Prepared according to General Procedure B with **S25-1** (785.0 mg, 3.30 mmol). Purification by column chromatography, gradient eluting with ethyl acetate in petrol (25% to 50%), yielded intermediate **S25-2** as an oil (327.0 mg, 28.1% yield in 3 steps). **^{1}H NMR** (400 MHz, $CDCl_3$), δ = 7.82 – 7.61 (m, 1H), 7.58 – 7.46 (m, 21H), 7.41 – 7.20 (m, 3H), 7.18 – 6.85 (m, 1H), 4.52 – 4.13 (m, 2H), 3.76 – 3.57 (m, 2H), 3.55 – 3.34 (m, 2H), 3.33 – 2.97 (m, 2H), 2.85 – 2.60 (m, 1H), 2.19 (s, 1H), 1.48 – 1.15 (m, 2H), 0.98 – 0.56 (m, 1H) ppm; **^{13}C NMR** (101 MHz, $CDCl_3$), δ = 170.9, 170.3, 170.1, 169.8, 169.1, 145.7, 145.7, 145.0, 139.2, 138.3, 135.8, 135.3, 134.8, 134.7, 134.6, 134.1, 132.2, 132.0, 129.5, 128.8, 128.7, 128.4, 128.3, 127.2, 126.5, 117.7, 117.4, 117.3, 111.5, 110.8, 110.7, 61.8, 61.6, 61.6, 52.2, 52.1, 43.4, 42.8, 42.7, 38.8, 35.8, 35.4, 14.7, 14.1, 13.7, 13.0, 12.4 ppm; **FTIR (neat) ν/cm^{-1}** 2980.75, 2360.66, 1698.37, 1386.29, 1077.40, 955.32;

HRMS (ESI$^+$) $C_{20}H_{21}N_2O_4$ $[M+H]^+$ requires 353.1496; found 353.1496.

Compound S25:

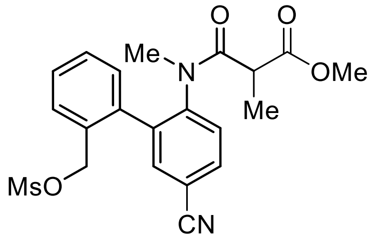

S25

Prepared according to General Procedure C with **S25-2** (310.0 mg, 0.878 mmol), methanesulfonic anhydride (184.0 mg, 1.06 mmol) and triethylamine (0.19 mL, 1.32 mmol). Purification by column chromatography, gradient eluting with ethyl acetate in petrol (25% to 50%), yielded product **S25** as an oil (210.0 mg, 55.6% yield). **^{1}H NMR** (400 MHz, $CDCl_3$), δ = 7.79 – 7.65 (m, 1H), 7.65 – 7.49 (m, 2H), 7.48 – 7.29 (m, 3H), 7.16 – 6.93 (m, 1H), 5.14 – 4.72 (m, 2H), 3.79 – 3.31 (m, 4H), 3.26 – 2.96 (m, 2H), 2.95 – 2.56 (m, 3H), 1.56 – 1.18 (m, 2H), 1.01 – 0.64 (m, 1H) ppm; **^{13}C NMR** (101 MHz, $CDCl_3$), δ = 170.3, 170.1, 170.0, 169.9, 169.8, 169.1, 168.3, 146.1, 146.0, 145.4, 145.2, 145.1, 138.8, 138.0, 137.8, 136.3, 136.3, 135.8, 135.6, 135.4, 135.2, 134.8, 134.6, 132.9, 132.7, 132.5, 132.3, 131.4, 130.8, 130.4, 130.0, 129.8, 129.4, 129.3, 129.1, 128.7, 128.5, 117.6, 117.2, 112.2, 112.0, 111.2, 111.0, 69.2, 68.2, 67.9, 67.5, 61.8, 52.3, 52.2, 52.2, 43.5, 43.2, 42.8, 42.8, 39.0, 37.5, 37.0, 36.0, 35.5, 14.8, 14.2, 13.2, 12.8 ppm; **FTIR (neat) ν/cm^{-1}** 2980.75, 2360.43, 1745.93, 1354.49, 1174.03, 931.43;

HRMS (ESI^{+}) $C_{21}H_{23}N_2O_6S$ $[M+H]^+$ requires 431.1271; found 431.1272.

Compound S26-1:

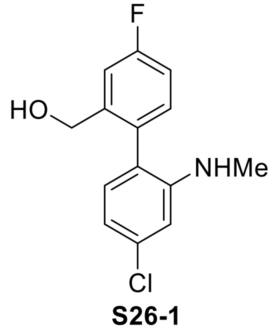

S26-1

Prepared according to General Procedure A with 4-chloro-2-iodo-*N*-methylaniline (1.10 g, 3.95 mmol), 5-fluorobenzo[c][1,2]oxaborol-1(3H)-ol (500.0 mg, 3.29 mmol), palladium acetate (72.0 mg, 0.33 mmol), triphenylphosphine (347.0 mg, 1.32 mmol), cesium carbonate (2.69 g, 8.23

mmol), and TBAB (107.2 mg, 0.33 mmol) in the mixed solvent of toluene and water (v/v = 1:1) . Purification by column chromatography, eluting with 15% ethyl acetate in petrol, yielded intermediate **S26-1** as an oil (820.0 mg, 94.1% yield).

^{1}H NMR (400 MHz, $CDCl_3$), δ = 7.21 (dd, J = 9.5, 2.7 Hz, 1H), 7.04 (dd, J = 8.4, 5.8 Hz, 1H), 6.97 (td, J = 8.3, 2.7 Hz, 1H), 6.79 (d, J = 7.9 Hz, 1H), 6.69 (dd, J = 7.9, 2.0 Hz, 1H), 6.60 (d, J = 2.1 Hz, 1H), 4.30 – 4.23 (m, 2H), 2.65 (s, 3H) ppm; **^{13}C NMR** (101 MHz, $CDCl_3$), δ = 164.1, 161.6, 147.4, 142.5 (d, J = 7.1 Hz), 135.0, 132.0 (d, J = 8.0 Hz), 130.7, 124.0, 117.4, 115.6 (d, J = 21.9 Hz), 115.1 (d, J = 21.2 Hz), 110.5, 62.9 (d, J = 1.5 Hz), 30.5 ppm; **^{19}F NMR** (377 MHz, $CDCl_3$), δ = -113.04; **FTIR (neat) ν/cm^{-1}** 2980.69, 2360.67, 1636.22, 1489.26, 1249.84, 953.29; **HRMS** (ESI$^+$) $C_{14}H_{14}ClFNO$ $[M+H]^+$ requires 266.0742; found 266.0742.

Compound S26-2:

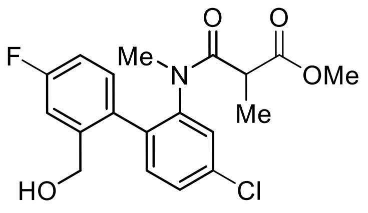

S26-2

Prepared according to General Procedure B with **S26-1** (820.0 mg, 3.09 mmol). Purification by column chromatography, gradient eluting with ethyl acetate in petrol (25% to 50%), yielded intermediate **S26-2** as an oil (710.0, 60.0% yield in 3 steps).

^{1}H NMR (500 MHz, $CDCl_3$), δ = 7.42 (dd, J = 15.4, 8.9 Hz, 1H), 7.33 (dd, J = 16.2, 8.9 Hz, 2H), 7.28 – 7.12 (m, 1H), 7.09 – 6.86 (m, 2H), 4.59 – 4.11 (m, 2H), 3.82 – 3.64 (m, 2H), 3.64 – 3.39 (m, 2H), 3.40 – 3.13 (m, 2H), 2.76 (d, J = 52.5 Hz, 1H), 1.56 – 1.11 (m, 2H), 1.02 – 0.75 (m, 1H) ppm; **^{13}C NMR** (126 MHz, $CDCl_3$), δ = 170.9, 170.6, 170.3, 170.1, 169.9, 169.5, 169.1, 164.2, 163.7, 163.5, 163.4, 161.8, 161.7, 161.5, 143.3, 142.9, 142.8, 142.3, 141.4, 141.1, 137.4, 136.7, 135.9, 135.5, 134.4, 134.2, 134.0, 133.6, 133.3, 133.0, 132.8, 132.5, 132.3, 131.8, 131.8, 131.6, 131.1, 130.9, 130.7, 130.3, 130.1, 129.8, 129.7, 129.3, 129.1, 128.5, 127.7, 127.6, 127.5, 127.4, 117.4, 116.4, 116.2, 116.0, 115.9, 115.7, 115.2, 115.1, 114.9, 114.4, 114.2, 114.1, 113.5, 113.3, 62.0, 61.8, 61.7, 52.5, 52.5, 52.4, 43.8, 43.7, 43.4, 43.3, 43.0, 39.8, 39.7, 39.4, 39.1, 37.9, 36.2, 35.8, 33.2, 29.6, 28.3, 25.0, 24.3, 23.7, 20.7, 20.0, 19.5, 17.4, 17.2, 15.0, 14.5, 14.5, 13.6, 13.4,

12.7 ppm; **^{19}F NMR** (377 MHz, $CDCl_3$), δ = -112.09, -112.19, -112.59, -113.44, -114.02 ppm; **FTIR (neat) ν/cm^{-1}** 2980.95, 2360.52, 1745.75, 1473.76, 1209.10, 1026.25;
HRMS (ESI$^+$) $C_{19}H_{20}ClFNO_4$ $[M+H]^+$ requires 380.1059; found 380.1058.

Compound S26:

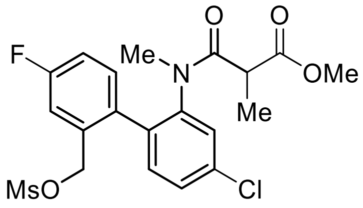

S26

Prepared according to General Procedure C with **S26-2** (700.0 mg, 1.84 mmol), methanesulfonic anhydride (384.6 mg, 2.20 mmol) and triethylamine (0.39 mL, 2.76 mmol). Purification by column chromatography, gradient eluting with ethyl acetate in petrol (25% to 50%), yielded product **S26** as an oil (531.0 mg, 62.4% yield).
^{1}H NMR (400 MHz, $CDCl_3$), δ = 7.56 – 7.31 (m, 1H), 7.31 – 7.16 (m, 3H), 7.15 – 7.04 (m, 1H), 7.02 – 6.85 (m, 1H), 5.09 – 4.65 (m, 2H), 3.77 – 3.44 (m, 3H), 3.43 – 3.23 (m, 1H), 3.22 – 2.99 (m, 2H), 2.98 – 2.49 (m, 4H), 1.38 – 0.98 (m, 2H), 0.86 – 0.66 (m, 1H) ppm; **^{13}C NMR** (101 MHz, $CDCl_3$), δ = 170.4, 170.1, 169.9, 169.6, 169.0, 168.3, 163.2, 163.1, 160.8, 160.8, 160.7, 142.9, 142.9, 142.3, 142.1, 135.3, 134.6, 134.3, 134.1, 134.0, 133.6, 133.3, 132.9, 132.8, 132.7, 132.6, 132.5, 132.1, 131.8, 131.5, 130.8, 130.5, 129.6, 129.2, 128.4, 127.8, 127.5, 127.4, 117.0, 116.8, 116.6, 116.6, 116.4, 116.1, 116.0, 115.9, 115.8, 115.5, 114.8, 68.8, 67.5, 67.3, 67.1, 52.1, 52.0, 43.5, 43.3, 43.2, 42.6, 40.5, 38.8, 37.2, 36.6, 35.8, 35.5, 31.2, 23.4, 20.4, 17.0, 16.8, 14.6, 14.2, 13.3, 13.2, 12.5 ppm; **^{19}F NMR** (377 MHz, $CDCl_3$), δ = -95.01 – -126.67 (m) ppm; **FTIR (neat) ν/cm^{-1}** 2980.96, 2360.45, 1655.91, 1355.97, 1195.16, 826.96;
HRMS (ESI$^+$) $C_{20}H_{22}ClFNO_6S$ $[M+H]^+$ requires 458.0835; found 458.0835.

Compound S27-1:

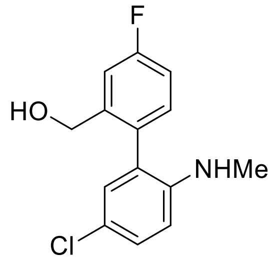

S27-1

Prepared according to General Procedure A with 4-chloro-2-iodo-*N*-methylaniline (1.10 g, 3.95 mmol), 5-fluorobenzo[c][1,2]oxaborol-1(3H)-ol (500.0 mg, 3.29 mmol), palladium acetate (72.0 mg, 0.33 mmol), triphenylphosphine (347.0 mg, 1.32 mmol), cesium carbonate (2.69 g, 8.23 mmol), and TBAB (107.2 mg, 0.33 mmol) in the mixed solvent of toluene and water (v/v = 1:1). Purification by column chromatography, eluting with 10% ethyl acetate in petrol, yielded intermediate **S27-1** as an oil (800.0 mg, 91.7% yield).

^{1}H NMR (400 MHz, $CDCl_3$), δ = 7.28 – 7.13 (m, 2H), 7.11 – 7.00 (m, 1H), 6.98 (dd, *J* = 8.3, 2.8 Hz, 1H), 6.89 (d, *J* = 2.5 Hz, 1H), 6.57 (d, *J* = 8.7 Hz, 1H), 4.33 – 4.21 (m, 2H), 2.65 (s, 3H) ppm; **^{13}C NMR** (101 MHz, $CDCl_3$), δ = 164.1, 161.7, 145.1, 142.5 (d, *J* = 7.0 Hz), 131.8 (d, *J* = 8.1 Hz), 129.5, 128.8, 127.3, 122.4, 115.7 (d, *J* = 21.9 Hz), 115.1 (d, *J* = 21.2 Hz), 111.6, 62.9 (d, *J* = 1.5 Hz), 30.7 pm; **^{19}F NMR** (400 MHz, $CDCl_3$), δ = – 112.9 ppm; **FTIR (neat) ν/cm^{-1}** 2980.85, 2360.86, 1770.79, 1508.21, 1313.11, 952.74;
HRMS (ESI^+) $C_{14}H_{14}ClFNO$ $[M+H]^+$ requires 266.0742; found 266.0743.

Compound S27-2:

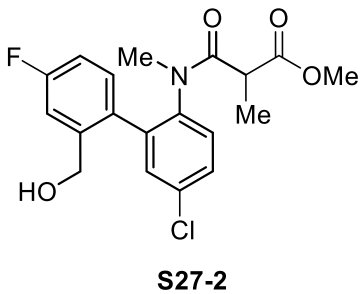

S27-2

Prepared according to General Procedure B with **S27-1** (800.0 mg, 3.01 mmol). Purification by column chromatography, gradient eluting with ethyl acetate in petrol (25% to 50%), yielded intermediate **S27-2** as an oil (501.0 mg, 43.9% yield in 3 steps).

^{1}**H NMR** (500 MHz, $CDCl_3$), δ = 7.44 – 7.32 (m, 1H), 7.31 – 7.19 (m, 2H), 7.19 – 7.07 (m, 1H), 7.05 – 6.81 (m, 2H), 4.90 – 4.10 (m, 2H), 3.71 – 3.53 (m, 2H), 3.53 – 3.33 (m, 2H), 3.27 – 2.96 (m, 2H), 2.69 (d, J = 55.8 Hz, 1H), 1.43 – 1.03 (m, 2H), 0.94 - 0.78 (m, 1H) ppm; ^{13}**C NMR** (126 MHz, $CDCl_3$), δ = 170.9, 170.7, 170.4, 170.2, 169.9, 169.7, 169.2, 164.8, 163.8, 163.6, 161.9, 161.8, 161.6, 143.3, 141.1, 140.8, 140.6, 140.5, 140.1, 140.0, 139.9, 139.1, 138.7, 135.2, 133.1, 133.0, 132.4, 132.1, 131.8, 131.7, 131.5, 131.3, 130.9, 130.8, 130.4, 130.2, 130.0, 130.0, 129.7, 129.6, 129.5, 129.4, 129.2, 129.0, 128.6, 128.5, 117.7, 116.6, 116.3, 116.1, 115.8, 115.6, 115.3, 115.1, 114.9, 114.6, 114.4, 114.3, 113.6, 113.4, 62.1, 62.0, 61.8, 52.6, 52.5, 43.8, 43.5, 43.4, 43.1, 39.9, 39.7, 39.5, 39.2, 36.4, 35.4, 15.1, 14.9, 14.1, 13.8, 13.7, 12.8 ppm; ^{19}**F NMR** (377 MHz, $CDCl_3$), δ = -111.85, -111.98, -112.37, -113.19, -113.75 ppm; **FTIR (neat) ν/cm^{-1}** 2980.71, 2360.46, 1683.31, 1474.64, 1339.04, 953.72;
HRMS (ESI$^+$) $C_{19}H_{20}ClFNO_4$ $[M+H]^+$ requires 380.1059; found 380.1060.

Compound S27:

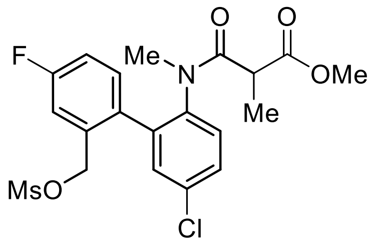

S27

Prepared according to General Procedure C with **S27-2** (500.0 mg, 1.31 mmol), methanesulfonic anhydride (273.8 mg, 1.57 mmol) and triethylamine (0.27 mL, 1.96 mmol). Purification by column chromatography, gradient eluting with ethyl acetate in petrol (25% to 50%), yielded product **S27** as an oil (492.0 mg, 82.1% yield).
^{1}**H NMR** (400 MHz, $CDCl_3$), δ = 7.60 – 7.33 (m, 1H), 7.31 – 7.18 (m, 3H), 7.17 – 7.06 (m, 1H), 7.05 – 6.90 (m, 1), 5.09 – 4.78 (m, 2H), 3.68 (d, J = 5.0 Hz, 1H), 3.63 (s, 1H), 3.57 – 3.32 (m, 2H), 3.23 – 2.97 (m, 2H), 2.95 – 2.43 (m, 4H), 1.44 – 1.13 (m, 2H), 0.86 (d, J = 7.0 Hz, 1H) ppm; ^{13}**C NMR** (101 MHz, $CDCl_3$), δ = 170.4, 170.3, 170.1, 170.0, 169.9, 169.7, 169.2, 168.5, 163.3, 163.2, 160.8, 160.8, 160.7, 140.5, 140.5, 140.2, 140.0, 139.8, 139.7, 138.3, 137.6, 137.2, 137.1, 135.1, 133.7, 133.6, 133.5, 133.5, 133.2, 133.1, 132.7, 132.7, 132.6, 132.6, 132.5, 132.2, 132.2, 132.0, 132.0, 131.6, 131.3, 130.8, 130.7, 130.6, 130.2, 129.6, 129.6, 129.3, 129.2, 128.8, 117.3, 117.0, 116.9, 116.8, 116.7, 116.6, 116.3, 116.2, 116.1, 115.9, 115.9, 115.6, 114.9, 114.7, 77.3, 77.0, 76.6,

68.8, 67.5, 67.2, 67.0, 52.1, 52.0, 51.6, 43.4, 43.1, 42.9, 42.6, 38.9, 37.2, 37.2, 36.5, 36.0, 35.5, 14.7, 14.1, 13.9, 13.7, 13.3, 13.2, 12.6 ppm; **^{19}F NMR** (377 MHz, $CDCl_3$), δ = -95.29 – -118.40 (m) ppm; **FTIR (neat) ν/cm^{-1}** 2981.44, 2361.12, 1655.72, 1356.11, 1175.09, 879.88; **HRMS** (ESI^+) $C_{20}H_{22}ClFNO_6S$ $[M+H]^+$ requires 458.0835; found 458.0830.

Compound 28:

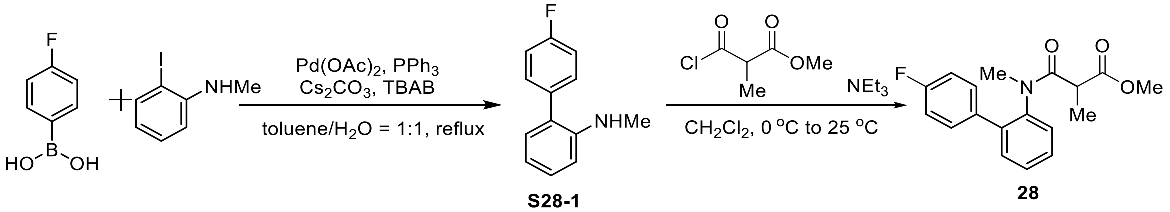

To a solution of 2-iodo-*N*-methylaniline (991.0 mg, 4.27 mmol) in 44.0 mL of mixed solvent toluene/H_2O (v/v = 1:1) were added sequentially (4-fluorophenyl)boronic acid (500.0 mg, 3.56 mmol), $Pd(OAc)_2$ (84.0 mg, 0.35 mmol), PPh_3(392.0 mg, 1.49 mmol), tetrabutylammonium bromide (120.0 mg, 0.37 mmol) and Cs_2CO_3 (3.04 g, 9.32 mmol). The resulting mixture was degassed and heated to reflux for 24 h. The solvent was removed under reduced pressure, the residue was purified by flash column chromatography (silica gel, 10% to 25% EtOAc in petroleum ether) to afford biaryl **S28-1** (709.0 mg, 99.0% yield) as a white foam. **^{1}H NMR** (400 MHz, $CDCl_3$), δ = 7.32 – 7.26 (m, 2H), 7.18 (ddd, *J* = 8.2, 7.5, 1.7 Hz, 1H), 7.06 – 7.00 (m, 2H), 6.96 (dd, *J* = 7.5, 1.5 Hz, 1H), 6.68 (td, *J* = 7.5, 1.2 Hz, 1H), 6.60 (dd, *J* = 8.1, 0.9 Hz, 1H), 3.75 (s, 1H), 2.71 (s, 3H) ppm; **^{13}C NMR** (101 MHz, $CDCl_3$), δ = 162.0 (d, *J* = 246.4 Hz), 146.1, 135.3 (d, *J* = 3.2 Hz), 131.0 (d, *J* = 7.9 Hz), 130.0, 128.8, 126.5, 116.8, 115.7 (d, *J* = 21.5 Hz), 109.8, 30.7 ppm; **FTIR (neat) ν/cm^{-1}** 2980.83, 2360.58, 1716.64, 1504.62, 1267.58, 967.77, 748.51; **HRMS** (ESI^+) $C_{13}H_{13}FN$ $[M+H]^+$ requires 202.1027; found 202.1027.

To a solution of **S28-1** (350.0 mg, 1.74 mmol) in 25.0 mL of dry dichloromethane, NEt_3 (0.73 mL, 5.22 mmol) and freshly prepared acyl chloride (3.48 mmol, prepared according to the General Procedure B) were added sequentially, the reaction mixture were stirred for 2 h at room temperature. Then, quenched the reaction with water, the aqueous phase was extracted with dichloromethane (20 mL x 2). The combined organic layers were washed with brine, dried over Na_2SO_4, concentrated and the crude product was purified by column chromatography to yield 201.0 mg (36.6% yield) of compound **28** as a white solid. **^{1}H NMR** (400 MHz, $CDCl_3$), δ = 7.44

– 7.31 (m, 6H), 7.31 – 7.24 (m, 1H), 7.22 – 7.12 (m, 3H), 7.05 (t, J = 8.7 Hz, 3H), 3.55 (s, 3H), 3.50 (s, 2H), 3.34 (q, J = 7.0 Hz, H), 3.20 – 3.15 (m, 0.67H), 3.14 (s, 3H), 2.93 (s, 2H), 1.29 (d, J = 7.0 Hz, 2H), 0.89 (d, J = 7.0 Hz, 3H) ppm; **^{13}C NMR** (101 MHz, $CDCl_3$), δ = 171.0, 170.9, 170.6, 169.5, 165.7, 163.6, 161.2, 161.1, 140.8, 140.3, 138.7, 138.6, 134.3, 134.2, 134.2, 134.1, 131.7, 131.1, 130.6, 130.5, 130.2, 130.1, 129.0, 128.9, 128.8, 128.8, 128.7, 128.3, 115.9, 115.7, 115.6, 115.4, 52.2, 52.1, 43.7, 43.3, 38.0, 37.2, 15.5, 13.6 ppm; **^{19}F NMR** (377 MHz, Tol – d^8) δ = -113.79, -114.42 ppm; **FTIR (neat) ν/cm^{-1}** 2979.63, 2360.44, 1659.77, 1345.66, 1078.30, 800.78; **HRMS** (ESI$^+$) $C_{18}H_{19}FNO_3$ [M+H]$^+$ requires 316.1343; found 316.1344.

4. General Procedure for the Counterion Mediated Cyclization (2, 9-34).

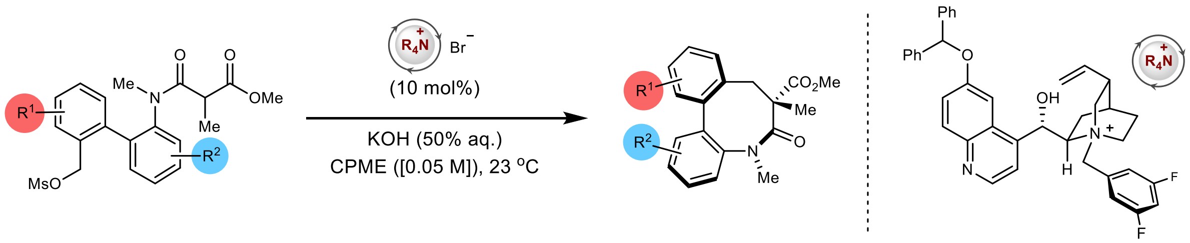

General Procedure D (preparation of racemic samples):

i) Preparation of racemic samples with the catalyst TBAB**.**

The appropriate starting material (0.05 mmol, 1.0 equiv.) and TBAB (1.7 mg, 0.005 mmol, 0.1 equiv.) were added to a 1.5 mL vial equipped with a magnetic stirrer bar under air. 1.0 mL CPME was added followed by KOH (50%. aq., w/w, 24 µL, 0.20 mmol, 4.0 equiv.). The reaction was stirred at 1400 rpm at 23 °C for the required reaction time. Hydrochloric acid (1.0 M aq., 0.2 mL, 4.0 equiv.) was added and the phases were separated and the aqueous phase was extracted with EtOAc (2 × 5.0 mL). The combined organic layers were concentrated in vacuo and purified by flash column chromatography using the appropriate eluent to give the corresponding racemic product with approximate 1:3 d.r..

ii) Preparation of racemic samples with the base LiHMDS.

The appropriate starting material (0.05 mmol, 1.0 equiv.) was added to a 1.5 mL vial equipped with a magnetic stirrer bar under nitrogen 1.0 mL dry THF was added, followed by LiHMDS (50 µL, 0.05 mmol, 1.0 equiv., solution 1.0 M in THF) at 0 °C. The reaction was stirred at 23 °C for the required reaction time. Water was added and the phases were separated and the aqueous phase was extracted with EtOAc (2 × 5.0 mL). The combined organic layers were concentrated in vacuo and purified by flash column chromatography using the appropriate eluent to give the corresponding racemic product with approximate > 20:1 d.r..

General Procedure E (Synthesis of enantioenriched products):

The appropriate starting material (0.10 mmol, 1.0 equiv.) and phase transfer catalyst **8** (6.8 mg, 0.01 mmol, 0.1 equiv.) were added to a 3.5 mL vial equipped with a magnetic stirrer bar under air. 2.0 mL CPME was added followed by KOH (50%. aq., w/w, 48 µL, 0.40 mmol, 4.0 equiv.). The reaction was stirred at 1400 rpm at 23 °C for the required reaction time. Hydrochloric acid (1.0 M aq., 0.40 mL, 4.0 equiv.) was added and the phases were separated and the aqueous phase was extracted with EtOAc (2 × 10.0 mL). The combined organic layers were concentrated in vacuo and purified by flash column chromatography using the appropriate eluent to give the corresponding enantioenriched product.

Compound 2:

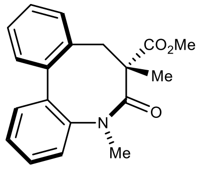

2

(white solid, 92% yield, 97:3 dr, 94% ee); **^{1}H NMR** (400 MHz, $CDCl_3$), δ = 7.64 (dd, J = 7.5, 1.2 Hz, 1H), 7.48 (dtd, J = 20.9, 7.5, 1.5 Hz, 2H), 7.41 – 7.31 (m, 4H), 7.28 (dd, J = 7.5, 1.4 Hz, 1H), 3.85 (s, 3H), 3.26 (d, J = 14.1 Hz, 1H), 2.94 (s, 3H), 2.22 (d, J = 14.1 Hz, 1H), 1.43 (s, 3H); **^{13}C NMR** (101 MHz, $CDCl_3$), δ = 174.1, 172.3, 141.9, 140.7, 138.6, 137.7, 130.6, 129.8, 129.1, 128.3, 128.3, 127.8, 127.2, 127.0, 57.1, 52.2, 41.0, 39.8, 22.4 ppm; **FTIR (neat) ν/cm^{-1}** 2980.67, 2360.59, 1595.58, 1479.81, 1271.63, 886.64;

HRMS (ESI^{+}) $C_{19}H_{20}NO_3$ $[M+H]^{+}$ requires 310.1438; found 310.1438.

m.p. = 126 °C

$\boldsymbol{\alpha_D^{25}}$ = − 97.0 (c = 0.5, $CHCl_3$);

Chiral HPLC: (Chiralpak IC, 50% $^{i-}$PrOH, 50% hexane, 1.00 mL min^{-1}, λ = 222 nm) τ_R (major) = 13.253 min, τ_R (minor) = 27.090 min.

Compound 9:

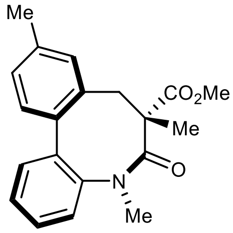

9

yellow oil, 65% yield, 87:13 dr, 87% ee); **^{1}H NMR** (400 MHz, $CDCl_3$), δ = 7.49 – 7.31 (m, 3H), 7.27 (dd, J = 7.6, 1.4 Hz, 1H), 7.23 (dd, J = 7.4, 1.8 Hz, 1H), 7.06 (q, J = 6.8, 6.0 Hz, 2H), 3.76 (s, 3H), 3.13 (d, J = 14.1 Hz, 1H), 2.85 (s, 3H), 2.31 (s, 3H), 2.09 (d, J = 14.1 Hz, 1H), 1.32 (s, 3H) ppm. **^{13}C NMR** (101 MHz, $CDCl_3$), δ = 174.2, 172.4, 143.1, 140.4, 138.1, 137.6, 135.8, 131.4, 129.9, 128.9, 128.2, 127.9, 127.8, 126.8, 57.1, 52.2, 41.0, 39.8, 22.5, 21.3 ppm; **FTIR (neat) ν/cm^{-1}** 2980.72, 2360.48, 1646.19, 1339.06, 1158.90, 954.87;

HRMS (ESI^{+}) $C_{20}H_{22}NO_3$ $[M+H]^{+}$ requires 324.1594; found 324.1595;

α_D^{25} = − 96.8 (c = 0.25, $CHCl_3$);

Chiral HPLC: (Chiralpak IC, 50% ^{i}PrOH, 50% hexane, 1.00 mL min^{-1}, λ = 222 nm) τ_R (major) = 11.027 min, τ_R (minor) = 16.133 min.

Compound 10:

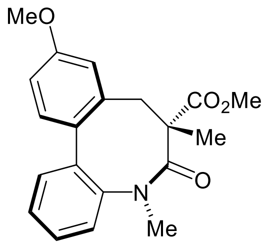

10

(white solid, 82.5% yield, 95:5 dr, 92.4% ee); **^{1}H NMR** (400 MHz, $CDCl_3$), δ = 7.38 (td, J = 7.5, 1.8 Hz, 1H), 7.33 (td, J = 7.3, 1.3 Hz, 1H), 7.27 (dd, J = 7.6, 1.6 Hz, 1H), 7.22 (dd, J = 7.5, 1.7 Hz, 1H), 7.14 – 7.08 (m, 2H), 7.08 (s, 1H), 6.77 (dd, J = 8.3, 2.7 Hz, 1H), 3.76 (s, 3H), 3.76 (s, 3H), 3.11 (d, J = 14.1 Hz, 1H), 2.84 (s, 3H), 2.09 (d, J = 13.9 Hz, 1H), 1.32 (s, 3H) ppm; **^{13}C NMR** (101 MHz, $CDCl_3$), δ = 174.0, 172.2, 159.5, 142.2, 140.4, 139.0, 131.0, 130.0, 128.8, 128.2, 127.9, 127.8, 116.0, 112.9, 57.0, 55.1, 52.2, 41.2, 39.8, 22.5 ppm; **FTIR (neat) ν/cm^{-1}** 2980.68, 2360.70, 1683.71, 1418.52, 1206.65, 955.82;

HRMS (ESI^+) $C_{20}H_{22}NO_4$ $[M+H]^+$ requires 340.1543; found 340.1544;

m.p. = 112 -113 ^{o}C;

α_D^{25} = 68.2 (c = 0.5, $CHCl_3$);

Chiral HPLC: (Chiralpak IC, 3% ^{i}PrOH, 97% hexane, 1.00 mL min^{-1}, λ = 222 nm) τ_R (major) = 22.433 min, τ_R (minor) = 17.600 min.

Compound 11:

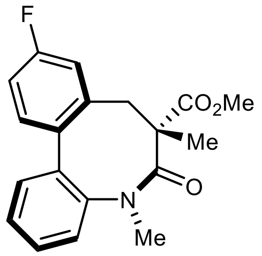

11

(white solid, 99% yield, 98:2 dr, 96.7% ee); **^{1}H NMR** (400 MHz, $CDCl_3$), δ = 7.42 (td, J = 7.6, 1.7 Hz, 1H), 7.36 (td, J = 7.5, 1.4 Hz, 1H), 7.32 – 7.25 (m, 2H), 7.22 (dd, J = 7.5, 1.6 Hz, 1H), 7.15 (dd, J = 8.4, 5.7 Hz, 1H), 6.94 (td, J = 8.4, 2.7 Hz, 1H), 3.77 (s, 3H), 3.12 (d, J = 14.0 Hz, 1H), 2.85 (s, 3H), 2.10 (d, J = 14.0 Hz, 1H), 1.33 (s, 3H) ppm; **^{13}C NMR** (101 MHz, $CDCl_3$), δ = 173.8, 172.0, 162.5 (d, J = 246.7 Hz), 142.0, 140.1 (d, J = 8.3 Hz), 139.7, 134.6 (d, J = 3.1 Hz), 129.9, 129.3, 128.4, 128.3, 127.9, 117.7 (d, J = 22.0 Hz), 114.2 (d, J = 21.6 Hz), 57.0, 52.3, 40.9 (d, J = 1.2 Hz), 39.9, 22.4 ppm; **^{19}F NMR** (377 MHz, $CDCl_3$), δ = -113.66 ppm (td, J = 9.3, 5.8 Hz); **FTIR (neat) ν/cm^{-1}** 2980.73, 2360.59, 1739.26, 1642.92, 1202.59, 763.58;

HRMS (ESI$^+$) $C_{19}H_{19}FNO_3$ $[M+H]^+$ requires 328.1343; found 328.1343;

m.p. = 111 − 112 ºC;

α_D^{25} = − 174.4 (c = 0.5, $CHCl_3$);

Chiral HPLC: (Chiralpak IC, 50% $^{i-}$PrOH, 50% hexane, 0.70 mL min^{-1}, λ = 222 nm) τ_R (major) = 18.207 min, τ_R (minor) = 25.530 min.

Compound 12:

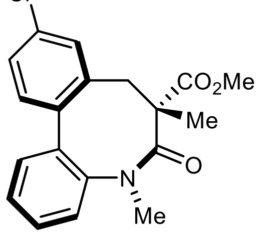

12

(white solid, 93.0% yield, 96:4 dr, 95.5% ee); **^{1}H NMR** (400 MHz, $CDCl_3$), δ = 7.53 (d, J = 2.2 Hz, 1H), 7.43 (td, J = 7.6, 1.8 Hz, 1H), 7.37 (td, J = 7.5, 1.4 Hz, 1H), 7.29 (dd, J = 7.8, 1.3 Hz, 1H), 7.26 – 7.18 (m, 2H), 7.12 (d, J = 8.1 Hz, 1H), 3.77 (s, 3H), 3.10 (d, J = 14.1 Hz, 1H), 2.86 (s,

3H), 2.08 (d, J = 14.1 Hz, 1H), 1.32 (s, 3H) ppm; **^{13}C NMR** (101 MHz, $CDCl_3$), δ = 173.8, 171.9, 141.9, 139.6, 139.6, 137.1, 134.1, 130.8, 129.7, 129.5, 128.5, 128.1, 128.0, 127.4, 56.9, 52.3, 40.8, 39.9, 22.4 ppm; **FTIR (neat) ν/cm^{-1}** 2980.66, 2360.49, 1683.73, 1445.65, 1094.63, 823.95;

HRMS (ESI^+) $C_{19}H_{19}ClNO_3$ $[M+H]^+$ requires 344.1048; found 344.1049;

m.p. = 133 - 134 °C;

α_D^{25} = − 130.0 (c = 0.5, $CHCl_3$);

Chiral HPLC: (Chiralpak IC, 50% ^{i}PrOH, 50% hexane, 1.00 mL min^{-1}, λ = 222 nm) τ_R (major) = 10.579 min, τ_R (minor) = 14.910 min.

Compound 13:

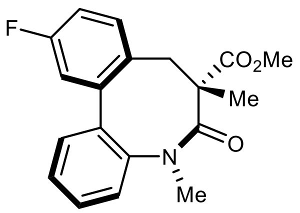

13

(colorless oil, 85.3% yield, 97:3 dr, 92.0% ee); **^{1}H NMR** (400 MHz, $CDCl_3$), δ = 7.52 (dd, J = 8.6, 5.7 Hz, 1H), 7.44 (td, J = 7.6, 1.7 Hz, 1H), 7.37 (td, J = 7.5, 1.5 Hz, 1H), 7.33 – 7.28 (m, 1H), 7.27 – 7.21 (m, 1H), 6.97 (td, J = 8.6, 2.7 Hz, 1H), 6.90 (dd, J = 8.8, 2.8 Hz, 1H), 3.75 (s, 3H), 3.14 (d, J = 14.2 Hz, 1H), 2.87 (s, 3H), 2.06 (d, J = 14.3 Hz, 1H), 1.32 (s, 3H) ppm; **^{13}C NMR** (101 MHz, $CDCl_3$), δ = 174.0, 172.2, 161.7 (d, J = 246.0 Hz), 141.8, 140.3 (d, J = 8.3 Hz), 139.7 (d, J = 2.0 Hz), 133.6 (d, J = 3.2 Hz), 132.3 (d, J = 8.3 Hz), 129.6, 128.4, 128.1, 115.1 (d, J = 20.7 Hz), 113.9 (d, J = 21.9 Hz), 57.0, 52.3, 40.2, 39.9, 22.4 ppm; **^{19}F NMR** (377 MHz, $CDCl_3$), δ = -115.45; **FTIR (neat) ν/cm^{-1}** 2980.69, 2360.36, 1644.28, 1446.90, 1047.10, 766.73;

HRMS (ESI^+) $C_{19}H_{19}FNO_3$ $[M+H]^+$ requires 328.1343; found 328.1342;

α_D^{25} = − 82.2 (c = 0.5, $CHCl_3$);

Chiral HPLC: (Chiralpak IC, 50% ^{i}PrOH, 50% hexane, 1.00 mL min^{-1}, λ = 222 nm) τ_R (major) = 7.993 min, τ_R (minor) = 16.877 min.

Compound 14:

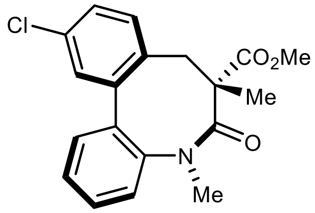

14

(white foam, 90.1% yield, 97:3 dr, 93.5% ee); **^{1}H NMR** (400 MHz, $CDCl_3$), δ = 7.48 (d, J = 8.2 Hz, 1H), 7.43 (td, J = 7.6, 1.7 Hz, 1H), 7.37 (td, J = 7.5, 1.4 Hz, 1H), 7.30 (dd, J = 7.8, 1.3 Hz, 1H), 7.24 (td, J = 7.8, 7.3, 2.0 Hz, 2H), 7.20 – 7.17 (m, 1H), 3.75 (s, 3H), 3.13 (d, J = 14.1 Hz, 1H), 2.87 (s, 3H), 2.06 (d, J = 14.2 Hz, 1H), 1.32 (s, 3H) ppm; **^{13}C NMR** (101 MHz, $CDCl_3$), δ = 173.9, 172.0, 141.8, 140.2, 139.4, 136.4, 132.8, 132.0, 129.6, 129.6, 128.5, 128.4, 128.0, 126.9, 57.0, 52.3, 40.3, 40.0, 22.4 ppm; **FTIR (neat) ν/cm^{-1}** 2980.68, 2360.56, 1683.17, 1445.48, 1097.81, 764.50;

HRMS (ESI$^+$) $C_{19}H_{19}ClNO_3$ $[M+H]^+$ requires 344.1048; found 344.1050;

$\boldsymbol{\alpha_D^{25}}$ = −107.2 (c = 0.25, $CHCl_3$)

Chiral HPLC: (Chiralpak IC, 50% $^{i-}$PrOH, 50% hexane, 1.00 mL min^{-1}, λ = 222 nm) τ_R (major) = 11.250 min, τ_R (minor) = 26.560 min.

Compound 15:

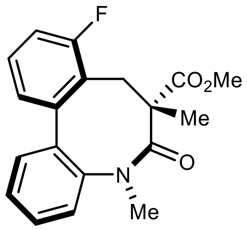

15

(white solid, 85% yield, 92:8 dr, 91.1% ee); **^{1}H NMR** (400 MHz, $CDCl_3$), δ = 7.43 (td, J = 7.7, 1.7 Hz, 1H), 7.35 (td, J = 7.5, 1.3 Hz, 1H), 7.28 (dd, J = 7.8, 1.2 Hz, 1H), 7.23 (td, J = 7.9, 5.4 Hz, 1H), 7.15 (dd, J = 7.6, 1.6 Hz, 1H), 7.08 – 6.92 (m, 2H), 3.71 (s, 3H), 3.25 (d, J = 14.5 Hz, 1H), 2.97 (s, 3H), 1.94 (dd, J = 14.7, 1.6 Hz, 1H), 1.34 (s, 3H) ppm; **^{13}C NMR** (101 MHz, $CDCl_3$), δ = 173.4, 171.9, 160.9 (d, J = 317.0 Hz) 141.6, 141.4, 138.3, 130.2, 129.5, 128.6 (d, J = 9.1 Hz), 128.3, 127.6, 125.1 (d, J = 15.5 Hz), 122.8 (d, J = 3.2 Hz), 115.5 (d, J = 23.8 Hz), 56.6, 52.2, 40.2,

34.0, 23.0 ppm; **^{19}F NMR** (376 MHz, $CDCl_3$), δ = -114.61; **FTIR (neat) ν/cm^{-1}** 2980.69, 2360.45, 1698.37, 1437.33, 1158.80, 759.76;

HRMS (ESI^+) $C_{19}H_{19}FNO_3$ $[M+H]^+$ requires 328.1343; found 328.1340;

m.p. = 129 -130 °C;

$\boldsymbol{\alpha_D^{25}}$ = − 93.6 (c = 0.5, $CHCl_3$);

Chiral HPLC: (Chiralpak IC, 50% ^{i}-PrOH, 50% hexane, 1.00 mL min^{-1}, λ = 222 nm) τ_R (major) = 10.640 min, τ_R (minor) = 16.283 min.

Compound 16:

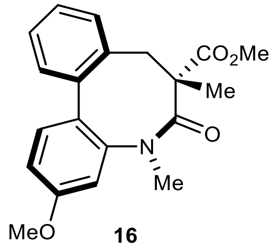

16

(white form, 86.4% yield, 97:3 dr, 96.2% ee); **^{1}H NMR** (400 MHz, $CDCl_3$), δ = 7.59 (dd, J = 7.4, 1.3 Hz, 1H), 7.31 (dtd, J = 16.6, 7.3, 1.5 Hz, 2H), 7.25 – 7.16 (m, 2H), 6.98 (dd, J = 8.5, 2.6 Hz, 1H), 6.87 (d, J = 2.6 Hz, 1H), 3.88 (s, 3H), 3.83 (s, 3H), 3.22 (d, J = 14.0 Hz, 1H), 2.92 (s, 3H), 2.25 (d, J = 14.0 Hz, 1H), 1.44 (s, 3H) ppm; **^{13}C NMR** (101 MHz, $CDCl_3$), δ = 174.1, 172.2, 160.0, 142.8, 138.4, 138.0, 133.1, 130.6, 130.5, 128.1, 127.3, 127.1, 113.8, 113.2, 57.2, 55.6, 52.2, 41.1, 39.8, 22.8 ppm;

HRMS (ESI^+) $C_{20}H_{22}NO_4$ $[M+H]^+$ requires 340.1543; found 340.1544;

$\boldsymbol{\alpha_D^{25}}$ = − 64.2 (c = 0.5, $CHCl_3$);

Chiral HPLC: (Chiralpak IC, 50% ^{i}-PrOH, 50% hexane, 1.00 mL min^{-1}, λ = 222 nm) τ_R (major) = 12.697 min, τ_R (minor) = 32.217 min.

Compound 17:

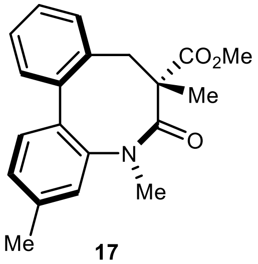

17

(white solid, 74.6% yield, 97:3 dr, 95.5% ee); **^{1}H NMR** (400 MHz, $CDCl_3$), δ = 7.52 (dd, J = 7.3, 1.9 Hz, 1H), 7.26 (td, J = 7.4, 1.7 Hz, 1H), 7.22 (td, J = 7.3, 1.6 Hz, 1H), 7.18 – 7.14 (m, 2H), 7.12 (d, J = 7.7 Hz, 1H), 7.09 (dt, J = 1.6, 0.7 Hz, 1H), 3.76 (s, 3H), 3.14 (d, J = 13.9 Hz, 1H), 2.84 (s, 3H), 2.58 – 2.26 (m, 3H), 2.14 (d, J = 13.9 Hz, 1H), 1.35 (s, 3H) ppm; **^{13}C NMR** (101 MHz, $CDCl_3$), δ = 174.2, 172.3, 141.8, 139.2, 138.7, 137.8, 137.7, 130.6, 129.5, 129.1, 128.2, 128.1, 127.1, 127.1, 57.1, 52.2, 41.0, 39.9, 22.6, 21.0 ppm; **FTIR (neat) ν/cm^{-1}** 2980.68, 2360.79, 1645.79, 1211.21, 1137.07, 771.30;

HRMS (ESI$^+$) $C_{20}H_{22}NO_3$ $[M+H]^+$ requires 324.1594; found 324.1594;

m.p. = 118 -119 °C;

$\boldsymbol{\alpha_D^{25}}$ = − 103.4 (c = 0.5, $CHCl_3$);

Chiral HPLC: (Chiralpak IC, 50% ^{i}PrOH, 50% hexane, 1.00 mL min^{-1}, λ = 222 nm) τ_R (major) = 12.533 min, τ_R (minor) = 34.623 min.

Compound 18:

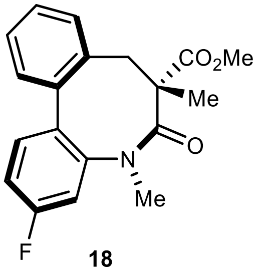

18

(white solid, 90.2% yield, 94:6 dr, 97.1% ee); **^{1}H NMR** (400 MHz, $CDCl_3$), δ = 7.57 – 7.50 (m, 1H), 7.29 (td, J = 7.5, 1.6 Hz, 1H), 7.26 – 7.20 (m, 2H), 7.14 (dd, J = 7.3, 1.7 Hz, 1H), 7.09 (td, J = 8.3, 2.6 Hz, 1H), 7.02 (dd, J = 9.0, 2.6 Hz, 1H), 3.76 (s, 3H), 3.19 (d, J = 14.1 Hz, 1H), 2.84 (s, 3H), 2.13 (d, J = 14.1 Hz, 1H), 1.36 (s, 3H) ppm; **^{13}C NMR** (101 MHz, $CDCl_3$), δ = 173.9, 172.2, 162.5 (d, J = 249.1 Hz), 143.1 (d, J = 9.5 Hz), 137.8, 137.7, 136.9 (d, J = 3.6 Hz), 131.1 (d, J =

8.7 Hz), 130.7, 128.6, 127.3, 127.2, 115.6 (d, J = 21.1 Hz), 114.8 (d, J = 22.3 Hz), 57.1, 52.3, 41.0, 39.8, 22.6 ppm; **^{19}F NMR** (377 MHz, $CDCl_3$), δ = -111.80 ppm; **FTIR (neat) ν/cm^{-1}** 2980.72, 2360.77, 1646.91, 1210.25, 1088.77, 768.27;

HRMS (ESI$^+$) $C_{19}H_{19}FNO_3$ $[M+H]^+$ requires 328.1343; found 328.1339;

m.p. = 144 -145 °C;

$\boldsymbol{\alpha_D^{25}}$ = − 154.2 (c = 0.5, $CHCl_3$);

Chiral HPLC: (Chiralpak IC, 50% ^{i}PrOH, 50% hexane, 1.00 mL min^{-1}, λ = 222 nm) τ_R (major) = 11.430 min, τ_R (minor) = 32.350 min.

Compound 19:

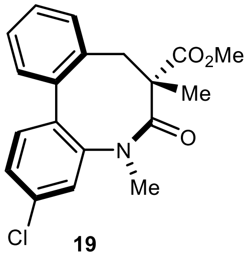

19

(white solid, 98% yield, 92:8 dr, 94% ee); **^{1}H NMR** (400 MHz, $CDCl_3$), δ = 7.54 (dd, J = 7.6, 1.1 Hz, 1H), 7.35 (dd, J = 8.2, 2.1 Hz, 1H), 7.32 – 7.27 (m, 2H), 7.24 (td, J = 7.5, 1.4 Hz, 1H), 7.21 – 7.18 (m, 1H), 7.14 (dd, J = 7.4, 1.4 Hz, 1H), 3.76 (s, 3H), 3.19 (d, J = 14.1 Hz, 1H), 2.84 (s, 3H), 2.12 (d, J = 14.2 Hz, 1H), 1.36 (s, 3H) ppm; **^{13}C NMR** (101 MHz, $CDCl_3$), δ = 173.8, 172.2, 143.1, 139.4, 137.7, 137.5, 134.3, 130.9, 130.8, 128.7, 128.6, 127.9, 127.3, 127.0, 57.0, 52.3, 40.9, 39.9, 22.5 ppm; **FTIR (neat) ν/cm^{-1}** 2980.75, 2360.99, 1656.75, 1388.16, 11986.19, 7776.55;

HRMS (ESI$^+$) $C_{19}H_{19}ClNO_3$ $[M+H]^+$ requires 344.1048; found 344.1049;

m.p. = 118 -119 °C;

$\boldsymbol{\alpha_D^{25}}$ = − 97.0 (c = 0.5, $CHCl_3$);

Chiral HPLC: (Chiralpak ADH, 3% ^{i}PrOH, 97% hexane, 1.00 mL min^{-1}, λ = 222 nm) τ_R (major) = 16.863 min, τ_R (minor) = 13.870 min.

Compound 20:

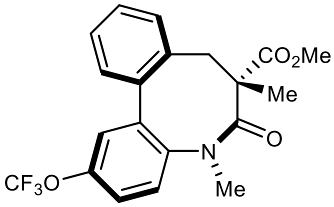

20

(white solid, 63.0% yield, 93:7 dr, 96.5% ee); **^{1}H NMR** (400 MHz, $CDCl_3$), δ = 7.59 – 7.53 (m, 1H), 7.35 – 7.33 (m, 1H), 7.32 (d, J = 2.3 Hz, 1H), 7.31 – 7.23 (m, 2H), 7.20 – 7.16 (m, 1H), 7.12 (dd, J = 2.8, 1.0 Hz, 1H), 3.76 (s, 3H), 3.20 (d, J = 14.2 Hz, 1H), 2.84 (s, 3H), 2.10 (d, J = 14.2 Hz, 1H), 1.34 (s, 3H) ppm; **^{13}C NMR** (101 MHz, $CDCl_3$), δ = 173.8, 172.2, 148.3, 148.3, 142.8, 140.4, 137.6, 137.3, 130.9, 129.5, 129.0, 127.4, 126.8, 124.1, 121.8, 121.6, 121.2, 119.0, 116.7, 56.9, 52.3, 40.9, 39.9, 22.4 ppm; **^{19}F NMR** (377 MHz, $CDCl_3$), δ = -57.82; **FTIR (neat) ν/cm^{-1}** 2980.73, 2360.75, 1698.52, 1254.73, 1163.60, 952.43;

HRMS (ESI^+) $C_{20}H_{19}F_3NO_4$ $[M+H]^+$ requires 394.1261; found 394.1258;

m.p. = 154 -155 °C;

$\boldsymbol{\alpha_D^{25}}$ = − 84.8 (c = 0.5, $CHCl_3$);

Chiral HPLC: (Chiralpak IC, 50% ^{i}PrOH, 50% hexane, 1.00 mL min^{-1}, λ = 222 nm) τ_R (major) = 8.577 min, τ_R (minor) = 17.580 min.

Compound 21:

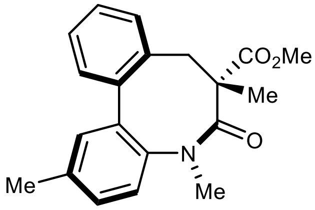

21

(white foam, 95% yield, 97:3 dr, 91.4% ee); **^{1}H NMR** (400 MHz, $CDCl_3$), δ = 7.57 – 7.47 (m, 1H), 7.30 – 7.22 (m, 2H), 7.21 – 7.13 (m, 3H), 7.08 – 7.03 (m, 1H), 3.75 (s, 3H), 3.14 (d, J = 13.9 Hz, 1H), 2.82 (s, 3H), 2.35 (s, 3H), 2.14 (d, J = 14.1 Hz, 1H), 1.34 (s, 3H); **^{13}C NMR** (101 MHz, $CDCl_3$), δ = 174.2, 172.4, 140.4, 139.4, 138.8, 138.3, 137.7, 130.6, 130.3, 129.7, 128.2, 127.6, 127.1, 126.9, 57.1, 52.2, 41.0, 39.8, 22.5, 21.0 ppm; **FTIR (neat) ν/cm^{-1}** 2980.66, 2360.34, 1683.73, 1418.13, 1206.91, 754.48;

HRMS (ESI^+) $C_{20}H_{22}NO_3$ $[M+H]^+$ requires 324.1594; found 324.1594;

α_D^{25} = −142.0 (c = 0.5, $CHCl_3$);

Chiral HPLC: (Chiralpak IC, 50% $^{i-}$PrOH, 50% hexane, 1.00 mL min^{-1}, λ = 222 nm) τ_R (major) = 14.383 min, τ_R (minor) = 22.803 min.

Compound 22:

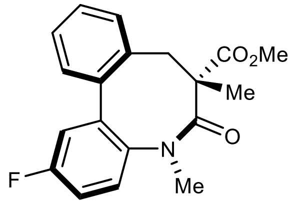

22

(white solid, 84.3% yield, 86:17 dr, 94.3% ee); **^{1}H NMR** (400 MHz, $CDCl_3$), δ = 7.55 (d, J = 7.6 Hz, 1H), 7.34 – 7.22 (m, 3H), 7.20 – 7.15 (m, 1H), 7.10 (td, J = 8.3, 3.0 Hz, 1H), 6.96 (dd, J = 8.5, 3.0 Hz, 1H), 3.76 (s, 3H), 3.19 (d, J = 14.1 Hz, 1H), 2.82 (s, 3H), 2.12 (d, J = 14.1 Hz, 1H), 1.34 (s, 3H) ppm; **^{13}C NMR** (101 MHz, $CDCl_3$), δ = 173.9, 172.3, 161.5 (d, J = 250.2 Hz), 142.9 (d, J = 8.4 Hz), 138.1 (d, J = 3.3 Hz), 137.6 (d, J = 1.3 Hz), 137.6, 130.8, 129.7 (d, J = 8.9 Hz), 128.8, 127.3, 126.8, 116.5 (d, J = 22.4 Hz), 116.1 (d, J = 22.6 Hz), 56.9, 52.2, 40.9, 39.9, 22.4 ppm; **^{19}F NMR** (377 MHz, $CDCl_3$), δ = -112.26 (td, J = 8.4, 5.2 Hz); **FTIR (neat) ν/cm^{-1}** 2980.72, 2360.83, 1771.59, 1450.75, 1124.61, 768.47;

HRMS (ESI^+) $C_{19}H_{19}FNO_3$ $[M+H]^+$ requires 328.1343; found 328.1343;

m.p. = 124 -125 ^{o}C.

α_D^{25} **=** − 83.1 (c = 0.5, $CHCl_3$);

Chiral HPLC: (Chiralpak IC, 50% $^{i-}$PrOH, 50% hexane, 1.00 mL min^{-1}, λ = 222 nm) τ_R (major) = 11.420 min, τ_R (minor) = 23.687 min.

Compound 23:

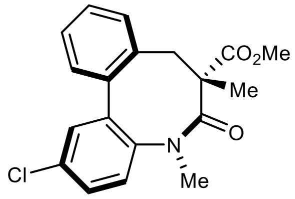

23

(white solid, 99.0% yield, 93:7 dr, 96% ee); **^{1}H NMR** (400 MHz, $CDCl_3$), δ = 7.57 – 7.51 (m, 1H), 7.38 (dd, J = 8.4, 2.4 Hz, 1H), 7.30 (td, J = 7.5, 1.5 Hz, 1H), 7.27 – 7.21 (m, 3H), 7.16 (dd, J

= 7.5, 1.5 Hz, 1H), 3.76 (s, 3H), 3.19 (d, J = 14.2 Hz, 1H), 2.82 (s, 3H), 2.13 (d, J = 14.2 Hz, 1H), 1.34 (s, 3H) ppm; **^{13}C NMR** (101 MHz, $CDCl_3$), δ = 173.9, 172.2, 142.5, 140.6, 137.6, 137.4, 133.9, 130.8, 129.8, 129.2, 129.2, 128.9, 127.3, 126.8, 57.0, 52.3, 40.9, 39.9, 22.4 ppm; **FTIR (neat) ν/cm^{-1}** 2980.71, 2359.93, 1647.29, 1449.61, 1159.37, 954.29;

HRMS (ESI$^+$) $C_{19}H_{19}ClNO_3$ [M+H]$^+$ requires 344.1048; found 344.1049;

m.p. = 156 -157 °C;

α_D^{25} = − 84.6 (c = 0.5, $CHCl_3$);

Chiral HPLC: (Chiralpak IC, 50% ^{i}-PrOH, 50% hexane, 1.00 mL min^{-1}, λ = 222 nm) τ_R (major) = 11.600 min, τ_R (minor) = 21.437 min.

Compound 24:

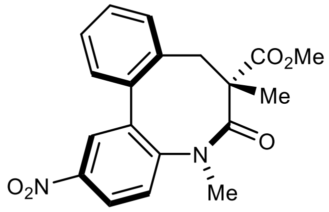

24

(yellow foam, 55.0% yield, 37:63 dr, 95.3% ee); **^{1}H NMR** (400 MHz, $CDCl_3$), δ = 8.28 (dd, J = 8.7, 2.7 Hz, 1H), 8.21 (dd, J = 8.6, 2.7 Hz, 2H), 8.16 (d, J = 2.6 Hz, 2H), 7.58 (dd, J = 7.6, 1.0 Hz, 1H), 7.49 (d, J = 8.7 Hz, 1H), 7.36 (td, J = 7.6, 1.6 Hz, 1H), 7.33 – 7.27 (m, 5.6H), 7.23 – 7.16 (m, 6.6H), 3.77 (s, 3H), 3.57 (s, 5.6H), 3.25 (d, J = 14.3 Hz, 1H), 3.20 (d, J = 14.5 Hz, 2H), 2.89 (s, 3H), 2.85 (s, 5.6H), 2.49 (d, J = 14.5 Hz, 2H), 2.10 (d, J = 14.3 Hz, 1H), 1.58 (s, 5.6H), 1.33 (s, 3H) ppm; **^{13}C NMR** (126 MHz, $CDCl_3$), δ = 173.7, 173.4, 171.9, 169.6, 147.7, 147.4, 147.2, 146.9, 142.9, 142.4, 137.5, 136.5, 136.5, 136.4, 131.0, 130.0, 129.6, 129.5, 129.1, 129.0, 127.8, 127.7, 127.3, 127.0, 125.2, 124.6, 124.2, 124.0, 57.0, 54.5, 52.9, 52.4, 41.0, 40.9, 40.0, 39.1, 26.8, 22.5 ppm; **FTIR (neat) ν/cm^{-1}** 2980.70, 2360.37, 1735.95, 1507.44, 1348.98, 1134.10, 760.60;

α_D^{25} = − 53.0 (c = 0.5, $CHCl_3$);

HRMS (ESI$^+$) $C_{19}H_{19}N_2O_5$ [M+H]$^+$ requires 355.1288; found 355.1288;

Chiral HPLC: (Chiralpak IC, 50% ^{i}-PrOH, 50% hexane, 1.00 mL min^{-1}, λ = 222 nm) τ_R (major) = 16.010 min, τ_R (minor) = 39.767 min.

Compound 25:

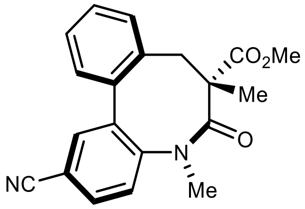

25

(white solid, 62.8% yield, 70:30 dr, 96.5:3.5 ee); **^{1}H NMR** (400 MHz, $CDCl_3$), δ = 7.72 (dd, J = 8.2, 2.0 Hz, 1H), 7.61 – 7.54 (m, 2H), 7.43 (d, J = 8.2 Hz, 1H), 7.34 (td, J = 7.6, 1.6 Hz, 1H), 7.29 – 7.25 (m, 1H), 7.16 (dd, J = 7.5, 1.7 Hz, 1H), 3.76 (s, 3H), 3.23 (d, J = 14.2 Hz, 1H), 2.87 (s, 3H), 2.07 (d, J = 14.3 Hz, 1H), 1.32 (s, 3H) ppm; **^{13}C NMR** (101 MHz, $CDCl_3$), δ = 173.53, 171.99, 146.15, 142.36, 137.54, 136.37, 133.77, 132.77, 130.97, 129.39, 129.05, 129.03, 127.66, 126.97, 117.65, 112.34, 57.04, 52.42, 40.87, 39.96, 22.46 ppm; **FTIR (neat) ν/cm^{-1}** 2980.78, 2360.38, 1647.33, 1419.27, 1100.81, 757.86;

HRMS (ESI^{+}) $C_{20}H_{19}N_2O_3$ $[M+H]^{+}$ requires 335.1390; found 335.1391;

m.p. = 165 -166 °C;

$\boldsymbol{\alpha_D^{25}}$ **=** − 162.4 (c = 0.5, $CHCl_3$);

Chiral HPLC: (Chiralpak IC, 50% ^{i}PrOH, 50% hexane, 1.00 mL min^{-1}, λ = 222 nm) τ_R (major) = 19.950 min, τ_R (minor) = 45.377 min.

Compound 26:

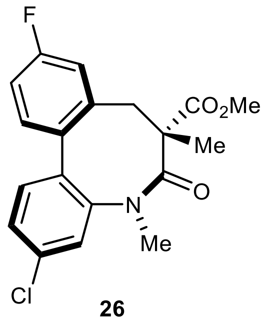

26

(white solid, 77.3% yield, 95:5 dr, 98.5% ee); **^{1}H NMR** (400 MHz, $CDCl_3$), δ = 7.35 (dd, J = 8.2, 2.2 Hz, 1H), 7.31 (d, J = 2.1 Hz, 1H), 7.28 (dd, J = 9.7, 2.7 Hz, 1H), 7.18 (d, J = 8.2 Hz, 1H), 7.12 (dd, J = 8.4, 5.6 Hz, 1H), 6.94 (td, J = 8.4, 2.7 Hz, 1H), 3.77 (s, 4H), 3.15 (d, J = 14.1 Hz, 1H), 2.85 (s, 3H), 2.10 (d, J = 14.1 Hz, 1H), 1.36 (s, 3H) ppm; **^{13}C NMR** (101 MHz, $CDCl_3$), δ = 173.6, 171.9, 163.9, 161.5, 143.1, 140.1, 140.0, 138.3, 134.6, 133.4, 133.4, 131.0, 128.7, 128.5, 128.4,

128.0, 118.0, 117.8, 114.5, 114.3, 56.9, 52.4, 40.9, 40.8, 40.0, 22.4 ppm; **^{19}F NMR** (377 MHz, $CDCl_3$), δ = -112.89; **FTIR (neat) ν/cm^{-1}** 2949.02, 2354.48, 1650.12, 1472.81, 1268.84, 1102.74, 836.97;

HRMS (ESI^+) $C_{19}H_{18}ClFNO_3$ $[M+H]^+$ requires 362.0954; found 362.0955;

m.p. = 147 -148 °C;

α_D^{25} = − 141.6 (c = 0.5, $CHCl_3$);

Chiral HPLC: (Chiralpak IC, 50% $^{i-}$PrOH, 50% hexane, 0.70 mL min^{-1}, λ = 222 nm) τ_R (major) = 15.113 min, τ_R (minor) = 36.263 min.

Compound 27:

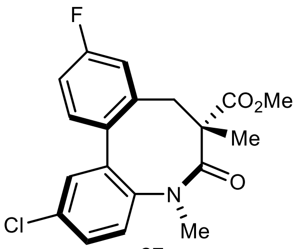

27

(white solid, 86.4% yield, 95:5 dr, 97.7% ee); **^{1}H NMR** (400 MHz, $CDCl_3$), δ = 7.39 (dd, *J* = 8.6, 2.4 Hz, 1H), 7.29 (dd, *J* = 9.6, 2.6 Hz, 1H), 7.24 (dd, *J* = 5.5, 2.9 Hz, 2H), 7.14 (dd, *J* = 8.4, 5.6 Hz, 1H), 6.94 (td, *J* = 8.4, 2.7 Hz, 1H), 3.77 (s, 3H), 3.15 (d, *J* = 14.2 Hz, 1H), 2.83 (s, 3H), 2.11 (d, *J* = 14.1 Hz, 1H), 1.34 (s, 3H) ppm; **^{13}C NMR** (101 MHz, $CDCl_3$), δ = 173.6, 172.0, 162.8 (d, *J* = 247.6 Hz), 141.4, 140.7, 140.1 (d, *J* = 8.3 Hz), 134.0, 133.3 (d, *J* = 3.2 Hz), 129.9, 129.5, 129.3, 128.3 (d, *J* = 8.3 Hz), 118.0 (d, *J* = 22.3 Hz), 114.4 (d, *J* = 21.5 Hz), 56.9, 52.4, 40.9 (d, *J* = 1.6 Hz), 39.9, 22.3 ppm; **^{19}F NMR** (376 MHz, $CDCl_3$), δ = - 112.64 ppm; **FTIR (neat) ν/cm^{-1}** 2980.78, 2360.32, 1698.50, 1476.81, 1101.05, 828.54;

HRMS (ESI^+) $C_{19}H_{18}ClFNO_3$ $[M+H]^+$ requires 362.0954; found 362.0953;

m.p. = 156 - 157 °C;

α_D^{25} **=** − 104.7 (c = 0.5, $CHCl_3$);

Chiral HPLC: (Chiralpak IC, 50% $^{i-}$PrOH, 50% hexane, 0.70 mL min^{-1}, λ = 222 nm) τ_R (major) = 16.303 min, τ_R (minor) = 22.127 min.

Compound 31:

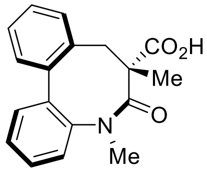

31

To the solution of **2** (31 mg, 0.1 mmol) in EtOH (5 mL) was added NaOH solid (8 mg, 0.2 mmol) at room temperature, the mixture was stirred for another 8 hours at 40 °C. Adjusted the pH of the mixture to 1~2, then extracted with EtOAc (10 mL x 2). The organic layer was dried over Na_2SO_4, filtered and concentrated in vacuo to give the pure compound **31** as a white solid (25 mg, 83% yield). **^{1}H NMR** (400 MHz, $CDCl_3$), δ = 7.53 – 7.38 (m, 2H), 7.31 (d, *J* = 2.1 Hz, 1H), 7.30 – 7.22 (m, 4H), 3.69 (d, *J* = 14.4 Hz, 1H), 2.94 (s, 3H), 2.19 (d, *J* = 14.4 Hz, 1H), 1.47 (s, 3H) ppm; **^{13}C NMR** (101 MHz, $CDCl_3$), δ = 176.9, 176.0, 141.7, 139.6, 137.7, 137.2, 130.0, 129.4, 129.1, 129.0, 128.7, 127.8, 126.7, 126.7, 53.1, 41.6, 40.6, 25.4 ppm; **FTIR (neat) ν/cm^{-1}** 2980.68, 2360.56, 1684.04, 1473.47, 1243.47, 1094.71, 773.07;
HRMS (ESI$^+$) $C_{18}H_{18}NO_3$ [M+H]$^+$ requires 296.1281; found 296.1281;
m.p. = 176 -178 °C;
$\boldsymbol{\alpha_D^{25}}$ = + 34.8 (c = 0.5, $CHCl_3$).

Compound 32:

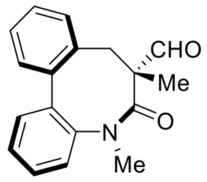

32

To the solution of **2** (31 mg, 0.1 mmol) in DCM (5 mL) was added DIBAL-H (0.15 mL, 0.15 mmol, 1.0 M in toluene) at -78 °C, at the same temperature the mixture was stirred overnight. Then, water (3 mL) was added to the mixture and stirred for 5 min at room temperature. Extracted with DCM (10 mL x 3), the organic layer was dried over Na_2SO_4, filtered and concentrated in vacuo followed by flash column chromatography purification to give the aldehyde **32** as a white solid (24 mg, 83.0 % yield, 99:1 dr, 96.0% ee). **^{1}H NMR** (400 MHz, $CDCl_3$), δ = 9.88 (s, 1H), 7.44 (td,

J = 7.5, 1.8 Hz, 1H), 7.39 (td, *J* = 7.5, 1.5 Hz, 1H), 7.31 (dd, *J* = 7.7, 1.3 Hz, 1H), 7.29 – 7.21 (m, 4H), 7.19 (s, 1H), 3.02 (d, *J* = 14.3 Hz, 1H), 2.87 (s, 3H), 2.07 (d, *J* = 14.2 Hz, 1H), 1.16 (s, 3H) ppm; **^{13}C NMR** (101 MHz, $CDCl_3$), δ = 198.1, 173.1, 141.4, 140.4, 138.6, 137.3, 129.8, 129.4, 129.3, 128.8, 128.5, 127.7, 127.4, 127.2, 56.4, 39.3, 37.7, 19.4 ppm;. **FTIR (neat) ν/cm^{-1}** 2932.78, 1734.24, 1628.82, 1446.50, 1126.88, 774.75;

HRMS (ESI$^+$) $C_{18}H_{18}NO_2$ $[M+H]^+$ requires 280.1332; found 280.1332

m.p. = 143 -145 °C.

α_D^{25} = − 91.8 (c = 0.5, $CHCl_3$)

Chiral HPLC: (Chiralpak IC, 50% $^{i-}$PrOH, 50% hexane, 1.00 mL min^{-1}, λ = 222 nm) τ_R (major) = 45.550 min, τ_R (minor) = 16.167 min.

Compound 34:

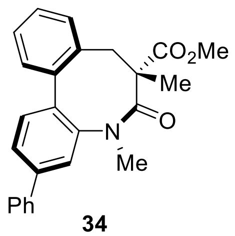

34

The compound **19** (18.0 mg, 0.05 mmol), CsF (22.0 mg, 0.15 mmol), Pd(OAc)2 (1.2 mg, 0.005 mmol), DavePhos (4.0 mg, 0.01 mmol) and $PhB(OH)_2$ (9.2 mg, 0.075 mmol) were added to a 10 mL reaction tubed with a magnetic stirrer bar under N_2. 2.0 mL of 1,4-dioxane was added and the reaction was stirred overnight at 30 °C. The mixture was concentrated in vacuo to remove the solvent and purified by flash column chromatography to give the coupling product **34** as a white solid (18 mg, 89.0% yield, 92:8 dr, 94.0% ee). **^{1}H NMR** (400 MHz, $CDCl_3$), δ = 7.70 – 7.62 (m, 4H), 7.58 (d, *J* = 1.7 Hz, 1H), 7.54 – 7.47 (m, 2H), 7.46 – 7.35 (m, 3H), 7.33 (dd, *J* = 7.3, 1.5 Hz, 1H), 7.30 (dd, *J* = 7.4, 1.8 Hz, 1H), 3.85 (s, 3H), 3.27 (d, *J* = 14.2 Hz, 1H), 2.98 (s, 3H), 2.28 (d, *J* = 14.1 Hz, 1H), 1.47 (s, 3H) ppm; **^{13}C NMR** (126 MHz, $CDCl_3$), δ = 174.1, 172.3, 142.5, 142.4, 139.6, 139.4, 138.4, 137.8, 130.8, 130.2, 129.0, 128.5, 128.0, 127.3, 127.0, 127.0, 126.9, 126.3, 57.1, 52.3, 41.0, 40.0, 22.6 ppm; **FTIR (neat) ν/cm^{-1}** 2947.27, 2363.05, 1738.52, 1644.96, 1450.06, 1207.53, 1098.19, 755.63;

HRMS (ESI$^+$) $C_{25}H_{24}NO_3$ $[M+H]^+$ requires 386.1751; found 386.1752;

m.p. = 170 -172 °C;

$\boldsymbol{\alpha}_{\mathbf{D}}^{\mathbf{25}}$ = − 60.0 (c = 0.5, $CHCl_3$);

Chiral HPLC: (Chiralpak IC, 50% ^{i}PrOH, 50% hexane, 1.00 mL min^{-1}, λ = 222 nm) τ_R (major) = 15.357 min, τ_R (minor) = 40.073 min.

5. Thermal Equilibration Studies

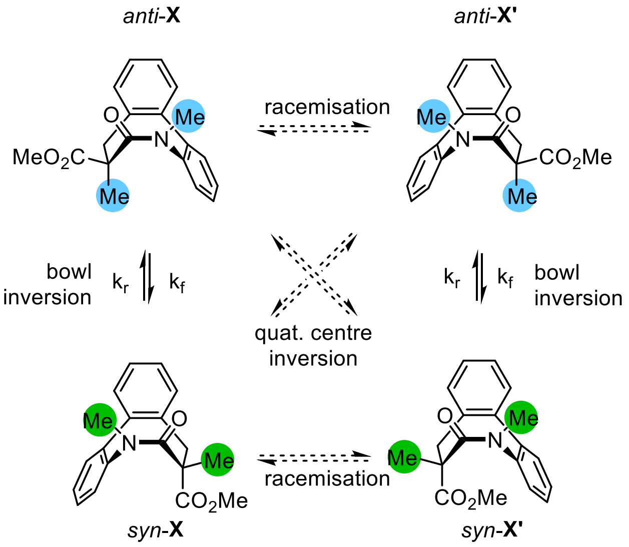

Figure S2. Stereochemical relationships between all isomers of cyclic products

- The relationship between of equilibrium constant and Gibbs energy (Eyring–Polanyi equation):

$$k = \frac{k_B T}{h} e^{\left(-\frac{\Delta G^{\ddagger}}{RT}\right)}$$

k_B is Boltzmann's constant, and h is Planck's constant. k_B = 1.380649×10^{-23} J· K^{-1}, h = 6.62607015×10^{-34} m^2 kg / s, R = 8.314 J·k^{-1}·mol^{-1}.

- Rearranging gives the following equations involving the Gibbs free energy:

$$\Delta G^{\ddagger}\ (\mathrm{J\cdot mol^{-1}}) = \ln\left(\frac{k_B T}{kh}\right) RT$$

- Enantiomerically-enriched *anti*-**2** was dissolved in *m*-xylene (10 mg/mL) and stirred at 90 °C (363 K). At the given intervals of time, about 5 μL of the solution was injected to the HPLC and the enantiomeric ratio was measured by using peak integrations.

Table S2. Thermal isomerization of *anti*-2 and *syn*-2 at 363K

entry	Time (mins)	*anti*-**2**	*syn*-**2**	d.r.(anti/syn)	ee(A)	ee(B)
1	0	98	2	98/2	98.6	-12.5
2	30	63	37	63/37	95.6	89.9
3	60	44	56	44/56	95.7	91.4
4	90	37	63	37/63	94.3	92.3

5	120	29	71	29/61	94.5	92.3
6	150	22	78	22/78	93.7	92.7
7	180	18	82	18/82	94.3	92.9
8	210	17	83	17/83	93.6	92.9
9	240	16	84	16/84	93.6	92.9
10	270	15	85	15/85	92.9	93
11	300	15	85	15/85	93.1	93

- To calculate the kinetic constant (k), utilizing the data in the Table S2, plotting the value of $\ln(\frac{R-Req}{R+1})$ against time (in sec), here R represents the diastereomeric ratio; Req represents the diastereomeric ratio at equilibrium. A linear equation was obtained with the slope equal to the negative of the rate constant (k):

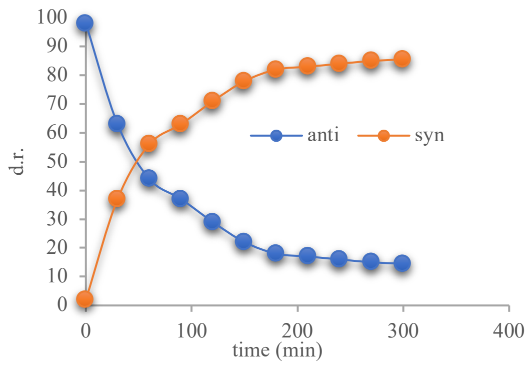

d.r. versus time

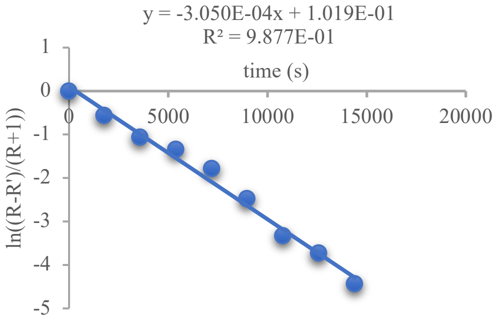

Equilibration of anti and syn versus time

Figure S3. Thermal isomerization of *anti*-**2** and *syn*-**2**

In this equilibrium, the k is the sum of k_r and k_f,, and the ratio between the values of k_f and k_r, is the inversely proportional to the equilibrium concentration of anti and syn isomers, therefore , we have:

$$\text{Slope} = -k;$$

$$k_r / k_f = 15/85;$$

$$k = -\text{slope} = k_r + k_f.$$

Then was given:

$$k_r = 2.287\text{x}10^{-5};\ \Delta G^{\ddagger} = 31.02\ \text{Kcal/mol}$$

$$k_f = 1.296\text{x}10^{-4};\ \Delta G^{\ddagger} = 29.66 \text{ Kcal/mol}$$

- The change in e.e of *anti*-**2** and *syn*-**2** in this process is graphically represented below:

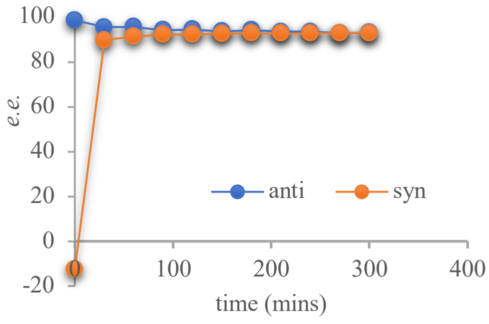

e.e.s of *anti*-**2** and *syn*-**2** versus time

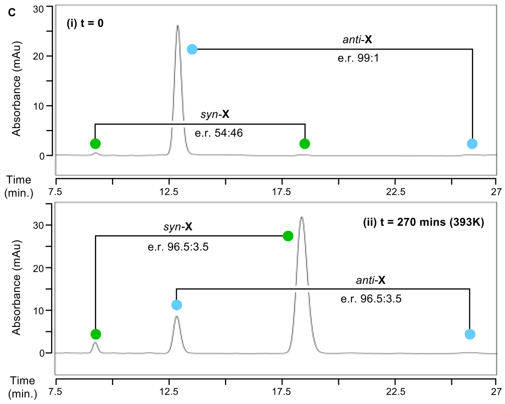

Figure S4. Analytical chiral HPLC traces of the cyclization product of *anti*-**2** and *syn*-**2**

6. Absolute configuration assignment of *anti*-2.

The absolute configuration of *anti*-**2** (ee > 99%; CCDC 2071683) shown below was unambiguously determined by X-ray crystallographic analysis. The intensity data was collected on an Oxford SuperNova diffractometer using graphite-monochromated Cu Kα radiation.

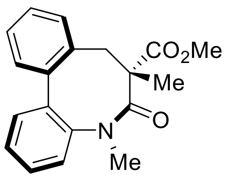

anti-**2**

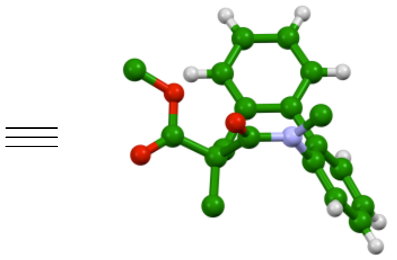

Table S3. Crystal data and structure refinement for compound *anti*-2.

Identification code	*anti*-**2**
Empirical formula	$C_{19}H_{19}NO_3$
Formula weight	309.36
Temperature/K	150
Crystal system	N/A
Space group	$P2_1$
a/Å	9.28110(10)
b/Å	6.20260(10)
c/Å	13.7987(2)
α/°	90
β/°	99.2193(12)
γ/°	90
Volume/Å^3	784.088(19)
Z	2
ρ_{calc}g/cm^3	1.310
μ/mm^{-1}	0.716
F(000)	328.0
Crystal size/mm^3	0.150 × 0.130 × 0.100
Radiation	Cu Kα (λ = 1.54184)
2Θ range for data collection/°	9.654 to 152.564
Index ranges	$-11 \leq h \leq 11$, $-7 \leq k \leq 7$, $-17 \leq l \leq 14$
Reflections collected	16962
Independent reflections	3248 [R_{int} = 0.035, R_{sigma} = 0.038]
Data/restraints/parameters	3248/1/209
Goodness-of-fit on F^2	1.003
Final R indexes [I>=2σ (I)]	R_1 = 0.0289, wR_2 = 0.0757
Final R indexes [all data]	R_1 = 0.0294, wR_2 = 0.0763
Largest diff. peak/hole / e Å^{-3}	0.16/-0.18
Flack parameter	-0.02(6)

7. Relative Configuration assignment of *syn*-2.

The relative configuration of *syn*-**2** (CCDC 2071682) shown below was unambiguously determined by X-ray crystallographic analysis. The intensity data was collected on an Oxford SuperNova diffractometer using graphite-monochromated Cu Kα radiation.

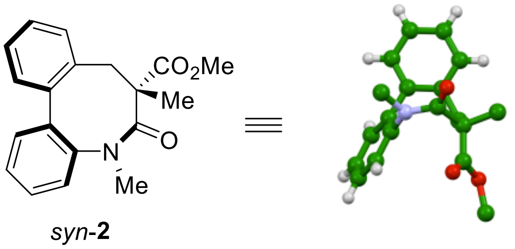

syn-**2**

Table S4. Crystal data and structure refinement for *syn*-2.

Identification code	*syn*-**2**
Empirical formula	$C_{19}H_{19}NO_3$
Formula weight	309.36
Temperature/K	150
Crystal system	N/A
Space group	$P2_1/c$
a/Å	9.8741(2)
b/Å	10.9069(3)
c/Å	14.6377(4)
α/°	90
β/°	91.379(2)
γ/°	90
Volume/Å^3	1575.96(7)
Z	4
ρ_{calc}g/cm^3	1.304
μ/mm^{-1}	0.712
F(000)	656.0
Crystal size/mm^3	0.180 × 0.150 × 0.060
Radiation	Cu Kα (λ = 1.54180)
2Θ range for data collection/°	8.958 to 152.792
Index ranges	$-6 \leq h \leq 12$, $-13 \leq k \leq 13$, $-18 \leq l \leq 17$
Reflections collected	8182
Independent reflections	3277 [R_{int} = 0.028, R_{sigma} = 0.038]
Data/restraints/parameters	3277/0/208
Goodness-of-fit on F^2	1.006
Final R indexes [I>=2σ (I)]	R_1 = 0.0410, wR_2 = 0.1137
Final R indexes [all data]	R_1 = 0.0494, wR_2 = 0.1224
Largest diff. peak/hole / e Å^{-3}	0.28/-0.27

8. NMR studies of rotational barriers of 28 and 30

Exchange dynamics were investigated using 1D and 2D 19F exchange (EXSY) experiments on a Bruker AVIII HD 500 MHz spectrometer equipped with a BBFO SMART probe operating under TOPSPIN 3.6.3. Experiments recorded below ambient probe temperature (~ 298 K) utilized a nitrogen exchange unit for cooling. Probe temperature calibrations were performed using either methanol-d4 (below or at ambient) or neat ethylene glycol (above ambient).

Exchange in reference compound 28

The ^{19}F NMR spectrum at 298K of compound **28** (in d_8-toluene) displayed a total of four resonances, two of which represented conformers of very low population (Fig. Sx1). NOE studies performed in $CDCl_3$ at 253 K (where no exchange between peaks was observed in ^{1}H NOESY spectra) indicated the two major resonances corresponded to conformers in which the amide C=O group was *anti-* to the aryl ring. The two far smaller species are believed therefore to arise from conformers in which the carbonyl exists *syn-* to the aryl. At 298K, exchange between the two minor forms arising from *N*-aryl rotation is readily seen whereas exchange between the two major and two minor forms due to amide *anti-syn* inversion could be only observed in ^{19}F EXSY 2D spectra with mixing times > 200 ms (Fig. Sx2). Under these conditions, the *N*-aryl exchange between the two major forms could not be detected.

Exchange rates between the two dominant resonances were therefore investigated as a function of temperature using ^{19}F 1D selective magnetization transfer (1D EXSY) experiments. These were performed using a d1-90°-τ-90°-acq pulse sequence with $\tau = 1/(2*\delta\Delta)$, where δΔ represents frequency separation in hz between the exchanging peaks. This leads to the on-resonance peak being inverted with the off-resonance exchanging peak left unperturbed. The mixing delay τ allows for magnetization exchange between resonances leading to intensity variation in the off-resonance peak as the inverted peak recovers to equilibrium (Fig Sx3); τ was varied between 5 ms to 5 s. The recovery delay d1 was set to 4 s and 16 transients acquired per spectrum at temperatures of 298, 323, 333, 343 and 353 K that were calibrated with ethylene glycol. Data fitting to yield exchange rate constants was performed using the CIFIT program (REF) and rates plotted as ln(k/T) vs 1/T to yield thermodynamic parameters (Eyring plot) (Fig Sx4). These results yielded $\Delta G^{\ddagger}_{298}$ = 79.6 kJ mol^{-1} for the *N*-aryl rotation barrier. The 2D EXSY study in $CDCl_3$ indicated the exchange barrier for interconversion between the two minor species was significantly reduced to ~ 67 kJ mol^{-1}. The large difference in barriers can be correlated with the greater steric hindrance occurring in the dominant forms in which the amide C=O adopts the *anti*-conformation relative to the aryl ring.

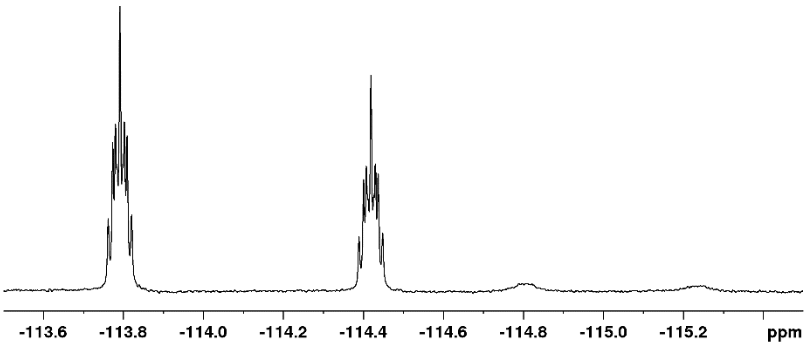

Figure S5: ^{19}F (^{1}H-coupled) NMR spectrum of **28** in d_8-toluene, 298K.

a)

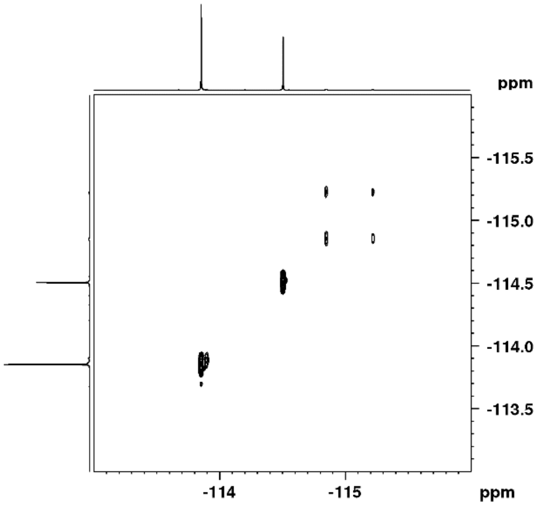

b)

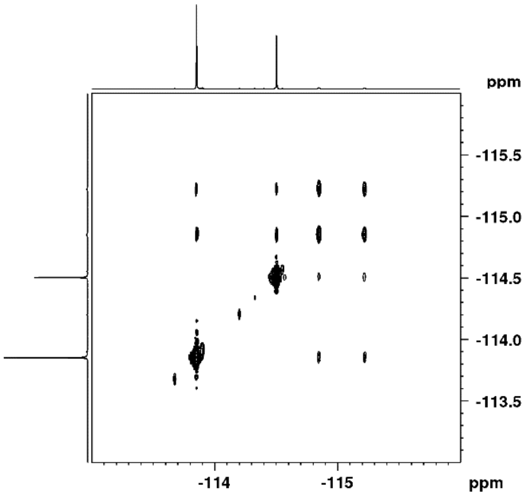

Figure S6: ^{19}F EXSY spectra of **28** in $CDCl_3$, 298K. a) 2D EXSY 200 ms mixing, b) 2D EXSY 1 s mixing. Projections show the ^{1}H-decoupled ^{19}F spectrum. In (a) only the exchange arising from aryl inversion in the two minor *syn*-CO forms is observed whilst in (b) the *anti*-*syn* amide inversion becomes apparent whilst the *N*-aryl inversion of the two major *anti*-CO forms is still not evident.

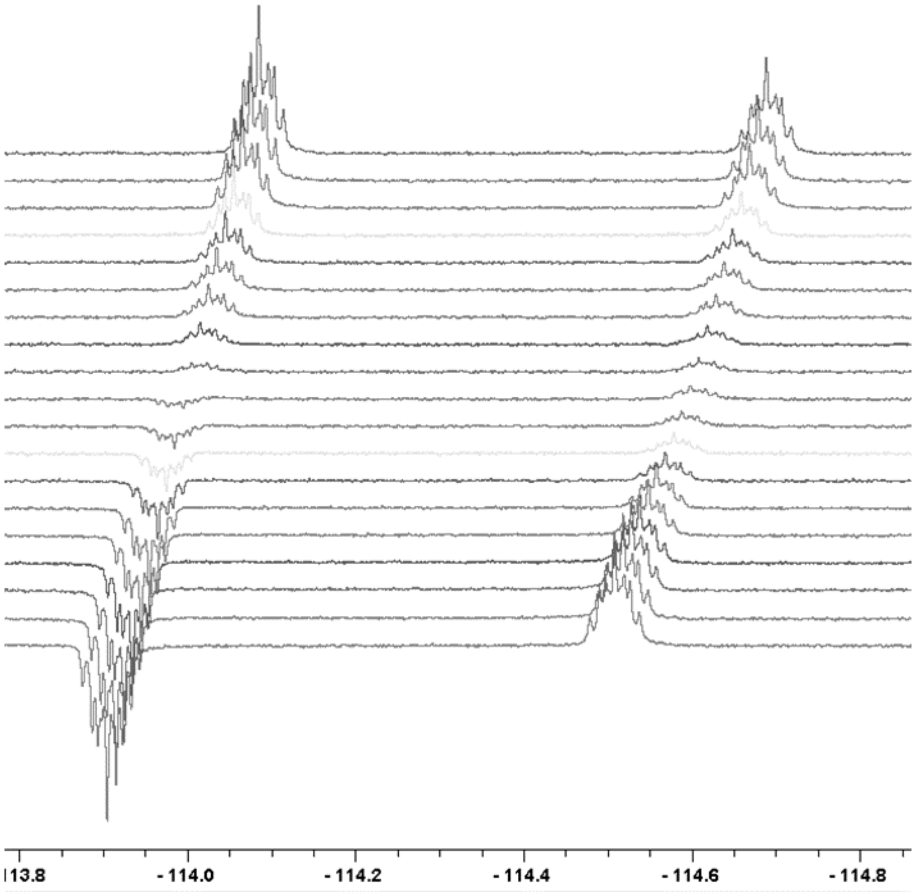

Figure S7: ^{19}F 1D EXSY spectra (333 K, d_8-toluene) of **28** with mixing times ranging from 5 ms to 5 s and on-resonance inversion at -113.9 ppm

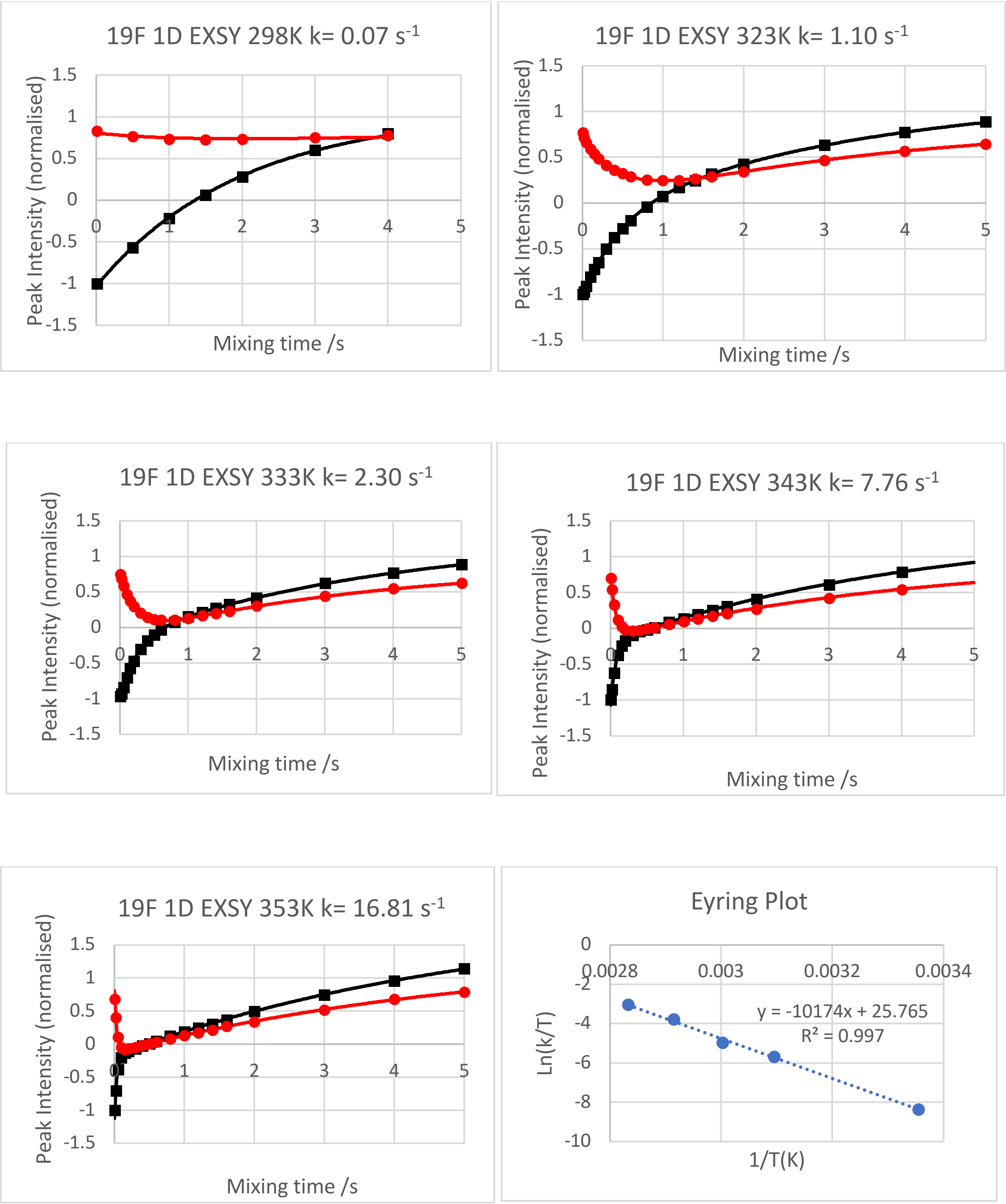

Figure S8: 19F 1D EXSY data of **28** fitted with the CIFIT routine to yield rate constants and the resulting Eyring plot. Solid data points represent experimental peak intensities (integrals) with solids lines derived from CIFIT fitting.

Exchange in compound Y (30)

Compound **30** exhibited 8 distinct resonances in its ^{19}F spectrum at 253 K in CDCl3 (500 MHz ^{1}H) corresponding to three hindered rotational processes (aryl-N, aryl-aryl, and N(Me)-CO). Exchange dynamics were investigated over the temperature range 253-303 K using 2D ^{19}F EXSY experiments (Fig Sy1) with mixing times τ ranging from 50 to 800 ms. In addition, "zero mixing time" (τ = 20 μs) experiments were recorded to provide reference data for the extraction of exchange rates. At temperatures below 253 K, exchange cross peaks became too weak to detect whilst above 303 K peaks became broad and those of lower intensity were lost in the baseline. Within this temperature range, only a single hindered exchange process was observed to give rise to exchange cross peaks of significant intensity (Fig Sy1). Analysis of the 2D EXSY data to extract rate constants employed the EXSYCalc software from MestreLabs (www.mestrelab.com) which requires intensity data (as 2D volume integrals) of diagonal and cross peaks from two data sets: 1) reference EXSY spectrum with zero mixing time (diagonal peaks only) and 2) EXSY spectrum with exchange peaks present (diagonal and cross peak intensities). 2D volume integration was performed using Bruker Topspin v4.0.8. The thermodynamic parameters $\Delta H^{\ddagger}$ and $\Delta S^{\ddagger}$ were determined from the Eyring plot (Fig Sy2).

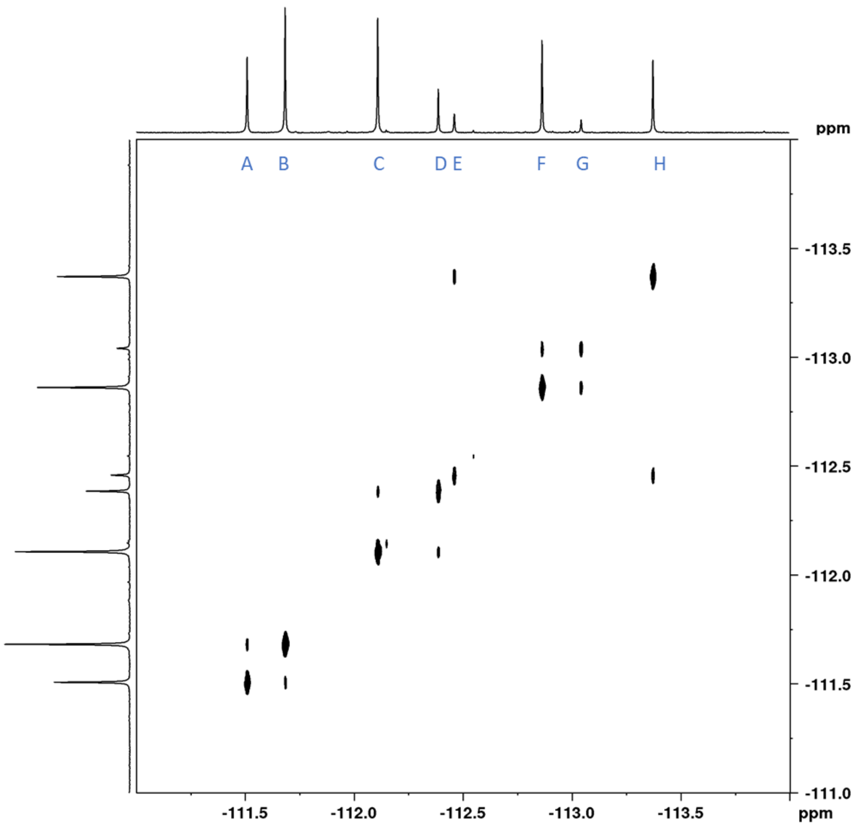

Figure S9: ^{19}F 2D EXSY spectrum of **30** at 253 K ($CDCl_3$) with mixing time 800 ms.

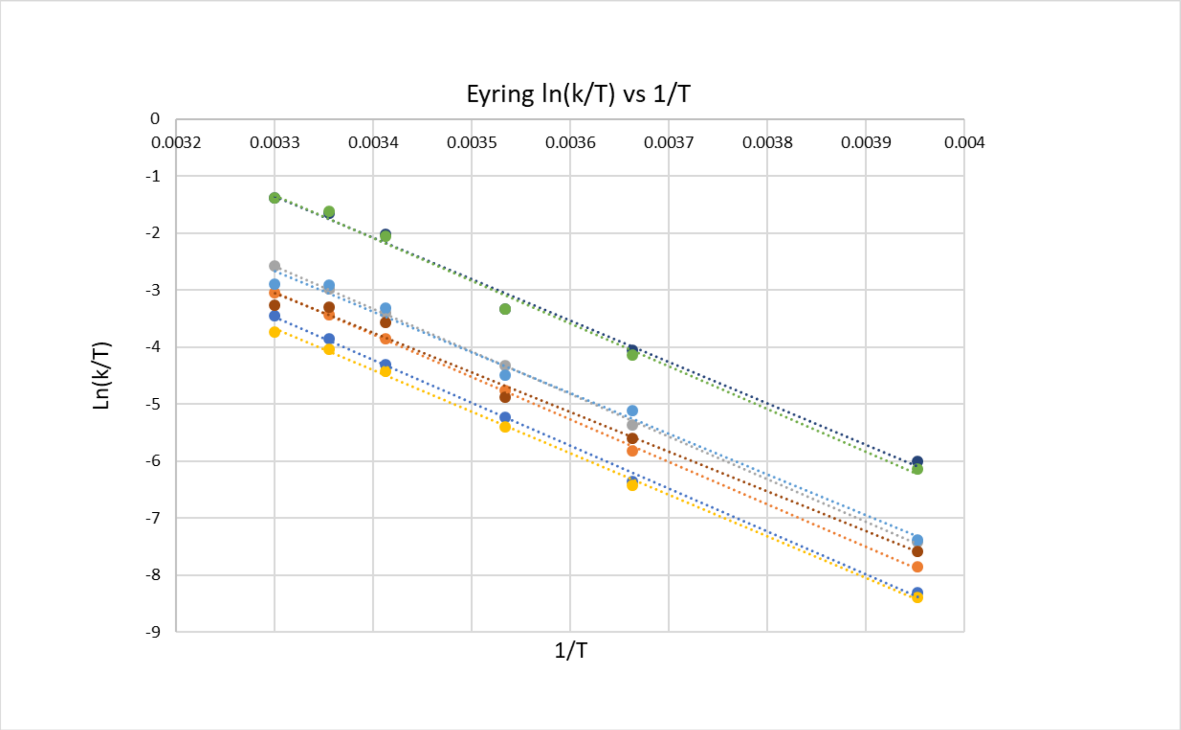

Figure S10: Eyring plot derived from 2D EXSY rate constants for **30** (253-303 K).

Exchange peaks	ΔH‡ kJ/mol	ΔS‡ J/K/mol	$\Delta G^{\ddagger}_{298}$ kJ/mol
BA	62.6	-19.7	68.5
AB	61.9	-18.6	67.4
DC	62.2	-13.6	66.3
CD	60.6	-28.1	69.0
HE	59.3	-23.8	66.4
EH	62.4	-2.6	63.2
GF	60.5	-9.2	63.2
FG	57.9	-31.8	67.4

Table S5: Thermodynamic parameters determined from the Eyring plot of 2D ^{19}F EXSY data for the exchange barriers in **30**.

1D ^{19}F experiments were also recorded over the range 298-358 K in d_8-toluene, and revealed a complex pattern of (partial) peak coalescences, although complete coalescence of all peaks was not observed and it was not possible to determine exchange partners at the higher temperatures (Fig Sy3). As such, lineshape fitting was not undertaken for these data, although estimates derived from peak separations suggested the

energy barriers to be considerably in excess of 71 kJ mol^{-1}, consistent with the data from reference compound **28** above.

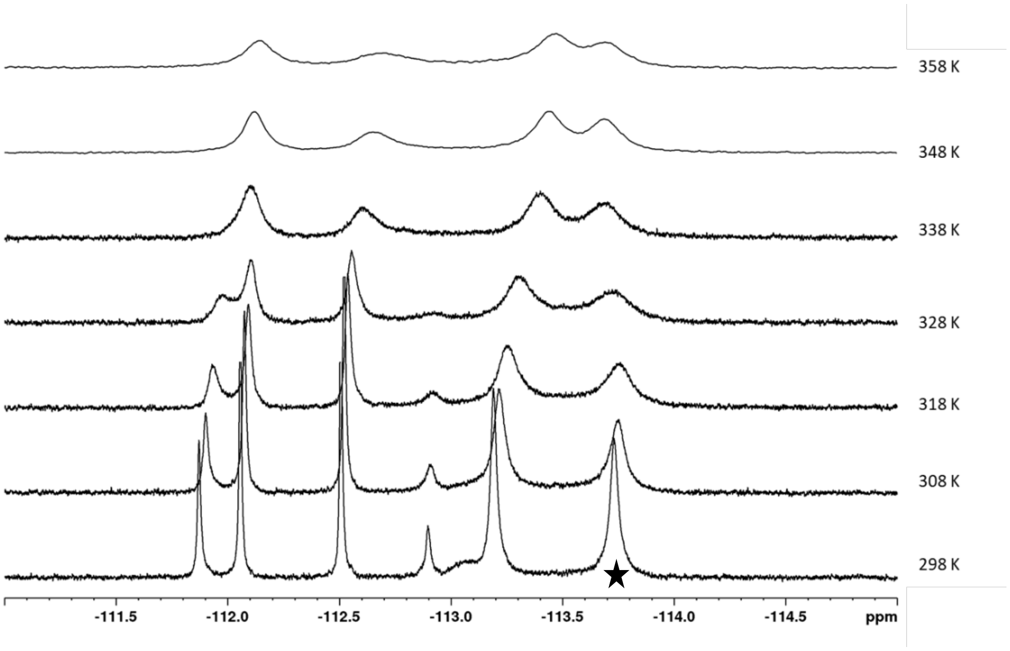

Figure S11: ^{19}F spectra of **30** in d_8-toluene as a function of temperature from 298-358 K.

The star indicates the location of two overlapped resonances.

NMR of enolate 29

The interconversion of the enolate **29** of compound **28** in d_8-toluene/d_8-THF (1:3) was studied by ^{19}F NMR at low temperature. Although line broadening was observed, no resonance decoalesence was seen at temperatures down to 223 K.

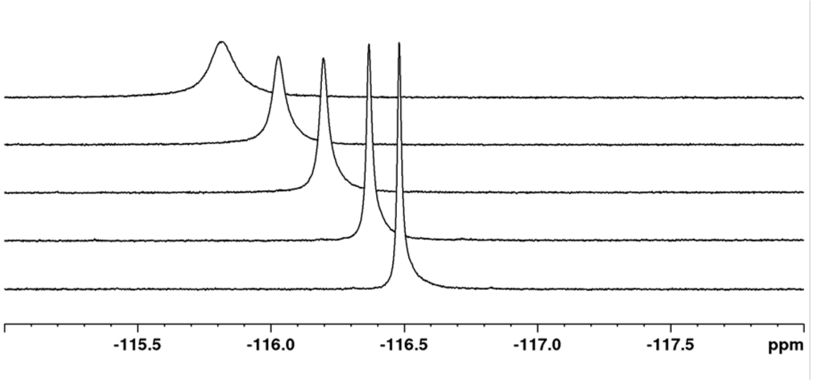

Figure S11: ^{19}F NMR spectra of enolate **29** in d_8-toluene/d_8-THF (1:3) as a function of temperature from 298-223 K.

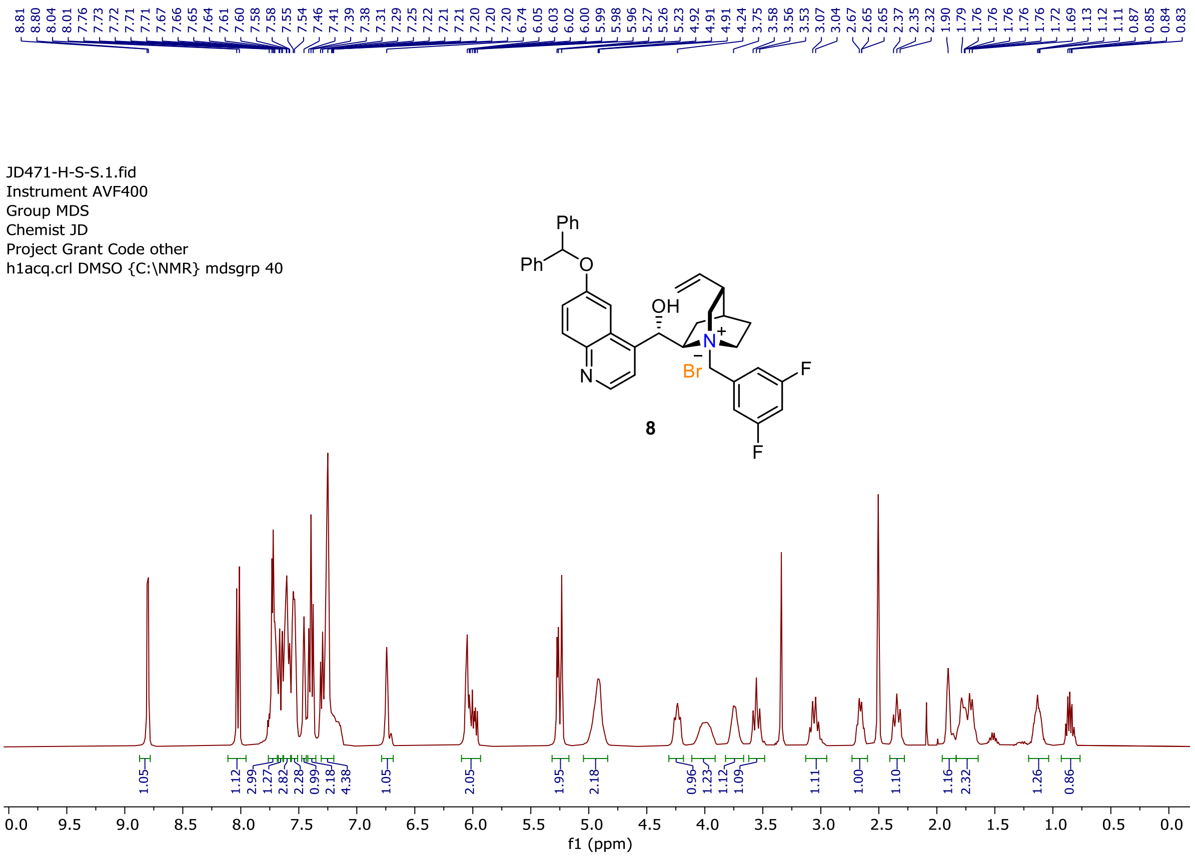
8.81 8.80 8.04 8.01 7.76 7.73 7.72 7.71 7.71 7.67 7.66 7.65 7.64 7.61 7.60 7.58 7.58 7.55 7.54 7.46 7.41 7.39 7.38 7.31 7.29 7.25 7.22 7.21 7.21 7.20 7.20 7.20 6.74 6.05 6.03 6.02 6.00 5.99 5.98 5.96 5.27 5.26 5.23 4.92 4.91 4.91 4.24 3.75 3.58 3.56 3.53 3.07 3.04 2.67 2.65 2.65 2.37 2.35 2.32 1.90 1.79 1.76 1.76 1.76 1.76 1.72 1.69 1.13 1.12 1.11 0.87 0.85 0.84 0.83
JD471-H-S-S.1.fid
Instrument AVF400
Group MDS
Chemist JD
Project Grant Code other
h1acq.crl DMSO {C:\NMR} mdsgrp 40
Ph
Ph
O
OH
N
N
+
−
Br
F
F
8
1.05 1.12 2.99 1.27 2.82 2.28 0.99 2.18 4.38 1.05 2.05 1.95 2.18 0.96 1.23 1.12 1.09 1.11 1.00 1.10 1.16 2.32 1.26 0.86
0.0 9.5 9.0 8.5 8.0 7.5 7.0 6.5 6.0 5.5 5.0 4.5 4.0 3.5 3.0 2.5 2.0 1.5 1.0 0.5 0.0
f1 (ppm)

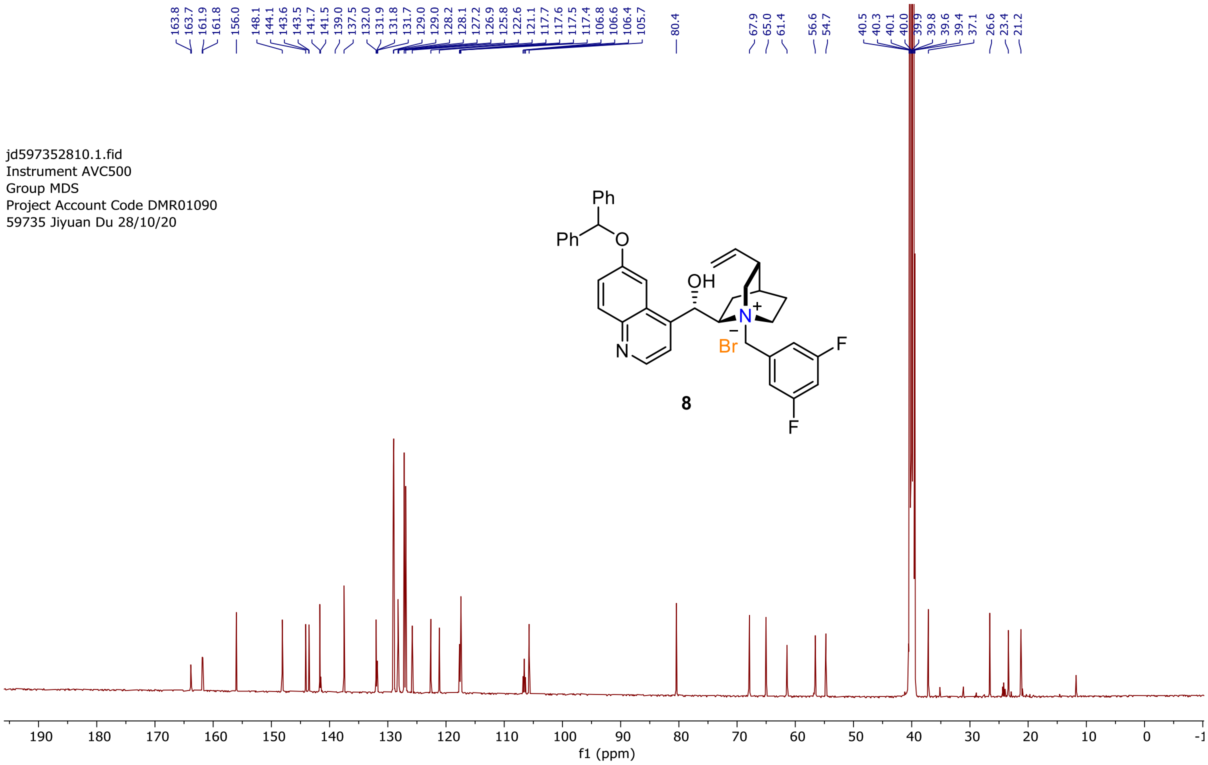
163.8 163.7 161.9 161.8 156.0 148.1 144.1 143.6 143.5 141.7 141.5 139.0 137.5 132.0 131.9 131.8 131.7 129.0 128.2 128.1 127.2 126.9 125.8 122.6 121.1 117.7 117.6 117.5 117.4 106.8 106.6 106.4 105.7 80.4 67.9 65.0 61.4 56.6 54.7 40.5 40.3 40.1 40.0 39.9 39.8 39.6 39.4 37.1 26.6 23.4 21.2
jd597352810.1.fid
Instrument AVC500
Group MDS
Project Account Code DMR01090
59735 Jiyuan Du 28/10/20
Ph
Ph
O
OH
N
N
+
−
Br
F
F
8
190 180 170 160 150 140 130 120 110 100 90 80 70 60 50 40 30 20 10 0
f1 (ppm)

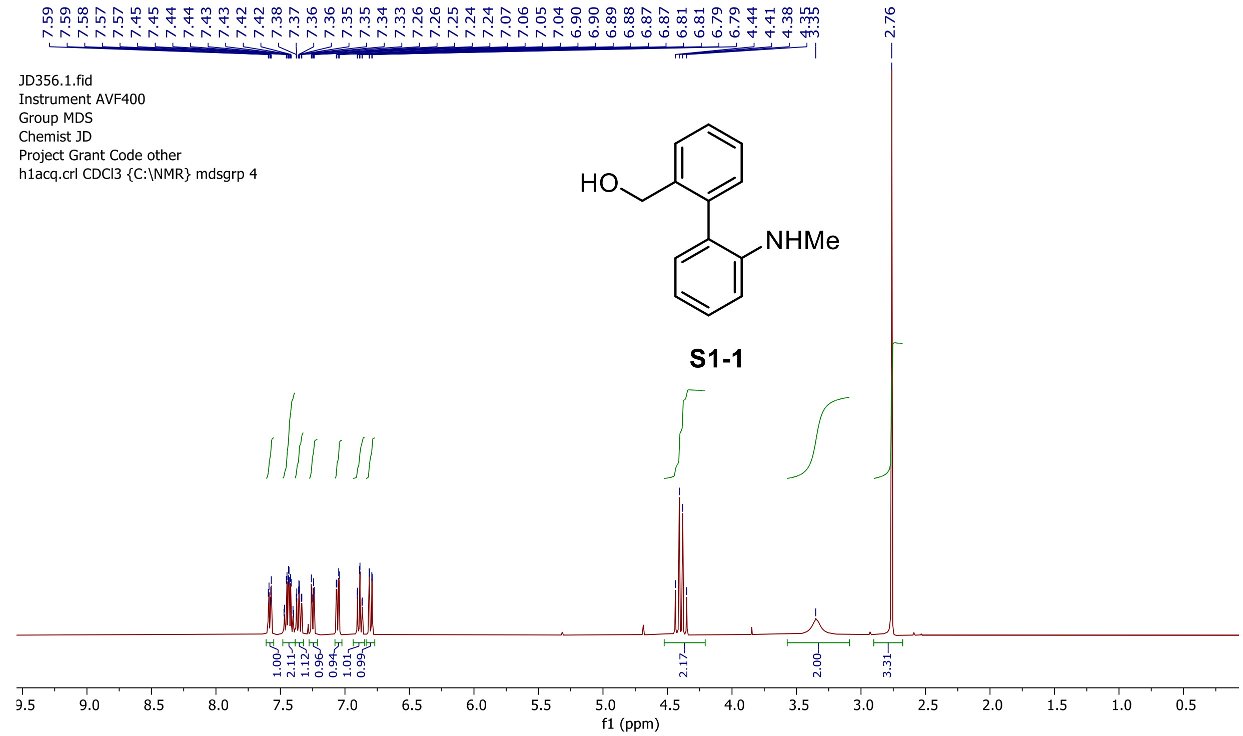
7.59 7.59 7.58 7.57 7.57 7.45 7.44 7.44 7.43 7.43 7.42 7.42 7.38 7.37 7.36 7.36 7.35 7.35 7.34 7.33 7.26 7.26 7.25 7.24 7.24 7.07 7.06 7.05 7.04 6.90 6.90 6.89 6.88 6.87 6.87 6.81 6.81 6.79 6.79 4.44 4.41 4.38 4.35 3.35
2.76
JD356.1.fid
Instrument AVF400
Group MDS
Chemist JD
Project Grant Code other
h1acq.crl CDCl3 {C:\NMR} mdsgrp 4
HO
NHMe
S1-1
1.00 2.11 1.12 0.96 0.94 1.01 0.99
2.17
2.00
3.31
f1 (ppm)

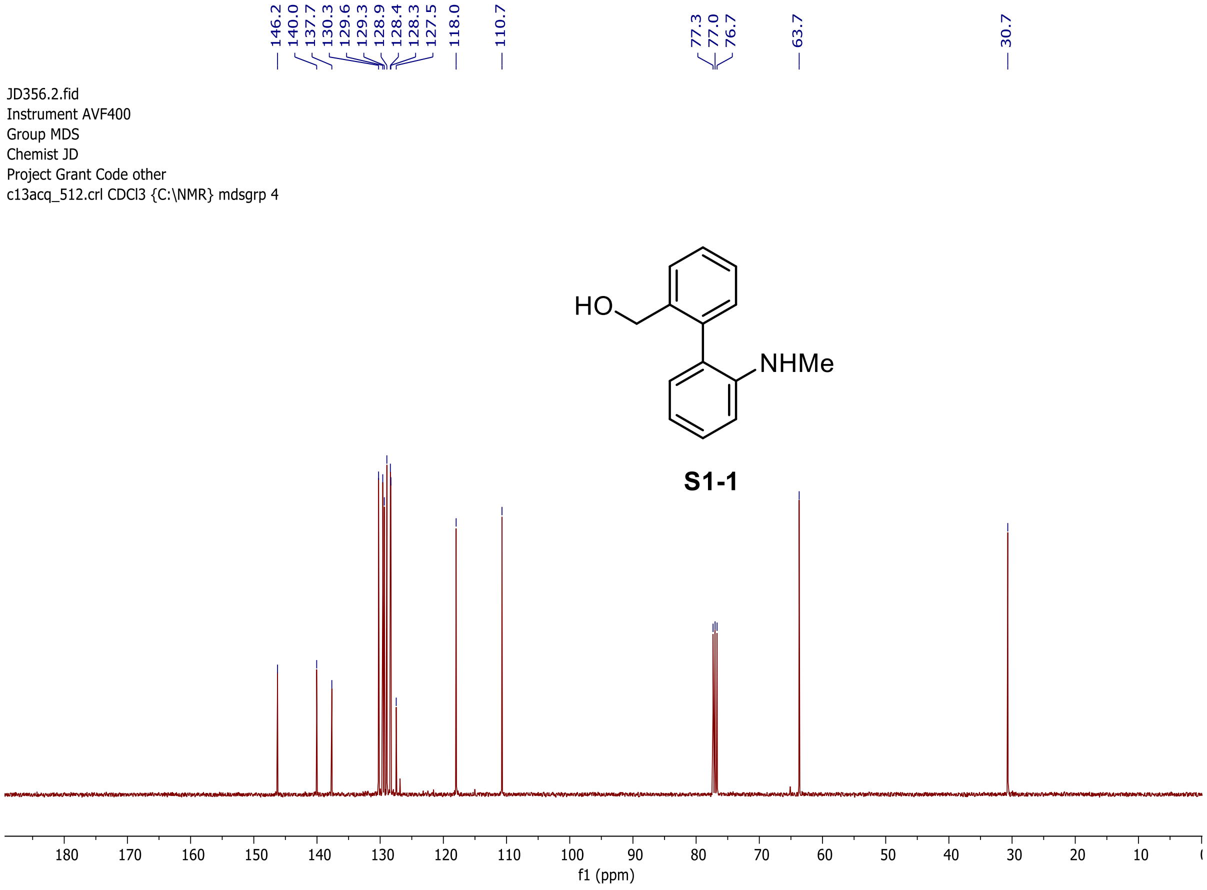
146.2 140.0 137.7 130.3 129.6 129.3 128.9 128.4 128.3 127.5 118.0 110.7
77.3 77.0 76.7
63.7
30.7
JD356.2.fid
Instrument AVF400
Group MDS
Chemist JD
Project Grant Code other
c13acq_512.crl CDCl3 {C:\NMR} mdsgrp 4
HO
NHMe
S1-1
f1 (ppm)

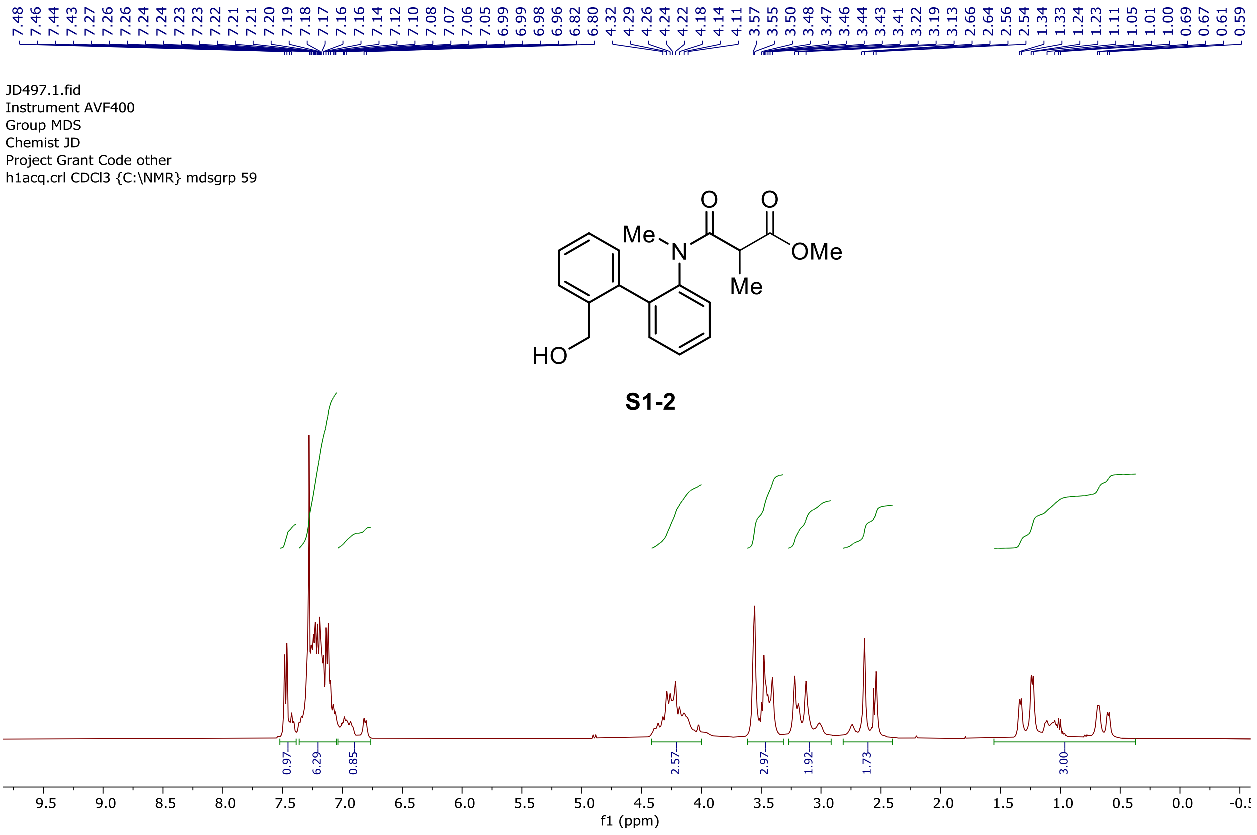
7.48 7.46 7.44 7.43 7.27 7.26 7.26 7.24 7.24 7.23 7.23 7.22 7.21 7.21 7.20 7.19 7.18 7.17 7.16 7.16 7.14 7.12 7.10 7.08 7.07 7.06 7.05 6.99 6.99 6.98 6.96 6.82 6.80 4.32 4.29 4.26 4.24 4.22 4.18 4.14 4.11 3.57 3.55 3.50 3.48 3.47 3.46 3.44 3.43 3.41 3.22 3.19 3.13 2.66 2.64 2.56 2.54 1.34 1.33 1.24 1.23 1.11 1.05 1.01 1.00 0.69 0.67 0.61 0.59
JD497.1.fid
Instrument AVF400
Group MDS
Chemist JD
Project Grant Code other
h1acq.crl CDCl3 {C:\NMR} mdsgrp 59
Me
N
O
O
OMe
Me
HO
S1-2
0.97
6.29
0.85
2.57
2.97
1.92
1.73
3.00
f1 (ppm)

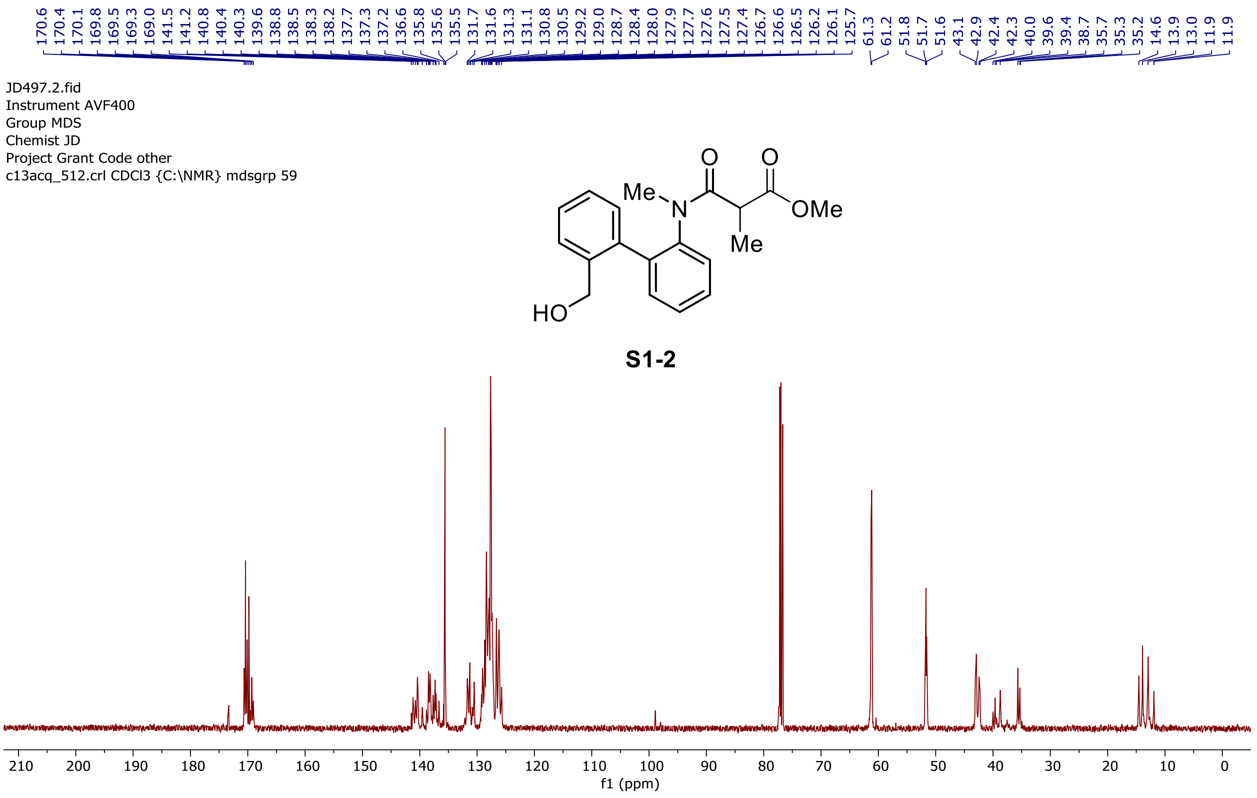
170.6 170.4 170.1 169.8 169.5 169.3 169.0 141.5 141.2 140.8 140.4 140.3 139.6 138.8 138.5 138.3 138.2 137.7 137.3 137.2 136.6 135.8 135.6 135.5 131.7 131.6 131.3 131.1 130.8 130.5 129.2 129.0 128.7 128.4 128.0 127.9 127.7 127.6 127.5 127.4 126.7 126.6 126.5 126.2 126.1 125.7 61.3 61.2 51.8 51.7 51.6 43.1 42.9 42.4 42.3 40.0 39.6 39.4 38.7 35.7 35.3 35.2 14.6 13.9 13.0 11.9 11.9
JD497.2.fid
Instrument AVF400
Group MDS
Chemist JD
Project Grant Code other
c13acq_512.crl CDCl3 {C:\NMR} mdsgrp 59
Me
N
O
O
OMe
Me
HO
S1-2
f1 (ppm)

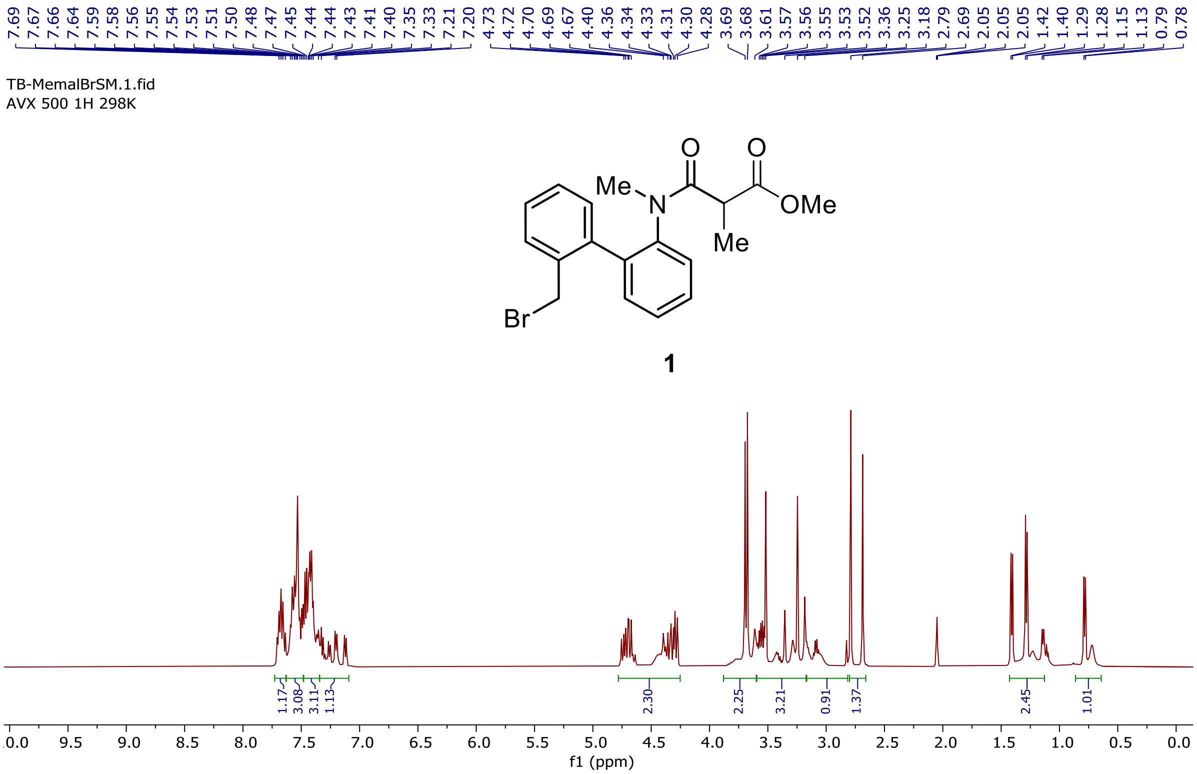
7.69 7.67 7.66 7.64 7.59 7.58 7.56 7.55 7.54 7.53 7.51 7.50 7.48 7.47 7.45 7.44 7.44 7.43 7.41 7.40 7.35 7.33 7.21 7.20 4.73 4.72 4.70 4.69 4.67 4.40 4.36 4.34 4.33 4.31 4.30 4.28 3.69 3.68 3.61 3.57 3.56 3.55 3.53 3.52 3.36 3.25 3.18 2.79 2.69 2.05 2.05 2.05 1.42 1.40 1.29 1.28 1.15 1.13 0.79 0.78
TB-MemalBrSM.1.fid
AVX 500 1H 298K
Me
N
O
O
OMe
Me
Br
1
1.17 3.08 3.11 1.13 2.30 2.25 3.21 0.91 1.37 2.45 1.01
f1 (ppm)

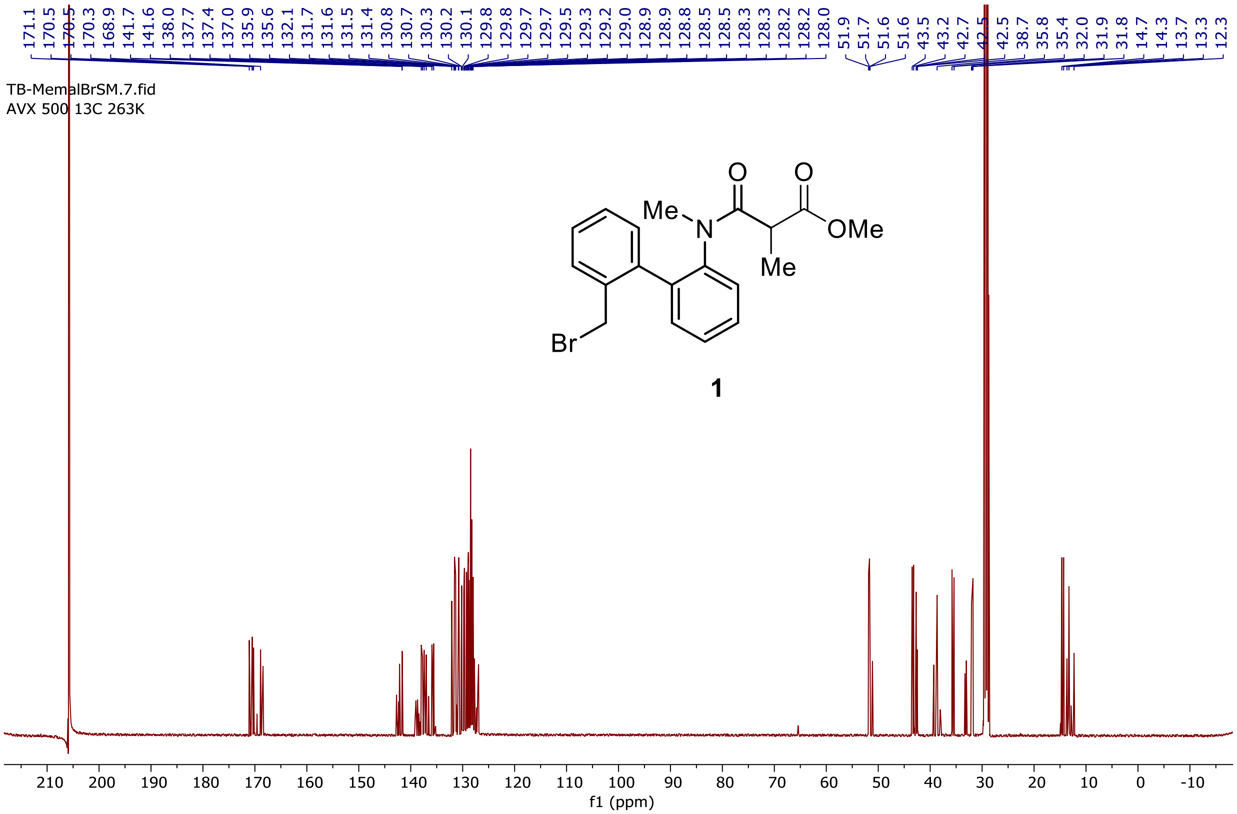
171.1 170.5 170.5 170.3 168.9 141.7 141.6 138.0 137.7 137.4 137.0 135.9 135.6 132.1 131.7 131.6 131.5 131.4 130.8 130.7 130.3 130.2 130.1 129.8 129.8 129.7 129.7 129.5 129.3 129.2 129.0 128.9 128.9 128.8 128.5 128.5 128.3 128.3 128.2 128.2 128.0 51.9 51.7 51.6 51.6 43.5 43.2 42.7 42.5 42.5 38.7 35.8 35.4 32.0 31.9 31.8 14.7 14.3 13.7 13.3 12.3
TB-MemalBrSM.7.fid
AVX 500 13C 263K
Me
N
O
O
OMe
Me
Br
1
f1 (ppm)

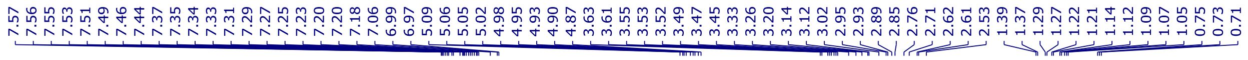

JD505.1.fid
Instrument AVH400
Group MDS
Chemist JD
Project Account Code other
h1acq.crl CDCl3 {C:\NMR} mdsgrp 3

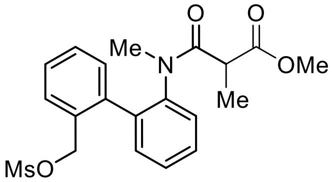

5

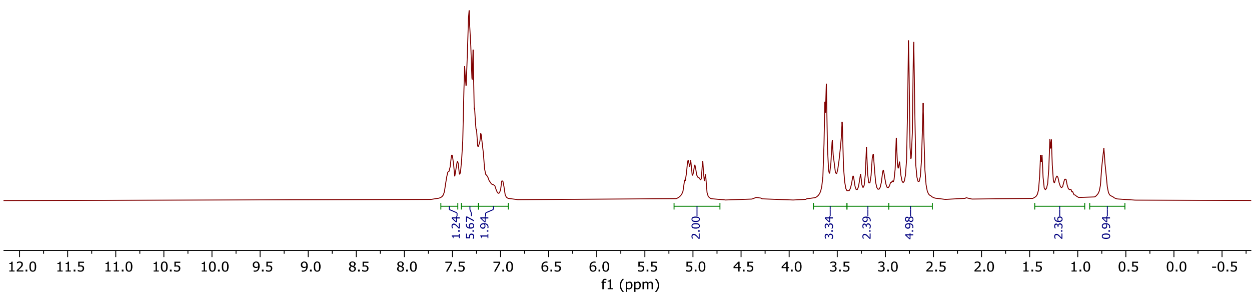

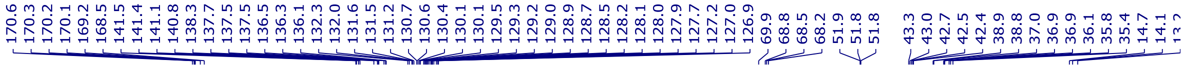

JD505.2.fid
Instrument AVH400
Group MDS
Chemist JD
Project Account Code other
c13acq_512.crl CDCl3 {C:\NMR} mdsgrp 3

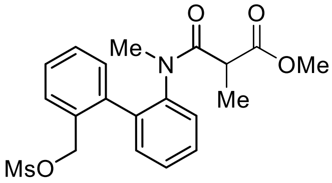

5

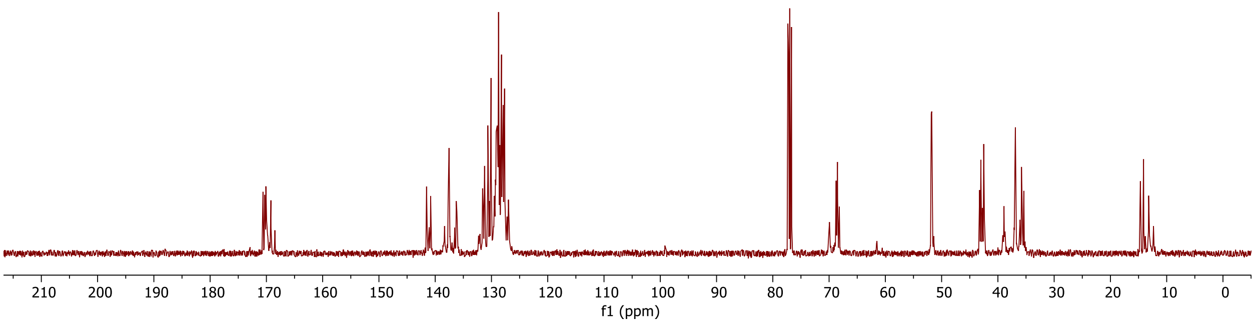

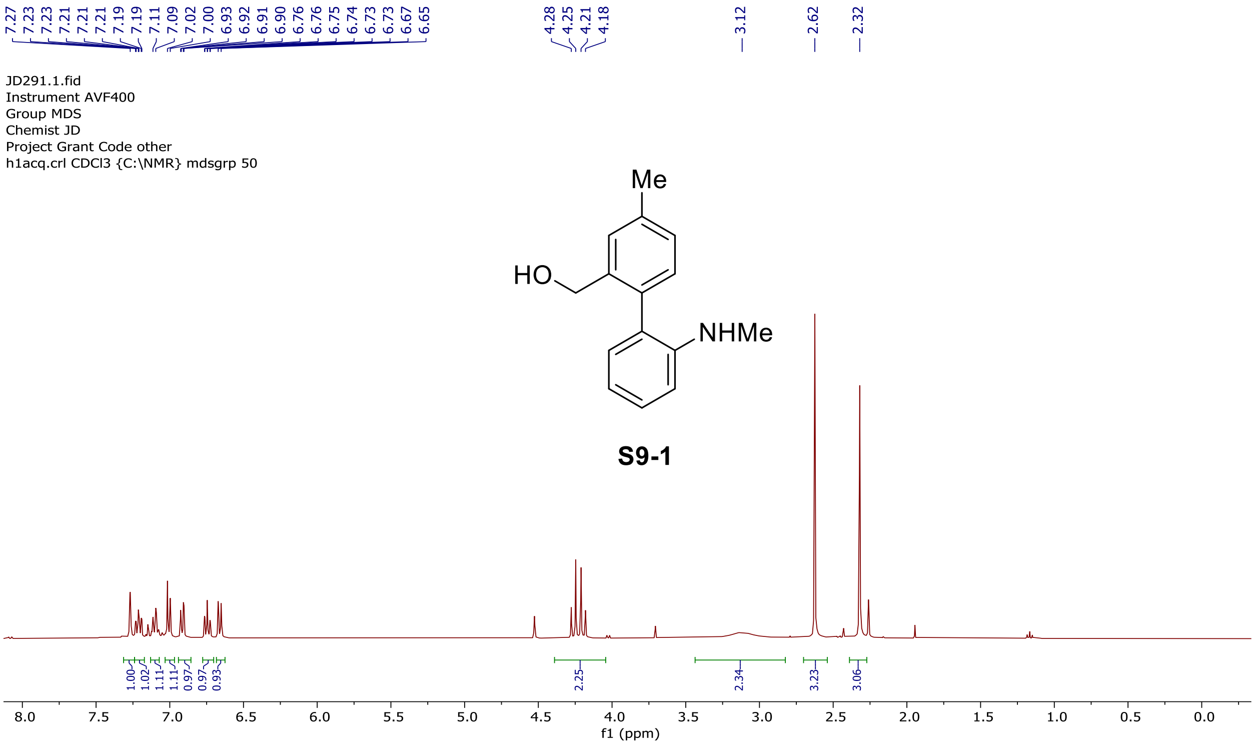
7.27 7.23 7.23 7.21 7.21 7.21 7.19 7.19 7.11 7.09 7.02 7.00 6.93 6.92 6.91 6.90 6.76 6.76 6.75 6.74 6.73 6.73 6.67 6.65
4.28 4.25 4.21 4.18
3.12
2.62
2.32
JD291.1.fid
Instrument AVF400
Group MDS
Chemist JD
Project Grant Code other
h1acq.crl CDCl3 {C:\NMR} mdsgrp 50
Me
HO
NHMe
S9-1
1.00 1.02 1.11 1.11 0.97 0.97 0.93
2.25
2.34
3.23
3.06
8.0 7.5 7.0 6.5 6.0 5.5 5.0 4.5 4.0 3.5 3.0 2.5 2.0 1.5 1.0 0.5 0.0
f1 (ppm)

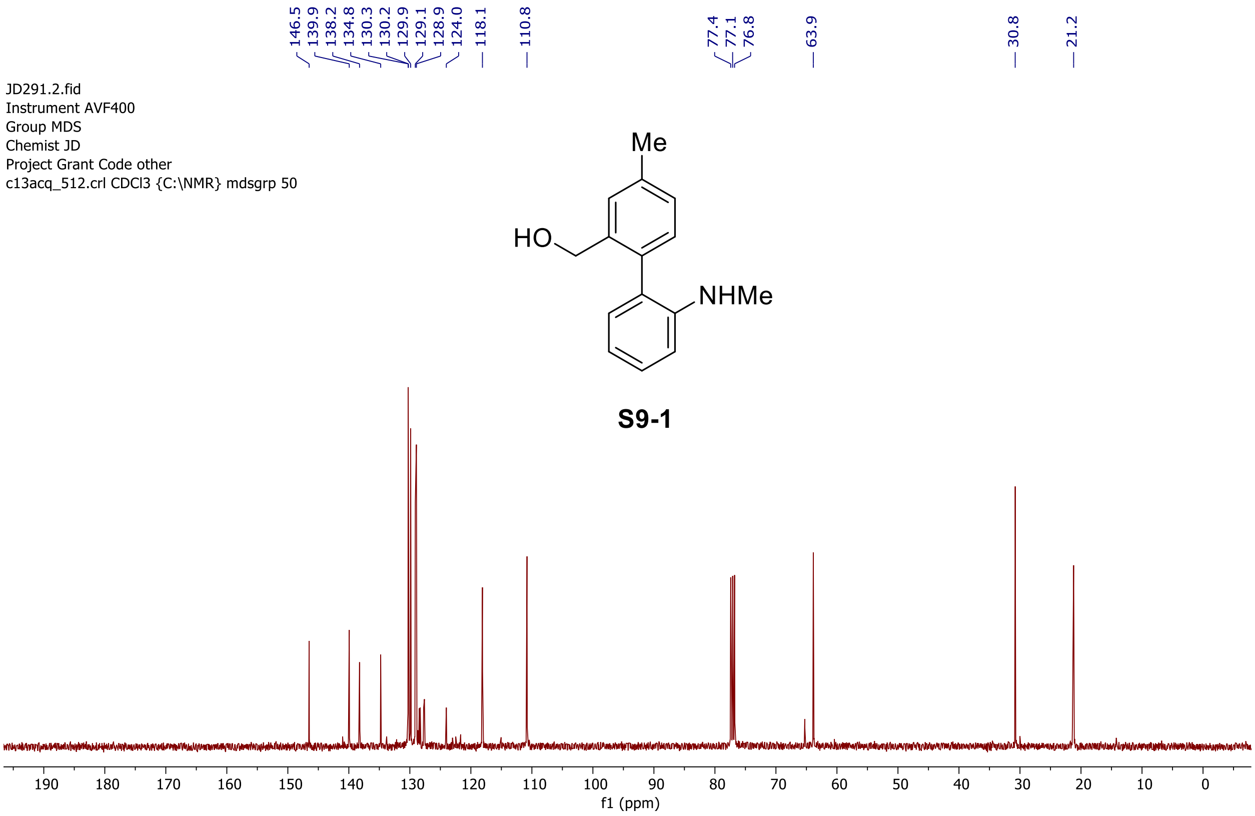
146.5 139.9 138.2 134.8 130.3 130.2 129.9 129.1 128.9 124.0 118.1 110.8
77.4 77.1 76.8
63.9
30.8
21.2
JD291.2.fid
Instrument AVF400
Group MDS
Chemist JD
Project Grant Code other
c13acq_512.crl CDCl3 {C:\NMR} mdsgrp 50
Me
HO
NHMe
S9-1
190 180 170 160 150 140 130 120 110 100 90 80 70 60 50 40 30 20 10 0
f1 (ppm)

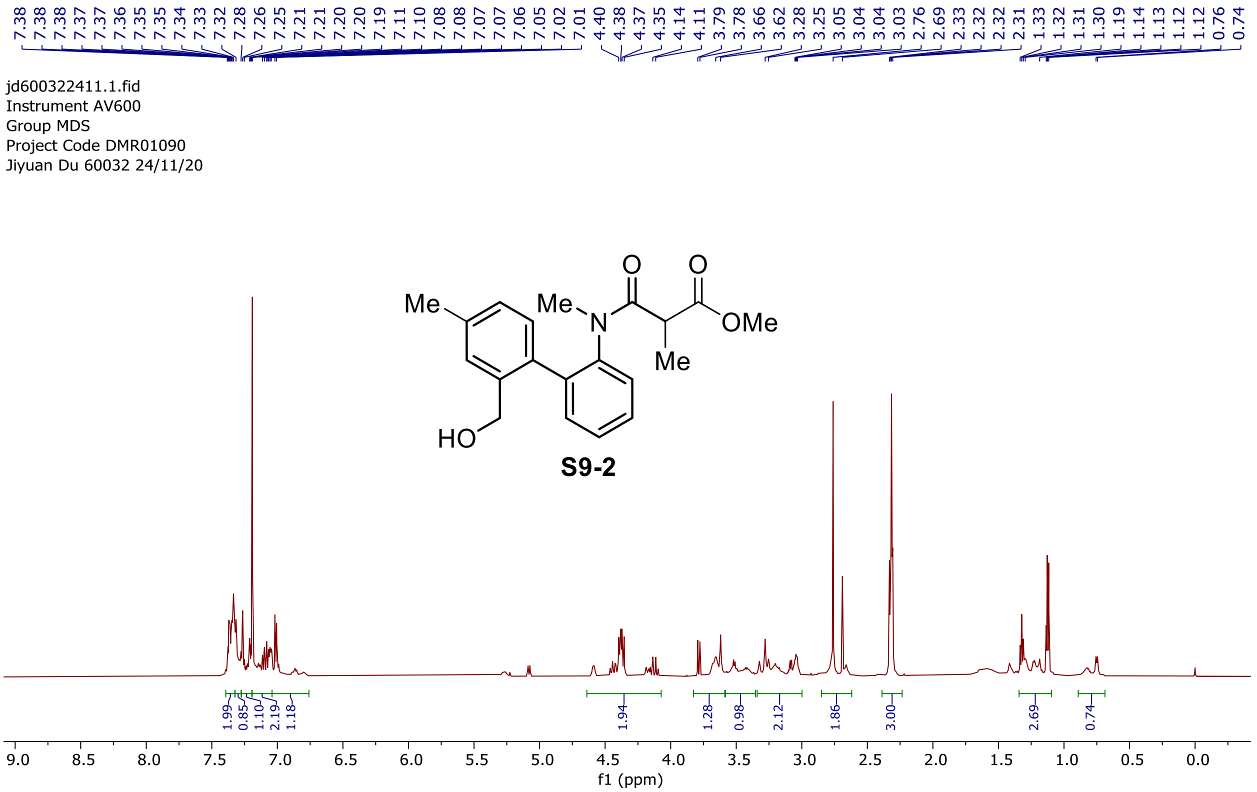
7.38 7.38 7.38 7.37 7.37 7.36 7.35 7.35 7.34 7.33 7.32 7.28 7.26 7.25 7.21 7.21 7.20 7.20 7.19 7.11 7.10 7.08 7.08 7.07 7.07 7.06 7.05 7.02 7.01 4.40 4.38 4.37 4.35 4.14 4.11 3.79 3.78 3.66 3.62 3.28 3.25 3.05 3.04 3.04 3.03 2.76 2.69 2.33 2.32 2.32 2.31 1.33 1.32 1.31 1.30 1.19 1.14 1.13 1.12 1.12 0.76 0.74
jd600322411.1.fid
Instrument AV600
Group MDS
Project Code DMR01090
Jiyuan Du 60032 24/11/20
Me
Me
N
O
O
OMe
Me
HO
S9-2
1.99 0.85 1.10 2.19 1.18 1.94 1.28 0.98 2.12 1.86 3.00 2.69 0.74
f1 (ppm)

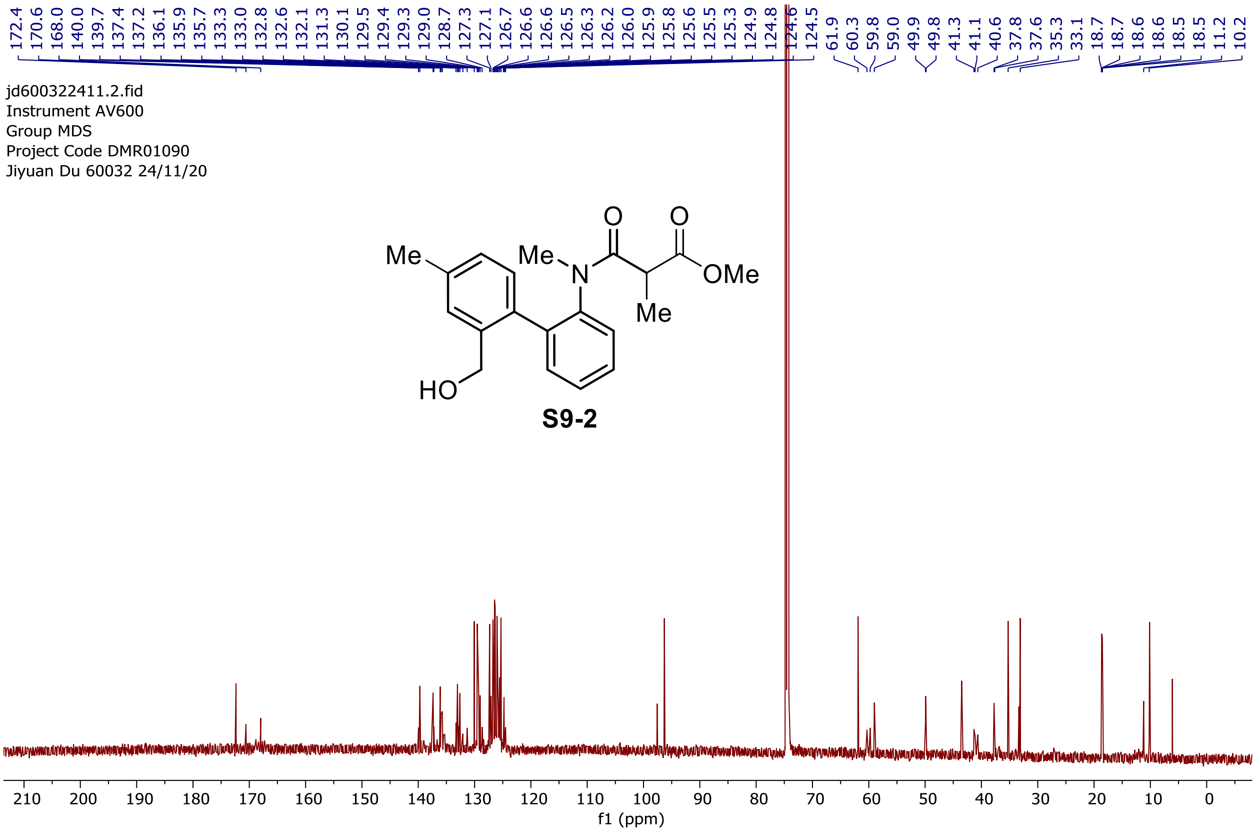
172.4 170.6 168.0 140.0 139.7 137.4 137.2 136.1 135.9 135.7 133.3 133.0 132.8 132.6 132.1 131.3 130.1 129.5 129.4 129.3 129.0 128.7 127.3 127.1 126.7 126.6 126.6 126.5 126.3 126.2 126.0 125.9 125.8 125.6 125.5 125.3 124.9 124.8 124.6 124.5 61.9 60.3 59.8 59.0 49.9 49.8 41.3 41.1 40.6 37.8 37.6 35.3 33.1 18.7 18.7 18.6 18.6 18.5 18.5 11.2 10.2
jd600322411.2.fid
Instrument AV600
Group MDS
Project Code DMR01090
Jiyuan Du 60032 24/11/20
Me
Me
N
O
O
OMe
Me
HO
S9-2
f1 (ppm)

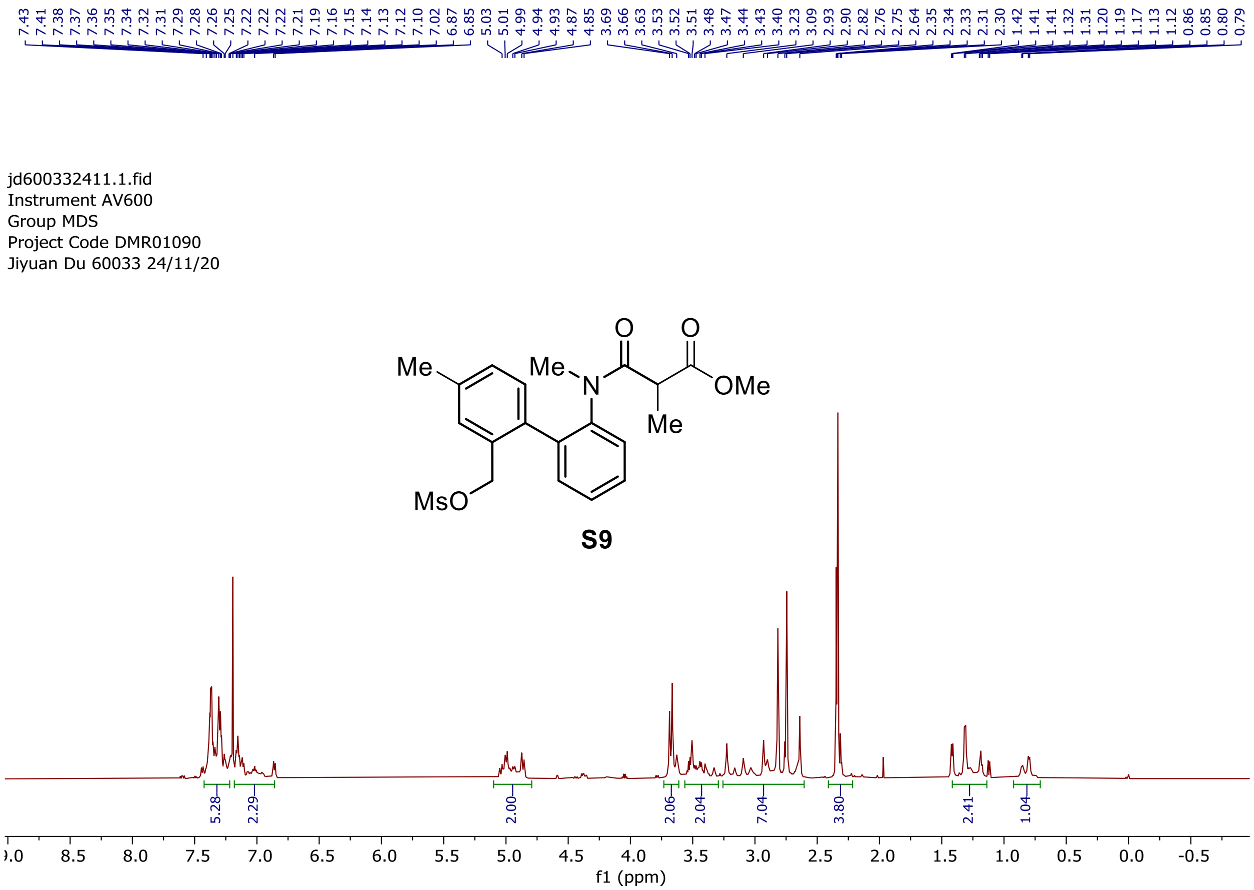
7.43 7.41 7.38 7.37 7.36 7.35 7.34 7.32 7.31 7.29 7.28 7.26 7.25 7.22 7.22 7.22 7.21 7.19 7.16 7.15 7.14 7.13 7.12 7.10 7.02 6.87 6.85 5.03 5.01 4.99 4.94 4.93 4.87 4.85 3.69 3.66 3.63 3.53 3.52 3.51 3.48 3.47 3.44 3.43 3.40 3.23 3.09 2.93 2.90 2.82 2.76 2.75 2.64 2.35 2.34 2.33 2.31 2.30 1.42 1.41 1.32 1.31 1.20 1.19 1.17 1.13 1.12 0.86 0.85 0.80 0.79
jd600332411.1.fid
Instrument AV600
Group MDS
Project Code DMR01090
Jiyuan Du 60033 24/11/20
Me
Me
N
O
O
OMe
Me
MsO
S9
5.28 2.29 2.00 2.06 2.04 7.04 3.80 2.41 1.04
f1 (ppm)

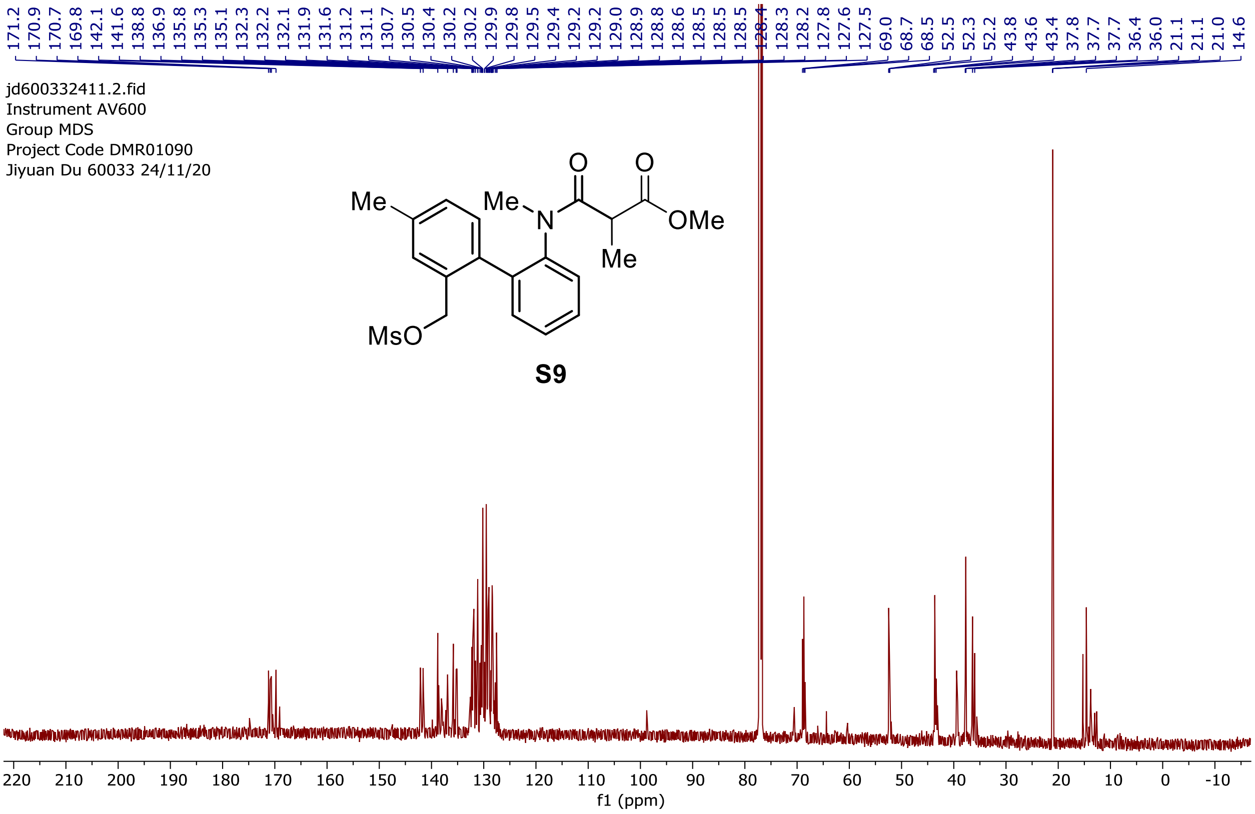
171.2 170.9 170.7 169.8 142.1 141.6 138.8 136.9 135.8 135.3 135.1 132.3 132.2 132.1 131.9 131.6 131.2 131.1 130.7 130.5 130.4 130.2 130.2 129.9 129.8 129.5 129.4 129.2 129.2 129.0 128.9 128.8 128.6 128.5 128.5 128.5 128.4 128.3 128.2 127.8 127.6 127.5 69.0 68.7 68.5 52.5 52.3 52.2 43.8 43.6 43.4 37.8 37.7 37.7 36.4 36.0 21.1 21.1 21.0 14.6
jd600332411.2.fid
Instrument AV600
Group MDS
Project Code DMR01090
Jiyuan Du 60033 24/11/20
Me
Me
N
O
O
OMe
Me
MsO
S9
f1 (ppm)

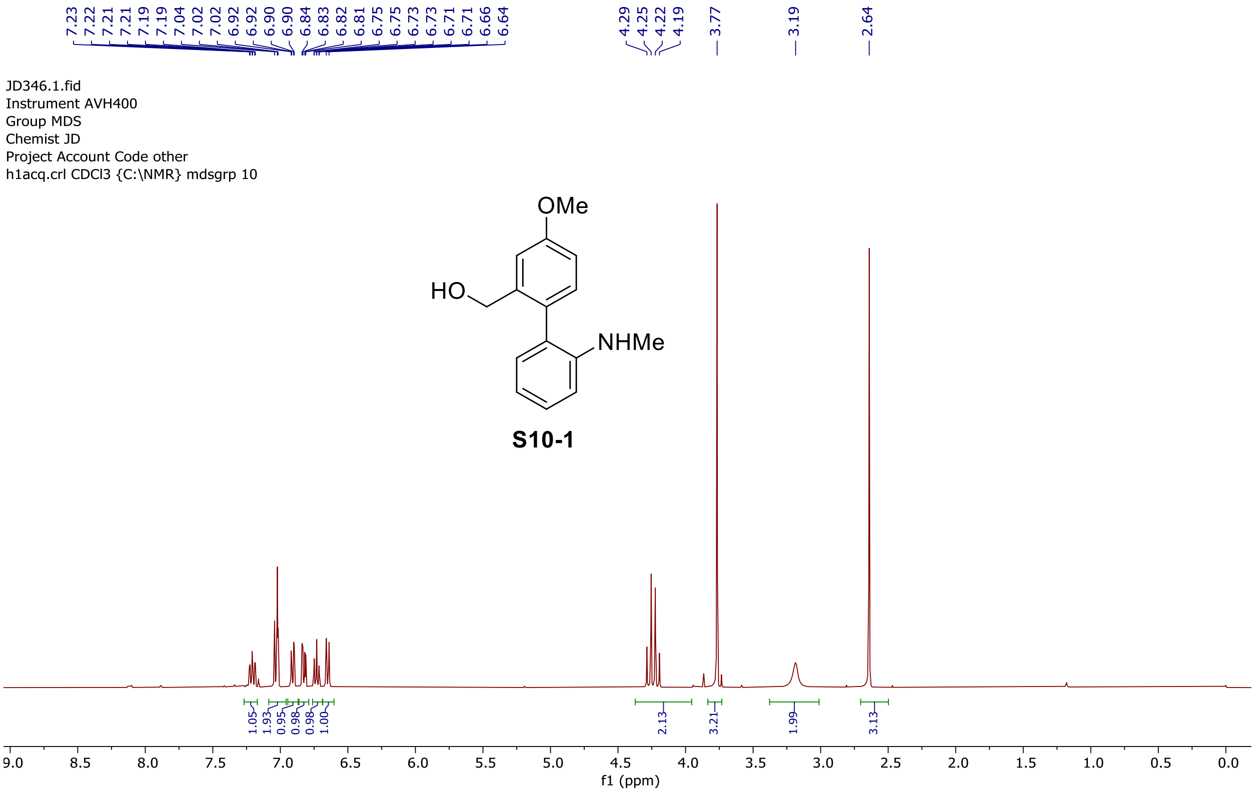
7.23 7.22 7.21 7.21 7.19 7.19 7.04 7.02 7.02 6.92 6.92 6.90 6.90 6.84 6.83 6.82 6.81 6.75 6.75 6.73 6.73 6.71 6.71 6.66 6.64
4.29 4.25 4.22 4.19 3.77 3.19 2.64
JD346.1.fid
Instrument AVH400
Group MDS
Chemist JD
Project Account Code other
h1acq.crl CDCl3 {C:\NMR} mdsgrp 10
OMe
HO
NHMe
S10-1
1.05 1.93 0.95 0.98 0.98 1.00 2.13 3.21 1.99 3.13
f1 (ppm)

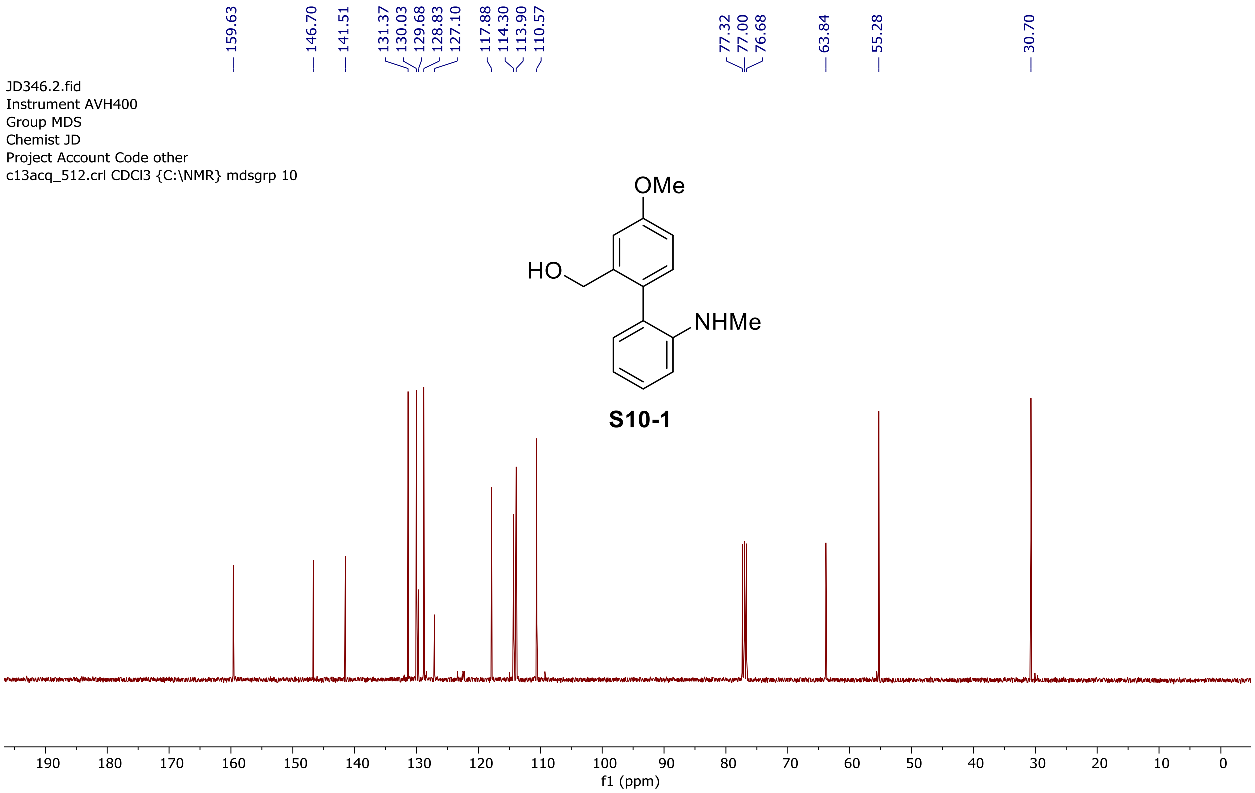
159.63 146.70 141.51 131.37 130.03 129.68 128.83 127.10 117.88 114.30 113.90 110.57 77.32 77.00 76.68 63.84 55.28 30.70
JD346.2.fid
Instrument AVH400
Group MDS
Chemist JD
Project Account Code other
c13acq_512.crl CDCl3 {C:\NMR} mdsgrp 10
OMe
HO
NHMe
S10-1
f1 (ppm)

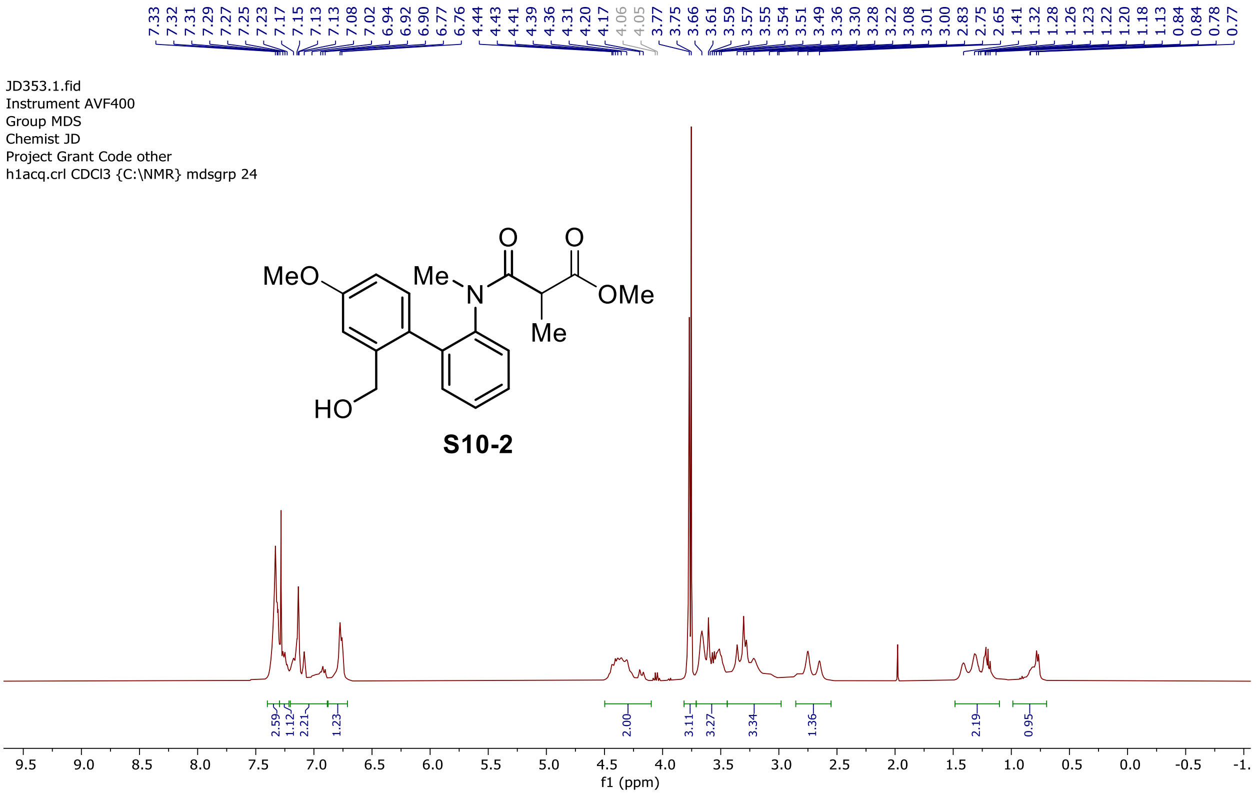
7.33 7.32 7.31 7.29 7.27 7.25 7.23 7.17 7.15 7.13 7.13 7.08 7.02 6.94 6.92 6.90 6.77 6.76 4.44 4.43 4.41 4.39 4.36 4.31 4.20 4.17 4.06 4.05 3.77 3.75 3.66 3.61 3.59 3.57 3.55 3.54 3.51 3.49 3.36 3.30 3.28 3.22 3.08 3.01 3.00 2.83 2.75 2.65 1.41 1.32 1.28 1.26 1.23 1.22 1.20 1.18 1.13 0.84 0.84 0.78 0.77
JD353.1.fid
Instrument AVF400
Group MDS
Chemist JD
Project Grant Code other
h1acq.crl CDCl3 {C:\NMR} mdsgrp 24
MeO
Me
N
O
O
OMe
Me
HO
S10-2
2.59 1.12 2.21 1.23 2.00 3.11 3.27 3.34 1.36 2.19 0.95
f1 (ppm)

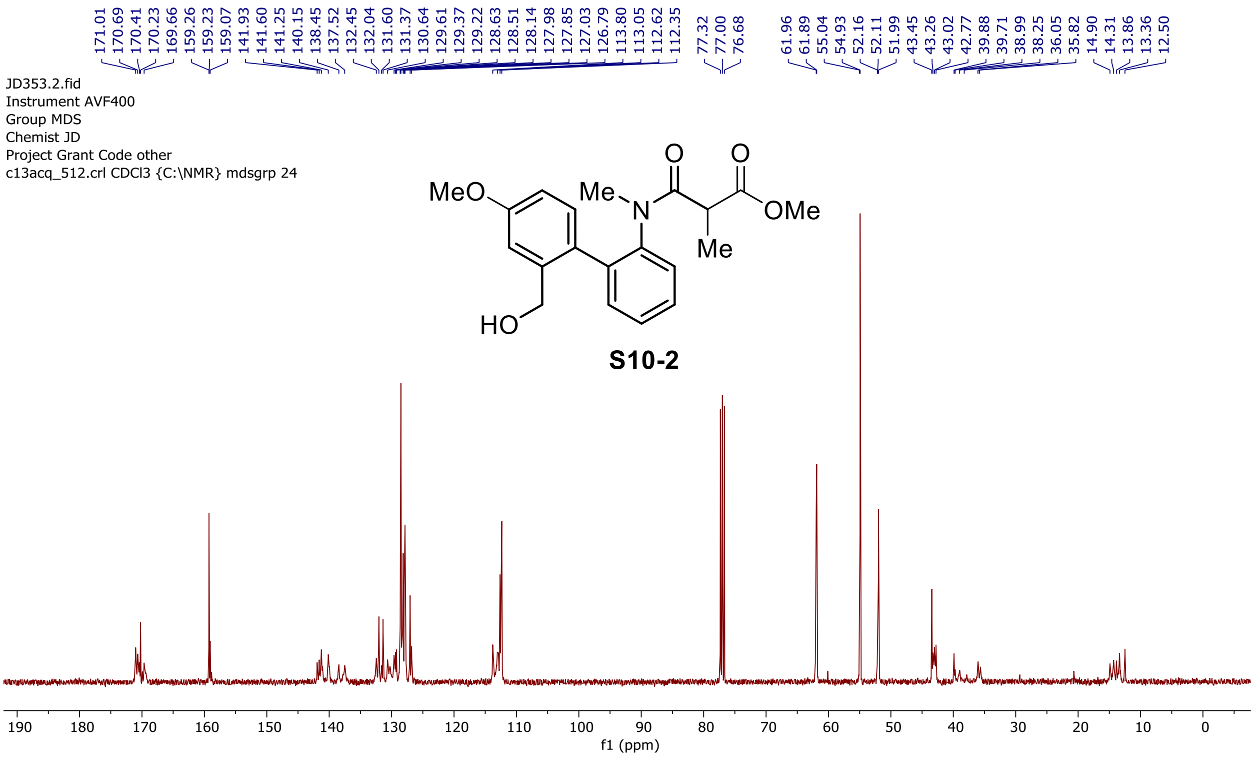
171.01 170.69 170.41 170.23 169.66 159.26 159.23 159.07 141.93 141.60 141.25 140.15 138.45 137.52 132.45 132.04 131.60 131.37 130.64 129.61 129.37 129.22 128.63 128.51 128.14 127.98 127.85 127.03 126.79 113.80 113.05 112.62 112.35 77.32 77.00 76.68 61.96 61.89 55.04 54.93 52.16 52.11 51.99 43.45 43.26 43.02 42.77 39.88 39.71 38.99 38.25 36.05 35.82 14.90 14.31 13.86 13.36 12.50
JD353.2.fid
Instrument AVF400
Group MDS
Chemist JD
Project Grant Code other
c13acq_512.crl CDCl3 {C:\NMR} mdsgrp 24
MeO
Me
N
O
O
OMe
Me
HO
S10-2
f1 (ppm)

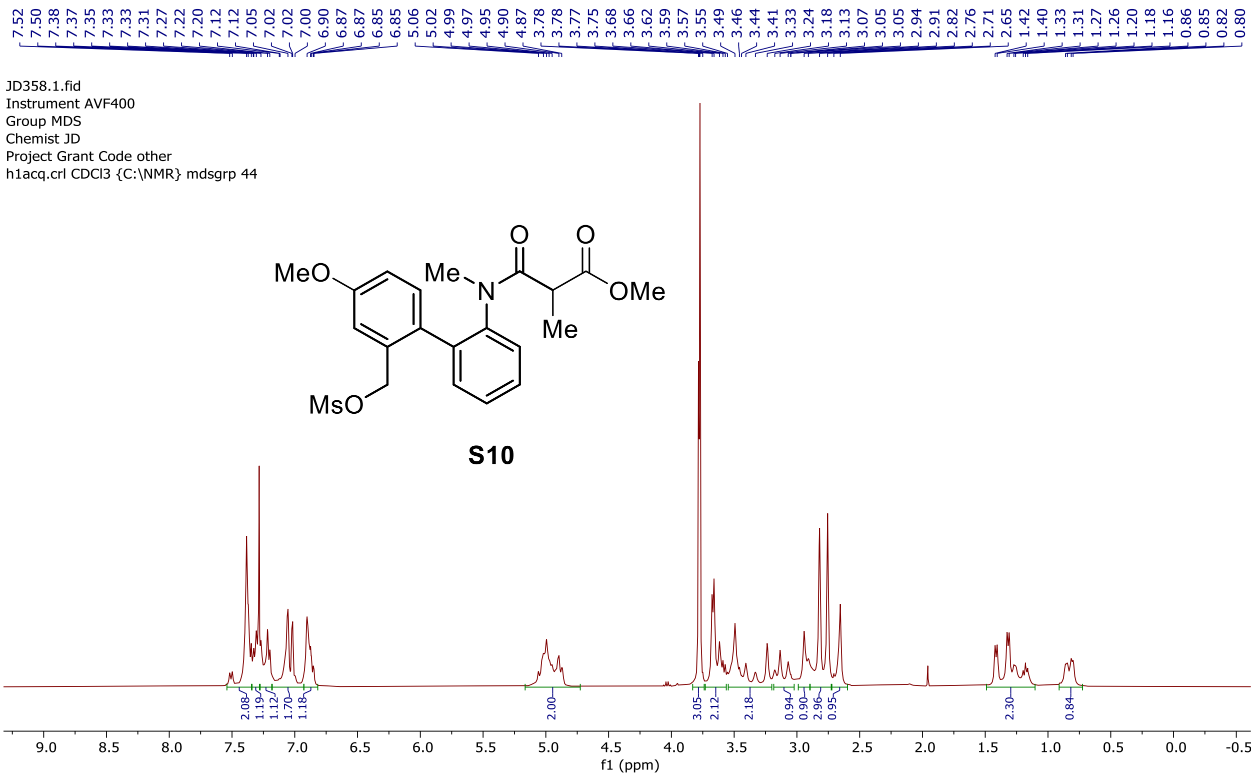
7.52 7.50 7.38 7.37 7.35 7.33 7.33 7.31 7.27 7.22 7.20 7.12 7.12 7.05 7.02 7.02 7.00 6.90 6.87 6.85 6.85 5.06 5.02 4.99 4.97 4.95 4.90 4.87 3.78 3.78 3.77 3.75 3.68 3.66 3.62 3.59 3.57 3.55 3.49 3.46 3.44 3.41 3.33 3.24 3.18 3.13 3.07 3.05 3.05 2.94 2.91 2.82 2.76 2.71 2.65 1.42 1.40 1.33 1.31 1.27 1.26 1.20 1.18 1.16 0.86 0.85 0.82 0.80
JD358.1.fid
Instrument AVF400
Group MDS
Chemist JD
Project Grant Code other
h1acq.crl CDCl3 {C:\NMR} mdsgrp 44
MeO
Me
N
O
O
OMe
Me
MsO
S10
2.08 1.19 1.12 1.70 1.18 2.00 3.05 2.12 2.18 0.94 0.90 2.96 0.95 2.30 0.84
9.0 8.5 8.0 7.5 7.0 6.5 6.0 5.5 5.0 4.5 4.0 3.5 3.0 2.5 2.0 1.5 1.0 0.5 0.0 -0.5
f1 (ppm)

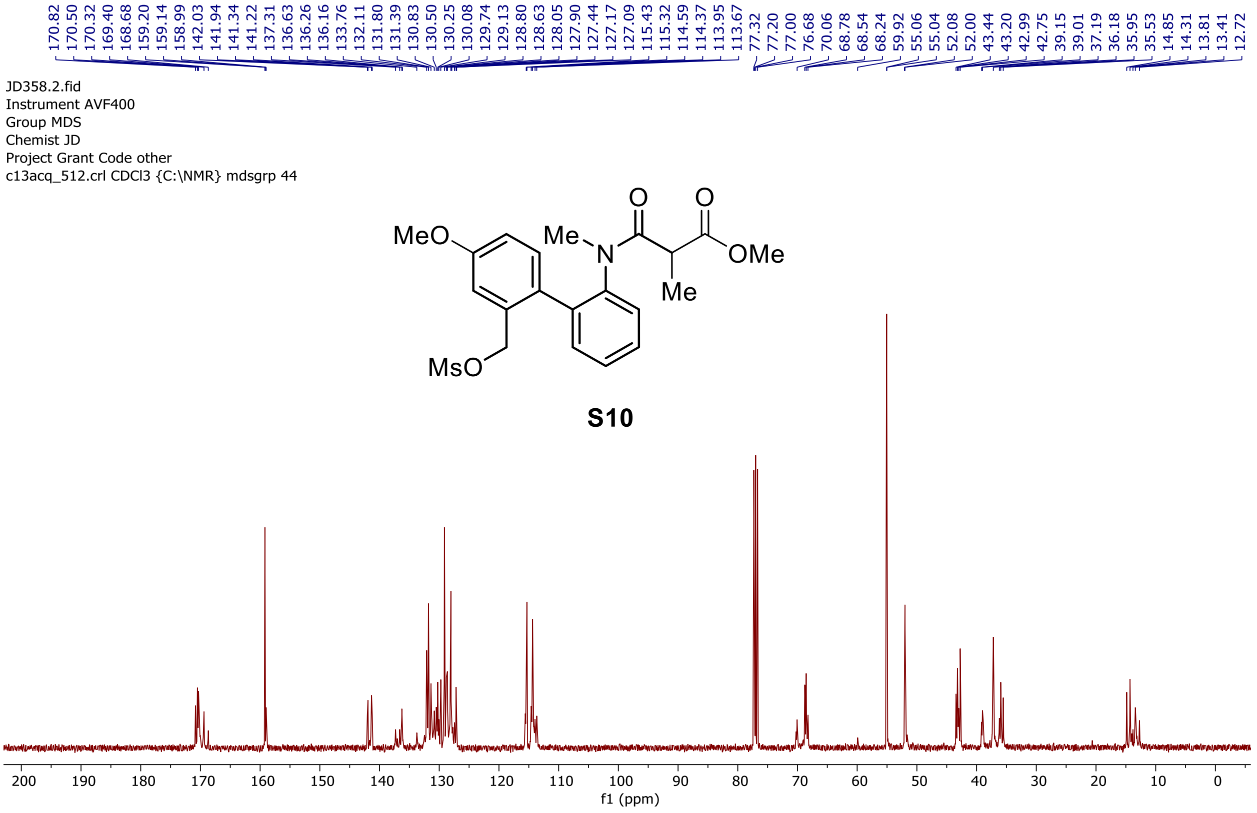
170.82 170.50 170.32 169.40 168.68 159.20 159.14 158.99 142.03 141.94 141.34 141.22 137.31 136.63 136.26 136.16 133.76 132.11 131.80 131.39 130.83 130.50 130.25 130.08 129.74 129.13 128.80 128.63 128.05 127.90 127.44 127.17 127.09 115.43 115.32 114.59 114.37 113.95 113.67 77.32 77.20 77.00 76.68 70.06 68.78 68.54 68.24 59.92 55.06 55.04 52.08 52.00 43.44 43.20 42.99 42.75 39.15 39.01 37.19 36.18 35.95 35.53 14.85 14.31 13.81 13.41 12.72
JD358.2.fid
Instrument AVF400
Group MDS
Chemist JD
Project Grant Code other
c13acq_512.crl CDCl3 {C:\NMR} mdsgrp 44
MeO
Me
N
O
O
OMe
Me
MsO
S10
200 190 180 170 160 150 140 130 120 110 100 90 80 70 60 50 40 30 20 10 0
f1 (ppm)

JD556.1.fid
Instrument AVF400
Group MDS
Chemist JD
Project Grant Code other
h1acq.crl CDCl3 {C:\NMR} mdsgrp 29

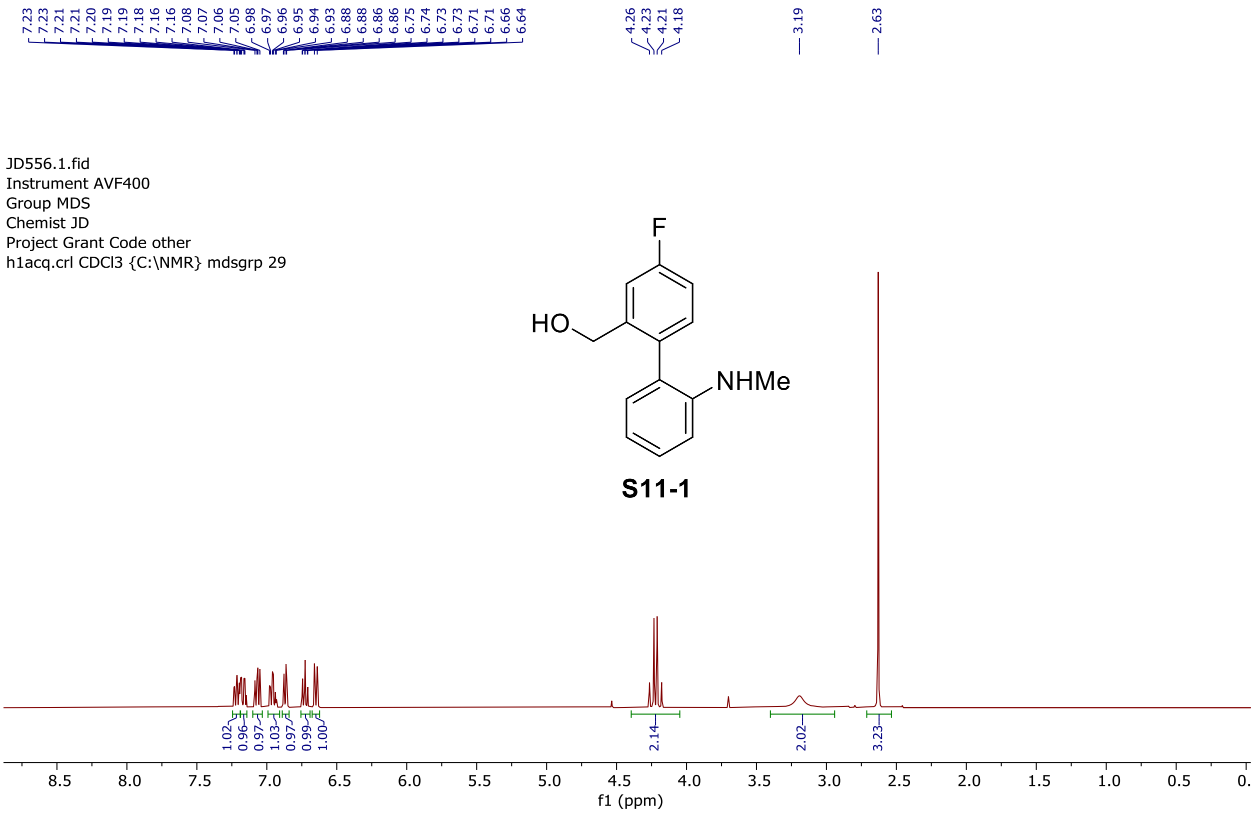

S11-1

JD556-C.1.fid
Instrument AVF400
Group MDS
Chemist JD
Project Grant Code other
c13acq_512.crl CDCl3 {C:\NMR} mdsgrp 45

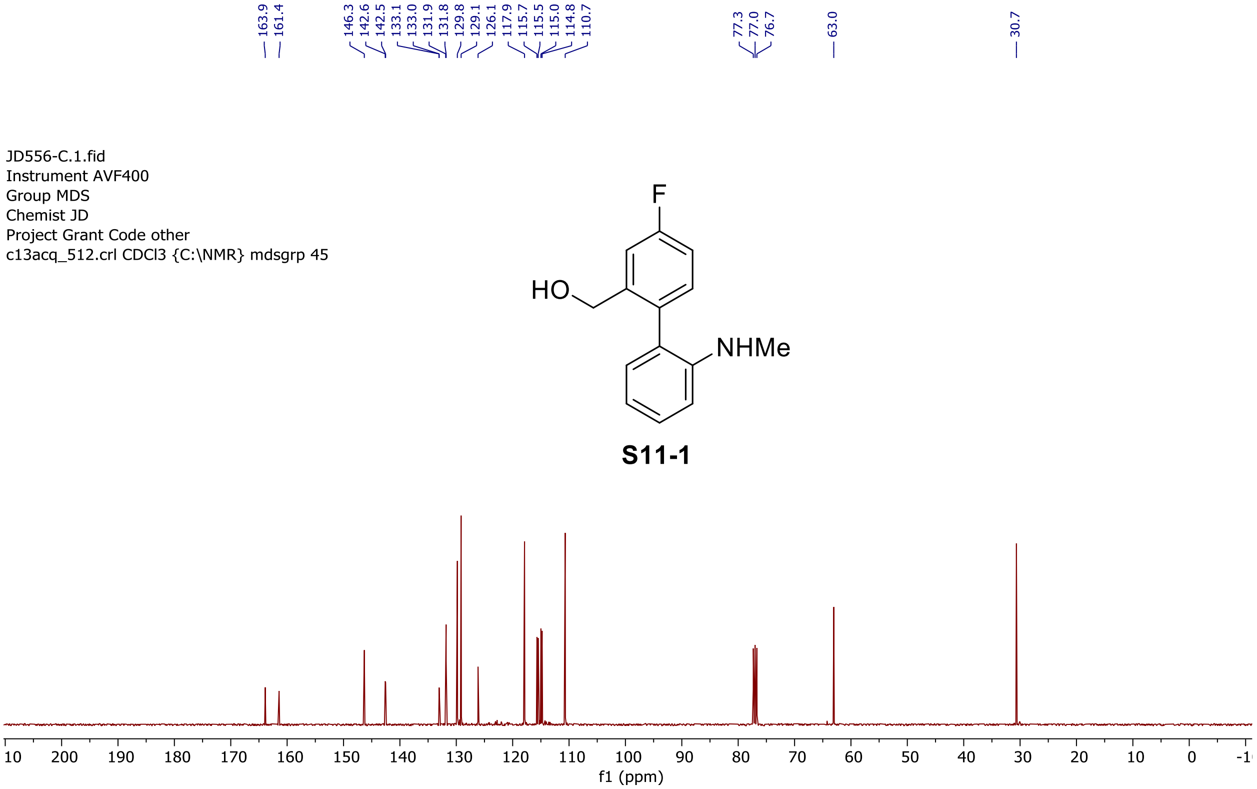

S11-1

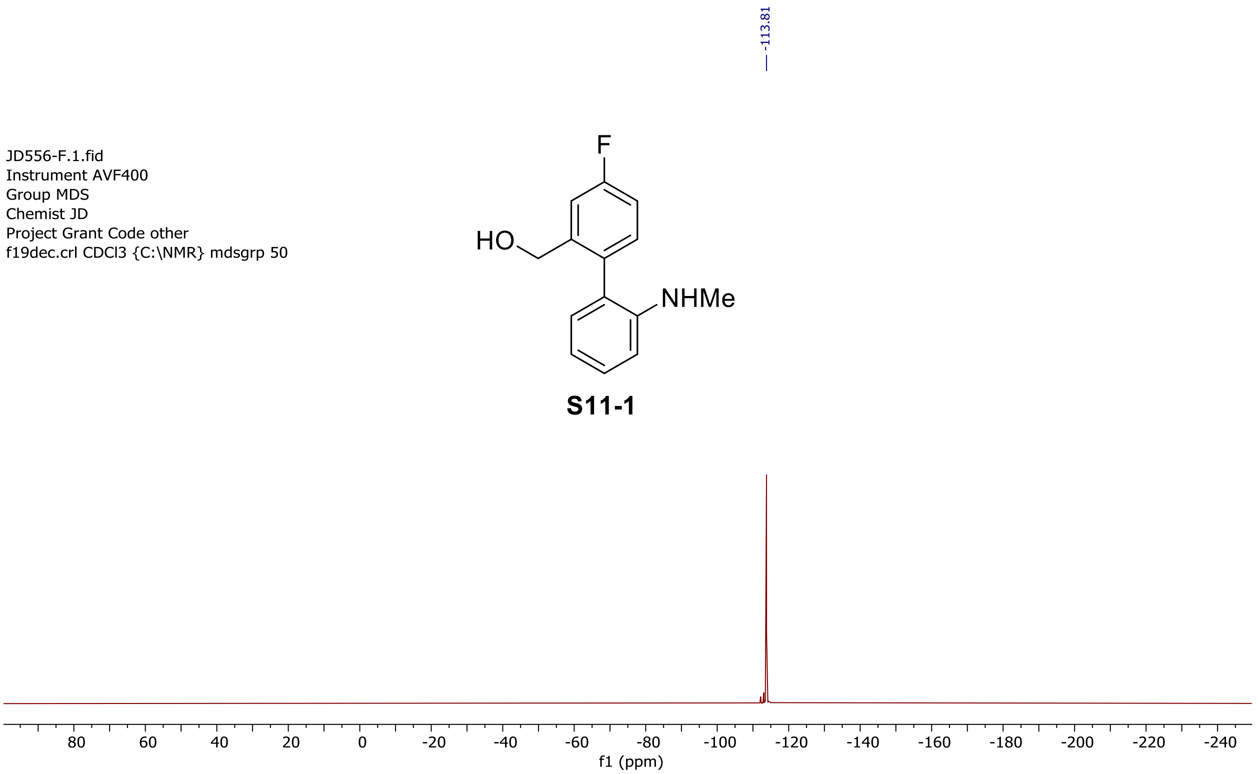
-113.81
JD556-F.1.fid
Instrument AVF400
Group MDS
Chemist JD
Project Grant Code other
f19dec.crl CDCl3 {C:\NMR} mdsgrp 50
F
HO
NHMe
S11-1
f1 (ppm)

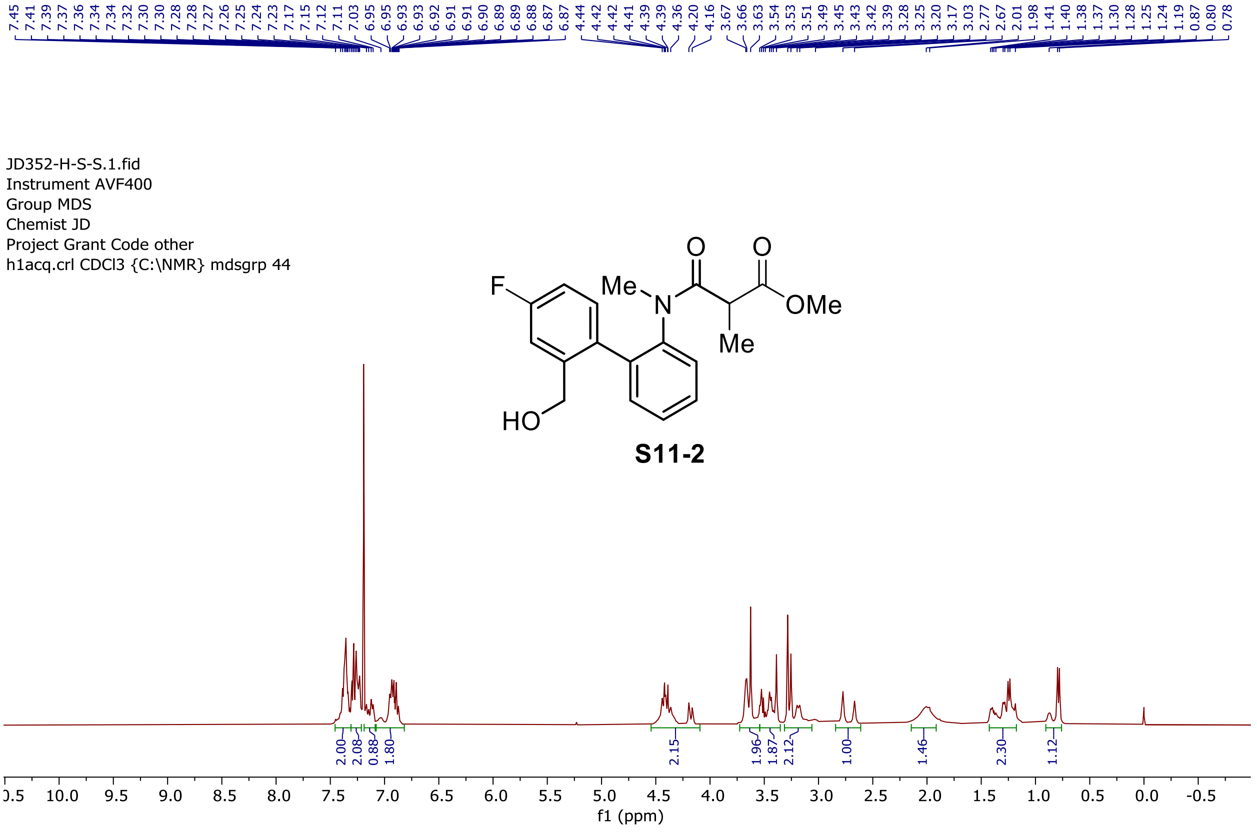
7.45 7.41 7.39 7.37 7.36 7.34 7.34 7.32 7.30 7.30 7.28 7.28 7.27 7.26 7.25 7.24 7.23 7.17 7.15 7.12 7.11 7.03 6.95 6.95 6.93 6.92 6.91 6.91 6.90 6.89 6.89 6.88 6.87 6.87 4.44 4.42 4.41 4.39 4.39 4.36 4.20 4.16 3.67 3.66 3.63 3.54 3.53 3.51 3.49 3.45 3.43 3.42 3.39 3.28 3.25 3.20 3.17 3.03 2.77 2.67 2.01 1.98 1.41 1.40 1.38 1.37 1.30 1.28 1.25 1.24 1.19 0.87 0.80 0.78
JD352-H-S-S.1.fid
Instrument AVF400
Group MDS
Chemist JD
Project Grant Code other
h1acq.crl CDCl3 {C:\NMR} mdsgrp 44
F
Me
N
O
O
OMe
Me
HO
S11-2
2.00 2.08 0.88 1.80 2.15 1.96 1.87 2.12 1.00 1.46 2.30 1.12
f1 (ppm)

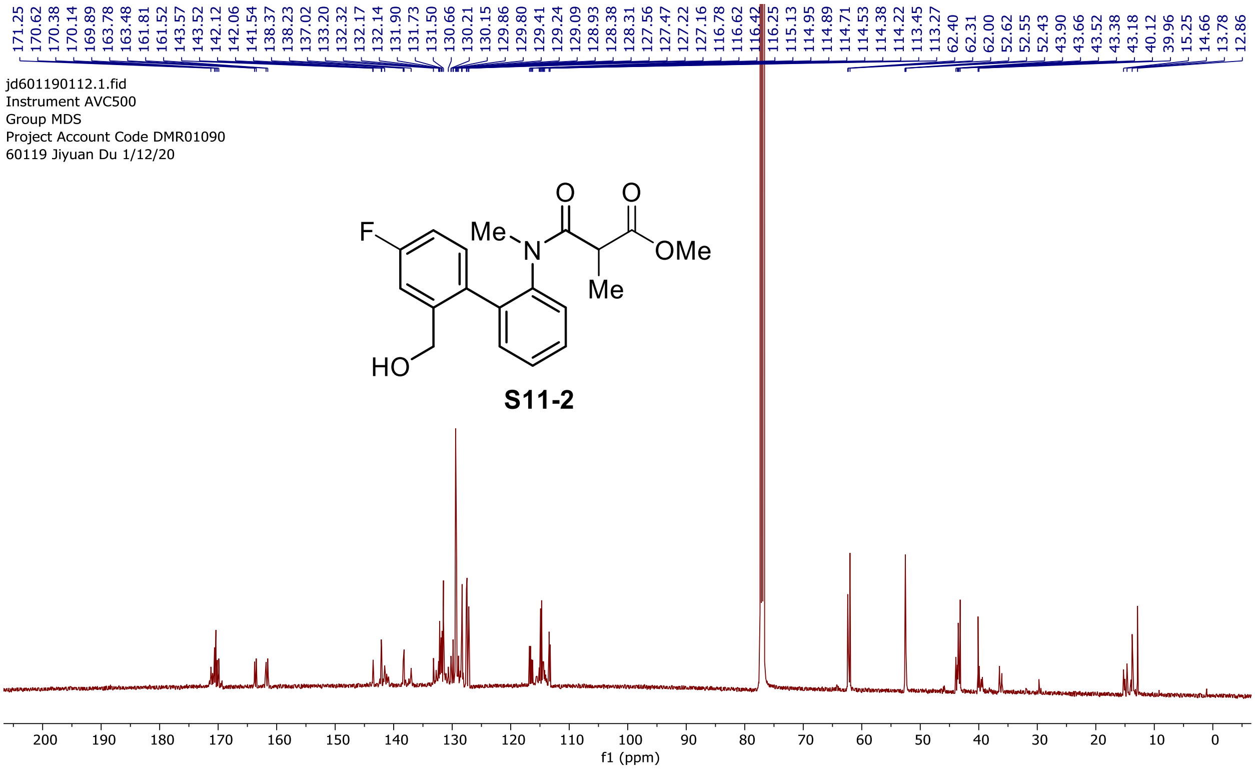
171.25 170.62 170.38 170.14 169.89 163.78 163.48 161.81 161.52 143.57 143.52 142.12 142.06 141.54 138.37 138.23 137.02 133.20 132.32 132.17 132.14 131.90 131.73 131.50 130.66 130.21 130.15 129.86 129.80 129.41 129.24 129.09 128.93 128.38 128.31 127.56 127.47 127.22 127.16 116.78 116.62 116.42 116.25 115.13 114.95 114.89 114.71 114.53 114.38 114.22 113.45 113.27 62.40 62.31 62.00 52.62 52.55 52.43 43.90 43.66 43.52 43.38 43.18 40.12 39.96 15.25 14.66 13.78 12.86
jd601190112.1.fid
Instrument AVC500
Group MDS
Project Account Code DMR01090
60119 Jiyuan Du 1/12/20
F
Me
N
O
O
OMe
Me
HO
S11-2
200 190 180 170 160 150 140 130 120 110 100 90 80 70 60 50 40 30 20 10 0
f1 (ppm)

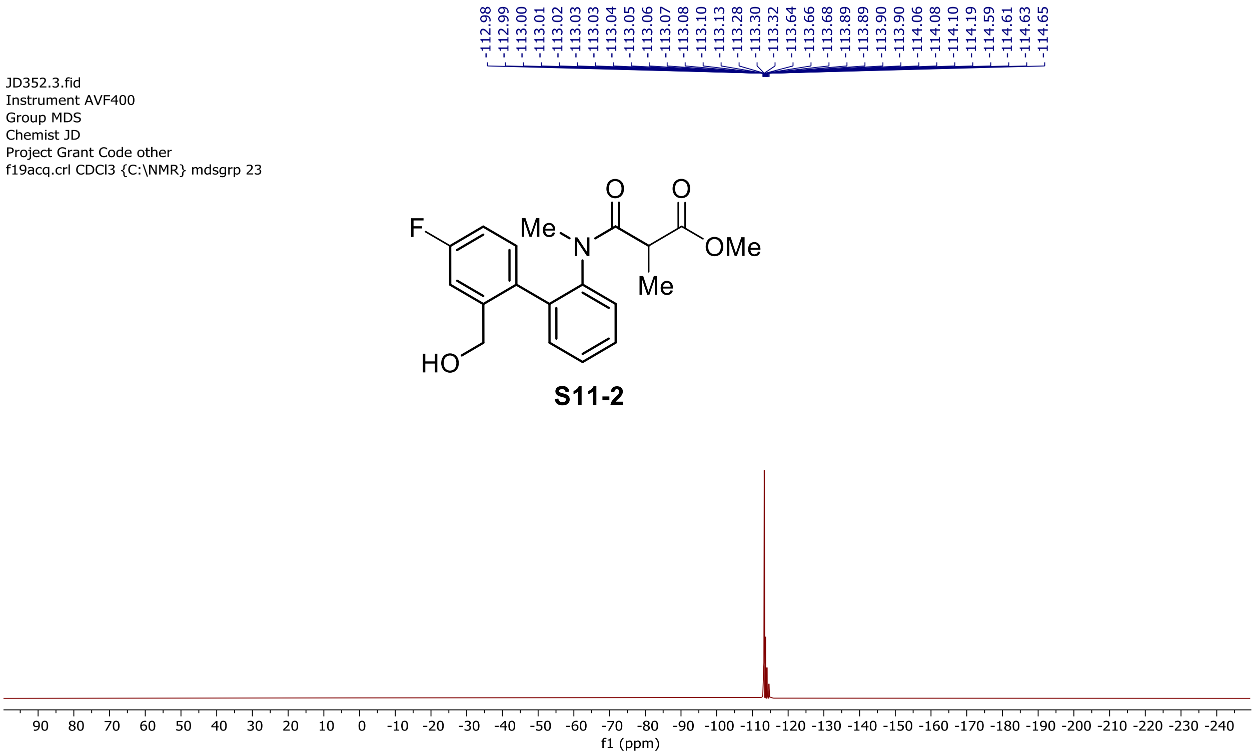
-112.98 -112.99 -113.00 -113.01 -113.02 -113.03 -113.04 -113.05 -113.06 -113.07 -113.08 -113.10 -113.13 -113.28 -113.30 -113.32 -113.64 -113.66 -113.68 -113.89 -113.90 -113.90 -114.06 -114.08 -114.10 -114.19 -114.59 -114.61 -114.63 -114.65
JD352.3.fid
Instrument AVF400
Group MDS
Chemist JD
Project Grant Code other
f19acq.crl CDCl3 {C:\NMR} mdsgrp 23
F
Me
N
O
O
OMe
Me
HO
S11-2
90 80 70 60 50 40 30 20 10 0 -10 -20 -30 -40 -50 -60 -70 -80 -90 -100 -110 -120 -130 -140 -150 -160 -170 -180 -190 -200 -210 -220 -230 -240
f1 (ppm)

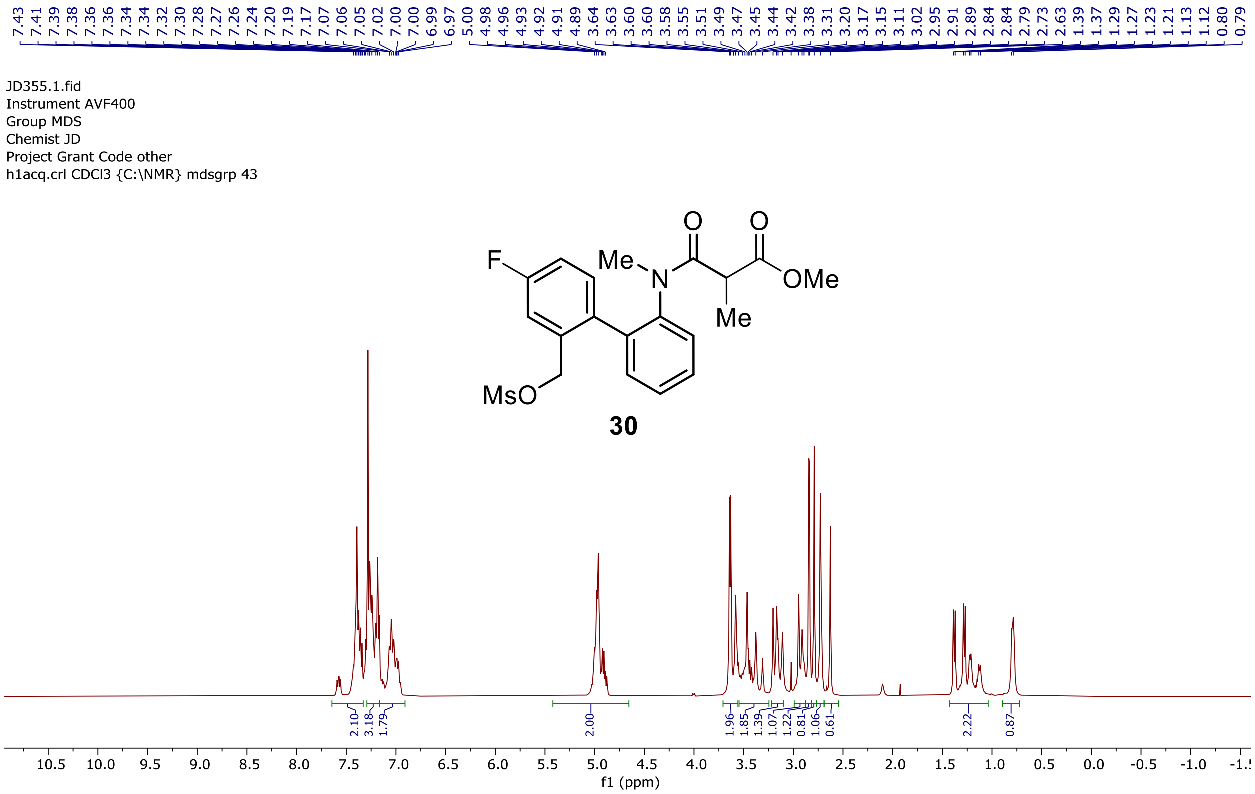
7.43 7.41 7.39 7.38 7.36 7.36 7.34 7.34 7.32 7.30 7.28 7.27 7.26 7.24 7.20 7.19 7.17 7.07 7.06 7.05 7.02 7.00 7.00 6.99 6.97 5.00 4.98 4.96 4.93 4.92 4.91 4.89 3.64 3.63 3.60 3.58 3.55 3.51 3.49 3.47 3.45 3.44 3.42 3.38 3.31 3.20 3.17 3.15 3.11 3.02 2.95 2.91 2.89 2.84 2.84 2.79 2.73 2.63 1.39 1.37 1.29 1.27 1.23 1.21 1.13 1.12 0.80 0.79
JD355.1.fid
Instrument AVF400
Group MDS
Chemist JD
Project Grant Code other
h1acq.crl CDCl3 {C:\NMR} mdsgrp 43
F
Me
N
O
O
OMe
Me
MsO
30
2.10 3.18 1.79 2.00 1.96 1.85 1.39 1.07 1.22 0.81 1.06 0.61 2.22 0.87
10.5 10.0 9.5 9.0 8.5 8.0 7.5 7.0 6.5 6.0 5.5 5.0 4.5 4.0 3.5 3.0 2.5 2.0 1.5 1.0 0.5 0.0 -0.5 -1.0
f1 (ppm)

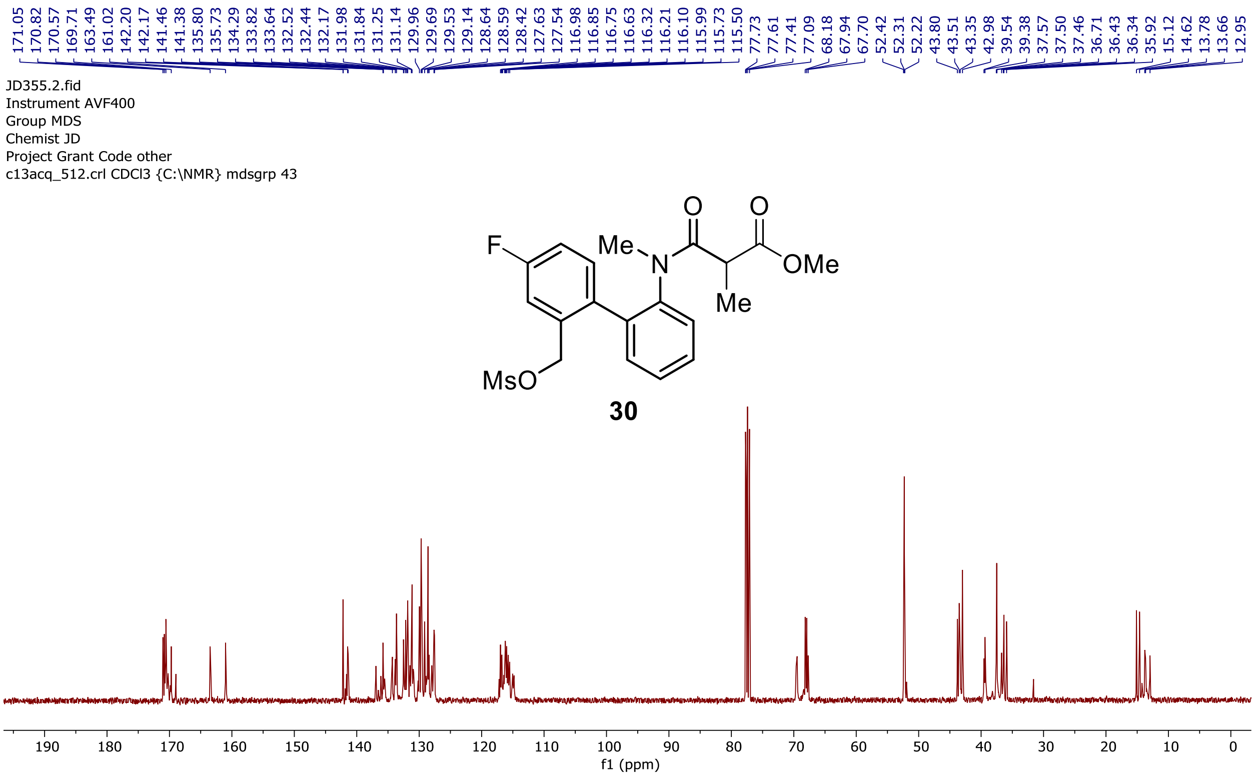
171.05 170.82 170.57 169.71 163.49 161.02 142.20 142.17 141.46 141.38 135.80 135.73 134.29 133.82 133.64 132.52 132.44 132.17 131.98 131.84 131.25 131.14 129.96 129.69 129.53 129.14 128.64 128.59 128.42 127.63 127.54 116.98 116.85 116.75 116.63 116.32 116.21 116.10 115.99 115.73 115.50 77.73 77.61 77.41 77.09 68.18 67.94 67.70 52.42 52.31 52.22 43.80 43.51 43.35 42.98 39.54 39.38 37.57 37.50 37.46 36.71 36.43 36.34 35.92 15.12 14.62 13.78 13.66 12.95
JD355.2.fid
Instrument AVF400
Group MDS
Chemist JD
Project Grant Code other
c13acq_512.crl CDCl3 {C:\NMR} mdsgrp 43
F
Me
N
O
O
OMe
Me
MsO
30
190 180 170 160 150 140 130 120 110 100 90 80 70 60 50 40 30 20 10 0
f1 (ppm)

JD355.3.fid
Instrument AVF400
Group MDS
Chemist JD
Project Grant Code other
f19acq.crl CDCl3 {C:\NMR} mdsgrp 43

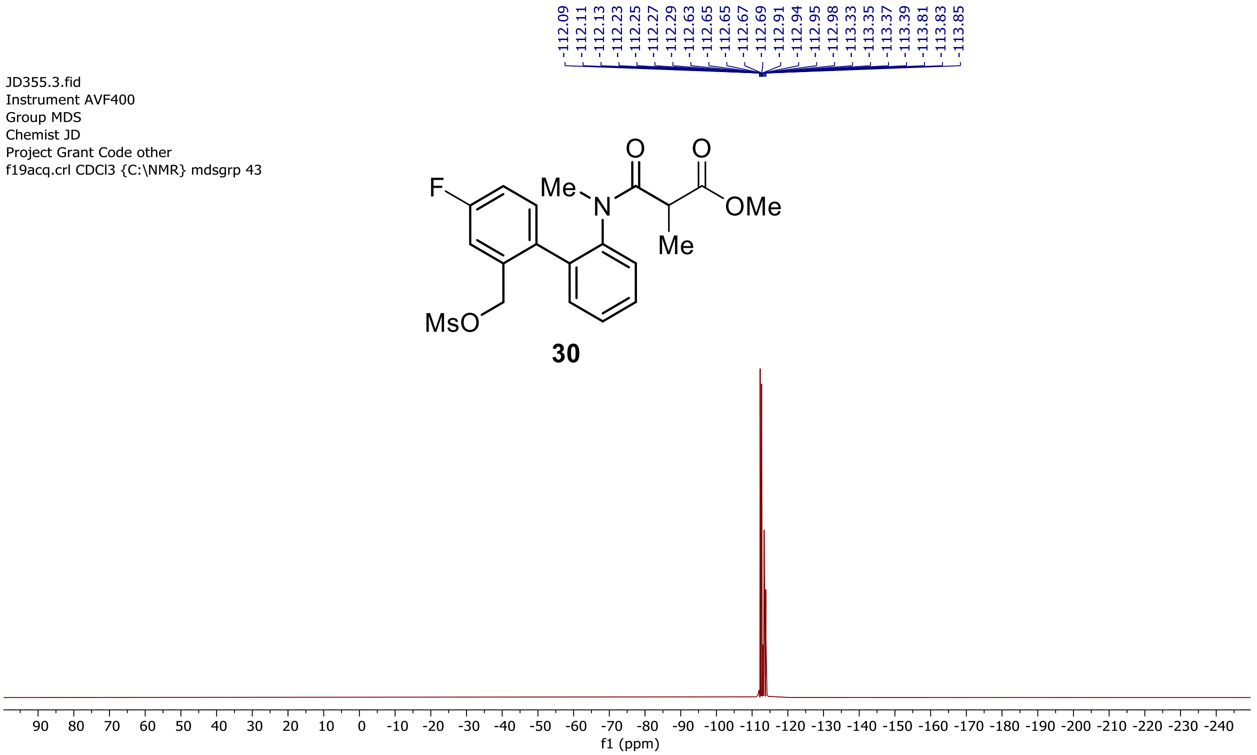

30

JD412-s.1.fid
Instrument AVF400
Group MDS
Chemist JD
Project Grant Code other
h1acq.crl CDCl3 {C:\NMR} mdsgrp 42

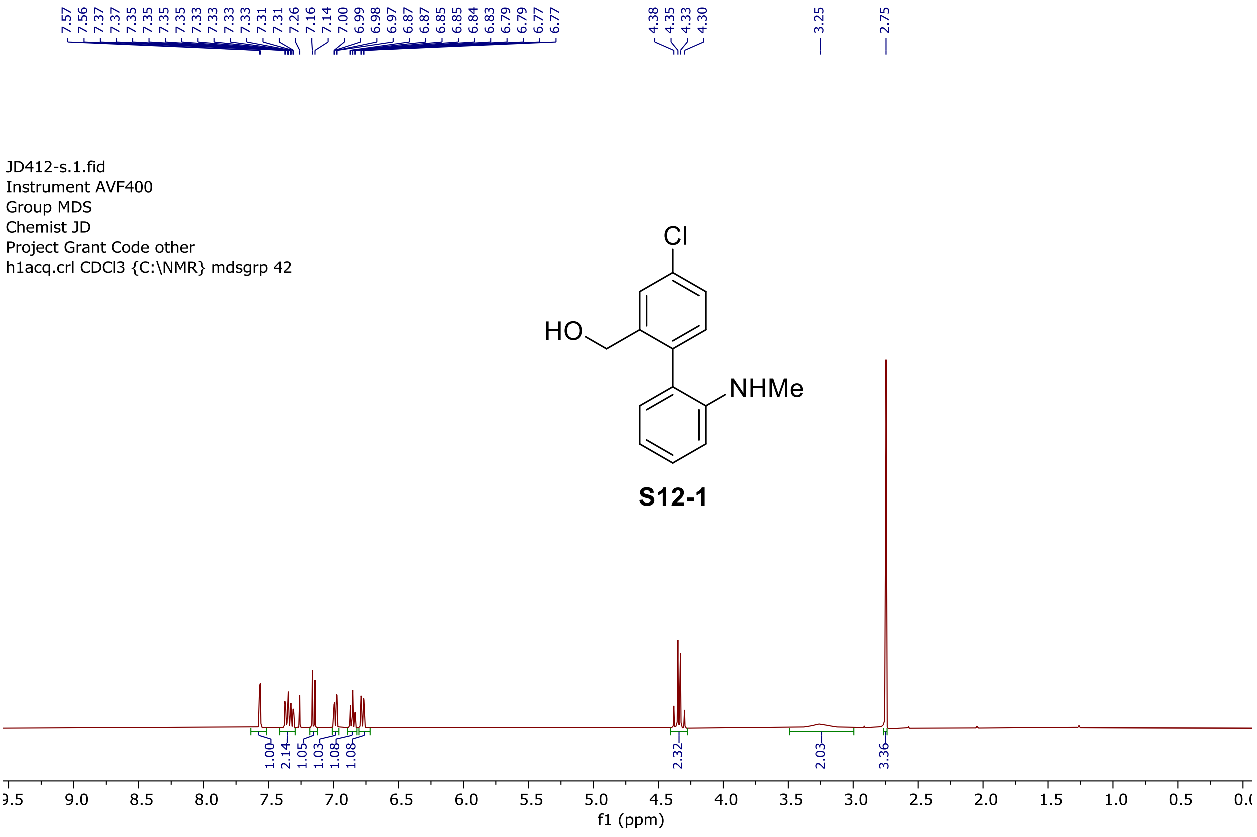

S12-1

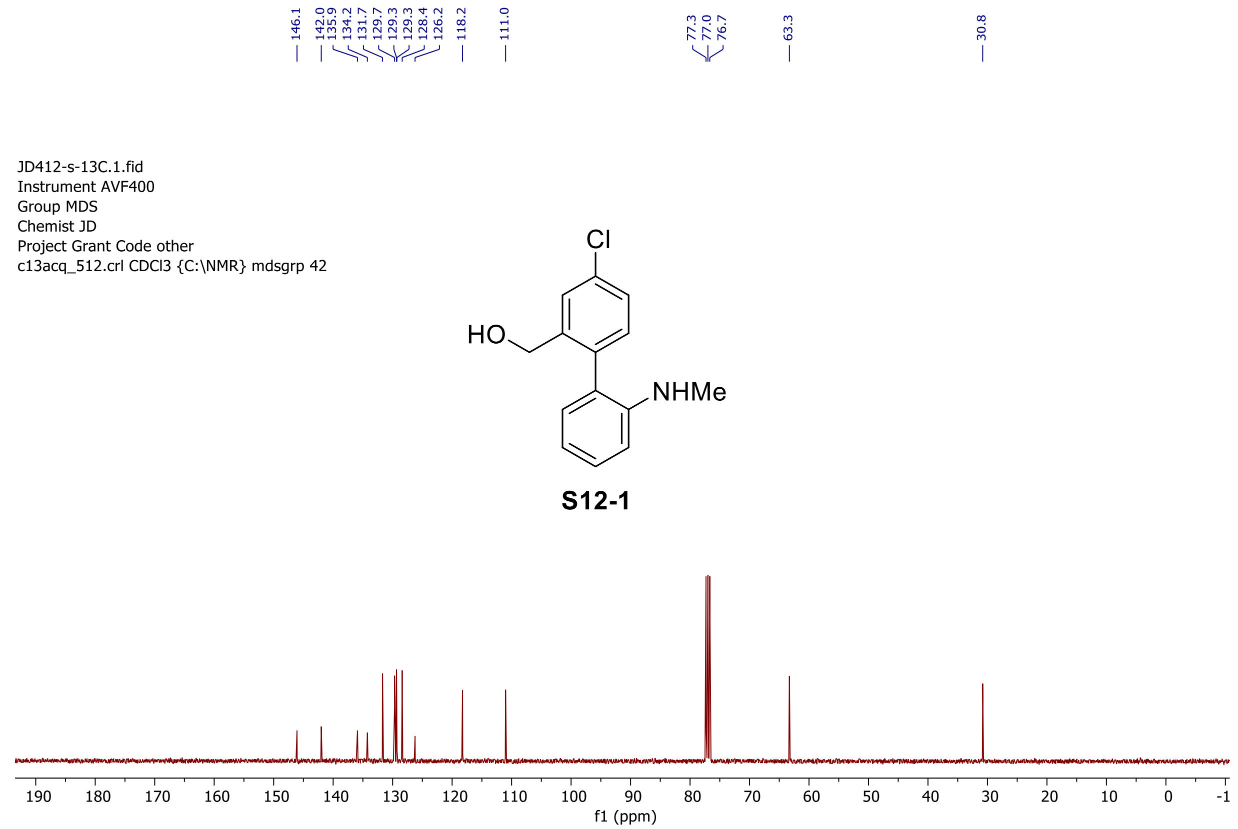
146.1
142.0
135.9
134.2
131.7
129.7
129.3
129.3
128.4
126.2
118.2
111.0
77.3
77.0
76.7
63.3
30.8
JD412-s-13C.1.fid
Instrument AVF400
Group MDS
Chemist JD
Project Grant Code other
c13acq_512.crl CDCl3 {C:\NMR} mdsgrp 42
Cl
HO
NHMe
190 180 170 160 150 140 130 120 110 100 90 80 70 60 50 40 30 20 10 0 -1
f1 (ppm)

S12-1

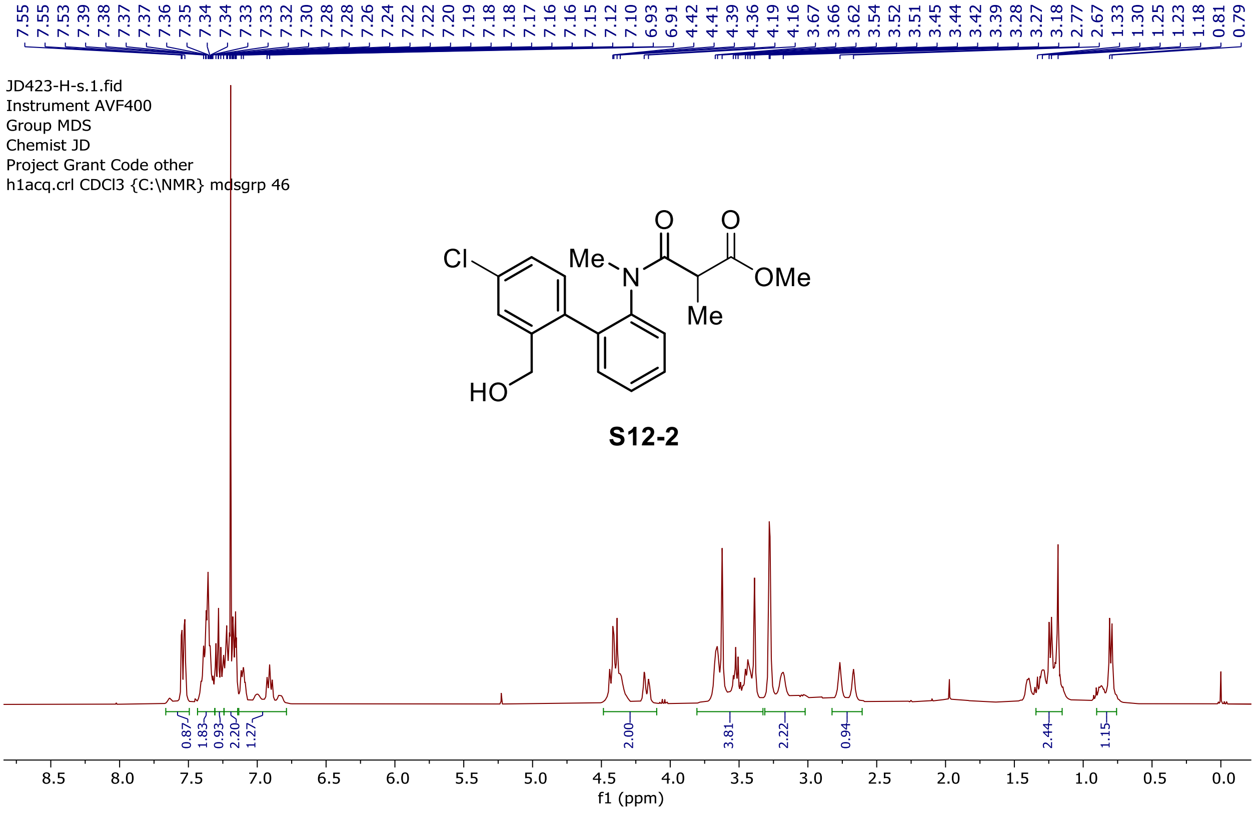
7.55 7.55 7.53 7.39 7.38 7.37 7.37 7.36 7.35 7.34 7.34 7.33 7.32 7.30 7.28 7.28 7.26 7.24 7.22 7.22 7.20 7.19 7.18 7.18 7.17 7.16 7.16 7.15 7.12 7.10 6.93 6.91 4.42 4.41 4.39 4.36 4.19 4.16 3.67 3.66 3.62 3.54 3.52 3.51 3.45 3.44 3.42 3.39 3.28 3.27 3.18 2.77 2.67 1.33 1.30 1.25 1.23 1.18 0.81 0.79
JD423-H-s.1.fid
Instrument AVF400
Group MDS
Chemist JD
Project Grant Code other
h1acq.crl CDCl3 {C:\NMR} mdsgrp 46
Cl
Me
N
O
O
OMe
Me
HO
0.87
1.83
0.93
2.20
1.27
2.00
3.81
2.22
0.94
2.44
1.15
8.5 8.0 7.5 7.0 6.5 6.0 5.5 5.0 4.5 4.0 3.5 3.0 2.5 2.0 1.5 1.0 0.5 0.0
f1 (ppm)

S12-2

JD423.2.fid
Instrument AVF400
Group MDS
Chemist JD
Project Grant Code other
c13acq_512.crl CDCl3 {C:\NMR} mdsgrp 4

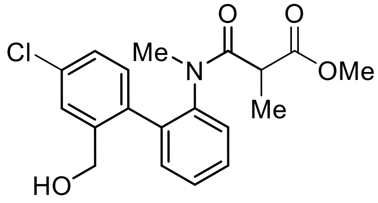

S12-2

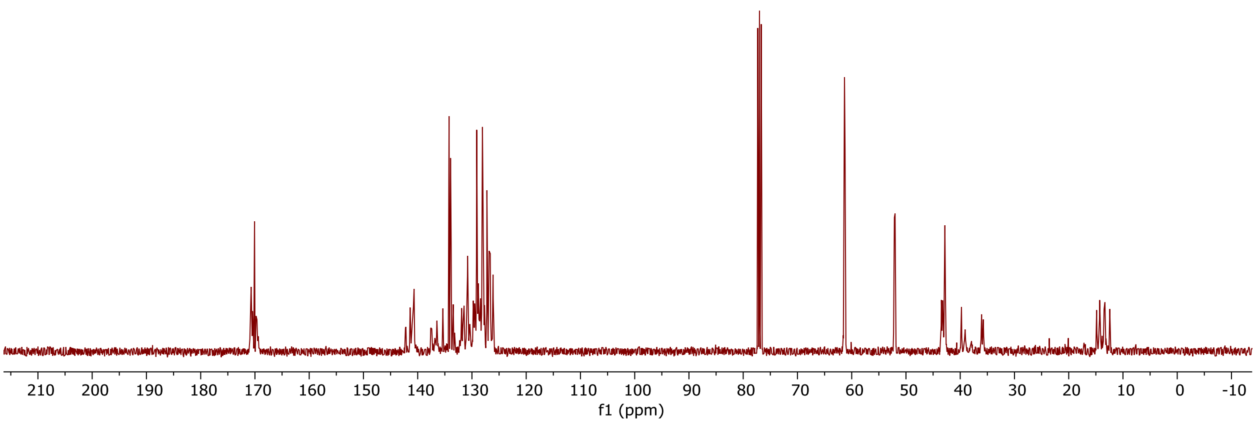

JD429.1.fid
Instrument AVF400
Group MDS
Chemist JD
Project Grant Code other
h1acq.crl CDCl3 {C:\NMR} mdsgrp 23

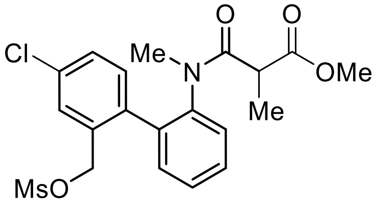

S12

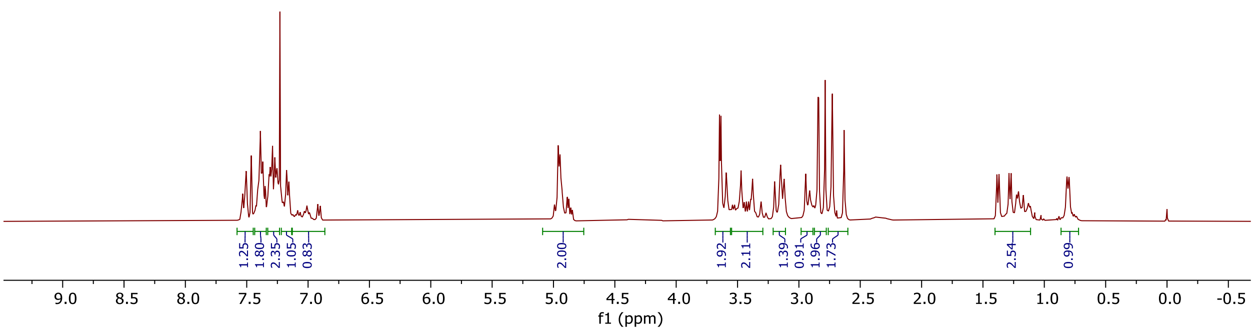

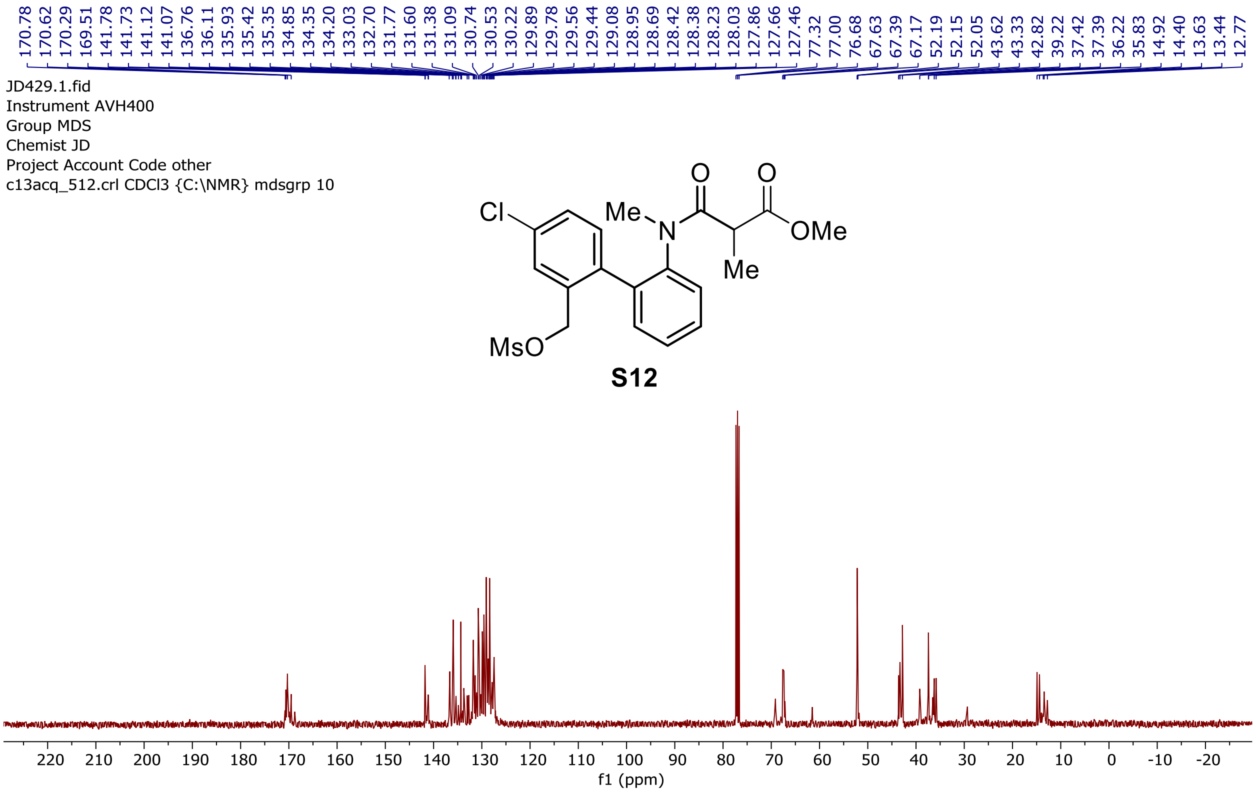
170.78 170.62 170.29 169.51 141.78 141.73 141.12 141.07 136.76 136.11 135.93 135.42 135.35 134.85 134.35 134.20 133.03 132.70 131.77 131.60 131.38 131.09 130.74 130.53 130.22 129.89 129.78 129.56 129.44 129.08 128.95 128.69 128.42 128.38 128.23 128.03 127.86 127.66 127.46 77.32 77.00 76.68 67.63 67.39 67.17 52.19 52.15 52.05 43.62 43.33 42.82 39.22 37.42 37.39 36.22 35.83 14.92 14.40 13.63 13.44 12.77
JD429.1.fid
Instrument AVH400
Group MDS
Chemist JD
Project Account Code other
c13acq_512.crl CDCl3 {C:\NMR} mdsgrp 10
Cl
Me
N
O
O
OMe
Me
MsO
S12
f1 (ppm)

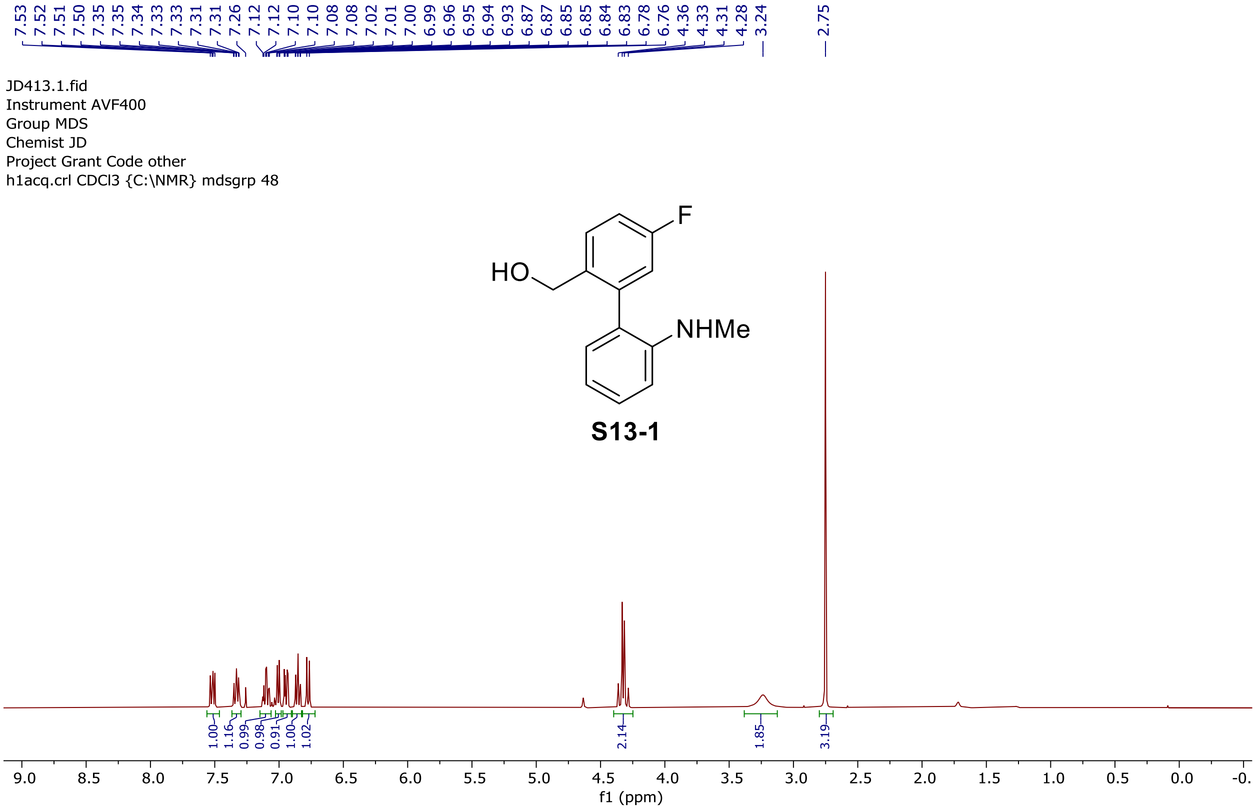
7.53 7.52 7.51 7.50 7.35 7.35 7.34 7.33 7.33 7.31 7.31 7.26 7.12 7.12 7.10 7.10 7.08 7.08 7.02 7.01 7.00 6.99 6.96 6.95 6.94 6.93 6.87 6.87 6.85 6.85 6.84 6.83 6.78 6.76 4.36 4.33 4.31 4.28 3.24 2.75
JD413.1.fid
Instrument AVF400
Group MDS
Chemist JD
Project Grant Code other
h1acq.crl CDCl3 {C:\NMR} mdsgrp 48
F
HO
NHMe
S13-1
1.00 1.16 0.99 0.98 0.91 1.00 1.02 2.14 1.85 3.19
f1 (ppm)

JD413.2.fid
Instrument AVF400
Group MDS
Chemist JD
Project Grant Code other
c13acq_512.crl CDCl3 {C:\NMR} mdsgrp 48

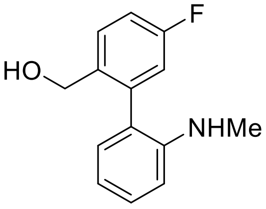

S13-1

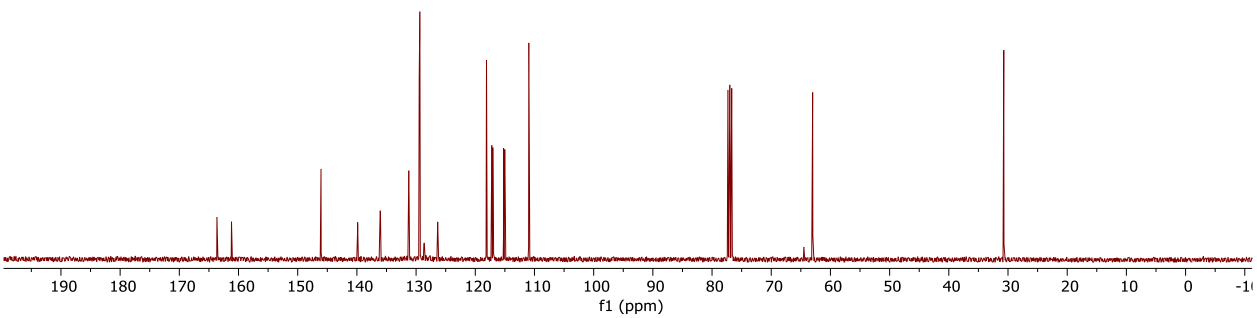

JD413.3.fid
Instrument AVF400
Group MDS
Chemist JD
Project Grant Code other
f19acq.crl CDCl3 {C:\NMR} mdsgrp 48

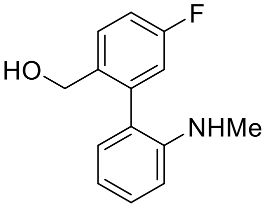

S13-1

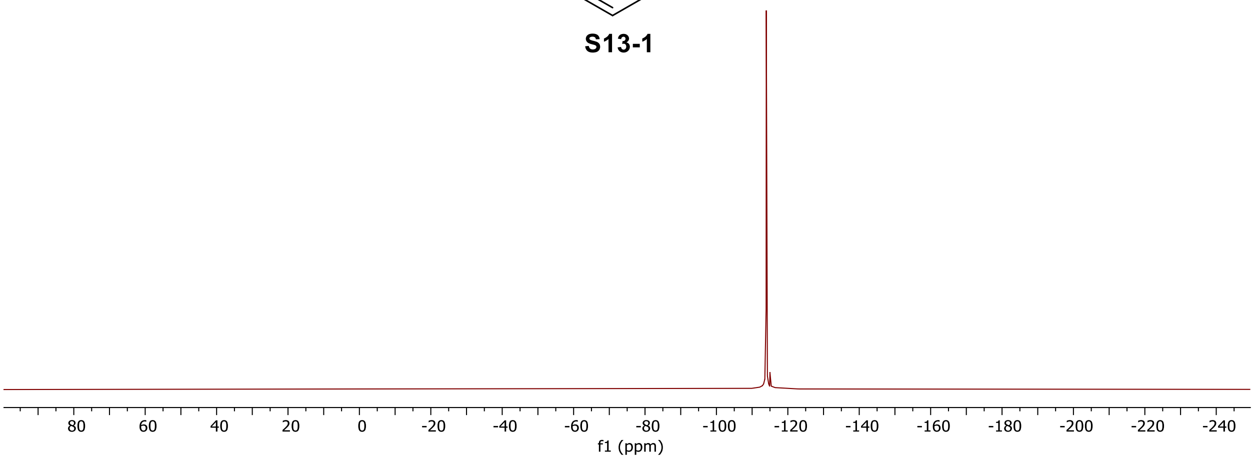

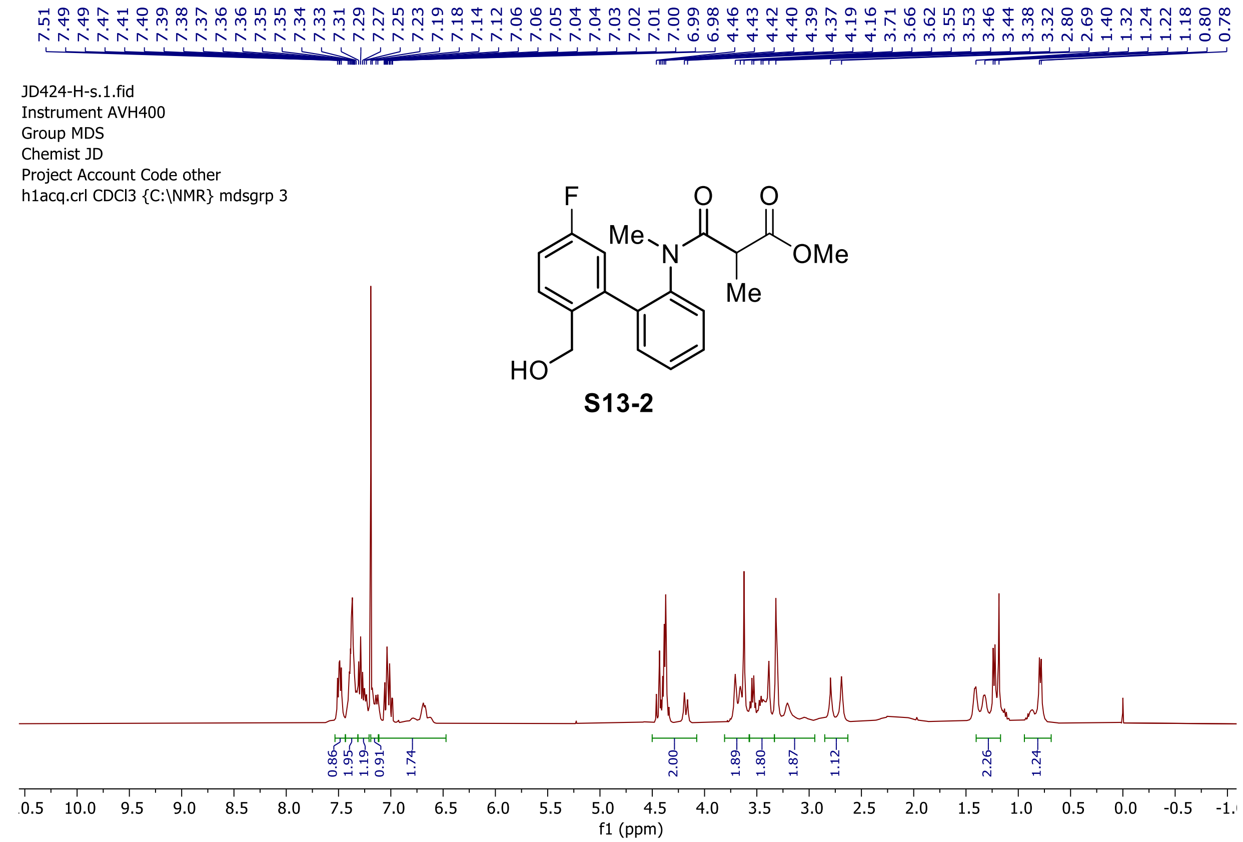
7.51 7.49 7.47 7.41 7.40 7.39 7.38 7.37 7.36 7.35 7.35 7.34 7.33 7.31 7.29 7.27 7.25 7.23 7.19 7.18 7.14 7.12 7.06 7.06 7.05 7.04 7.03 7.02 7.01 7.00 6.99 6.98 4.46 4.43 4.42 4.40 4.39 4.37 4.19 4.16 3.71 3.66 3.62 3.55 3.53 3.46 3.44 3.38 3.32 2.80 2.69 1.40 1.32 1.24 1.22 1.18 0.80 0.78
JD424-H-s.1.fid
Instrument AVH400
Group MDS
Chemist JD
Project Account Code other
h1acq.crl CDCl3 {C:\NMR} mdsgrp 3
F
Me
N
O
O
OMe
Me
HO
S13-2
0.86 1.95 1.19 0.91 1.74 2.00 1.89 1.80 1.87 1.12 2.26 1.24
0.5 10.0 9.5 9.0 8.5 8.0 7.5 7.0 6.5 6.0 5.5 5.0 4.5 4.0 3.5 3.0 2.5 2.0 1.5 1.0 0.5 0.0 -0.5
f1 (ppm)

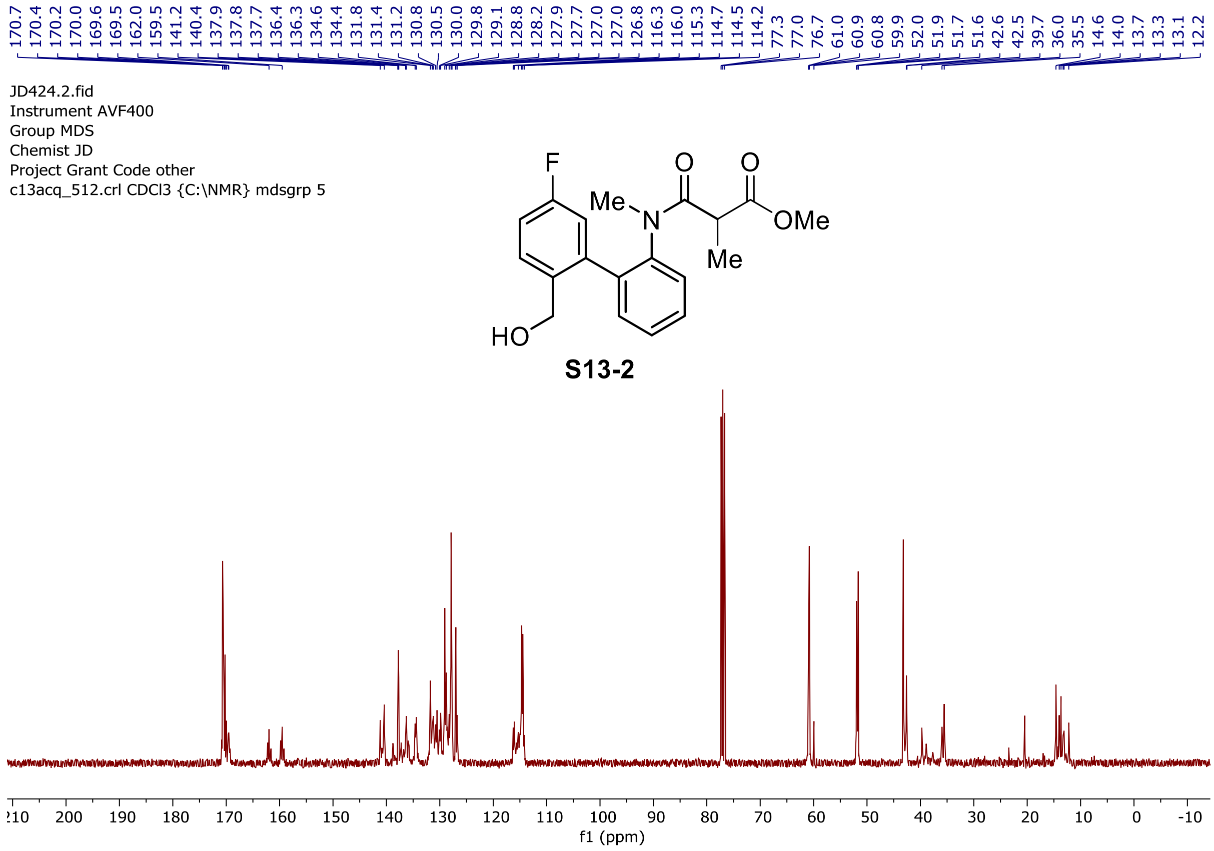
170.7 170.4 170.2 170.0 169.6 169.5 162.0 159.5 141.2 140.4 137.9 137.8 137.7 136.4 136.3 134.6 134.4 131.8 131.4 131.2 130.8 130.5 130.0 129.8 129.1 128.8 128.2 127.9 127.7 127.0 127.0 126.8 116.3 116.0 115.3 114.7 114.5 114.2 77.3 77.0 76.7 61.0 60.9 60.8 59.9 52.0 51.9 51.7 51.6 42.6 42.5 39.7 36.0 35.5 14.6 14.0 13.7 13.3 13.1 12.2
JD424.2.fid
Instrument AVF400
Group MDS
Chemist JD
Project Grant Code other
c13acq_512.crl CDCl3 {C:\NMR} mdsgrp 5
F
Me
N
O
O
OMe
Me
HO
S13-2
210 200 190 180 170 160 150 140 130 120 110 100 90 80 70 60 50 40 30 20 10 0 -10
f1 (ppm)

-114.73
-115.11
-115.53
-115.55
-115.65
-115.91
-115.93

JD424.3.fid
Instrument AVF400
Group MDS
Chemist JD
Project Grant Code other
f19acq.crl CDCl3 {C:\NMR} mdsgrp 5

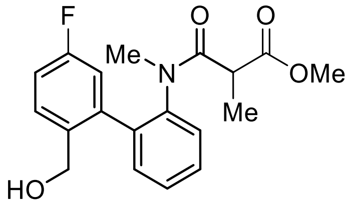

S13-2

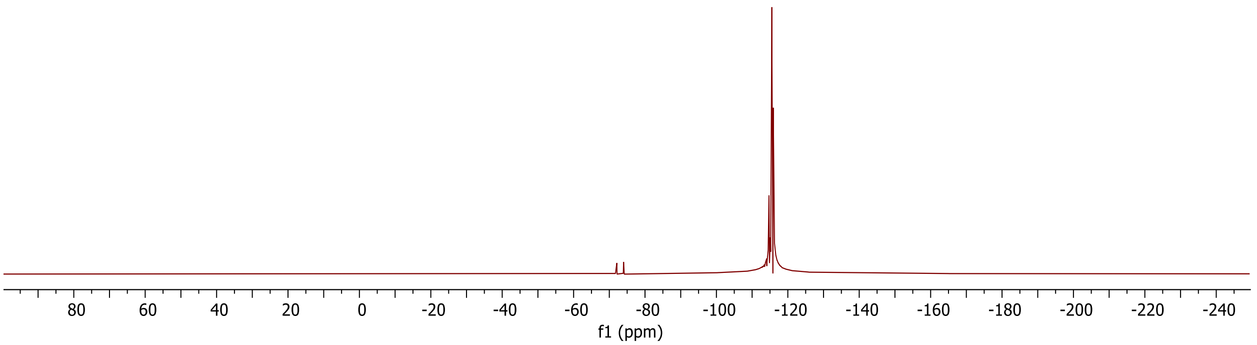

7.53, 7.51, 7.51, 7.49, 7.49, 7.48, 7.46, 7.43, 7.41, 7.40, 7.38, 7.36, 7.34, 7.32, 7.31, 7.24, 7.22, 7.20, 7.19, 7.12, 7.11, 7.10, 7.09, 7.09, 7.07, 7.07, 7.06, 7.05, 7.05, 5.06, 5.03, 5.02, 4.99, 4.96, 4.87, 4.84, 3.71, 3.67, 3.63, 3.55, 3.54, 3.52, 3.43, 3.42, 3.23, 3.17, 2.94, 2.91, 2.85, 2.83, 2.78, 2.77, 2.68, 1.43, 1.41, 1.35, 1.34, 1.25, 1.19, 0.86, 0.85

JD430-H-s.1.fid
Instrument AVH400
Group MDS
Chemist JD
Project Account Code other
h1acq.crl CDCl3 {C:\NMR} mdsgrp 5

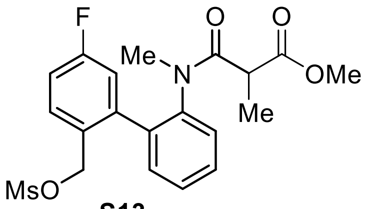

S13

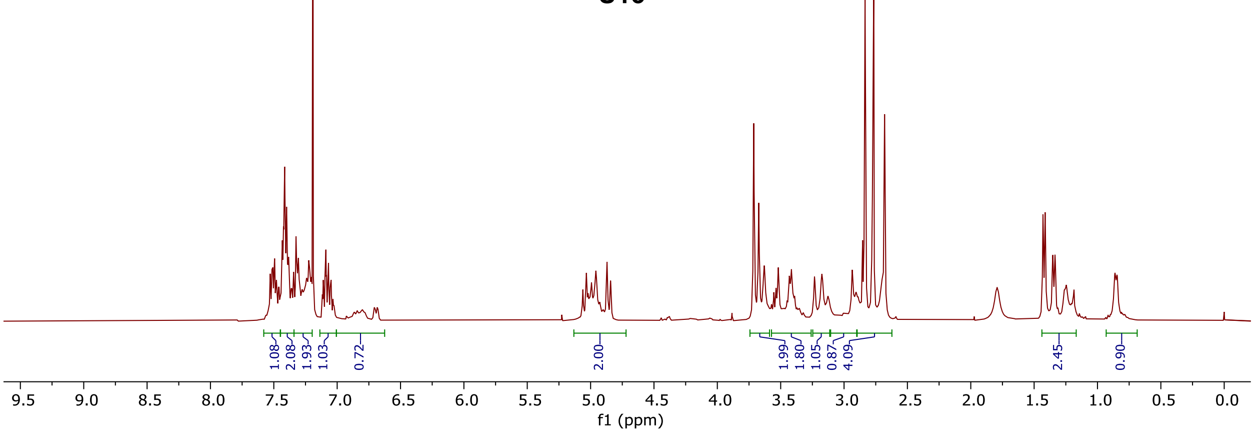

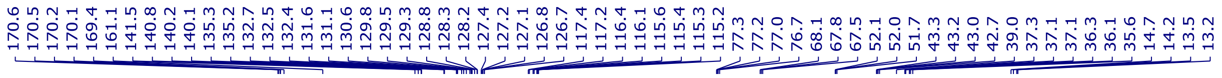

JD430.1.fid
Instrument AVH400
Group MDS
Chemist JD
Project Account Code other
c13acq_512.crl CDCl3 {C:\NMR} mdsgrp 11

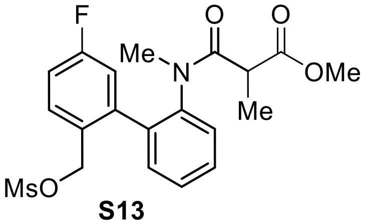

S13

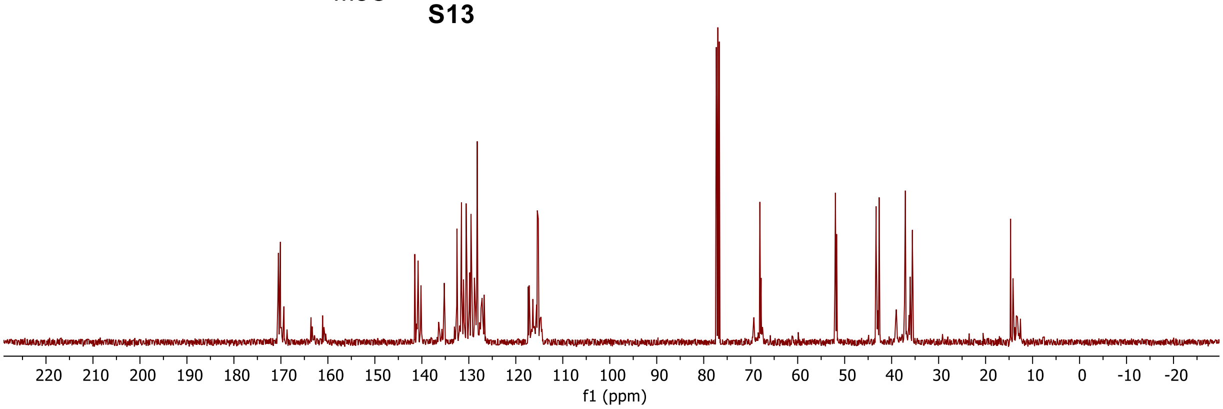

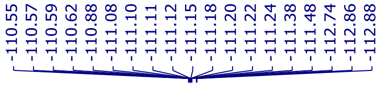

JD430.3.fid
Instrument AVF400
Group MDS
Chemist JD
Project Grant Code other
f19acq.crl CDCl3 {C:\NMR} mdsgrp 24

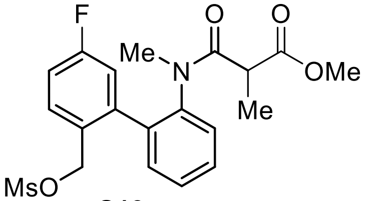

S13

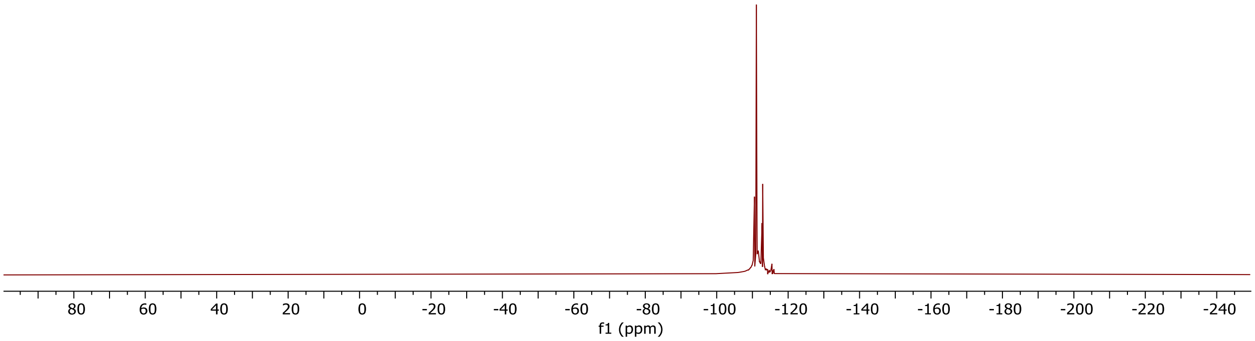

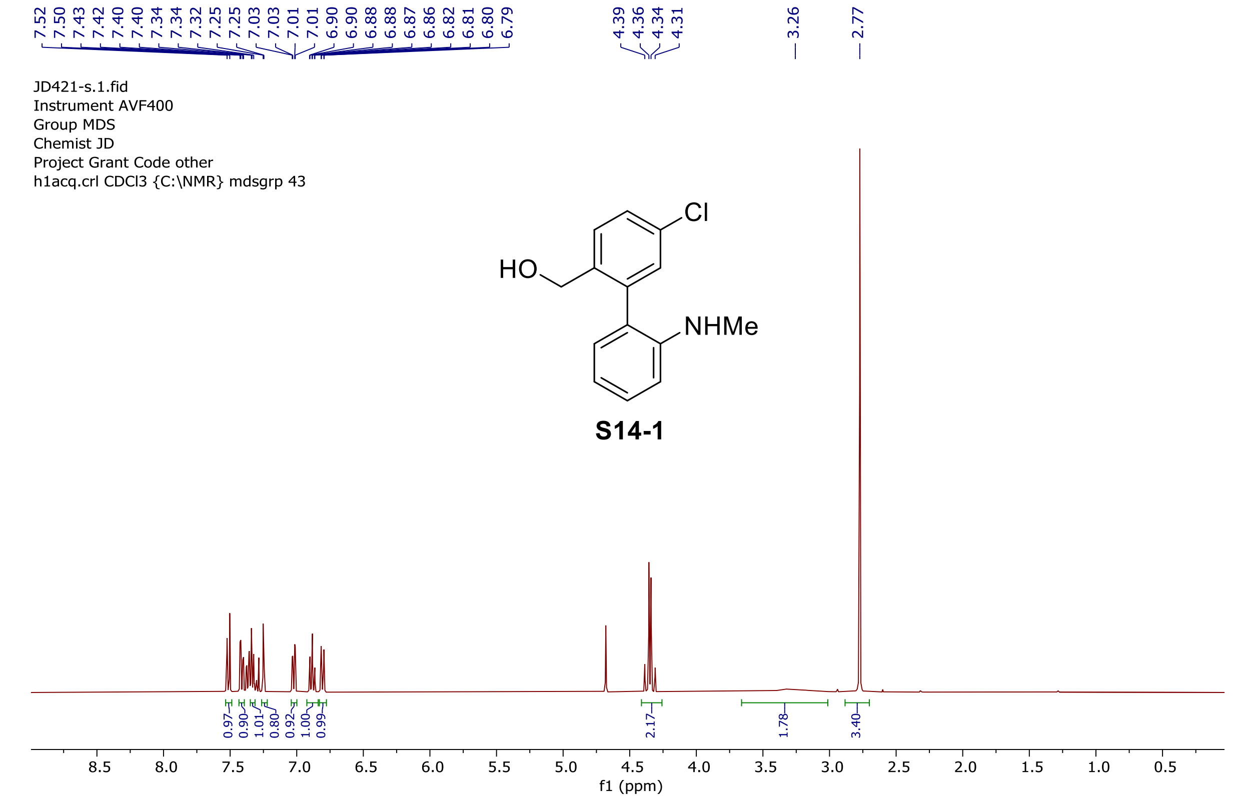
7.52 7.50 7.43 7.42 7.40 7.40 7.34 7.34 7.32 7.25 7.03 7.03 7.01 7.01 6.90 6.90 6.88 6.88 6.87 6.86 6.82 6.81 6.80 6.79
4.39 4.36 4.34 4.31
3.26
2.77
JD421-s.1.fid
Instrument AVF400
Group MDS
Chemist JD
Project Grant Code other
h1acq.crl CDCl3 {C:\NMR} mdsgrp 43
Cl
HO
NHMe
S14-1
0.97 0.90 1.01 0.80 0.92 1.00 0.99
2.17
1.78
3.40
8.5 8.0 7.5 7.0 6.5 6.0 5.5 5.0 4.5 4.0 3.5 3.0 2.5 2.0 1.5 1.0 0.5
f1 (ppm)

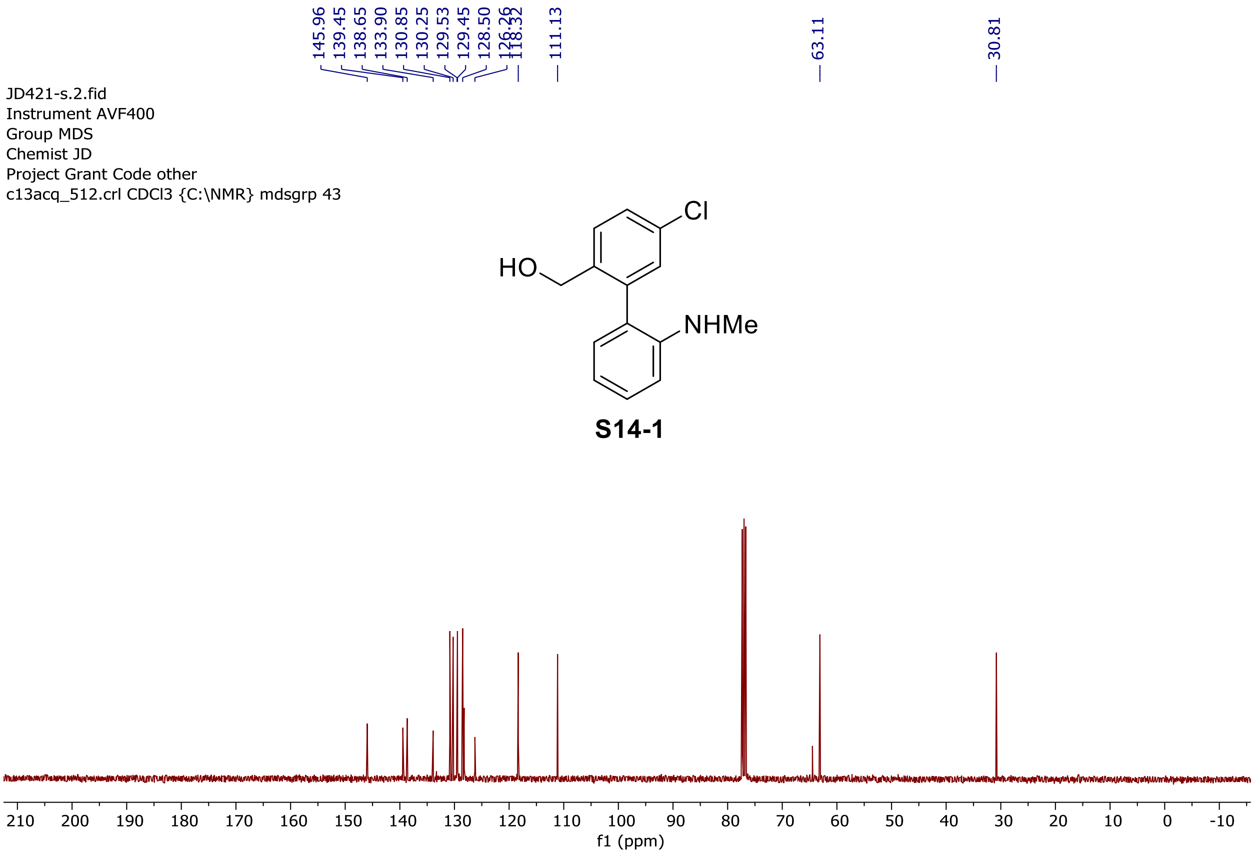
145.96 139.45 138.65 133.90 130.85 130.25 129.53 129.45 128.50 126.26 118.32
111.13
63.11
30.81
JD421-s.2.fid
Instrument AVF400
Group MDS
Chemist JD
Project Grant Code other
c13acq_512.crl CDCl3 {C:\NMR} mdsgrp 43
Cl
HO
NHMe
S14-1
210 200 190 180 170 160 150 140 130 120 110 100 90 80 70 60 50 40 30 20 10 0 -10
f1 (ppm)

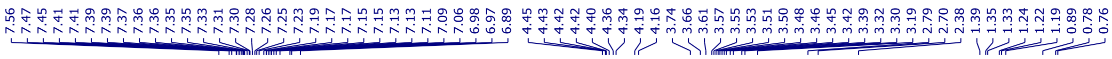

JD434-H-s.1.fid
Instrument AVH400
Group MDS
Chemist JD
Project Account Code other
h1acq.crl CDCl3 {C:\NMR} mdsgrp 4

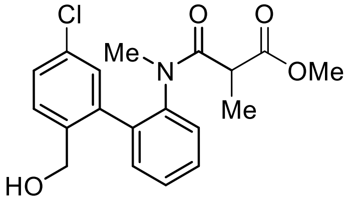

S14-2

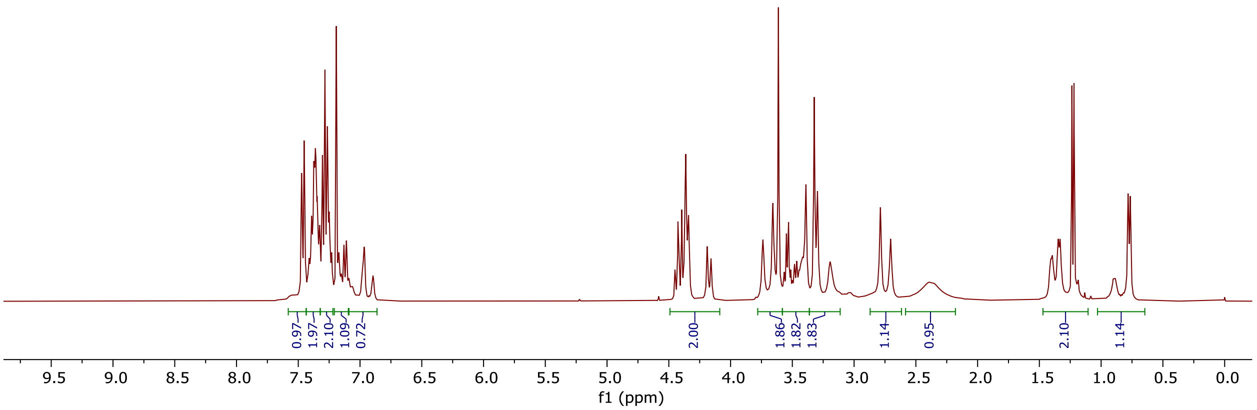

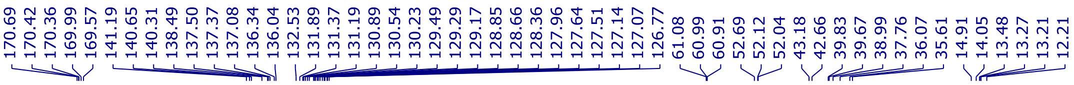

JD434.2.fid
Instrument AVH400
Group MDS
Chemist JD
Project Account Code other
c13acq_512.crl CDCl3 {C:\NMR} mdsgrp 7

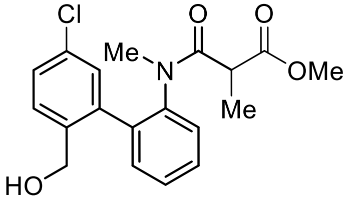

S14-2

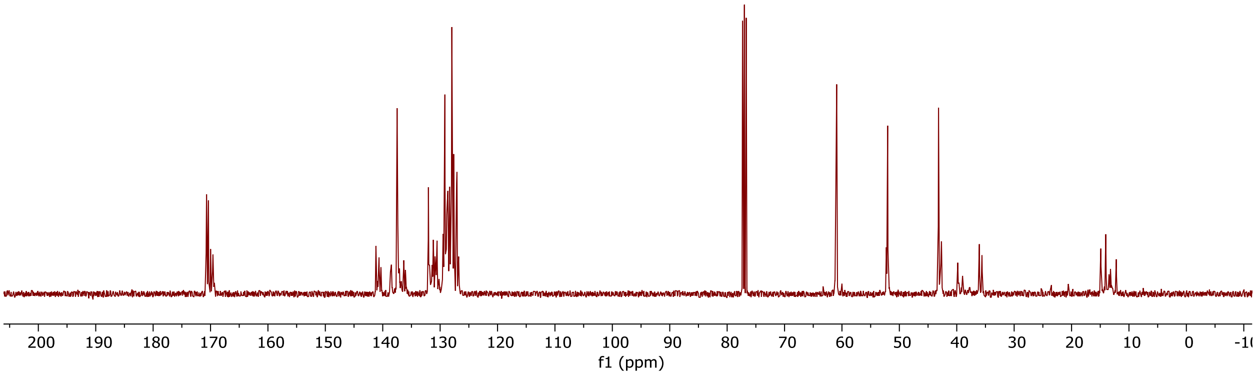

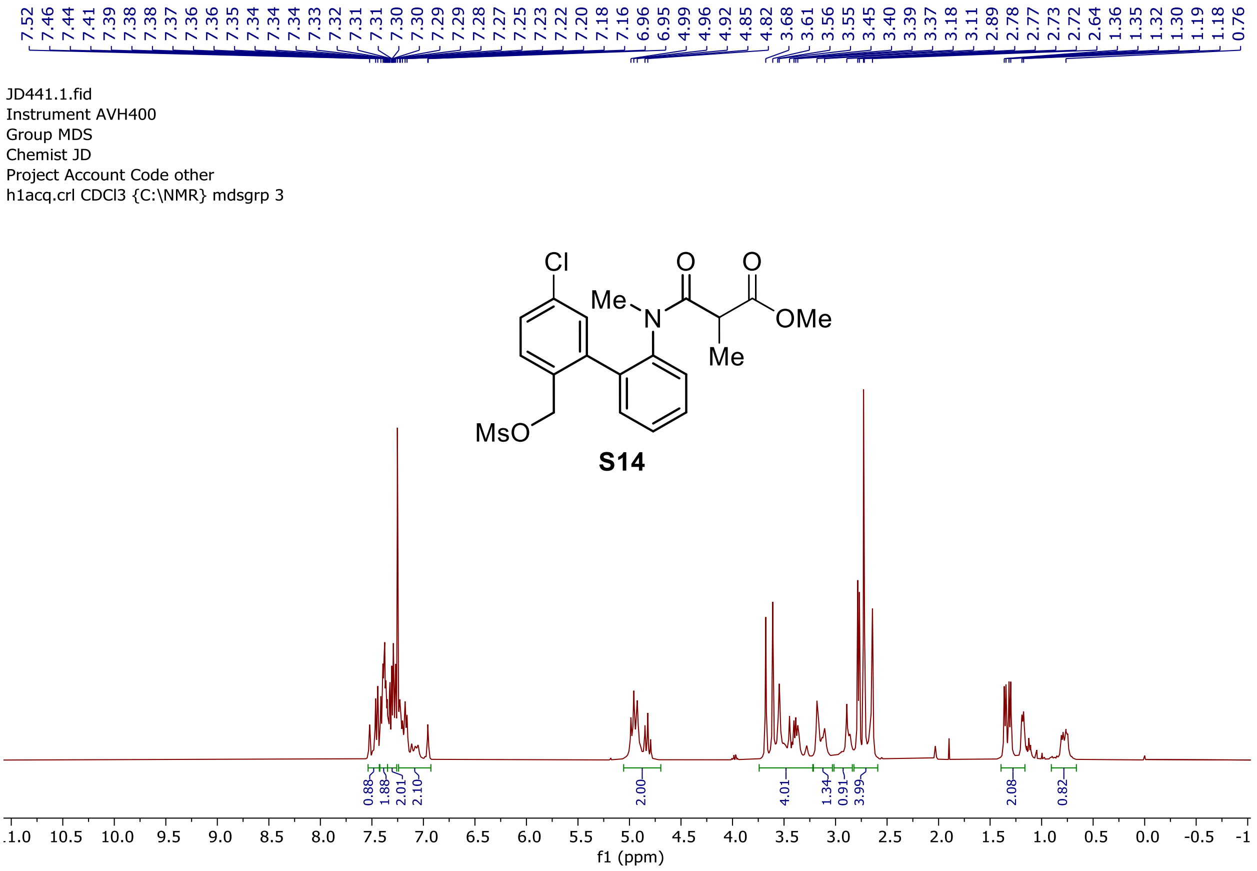
7.52 7.46 7.44 7.41 7.39 7.38 7.38 7.37 7.36 7.36 7.35 7.34 7.34 7.34 7.33 7.32 7.31 7.31 7.30 7.30 7.29 7.29 7.28 7.27 7.25 7.23 7.22 7.20 7.18 7.16 6.96 6.95 4.99 4.96 4.92 4.85 4.82 3.68 3.61 3.56 3.55 3.45 3.40 3.39 3.37 3.18 3.11 2.89 2.78 2.77 2.73 2.72 2.64 1.36 1.35 1.32 1.30 1.19 1.18 0.76
JD441.1.fid
Instrument AVH400
Group MDS
Chemist JD
Project Account Code other
h1acq.crl CDCl3 {C:\NMR} mdsgrp 3
Cl
O
O
Me
N
OMe
Me
MsO
S14
0.88 1.88 2.01 2.10 2.00 4.01 1.34 0.91 3.99 2.08 0.82
f1 (ppm)

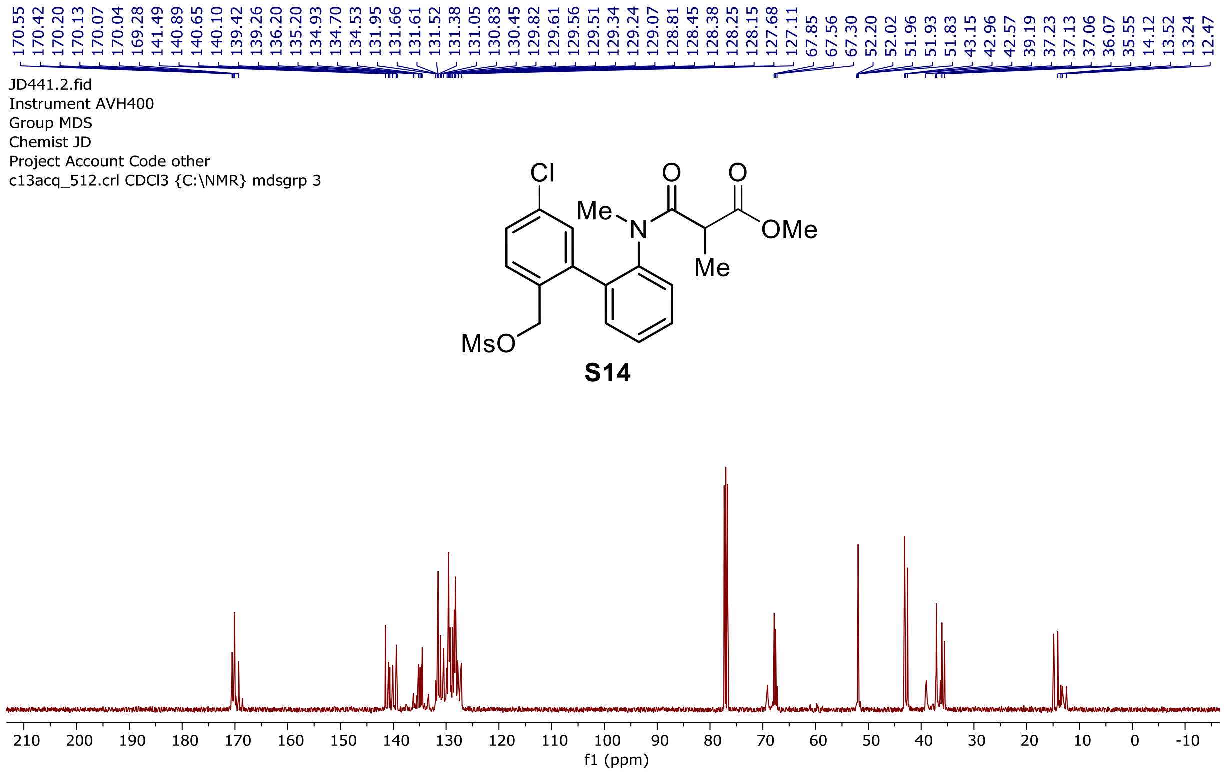
170.55 170.42 170.20 170.13 170.07 170.04 169.28 141.49 140.89 140.65 140.10 139.42 139.26 136.20 135.20 134.93 134.70 134.53 131.95 131.66 131.61 131.52 131.38 131.05 130.83 130.45 129.82 129.61 129.56 129.51 129.34 129.24 129.07 128.81 128.45 128.38 128.25 128.15 127.68 127.11 67.85 67.56 67.30 52.20 52.02 51.96 51.93 51.83 43.15 42.96 42.57 39.19 37.23 37.13 37.06 36.07 35.55 14.12 13.52 13.24 12.47
JD441.2.fid
Instrument AVH400
Group MDS
Chemist JD
Project Account Code other
c13acq_512.crl CDCl3 {C:\NMR} mdsgrp 3
Cl
O
O
Me
N
OMe
Me
MsO
S14
f1 (ppm)

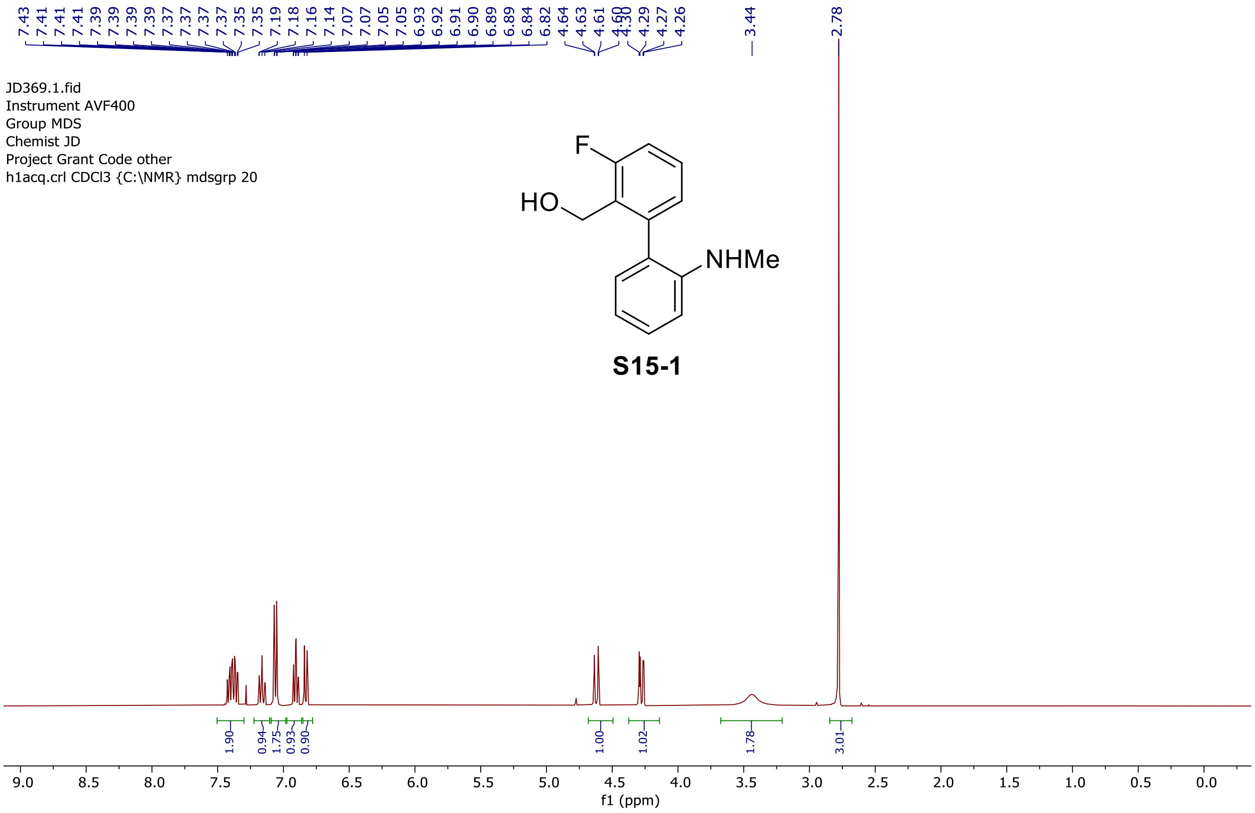
7.43 7.41 7.41 7.39 7.39 7.39 7.37 7.37 7.37 7.37 7.35 7.35 7.19 7.18 7.16 7.14 7.07 7.05 7.05 6.93 6.92 6.91 6.90 6.89 6.89 6.84 6.82 4.64 4.63 4.61 4.60 4.30 4.29 4.27 4.26
3.44
2.78
JD369.1.fid
Instrument AVF400
Group MDS
Chemist JD
Project Grant Code other
h1acq.crl CDCl3 {C:\NMR} mdsgrp 20
F
HO
NHMe
S15-1
1.90 0.94 1.75 0.93 0.90 1.00 1.02 1.78 3.01
9.0 8.5 8.0 7.5 7.0 6.5 6.0 5.5 5.0 4.5 4.0 3.5 3.0 2.5 2.0 1.5 1.0 0.5 0.0
f1 (ppm)

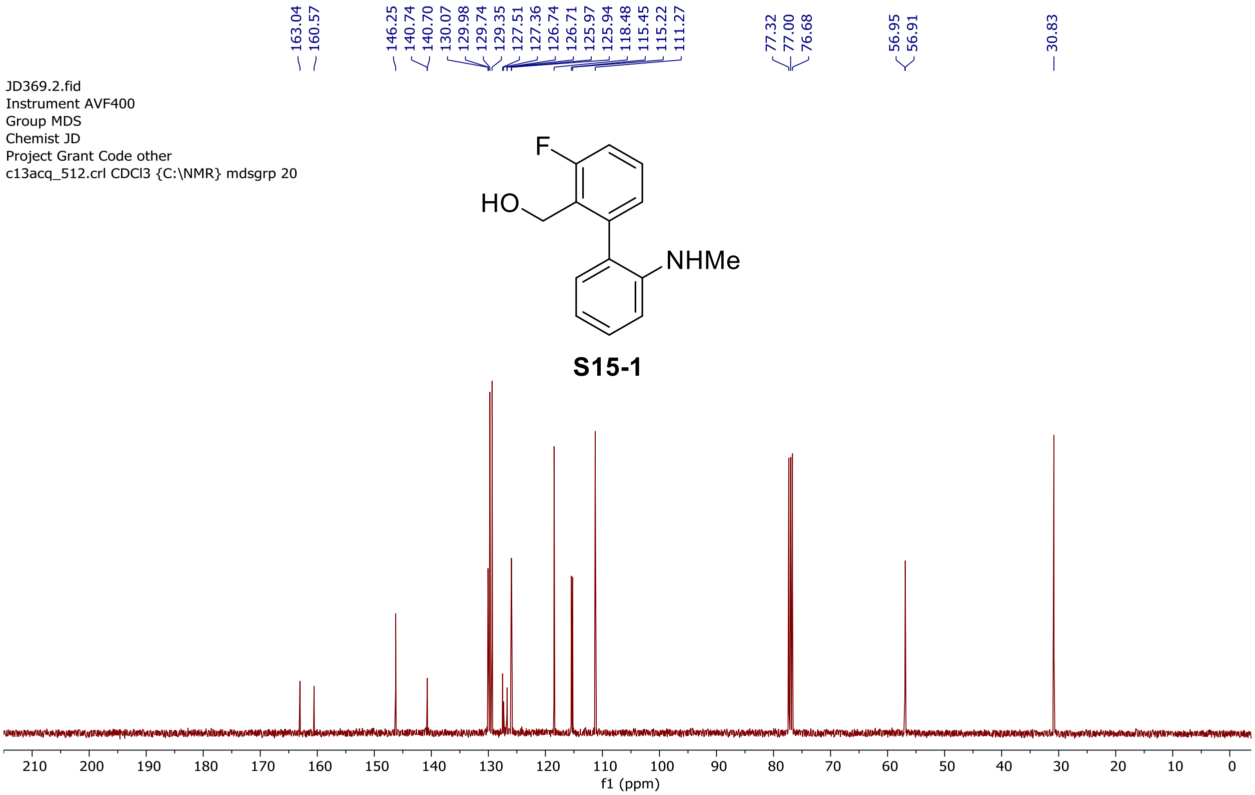
163.04 160.57
146.25 140.74 140.70 130.07 129.98 129.74 129.35 127.51 127.36 126.74 126.71 125.97 125.94 118.48 115.45 115.22 111.27
77.32 77.00 76.68
56.95 56.91
30.83
JD369.2.fid
Instrument AVF400
Group MDS
Chemist JD
Project Grant Code other
c13acq_512.crl CDCl3 {C:\NMR} mdsgrp 20
F
HO
NHMe
S15-1
210 200 190 180 170 160 150 140 130 120 110 100 90 80 70 60 50 40 30 20 10 0
f1 (ppm)

JD369.3.fid
Instrument AVF400
Group MDS
Chemist JD
Project Grant Code other
f19dec.crl CDCl3 {C:\NMR} mdsgrp 20

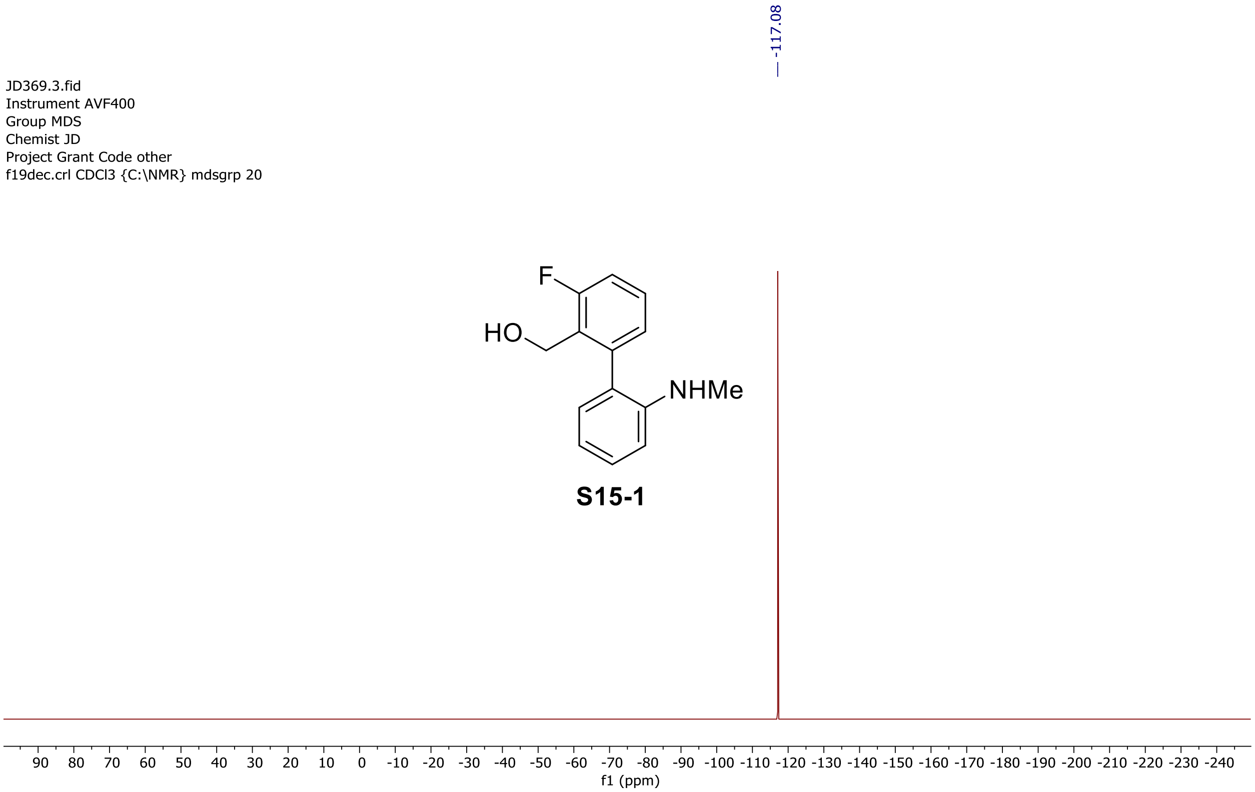

JD378.1.fid
Instrument AVF400
Group MDS
Chemist JD
Project Grant Code other
h1acq.crl CDCl3 {C:\NMR} mdsgrp 14

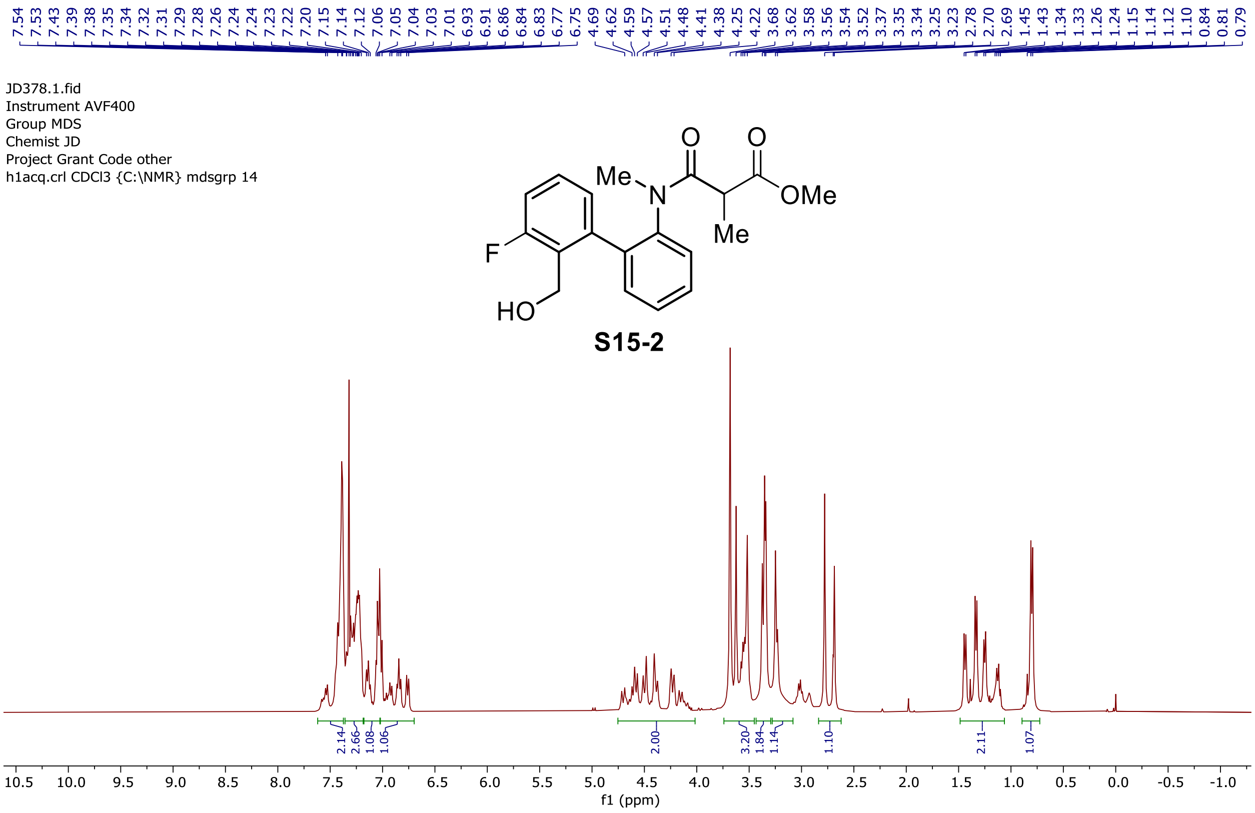

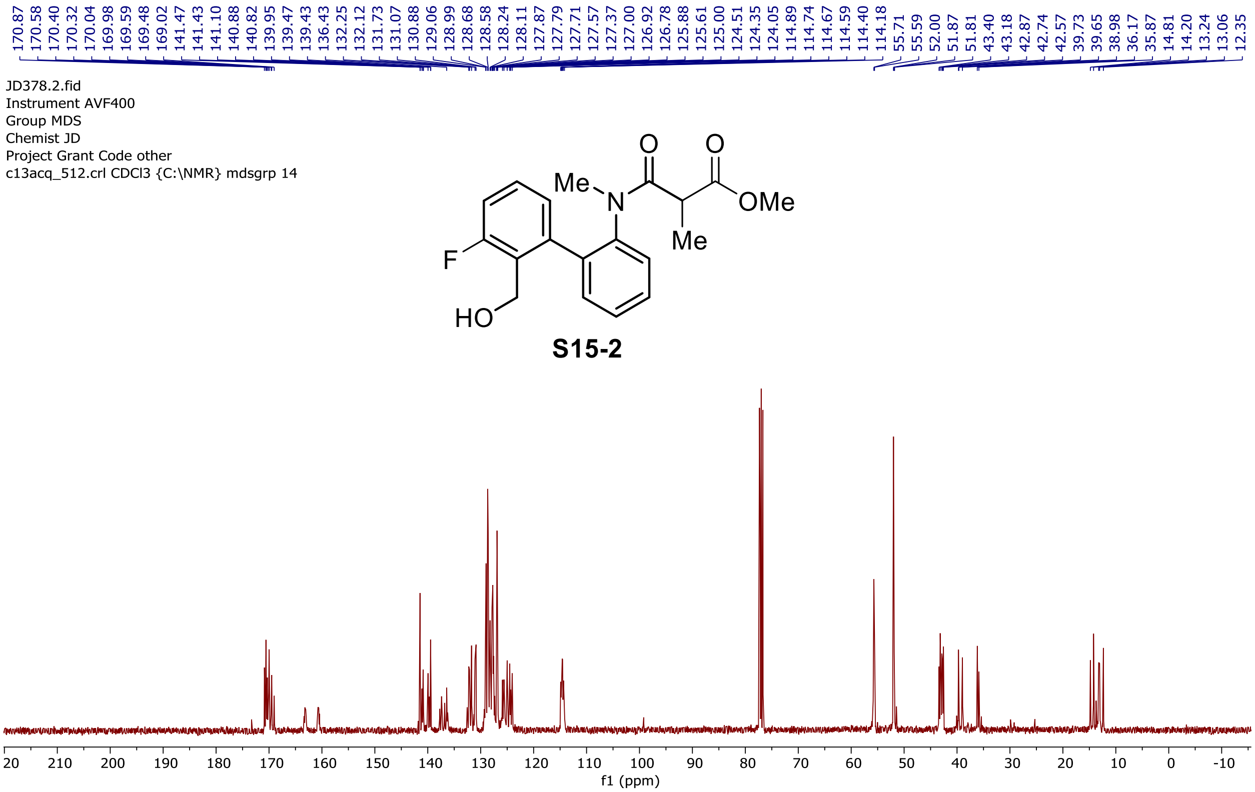
170.87 170.58 170.40 170.32 170.04 169.98 169.59 169.48 169.02 141.47 141.43 141.10 140.88 140.82 139.95 139.47 139.43 136.43 132.25 132.12 131.73 131.07 130.88 129.06 128.99 128.68 128.58 128.24 128.11 127.87 127.79 127.71 127.57 127.37 127.00 126.92 126.78 125.88 125.61 125.00 124.51 124.35 124.05 114.89 114.74 114.67 114.59 114.40 114.18 55.71 55.59 52.00 51.87 51.81 43.40 43.18 42.87 42.74 42.57 39.73 39.65 38.98 36.17 35.87 14.81 14.20 13.24 13.06 12.35
JD378.2.fid
Instrument AVF400
Group MDS
Chemist JD
Project Grant Code other
c13acq_512.crl CDCl3 {C:\NMR} mdsgrp 14
S15-2
f1 (ppm)

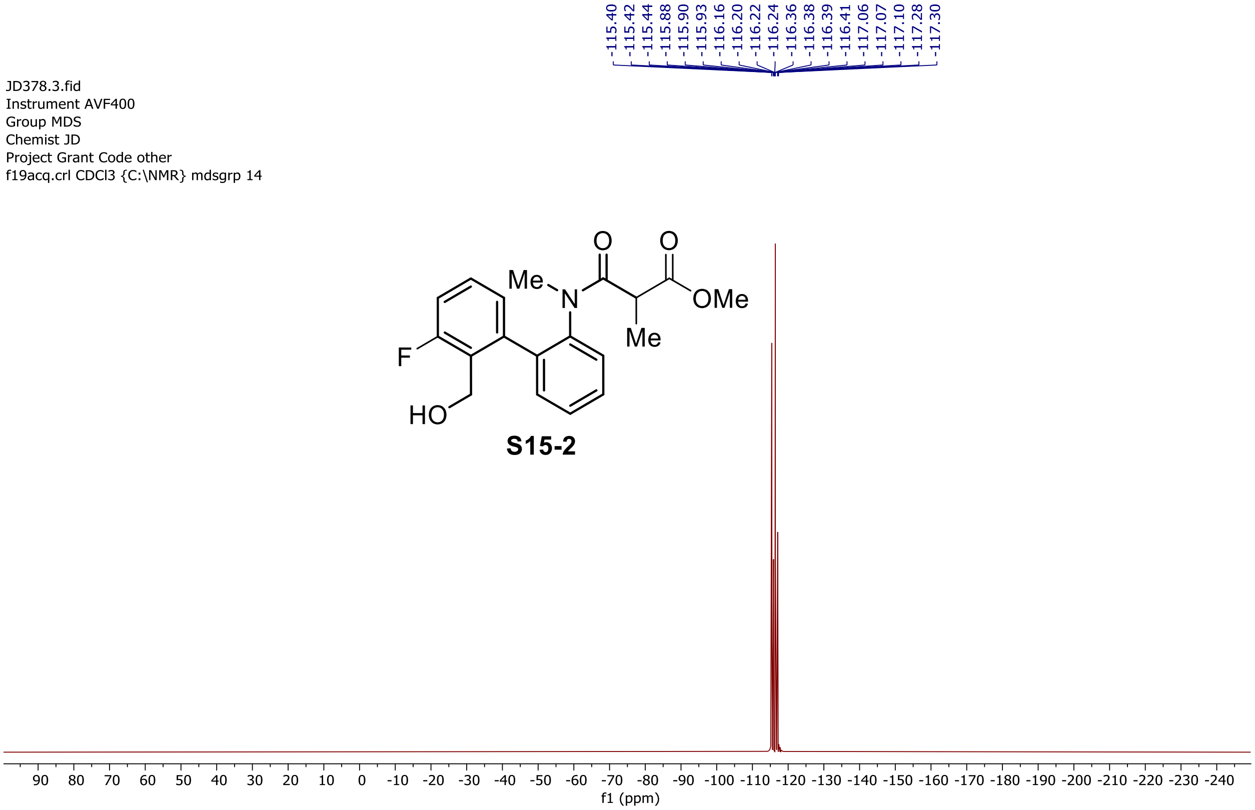
-115.40 -115.42 -115.44 -115.88 -115.90 -115.93 -116.16 -116.20 -116.22 -116.24 -116.36 -116.38 -116.39 -116.41 -117.06 -117.07 -117.10 -117.28 -117.30
JD378.3.fid
Instrument AVF400
Group MDS
Chemist JD
Project Grant Code other
f19acq.crl CDCl3 {C:\NMR} mdsgrp 14
S15-2
f1 (ppm)

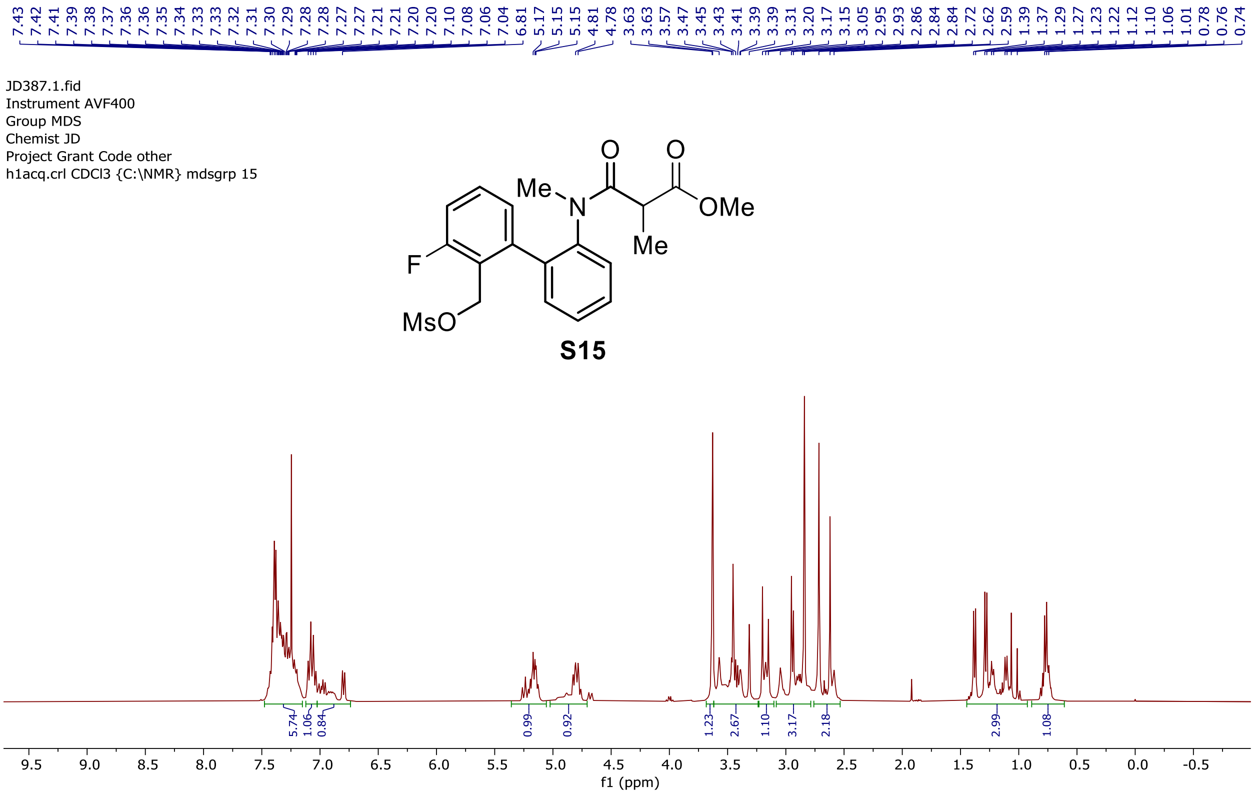
JD387.1.fid
Instrument AVF400
Group MDS
Chemist JD
Project Grant Code other
h1acq.crl CDCl3 {C:\NMR} mdsgrp 15
S15
f1 (ppm)

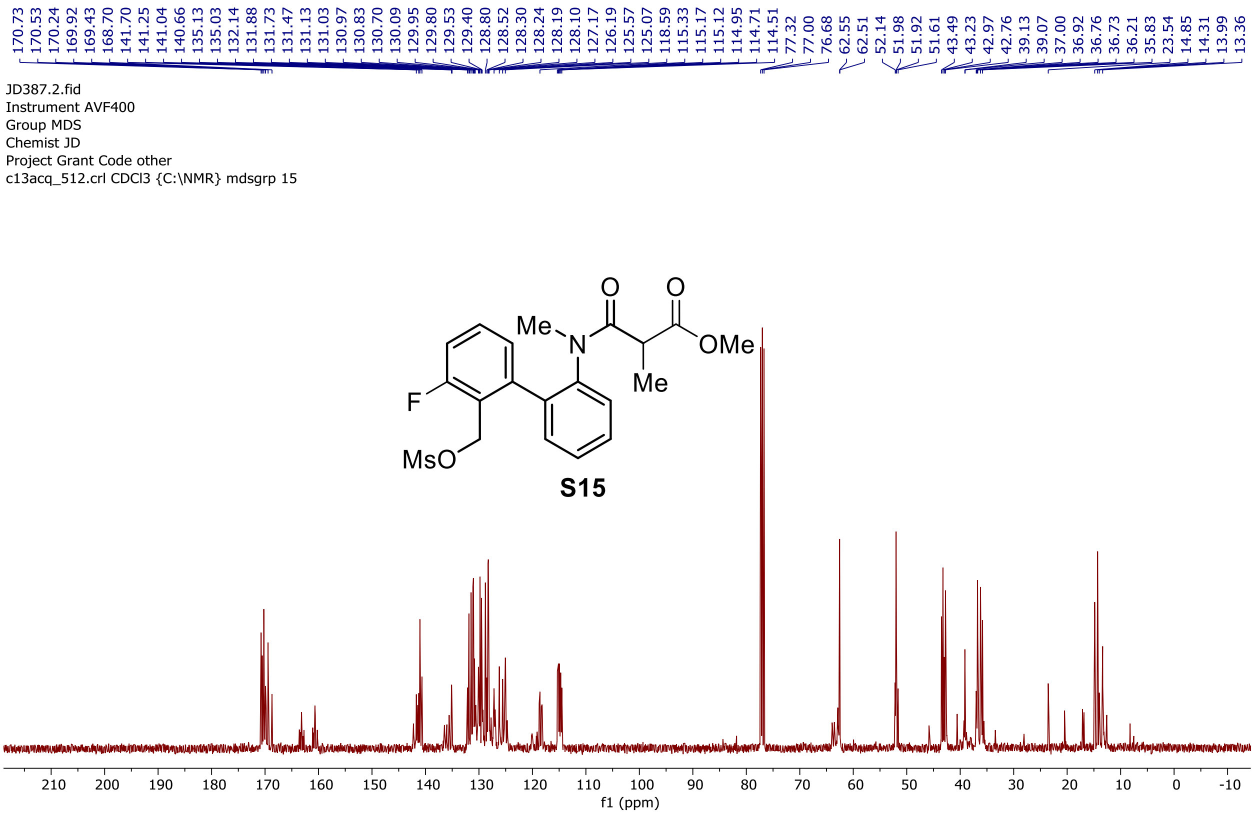
JD387.2.fid
Instrument AVF400
Group MDS
Chemist JD
Project Grant Code other
c13acq_512.crl CDCl3 {C:\NMR} mdsgrp 15
S15
f1 (ppm)

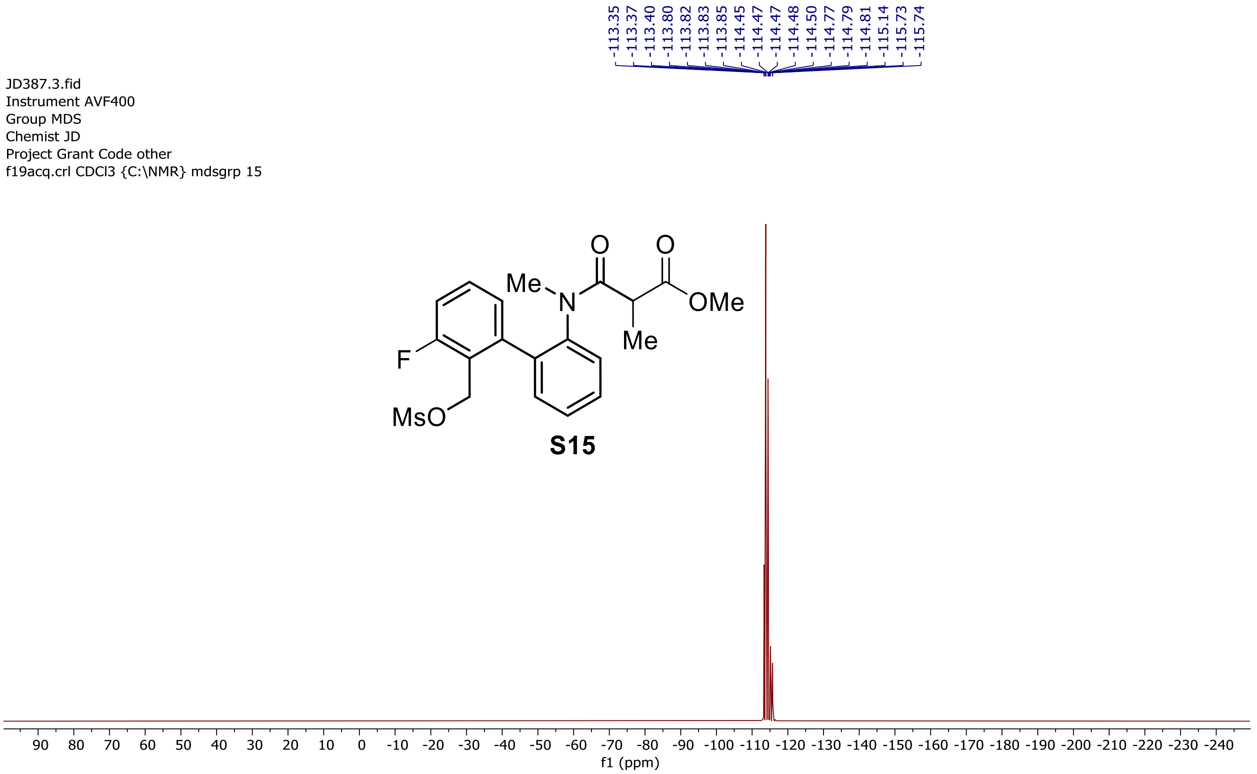
-113.35
-113.37
-113.40
-113.80
-113.82
-113.83
-113.85
-114.45
-114.47
-114.47
-114.48
-114.50
-114.77
-114.79
-114.81
-115.14
-115.73
-115.74
JD387.3.fid
Instrument AVF400
Group MDS
Chemist JD
Project Grant Code other
f19acq.crl CDCl3 {C:\NMR} mdsgrp 15
O
O
Me
N
OMe
Me
F
MsO
S15
90 80 70 60 50 40 30 20 10 0 -10 -20 -30 -40 -50 -60 -70 -80 -90 -100 -110 -120 -130 -140 -150 -160 -170 -180 -190 -200 -210 -220 -230 -240
f1 (ppm)

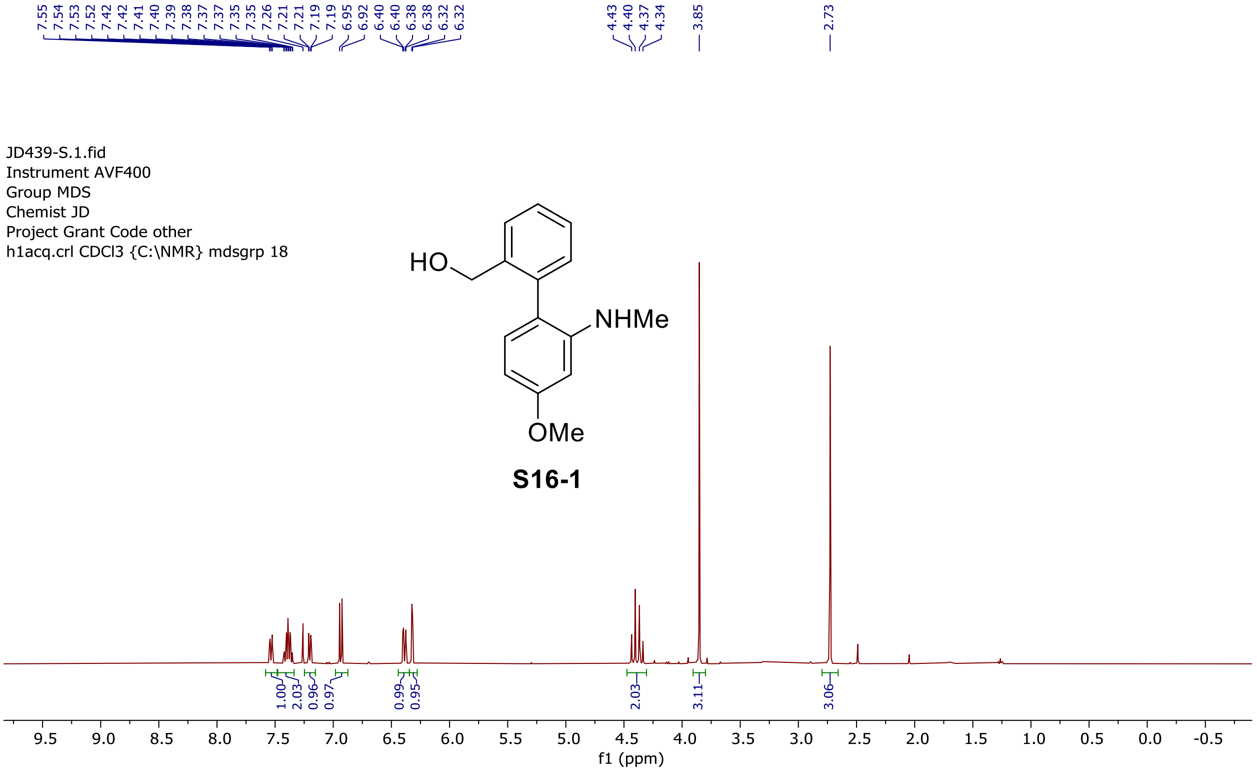
7.55
7.54
7.53
7.52
7.42
7.42
7.41
7.40
7.39
7.38
7.37
7.37
7.35
7.35
7.26
7.21
7.21
7.19
7.19
6.95
6.92
6.40
6.40
6.38
6.38
6.32
6.32
4.43
4.40
4.37
4.34
3.85
2.73
JD439-S.1.fid
Instrument AVF400
Group MDS
Chemist JD
Project Grant Code other
h1acq.crl CDCl3 {C:\NMR} mdsgrp 18
HO
NHMe
OMe
S16-1
1.00
2.03
0.96
0.97
0.99
0.95
2.03
3.11
3.06
9.5 9.0 8.5 8.0 7.5 7.0 6.5 6.0 5.5 5.0 4.5 4.0 3.5 3.0 2.5 2.0 1.5 1.0 0.5 0.0 -0.5
f1 (ppm)

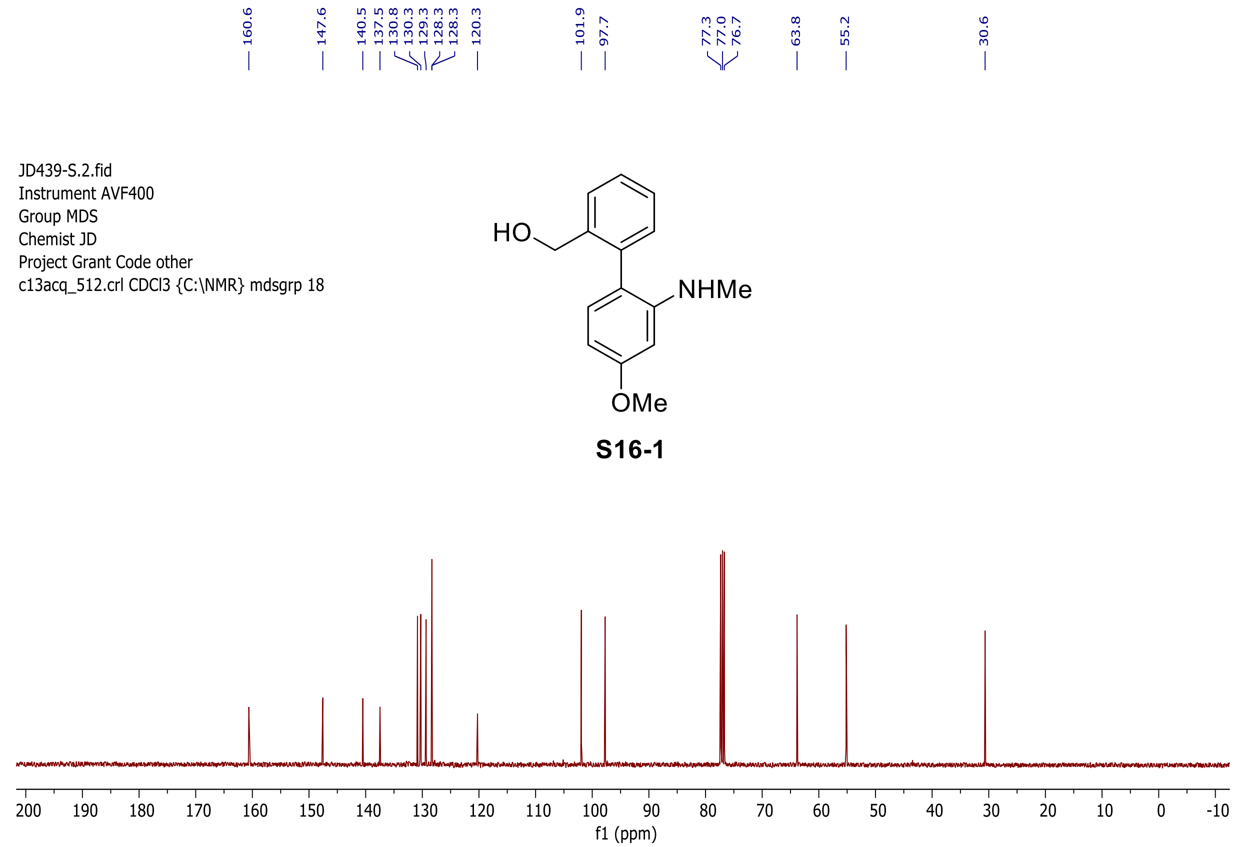
160.6
147.6
140.5
137.5
130.8
130.3
129.3
128.3
128.3
120.3
101.9
97.7
77.3
77.0
76.7
63.8
55.2
30.6
JD439-S.2.fid
Instrument AVF400
Group MDS
Chemist JD
Project Grant Code other
c13acq_512.crl CDCl3 {C:\NMR} mdsgrp 18
HO
NHMe
OMe
S16-1
200 190 180 170 160 150 140 130 120 110 100 90 80 70 60 50 40 30 20 10 0 -10
f1 (ppm)

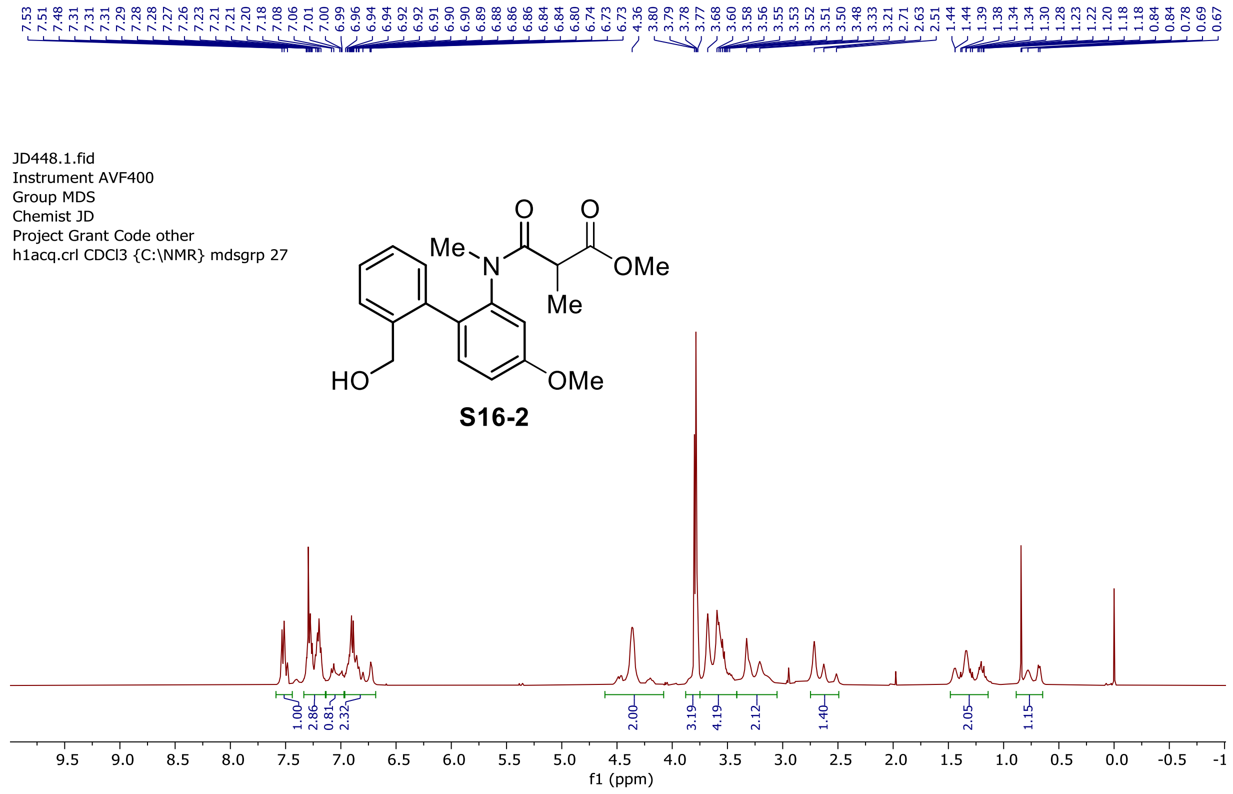
7.53 7.51 7.48 7.31 7.31 7.31 7.29 7.28 7.28 7.27 7.26 7.23 7.21 7.20 7.18 7.08 7.06 7.01 7.00 6.99 6.96 6.94 6.94 6.92 6.92 6.91 6.90 6.90 6.89 6.88 6.86 6.86 6.84 6.80 6.74 6.73 4.36 3.80 3.79 3.78 3.77 3.68 3.60 3.58 3.56 3.55 3.53 3.52 3.51 3.50 3.48 3.33 3.21 2.71 2.63 2.51 1.44 1.44 1.39 1.38 1.34 1.34 1.30 1.28 1.23 1.22 1.20 1.18 1.18 0.84 0.84 0.78 0.69 0.67
JD448.1.fid
Instrument AVF400
Group MDS
Chemist JD
Project Grant Code other
h1acq.crl CDCl3 {C:\NMR} mdsgrp 27
O
O
Me
N
OMe
Me
HO
OMe
S16-2
1.00 2.86 0.81 2.32 2.00 3.19 4.19 2.12 1.40 2.05 1.15
9.5 9.0 8.5 8.0 7.5 7.0 6.5 6.0 5.5 5.0 4.5 4.0 3.5 3.0 2.5 2.0 1.5 1.0 0.5 0.0 -0.5 -1
f1 (ppm)

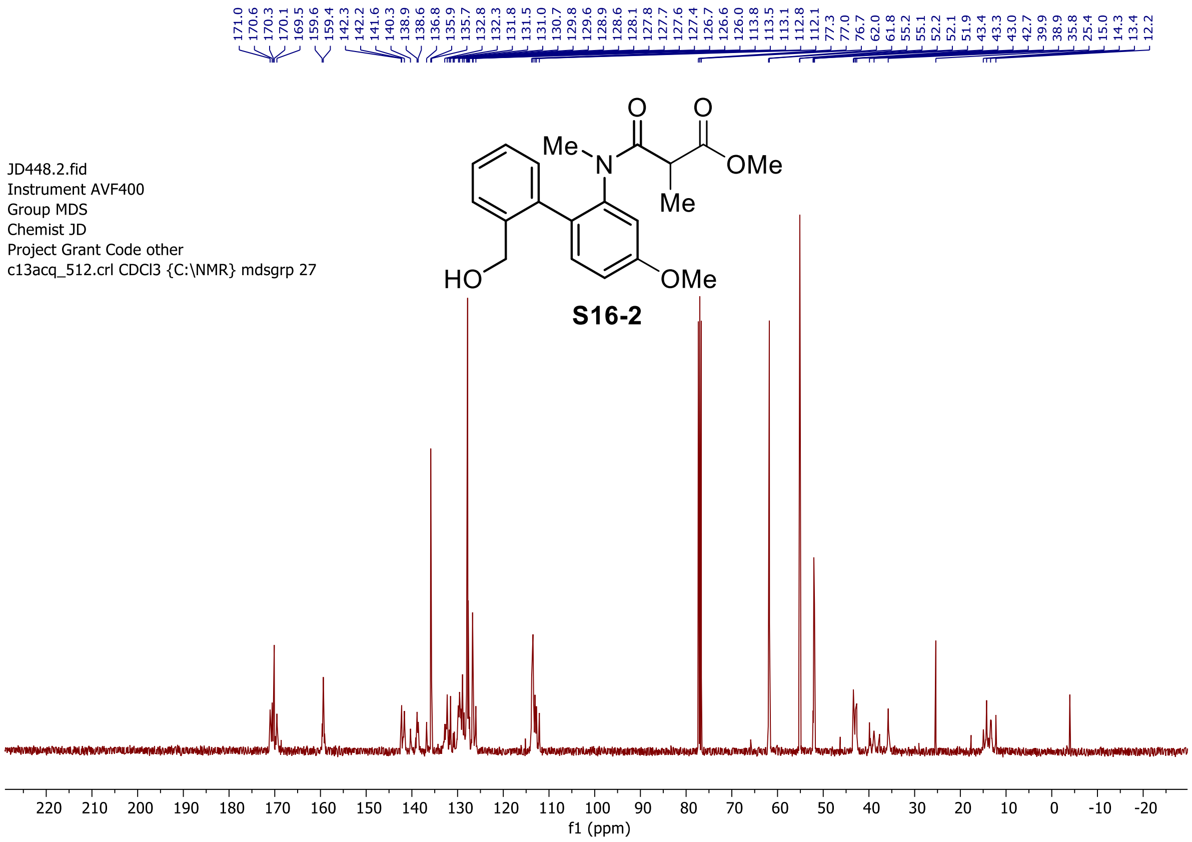
171.0
170.6
170.3
170.1
169.5
159.6
159.4
142.3
142.2
141.6
140.3
138.9
138.6
136.8
135.9
135.7
132.8
132.3
131.8
131.5
131.0
130.7
129.8
129.6
128.9
128.6
128.1
127.8
127.7
127.6
127.4
126.7
126.6
126.0
113.8
113.5
113.1
112.8
112.1
77.3
77.0
76.7
62.0
61.8
55.2
55.1
52.2
52.1
51.9
43.4
43.3
43.0
42.7
39.9
38.9
35.8
25.4
15.0
14.3
13.4
12.2
JD448.2.fid
Instrument AVF400
Group MDS
Chemist JD
Project Grant Code other
c13acq_512.crl CDCl3 {C:\NMR} mdsgrp 27
O
O
Me
N
OMe
Me
HO
OMe
S16-2
220 210 200 190 180 170 160 150 140 130 120 110 100 90 80 70 60 50 40 30 20 10 0 -10 -20
f1 (ppm)

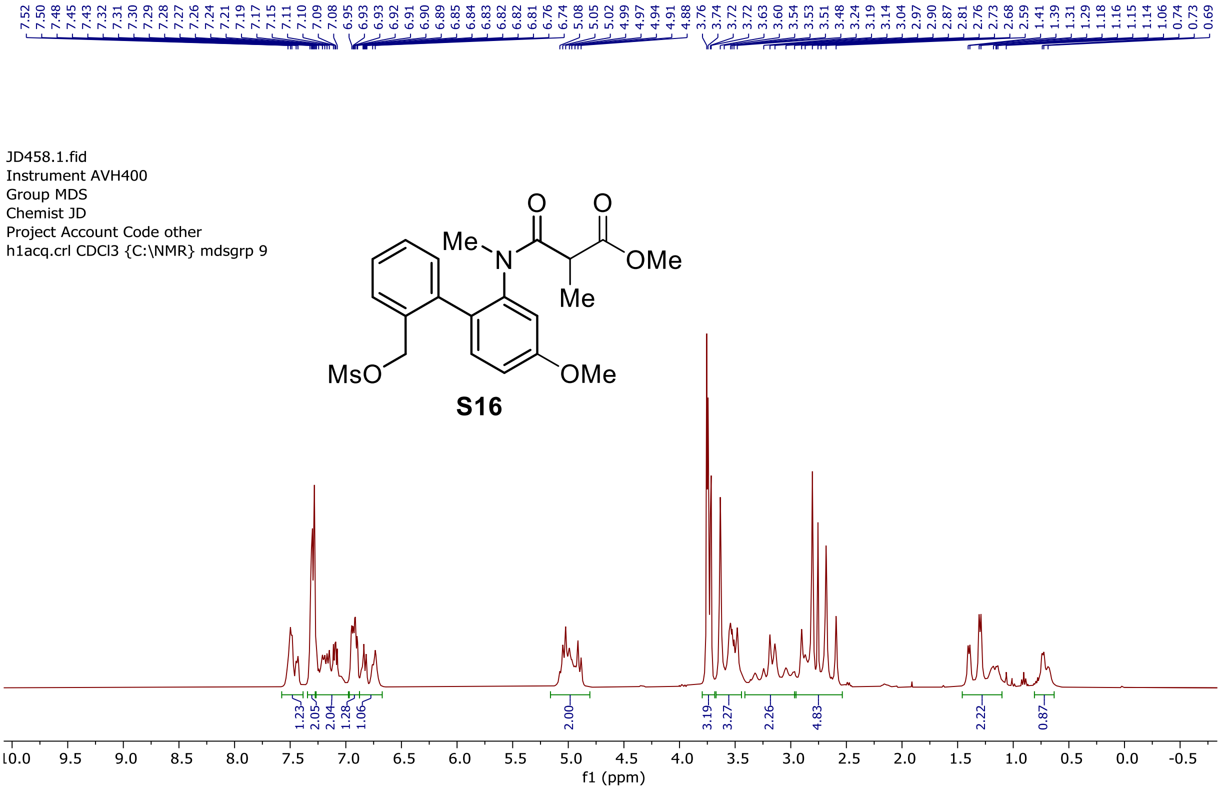
7.52
7.50
7.48
7.45
7.43
7.32
7.31
7.30
7.29
7.28
7.27
7.26
7.24
7.21
7.19
7.17
7.15
7.11
7.10
7.09
7.08
6.95
6.93
6.93
6.92
6.91
6.90
6.89
6.85
6.84
6.83
6.82
6.82
6.81
6.76
6.74
5.08
5.05
5.02
4.99
4.97
4.94
4.91
4.88
3.76
3.74
3.72
3.72
3.63
3.60
3.54
3.53
3.51
3.48
3.24
3.19
3.14
3.04
2.97
2.90
2.87
2.81
2.76
2.73
2.68
2.59
1.41
1.39
1.31
1.29
1.18
1.16
1.15
1.14
1.06
0.74
0.73
0.69
JD458.1.fid
Instrument AVH400
Group MDS
Chemist JD
Project Account Code other
h1acq.crl CDCl3 {C:\NMR} mdsgrp 9
O
O
Me
N
OMe
Me
MsO
OMe
S16
1.23
2.05
2.04
1.28
1.06
2.00
3.19
3.27
2.26
4.83
2.22
0.87
10.0 9.5 9.0 8.5 8.0 7.5 7.0 6.5 6.0 5.5 5.0 4.5 4.0 3.5 3.0 2.5 2.0 1.5 1.0 0.5 0.0 -0.5
f1 (ppm)

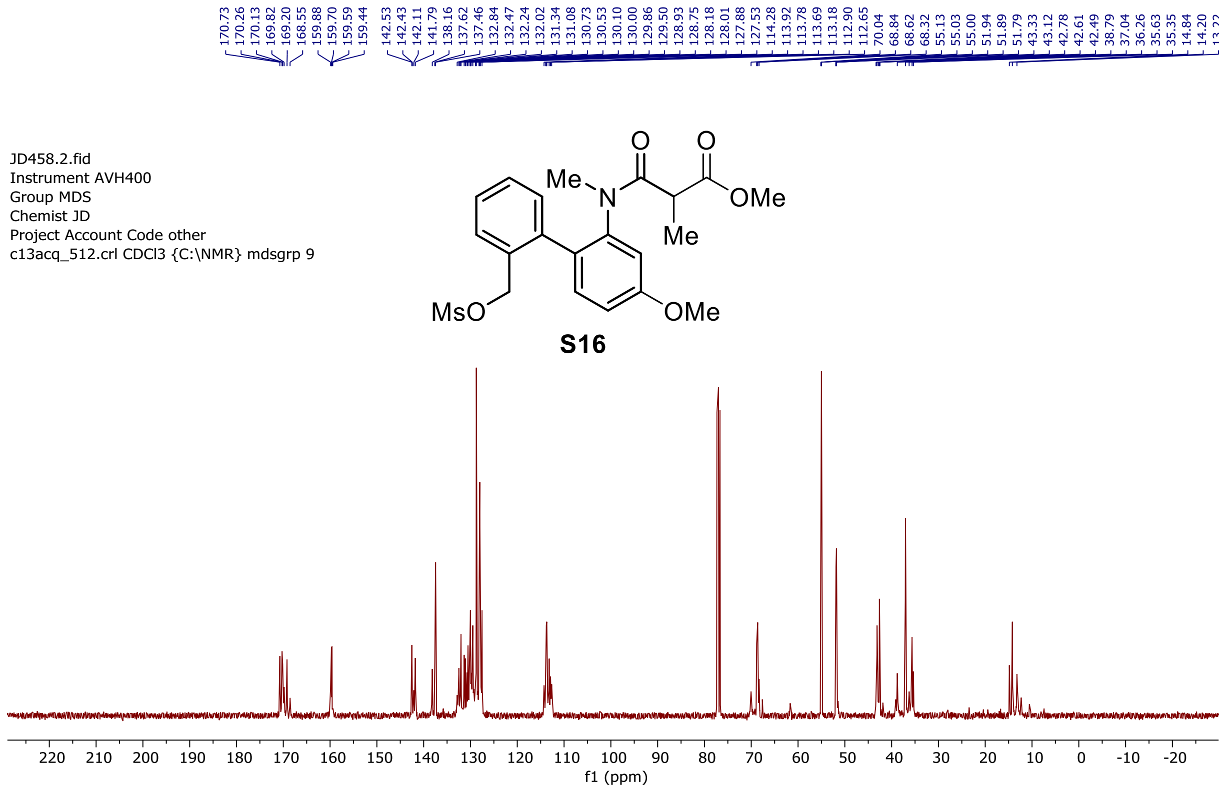
170.73 170.26 170.13 169.82 169.20 168.55 159.88 159.70 159.59 159.44 142.53 142.43 142.11 141.79 138.16 137.62 137.46 132.84 132.47 132.24 132.02 131.34 131.08 130.73 130.53 130.10 130.00 129.86 129.50 128.93 128.75 128.18 128.01 127.88 127.53 114.28 113.92 113.78 113.69 113.18 112.90 112.65 70.04 68.84 68.62 68.32 55.13 55.03 55.00 51.94 51.89 51.79 43.33 43.12 42.78 42.61 42.49 38.79 37.04 36.26 35.63 35.35 14.84 14.20
JD458.2.fid
Instrument AVH400
Group MDS
Chemist JD
Project Account Code other
c13acq_512.crl CDCl3 {C:\NMR} mdsgrp 9
Me
N
O
O
OMe
Me
MsO
OMe
S16
f1 (ppm)

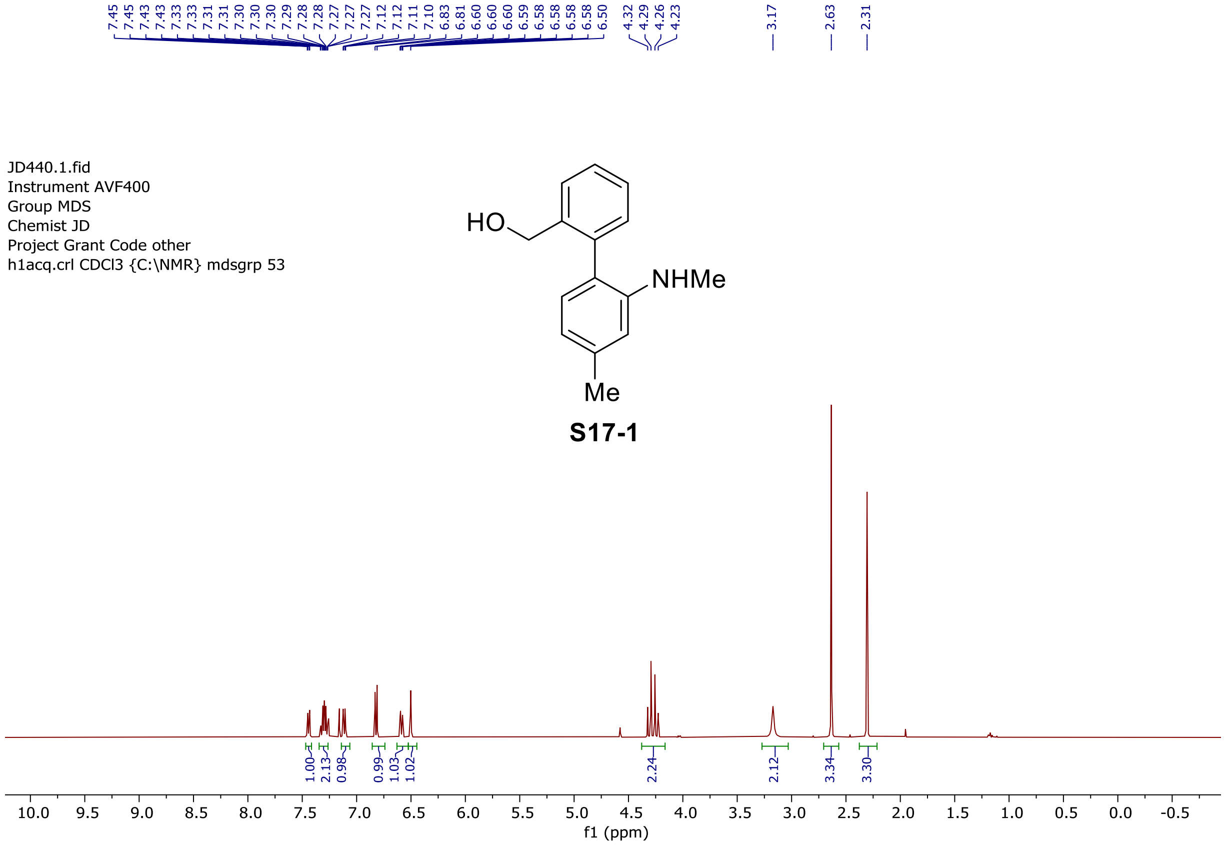
7.45 7.45 7.43 7.43 7.33 7.33 7.31 7.31 7.30 7.30 7.30 7.29 7.28 7.28 7.27 7.27 7.27 7.12 7.12 7.11 7.10 6.83 6.81 6.60 6.60 6.60 6.59 6.58 6.58 6.58 6.58 6.50 4.32 4.29 4.26 4.23 3.17 2.63 2.31
JD440.1.fid
Instrument AVF400
Group MDS
Chemist JD
Project Grant Code other
h1acq.crl CDCl3 {C:\NMR} mdsgrp 53
HO
NHMe
Me
S17-1
1.00 2.13 0.98 0.99 1.03 1.02 2.24 2.12 3.34 3.30
f1 (ppm)

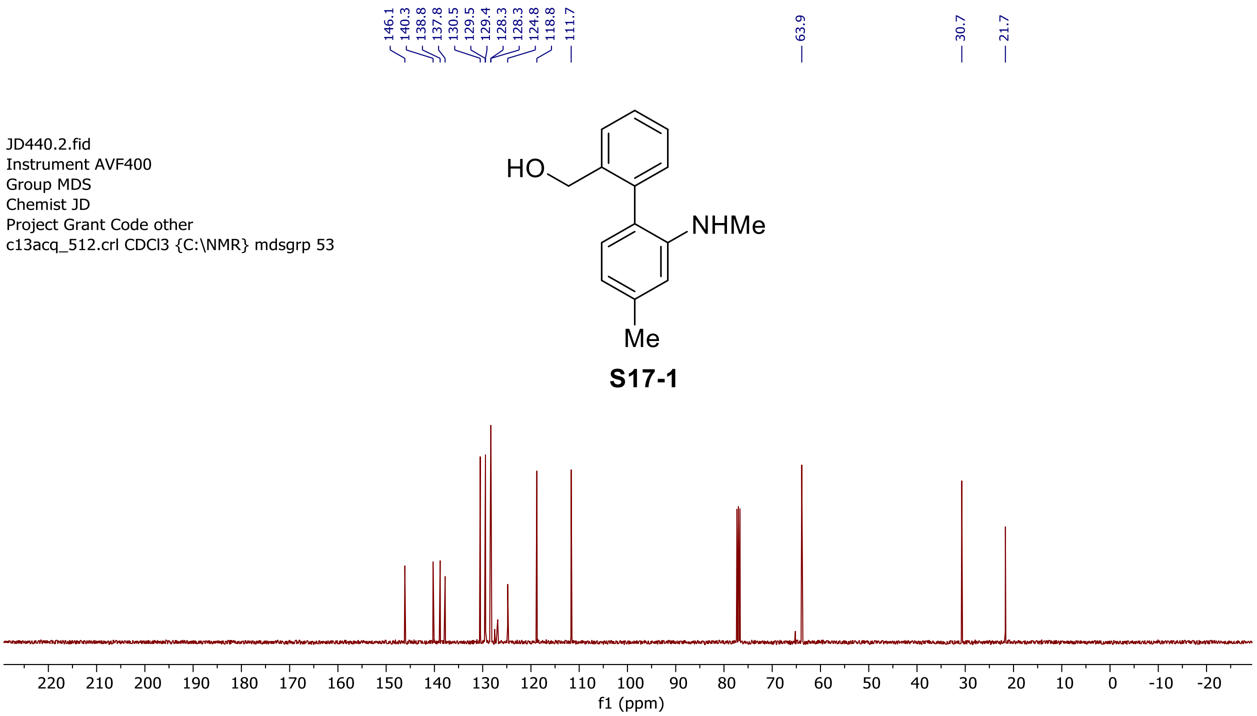
146.1 140.3 138.8 137.8 130.5 129.5 129.4 128.3 128.3 124.8 118.8 111.7
63.9
30.7
21.7
JD440.2.fid
Instrument AVF400
Group MDS
Chemist JD
Project Grant Code other
c13acq_512.crl CDCl3 {C:\NMR} mdsgrp 53
HO
NHMe
Me
S17-1
220 210 200 190 180 170 160 150 140 130 120 110 100 90 80 70 60 50 40 30 20 10 0 -10 -20
f1 (ppm)

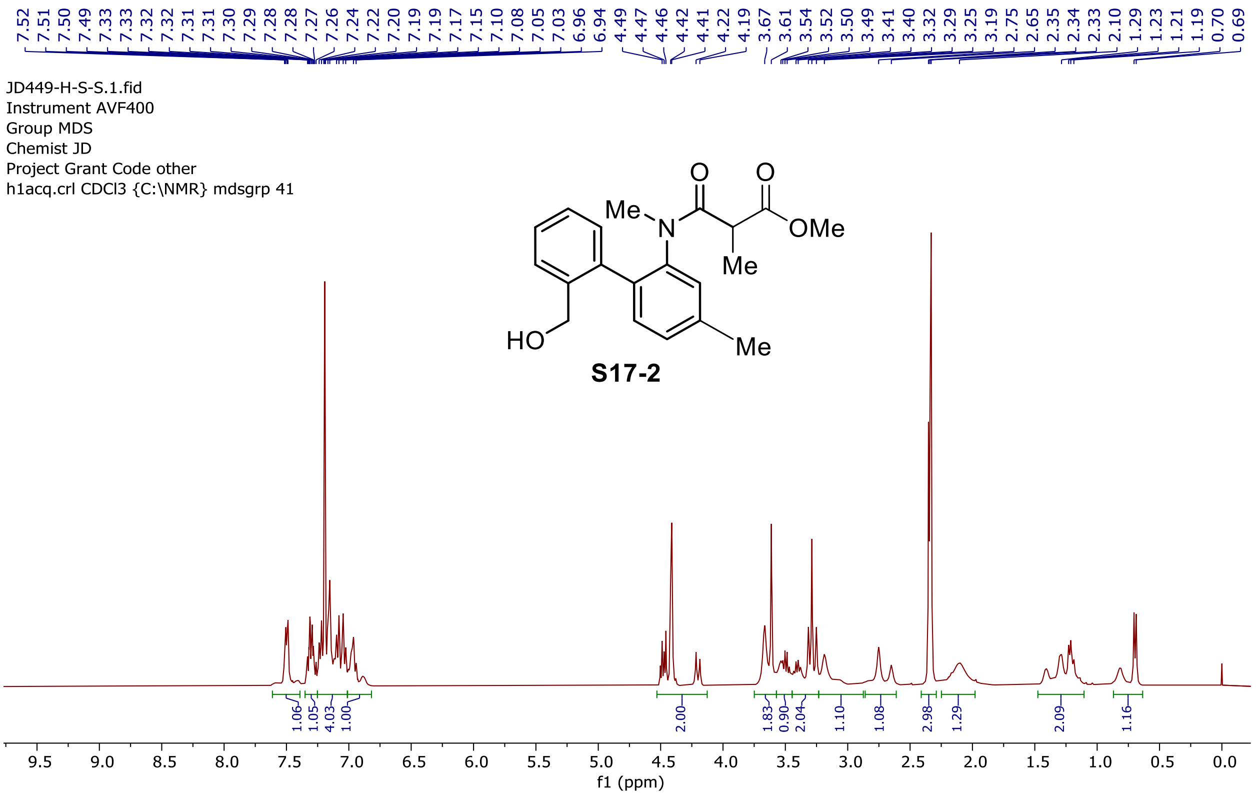
7.52 7.51 7.50 7.49 7.33 7.33 7.32 7.32 7.31 7.31 7.30 7.29 7.28 7.27 7.26 7.24 7.22 7.20 7.19 7.19 7.17 7.15 7.10 7.08 7.05 7.03 6.96 6.94 4.49 4.47 4.46 4.42 4.41 4.22 4.19 3.67 3.61 3.54 3.52 3.50 3.49 3.41 3.40 3.32 3.29 3.25 3.19 2.75 2.65 2.35 2.34 2.33 2.10 1.29 1.23 1.21 1.19 0.70 0.69
JD449-H-S-S.1.fid
Instrument AVF400
Group MDS
Chemist JD
Project Grant Code other
h1acq.crl CDCl3 {C:\NMR} mdsgrp 41
Me
N
O
O
OMe
Me
HO
Me
S17-2
1.06 1.05 4.03 1.00 2.00 1.83 0.90 2.04 1.10 1.08 2.98 1.29 2.09 1.16
9.5 9.0 8.5 8.0 7.5 7.0 6.5 6.0 5.5 5.0 4.5 4.0 3.5 3.0 2.5 2.0 1.5 1.0 0.5 0.0
f1 (ppm)

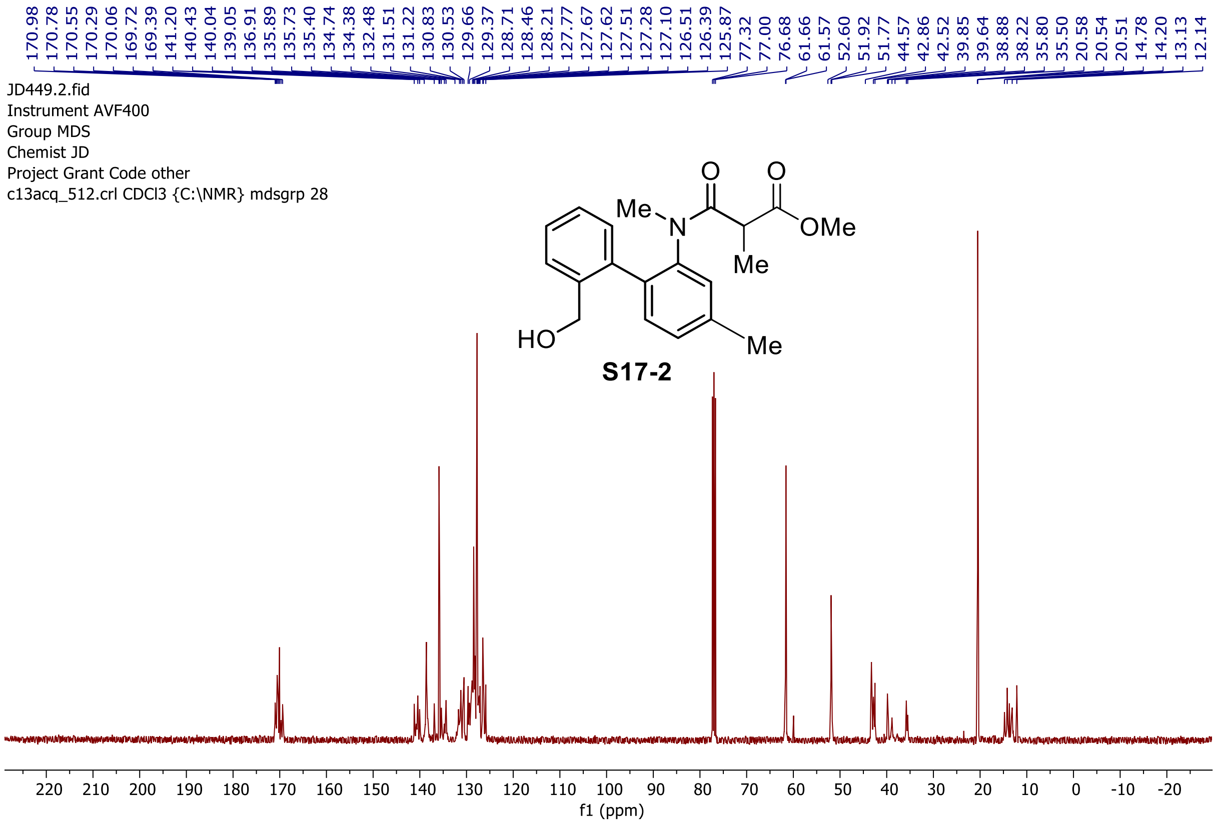
170.98 170.78 170.55 170.29 170.06 169.72 169.39 141.20 140.43 140.04 139.05 136.91 135.89 135.73 135.40 134.74 134.38 132.48 131.51 131.22 130.83 130.53 129.66 129.37 128.71 128.46 128.21 127.77 127.67 127.62 127.51 127.28 127.10 126.51 126.39 125.87 77.32 77.00 76.68 61.66 61.57 52.60 51.92 51.77 44.57 42.86 42.52 39.85 39.64 38.88 38.22 35.80 35.50 20.58 20.54 20.51 14.78 14.20 13.13 12.14
JD449.2.fid
Instrument AVF400
Group MDS
Chemist JD
Project Grant Code other
c13acq_512.crl CDCl3 {C:\NMR} mdsgrp 28
S17-2
f1 (ppm)

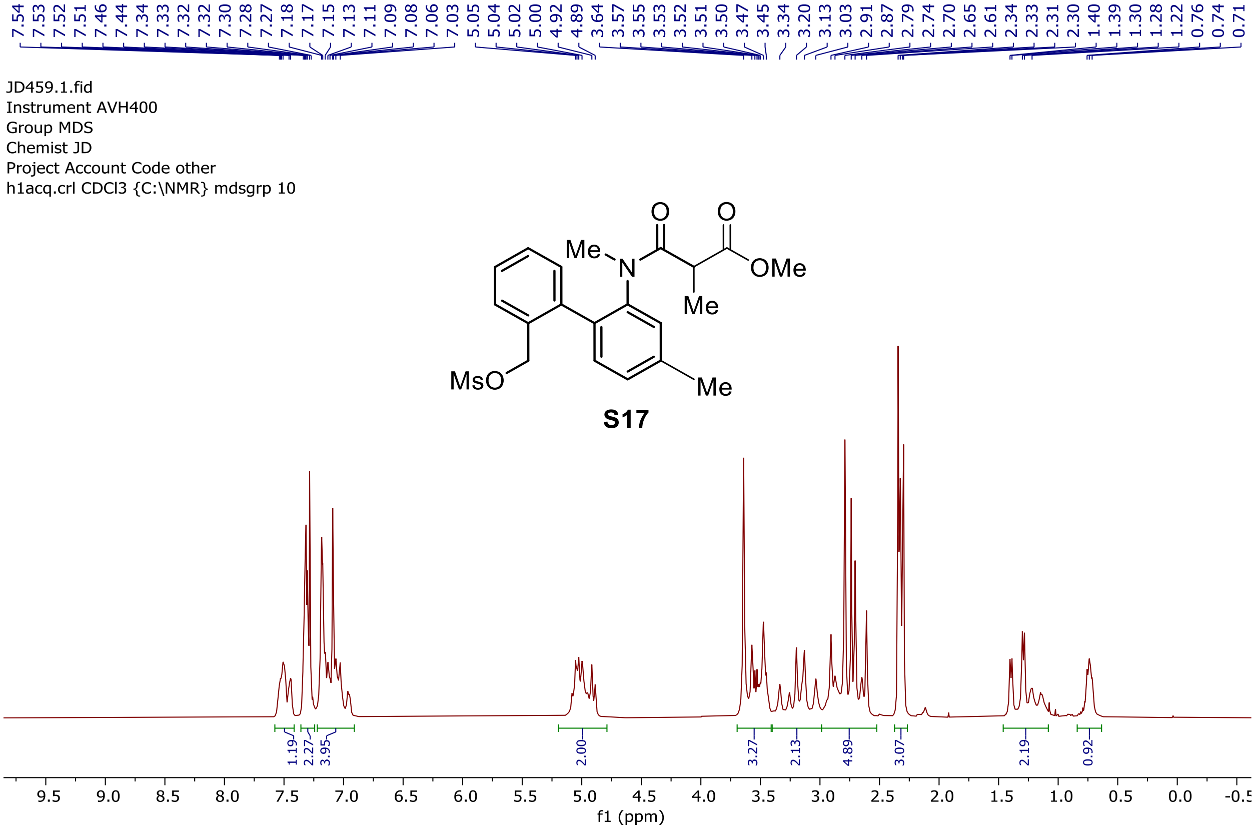
7.54 7.53 7.52 7.51 7.46 7.44 7.34 7.33 7.32 7.32 7.30 7.28 7.27 7.18 7.17 7.15 7.13 7.11 7.09 7.08 7.06 7.03 5.05 5.04 5.02 5.00 4.92 4.89 3.64 3.57 3.55 3.53 3.52 3.51 3.50 3.47 3.45 3.34 3.20 3.13 3.03 2.91 2.87 2.79 2.74 2.70 2.65 2.61 2.34 2.33 2.31 2.30 1.40 1.39 1.30 1.28 1.22 0.76 0.74 0.71
JD459.1.fid
Instrument AVH400
Group MDS
Chemist JD
Project Account Code other
h1acq.crl CDCl3 {C:\NMR} mdsgrp 10
S17
f1 (ppm)

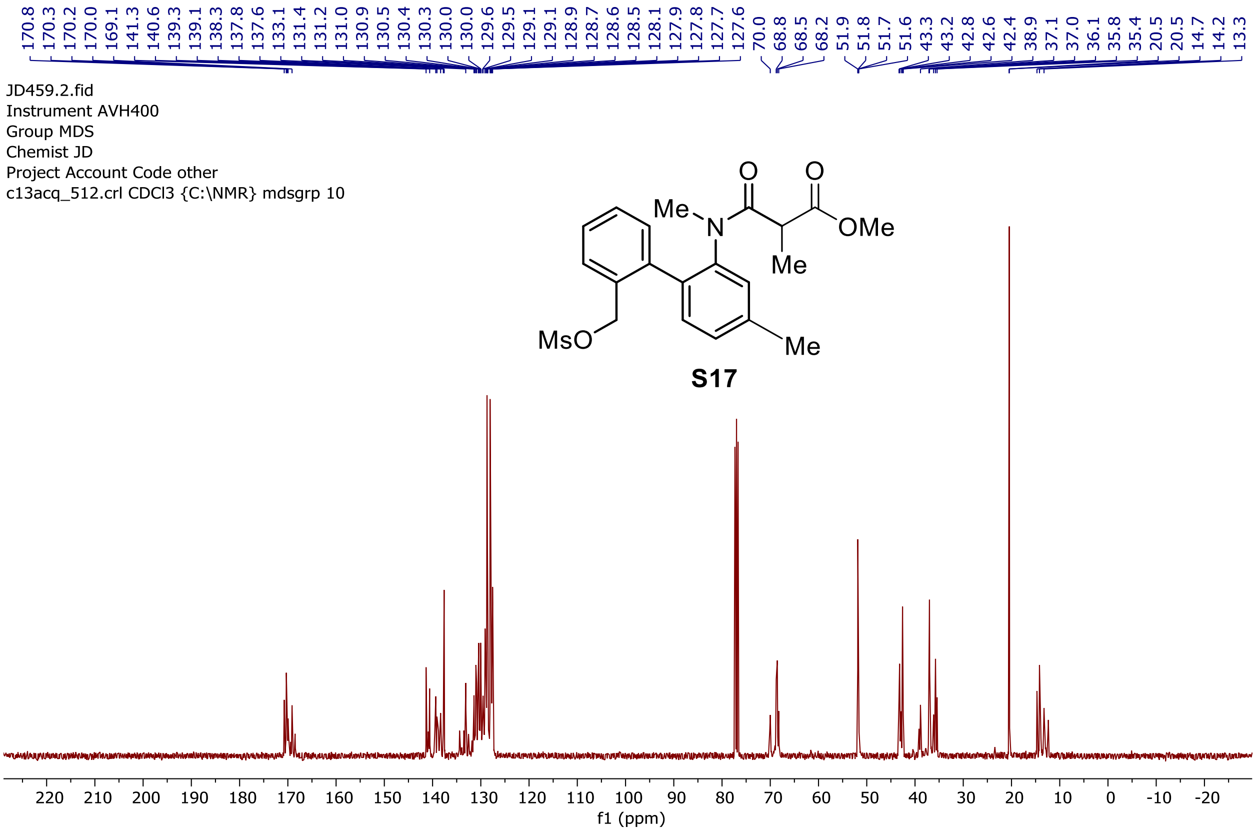
170.8 170.3 170.2 170.0 169.1 141.3 140.6 139.3 139.1 138.3 137.8 137.6 133.1 131.4 131.2 131.0 130.9 130.5 130.4 130.3 130.0 130.0 129.6 129.5 129.1 129.1 128.9 128.7 128.6 128.5 128.1 127.9 127.8 127.7 127.6 70.0 68.8 68.5 68.2 51.9 51.8 51.7 51.6 43.3 43.2 42.8 42.6 42.4 38.9 37.1 37.0 36.1 35.8 35.4 20.5 20.5 14.7 14.2 13.3
JD459.2.fid
Instrument AVH400
Group MDS
Chemist JD
Project Account Code other
c13acq_512.crl CDCl3 {C:\NMR} mdsgrp 10
Me
N
O
O
OMe
Me
MsO
Me
220 210 200 190 180 170 160 150 140 130 120 110 100 90 80 70 60 50 40 30 20 10 0 -10 -20
f1 (ppm)

S17

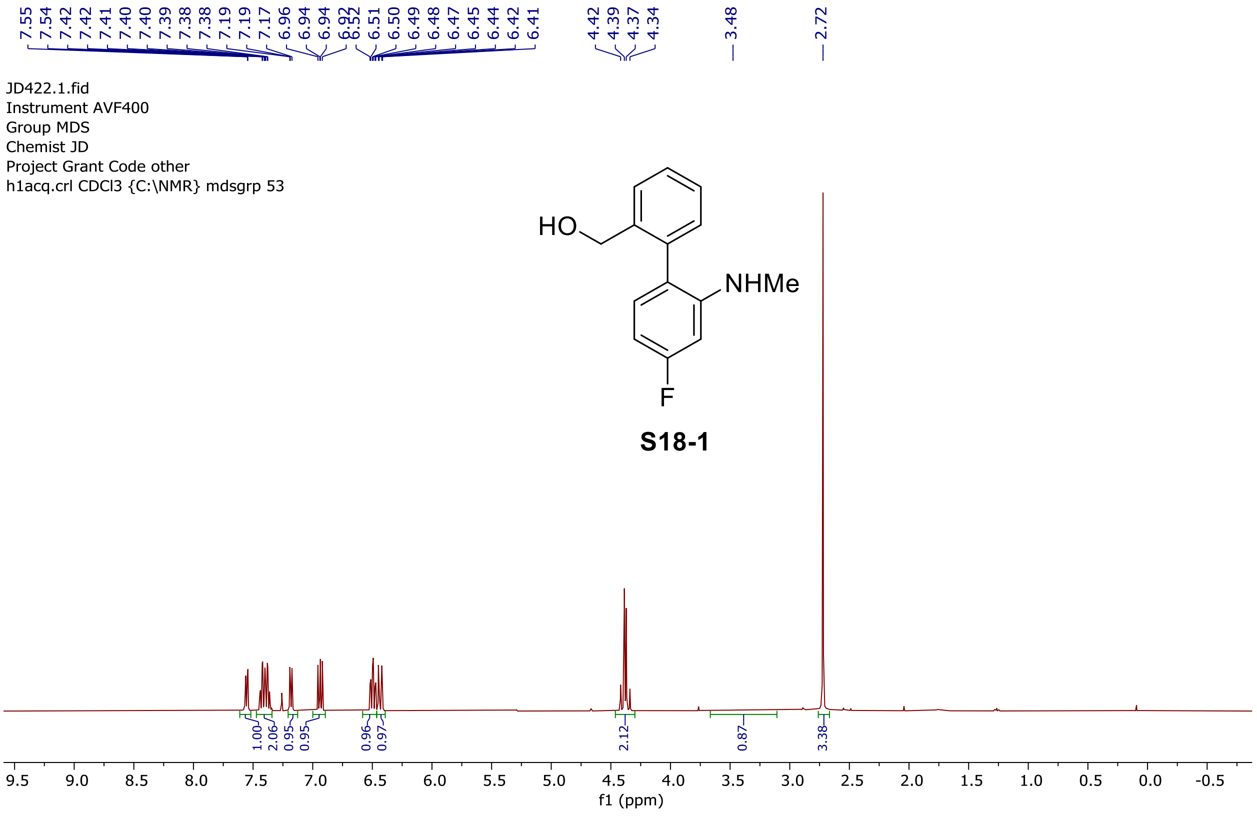
7.55 7.54 7.42 7.42 7.41 7.40 7.40 7.39 7.38 7.38 7.19 7.19 7.17 6.96 6.94 6.94 6.92 6.52 6.51 6.50 6.49 6.48 6.47 6.45 6.44 6.42 6.41 4.42 4.39 4.37 4.34 3.48 2.72
JD422.1.fid
Instrument AVF400
Group MDS
Chemist JD
Project Grant Code other
h1acq.crl CDCl3 {C:\NMR} mdsgrp 53
HO
NHMe
F
1.00 2.06 0.95 0.95 0.96 0.97 2.12 0.87 3.38
9.5 9.0 8.5 8.0 7.5 7.0 6.5 6.0 5.5 5.0 4.5 4.0 3.5 3.0 2.5 2.0 1.5 1.0 0.5 0.0 -0.5
f1 (ppm)

S18-1

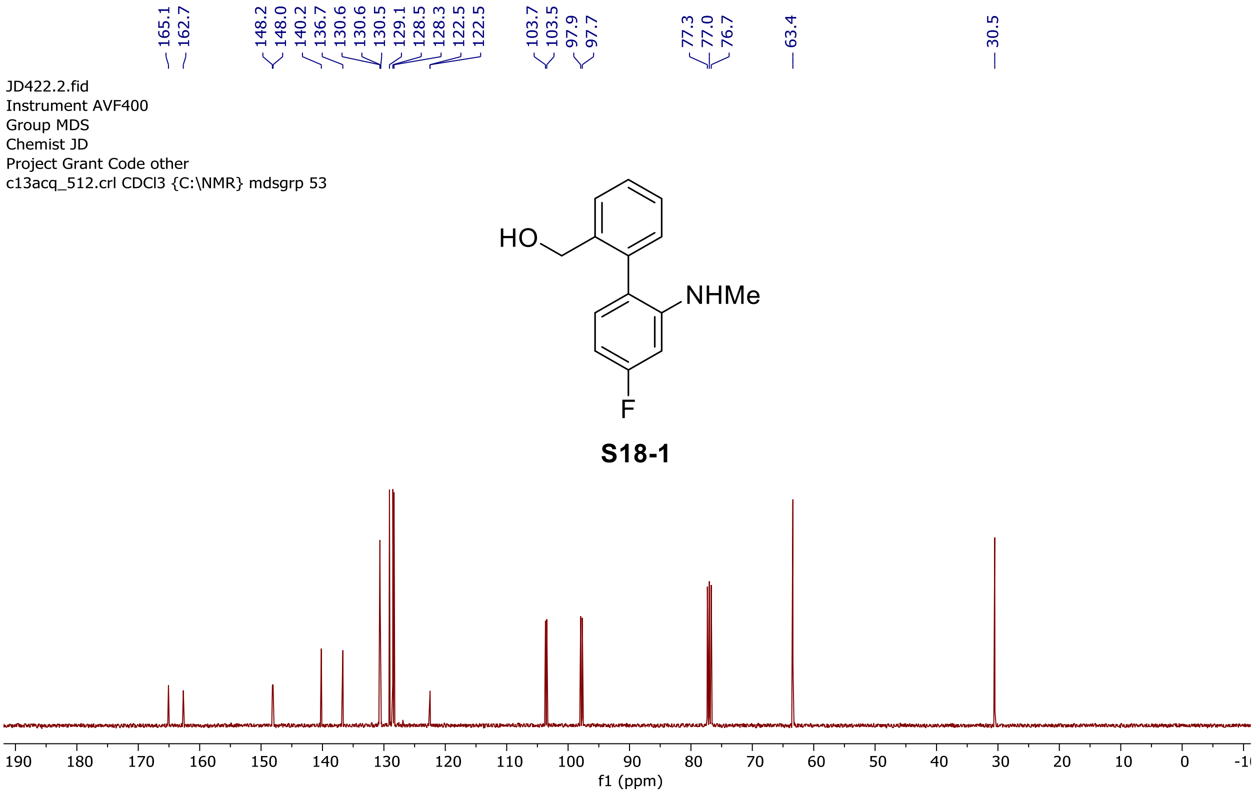
165.1
162.7
148.2
148.0
140.2
136.7
130.6
130.6
130.5
129.1
128.5
128.3
122.5
122.5
103.7
103.5
97.9
97.7
77.3
77.0
76.7
63.4
30.5
JD422.2.fid
Instrument AVF400
Group MDS
Chemist JD
Project Grant Code other
c13acq_512.crl CDCl3 {C:\NMR} mdsgrp 53
HO
NHMe
F
S18-1
f1 (ppm)

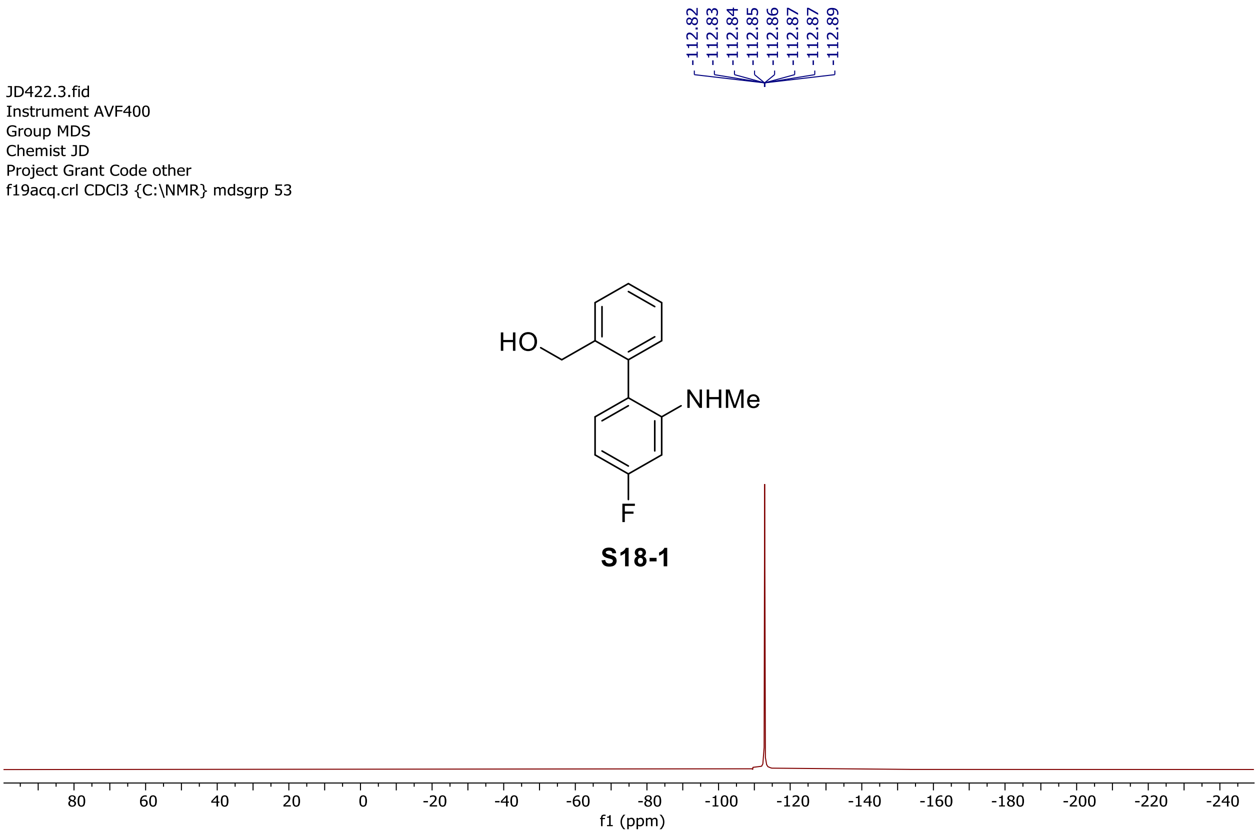
-112.82
-112.83
-112.84
-112.85
-112.86
-112.87
-112.87
-112.89
JD422.3.fid
Instrument AVF400
Group MDS
Chemist JD
Project Grant Code other
f19acq.crl CDCl3 {C:\NMR} mdsgrp 53
HO
NHMe
F
S18-1
f1 (ppm)

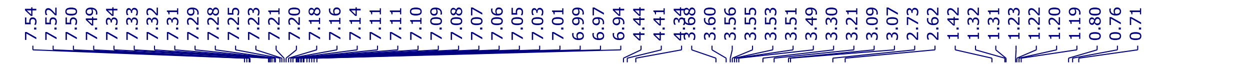

JD435.1.fid
Instrument AVH400
Group MDS
Chemist JD
Project Account Code other
h1acq.crl CDCl3 {C:\NMR} mdsgrp 8

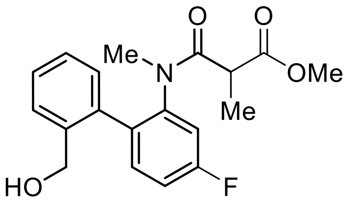

S18-2

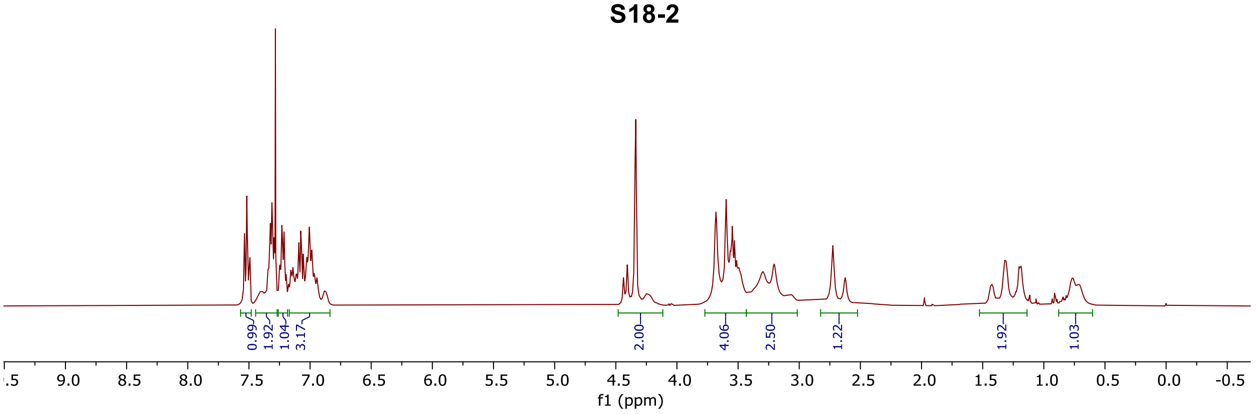

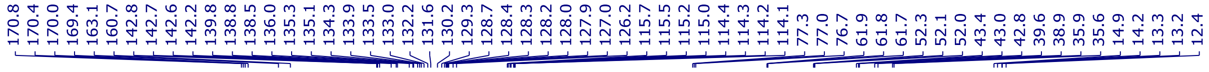

JD435.2.fid
Instrument AVH400
Group MDS
Chemist JD
Project Account Code other
c13acq_512.crl CDCl3 {C:\NMR} mdsgrp 8

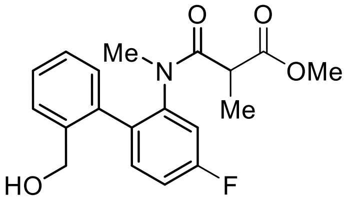

S18-2

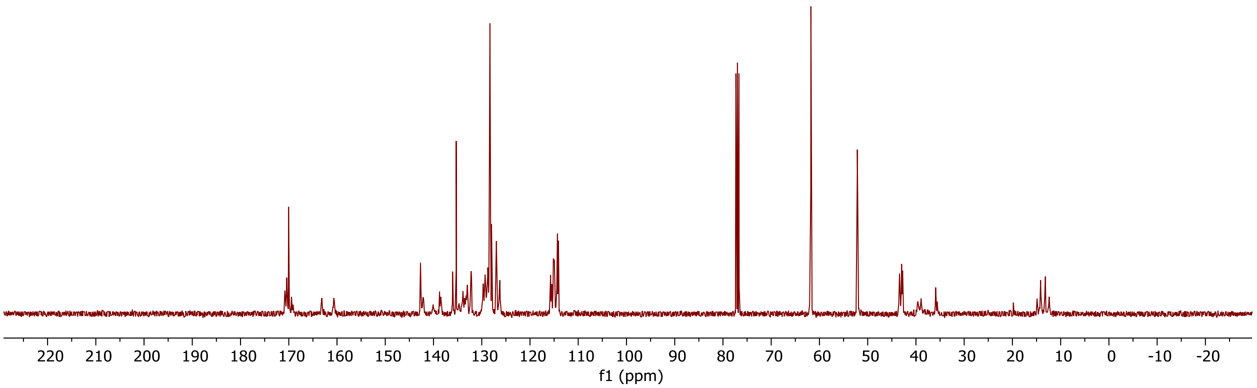

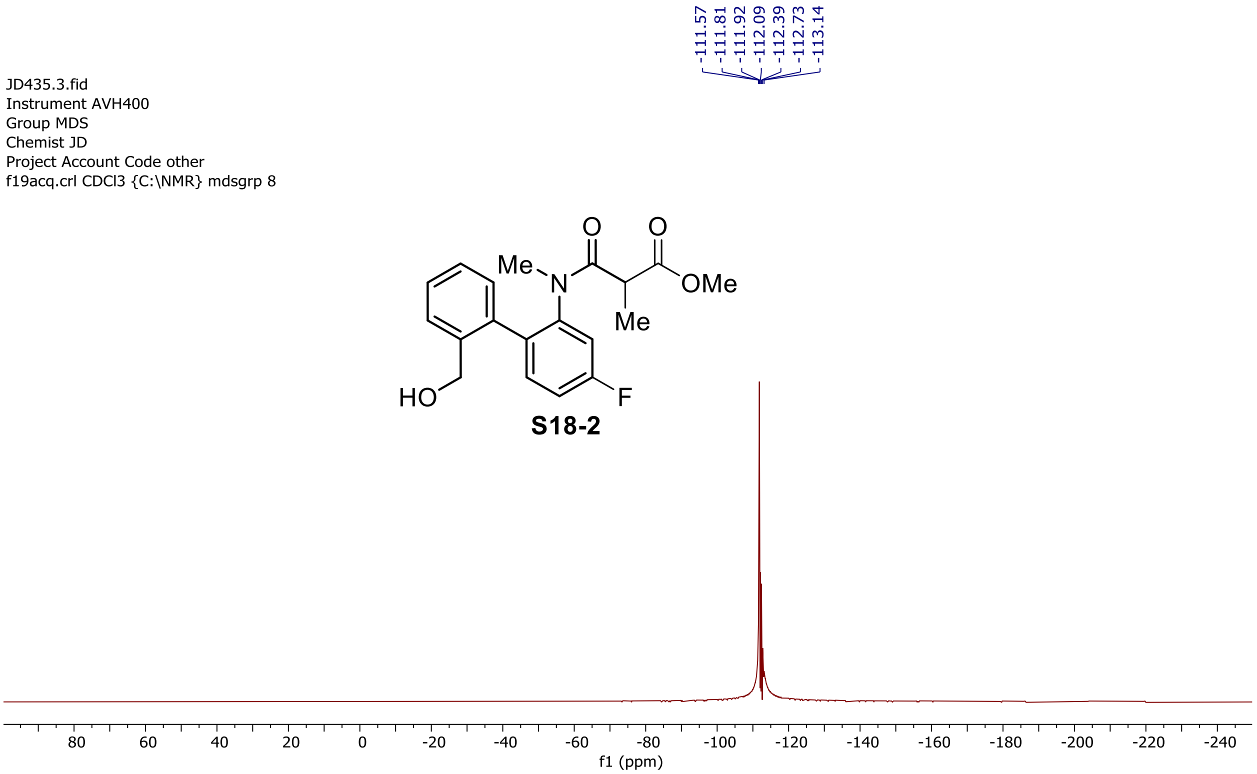
-111.57
-111.81
-111.92
-112.09
-112.39
-112.73
-113.14
JD435.3.fid
Instrument AVH400
Group MDS
Chemist JD
Project Account Code other
f19acq.crl CDCl3 {C:\NMR} mdsgrp 8
S18-2
f1 (ppm)

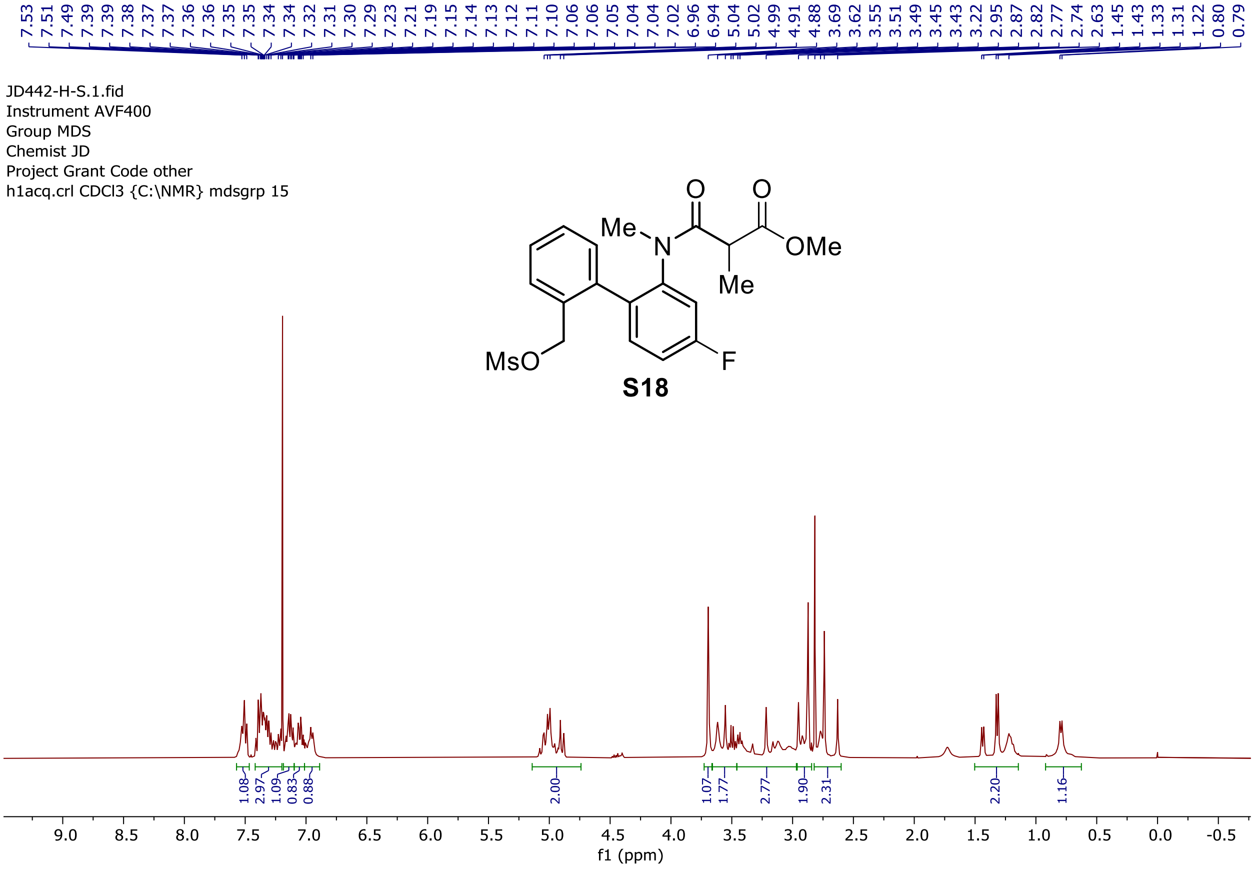
7.53 7.51 7.49 7.39 7.39 7.38 7.37 7.37 7.36 7.36 7.35 7.35 7.34 7.34 7.32 7.31 7.30 7.29 7.23 7.21 7.19 7.15 7.14 7.13 7.12 7.11 7.10 7.06 7.05 7.04 7.04 7.02 6.96 6.94 5.04 5.02 4.99 4.91 4.88 3.69 3.62 3.55 3.51 3.49 3.45 3.43 3.22 2.95 2.87 2.82 2.77 2.74 2.63 1.45 1.43 1.33 1.31 1.22 0.80 0.79
JD442-H-S.1.fid
Instrument AVF400
Group MDS
Chemist JD
Project Grant Code other
h1acq.crl CDCl3 {C:\NMR} mdsgrp 15
S18
1.08 2.97 1.09 0.83 0.88 2.00 1.07 1.77 2.77 1.90 2.31 2.20 1.16
f1 (ppm)

170.6, 170.3, 170.1, 169.2, 168.5, 163.5, 163.3, 161.0, 160.8, 160.5, 143.2, 143.1, 142.5, 142.4, 137.7, 137.6, 137.0, 136.9, 133.3, 133.2, 133.0, 132.9, 132.8, 132.2, 132.1, 131.1, 130.9, 130.7, 130.5, 129.9, 129.4, 129.1, 128.7, 128.5, 128.2, 128.1, 116.2, 116.0, 115.6, 115.5, 115.4, 115.3, 114.6, 114.4, 114.2, 68.6, 68.4, 68.1, 52.2, 52.1, 52.1, 43.5, 43.4, 43.1, 42.8, 42.7, 39.0, 37.4, 36.6, 35.9, 35.6, 14.9, 14.2, 13.3, [illegible]

JD442.2.fid
Instrument AVH400
Group MDS
Chemist JD
Project Account Code other
c13acq_512.crl CDCl3 {C:\NMR} mdsgrp 4

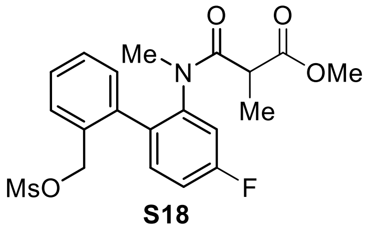

S18

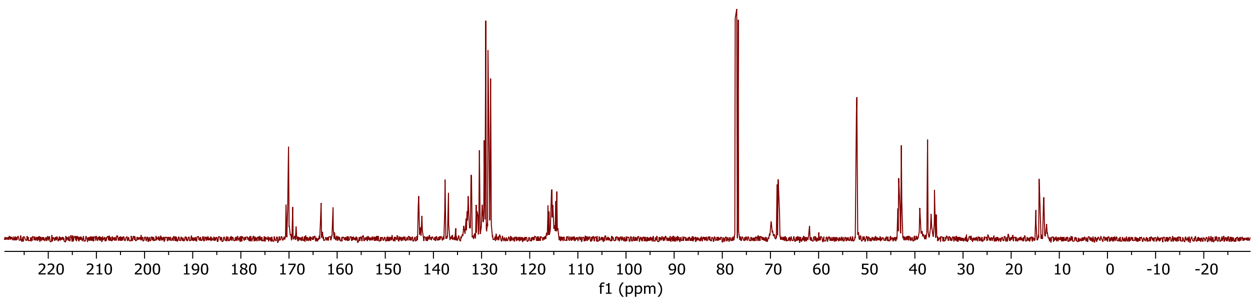

-110.44, -110.55, -110.57, -110.59, -110.61, -110.65, -110.67, -110.69, -110.80, -110.82, -110.85, -110.87, -111.84, -112.16

JD442.3.fid
Instrument AVH400
Group MDS
Chemist JD
Project Account Code other
f19acq.crl CDCl3 {C:\NMR} mdsgrp 4

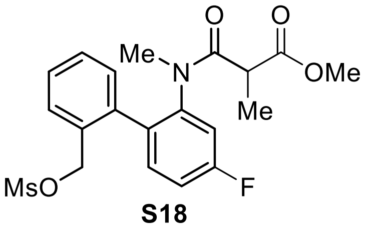

S18

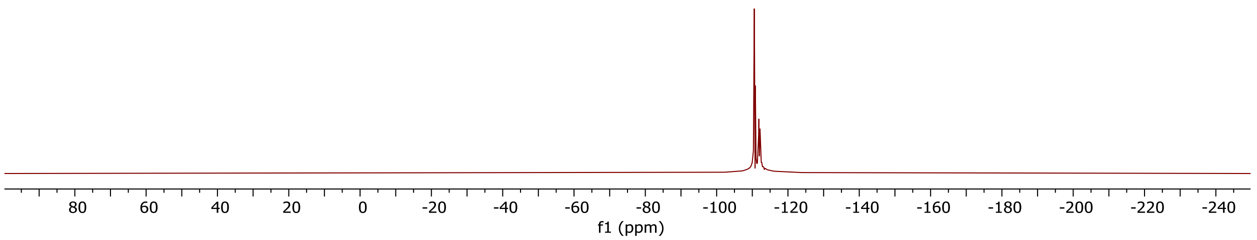

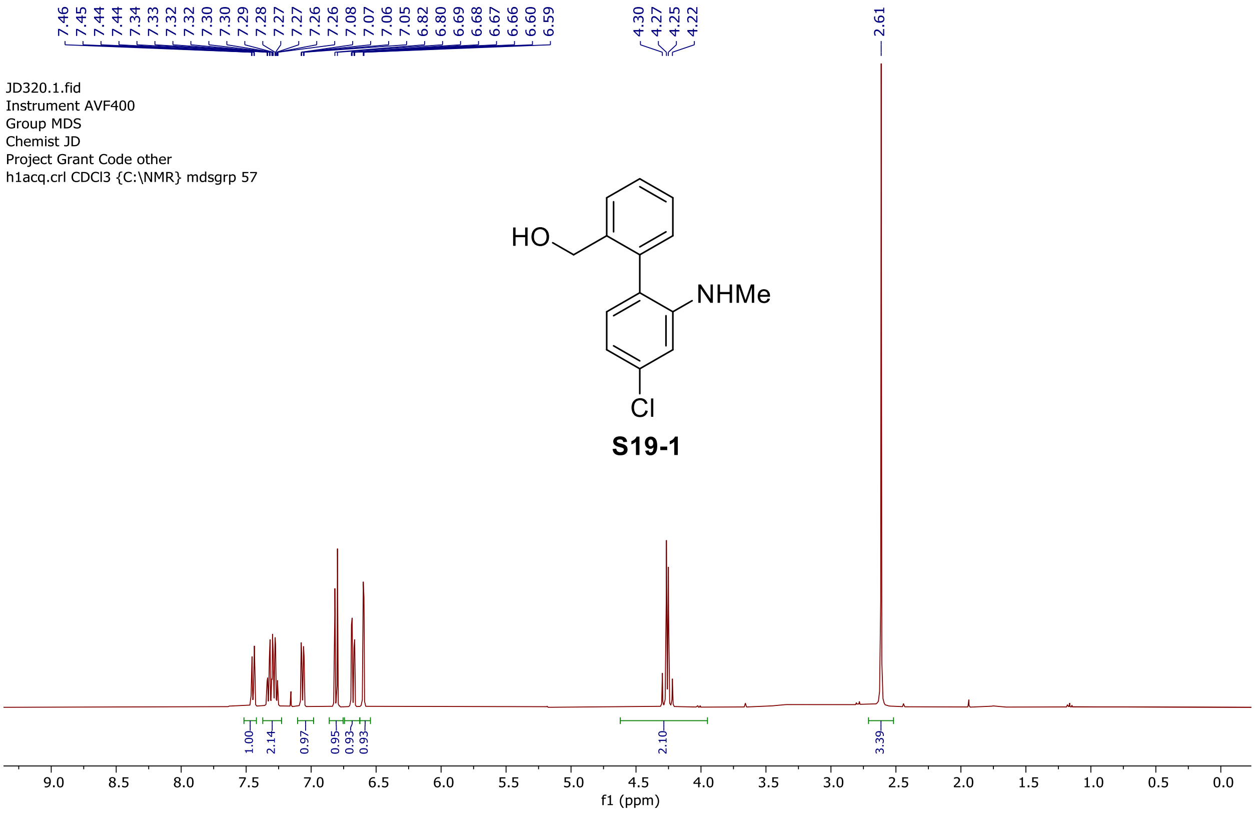
7.46 7.45 7.44 7.44 7.34 7.33 7.32 7.32 7.30 7.30 7.29 7.28 7.27 7.27 7.26 7.26 7.08 7.07 7.06 7.05 6.82 6.80 6.69 6.68 6.67 6.66 6.60 6.59
4.30 4.27 4.25 4.22
2.61
JD320.1.fid
Instrument AVF400
Group MDS
Chemist JD
Project Grant Code other
h1acq.crl CDCl3 {C:\NMR} mdsgrp 57
HO
NHMe
Cl
S19-1
1.00 2.14 0.97 0.95 0.93 0.93 2.10 3.39
f1 (ppm)

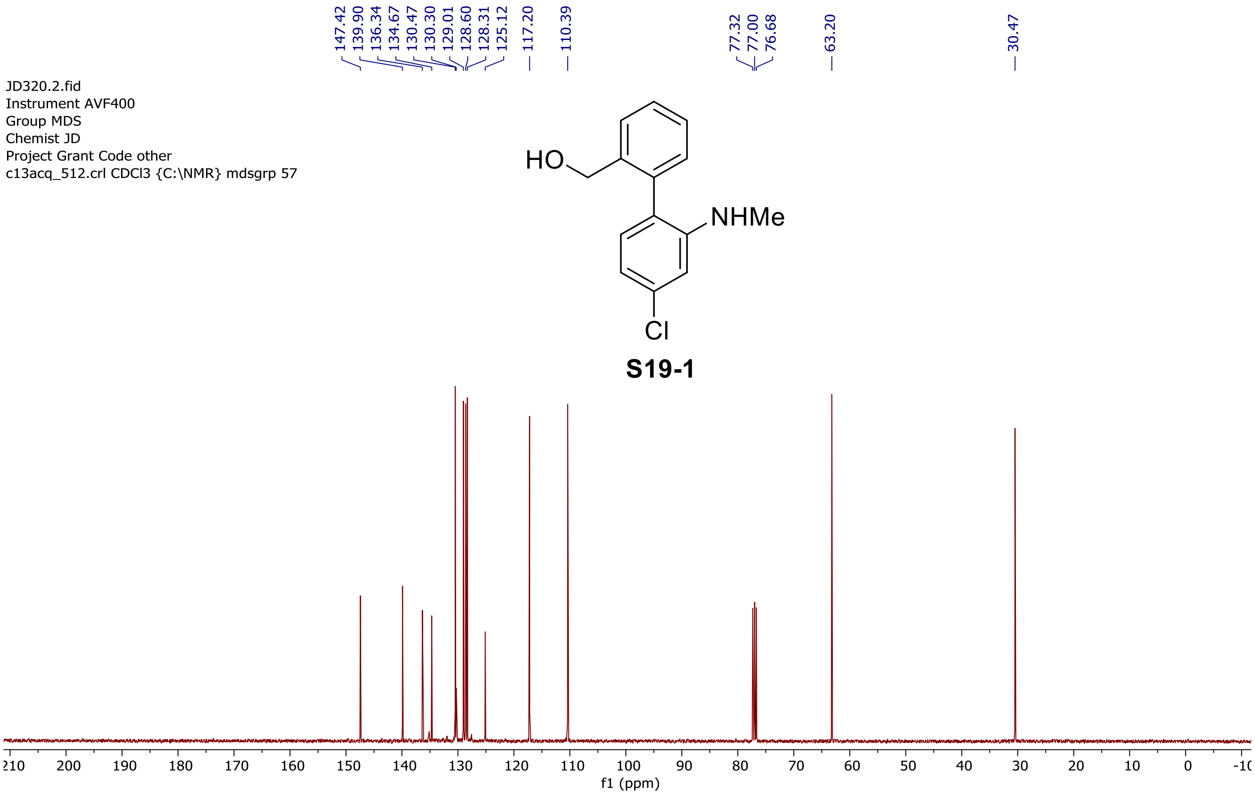
147.42 139.90 136.34 134.67 130.47 130.30 129.01 128.60 128.31 125.12 117.20 110.39
77.32 77.00 76.68 63.20 30.47
JD320.2.fid
Instrument AVF400
Group MDS
Chemist JD
Project Grant Code other
c13acq_512.crl CDCl3 {C:\NMR} mdsgrp 57
HO
NHMe
Cl
S19-1
f1 (ppm)

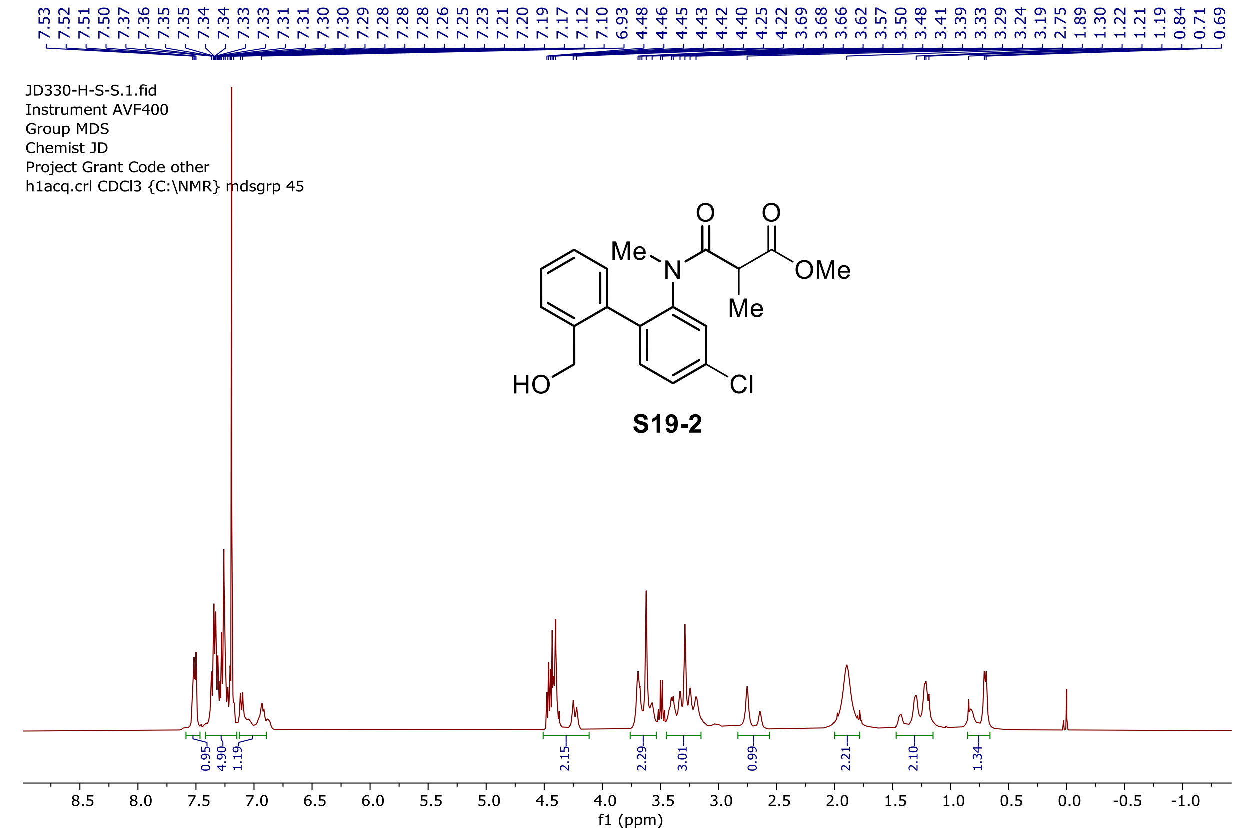
7.53 7.52 7.51 7.50 7.37 7.36 7.35 7.34 7.34 7.33 7.31 7.31 7.30 7.29 7.28 7.28 7.28 7.26 7.25 7.23 7.21 7.20 7.19 7.17 7.12 7.10 6.93 4.48 4.46 4.45 4.43 4.42 4.40 4.25 4.22 3.69 3.68 3.66 3.62 3.57 3.50 3.48 3.41 3.39 3.33 3.29 3.24 3.19 2.75 1.89 1.30 1.22 1.21 1.19 0.84 0.71 0.69
JD330-H-S-S.1.fid
Instrument AVF400
Group MDS
Chemist JD
Project Grant Code other
h1acq.crl CDCl3 {C:\NMR} mdsgrp 45
O
O
Me
N
OMe
Me
HO
Cl
S19-2
0.95
4.90
1.19
2.15
2.29
3.01
0.99
2.21
2.10
1.34
8.5 8.0 7.5 7.0 6.5 6.0 5.5 5.0 4.5 4.0 3.5 3.0 2.5 2.0 1.5 1.0 0.5 0.0 -0.5 -1.0
f1 (ppm)

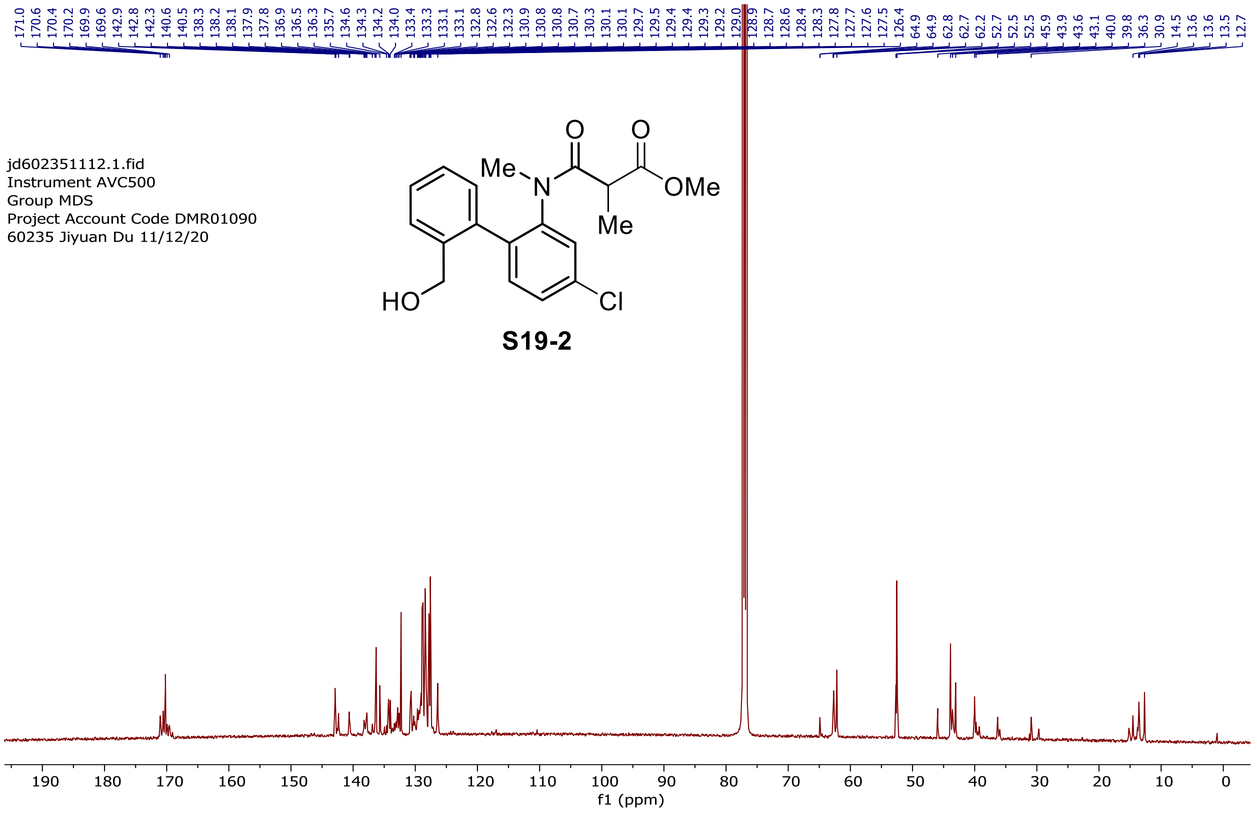
171.0 170.6 170.4 170.2 169.9 169.6 142.9 142.8 142.3 140.6 140.5 138.3 138.2 138.1 137.9 137.8 136.9 136.5 136.3 135.7 134.6 134.3 134.2 134.0 133.4 133.3 133.1 133.1 132.8 132.6 132.3 130.9 130.8 130.8 130.7 130.3 130.1 130.1 129.7 129.5 129.4 129.4 129.3 129.2 129.0 128.9 128.7 128.6 128.4 128.4 128.3 127.8 127.7 127.6 127.5 126.4 64.9 64.9 62.8 62.7 62.2 52.7 52.5 52.5 45.9 43.9 43.6 43.1 40.0 39.8 36.3 30.9 14.5 13.6 13.6 13.5 12.7
jd602351112.1.fid
Instrument AVC500
Group MDS
Project Account Code DMR01090
60235 Jiyuan Du 11/12/20
O
O
Me
N
OMe
Me
HO
Cl
S19-2
190 180 170 160 150 140 130 120 110 100 90 80 70 60 50 40 30 20 10 0
f1 (ppm)

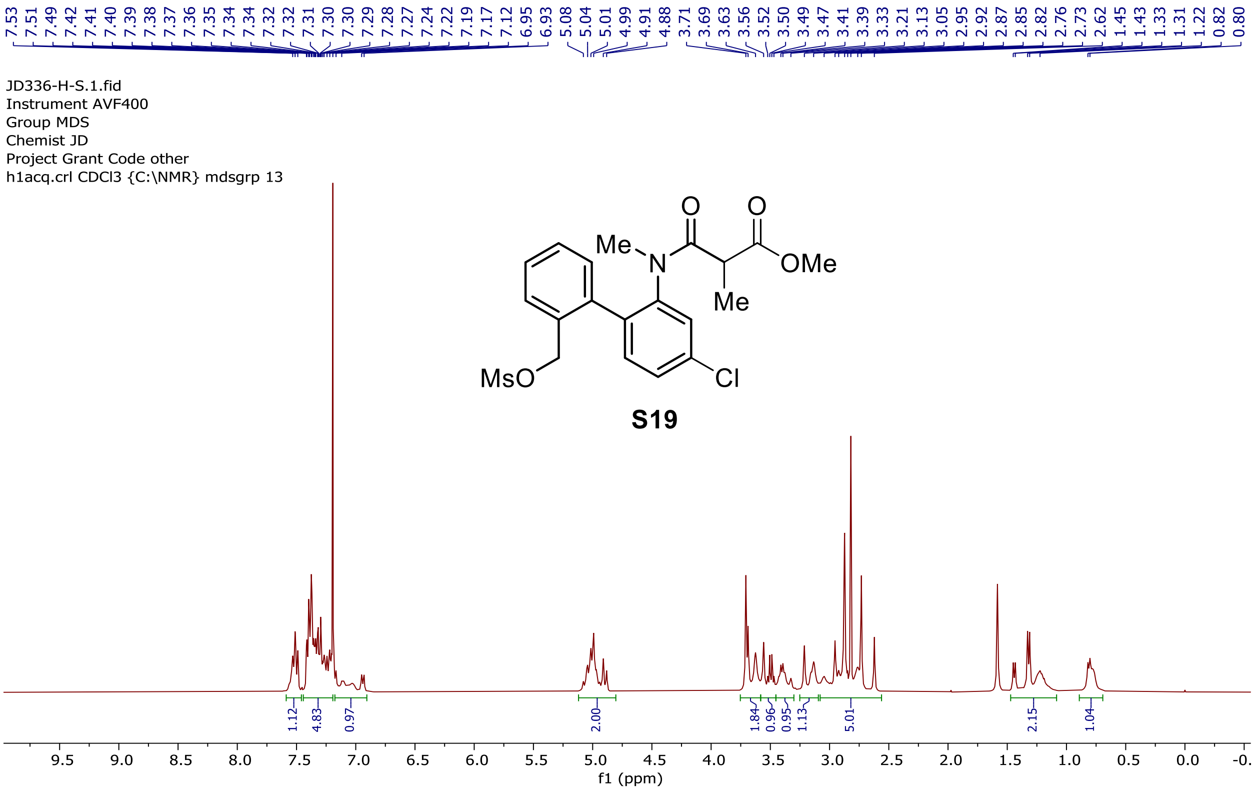
7.53 7.51 7.49 7.42 7.41 7.40 7.39 7.38 7.37 7.36 7.35 7.34 7.34 7.32 7.32 7.31 7.30 7.30 7.29 7.28 7.27 7.24 7.22 7.19 7.17 7.12 6.95 6.93 5.08 5.04 5.01 4.99 4.91 4.88 3.71 3.69 3.63 3.56 3.52 3.50 3.49 3.47 3.41 3.39 3.33 3.21 3.13 3.05 2.95 2.92 2.87 2.85 2.82 2.76 2.73 2.62 1.45 1.43 1.33 1.31 1.22 0.82 0.80
JD336-H-S.1.fid
Instrument AVF400
Group MDS
Chemist JD
Project Grant Code other
h1acq.crl CDCl3 {C:\NMR} mdsgrp 13
O
O
Me
N
OMe
Me
MsO
Cl
S19
1.12
4.83
0.97
2.00
1.84
0.96
0.95
1.13
5.01
2.15
1.04
9.5 9.0 8.5 8.0 7.5 7.0 6.5 6.0 5.5 5.0 4.5 4.0 3.5 3.0 2.5 2.0 1.5 1.0 0.5 0.0 -0.
f1 (ppm)

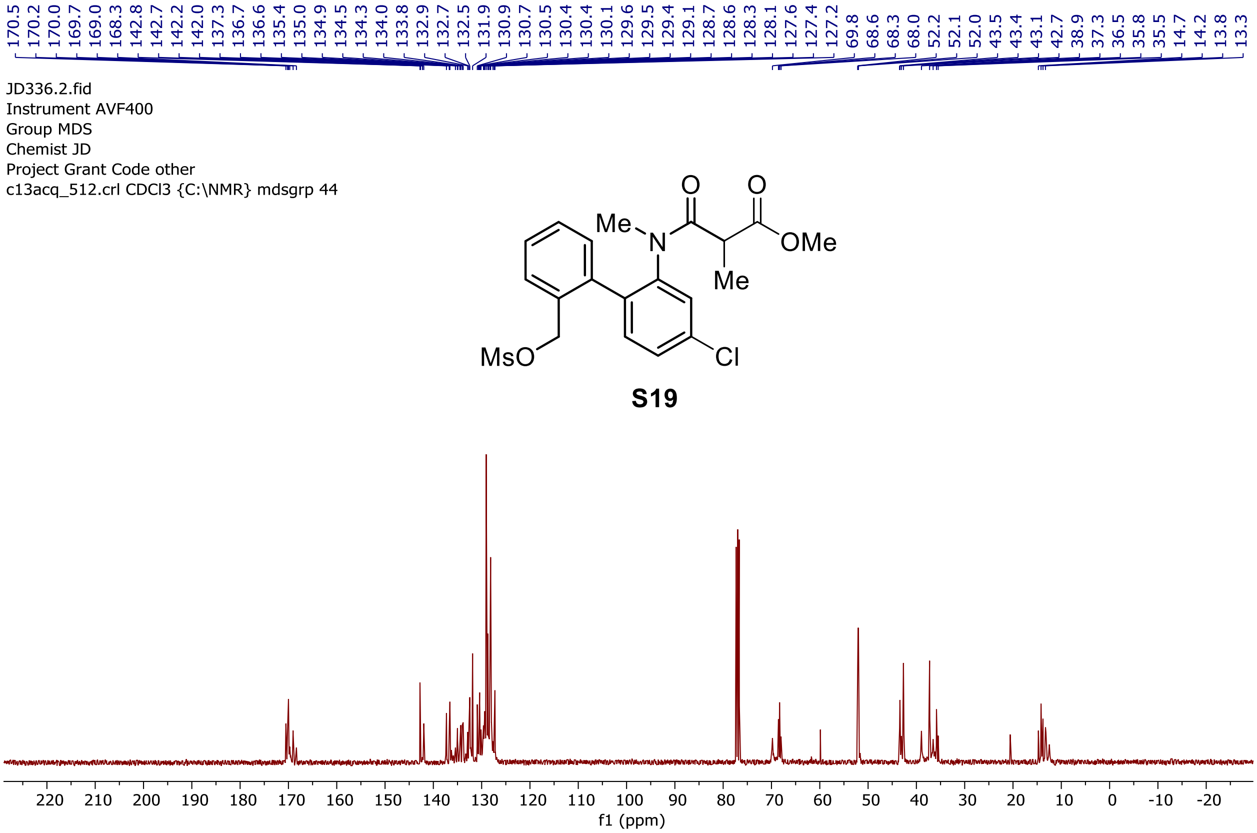
170.5 170.2 170.0 169.7 169.0 168.3 142.8 142.7 142.2 142.0 137.3 136.7 136.6 135.4 135.0 134.9 134.5 134.3 134.0 133.8 132.9 132.7 132.5 131.9 130.9 130.7 130.5 130.4 130.4 130.1 129.6 129.5 129.4 129.1 128.7 128.6 128.3 128.1 127.6 127.4 127.2 69.8 68.6 68.3 68.0 52.2 52.1 52.0 43.5 43.4 43.1 42.7 38.9 37.3 36.5 35.8 35.5 14.7 14.2 13.8 13.3
JD336.2.fid
Instrument AVF400
Group MDS
Chemist JD
Project Grant Code other
c13acq_512.crl CDCl3 {C:\NMR} mdsgrp 44
O
O
Me
N
OMe
Me
MsO
Cl
S19
220 210 200 190 180 170 160 150 140 130 120 110 100 90 80 70 60 50 40 30 20 10 0 -10 -20
f1 (ppm)

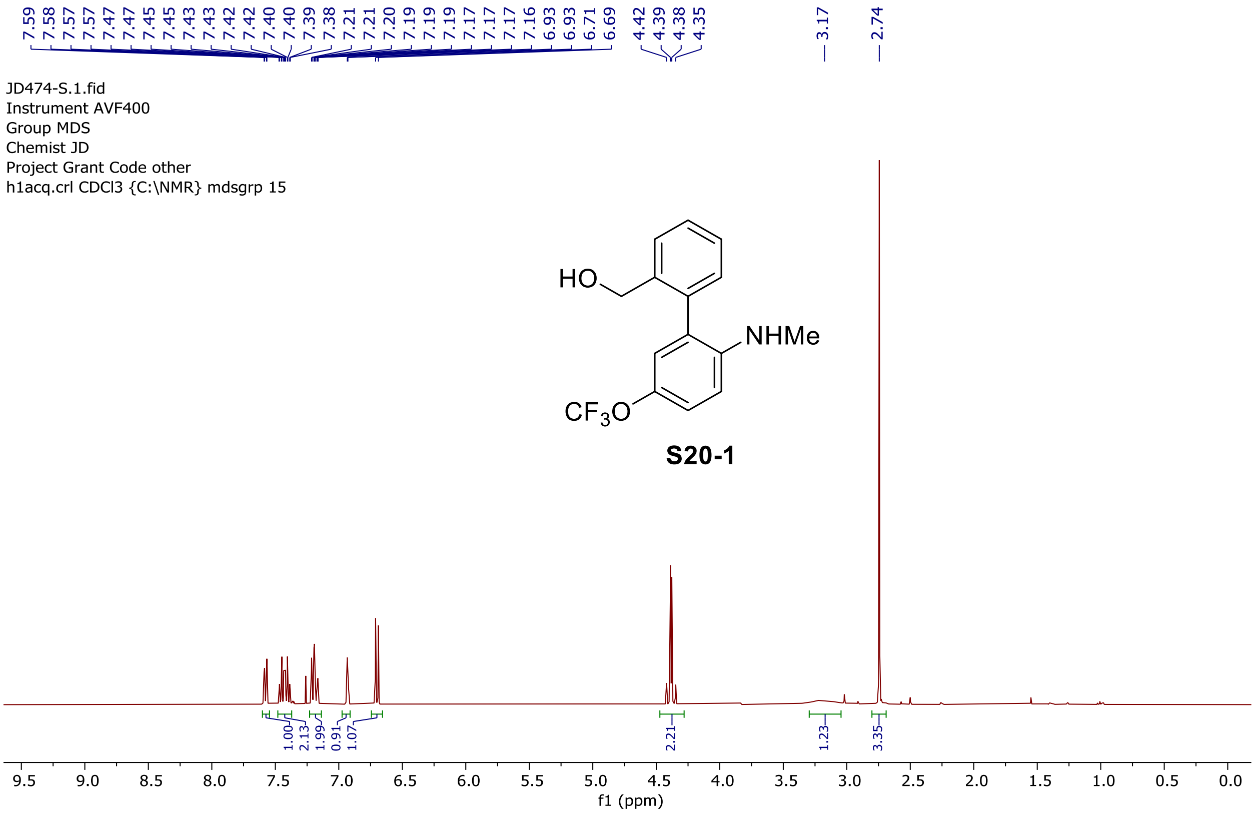
JD474-S.1.fid
Instrument AVF400
Group MDS
Chemist JD
Project Grant Code other
h1acq.crl CDCl3 {C:\NMR} mdsgrp 15
S20-1
f1 (ppm)

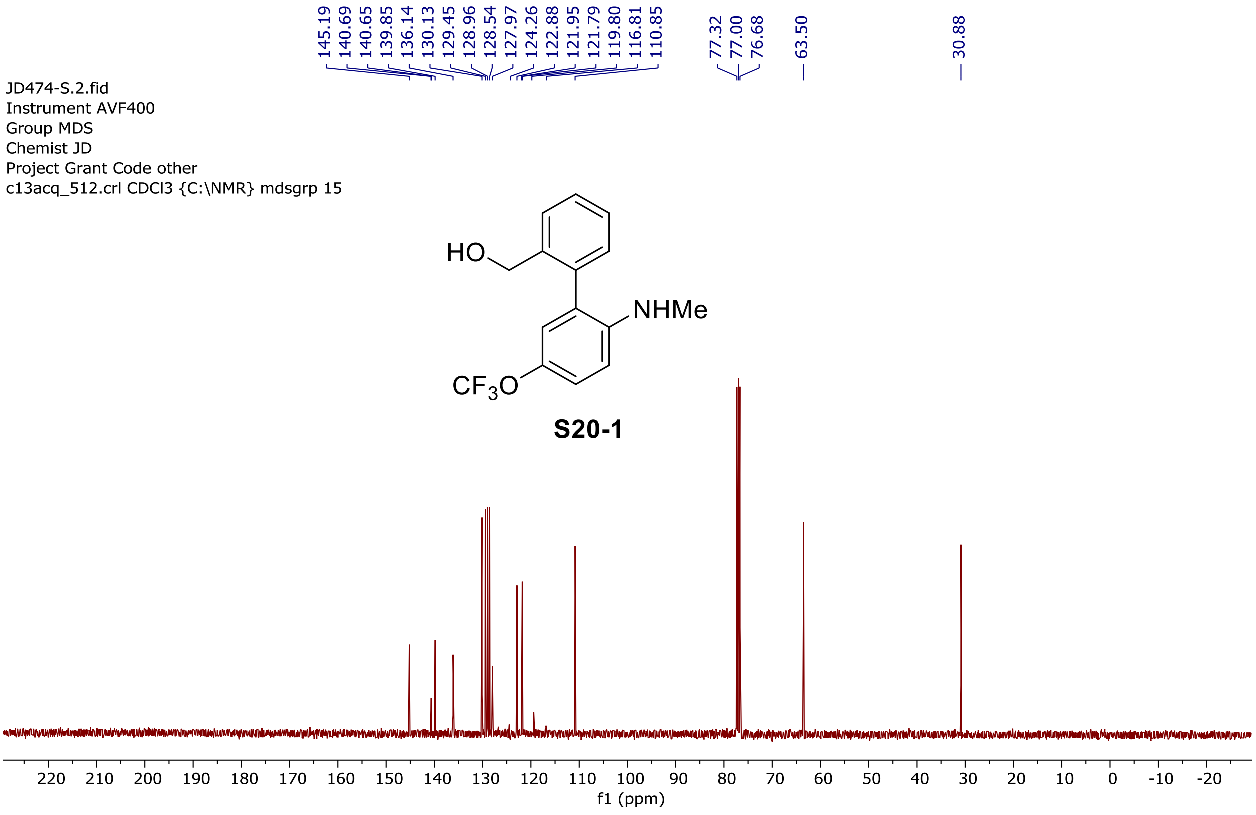
JD474-S.2.fid
Instrument AVF400
Group MDS
Chemist JD
Project Grant Code other
c13acq_512.crl CDCl3 {C:\NMR} mdsgrp 15
S20-1
f1 (ppm)

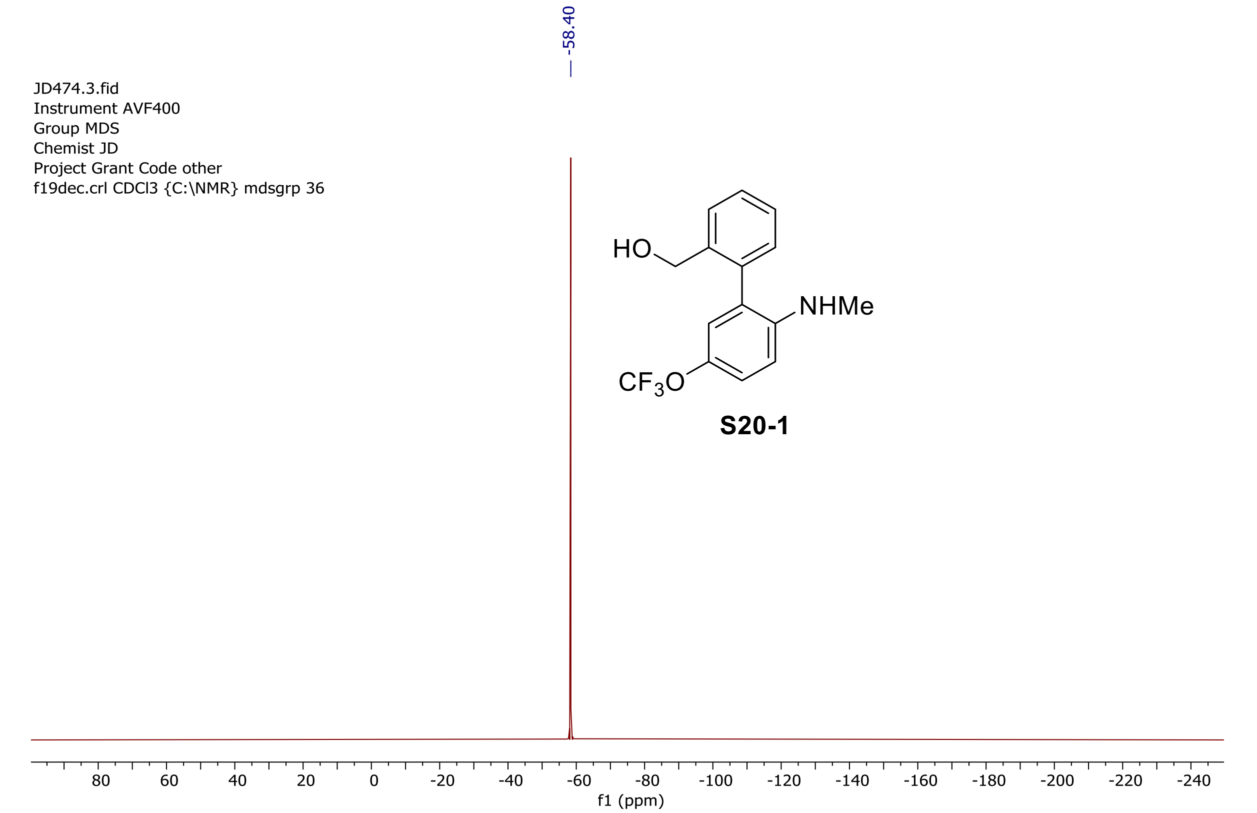
-58.40
JD474.3.fid
Instrument AVF400
Group MDS
Chemist JD
Project Grant Code other
f19dec.crl CDCl3 {C:\NMR} mdsgrp 36
HO
NHMe
CF3O
S20-1
f1 (ppm)

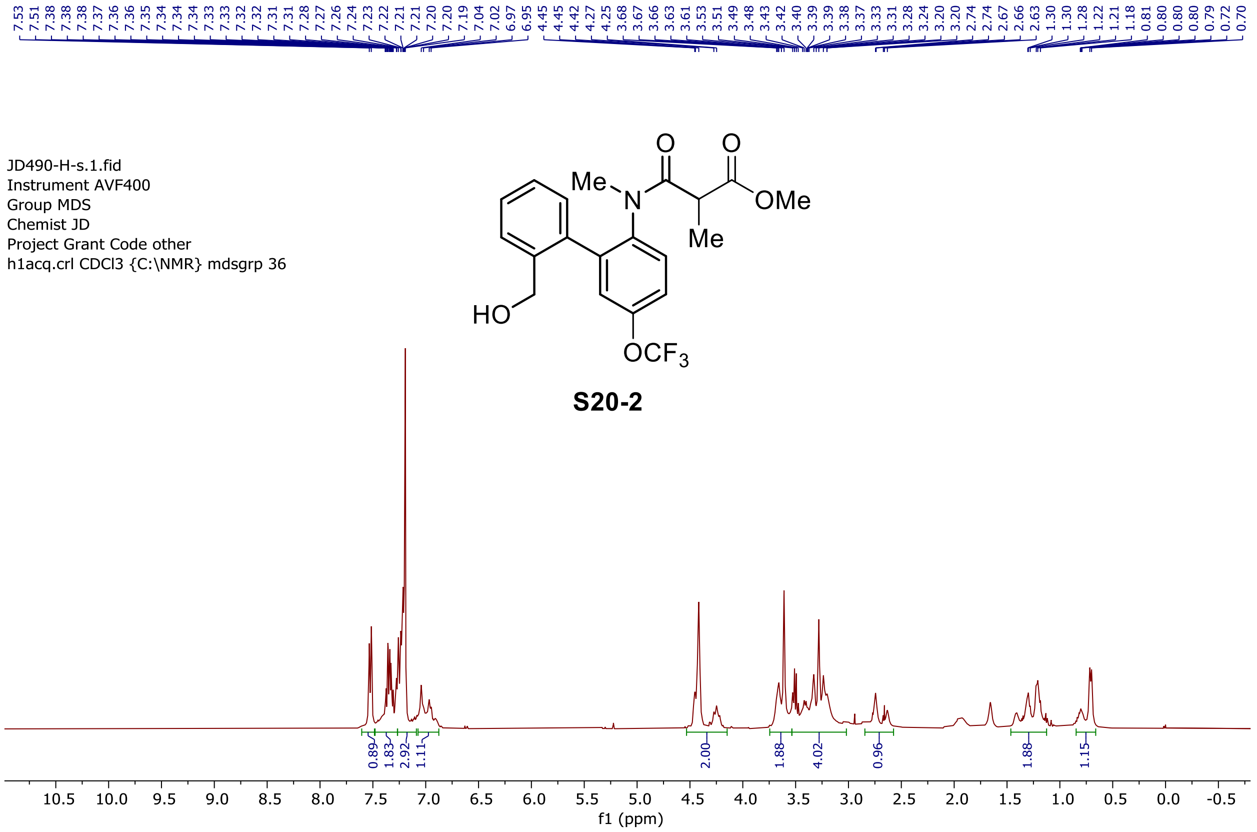
7.53 7.51 7.38 7.38 7.38 7.37 7.36 7.36 7.35 7.34 7.34 7.34 7.33 7.33 7.32 7.32 7.31 7.31 7.28 7.27 7.26 7.24 7.23 7.22 7.21 7.21 7.20 7.20 7.19 7.04 7.02 6.97 6.95 4.45 4.42 4.27 4.25 3.68 3.67 3.66 3.63 3.61 3.53 3.51 3.49 3.48 3.43 3.42 3.40 3.39 3.39 3.38 3.37 3.33 3.31 3.28 3.24 3.20 3.20 2.74 2.74 2.67 2.66 2.63 1.30 1.30 1.28 1.22 1.21 1.18 0.81 0.80 0.80 0.80 0.79 0.72 0.70
JD490-H-s.1.fid
Instrument AVF400
Group MDS
Chemist JD
Project Grant Code other
h1acq.crl CDCl3 {C:\NMR} mdsgrp 36
Me
N
O
O
OMe
Me
HO
OCF3
S20-2
0.89 1.83 2.92 1.11 2.00 1.88 4.02 0.96 1.88 1.15
f1 (ppm)

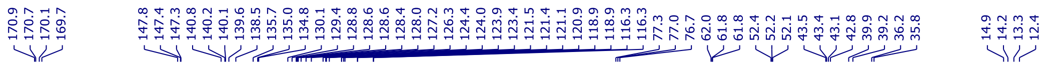

JD490.2.fid
Instrument AVF400
Group MDS
Chemist JD
Project Grant Code other
c13acq_512.crl CDCl3 {C:\NMR} mdsgrp 36

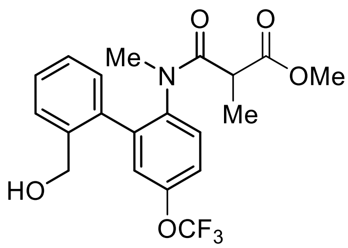

S20-2

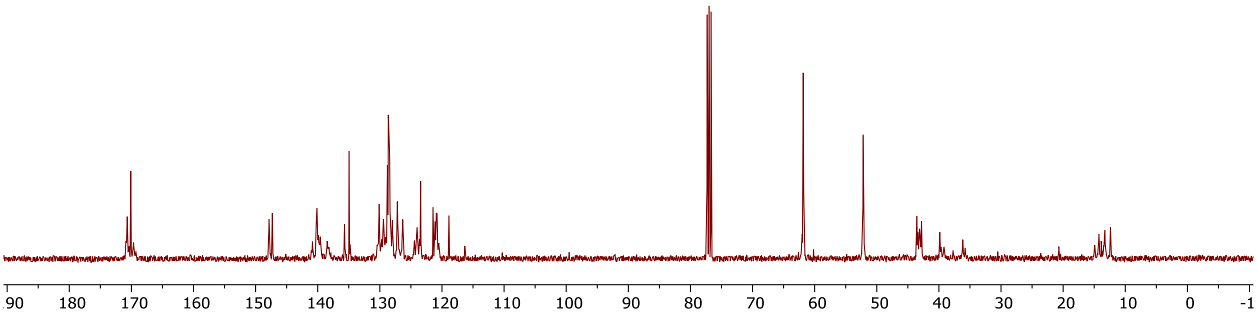

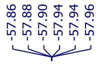

JD490.3.fid
Instrument AVF400
Group MDS
Chemist JD
Project Grant Code other
f19dec.crl CDCl3 {C:\NMR} mdsgrp 36

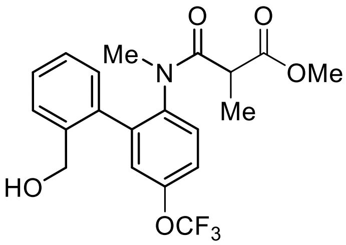

S20-2

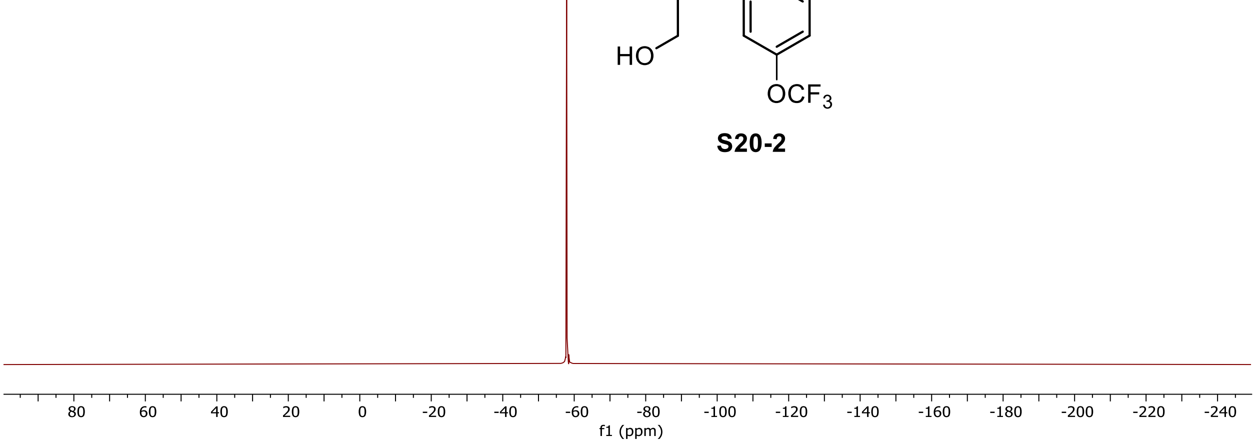

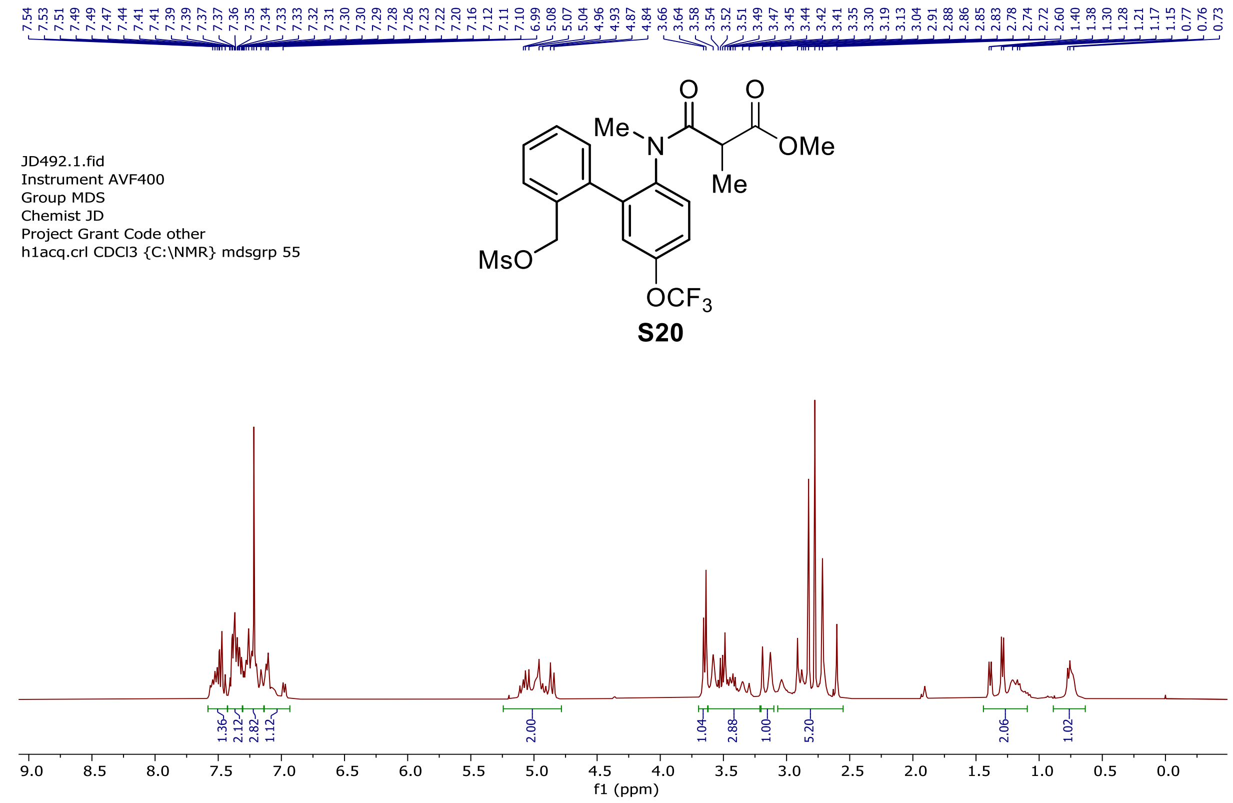
7.54 7.53 7.51 7.49 7.49 7.47 7.44 7.41 7.41 7.39 7.39 7.37 7.37 7.36 7.35 7.34 7.33 7.33 7.32 7.31 7.30 7.29 7.28 7.26 7.23 7.22 7.20 7.16 7.12 7.11 7.10 6.99 5.08 5.07 5.04 4.96 4.93 4.87 4.84 3.66 3.64 3.58 3.54 3.52 3.51 3.49 3.47 3.45 3.44 3.42 3.41 3.35 3.30 3.19 3.13 3.04 2.91 2.88 2.86 2.85 2.83 2.78 2.74 2.72 2.60 1.40 1.38 1.30 1.28 1.21 1.17 1.15 0.77 0.76 0.73
JD492.1.fid
Instrument AVF400
Group MDS
Chemist JD
Project Grant Code other
h1acq.crl CDCl3 {C:\NMR} mdsgrp 55
Me
N
O
O
OMe
Me
MsO
OCF3
S20
1.36 2.12 2.82 1.12 2.00 1.04 2.88 1.00 5.20 2.06 1.02
9.0 8.5 8.0 7.5 7.0 6.5 6.0 5.5 5.0 4.5 4.0 3.5 3.0 2.5 2.0 1.5 1.0 0.5 0.0
f1 (ppm)

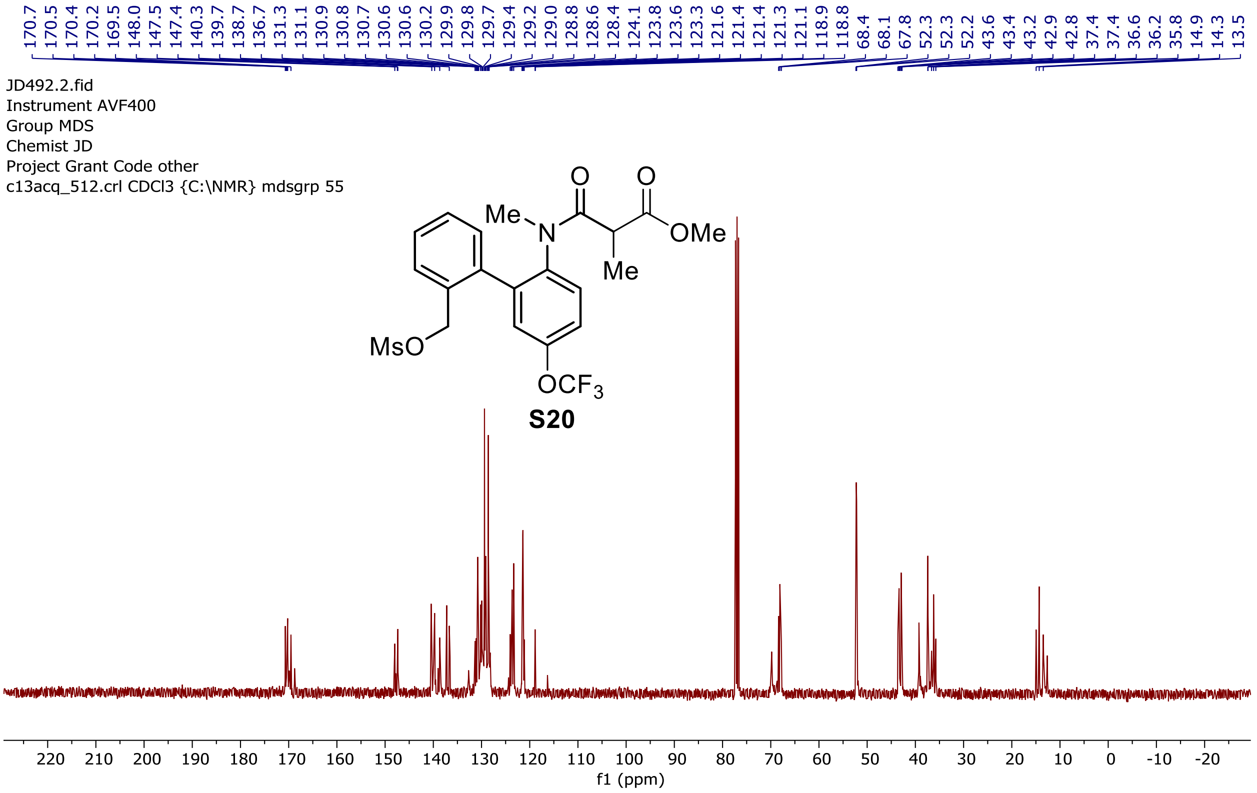
170.7 170.5 170.4 170.2 169.5 148.0 147.5 147.4 140.3 139.7 138.7 136.7 131.3 131.1 130.9 130.8 130.7 130.6 130.2 129.9 129.8 129.7 129.4 129.2 129.0 128.8 128.6 128.4 124.1 123.8 123.6 123.3 121.6 121.4 121.4 121.3 121.1 118.9 118.8 68.4 68.1 67.8 52.3 52.3 52.2 43.6 43.4 43.2 42.9 42.8 37.4 37.4 36.6 36.2 35.8 14.9 14.3 13.5
JD492.2.fid
Instrument AVF400
Group MDS
Chemist JD
Project Grant Code other
c13acq_512.crl CDCl3 {C:\NMR} mdsgrp 55
Me
N
O
O
OMe
Me
MsO
OCF3
S20
220 210 200 190 180 170 160 150 140 130 120 110 100 90 80 70 60 50 40 30 20 10 0 -10 -20
f1 (ppm)

JD492.3.fid
Instrument AVF400
Group MDS
Chemist JD
Project Grant Code other
f19dec.crl CDCl3 {C:\NMR} mdsgrp 55

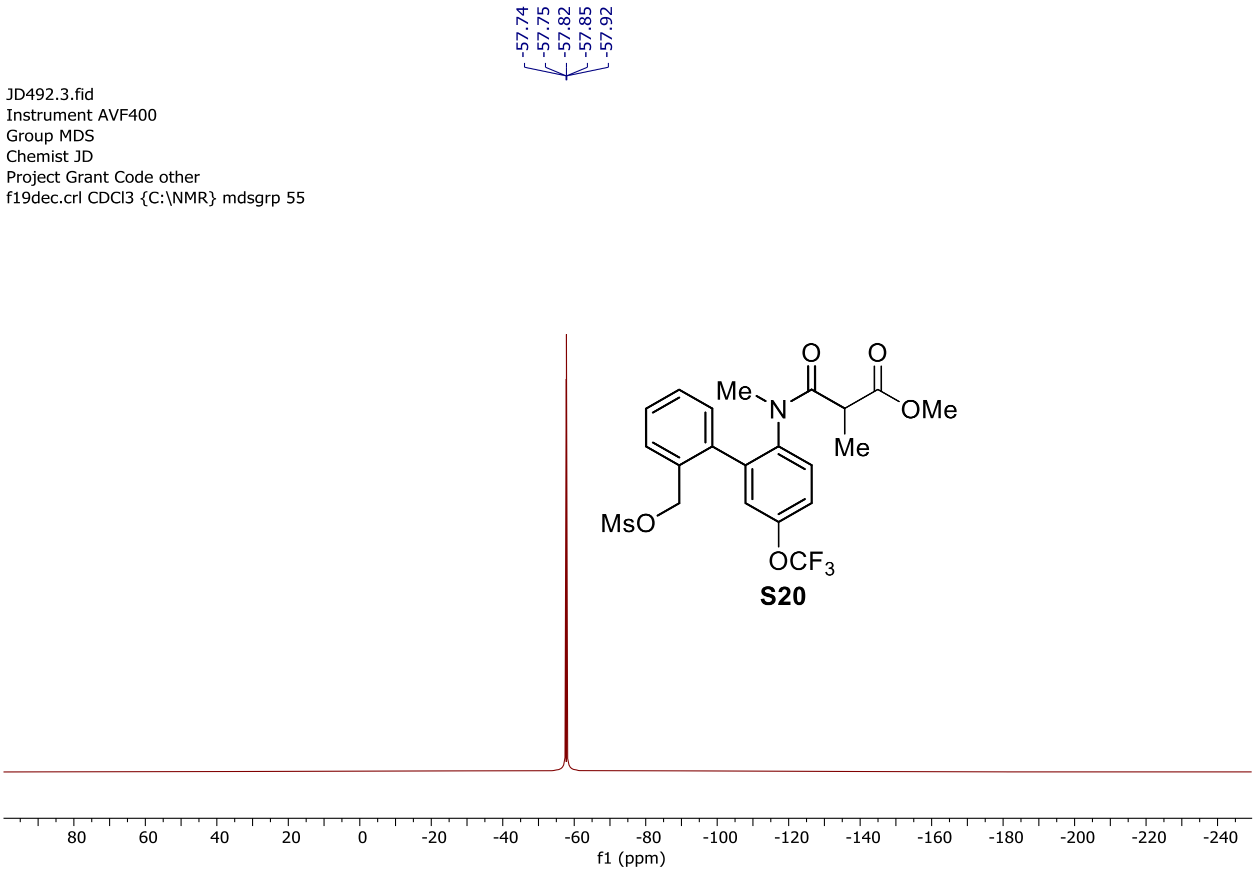

JD302.1.fid
Instrument AVF400
Group MDS
Chemist JD
Project Grant Code other
h1acq.crl CDCl3 {C:\NMR} mdsgrp 30

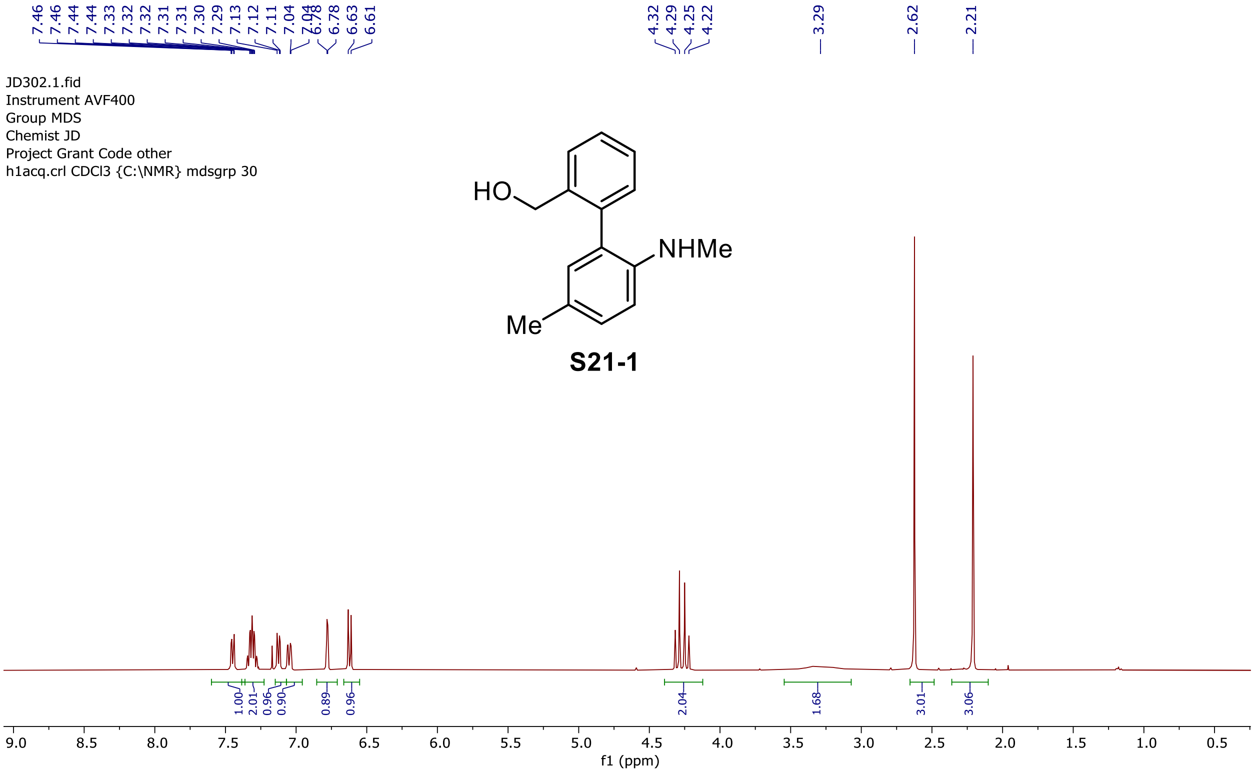

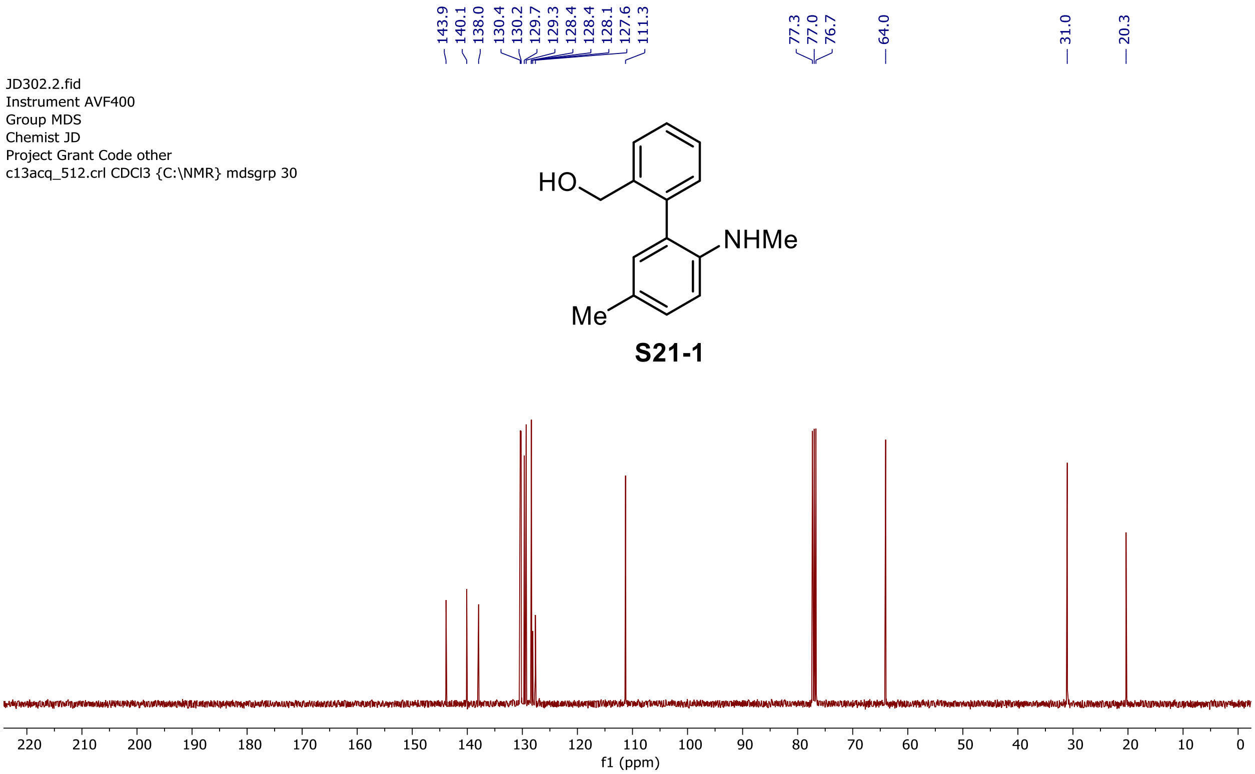
143.9
140.1
138.0
130.4
130.2
129.7
129.3
128.4
128.4
128.1
127.6
111.3
77.3
77.0
76.7
64.0
31.0
20.3
JD302.2.fid
Instrument AVF400
Group MDS
Chemist JD
Project Grant Code other
c13acq_512.crl CDCl3 {C:\NMR} mdsgrp 30
HO
NHMe
Me
220 210 200 190 180 170 160 150 140 130 120 110 100 90 80 70 60 50 40 30 20 10 0
f1 (ppm)

S21-1

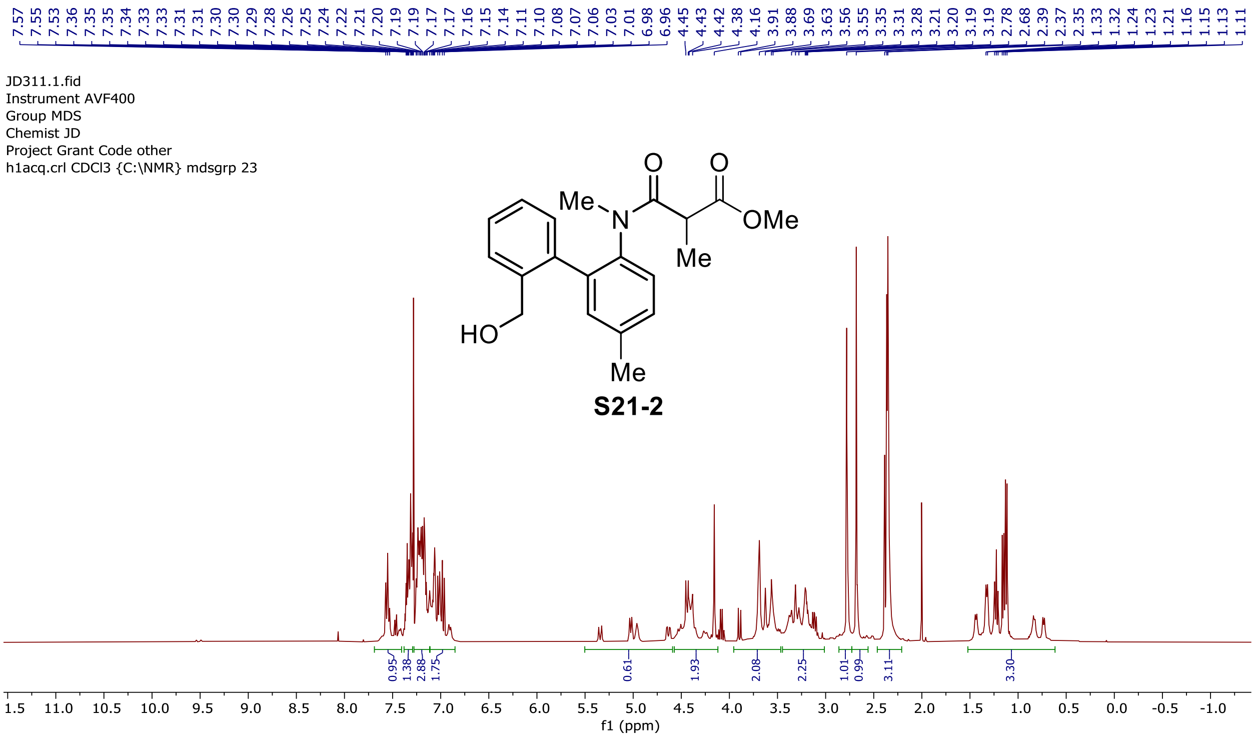
7.57
7.55
7.53
7.36
7.35
7.35
7.34
7.33
7.33
7.31
7.31
7.30
7.30
7.29
7.28
7.26
7.25
7.24
7.22
7.21
7.20
7.19
7.19
7.17
7.17
7.16
7.15
7.14
7.11
7.10
7.08
7.07
7.06
7.03
7.01
6.98
6.96
4.45
4.43
4.42
4.38
4.16
3.91
3.88
3.69
3.63
3.56
3.55
3.35
3.31
3.28
3.21
3.20
3.19
3.19
2.78
2.68
2.39
2.37
2.35
1.33
1.32
1.24
1.23
1.21
1.16
1.15
1.13
1.11
JD311.1.fid
Instrument AVF400
Group MDS
Chemist JD
Project Grant Code other
h1acq.crl CDCl3 {C:\NMR} mdsgrp 23
O
O
Me
N
OMe
Me
HO
Me
0.95
1.38
2.88
1.75
0.61
1.93
2.08
2.25
1.01
0.99
3.11
3.30
1.5 11.0 10.5 10.0 9.5 9.0 8.5 8.0 7.5 7.0 6.5 6.0 5.5 5.0 4.5 4.0 3.5 3.0 2.5 2.0 1.5 1.0 0.5 0.0 -0.5 -1.0
f1 (ppm)

S21-2

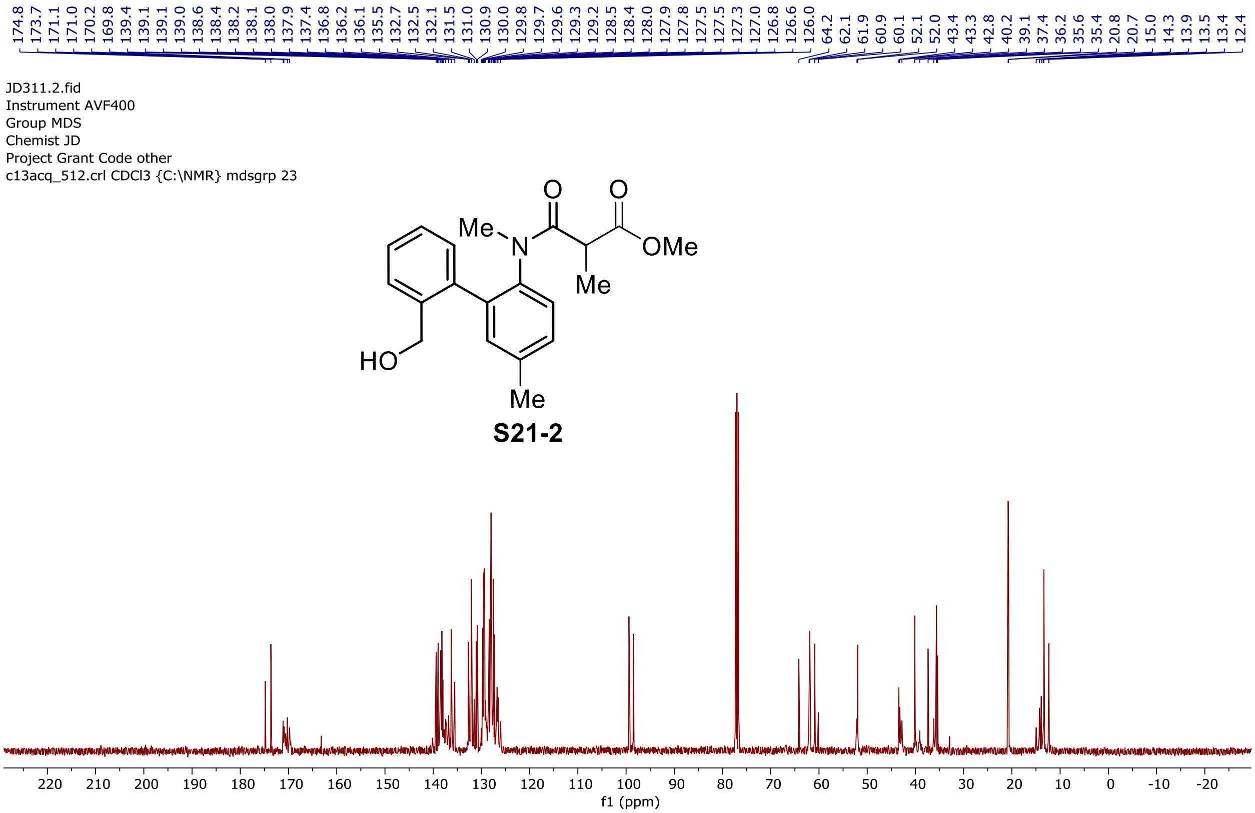
JD311.2.fid
Instrument AVF400
Group MDS
Chemist JD
Project Grant Code other
c13acq_512.crl CDCl3 {C:\NMR} mdsgrp 23
S21-2
f1 (ppm)

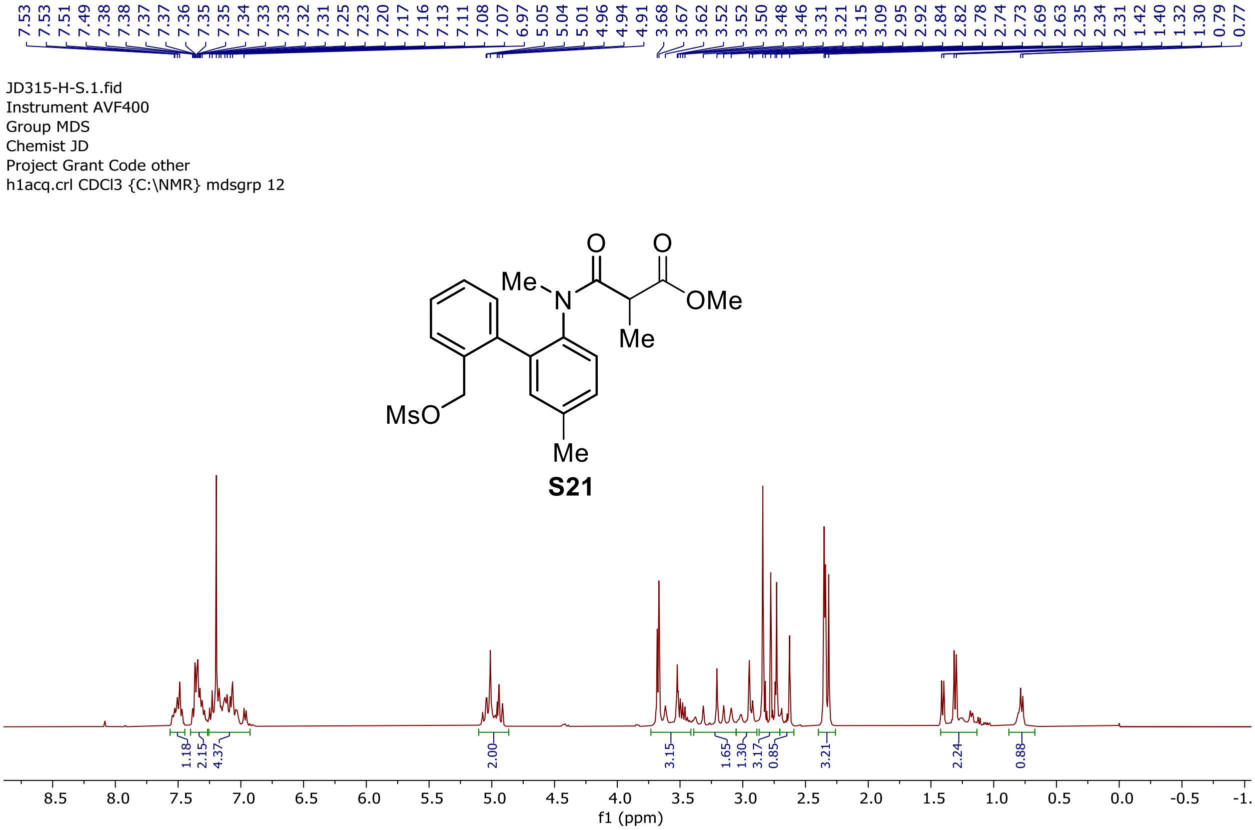
JD315-H-S.1.fid
Instrument AVF400
Group MDS
Chemist JD
Project Grant Code other
h1acq.crl CDCl3 {C:\NMR} mdsgrp 12
S21
f1 (ppm)

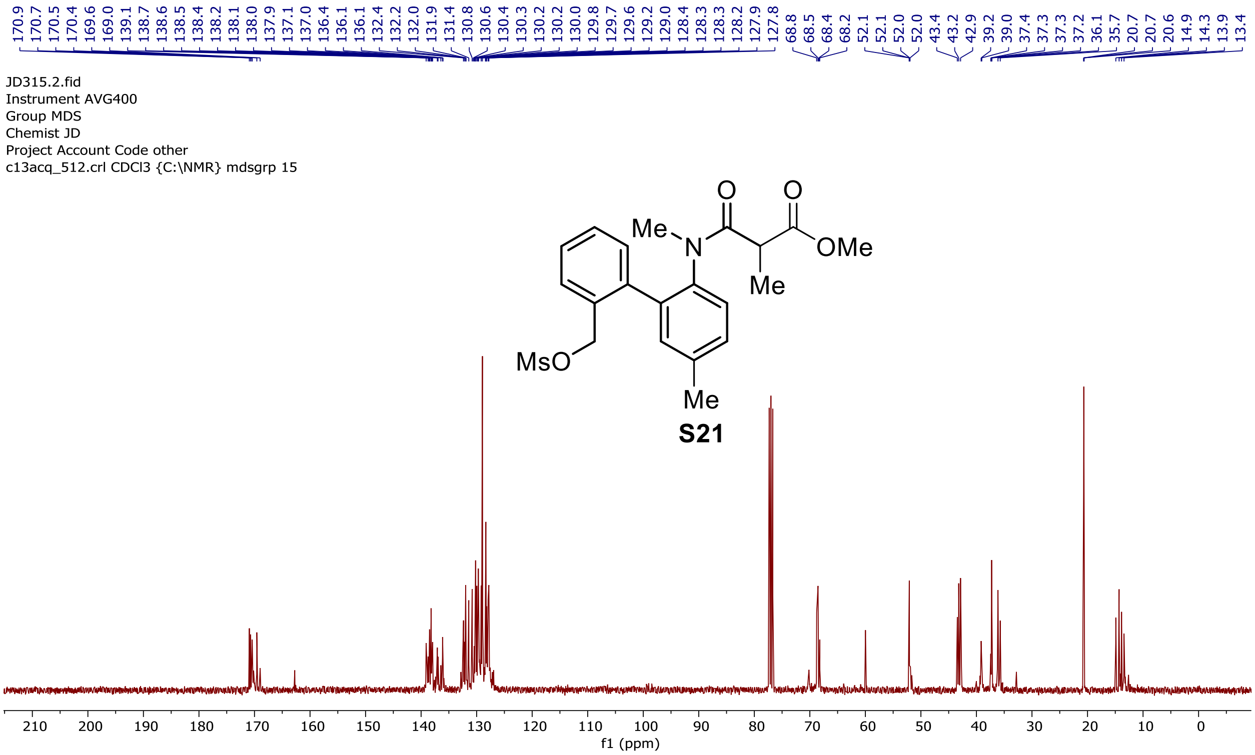
170.9 170.7 170.5 170.4 169.6 169.0 139.1 138.7 138.6 138.5 138.4 138.2 138.1 138.0 137.9 137.1 137.0 136.4 136.1 136.1 132.4 132.2 132.0 131.9 131.4 130.8 130.6 130.4 130.3 130.2 130.2 130.0 129.8 129.7 129.6 129.2 129.0 128.4 128.3 128.3 128.2 127.9 127.8 68.8 68.5 68.4 68.2 52.1 52.1 52.0 52.0 43.4 43.2 42.9 39.2 39.0 37.4 37.3 37.3 37.2 36.1 35.7 20.7 20.6 14.9 14.3 13.9 13.4
JD315.2.fid
Instrument AVG400
Group MDS
Chemist JD
Project Account Code other
c13acq_512.crl CDCl3 {C:\NMR} mdsgrp 15
Me
N
O
O
OMe
Me
MsO
Me
S21
210 200 190 180 170 160 150 140 130 120 110 100 90 80 70 60 50 40 30 20 10 0
f1 (ppm)

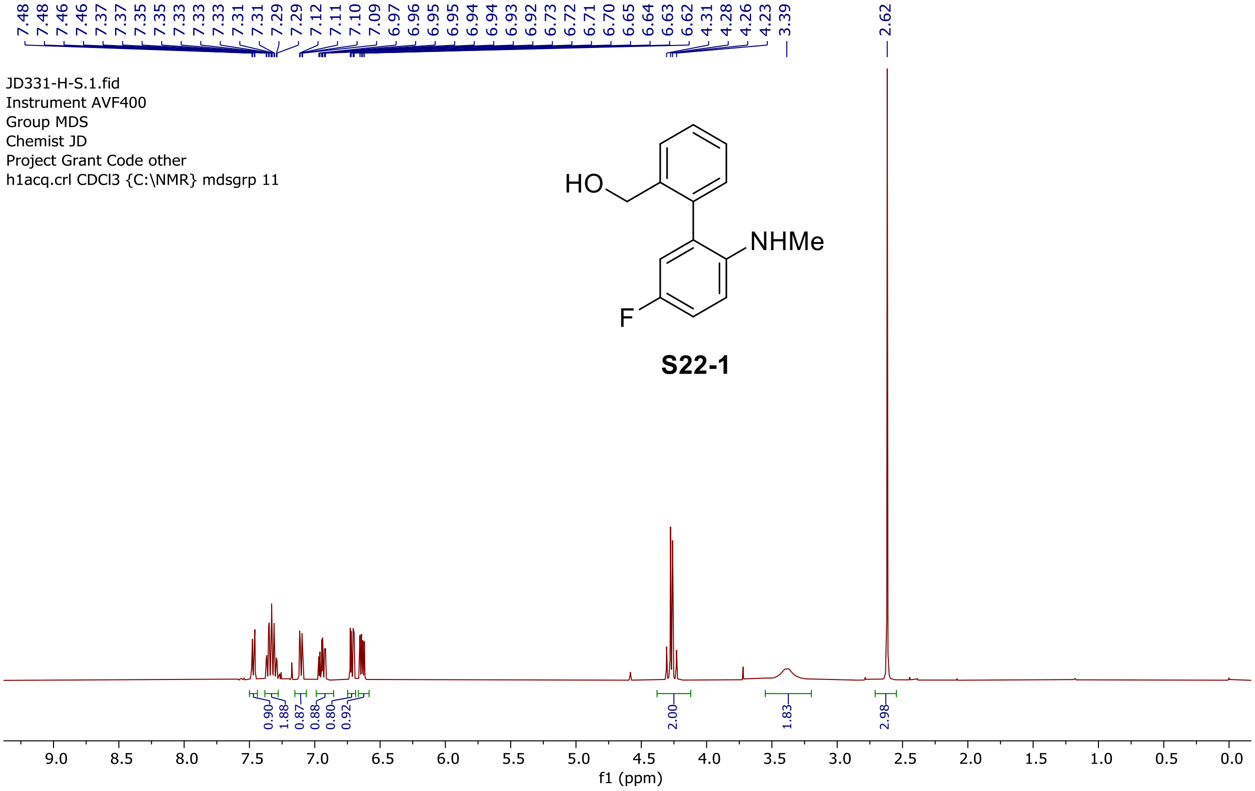
7.48 7.48 7.46 7.46 7.37 7.37 7.35 7.35 7.33 7.33 7.33 7.31 7.29 7.29 7.12 7.11 7.10 7.09 6.97 6.96 6.95 6.95 6.94 6.94 6.93 6.92 6.73 6.72 6.71 6.70 6.65 6.64 6.63 6.62 4.31 4.28 4.26 4.23 3.39 2.62
JD331-H-S.1.fid
Instrument AVF400
Group MDS
Chemist JD
Project Grant Code other
h1acq.crl CDCl3 {C:\NMR} mdsgrp 11
HO
NHMe
F
S22-1
0.90 1.88 0.87 0.88 0.80 0.92 2.00 1.83 2.98
9.0 8.5 8.0 7.5 7.0 6.5 6.0 5.5 5.0 4.5 4.0 3.5 3.0 2.5 2.0 1.5 1.0 0.5 0.0
f1 (ppm)

JD329-C13-S.1.fid
Instrument AVF400
Group MDS
Chemist JD
Project Grant Code other
c13acq_512.crl CDCl3 {C:\NMR} mdsgrp 11

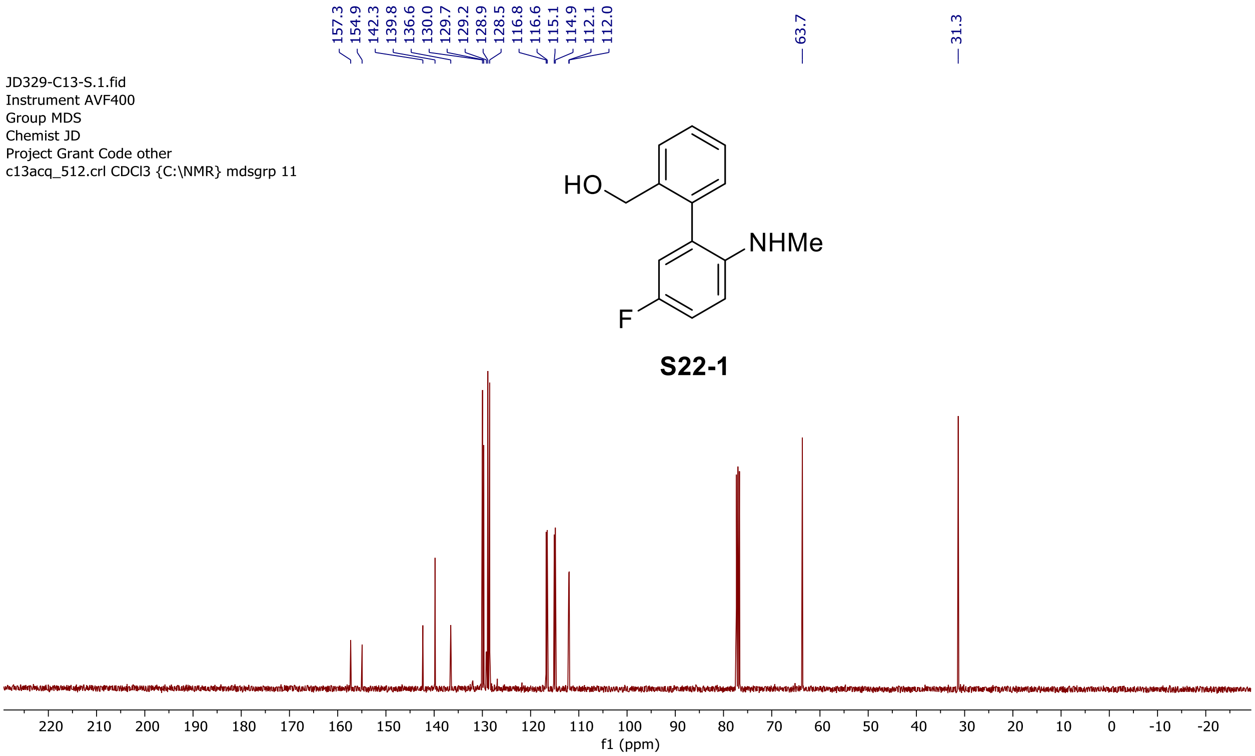

JD329-F.1.fid
Instrument AVF400
Group MDS
Chemist JD
Project Grant Code other
f19acq.crl CDCl3 {C:\NMR} mdsgrp 5

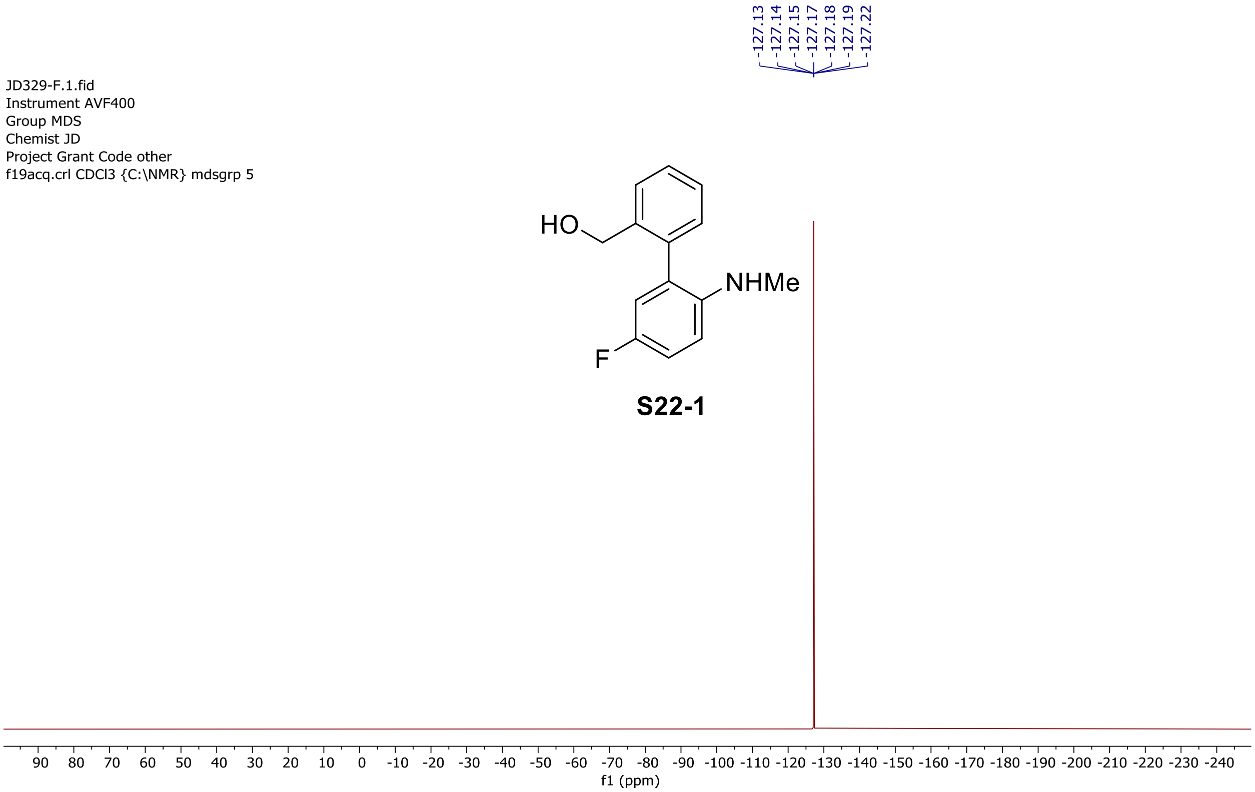

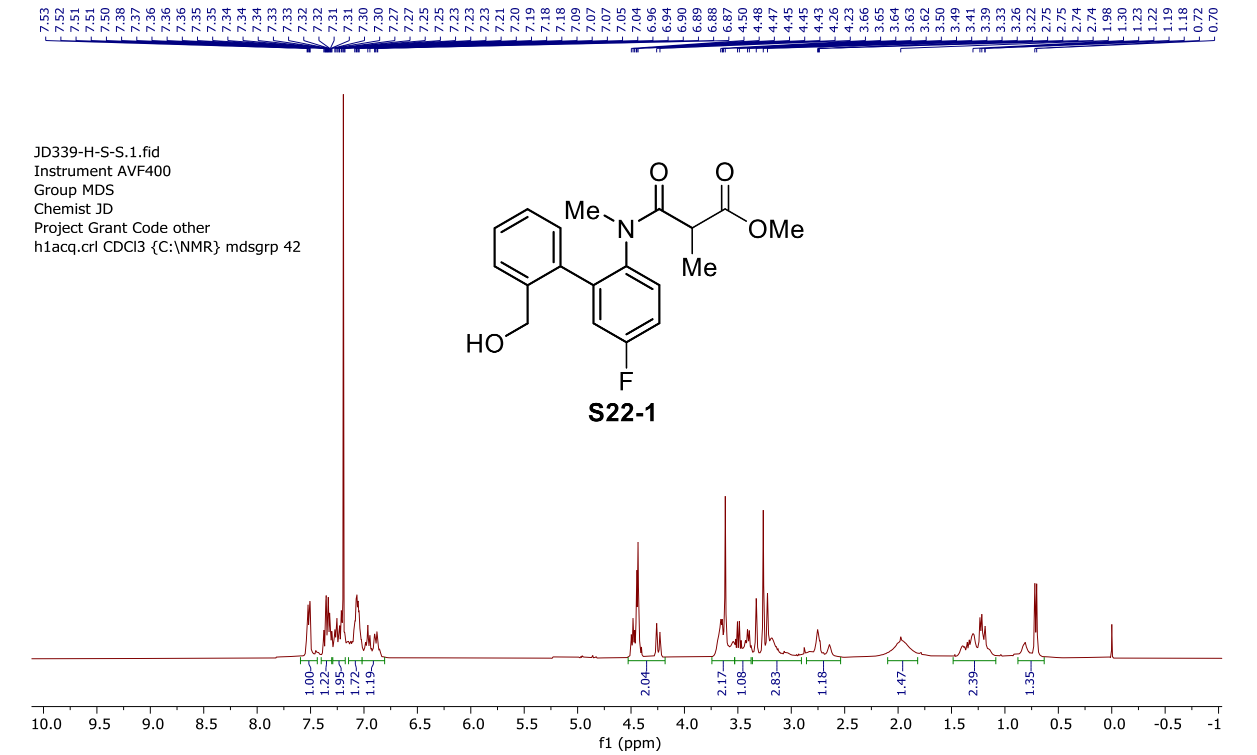
7.53 7.52 7.51 7.50 7.38 7.37 7.36 7.36 7.35 7.35 7.34 7.34 7.33 7.33 7.32 7.32 7.31 7.31 7.30 7.27 7.27 7.25 7.25 7.23 7.23 7.23 7.21 7.20 7.19 7.18 7.18 7.09 7.07 7.07 7.05 7.04 6.96 6.94 6.90 6.89 6.88 6.87 4.50 4.48 4.47 4.45 4.45 4.43 4.26 4.23 3.66 3.65 3.64 3.63 3.62 3.50 3.49 3.41 3.39 3.33 3.26 3.22 2.75 2.75 2.74 2.74 1.98 1.30 1.23 1.22 1.19 1.18 0.72 0.70
JD339-H-S-S.1.fid
Instrument AVF400
Group MDS
Chemist JD
Project Grant Code other
h1acq.crl CDCl3 {C:\NMR} mdsgrp 42
Me
N
O
O
OMe
Me
HO
F
S22-1
1.00 1.22 1.95 1.72 1.19 2.04 2.17 1.08 2.83 1.18 1.47 2.39 1.35
f1 (ppm)

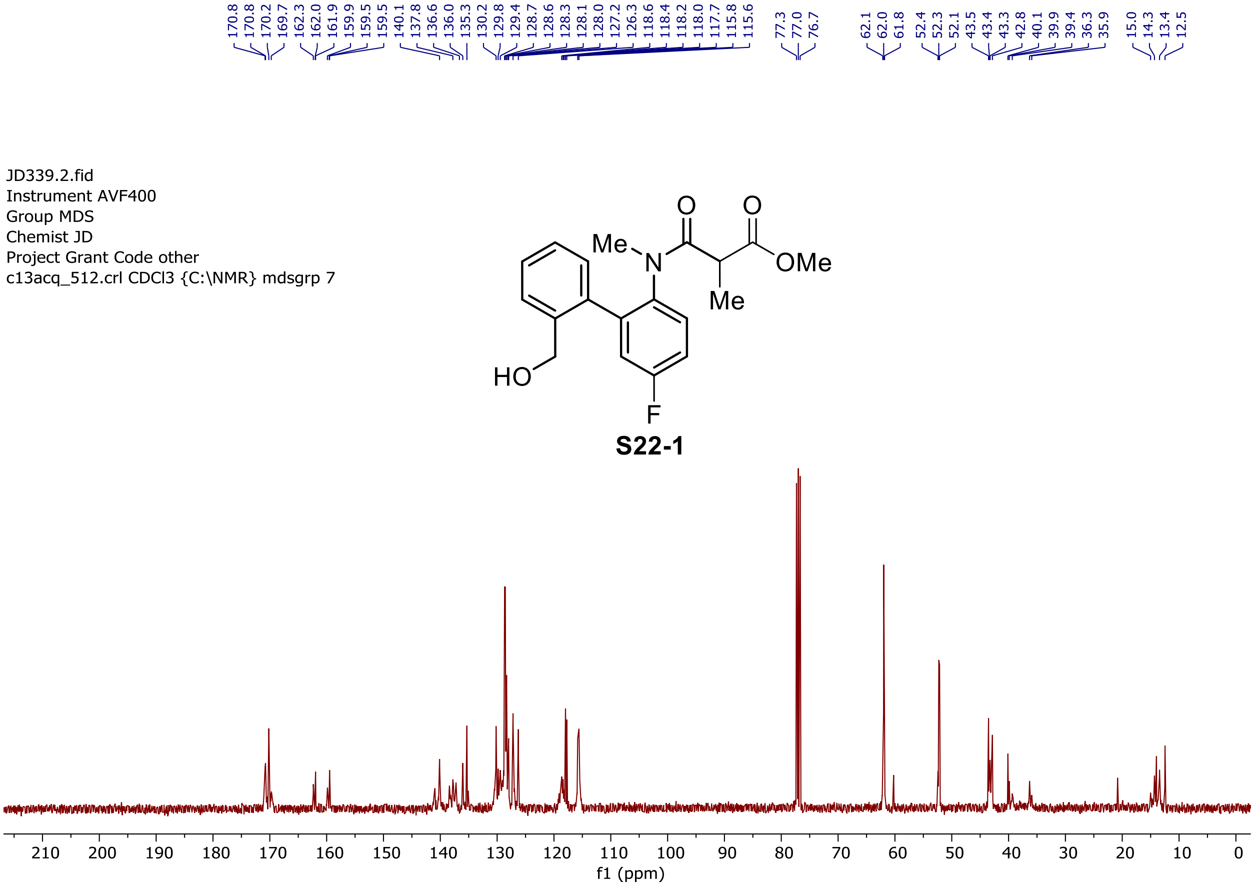
170.8 170.8 170.2 169.7 162.3 162.0 161.9 159.9 159.5 159.5 140.1 137.8 136.6 136.0 135.3 130.2 129.8 129.4 128.7 128.6 128.3 128.1 128.0 127.2 126.3 118.6 118.4 118.2 118.0 117.7 115.8 115.6 77.3 77.0 76.7 62.1 62.0 61.8 52.4 52.3 52.1 43.5 43.4 43.3 42.8 40.1 39.9 39.4 36.3 35.9 15.0 14.3 13.4 12.5
JD339.2.fid
Instrument AVF400
Group MDS
Chemist JD
Project Grant Code other
c13acq_512.crl CDCl3 {C:\NMR} mdsgrp 7
Me
N
O
O
OMe
Me
HO
F
S22-1
f1 (ppm)

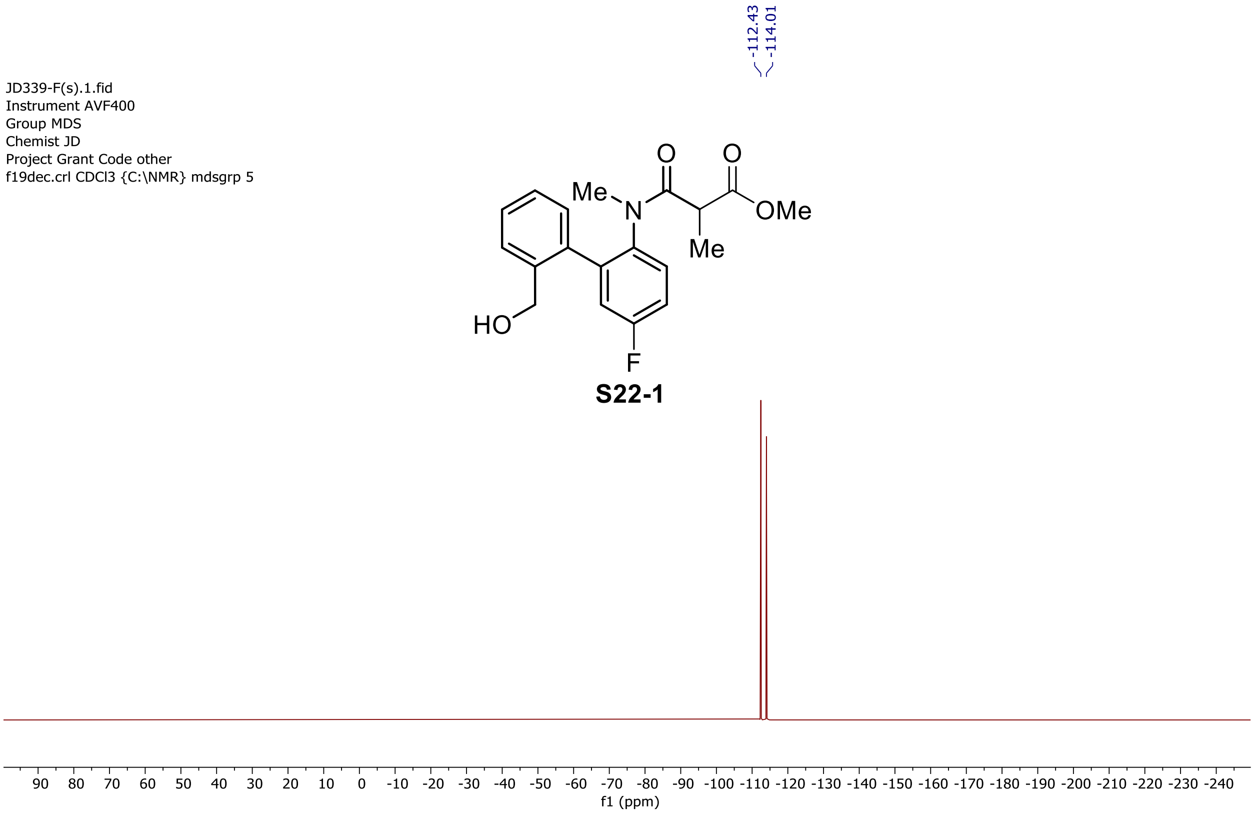
-112.43
-114.01
JD339-F(s).1.fid
Instrument AVF400
Group MDS
Chemist JD
Project Grant Code other
f19dec.crl CDCl3 {C:\NMR} mdsgrp 5
Me
N
O
O
OMe
Me
HO
F
S22-1
90 80 70 60 50 40 30 20 10 0 -10 -20 -30 -40 -50 -60 -70 -80 -90 -100 -110 -120 -130 -140 -150 -160 -170 -180 -190 -200 -210 -220 -230 -240
f1 (ppm)

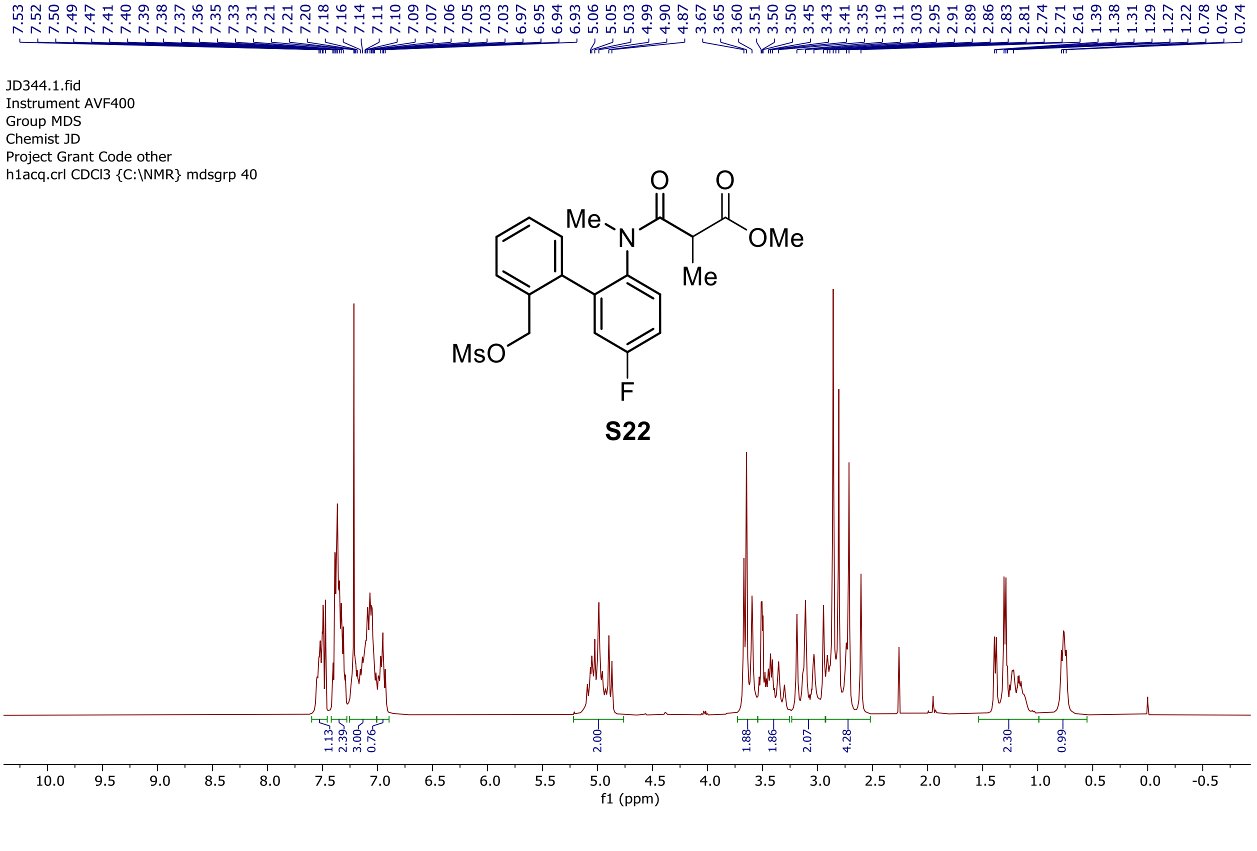
7.53 7.52 7.50 7.49 7.47 7.41 7.40 7.39 7.38 7.37 7.36 7.35 7.33 7.31 7.21 7.21 7.20 7.18 7.16 7.14 7.11 7.10 7.09 7.07 7.06 7.05 7.03 7.03 6.97 6.95 6.94 6.93 5.06 5.05 5.03 4.99 4.90 4.87 3.67 3.65 3.60 3.51 3.50 3.50 3.45 3.43 3.41 3.35 3.19 3.11 3.03 2.95 2.91 2.89 2.86 2.83 2.81 2.74 2.71 2.61 1.39 1.38 1.31 1.29 1.27 1.22 0.78 0.76 0.74
JD344.1.fid
Instrument AVF400
Group MDS
Chemist JD
Project Grant Code other
h1acq.crl CDCl3 {C:\NMR} mdsgrp 40
Me
N
O
O
OMe
Me
MsO
F
S22
1.13 2.39 3.00 0.76 2.00 1.88 1.86 2.07 4.28 2.30 0.99
10.0 9.5 9.0 8.5 8.0 7.5 7.0 6.5 6.0 5.5 5.0 4.5 4.0 3.5 3.0 2.5 2.0 1.5 1.0 0.5 0.0 -0.5
f1 (ppm)

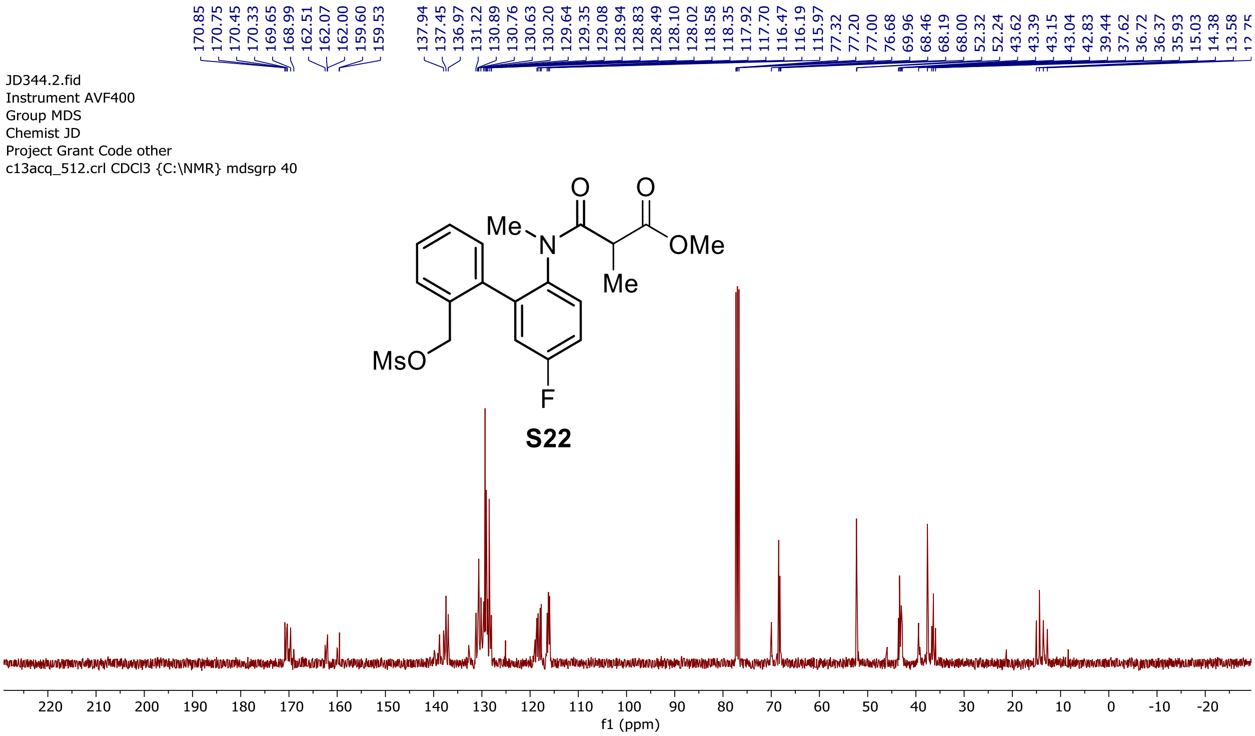
170.85
170.75
170.45
170.33
169.65
168.99
162.51
162.07
162.00
159.60
159.53
137.94
137.45
136.97
131.22
130.89
130.76
130.63
130.20
129.64
129.35
129.08
128.94
128.83
128.49
128.10
128.02
118.58
118.35
117.92
117.70
116.47
116.19
115.97
77.32
77.20
77.00
76.68
69.96
68.46
68.19
68.00
52.32
52.24
43.62
43.39
43.15
43.04
42.83
39.44
37.62
36.72
36.37
35.93
15.03
14.38
13.58
JD344.2.fid
Instrument AVF400
Group MDS
Chemist JD
Project Grant Code other
c13acq_512.crl CDCl3 {C:\NMR} mdsgrp 40
O
O
Me
N
OMe
Me
MsO
F
S22
220 210 200 190 180 170 160 150 140 130 120 110 100 90 80 70 60 50 40 30 20 10 0 -10 -20
f1 (ppm)

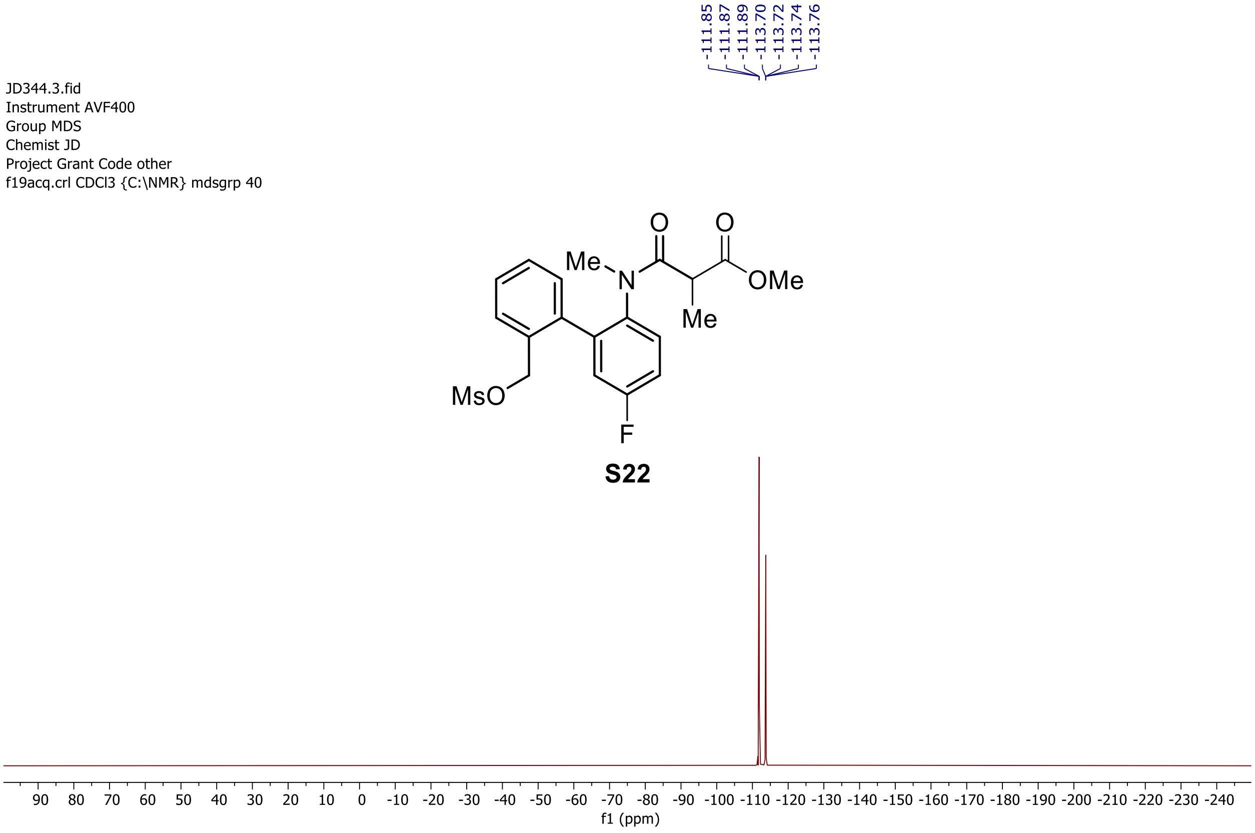
-111.85
-111.87
-111.89
-113.70
-113.72
-113.74
-113.76
JD344.3.fid
Instrument AVF400
Group MDS
Chemist JD
Project Grant Code other
f19acq.crl CDCl3 {C:\NMR} mdsgrp 40
O
O
Me
N
OMe
Me
MsO
F
S22
90 80 70 60 50 40 30 20 10 0 -10 -20 -30 -40 -50 -60 -70 -80 -90 -100 -110 -120 -130 -140 -150 -160 -170 -180 -190 -200 -210 -220 -230 -240
f1 (ppm)

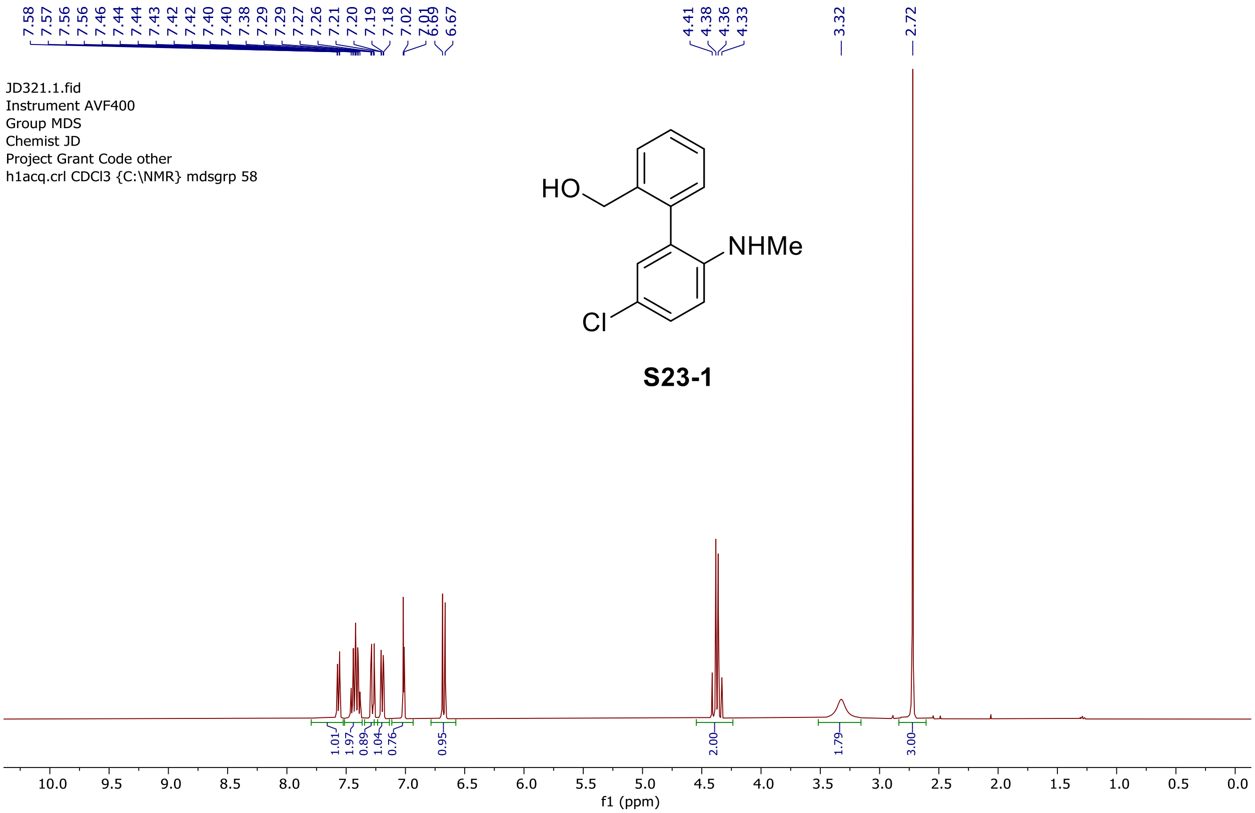
7.58 7.57 7.56 7.56 7.46 7.44 7.44 7.43 7.42 7.42 7.40 7.40 7.38 7.29 7.29 7.27 7.26 7.21 7.20 7.19 7.18 7.02 7.01 6.69 6.67
4.41 4.38 4.36 4.33
3.32
2.72
JD321.1.fid
Instrument AVF400
Group MDS
Chemist JD
Project Grant Code other
h1acq.crl CDCl3 {C:\NMR} mdsgrp 58
HO
NHMe
Cl
S23-1
1.01 1.97 0.89 1.04 0.76 0.95 2.00 1.79 3.00
10.0 9.5 9.0 8.5 8.0 7.5 7.0 6.5 6.0 5.5 5.0 4.5 4.0 3.5 3.0 2.5 2.0 1.5 1.0 0.5 0.0
f1 (ppm)

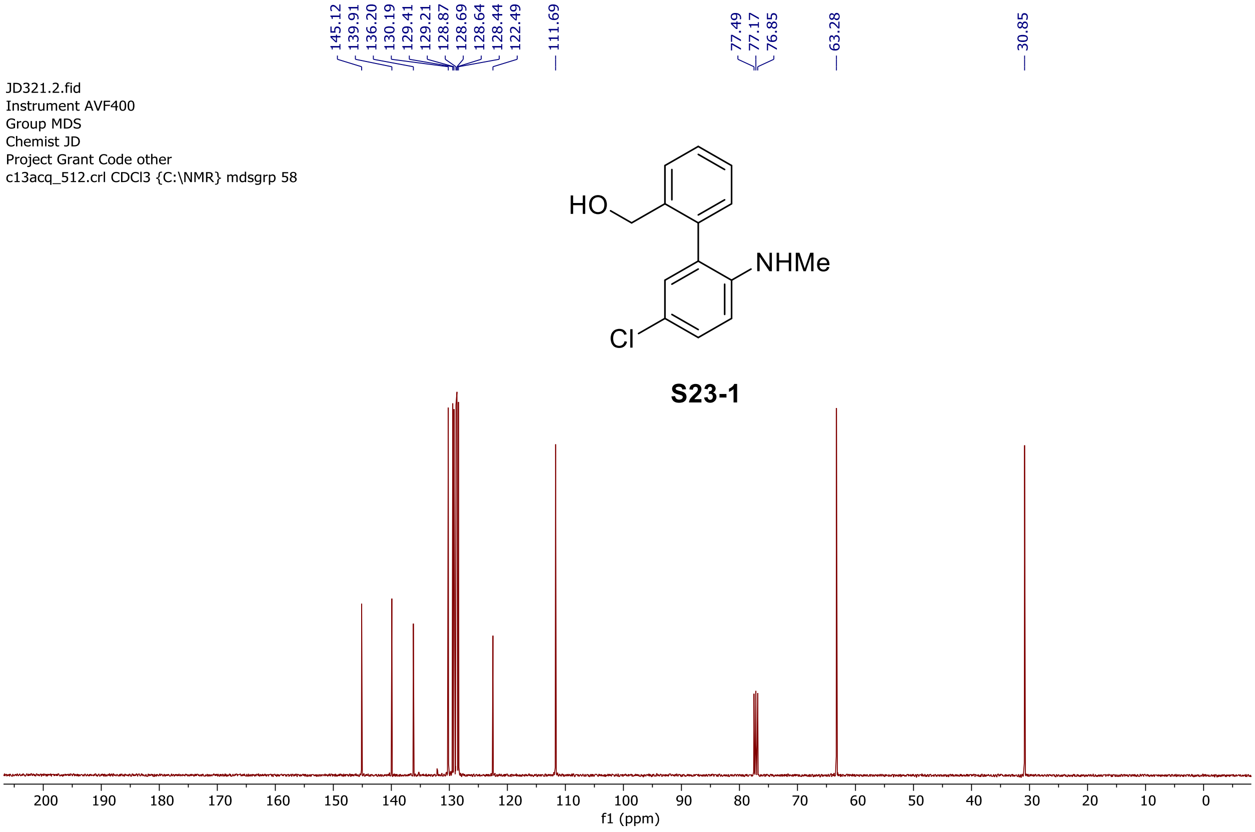
145.12 139.91 136.20 130.19 129.41 129.21 128.87 128.69 128.64 128.44 122.49
111.69
77.49 77.17 76.85
63.28
30.85
JD321.2.fid
Instrument AVF400
Group MDS
Chemist JD
Project Grant Code other
c13acq_512.crl CDCl3 {C:\NMR} mdsgrp 58
HO
NHMe
Cl
S23-1
200 190 180 170 160 150 140 130 120 110 100 90 80 70 60 50 40 30 20 10 0
f1 (ppm)

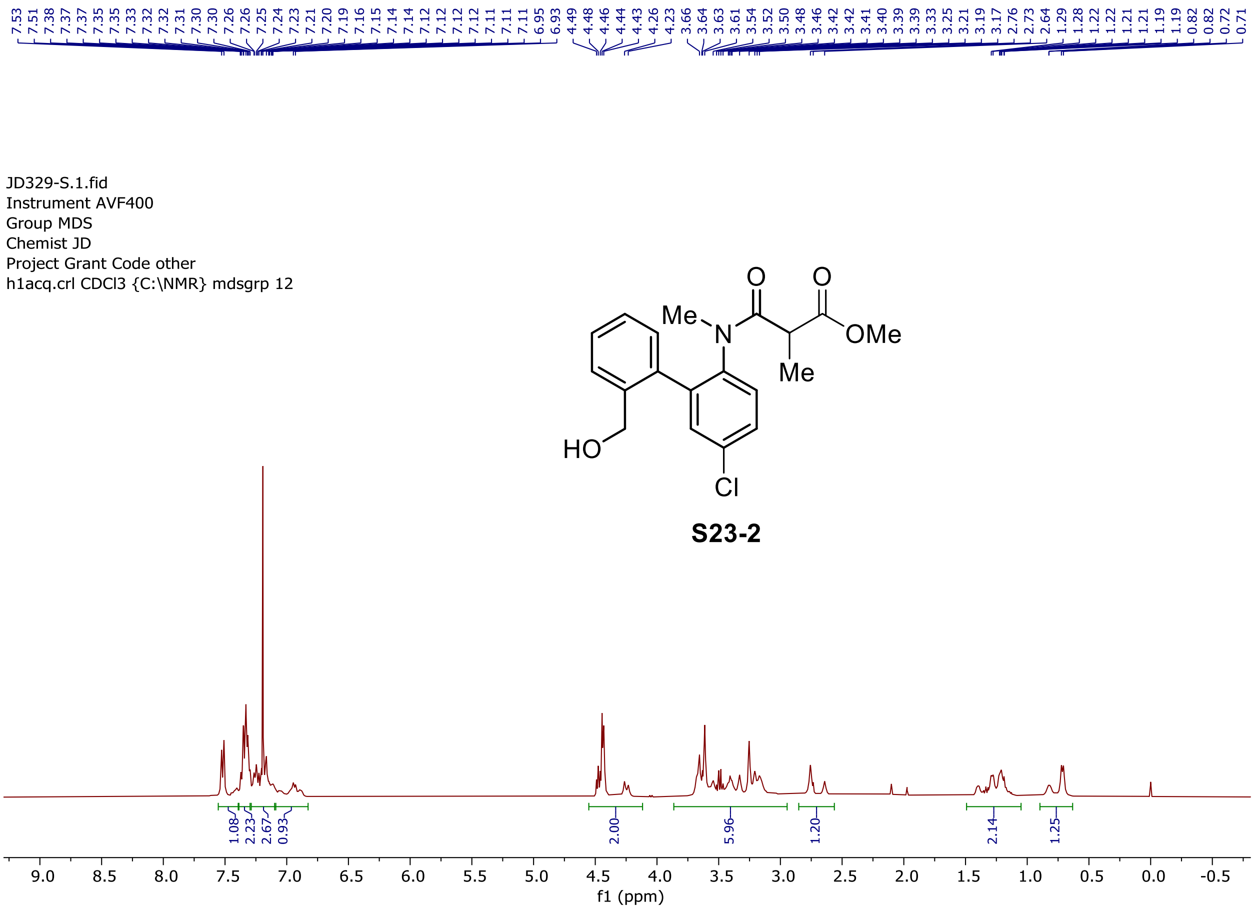
JD329-S.1.fid
Instrument AVF400
Group MDS
Chemist JD
Project Grant Code other
h1acq.crl CDCl3 {C:\NMR} mdsgrp 12
S23-2
f1 (ppm)

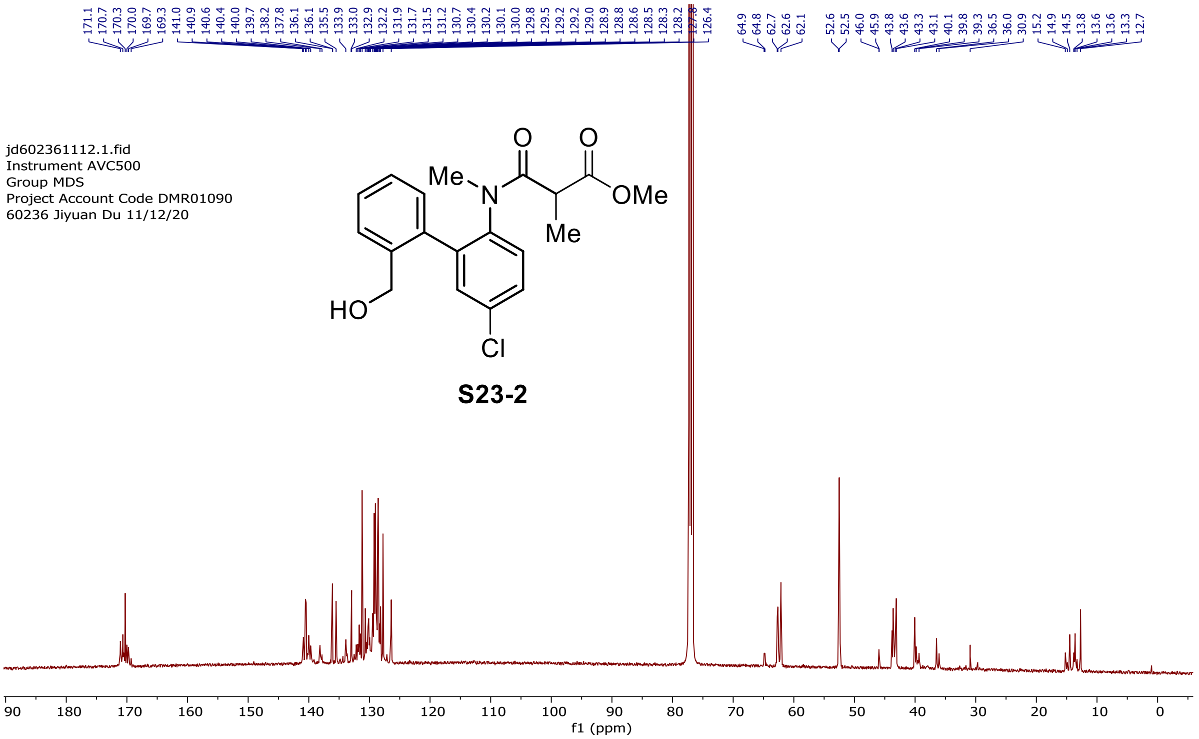
jd602361112.1.fid
Instrument AVC500
Group MDS
Project Account Code DMR01090
60236 Jiyuan Du 11/12/20
S23-2
f1 (ppm)

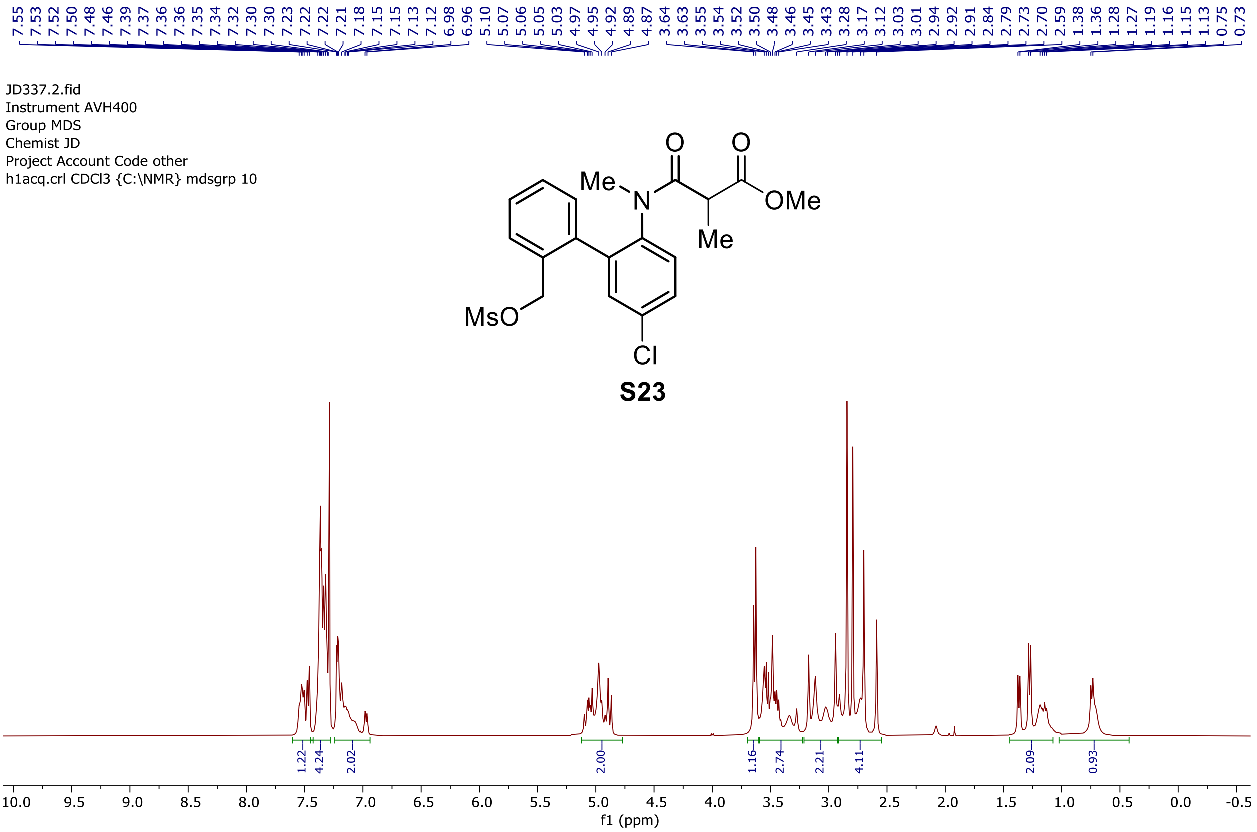
7.55 7.53 7.52 7.50 7.48 7.46 7.39 7.37 7.36 7.36 7.35 7.34 7.32 7.30 7.23 7.22 7.22 7.21 7.18 7.15 7.15 7.13 7.12 6.98 6.96 5.10 5.07 5.06 5.05 5.03 4.97 4.95 4.92 4.89 4.87 3.64 3.63 3.55 3.54 3.52 3.50 3.48 3.46 3.45 3.43 3.28 3.17 3.12 3.03 3.01 2.94 2.92 2.91 2.84 2.79 2.73 2.70 2.59 1.38 1.36 1.28 1.27 1.19 1.16 1.15 1.13 0.75 0.73
JD337.2.fid
Instrument AVH400
Group MDS
Chemist JD
Project Account Code other
h1acq.crl CDCl3 {C:\NMR} mdsgrp 10
Me
N
O
O
OMe
Me
MsO
Cl
S23
1.22 4.24 2.02 2.00 1.16 2.74 2.21 4.11 2.09 0.93
10.0 9.5 9.0 8.5 8.0 7.5 7.0 6.5 6.0 5.5 5.0 4.5 4.0 3.5 3.0 2.5 2.0 1.5 1.0 0.5 0.0 -0.5
f1 (ppm)

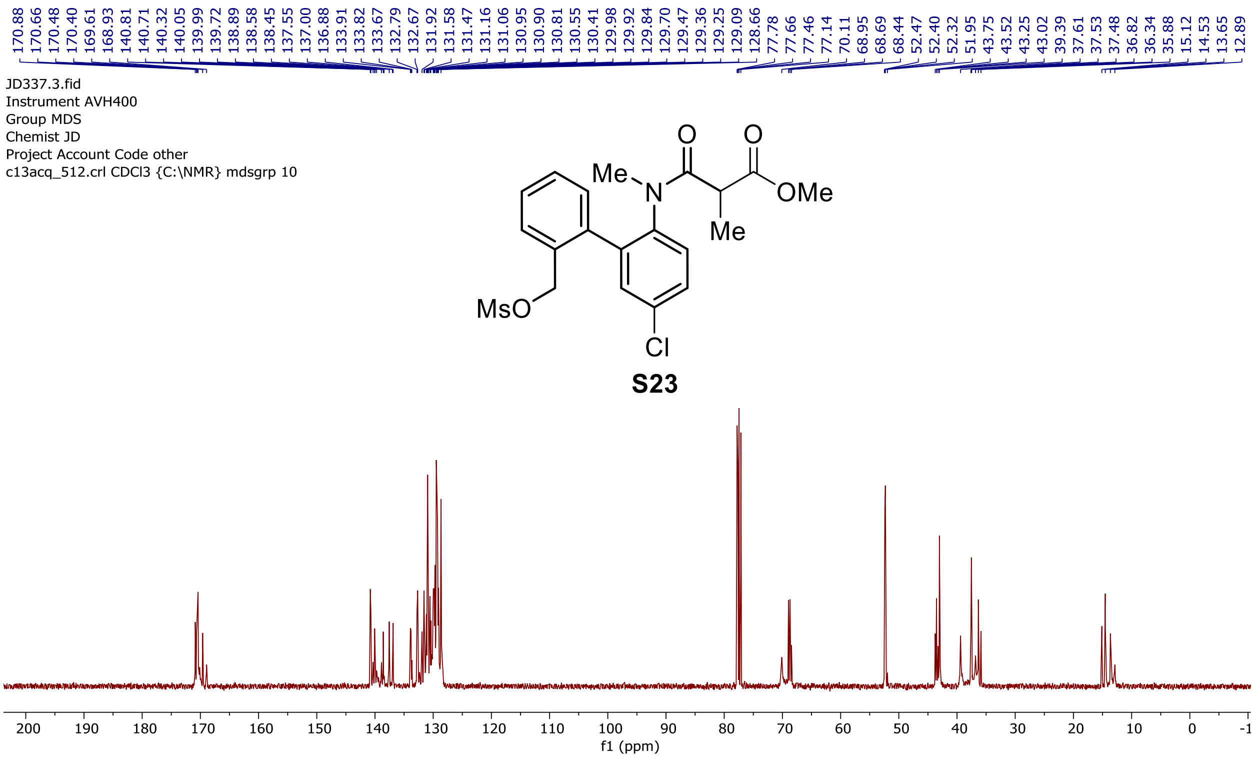
170.88 170.66 170.48 170.40 169.61 168.93 140.81 140.71 140.32 140.05 139.99 139.72 138.89 138.58 138.45 137.55 137.00 136.88 133.91 133.82 133.67 132.79 132.67 131.92 131.58 131.47 131.16 131.06 130.95 130.90 130.81 130.55 130.41 129.98 129.92 129.84 129.70 129.47 129.36 129.25 129.09 128.66 77.78 77.66 77.46 77.14 70.11 68.95 68.69 68.44 52.47 52.40 52.32 51.95 43.75 43.52 43.25 43.02 39.39 37.61 37.53 37.48 36.82 36.34 35.88 15.12 14.53 13.65 12.89
JD337.3.fid
Instrument AVH400
Group MDS
Chemist JD
Project Account Code other
c13acq_512.crl CDCl3 {C:\NMR} mdsgrp 10
Me
N
O
O
OMe
Me
MsO
Cl
S23
200 190 180 170 160 150 140 130 120 110 100 90 80 70 60 50 40 30 20 10 0 -1
f1 (ppm)

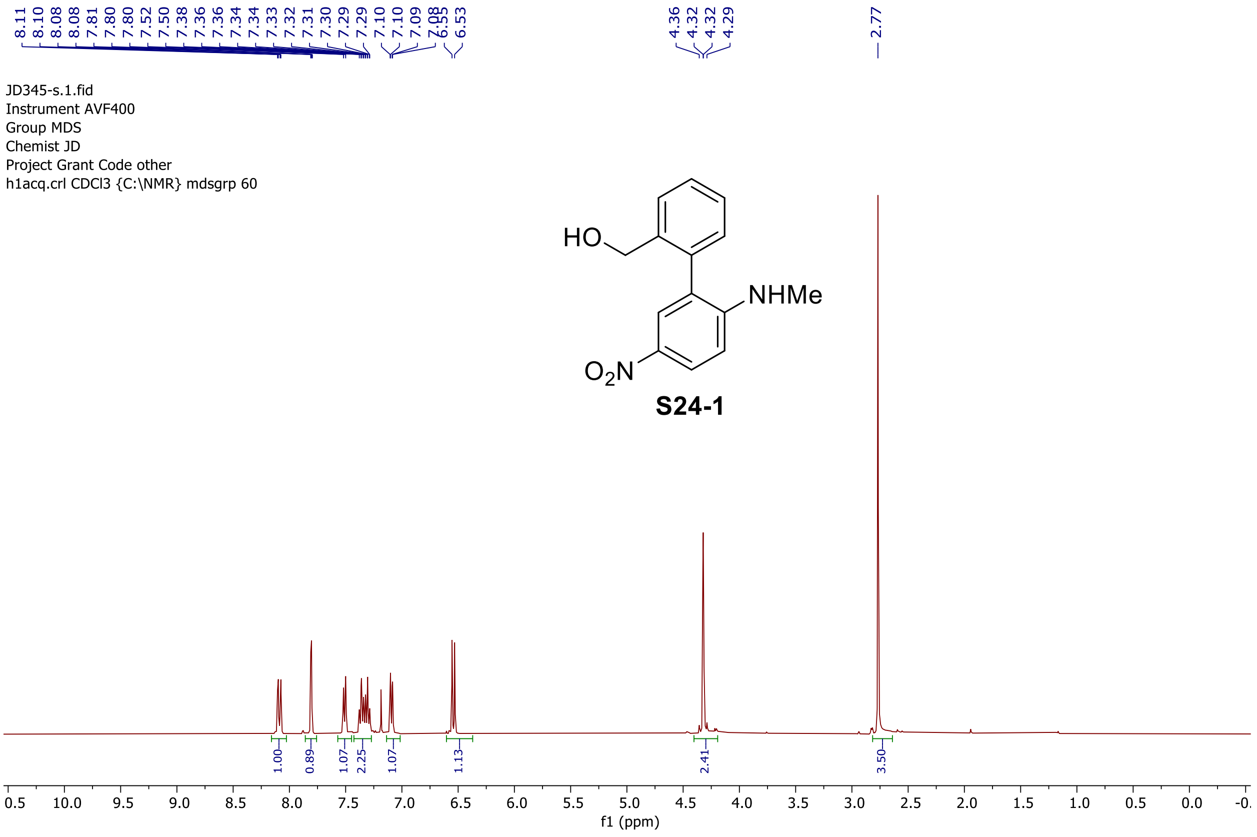
8.11
8.10
8.08
8.08
7.81
7.80
7.80
7.52
7.50
7.38
7.36
7.36
7.34
7.34
7.33
7.32
7.31
7.30
7.29
7.29
7.10
7.10
7.09
7.08
6.55
6.53
4.36
4.32
4.32
4.29
2.77
JD345-s.1.fid
Instrument AVF400
Group MDS
Chemist JD
Project Grant Code other
h1acq.crl CDCl3 {C:\NMR} mdsgrp 60
HO
NHMe
O_2N
S24-1
1.00
0.89
1.07
2.25
1.07
1.13
2.41
3.50
f1 (ppm)

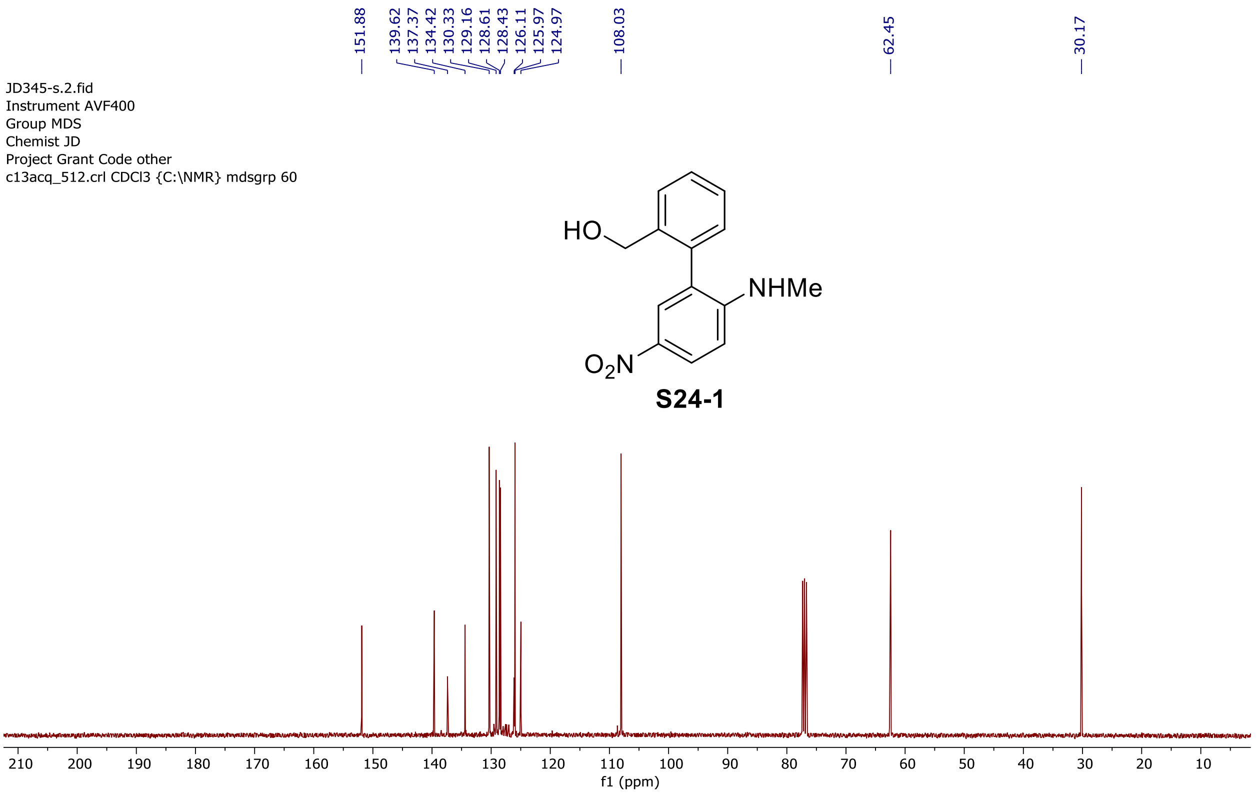
151.88
139.62
137.37
134.42
130.33
129.16
128.61
128.43
126.11
125.97
124.97
108.03
62.45
30.17
JD345-s.2.fid
Instrument AVF400
Group MDS
Chemist JD
Project Grant Code other
c13acq_512.crl CDCl3 {C:\NMR} mdsgrp 60
HO
NHMe
O_2N
S24-1
f1 (ppm)

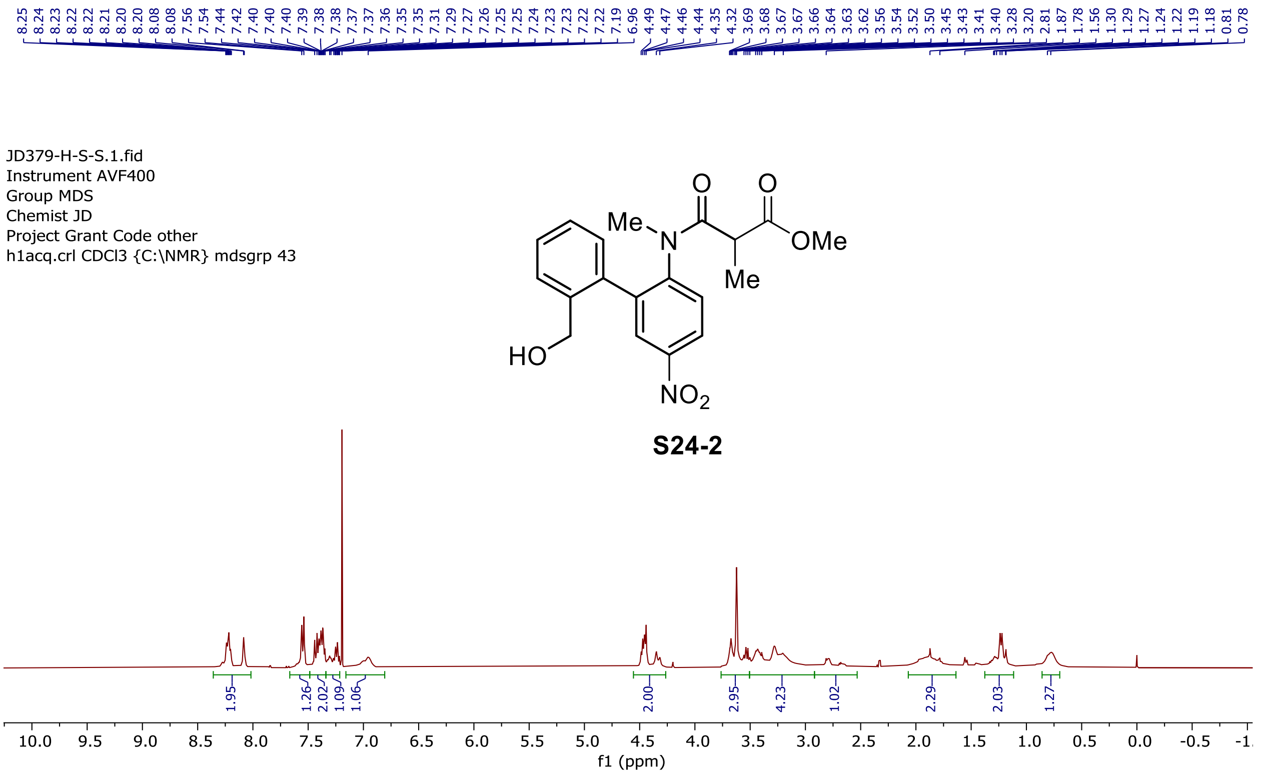
8.25 8.24 8.23 8.22 8.22 8.21 8.20 8.20 8.08 8.08 7.56 7.54 7.44 7.42 7.40 7.40 7.40 7.39 7.38 7.38 7.37 7.36 7.35 7.35 7.31 7.29 7.27 7.26 7.25 7.25 7.24 7.23 7.23 7.22 7.22 7.19 6.96 4.49 4.47 4.46 4.44 4.35 4.32 3.69 3.68 3.67 3.67 3.66 3.64 3.63 3.62 3.56 3.54 3.52 3.50 3.45 3.43 3.41 3.40 3.28 3.20 2.81 1.87 1.78 1.56 1.30 1.29 1.27 1.24 1.22 1.19 1.18 0.81 0.78
JD379-H-S-S.1.fid
Instrument AVF400
Group MDS
Chemist JD
Project Grant Code other
h1acq.crl CDCl3 {C:\NMR} mdsgrp 43
O
O
Me
N
OMe
Me
HO
NO2
S24-2
1.95
1.26
2.02
1.09
1.06
2.00
2.95
4.23
1.02
2.29
2.03
1.27
10.0 9.5 9.0 8.5 8.0 7.5 7.0 6.5 6.0 5.5 5.0 4.5 4.0 3.5 3.0 2.5 2.0 1.5 1.0 0.5 0.0 -0.5 -1.
f1 (ppm)

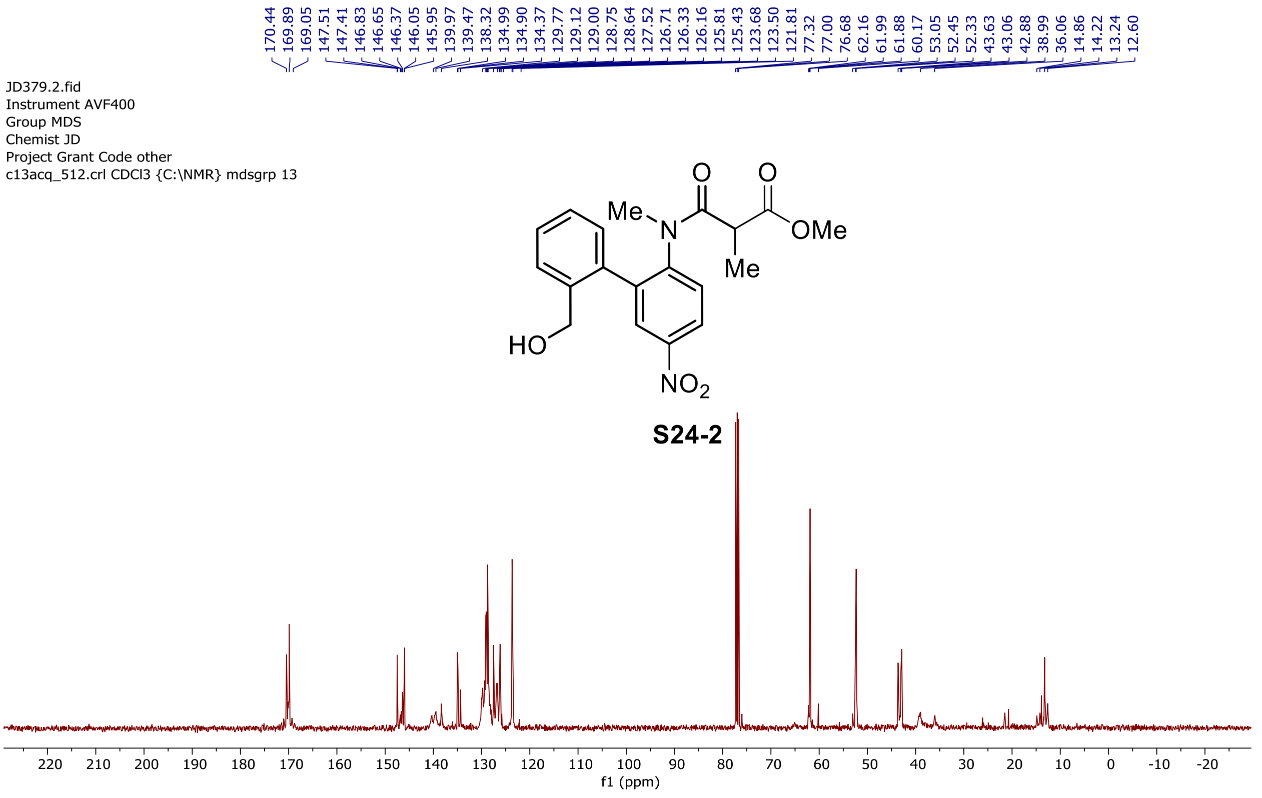
170.44 169.89 169.05 147.51 147.41 146.83 146.65 146.37 146.05 145.95 139.97 139.47 138.32 134.99 134.90 134.37 129.77 129.12 129.00 128.75 128.64 127.52 126.71 126.33 126.16 125.81 125.43 123.68 123.50 121.81 77.32 77.00 76.68 62.16 61.99 61.88 60.17 53.05 52.45 52.33 43.63 43.06 42.88 38.99 36.06 14.86 14.22 13.24 12.60
JD379.2.fid
Instrument AVF400
Group MDS
Chemist JD
Project Grant Code other
c13acq_512.crl CDCl3 {C:\NMR} mdsgrp 13
O
O
Me
N
OMe
Me
HO
NO2
S24-2
220 210 200 190 180 170 160 150 140 130 120 110 100 90 80 70 60 50 40 30 20 10 0 -10 -20
f1 (ppm)

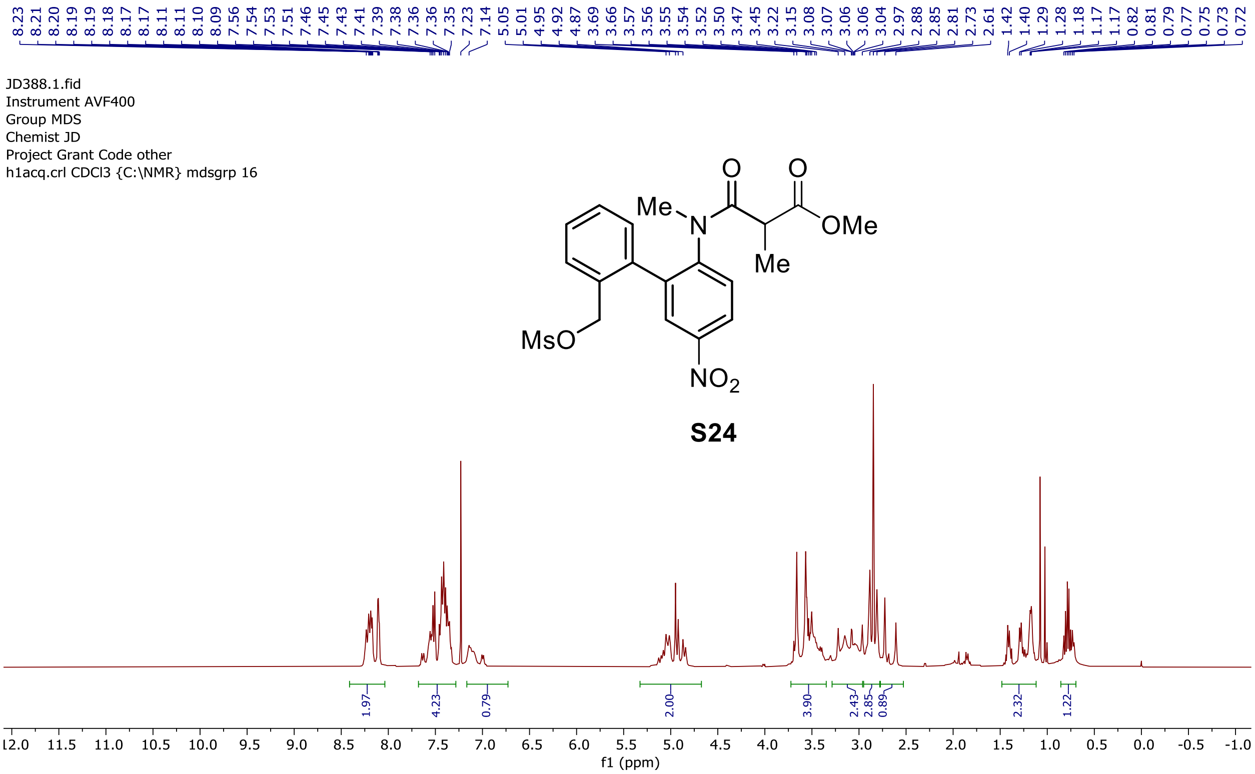
8.23 8.21 8.20 8.19 8.19 8.18 8.17 8.17 8.11 8.11 8.10 8.09 7.56 7.54 7.53 7.51 7.46 7.45 7.43 7.41 7.39 7.38 7.36 7.35 7.23 7.14 5.05 5.01 4.95 4.92 4.87 3.69 3.66 3.57 3.56 3.55 3.54 3.52 3.50 3.47 3.45 3.22 3.15 3.08 3.07 3.06 3.06 3.04 2.97 2.88 2.85 2.81 2.73 2.61 1.42 1.40 1.29 1.28 1.18 1.17 1.17 0.82 0.81 0.79 0.77 0.75 0.73 0.72
JD388.1.fid
Instrument AVF400
Group MDS
Chemist JD
Project Grant Code other
h1acq.crl CDCl3 {C:\NMR} mdsgrp 16
O
O
Me
N
OMe
Me
MsO
NO2
S24
1.97
4.23
0.79
2.00
3.90
2.43
2.85
0.89
2.32
1.22
12.0 11.5 11.0 10.5 10.0 9.5 9.0 8.5 8.0 7.5 7.0 6.5 6.0 5.5 5.0 4.5 4.0 3.5 3.0 2.5 2.0 1.5 1.0 0.5 0.0 -0.5 -1.0
f1 (ppm)

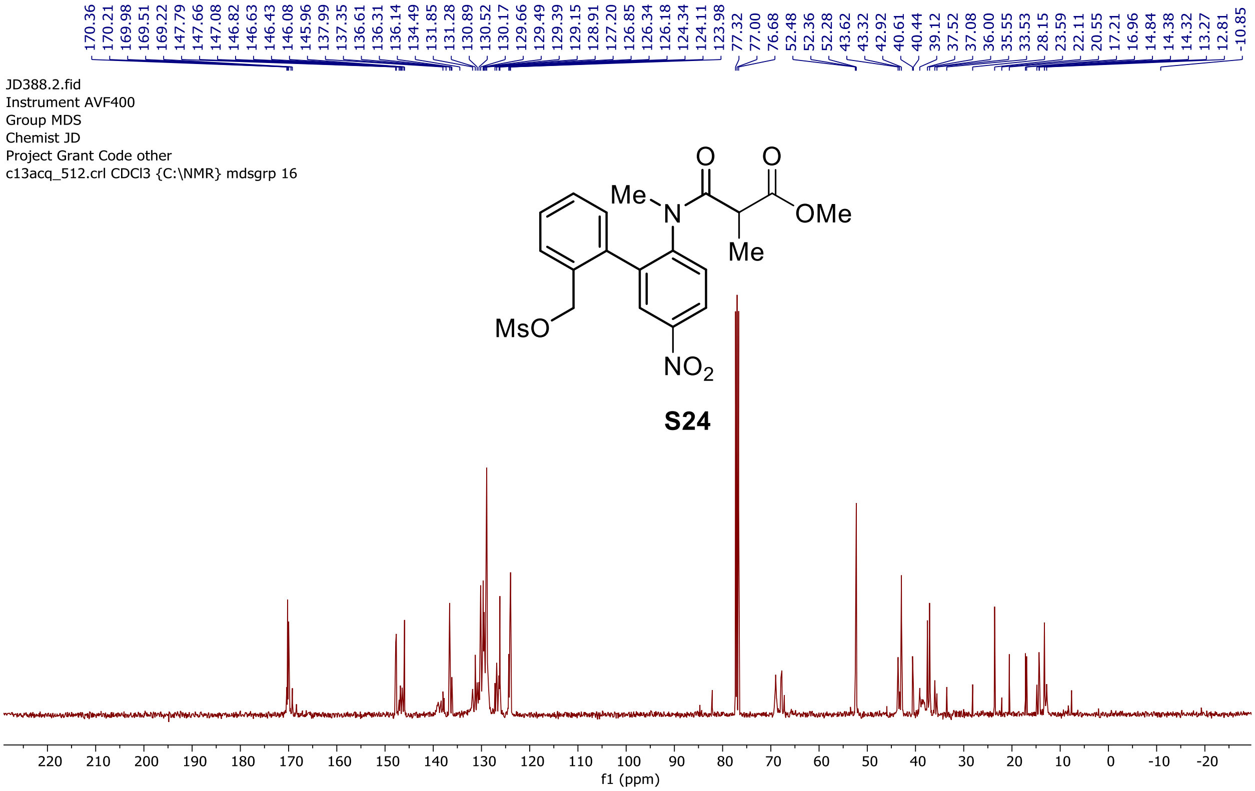
170.36 170.21 169.98 169.51 169.22 147.79 147.66 147.08 146.82 146.63 146.43 146.08 145.96 137.95 137.35 136.61 136.31 136.14 134.49 131.85 131.28 130.89 130.52 130.17 129.66 129.49 129.39 129.15 128.91 127.20 126.85 126.34 126.18 124.34 124.11 123.98 77.32 77.00 76.68 52.48 52.36 52.28 43.62 43.32 42.92 40.61 40.44 39.12 37.52 37.08 36.00 35.55 33.53 28.15 23.59 22.11 20.55 17.21 16.96 14.84 14.38 14.32 13.27 12.81 -10.85
JD388.2.fid
Instrument AVF400
Group MDS
Chemist JD
Project Grant Code other
c13acq_512.crl CDCl3 {C:\NMR} mdsgrp 16
O
O
Me
N
OMe
Me
MsO
NO2
S24
220 210 200 190 180 170 160 150 140 130 120 110 100 90 80 70 60 50 40 30 20 10 0 -10 -20
f1 (ppm)

JD341-S.1.fid
Instrument AVF400
Group MDS
Chemist JD
Project Grant Code other
h1acq.crl CDCl3 {C:\NMR} mdsgrp 14

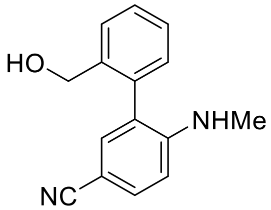

S25-1

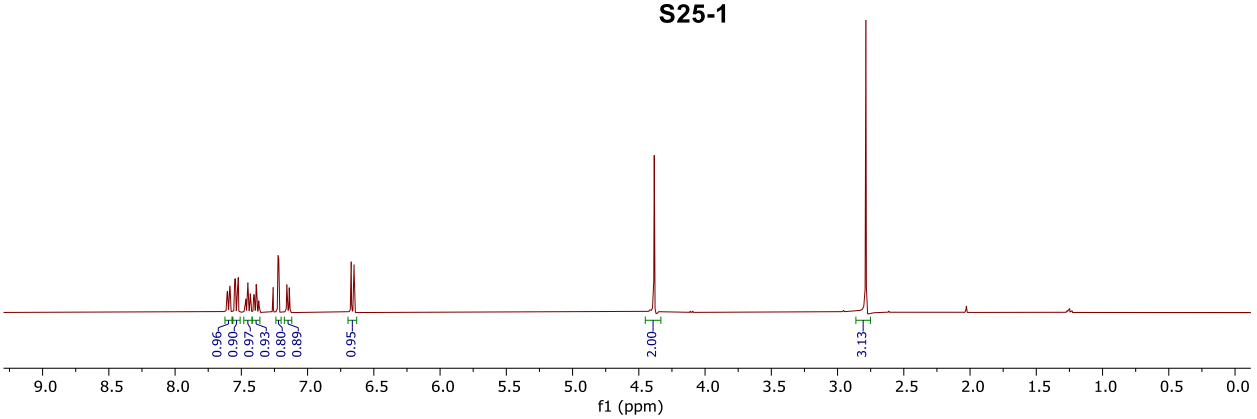

JD341-S.2.fid
Instrument AVF400
Group MDS
Chemist JD
Project Grant Code other
c13acq_512.crl CDCl3 {C:\NMR} mdsgrp 14

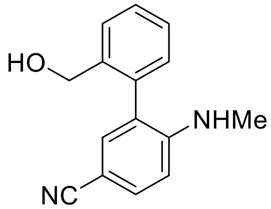

S25-1

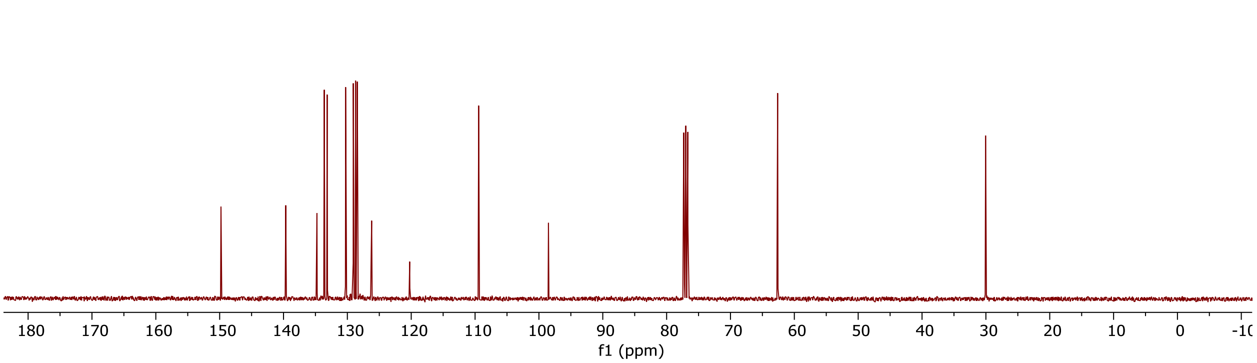

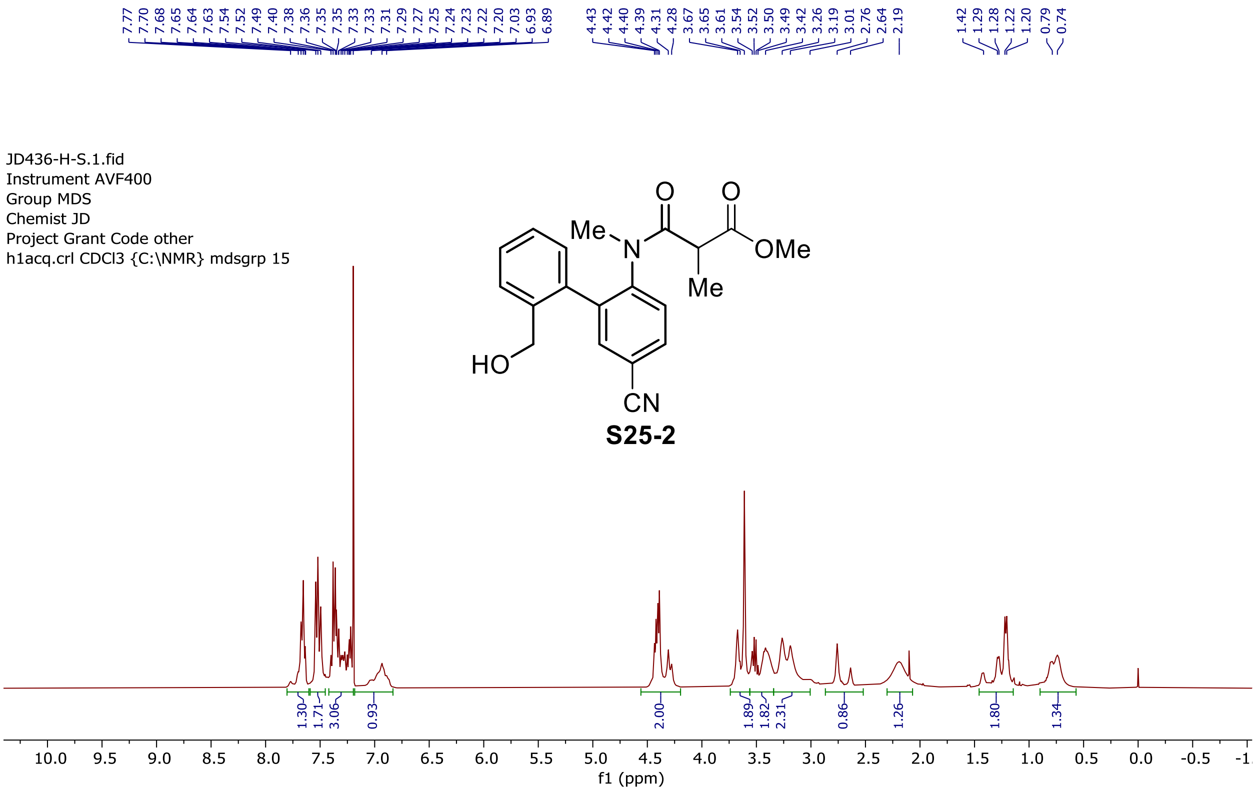
7.77 7.70 7.68 7.65 7.64 7.63 7.54 7.52 7.49 7.40 7.38 7.36 7.35 7.35 7.33 7.33 7.31 7.29 7.27 7.25 7.24 7.23 7.22 7.20 7.03 6.93 6.89
4.43 4.42 4.40 4.39 4.31 4.28 3.67 3.65 3.61 3.54 3.52 3.50 3.49 3.42 3.26 3.19 3.01 2.76 2.64 2.19
1.42 1.29 1.28 1.22 1.20 0.79 0.74
JD436-H-S.1.fid
Instrument AVF400
Group MDS
Chemist JD
Project Grant Code other
h1acq.crl CDCl3 {C:\NMR} mdsgrp 15
Me
N
O
O
OMe
Me
HO
CN
S25-2
1.30 1.71 3.06 0.93 2.00 1.89 1.82 2.31 0.86 1.26 1.80 1.34
10.0 9.5 9.0 8.5 8.0 7.5 7.0 6.5 6.0 5.5 5.0 4.5 4.0 3.5 3.0 2.5 2.0 1.5 1.0 0.5 0.0 -0.5
f1 (ppm)

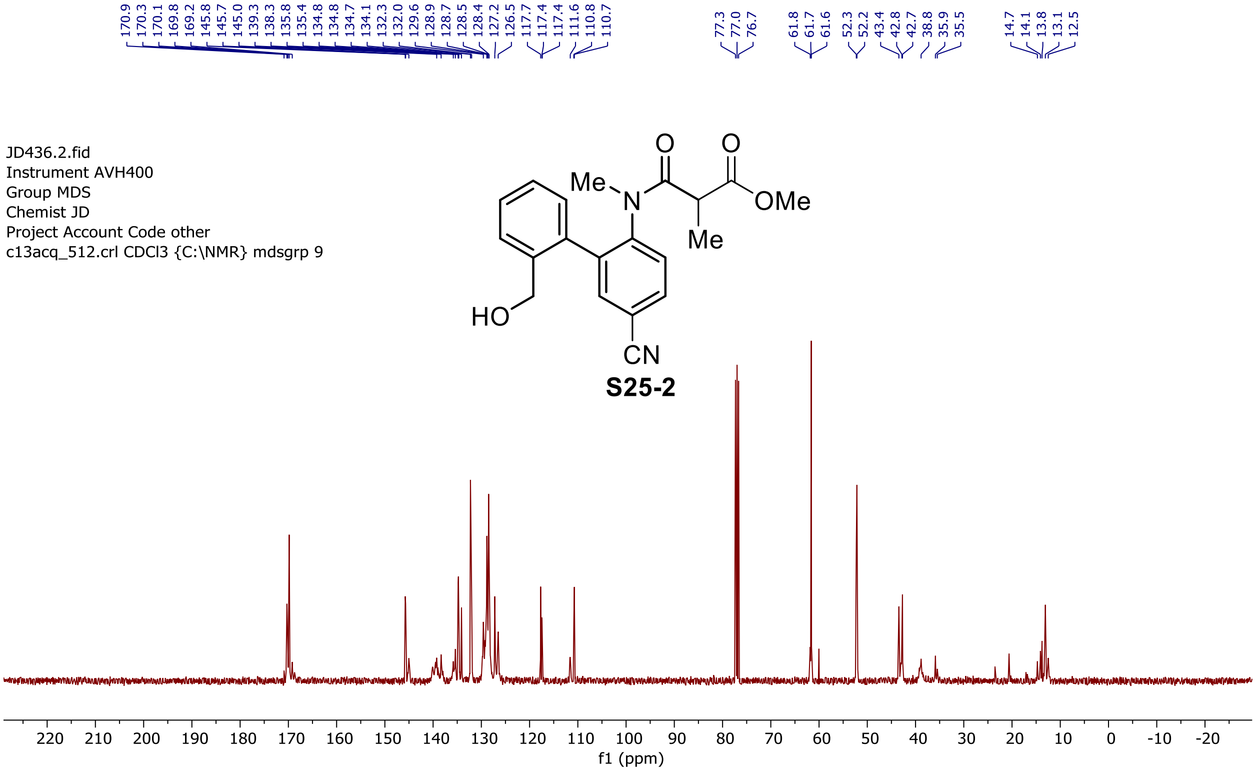
170.9 170.3 170.1 169.8 169.2 145.8 145.7 145.0 139.3 138.3 135.8 135.4 134.8 134.7 134.1 132.3 132.0 129.6 128.9 128.7 128.5 128.4 127.2 126.5 117.7 117.4 117.4 111.6 110.8 110.7
77.3 77.0 76.7
61.8 61.7 61.6 52.3 52.2 43.4 42.8 42.7 38.8 35.9 35.5
14.7 14.1 13.8 13.1 12.5
JD436.2.fid
Instrument AVH400
Group MDS
Chemist JD
Project Account Code other
c13acq_512.crl CDCl3 {C:\NMR} mdsgrp 9
Me
N
O
O
OMe
Me
HO
CN
S25-2
220 210 200 190 180 170 160 150 140 130 120 110 100 90 80 70 60 50 40 30 20 10 0 -10 -20
f1 (ppm)

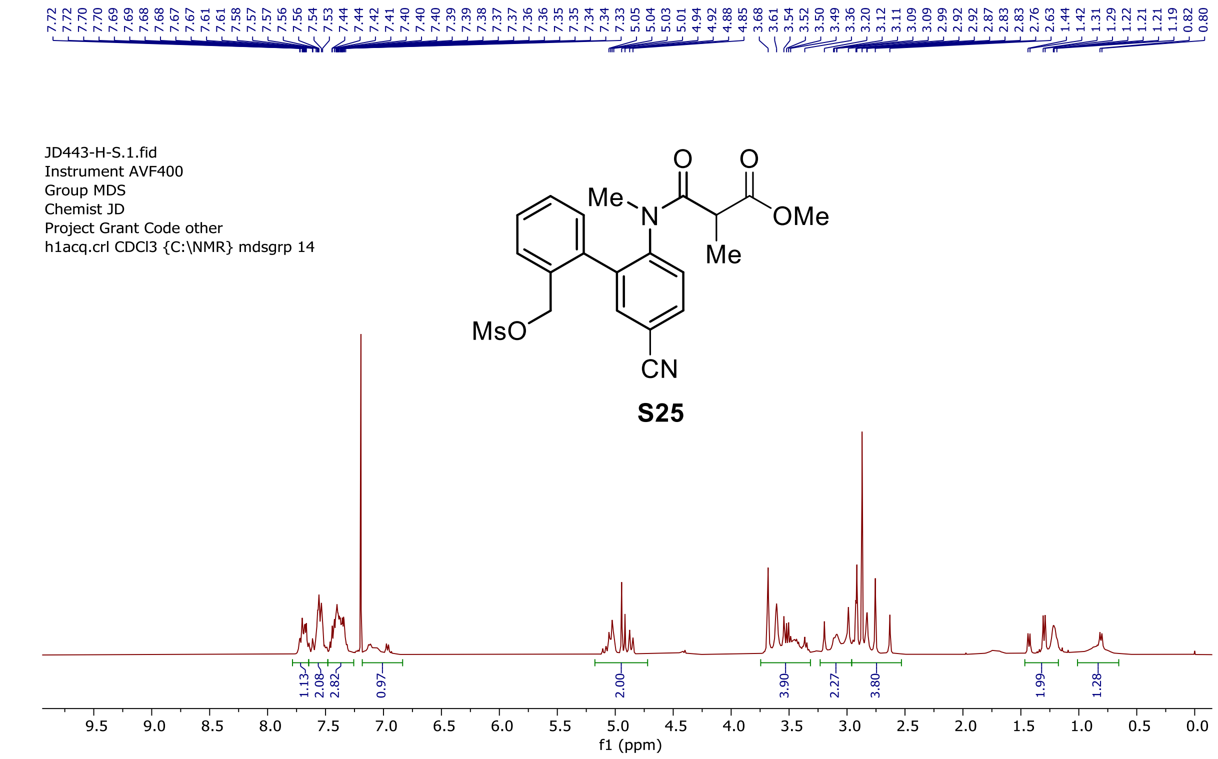
7.72 7.72 7.70 7.69 7.69 7.68 7.68 7.67 7.67 7.61 7.61 7.58 7.57 7.57 7.56 7.54 7.53 7.44 7.42 7.41 7.40 7.40 7.39 7.39 7.38 7.37 7.37 7.36 7.36 7.35 7.35 7.34 7.34 7.33 5.05 5.04 5.03 5.01 4.94 4.92 4.85 3.68 3.61 3.54 3.52 3.50 3.49 3.36 3.20 3.12 3.11 3.09 3.09 2.99 2.92 2.92 2.87 2.83 2.83 2.76 2.63 1.44 1.42 1.31 1.29 1.22 1.21 1.21 1.19 0.82 0.80
JD443-H-S.1.fid
Instrument AVF400
Group MDS
Chemist JD
Project Grant Code other
h1acq.crl CDCl3 {C:\NMR} mdsgrp 14
Me
N
O
O
OMe
Me
MsO
CN
S25
1.13 2.08 2.82 0.97 2.00 3.90 2.27 3.80 1.99 1.28
9.5 9.0 8.5 8.0 7.5 7.0 6.5 6.0 5.5 5.0 4.5 4.0 3.5 3.0 2.5 2.0 1.5 1.0 0.5 0.0
f1 (ppm)

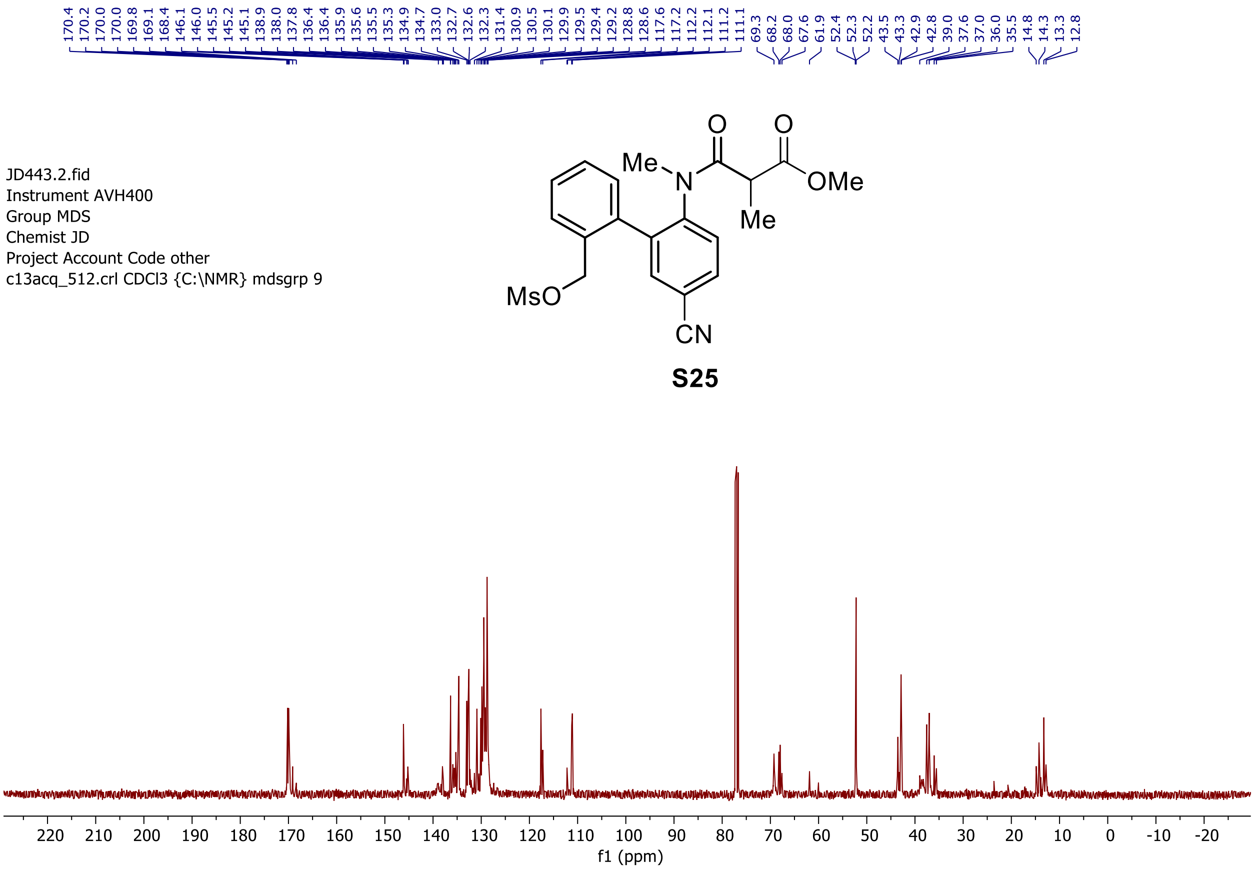
170.4 170.2 170.0 170.0 169.8 169.1 168.4 146.1 146.0 145.5 145.2 145.1 138.9 138.0 137.8 136.4 136.4 135.9 135.6 135.5 135.3 134.9 134.7 133.0 132.7 132.6 132.3 131.4 130.9 130.5 130.1 129.9 129.5 129.4 129.2 128.8 128.6 117.6 117.2 112.2 112.1 111.2 111.1 69.3 68.2 68.0 67.6 61.9 52.4 52.3 52.2 43.5 43.3 42.9 42.8 39.0 37.6 37.0 36.0 35.5 14.8 14.3 13.3 12.8
JD443.2.fid
Instrument AVH400
Group MDS
Chemist JD
Project Account Code other
c13acq_512.crl CDCl3 {C:\NMR} mdsgrp 9
Me
N
O
O
OMe
Me
MsO
CN
S25
220 210 200 190 180 170 160 150 140 130 120 110 100 90 80 70 60 50 40 30 20 10 0 -10 -20
f1 (ppm)

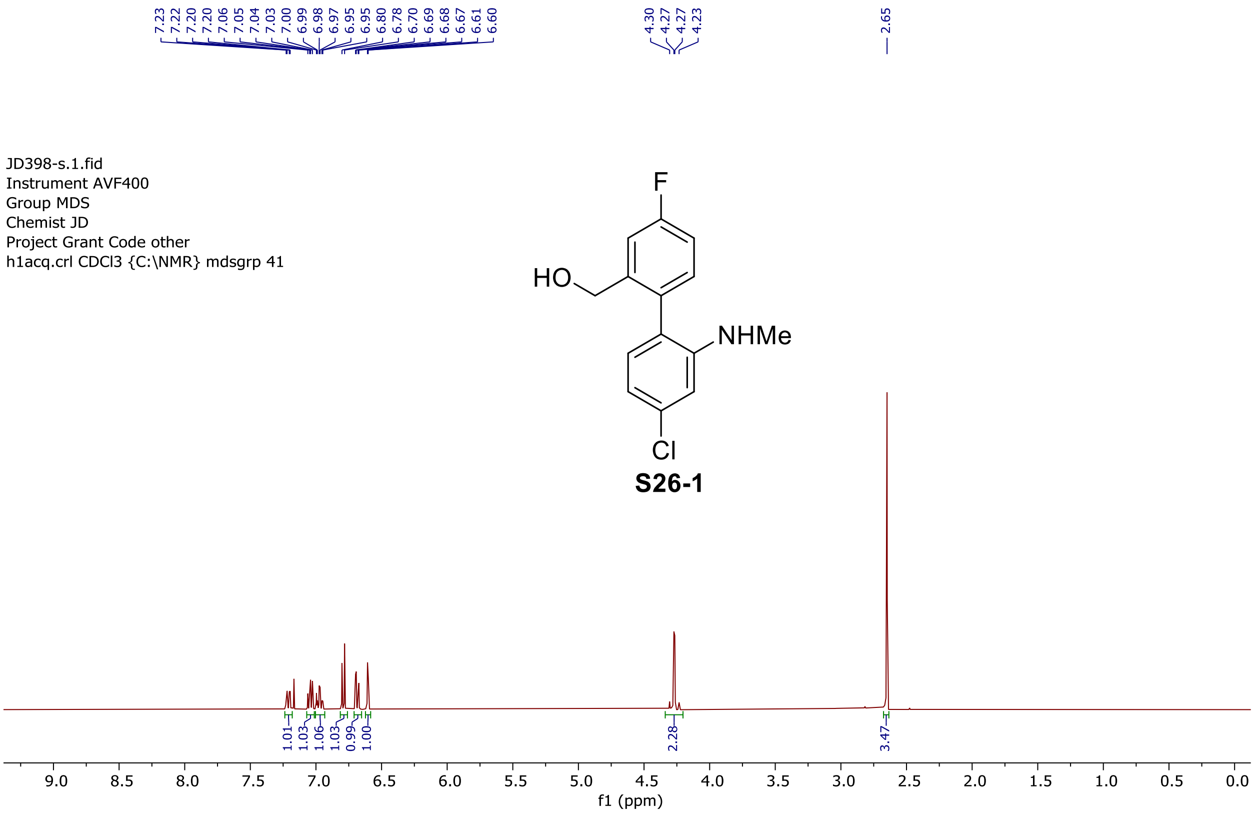
7.23 7.22 7.20 7.20 7.06 7.05 7.04 7.03 7.00 6.99 6.98 6.97 6.95 6.95 6.80 6.78 6.70 6.69 6.68 6.67 6.61 6.60
4.30 4.27 4.27 4.23
2.65
JD398-s.1.fid
Instrument AVF400
Group MDS
Chemist JD
Project Grant Code other
h1acq.crl CDCl3 {C:\NMR} mdsgrp 41
F
HO
NHMe
Cl
S26-1
1.01 1.03 1.06 1.03 0.99 1.00
2.28
3.47
9.0 8.5 8.0 7.5 7.0 6.5 6.0 5.5 5.0 4.5 4.0 3.5 3.0 2.5 2.0 1.5 1.0 0.5 0.0
f1 (ppm)

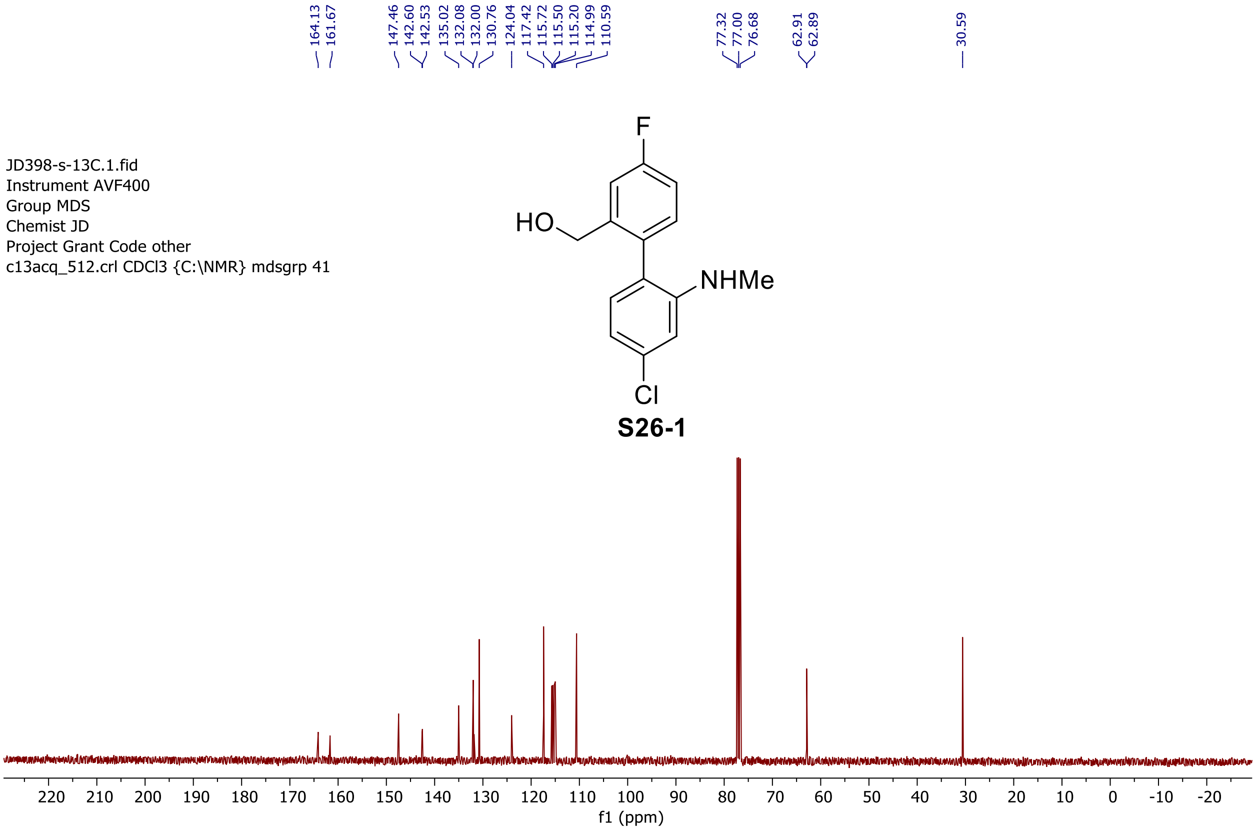
164.13 161.67
147.46 142.60 142.53 135.02 132.08 132.00 130.76 124.04 117.42 115.72 115.50 115.20 114.99 110.59
77.32 77.00 76.68
62.91 62.89
30.59
JD398-s-13C.1.fid
Instrument AVF400
Group MDS
Chemist JD
Project Grant Code other
c13acq_512.crl CDCl3 {C:\NMR} mdsgrp 41
F
HO
NHMe
Cl
S26-1
220 210 200 190 180 170 160 150 140 130 120 110 100 90 80 70 60 50 40 30 20 10 0 -10 -20
f1 (ppm)

JD398-F.1.fid
Instrument AVF400
Group MDS
Chemist JD
Project Grant Code other
f19dec.crl CDCl3 {C:\NMR} mdsgrp 26

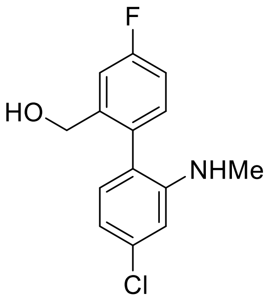

S26-1

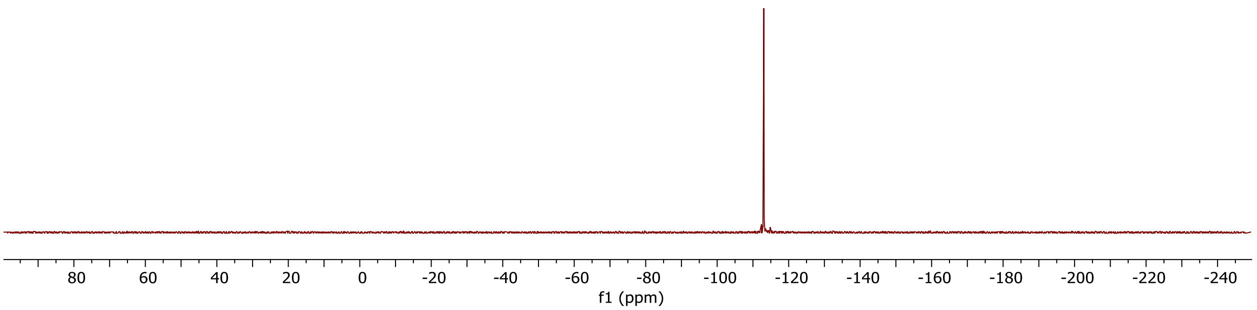

jd600502511.1.fid
Instrument AVC500
Group MDS
Project Account Code DMR01090
Jiyuan Du 60050 25/11/20

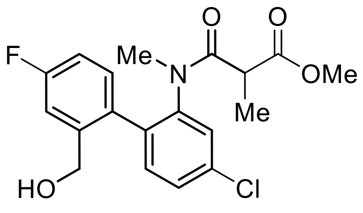

S26-2

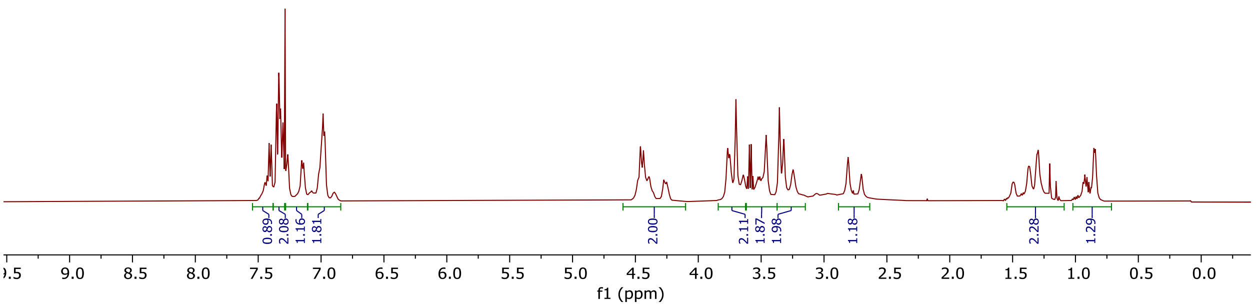

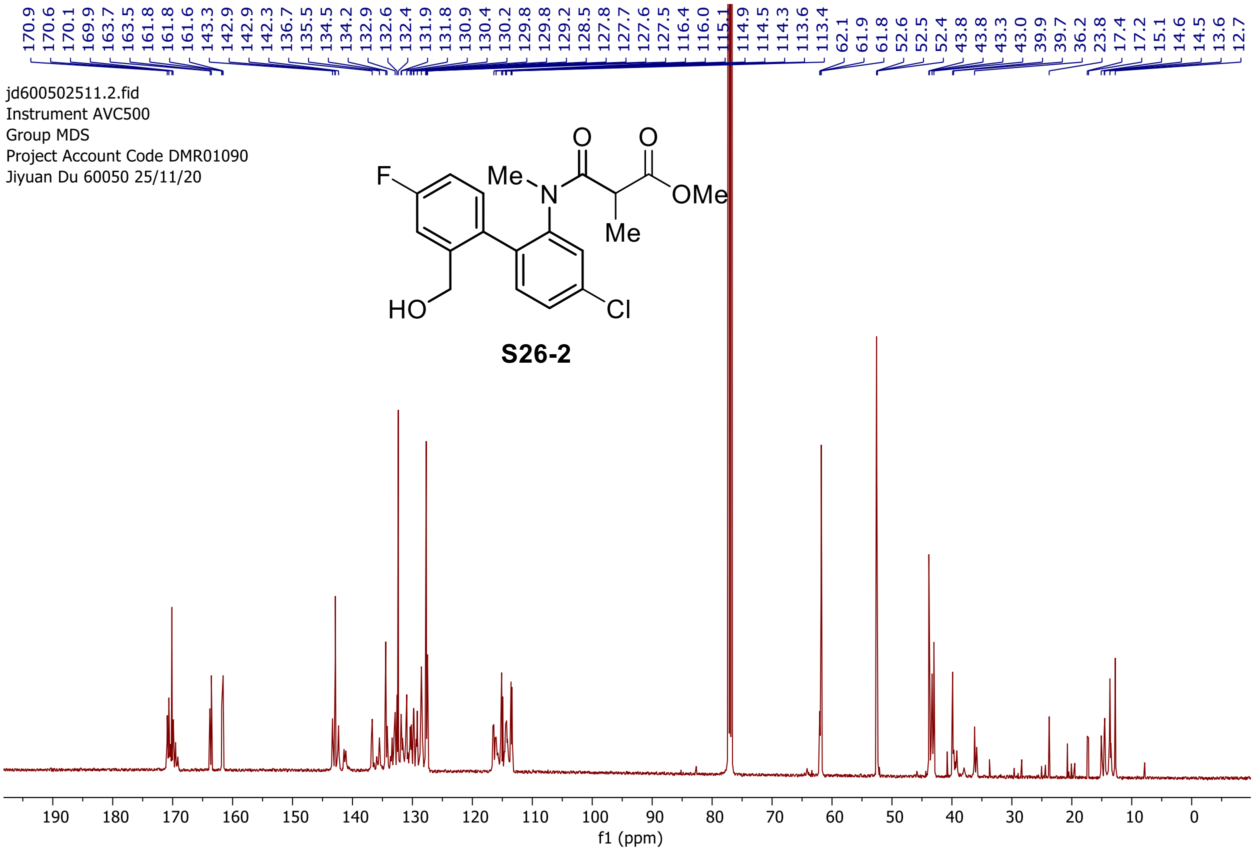
170.9 170.6 170.1 169.9 163.7 163.5 161.8 161.8 161.6 143.3 142.9 142.9 142.3 136.7 135.5 134.5 134.2 132.9 132.6 132.4 131.9 131.8 130.9 130.4 130.2 129.8 129.8 129.2 128.5 127.8 127.7 127.6 127.5 116.4 116.0 115.1 114.9 114.5 114.3 113.6 113.4 62.1 61.9 61.8 52.6 52.5 52.4 43.8 43.8 43.3 43.0 39.9 39.7 36.2 23.8 17.4 17.2 15.1 14.6 14.5 13.6 12.7
jd600502511.2.fid
Instrument AVC500
Group MDS
Project Account Code DMR01090
Jiyuan Du 60050 25/11/20
F
Me
N
O
O
OMe
Me
HO
Cl
S26-2
190 180 170 160 150 140 130 120 110 100 90 80 70 60 50 40 30 20 10 0
f1 (ppm)

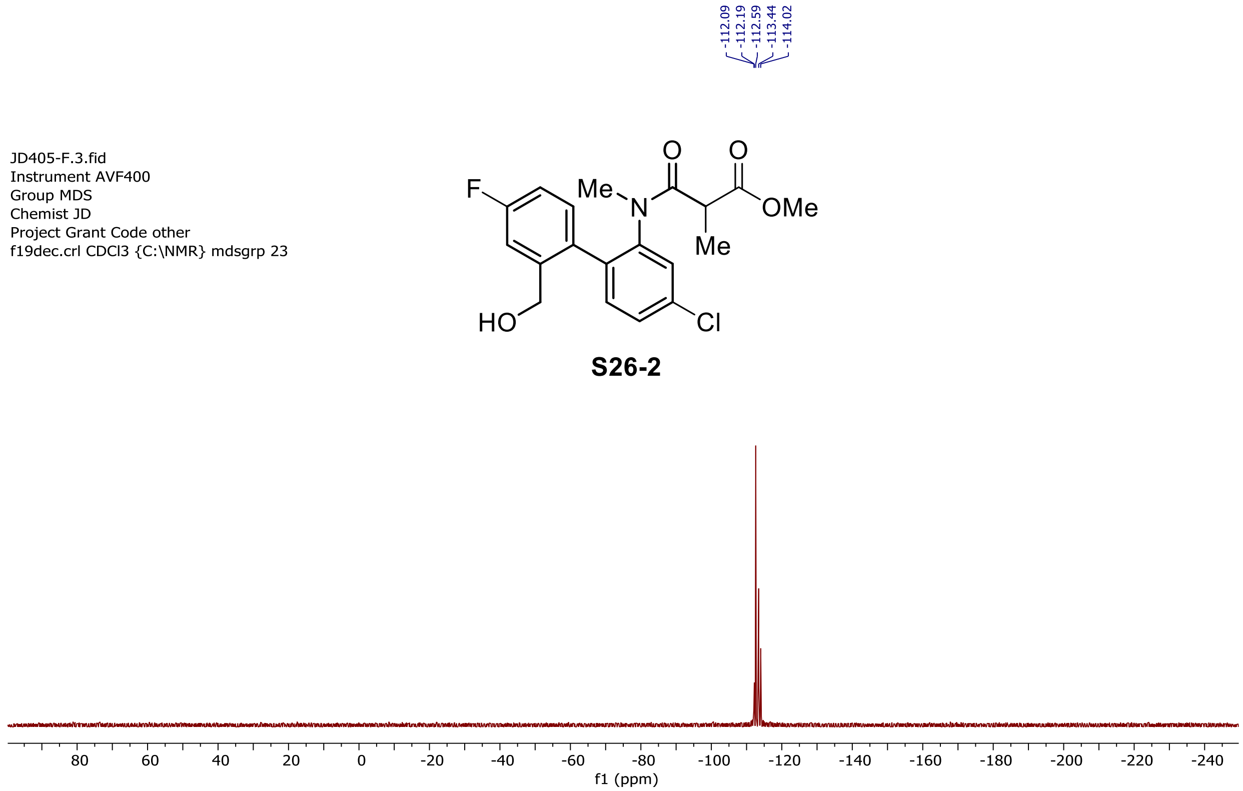
-112.09
-112.19
-112.59
-113.44
-114.02
JD405-F.3.fid
Instrument AVF400
Group MDS
Chemist JD
Project Grant Code other
f19dec.crl CDCl3 {C:\NMR} mdsgrp 23
F
Me
N
O
O
OMe
Me
HO
Cl
S26-2
80 60 40 20 0 -20 -40 -60 -80 -100 -120 -140 -160 -180 -200 -220 -240
f1 (ppm)

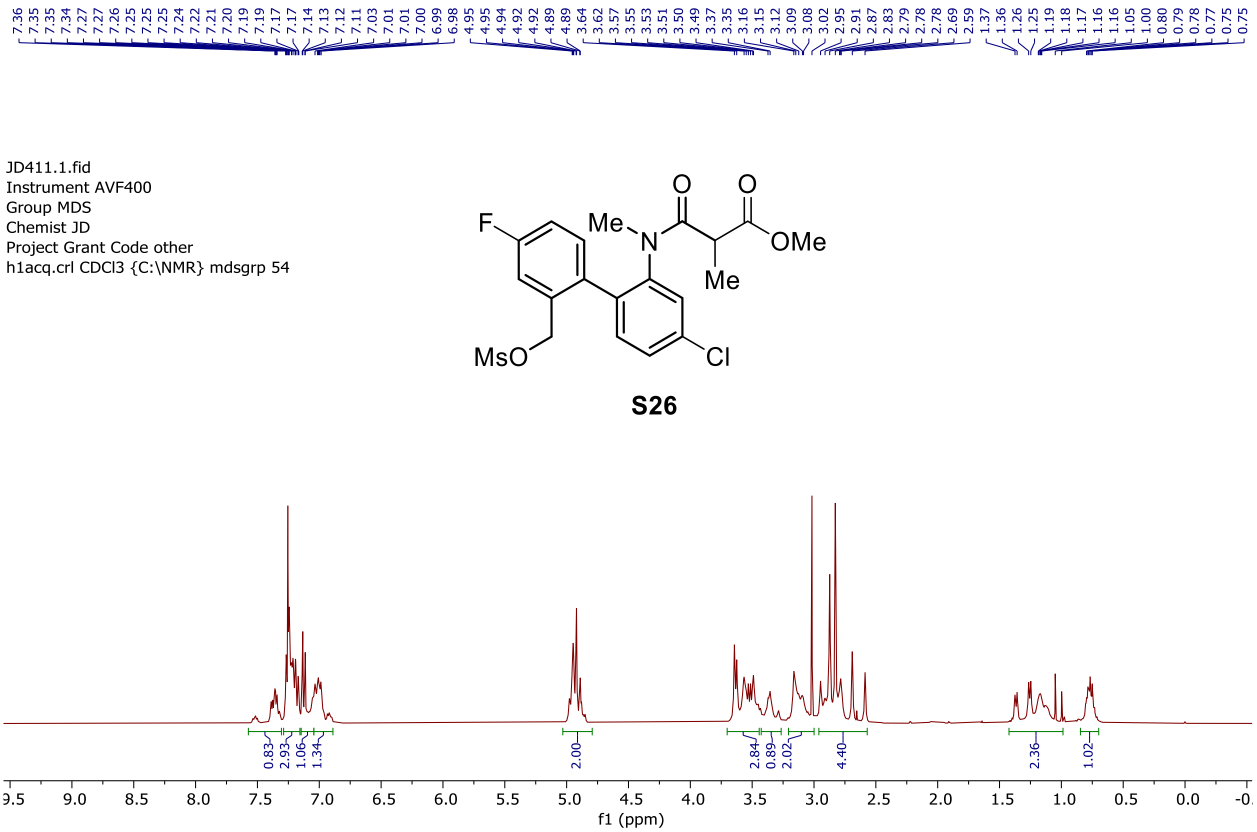
7.36 7.35 7.34 7.27 7.27 7.26 7.25 7.25 7.24 7.22 7.21 7.20 7.19 7.19 7.17 7.17 7.14 7.13 7.12 7.11 7.03 7.01 7.01 7.00 6.99 6.98 4.95 4.95 4.94 4.92 4.92 4.89 4.89 3.64 3.62 3.57 3.55 3.53 3.51 3.50 3.49 3.37 3.35 3.16 3.15 3.12 3.09 3.08 3.02 2.95 2.91 2.87 2.83 2.79 2.78 2.78 2.69 2.59 1.37 1.36 1.26 1.25 1.19 1.18 1.17 1.16 1.16 1.05 1.00 0.80 0.79 0.78 0.77 0.75 0.75
JD411.1.fid
Instrument AVF400
Group MDS
Chemist JD
Project Grant Code other
h1acq.crl CDCl3 {C:\NMR} mdsgrp 54
F
Me
N
O
O
OMe
Me
MsO
Cl
S26
0.83 2.93 1.06 1.34 2.00 2.84 0.89 2.02 4.40 2.36 1.02
f1 (ppm)

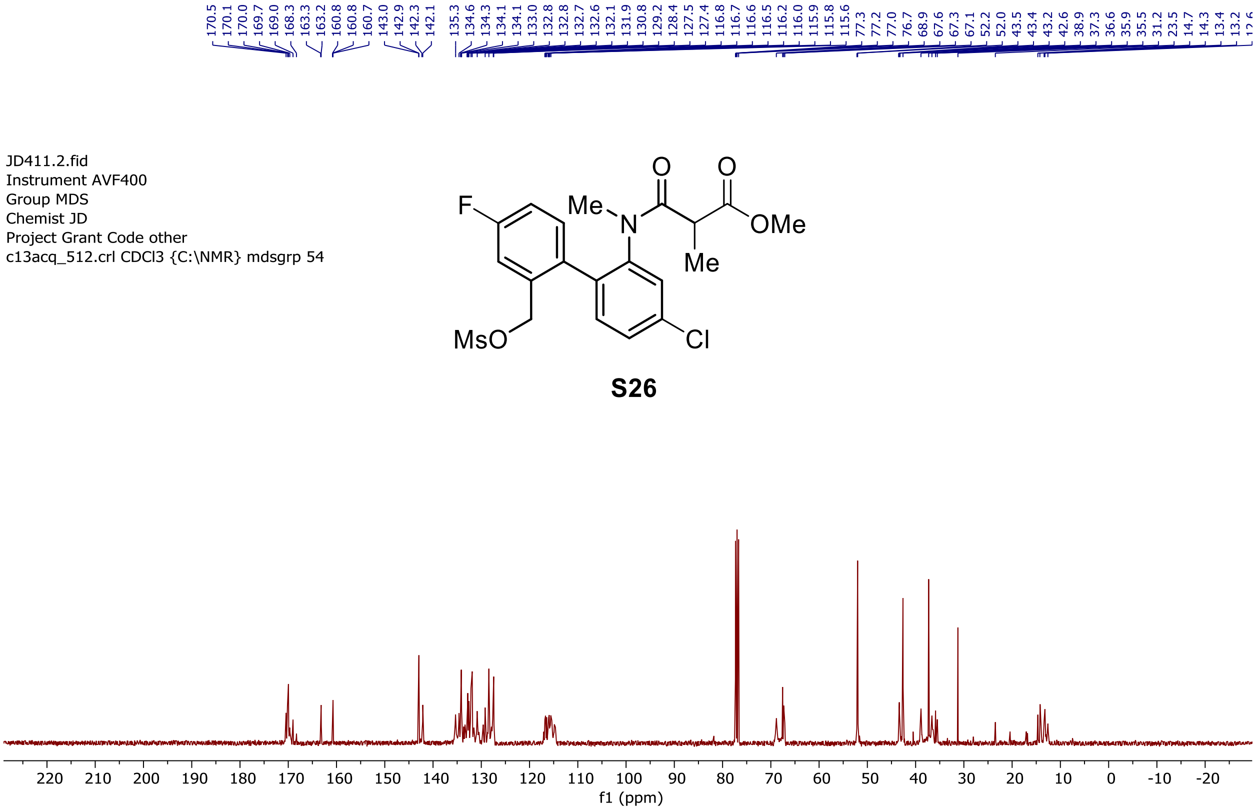
170.5 170.1 170.0 169.7 169.0 168.3 163.3 163.2 160.8 160.8 160.7 143.0 142.9 142.3 142.1 135.3 134.6 134.3 134.1 134.1 133.0 132.8 132.8 132.7 132.6 132.1 131.9 130.8 129.2 128.4 127.5 127.4 116.8 116.7 116.6 116.5 116.2 116.0 115.9 115.8 115.6 77.3 77.2 77.0 76.7 68.9 67.6 67.3 67.1 52.2 52.0 43.5 43.4 43.2 42.6 38.9 37.3 36.6 35.9 35.5 31.2 23.5 14.7 14.3 13.4 13.2
JD411.2.fid
Instrument AVF400
Group MDS
Chemist JD
Project Grant Code other
c13acq_512.crl CDCl3 {C:\NMR} mdsgrp 54
F
Me
N
O
O
OMe
Me
MsO
Cl
S26
f1 (ppm)

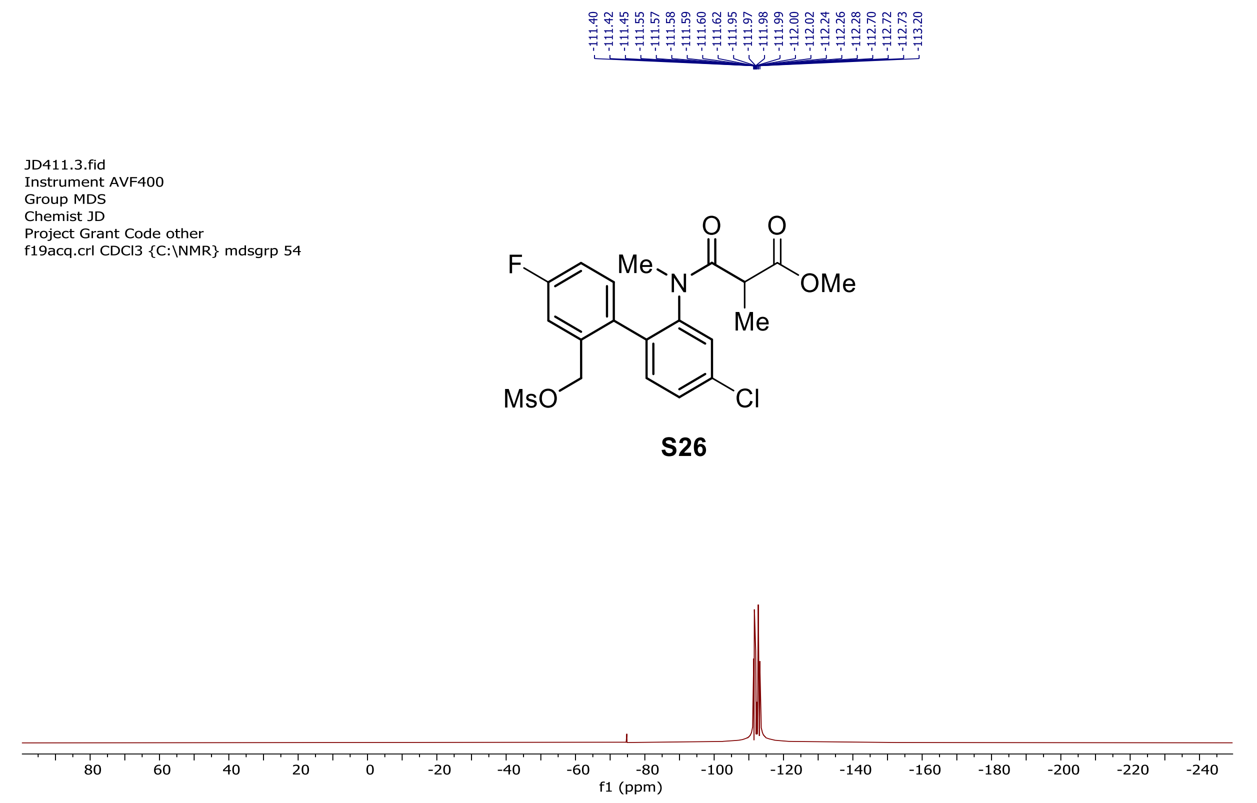
JD411.3.fid
Instrument AVF400
Group MDS
Chemist JD
Project Grant Code other
f19acq.crl CDCl3 {C:\NMR} mdsgrp 54
-111.40
-111.42
-111.45
-111.55
-111.57
-111.58
-111.59
-111.60
-111.62
-111.95
-111.97
-111.98
-111.99
-112.00
-112.02
-112.24
-112.26
-112.28
-112.70
-112.72
-112.73
-113.20
f1 (ppm)

S26

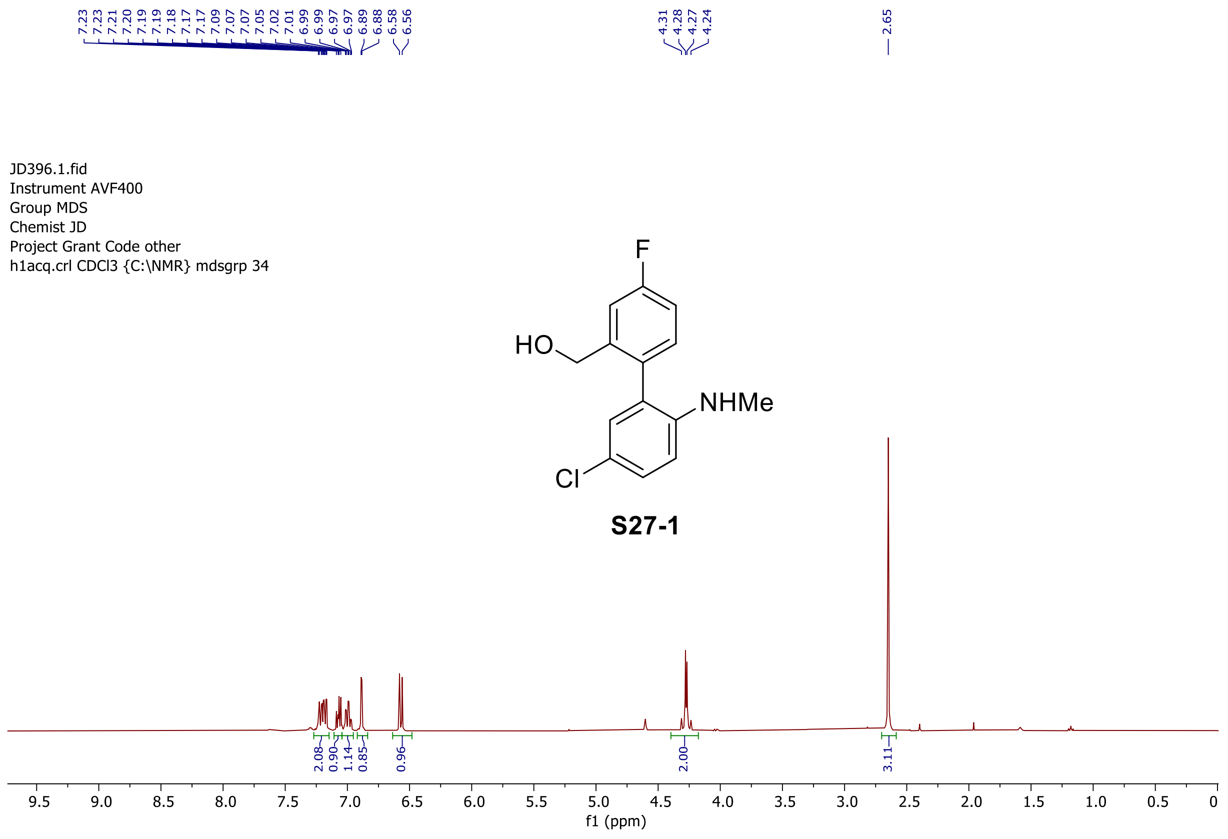
JD396.1.fid
Instrument AVF400
Group MDS
Chemist JD
Project Grant Code other
h1acq.crl CDCl3 {C:\NMR} mdsgrp 34
7.23
7.23
7.21
7.20
7.19
7.19
7.18
7.17
7.17
7.09
7.07
7.07
7.05
7.02
7.01
6.99
6.99
6.97
6.97
6.89
6.88
6.58
6.56
4.31
4.28
4.27
4.24
2.65
2.08
0.90
1.14
0.85
0.96
2.00
3.11
f1 (ppm)

S27-1

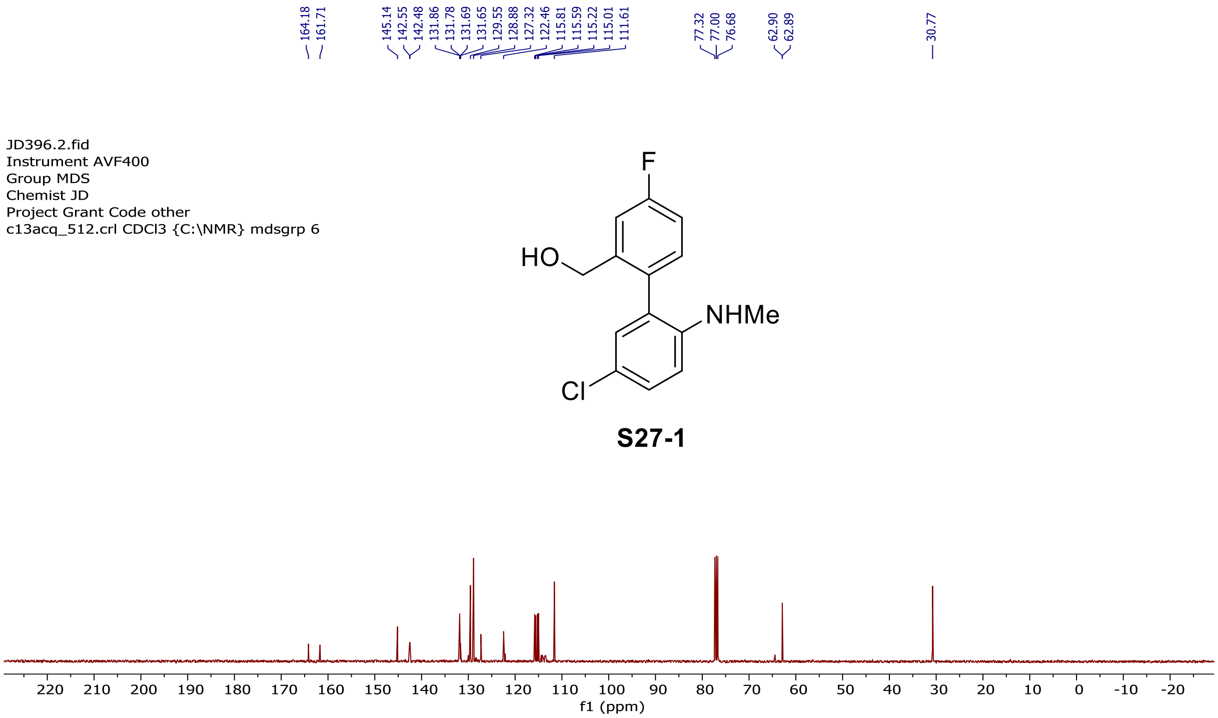
164.18
161.71
145.14
142.55
142.48
131.86
131.78
131.69
131.65
129.55
128.88
127.32
122.46
115.81
115.59
115.22
115.01
111.61
77.32
77.00
76.68
62.90
62.89
30.77
JD396.2.fid
Instrument AVF400
Group MDS
Chemist JD
Project Grant Code other
c13acq_512.crl CDCl3 {C:\NMR} mdsgrp 6
F
HO
NHMe
Cl
S27-1
220 210 200 190 180 170 160 150 140 130 120 110 100 90 80 70 60 50 40 30 20 10 0 -10 -20
f1 (ppm)

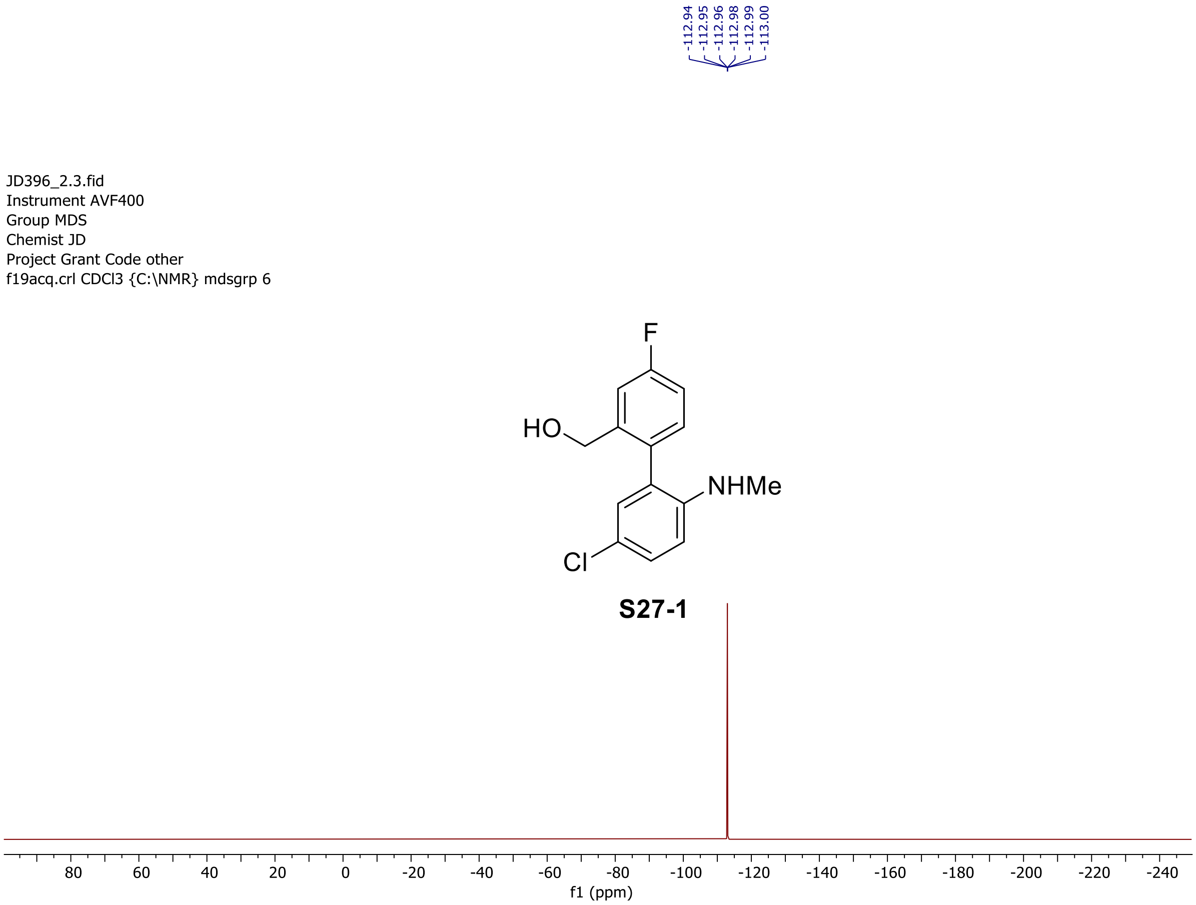
-112.94
-112.95
-112.96
-112.98
-112.99
-113.00
JD396_2.3.fid
Instrument AVF400
Group MDS
Chemist JD
Project Grant Code other
f19acq.crl CDCl3 {C:\NMR} mdsgrp 6
F
HO
NHMe
Cl
S27-1
80 60 40 20 0 -20 -40 -60 -80 -100 -120 -140 -160 -180 -200 -220 -240
f1 (ppm)

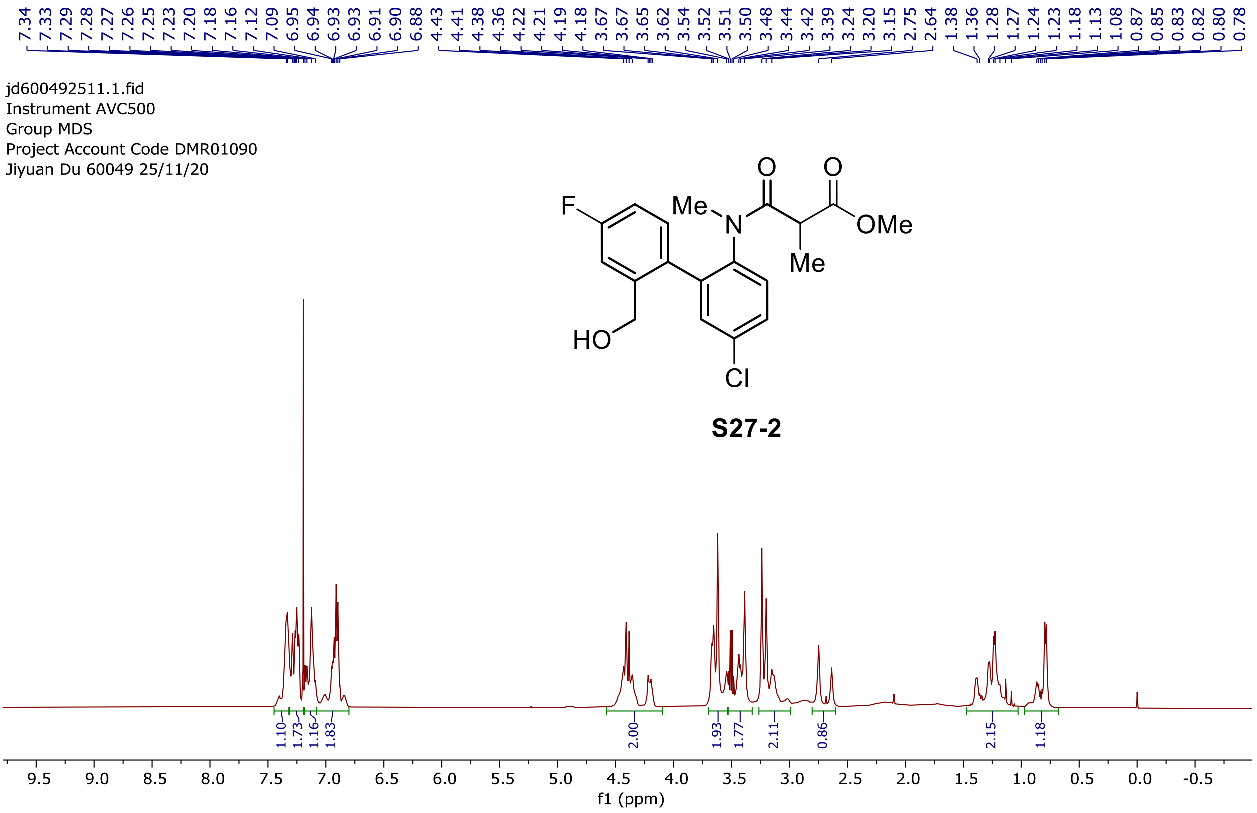
7.34 7.33 7.29 7.28 7.27 7.26 7.25 7.23 7.20 7.18 7.16 7.12 7.09 6.95 6.94 6.93 6.93 6.91 6.90 6.88 4.43 4.41 4.38 4.36 4.22 4.21 4.19 4.18 3.67 3.67 3.65 3.62 3.54 3.52 3.51 3.50 3.48 3.44 3.42 3.39 3.24 3.20 3.15 2.75 2.64 1.38 1.36 1.28 1.27 1.24 1.23 1.18 1.13 1.08 0.87 0.85 0.83 0.82 0.80 0.78
jd600492511.1.fid
Instrument AVC500
Group MDS
Project Account Code DMR01090
Jiyuan Du 60049 25/11/20
F
Me
N
O
O
OMe
Me
HO
Cl
S27-2
1.10 1.73 1.16 1.83 2.00 1.93 1.77 2.11 0.86 2.15 1.18
9.5 9.0 8.5 8.0 7.5 7.0 6.5 6.0 5.5 5.0 4.5 4.0 3.5 3.0 2.5 2.0 1.5 1.0 0.5 0.0 -0.5
f1 (ppm)

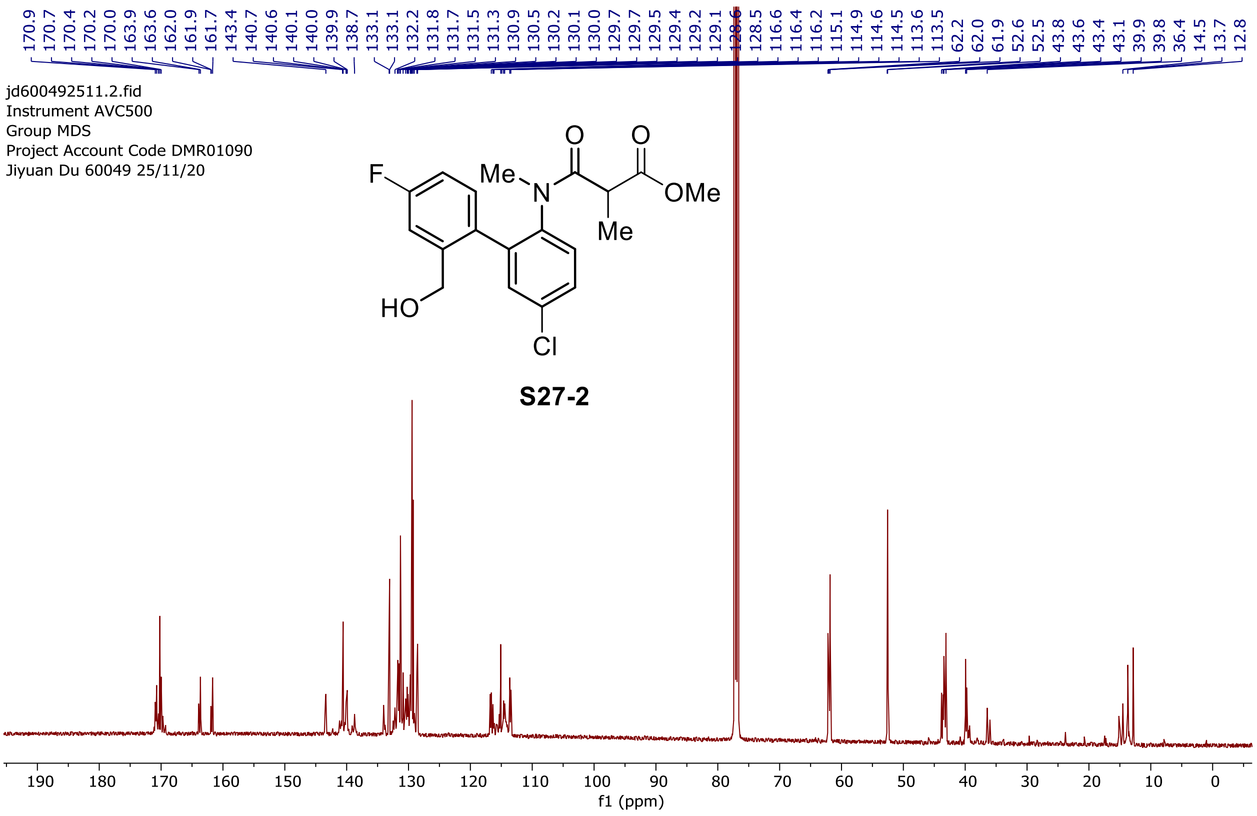
170.9 170.7 170.4 170.2 170.0 163.9 163.6 162.0 161.9 161.7 143.4 140.7 140.6 140.1 140.0 139.9 138.7 133.1 132.2 131.8 131.7 131.5 131.3 130.9 130.5 130.2 130.1 130.0 129.7 129.7 129.5 129.4 129.2 129.1 128.6 128.5 116.6 116.4 116.2 115.1 114.9 114.6 114.5 113.6 113.5 62.2 62.0 61.9 52.6 52.5 43.8 43.6 43.4 43.1 39.9 39.8 36.4 14.5 13.7 12.8
jd600492511.2.fid
Instrument AVC500
Group MDS
Project Account Code DMR01090
Jiyuan Du 60049 25/11/20
F
Me
N
O
O
OMe
Me
HO
Cl
S27-2
190 180 170 160 150 140 130 120 110 100 90 80 70 60 50 40 30 20 10 0
f1 (ppm)

JD404-F.3.fid
Instrument AVF400
Group MDS
Chemist JD
Project Grant Code other
f19dec.crl CDCl3 {C:\NMR} mdsgrp 22

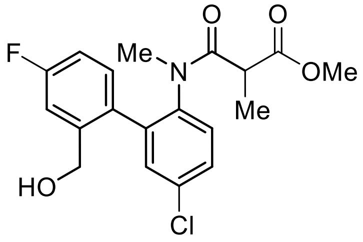

S27-2

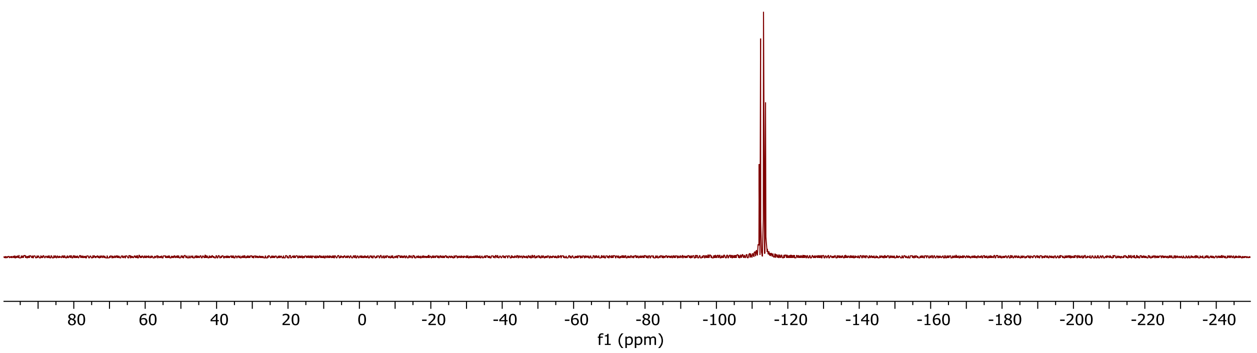

JD410-H-s.1.fid
Instrument AVF400
Group MDS
Chemist JD
Project Grant Code other
h1acq.crl CDCl3 {C:\NMR} mdsgrp 25

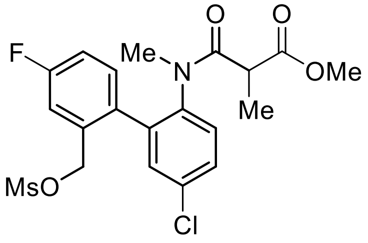

S27

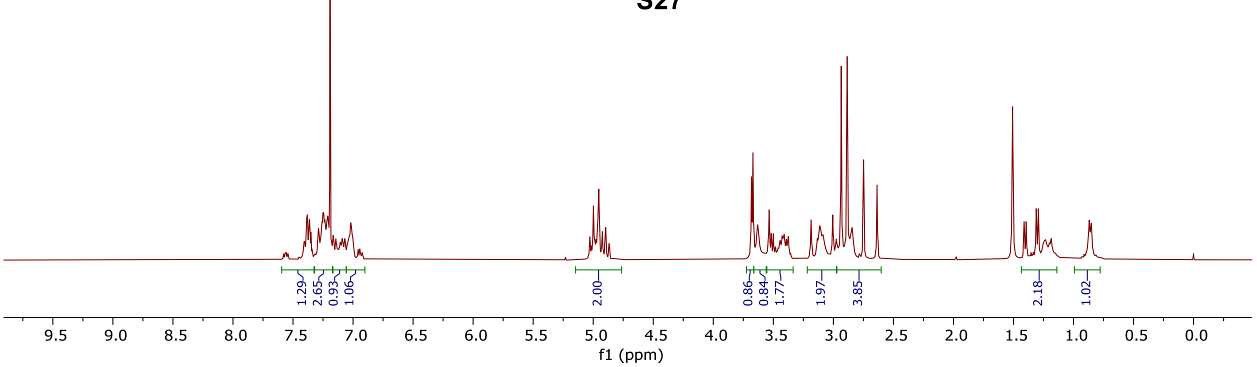

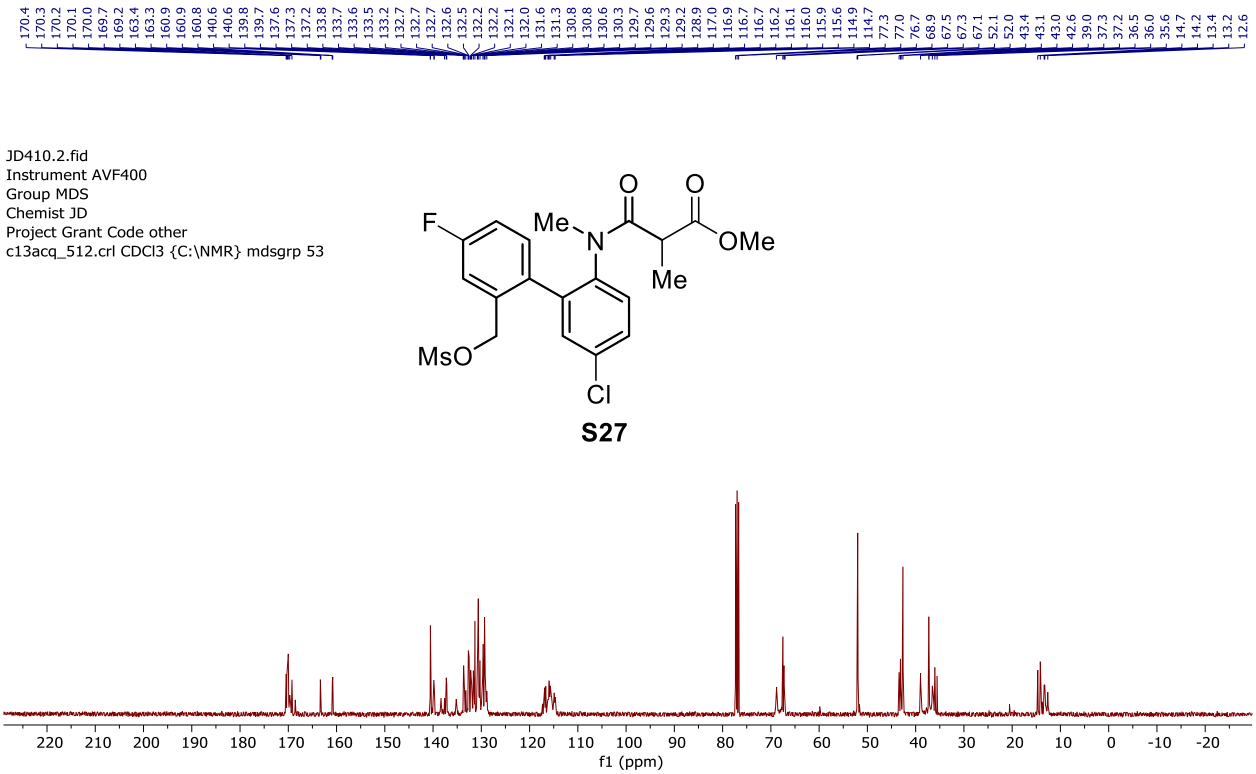
170.4
170.3
170.2
170.1
170.0
169.7
169.2
163.4
163.3
160.9
160.9
160.8
140.6
140.6
139.8
139.7
137.6
137.3
137.2
133.8
133.7
133.6
133.5
133.2
132.7
132.7
132.7
132.6
132.5
132.2
132.2
132.1
132.0
131.6
131.3
130.8
130.8
130.6
130.3
129.7
129.6
129.3
129.2
128.9
117.0
116.9
116.7
116.7
116.7
116.2
116.1
116.0
115.9
115.6
114.9
114.7
77.3
77.0
76.7
68.9
67.5
67.3
67.1
52.1
52.0
43.4
43.1
43.0
42.6
39.0
37.3
37.2
36.5
36.0
35.6
14.7
14.2
13.4
13.2
12.6
JD410.2.fid
Instrument AVF400
Group MDS
Chemist JD
Project Grant Code other
c13acq_512.crl CDCl3 {C:\NMR} mdsgrp 53
F
Me
N
O
O
OMe
Me
MsO
Cl
S27
220 210 200 190 180 170 160 150 140 130 120 110 100 90 80 70 60 50 40 30 20 10 0 -10 -20
f1 (ppm)

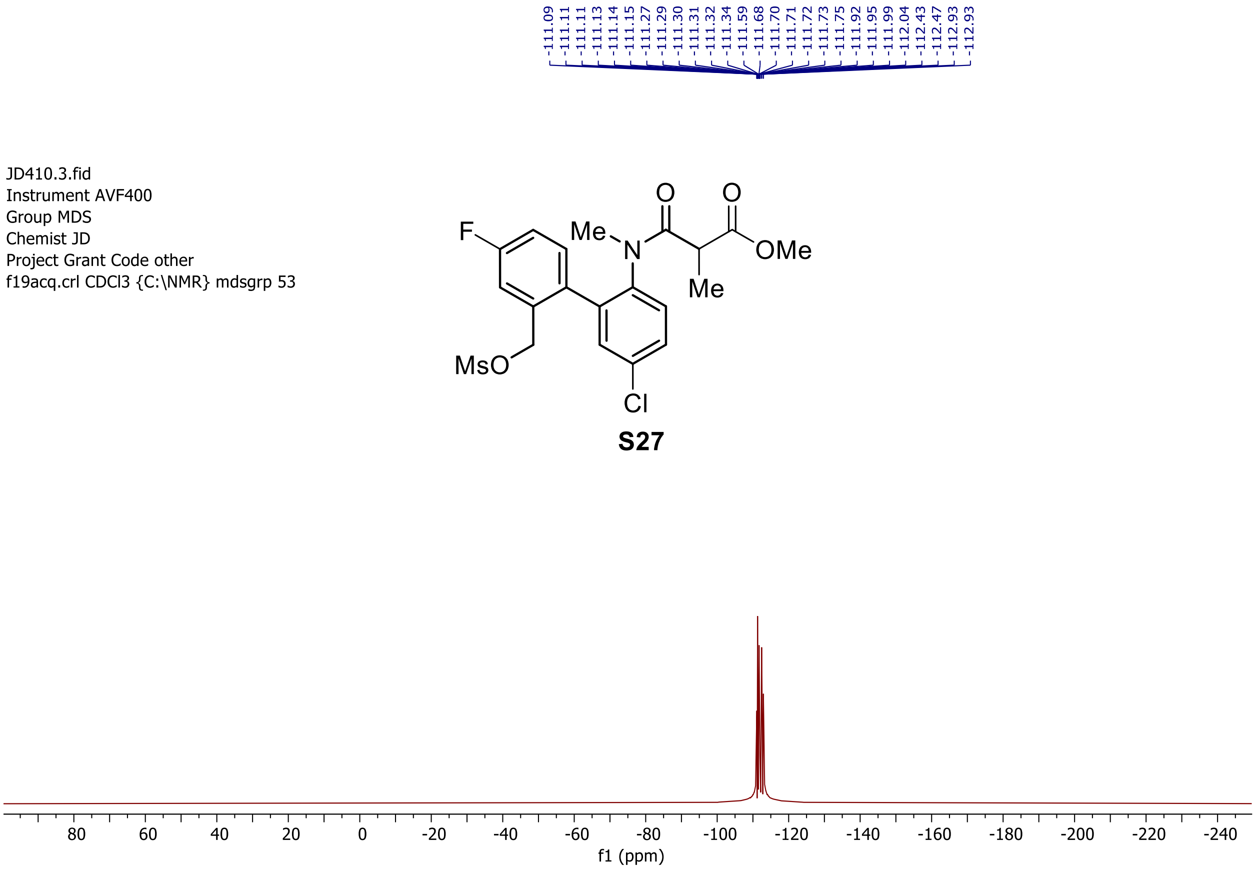
-111.09
-111.11
-111.11
-111.13
-111.14
-111.15
-111.27
-111.29
-111.30
-111.31
-111.32
-111.34
-111.59
-111.68
-111.70
-111.71
-111.72
-111.73
-111.75
-111.92
-111.95
-111.99
-112.04
-112.43
-112.47
-112.93
-112.93
JD410.3.fid
Instrument AVF400
Group MDS
Chemist JD
Project Grant Code other
f19acq.crl CDCl3 {C:\NMR} mdsgrp 53
F
Me
N
O
O
OMe
Me
MsO
Cl
S27
80 60 40 20 0 -20 -40 -60 -80 -100 -120 -140 -160 -180 -200 -220 -240
f1 (ppm)

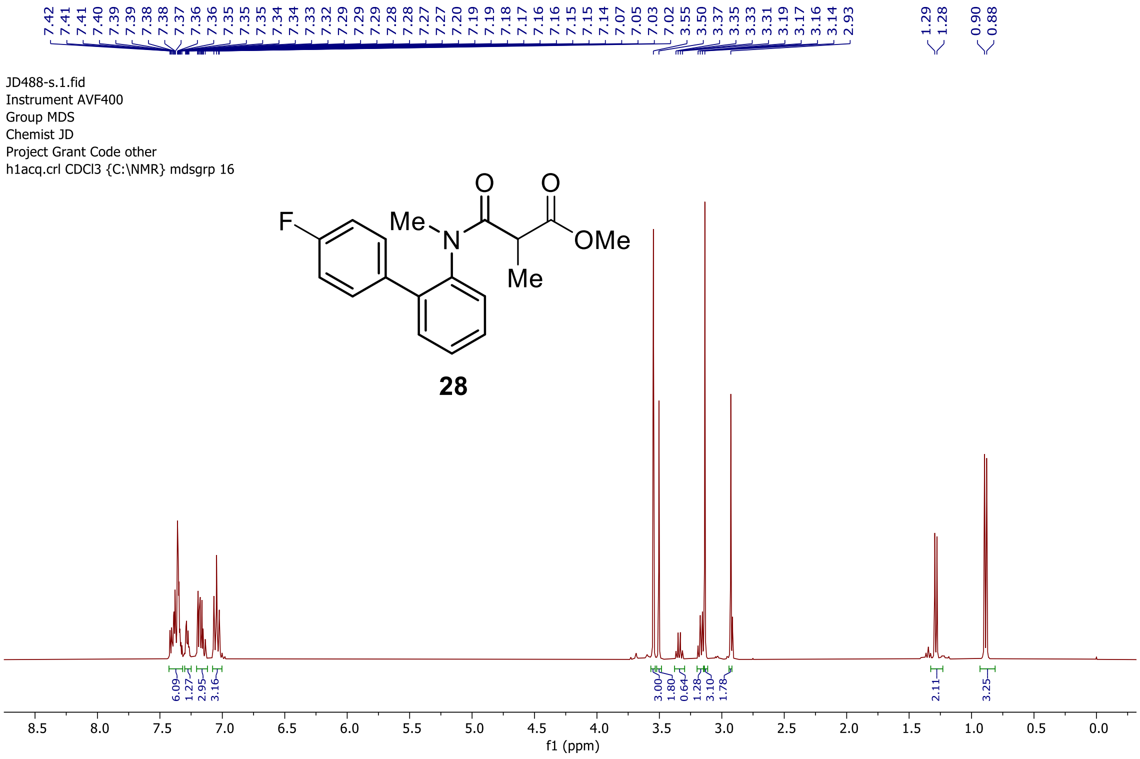
7.42 7.41 7.40 7.39 7.39 7.38 7.38 7.37 7.36 7.36 7.35 7.35 7.35 7.34 7.34 7.33 7.32 7.29 7.29 7.29 7.28 7.28 7.27 7.27 7.20 7.19 7.19 7.18 7.17 7.16 7.16 7.15 7.15 7.14 7.07 7.05 7.03 7.02 3.55 3.50 3.37 3.35 3.33 3.31 3.19 3.17 3.16 3.14 2.93
1.29 1.28 0.90 0.88
JD488-s.1.fid
Instrument AVF400
Group MDS
Chemist JD
Project Grant Code other
h1acq.crl CDCl3 {C:\NMR} mdsgrp 16
F
Me
N
O
O
OMe
Me
28
6.09 1.27 2.95 3.16 3.00 1.80 0.64 1.28 3.10 1.78 2.11 3.25
8.5 8.0 7.5 7.0 6.5 6.0 5.5 5.0 4.5 4.0 3.5 3.0 2.5 2.0 1.5 1.0 0.5 0.0
f1 (ppm)

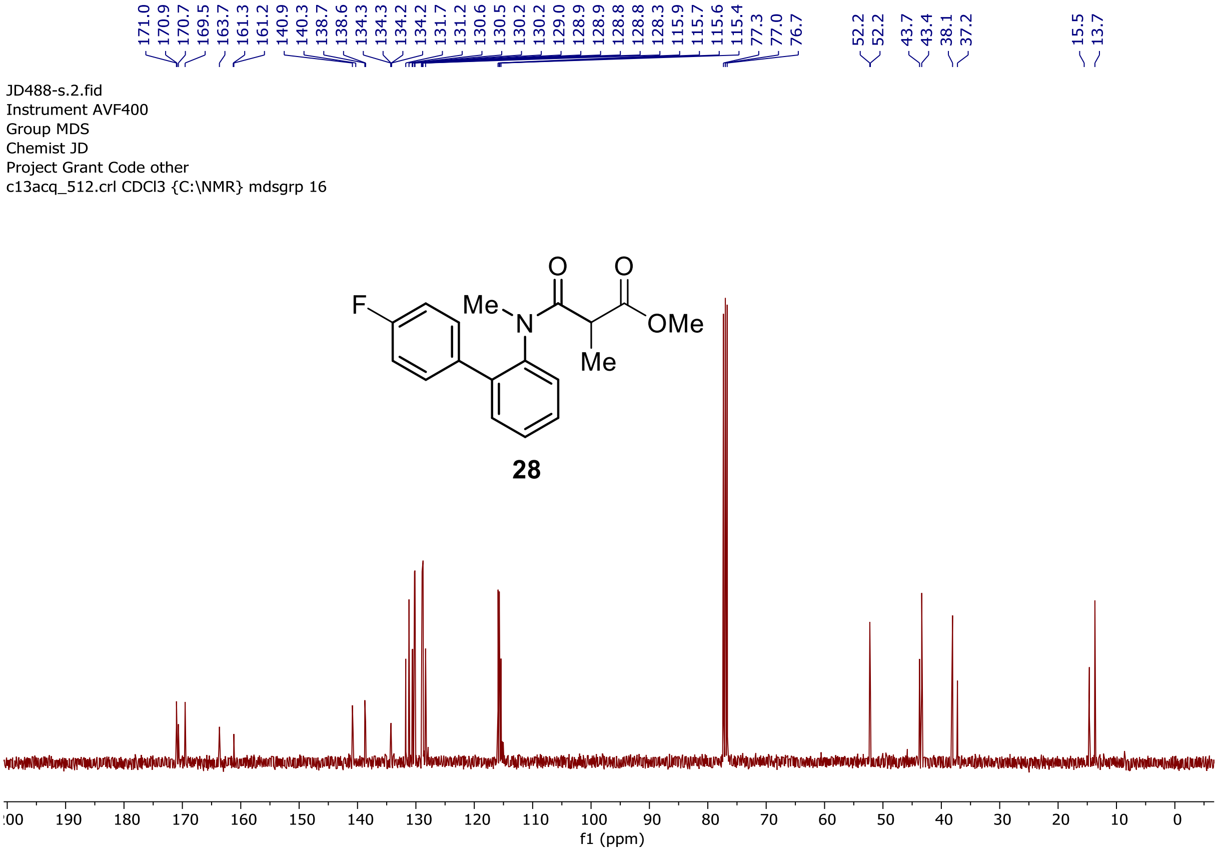
171.0 170.9 170.7 169.5 163.7 161.3 161.2 140.9 140.3 138.7 138.6 134.3 134.3 134.2 134.2 131.7 131.2 130.6 130.5 130.2 130.2 129.0 128.9 128.9 128.8 128.8 128.3 115.9 115.7 115.6 115.4 77.3 77.0 76.7
52.2 52.2 43.7 43.4 38.1 37.2 15.5 13.7
JD488-s.2.fid
Instrument AVF400
Group MDS
Chemist JD
Project Grant Code other
c13acq_512.crl CDCl3 {C:\NMR} mdsgrp 16
F
Me
N
O
O
OMe
Me
28
190 180 170 160 150 140 130 120 110 100 90 80 70 60 50 40 30 20 10 0
f1 (ppm)

JD488-tol.2.fid
Instrument AVF400
Group MDS
Chemist JD
Project Grant Code other
f19dec.crl Tol {C:\NMR} mdsgrp 8

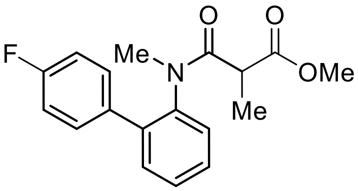

28

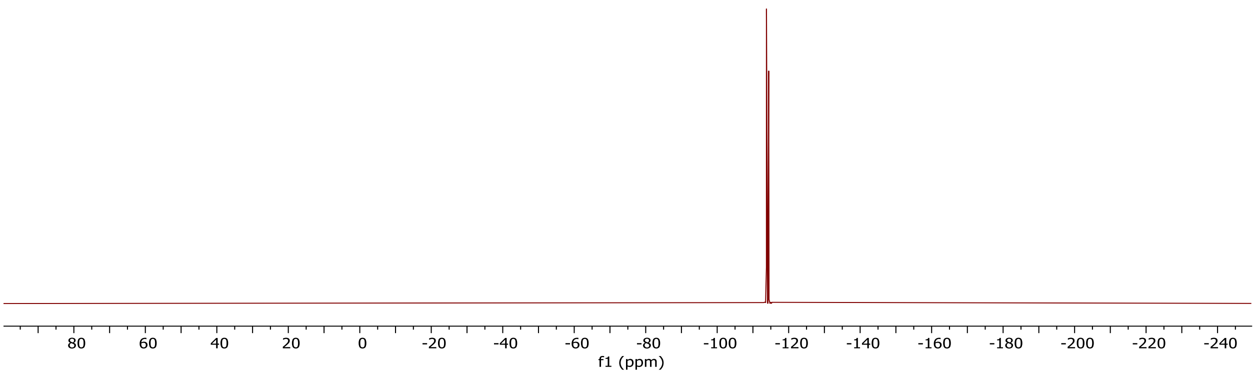

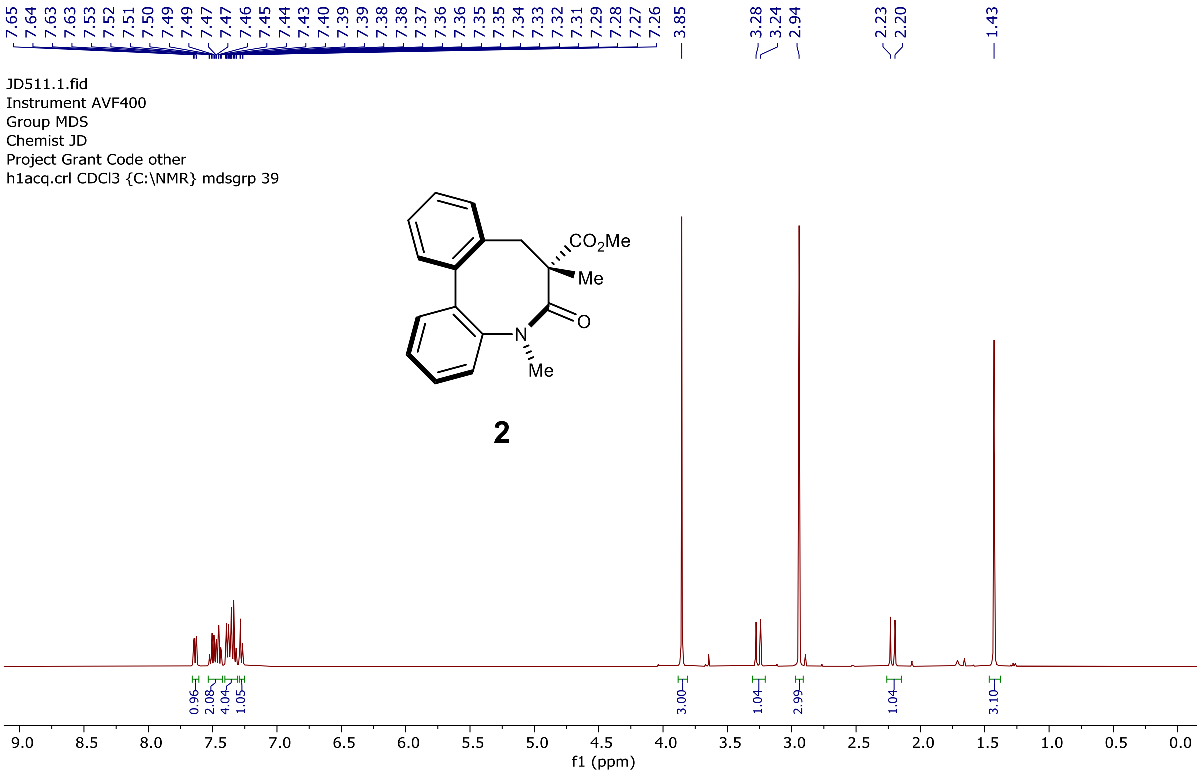
7.65 7.64 7.63 7.63 7.53 7.52 7.51 7.50 7.49 7.49 7.47 7.47 7.46 7.45 7.44 7.43 7.40 7.39 7.39 7.38 7.38 7.37 7.36 7.36 7.35 7.35 7.34 7.33 7.32 7.31 7.29 7.28 7.27 7.26 3.85 3.28 3.24 2.94 2.23 2.20 1.43
JD511.1.fid
Instrument AVF400
Group MDS
Chemist JD
Project Grant Code other
h1acq.crl CDCl3 {C:\NMR} mdsgrp 39
CO2Me
Me
O
N
Me
2
0.96 2.08 4.04 1.05 3.00 1.04 2.99 1.04 3.10
9.0 8.5 8.0 7.5 7.0 6.5 6.0 5.5 5.0 4.5 4.0 3.5 3.0 2.5 2.0 1.5 1.0 0.5 0.0
f1 (ppm)

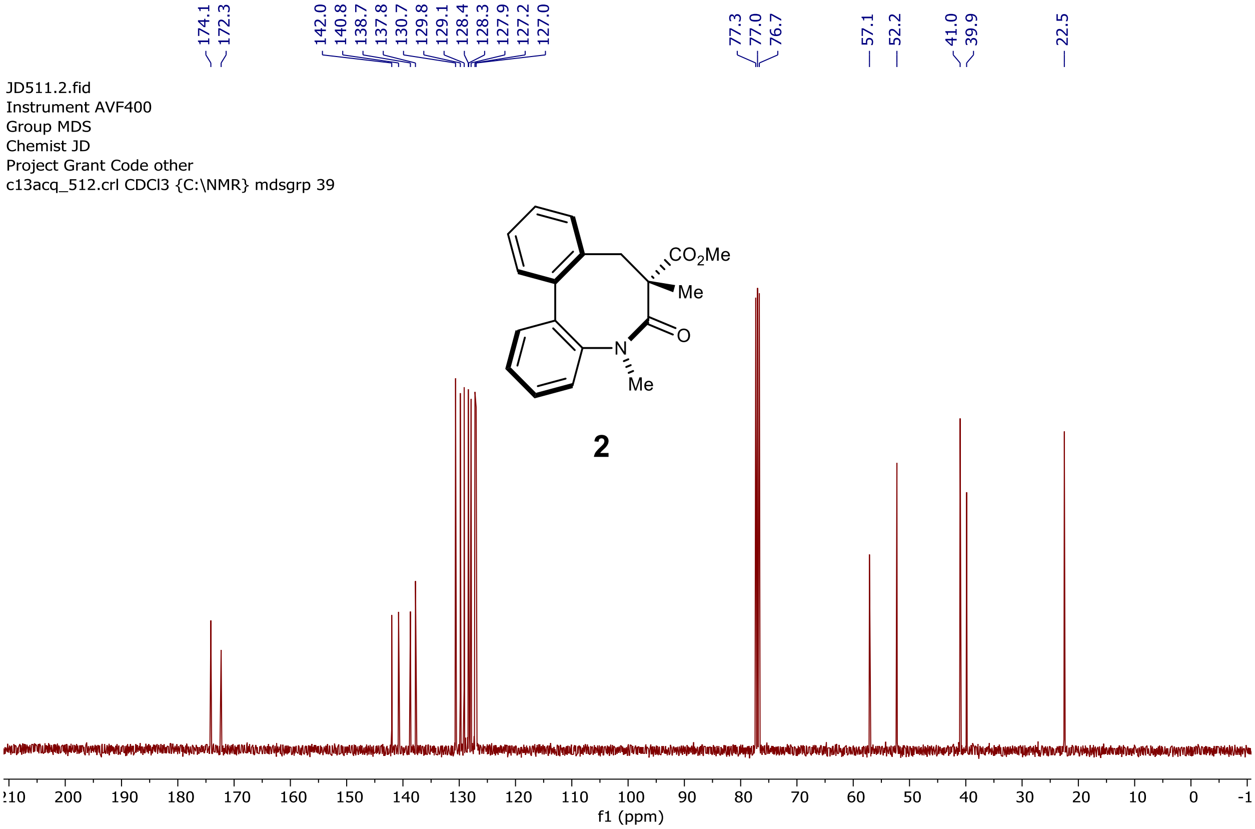
174.1 172.3 142.0 140.8 138.7 137.8 130.7 129.8 129.1 128.4 128.3 127.9 127.2 127.0 77.3 77.0 76.7 57.1 52.2 41.0 39.9 22.5
JD511.2.fid
Instrument AVF400
Group MDS
Chemist JD
Project Grant Code other
c13acq_512.crl CDCl3 {C:\NMR} mdsgrp 39
CO2Me
Me
O
N
Me
2
210 200 190 180 170 160 150 140 130 120 110 100 90 80 70 60 50 40 30 20 10 0 -1
f1 (ppm)

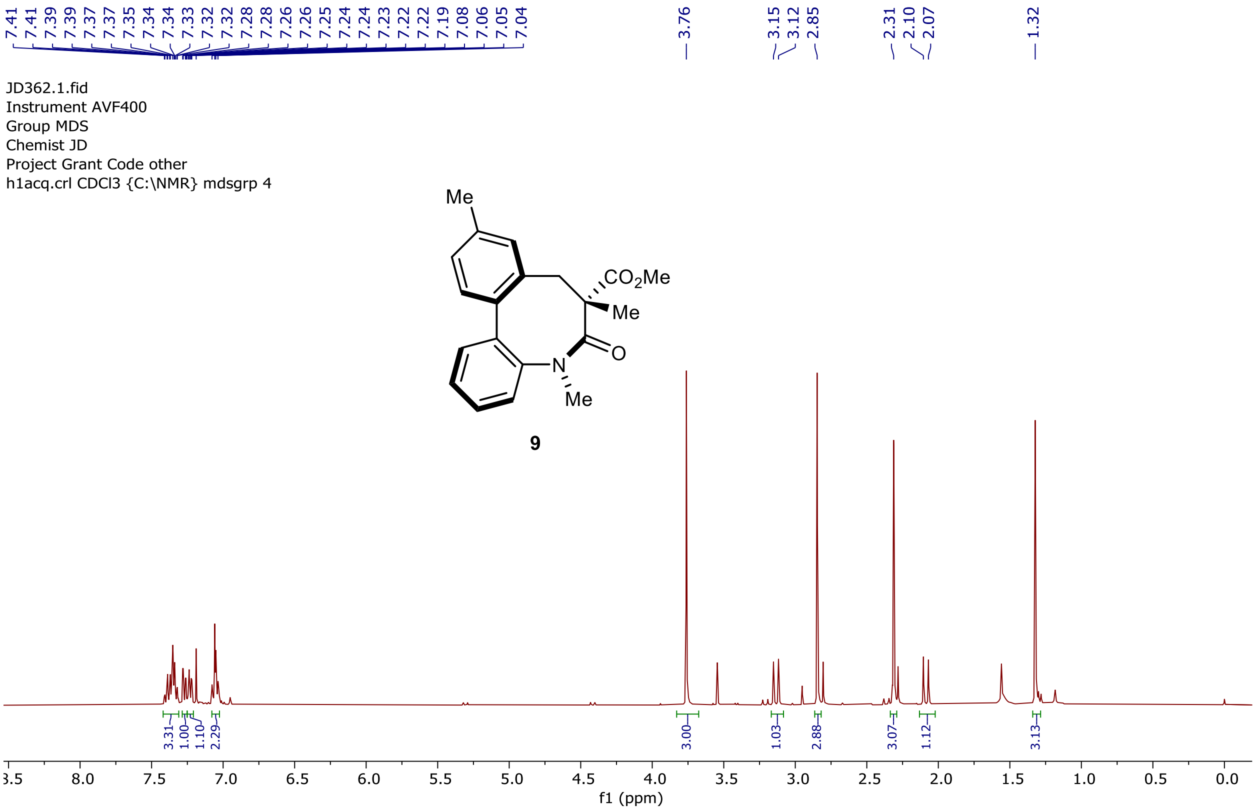
7.41 7.41 7.39 7.39 7.37 7.37 7.35 7.34 7.34 7.33 7.32 7.32 7.28 7.26 7.26 7.25 7.24 7.24 7.23 7.22 7.22 7.19 7.08 7.06 7.05 7.04
3.76
3.15 3.12 2.85
2.31 2.10 2.07
1.32
JD362.1.fid
Instrument AVF400
Group MDS
Chemist JD
Project Grant Code other
h1acq.crl CDCl3 {C:\NMR} mdsgrp 4
Me
CO2Me
Me
N
O
Me
9
3.31 1.00 1.10 2.29 3.00 1.03 2.88 3.07 1.12 3.13
f1 (ppm)

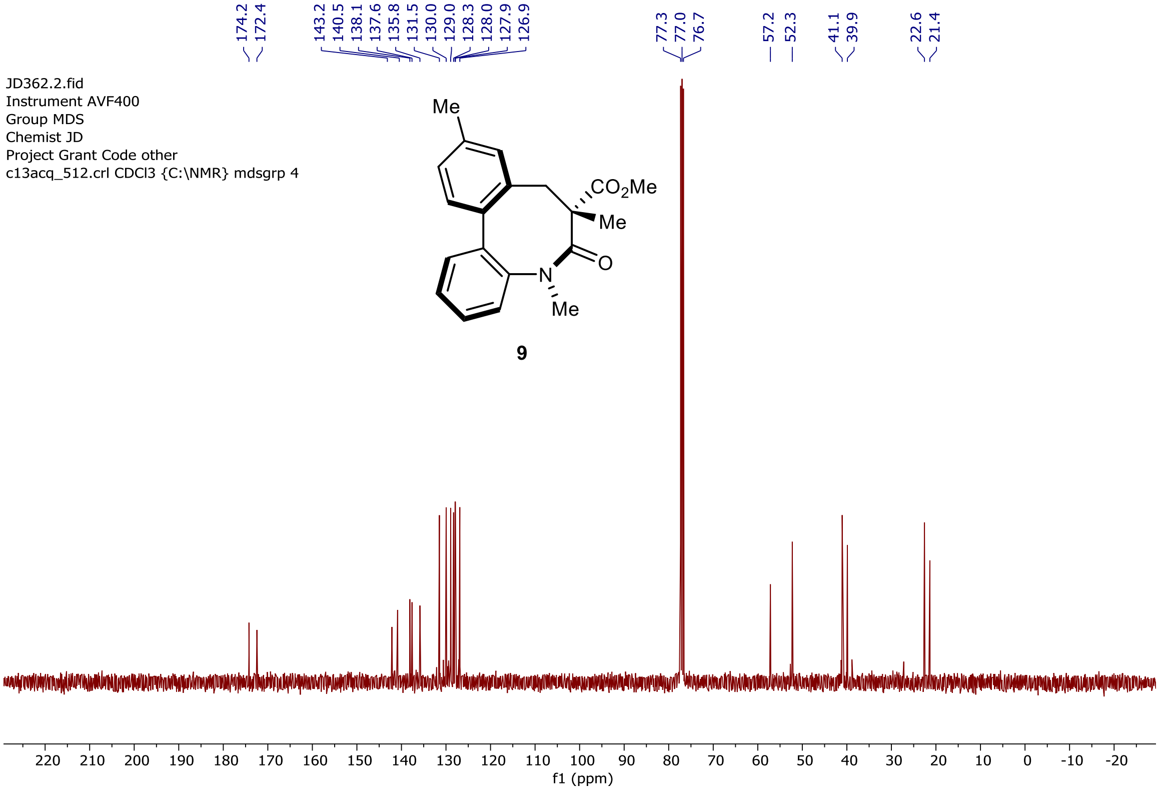
174.2 172.4
143.2 140.5 138.1 137.6 135.8 131.5 130.0 129.0 128.3 128.0 127.9 126.9
77.3 77.0 76.7
57.2 52.3
41.1 39.9
22.6 21.4
JD362.2.fid
Instrument AVF400
Group MDS
Chemist JD
Project Grant Code other
c13acq_512.crl CDCl3 {C:\NMR} mdsgrp 4
Me
CO2Me
Me
N
O
Me
9
f1 (ppm)

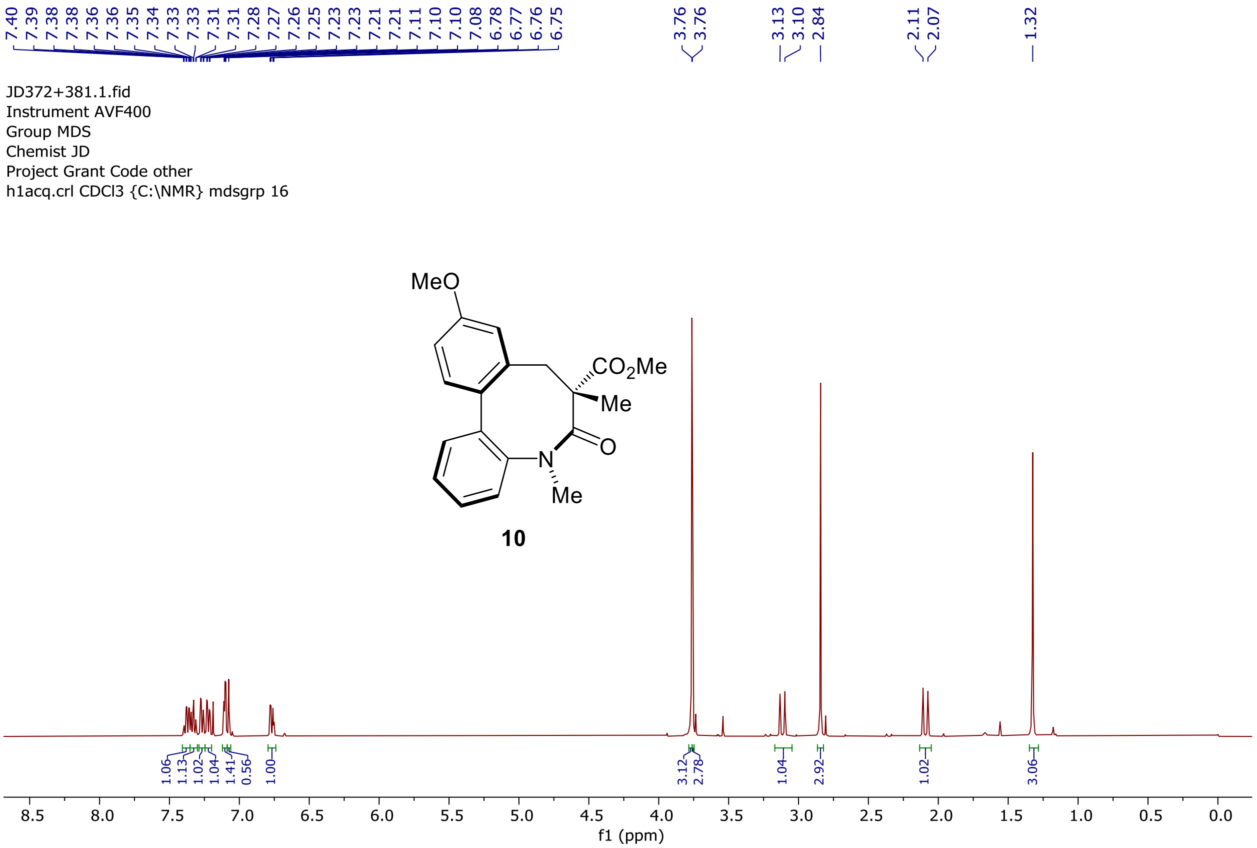
7.40 7.39 7.38 7.38 7.36 7.36 7.35 7.34 7.33 7.33 7.31 7.31 7.28 7.27 7.26 7.25 7.23 7.23 7.21 7.21 7.21 7.11 7.10 7.10 7.08 6.78 6.77 6.76 6.75
3.76 3.76
3.13 3.10 2.84
2.11 2.07
1.32
JD372+381.1.fid
Instrument AVF400
Group MDS
Chemist JD
Project Grant Code other
h1acq.crl CDCl3 {C:\NMR} mdsgrp 16
MeO
CO2Me
Me
O
N
Me
10
1.06 1.13 1.02 1.04 1.41 0.56 1.00
3.12 2.78
1.04 2.92
1.02
3.06
8.5 8.0 7.5 7.0 6.5 6.0 5.5 5.0 4.5 4.0 3.5 3.0 2.5 2.0 1.5 1.0 0.5 0.0
f1 (ppm)

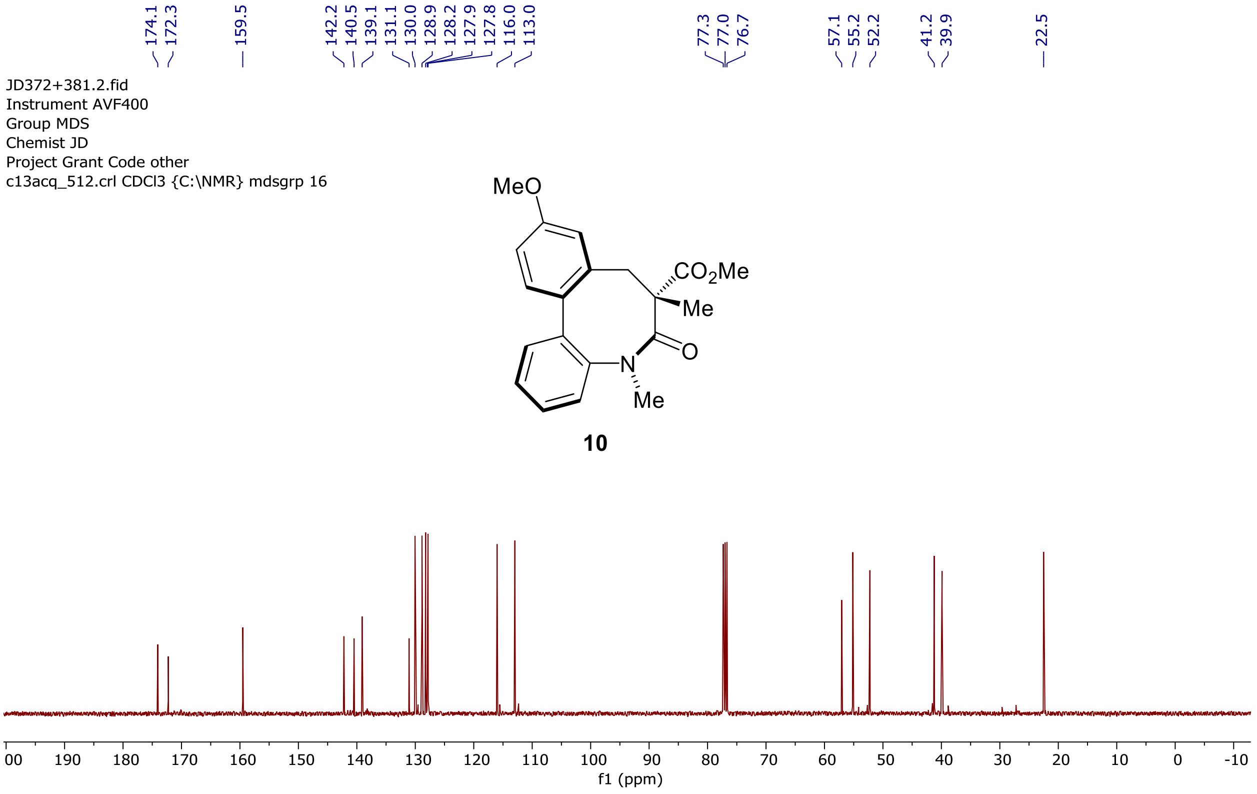
174.1 172.3
159.5
142.2 140.5 139.1 131.1 130.0 128.9 128.2 127.9 127.8 116.0 113.0
77.3 77.0 76.7
57.1 55.2 52.2
41.2 39.9
22.5
JD372+381.2.fid
Instrument AVF400
Group MDS
Chemist JD
Project Grant Code other
c13acq_512.crl CDCl3 {C:\NMR} mdsgrp 16
MeO
CO2Me
Me
O
N
Me
10
00 190 180 170 160 150 140 130 120 110 100 90 80 70 60 50 40 30 20 10 0 -10
f1 (ppm)

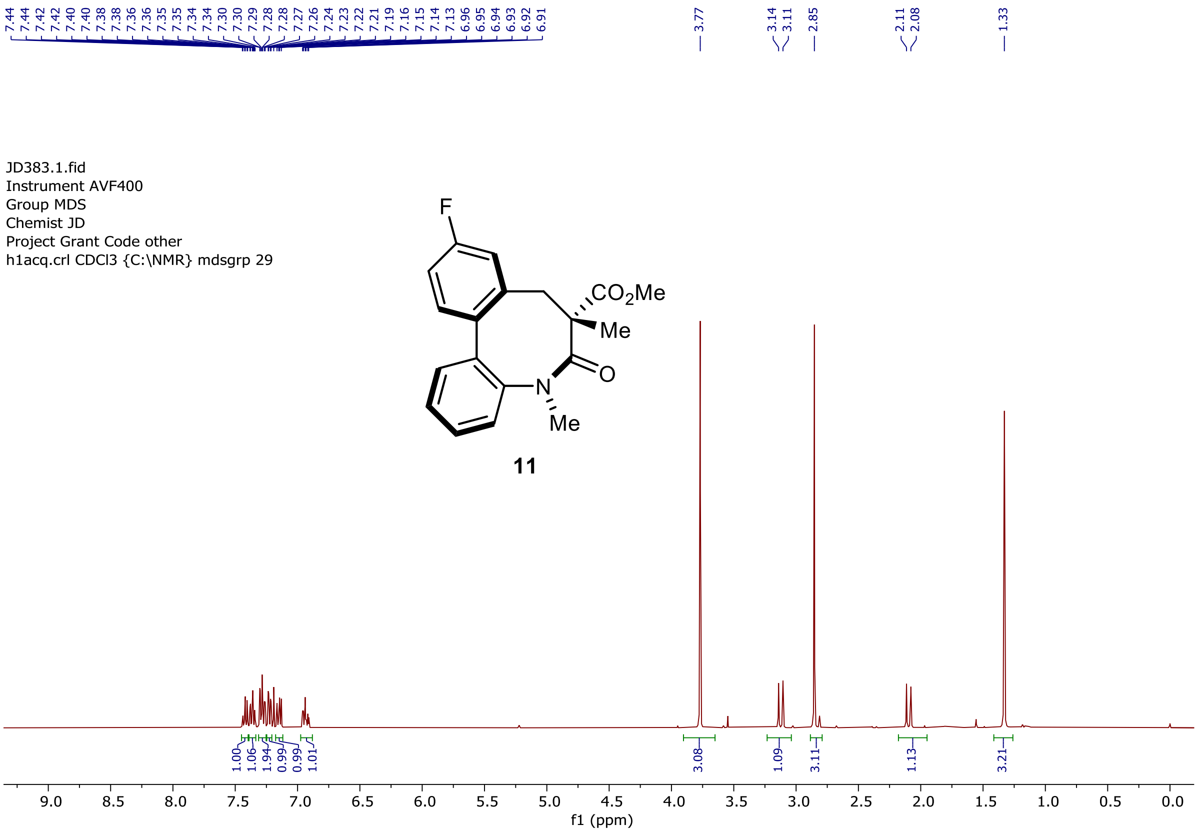
7.44 7.44 7.42 7.42 7.40 7.40 7.38 7.38 7.36 7.36 7.35 7.35 7.34 7.34 7.30 7.30 7.29 7.28 7.28 7.27 7.26 7.24 7.23 7.22 7.21 7.19 7.16 7.15 7.14 7.13 6.96 6.95 6.94 6.93 6.92 6.91
3.77
3.14 3.11
2.85
2.11 2.08
1.33
JD383.1.fid
Instrument AVF400
Group MDS
Chemist JD
Project Grant Code other
h1acq.crl CDCl3 {C:\NMR} mdsgrp 29
F
CO_2Me
Me
O
N
Me
11
1.00 1.06 1.94 0.99 0.99 1.01
3.08
1.09
3.11
1.13
3.21
9.0 8.5 8.0 7.5 7.0 6.5 6.0 5.5 5.0 4.5 4.0 3.5 3.0 2.5 2.0 1.5 1.0 0.5 0.0
f1 (ppm)

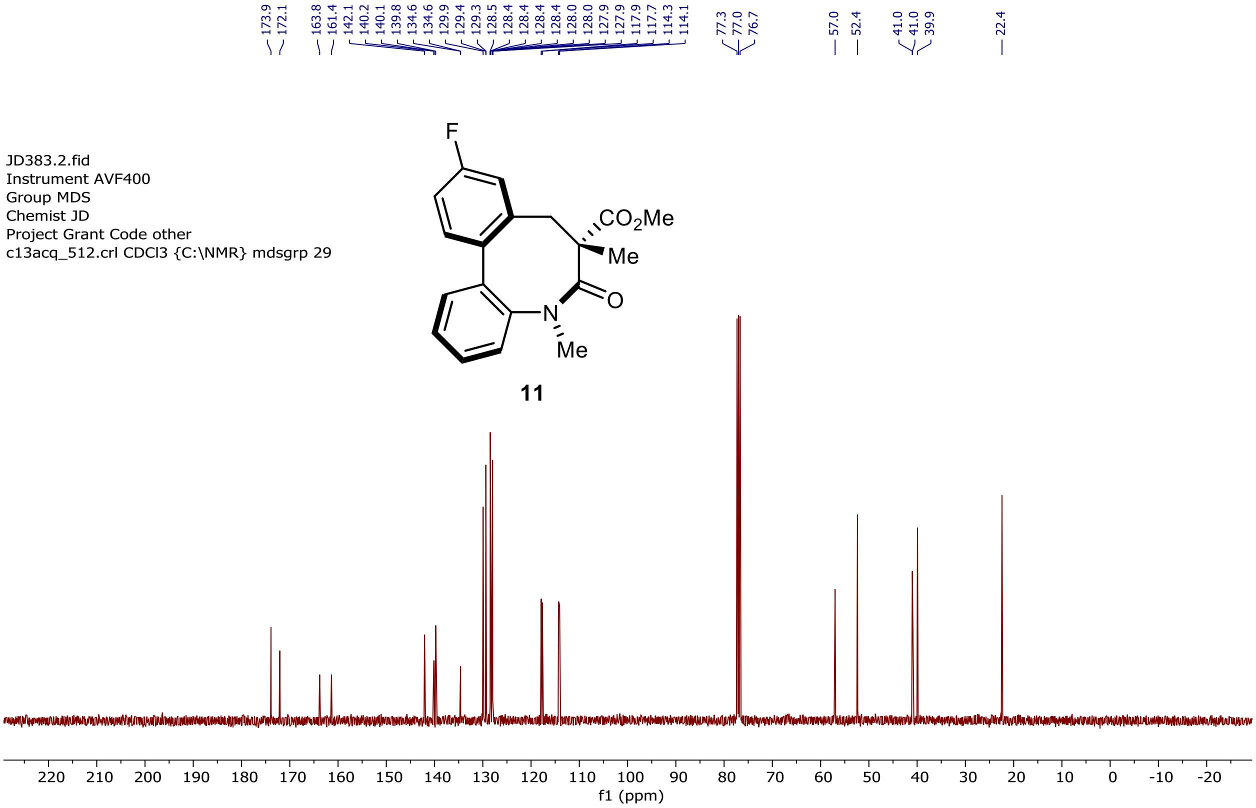
173.9 172.1 163.8 161.4 142.1 140.2 140.1 139.8 134.6 134.6 129.9 129.4 129.3 128.5 128.4 128.4 128.4 128.0 128.0 127.9 127.9 117.9 117.7 114.3 114.1
77.3 77.0 76.7
57.0
52.4
41.0 41.0 39.9
22.4
JD383.2.fid
Instrument AVF400
Group MDS
Chemist JD
Project Grant Code other
c13acq_512.crl CDCl3 {C:\NMR} mdsgrp 29
F
CO_2Me
Me
O
N
Me
11
220 210 200 190 180 170 160 150 140 130 120 110 100 90 80 70 60 50 40 30 20 10 0 -10 -20
f1 (ppm)

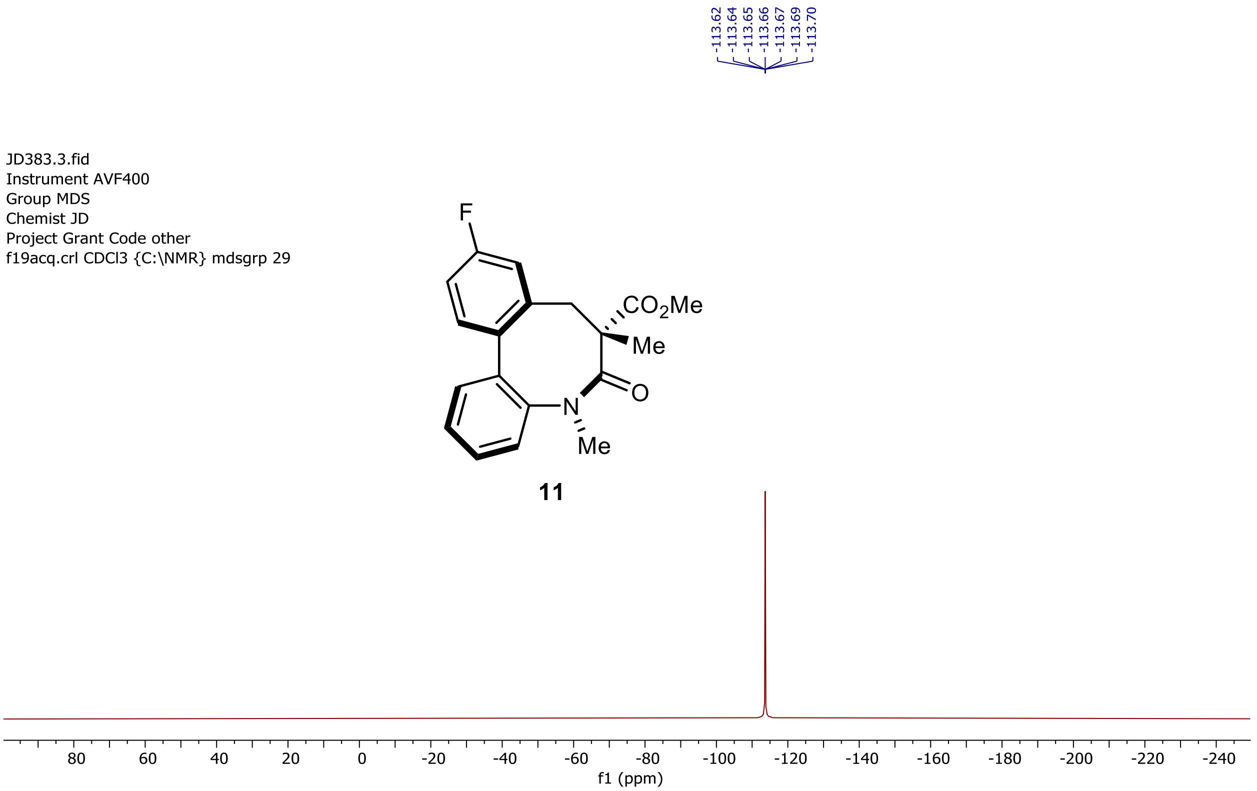
-113.62
-113.64
-113.65
-113.66
-113.67
-113.69
-113.70
JD383.3.fid
Instrument AVF400
Group MDS
Chemist JD
Project Grant Code other
f19acq.crl CDCl3 {C:\NMR} mdsgrp 29
F
CO_2Me
Me
O
N
Me
11
80 60 40 20 0 -20 -40 -60 -80 -100 -120 -140 -160 -180 -200 -220 -240
f1 (ppm)

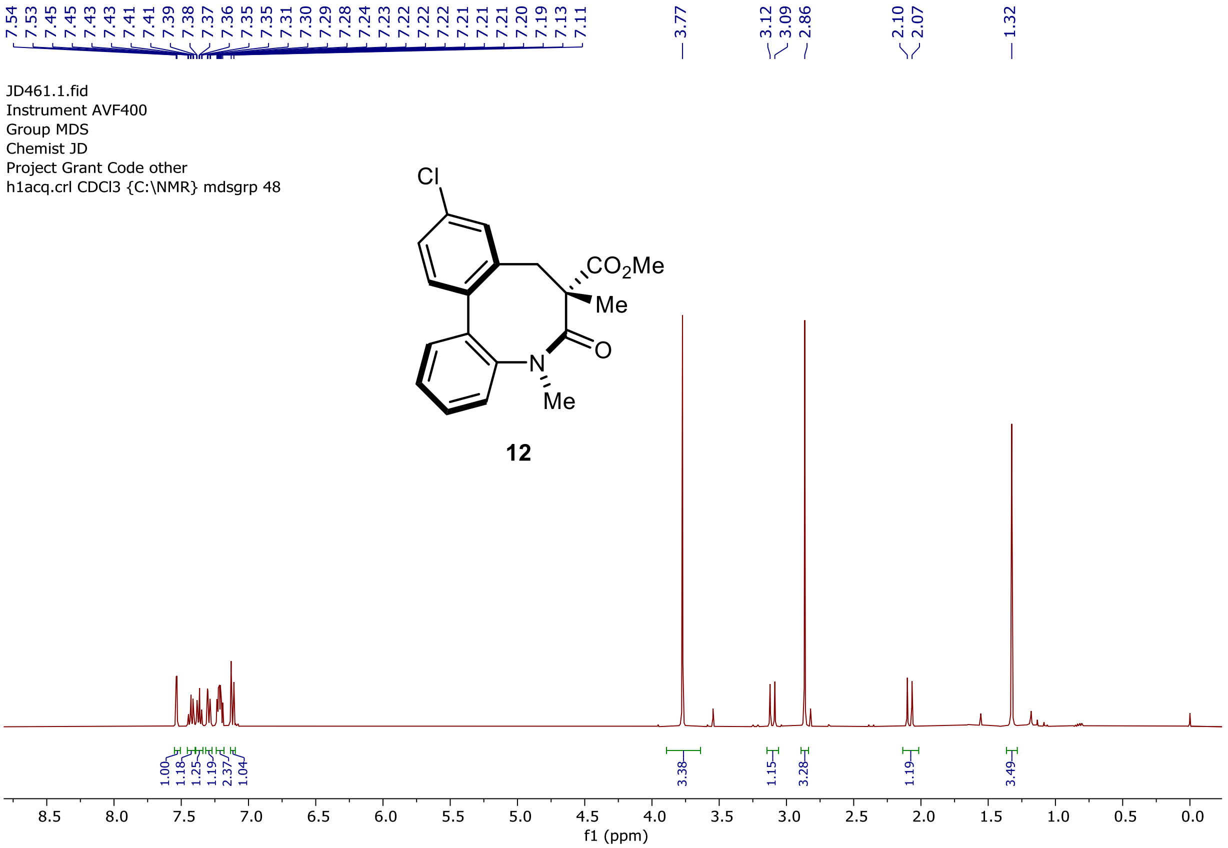
7.54
7.53
7.45
7.45
7.43
7.43
7.41
7.41
7.39
7.38
7.37
7.36
7.35
7.35
7.31
7.30
7.29
7.28
7.24
7.23
7.22
7.22
7.22
7.21
7.21
7.21
7.20
7.19
7.13
7.11
3.77
3.12
3.09
2.86
2.10
2.07
1.32
JD461.1.fid
Instrument AVF400
Group MDS
Chemist JD
Project Grant Code other
h1acq.crl CDCl3 {C:\NMR} mdsgrp 48
Cl
CO_2Me
Me
O
N
Me
12
1.00
1.18
1.25
1.19
2.37
1.04
3.38
1.15
3.28
1.19
3.49
8.5 8.0 7.5 7.0 6.5 6.0 5.5 5.0 4.5 4.0 3.5 3.0 2.5 2.0 1.5 1.0 0.5 0.0
f1 (ppm)

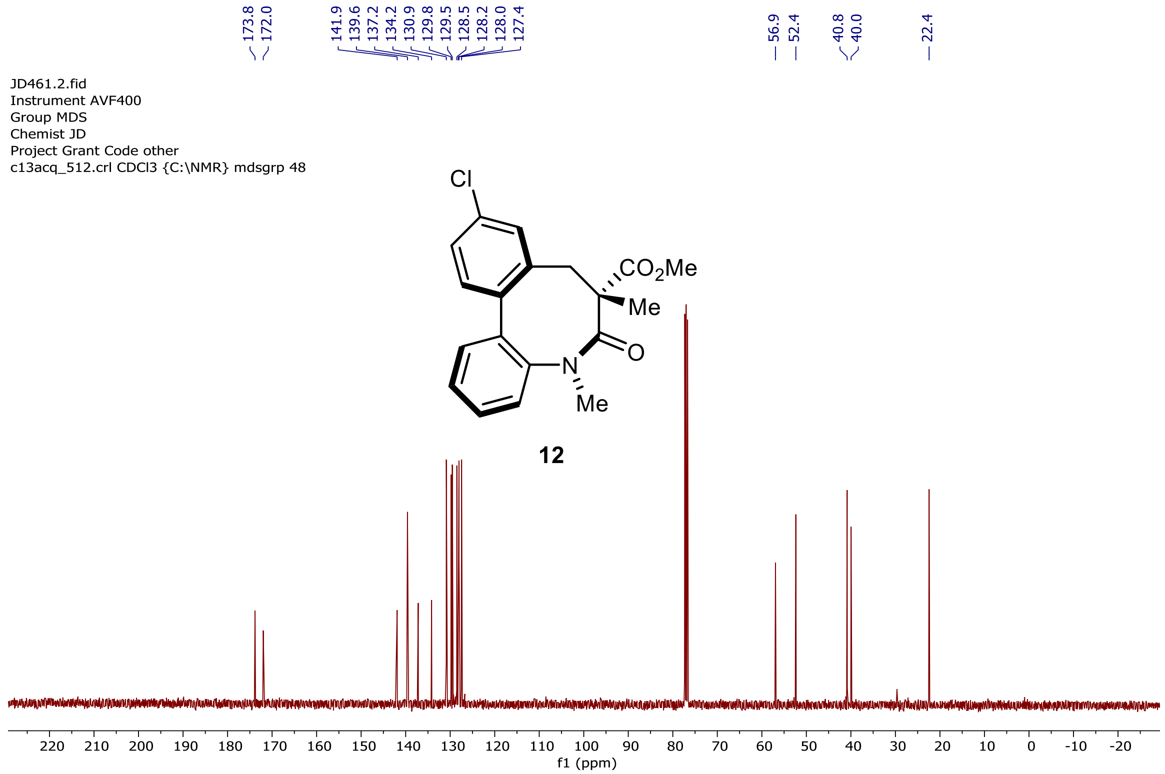
173.8
172.0
141.9
139.6
137.2
134.2
130.9
129.8
129.5
128.5
128.2
128.0
127.4
56.9
52.4
40.8
40.0
22.4
JD461.2.fid
Instrument AVF400
Group MDS
Chemist JD
Project Grant Code other
c13acq_512.crl CDCl3 {C:\NMR} mdsgrp 48
Cl
CO_2Me
Me
N
O
Me
f1 (ppm)

12

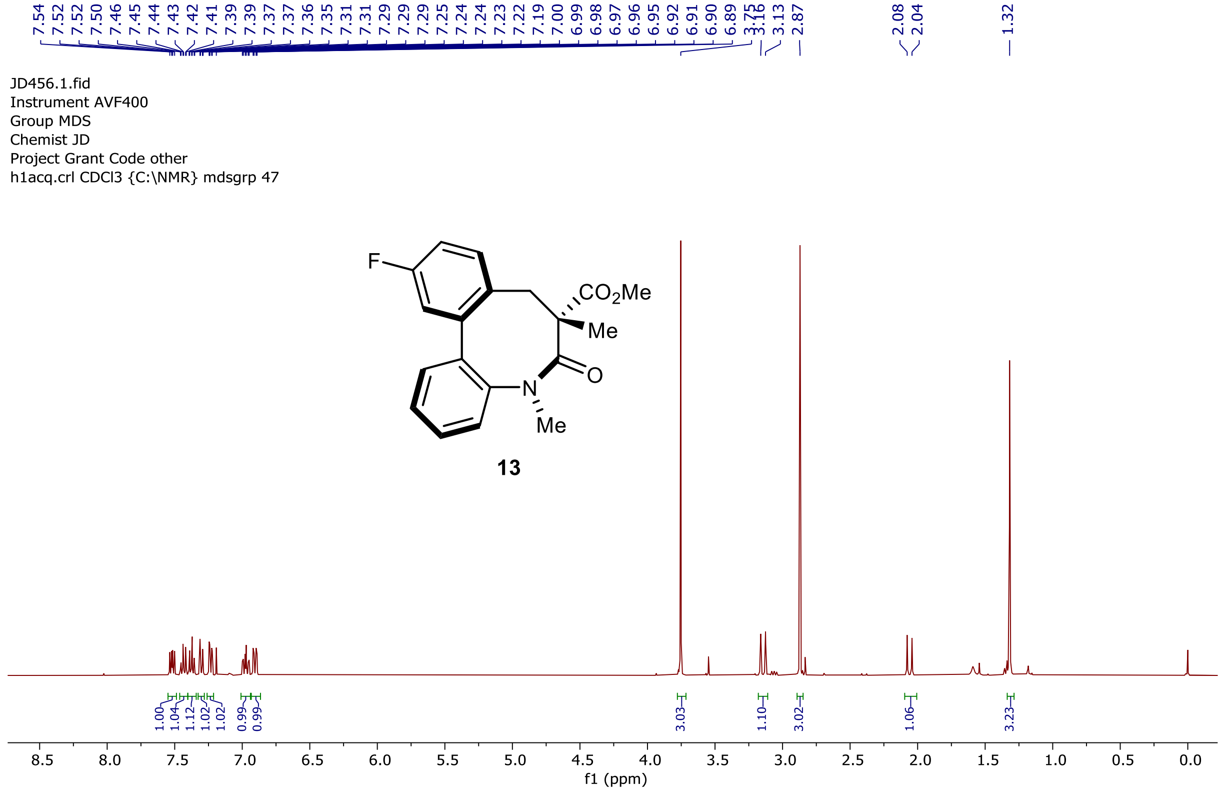
7.54
7.52
7.52
7.50
7.46
7.45
7.44
7.43
7.42
7.41
7.39
7.39
7.37
7.37
7.36
7.35
7.31
7.31
7.29
7.29
7.29
7.25
7.24
7.24
7.23
7.22
7.19
7.00
6.99
6.98
6.97
6.96
6.95
6.92
6.91
6.90
6.89
3.75
3.16
3.13
2.87
2.08
2.04
1.32
JD456.1.fid
Instrument AVF400
Group MDS
Chemist JD
Project Grant Code other
h1acq.crl CDCl3 {C:\NMR} mdsgrp 47
F
CO_2Me
Me
N
O
Me
1.00
1.04
1.12
1.02
1.02
0.99
0.99
3.03
1.10
3.02
1.06
3.23
f1 (ppm)

13

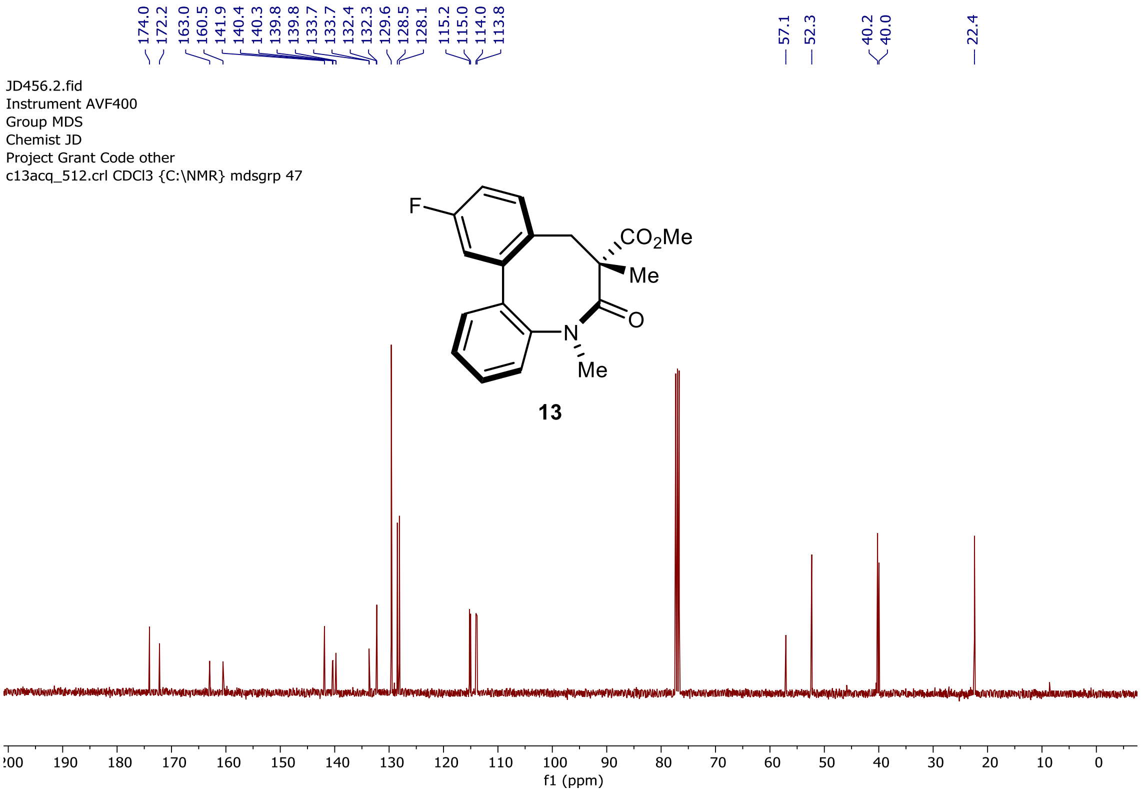
174.0
172.2
163.0
160.5
141.9
140.4
140.3
139.8
139.8
133.7
133.7
132.4
132.3
129.6
128.5
128.1
115.2
115.0
114.0
113.8
57.1
52.3
40.2
40.0
22.4
JD456.2.fid
Instrument AVF400
Group MDS
Chemist JD
Project Grant Code other
c13acq_512.crl CDCl3 {C:\NMR} mdsgrp 47
F
CO_2Me
Me
O
N
Me
13
f1 (ppm)

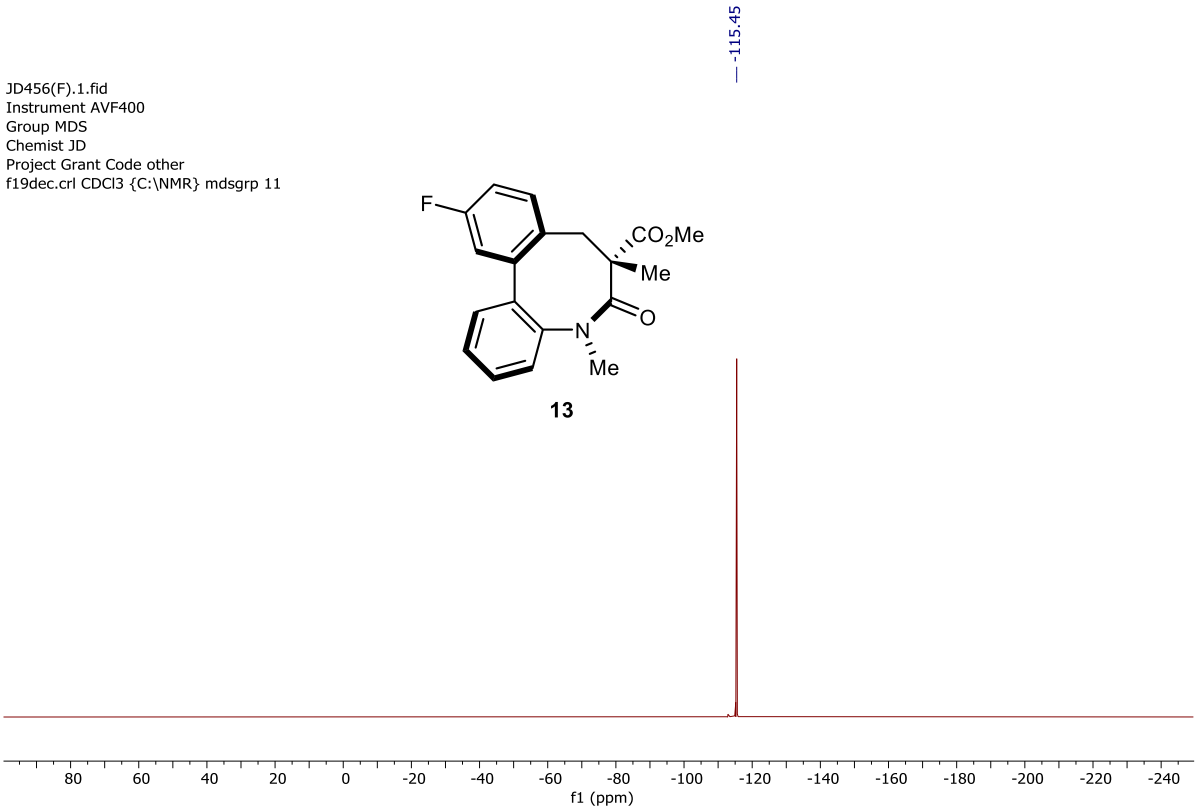
-115.45
JD456(F).1.fid
Instrument AVF400
Group MDS
Chemist JD
Project Grant Code other
f19dec.crl CDCl3 {C:\NMR} mdsgrp 11
F
CO_2Me
Me
O
N
Me
13
f1 (ppm)

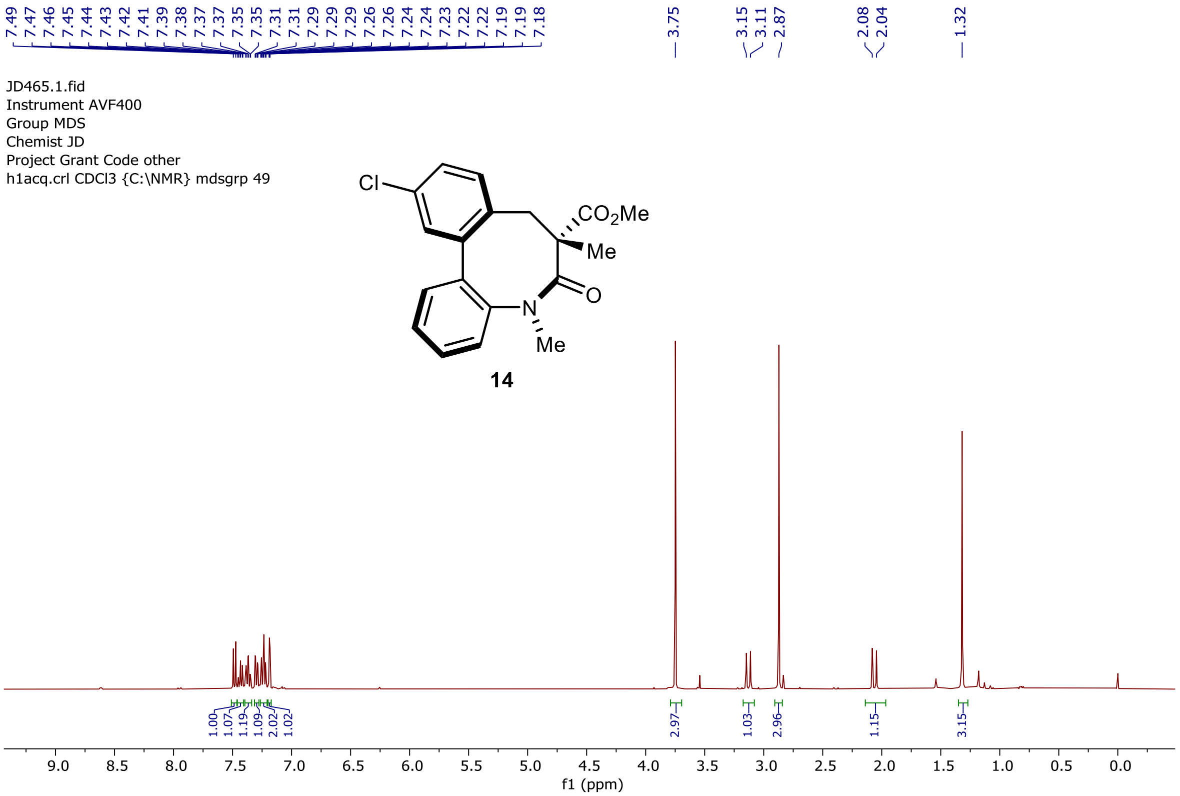
7.49 7.47 7.46 7.45 7.44 7.43 7.42 7.41 7.39 7.38 7.37 7.35 7.35 7.31 7.31 7.29 7.29 7.29 7.26 7.26 7.24 7.24 7.23 7.22 7.22 7.19 7.19 7.18
3.75
3.15 3.11 2.87
2.08 2.04
1.32
JD465.1.fid
Instrument AVF400
Group MDS
Chemist JD
Project Grant Code other
h1acq.crl CDCl3 {C:\NMR} mdsgrp 49
Cl
CO2Me
Me
O
N
Me
14
1.00 1.07 1.19 1.09 2.02 1.02
2.97
1.03
2.96
1.15
3.15
9.0 8.5 8.0 7.5 7.0 6.5 6.0 5.5 5.0 4.5 4.0 3.5 3.0 2.5 2.0 1.5 1.0 0.5 0.0
f1 (ppm)

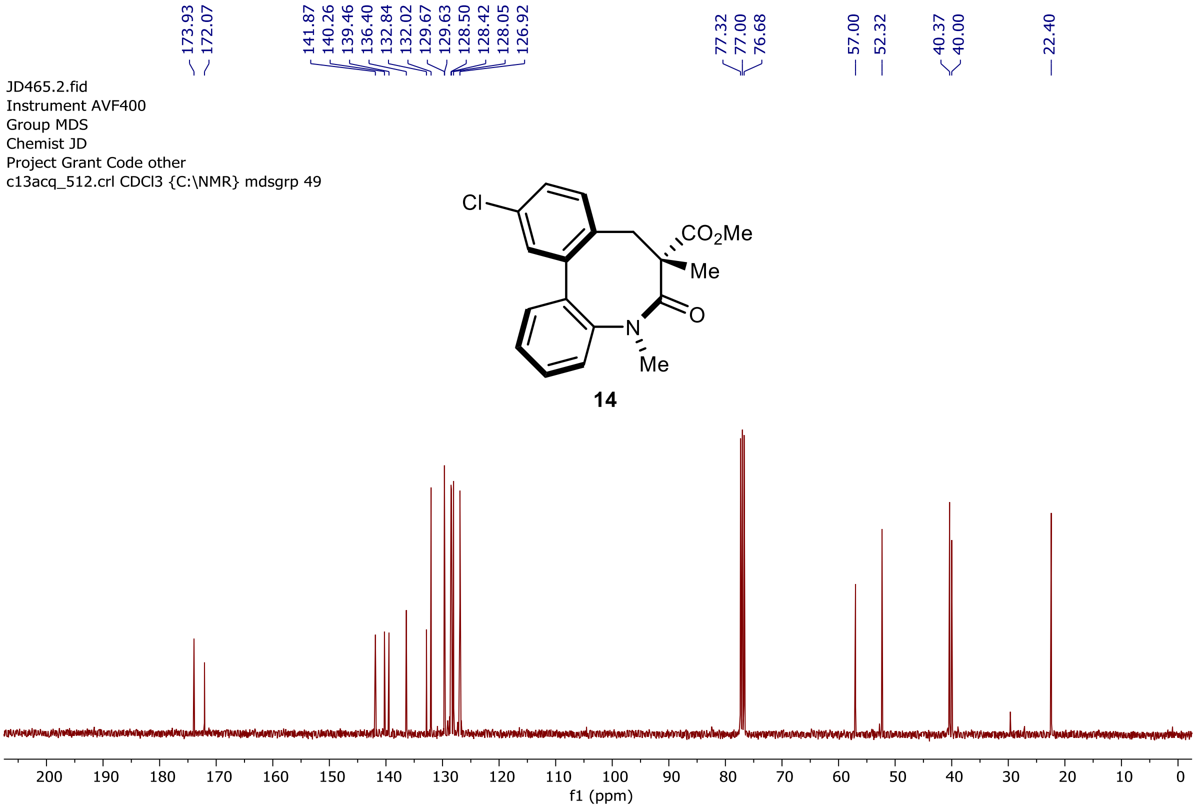
173.93 172.07
141.87 140.26 139.46 136.40 132.84 132.02 129.67 129.63 128.50 128.42 128.05 126.92
77.32 77.00 76.68
57.00
52.32
40.37 40.00
22.40
JD465.2.fid
Instrument AVF400
Group MDS
Chemist JD
Project Grant Code other
c13acq_512.crl CDCl3 {C:\NMR} mdsgrp 49
Cl
CO2Me
Me
O
N
Me
14
200 190 180 170 160 150 140 130 120 110 100 90 80 70 60 50 40 30 20 10 0
f1 (ppm)

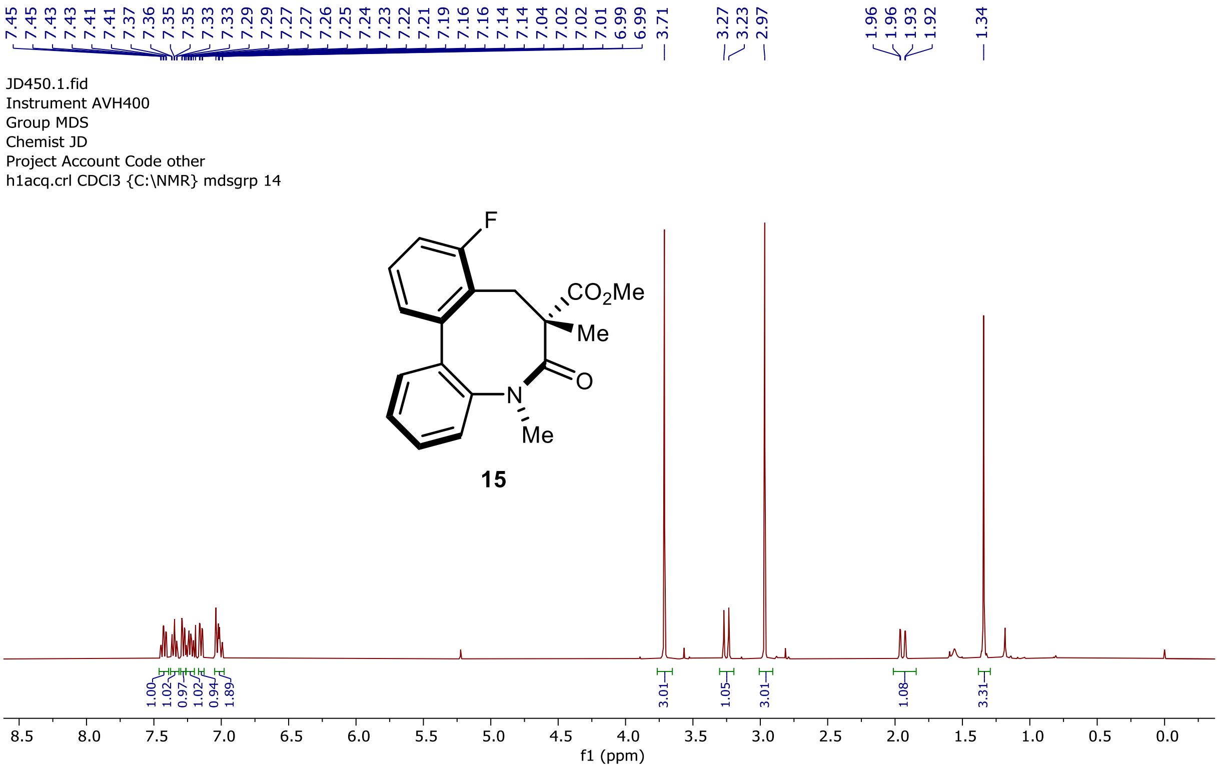
7.45 7.45 7.43 7.43 7.41 7.41 7.37 7.36 7.35 7.35 7.33 7.33 7.29 7.29 7.27 7.27 7.26 7.25 7.24 7.23 7.22 7.21 7.19 7.16 7.16 7.14 7.14 7.04 7.02 7.02 7.01 6.99 6.99 3.71 3.27 3.23 2.97 1.96 1.96 1.93 1.92 1.34
JD450.1.fid
Instrument AVH400
Group MDS
Chemist JD
Project Account Code other
h1acq.crl CDCl3 {C:\NMR} mdsgrp 14
F
CO_2Me
Me
O
N
Me
15
1.00 1.02 0.97 1.02 0.94 1.89 3.01 1.05 3.01 1.08 3.31
8.5 8.0 7.5 7.0 6.5 6.0 5.5 5.0 4.5 4.0 3.5 3.0 2.5 2.0 1.5 1.0 0.5 0.0
f1 (ppm)

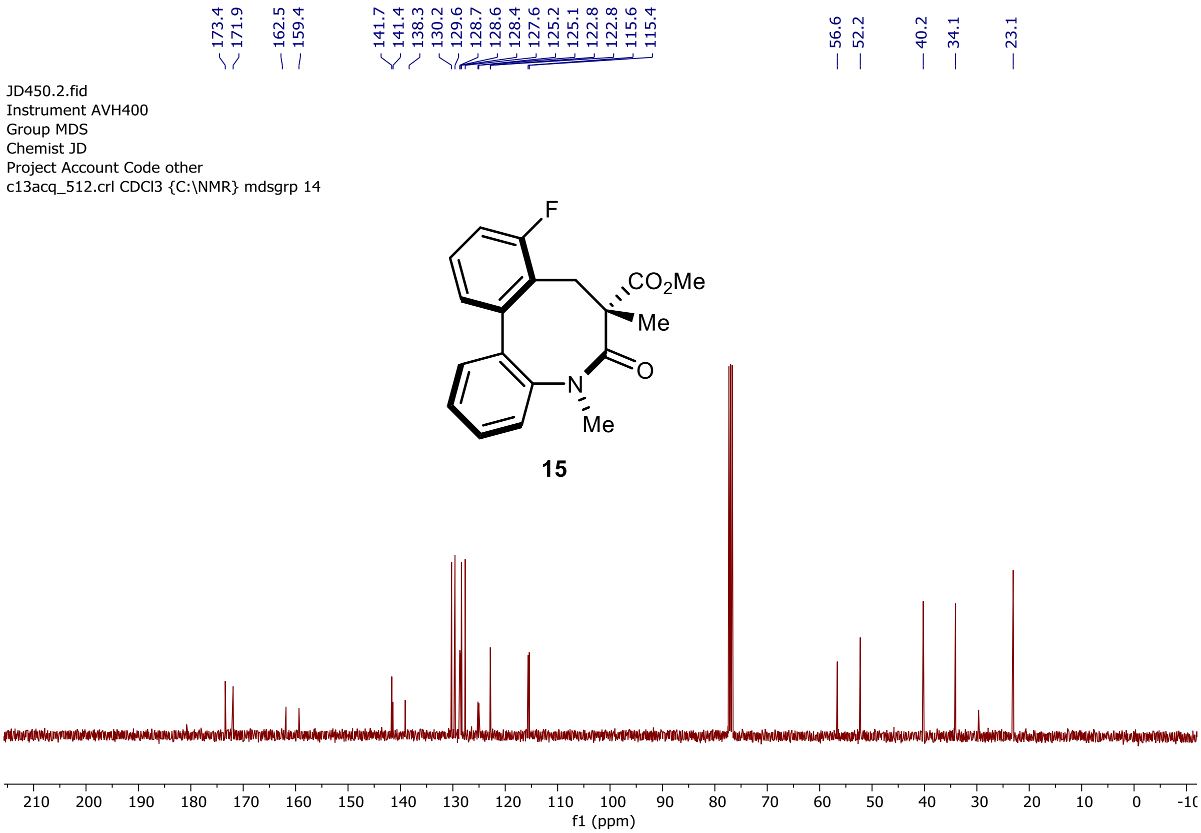
173.4 171.9 162.5 159.4 141.7 141.4 138.3 130.2 129.6 128.7 128.6 128.4 127.6 125.2 125.1 122.8 122.8 115.6 115.4 56.6 52.2 40.2 34.1 23.1
JD450.2.fid
Instrument AVH400
Group MDS
Chemist JD
Project Account Code other
c13acq_512.crl CDCl3 {C:\NMR} mdsgrp 14
F
CO_2Me
Me
O
N
Me
15
210 200 190 180 170 160 150 140 130 120 110 100 90 80 70 60 50 40 30 20 10 0 -10
f1 (ppm)

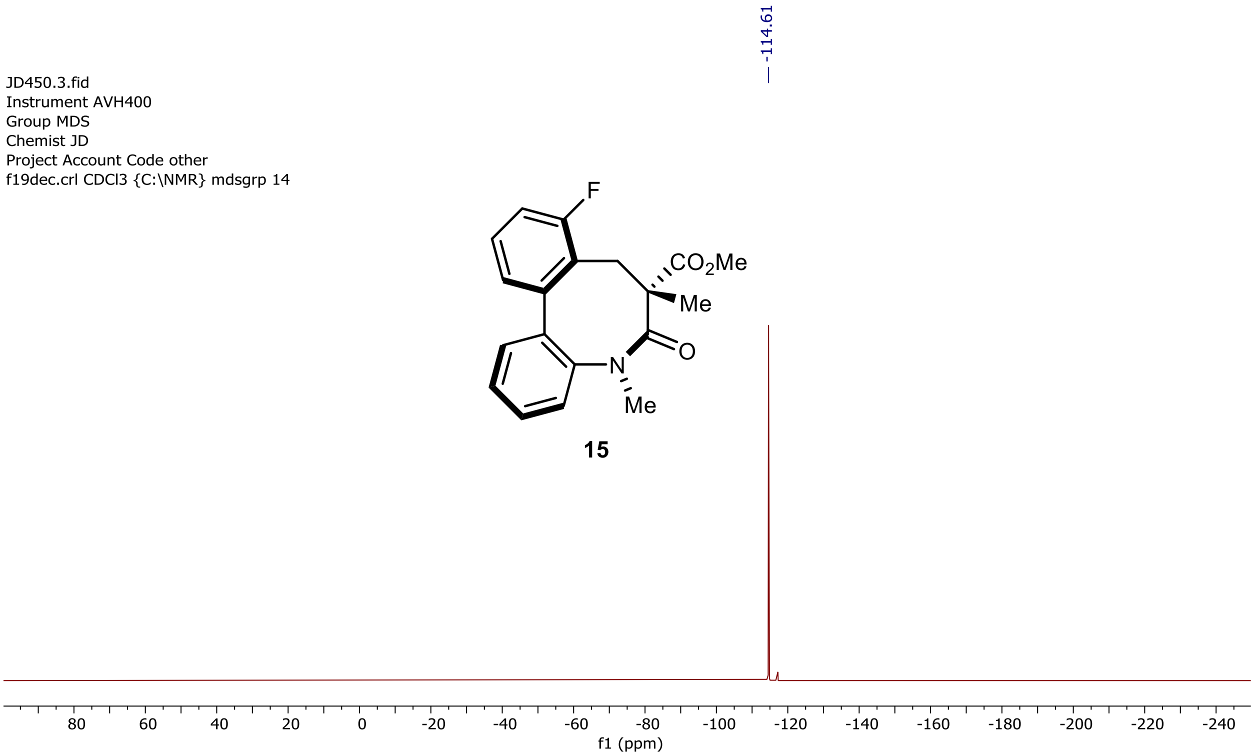
JD450.3.fid
Instrument AVH400
Group MDS
Chemist JD
Project Account Code other
f19dec.crl CDCl3 {C:\NMR} mdsgrp 14
-114.61
F
CO2Me
Me
O
N
Me
15
f1 (ppm)

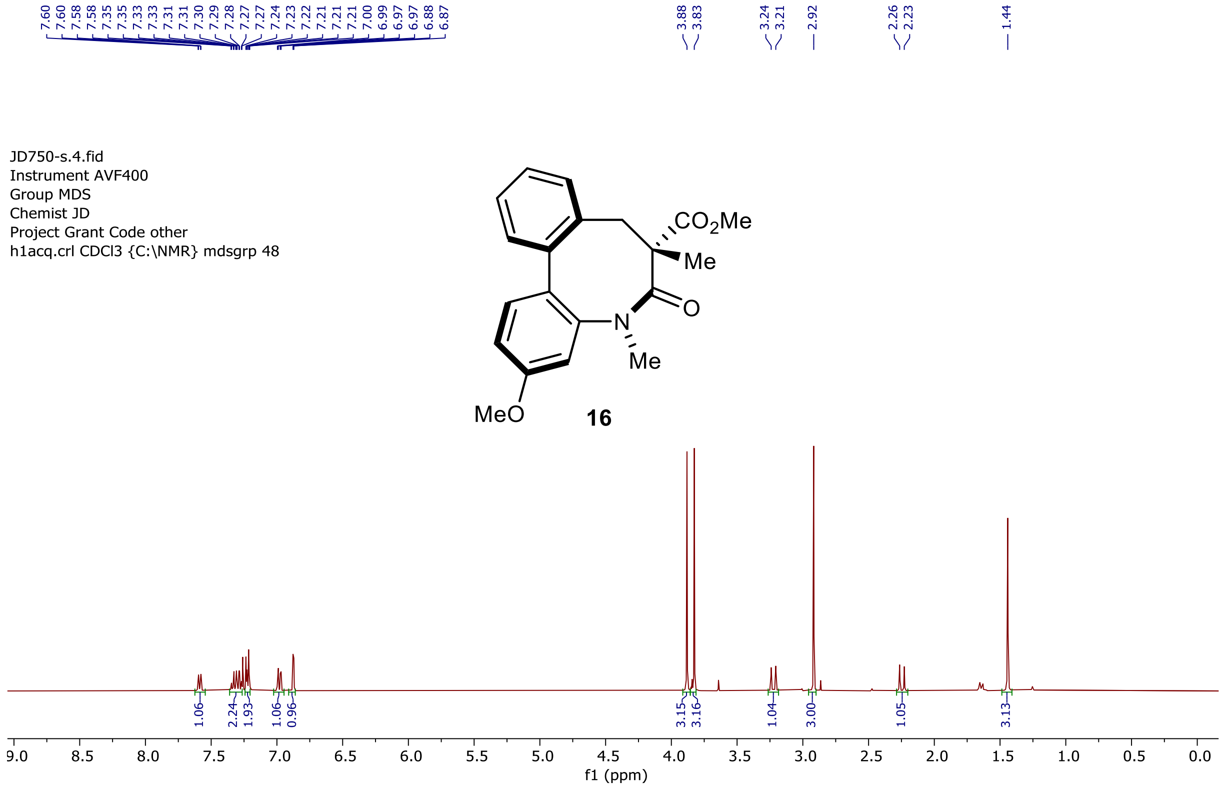
7.60 7.60 7.58 7.58 7.35 7.35 7.33 7.33 7.31 7.31 7.30 7.29 7.28 7.27 7.27 7.24 7.23 7.22 7.21 7.21 7.21 7.00 6.99 6.97 6.97 6.88 6.87
3.88 3.83
3.24 3.21
2.92
2.26 2.23
1.44
JD750-s.4.fid
Instrument AVF400
Group MDS
Chemist JD
Project Grant Code other
h1acq.crl CDCl3 {C:\NMR} mdsgrp 48
CO2Me
Me
O
N
Me
MeO
16
1.06 2.24 1.93 1.06 0.96 3.15 3.16 1.04 3.00 1.05 3.13
f1 (ppm)

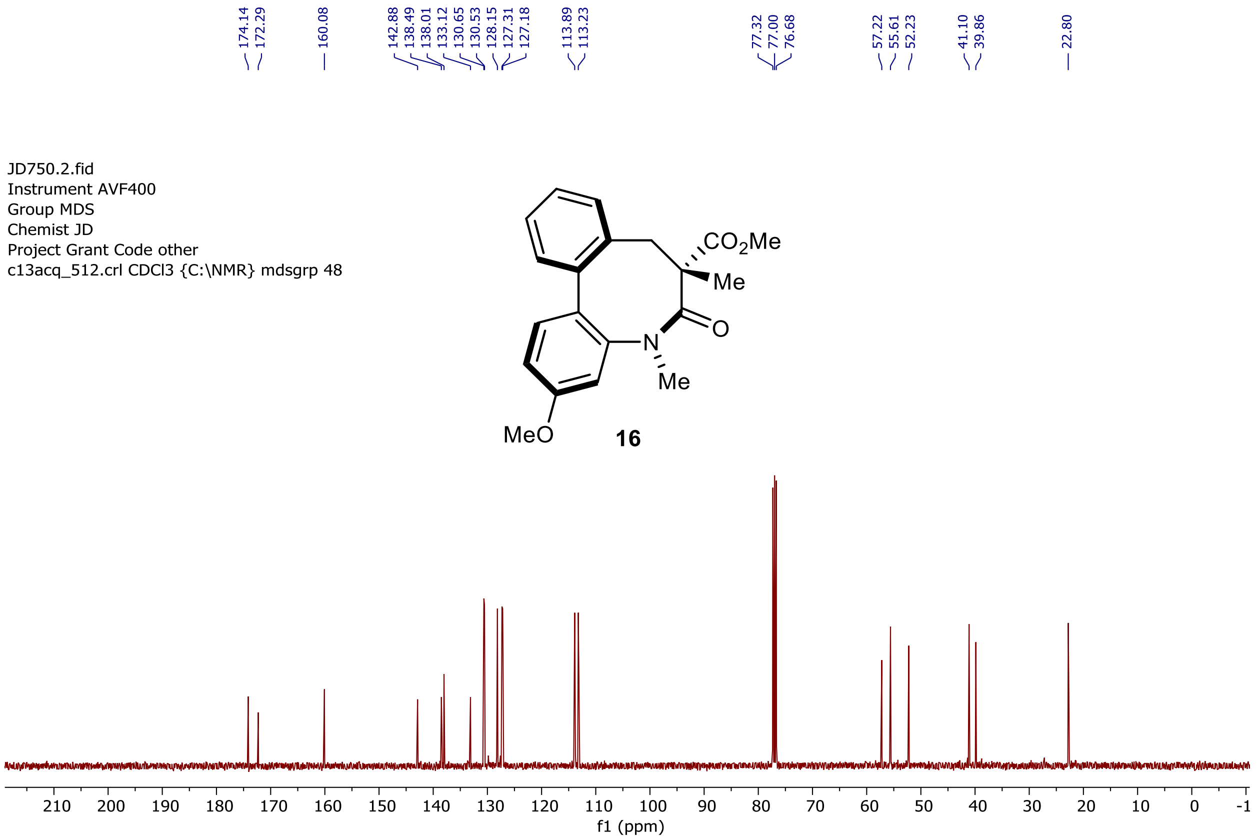
174.14
172.29
160.08
142.88
138.49
138.01
133.12
130.65
130.53
128.15
127.31
127.18
113.89
113.23
77.32
77.00
76.68
57.22
55.61
52.23
41.10
39.86
22.80
JD750.2.fid
Instrument AVF400
Group MDS
Chemist JD
Project Grant Code other
c13acq_512.crl CDCl3 {C:\NMR} mdsgrp 48
CO2Me
Me
O
N
Me
MeO
16
f1 (ppm)

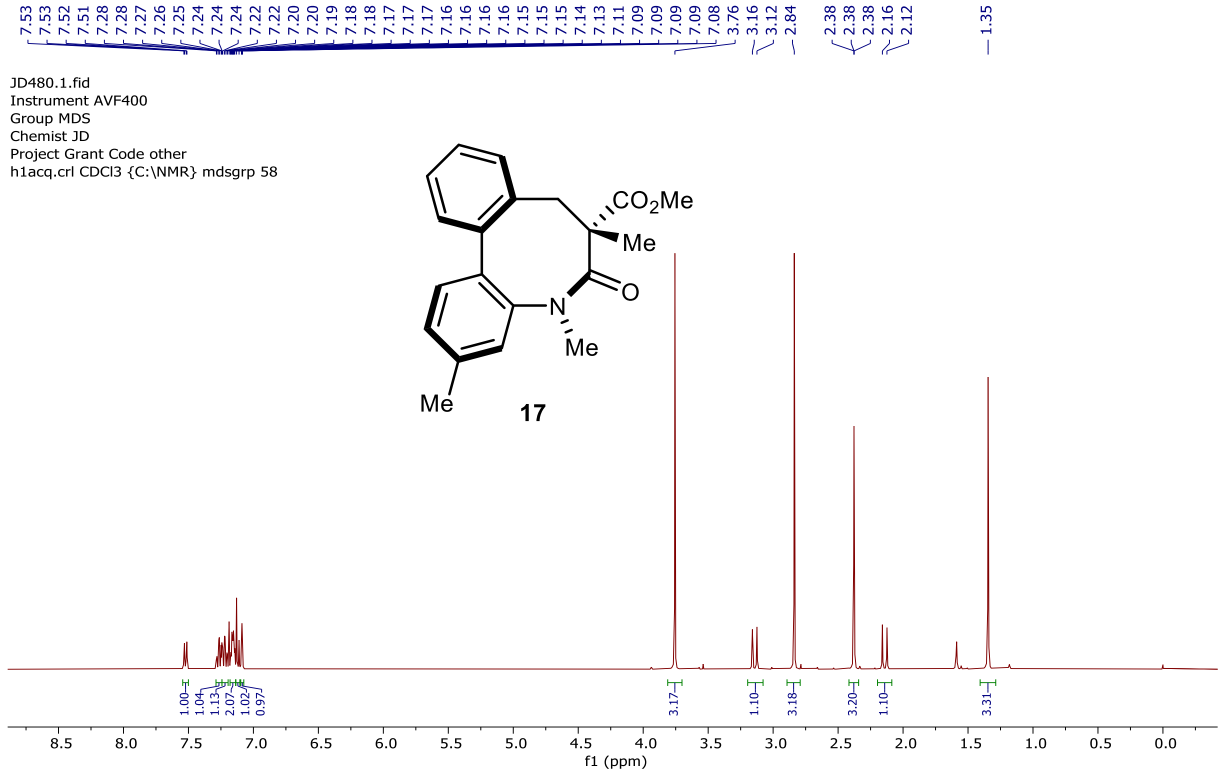
7.53
7.53
7.52
7.51
7.28
7.28
7.27
7.26
7.25
7.24
7.24
7.24
7.22
7.22
7.20
7.20
7.19
7.18
7.18
7.17
7.17
7.17
7.16
7.16
7.16
7.16
7.15
7.15
7.15
7.14
7.13
7.11
7.09
7.09
7.09
7.09
7.08
3.76
3.16
3.12
2.84
2.38
2.38
2.38
2.16
2.12
1.35
JD480.1.fid
Instrument AVF400
Group MDS
Chemist JD
Project Grant Code other
h1acq.crl CDCl3 {C:\NMR} mdsgrp 58
CO2Me
Me
O
N
Me
Me
17
1.00
1.04
1.13
2.07
1.02
0.97
3.17
1.10
3.18
3.20
1.10
3.31
f1 (ppm)

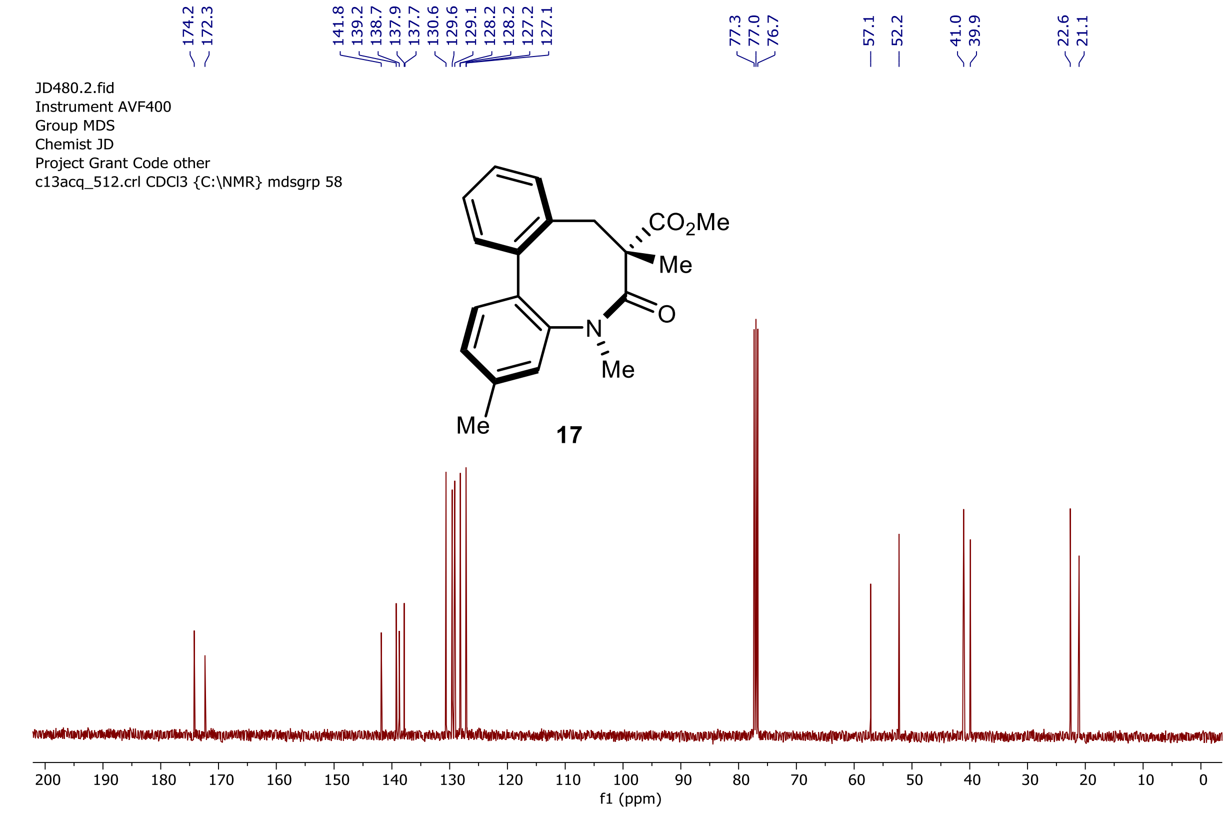
174.2
172.3
141.8
139.2
138.7
137.9
137.7
130.6
129.6
129.1
128.2
128.2
127.2
127.1
77.3
77.0
76.7
57.1
52.2
41.0
39.9
22.6
21.1
JD480.2.fid
Instrument AVF400
Group MDS
Chemist JD
Project Grant Code other
c13acq_512.crl CDCl3 {C:\NMR} mdsgrp 58
CO2Me
Me
O
N
Me
Me
17
200 190 180 170 160 150 140 130 120 110 100 90 80 70 60 50 40 30 20 10 0
f1 (ppm)

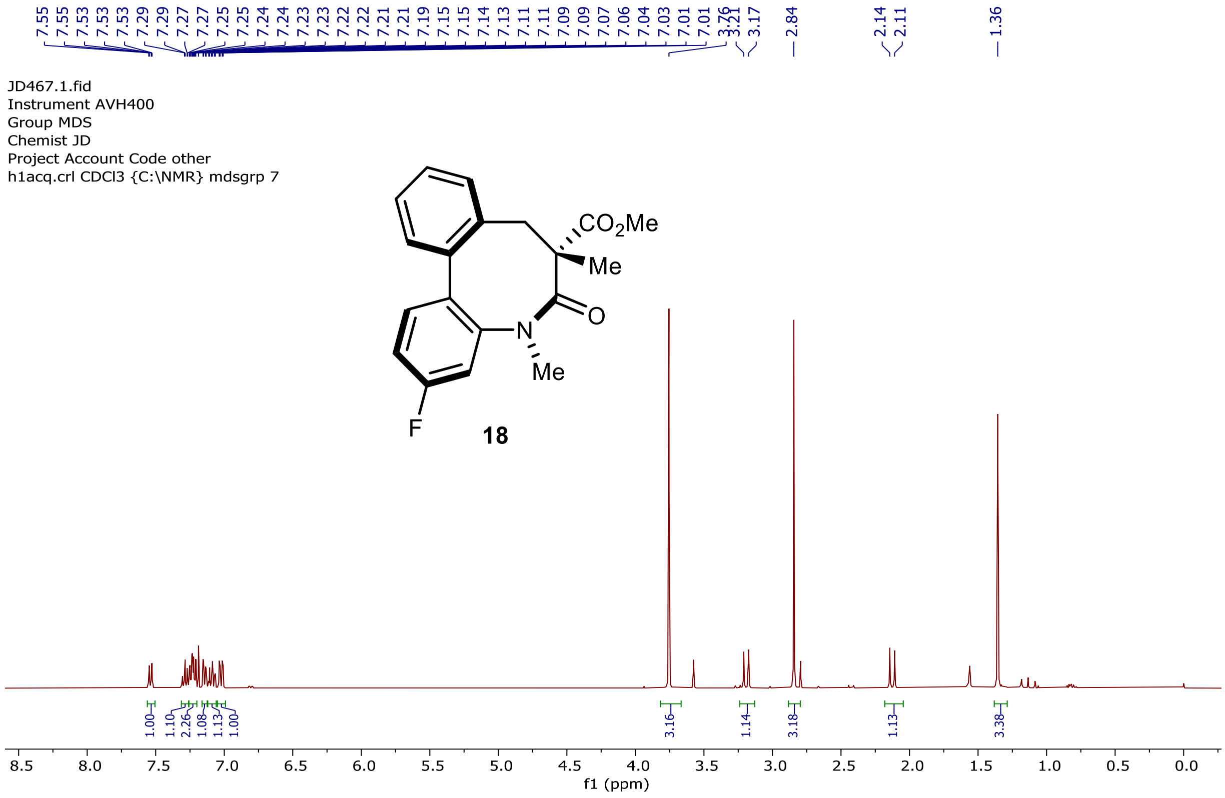
7.55
7.55
7.53
7.53
7.53
7.29
7.29
7.27
7.27
7.25
7.25
7.24
7.24
7.23
7.23
7.22
7.22
7.21
7.21
7.19
7.15
7.15
7.14
7.13
7.11
7.11
7.09
7.09
7.07
7.06
7.04
7.03
7.01
7.01
3.26
3.21
3.17
2.84
2.14
2.11
1.36
JD467.1.fid
Instrument AVH400
Group MDS
Chemist JD
Project Account Code other
h1acq.crl CDCl3 {C:\NMR} mdsgrp 7
CO2Me
Me
O
N
Me
F
18
1.00
1.10
2.26
1.08
1.13
1.00
3.16
1.14
3.18
1.13
3.38
8.5 8.0 7.5 7.0 6.5 6.0 5.5 5.0 4.5 4.0 3.5 3.0 2.5 2.0 1.5 1.0 0.5 0.0
f1 (ppm)

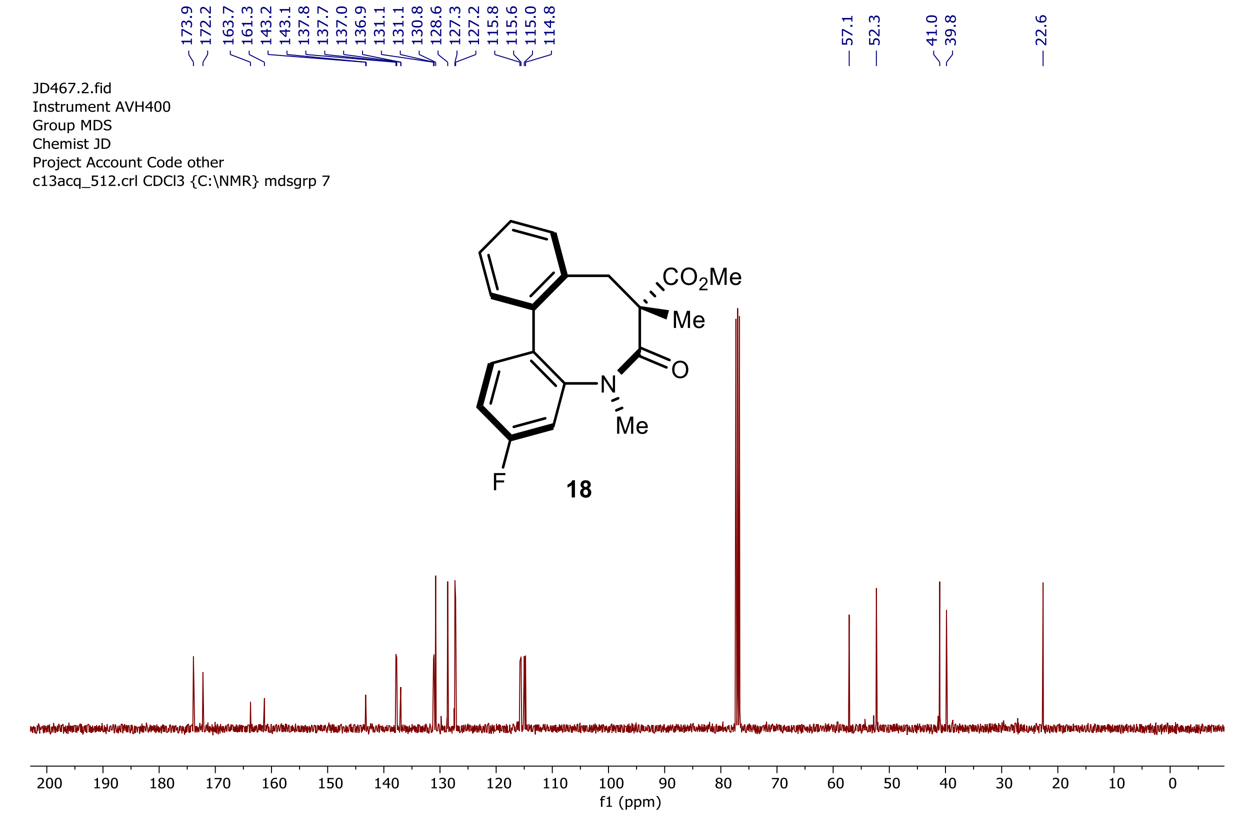
173.9
172.2
163.7
161.3
143.2
143.1
137.8
137.7
137.0
136.9
131.1
131.1
130.8
128.6
127.3
127.2
115.8
115.6
115.0
114.8
57.1
52.3
41.0
39.8
22.6
JD467.2.fid
Instrument AVH400
Group MDS
Chemist JD
Project Account Code other
c13acq_512.crl CDCl3 {C:\NMR} mdsgrp 7
CO2Me
Me
O
N
Me
F
18
f1 (ppm)

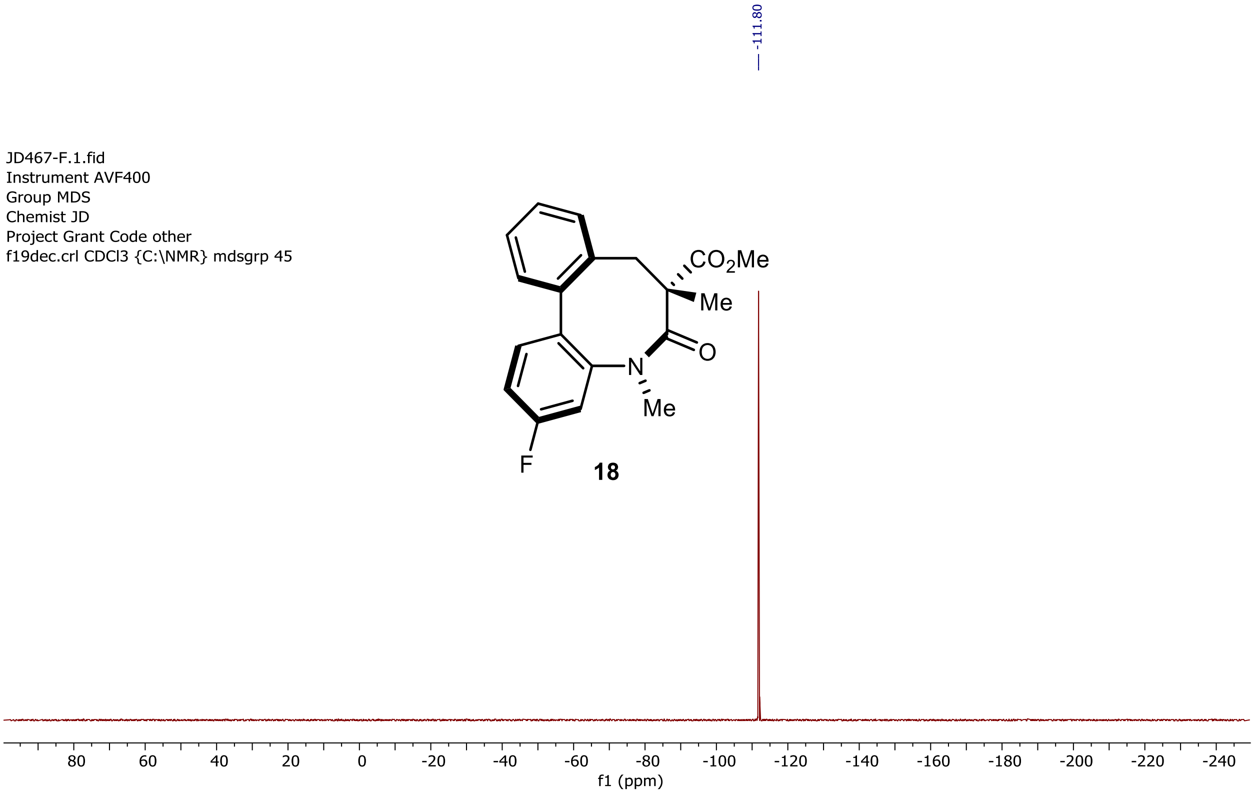
-111.80
JD467-F.1.fid
Instrument AVF400
Group MDS
Chemist JD
Project Grant Code other
f19dec.crl CDCl3 {C:\NMR} mdsgrp 45
CO2Me
Me
O
N
Me
F
18
f1 (ppm)

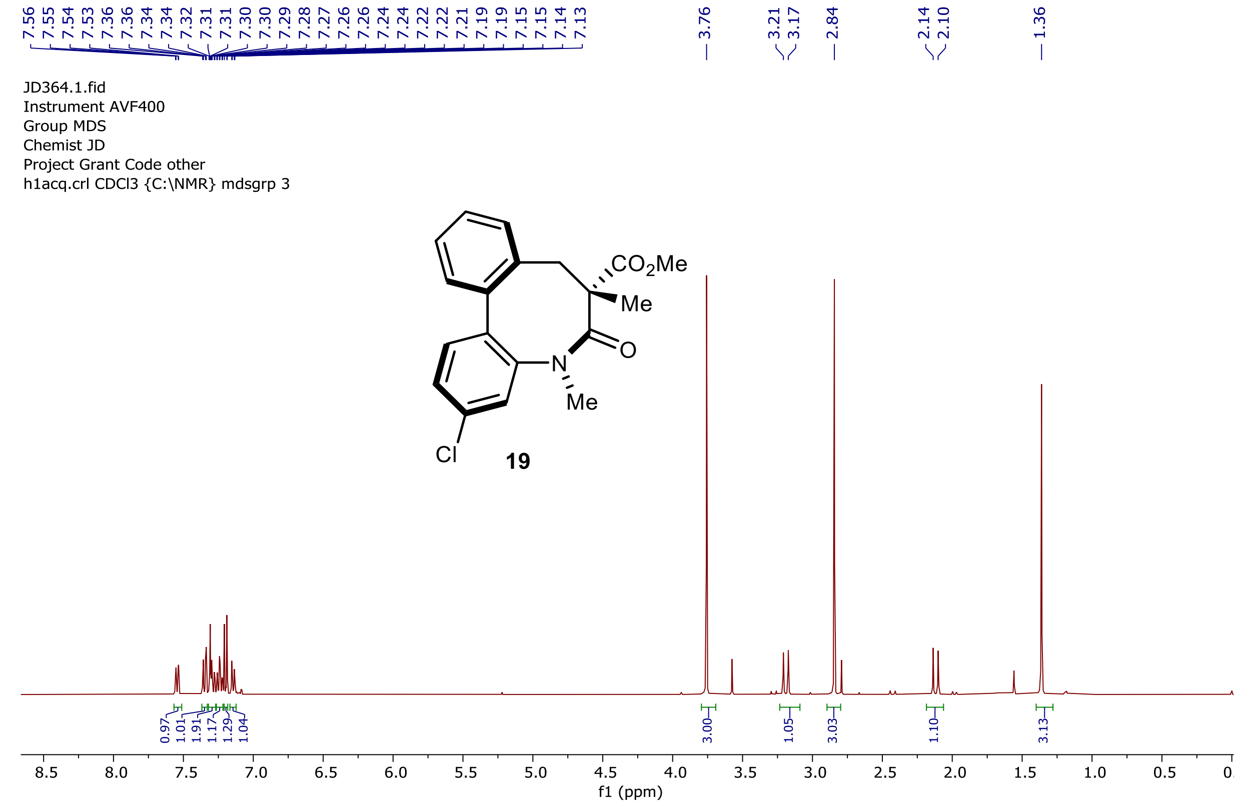
7.56 7.55 7.54 7.53 7.36 7.36 7.34 7.34 7.32 7.31 7.31 7.30 7.29 7.28 7.27 7.26 7.26 7.24 7.24 7.22 7.22 7.21 7.19 7.19 7.15 7.15 7.14 7.13
3.76
3.21 3.17
2.84
2.14 2.10
1.36
JD364.1.fid
Instrument AVF400
Group MDS
Chemist JD
Project Grant Code other
h1acq.crl CDCl3 {C:\NMR} mdsgrp 3
CO2Me
Me
O
N
Me
Cl
19
0.97 1.01 1.91 1.17 1.29 1.04
3.00
1.05
3.03
1.10
3.13
8.5 8.0 7.5 7.0 6.5 6.0 5.5 5.0 4.5 4.0 3.5 3.0 2.5 2.0 1.5 1.0 0.5 0.
f1 (ppm)

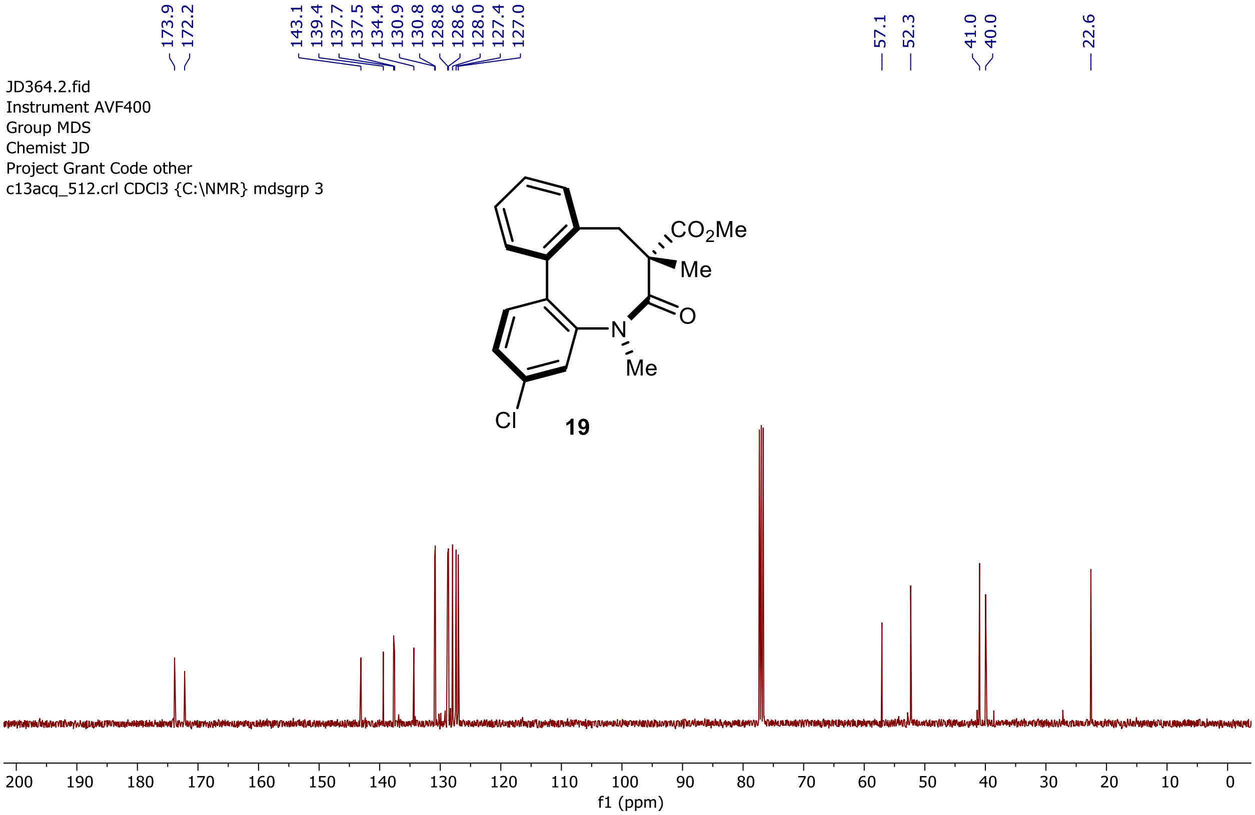
173.9 172.2
143.1 139.4 137.7 137.5 134.4 130.9 130.8 128.8 128.6 128.0 127.4 127.0
57.1 52.3
41.0 40.0
22.6
JD364.2.fid
Instrument AVF400
Group MDS
Chemist JD
Project Grant Code other
c13acq_512.crl CDCl3 {C:\NMR} mdsgrp 3
CO2Me
Me
O
N
Me
Cl
19
200 190 180 170 160 150 140 130 120 110 100 90 80 70 60 50 40 30 20 10 0
f1 (ppm)

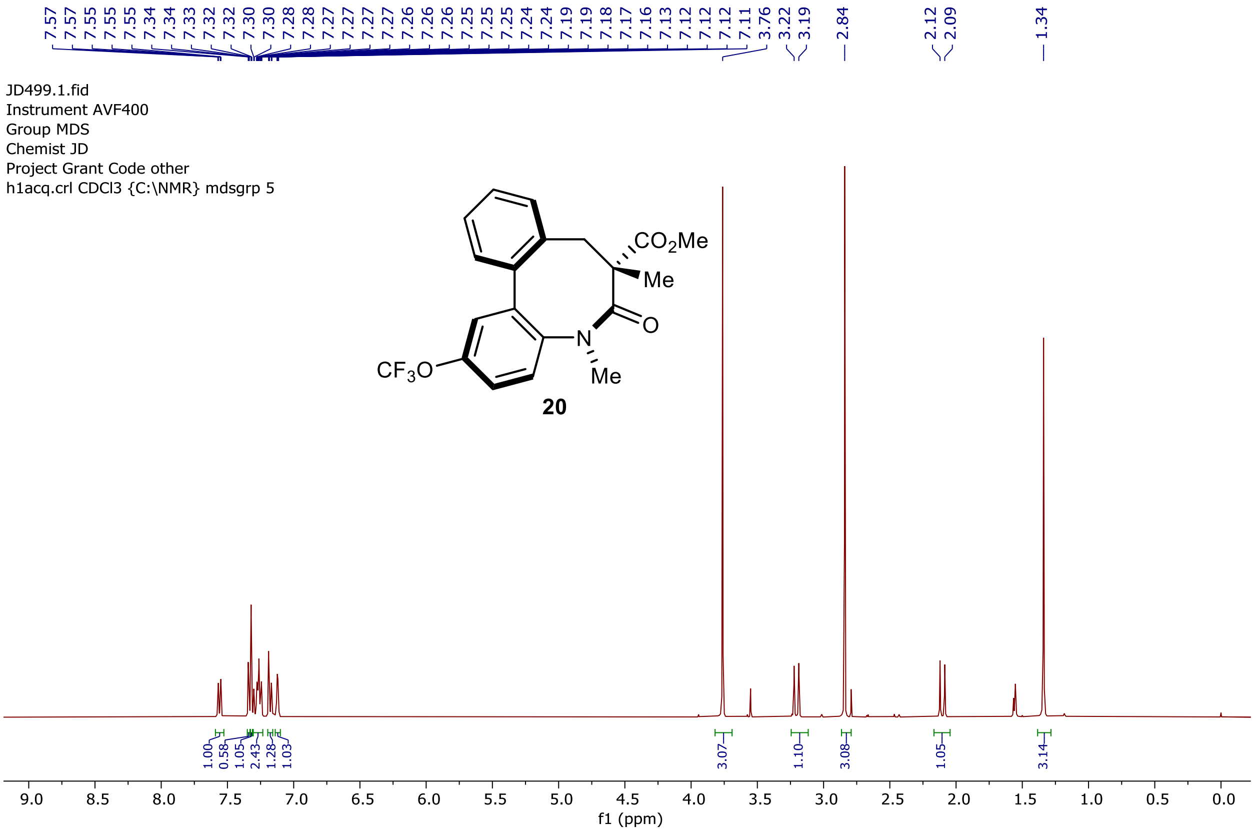
7.57 7.57 7.55 7.55 7.55 7.34 7.34 7.33 7.32 7.32 7.30 7.30 7.28 7.28 7.27 7.27 7.27 7.27 7.26 7.26 7.25 7.25 7.25 7.24 7.24 7.19 7.19 7.18 7.17 7.16 7.13 7.12 7.12 7.12 7.11 3.76 3.22 3.19 2.84 2.12 2.09 1.34
JD499.1.fid
Instrument AVF400
Group MDS
Chemist JD
Project Grant Code other
h1acq.crl CDCl3 {C:\NMR} mdsgrp 5
CO2Me
Me
O
N
Me
CF3O
20
1.00 0.58 1.05 2.43 1.28 1.03 3.07 1.10 3.08 1.05 3.14
9.0 8.5 8.0 7.5 7.0 6.5 6.0 5.5 5.0 4.5 4.0 3.5 3.0 2.5 2.0 1.5 1.0 0.5 0.0
f1 (ppm)

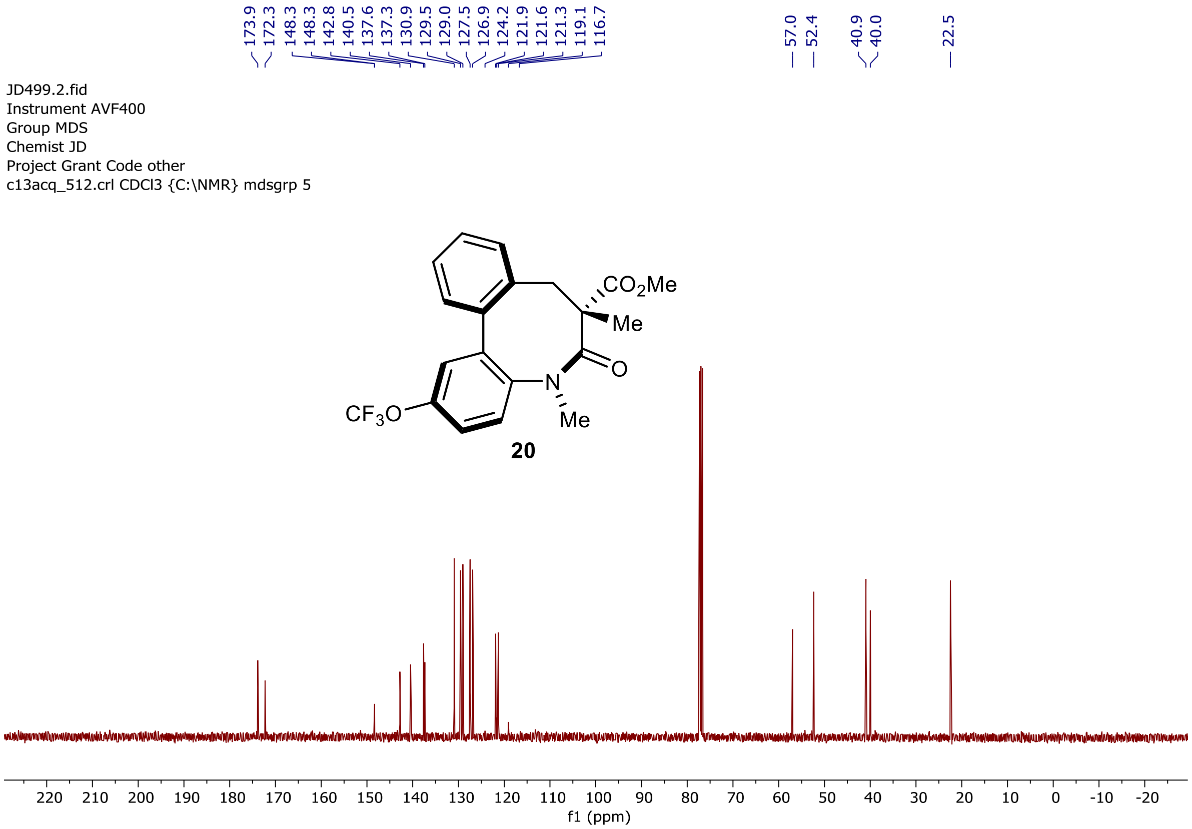
173.9
172.3
148.3
148.3
142.8
140.5
137.6
137.3
130.9
129.5
129.0
127.5
126.9
124.2
121.9
121.6
121.3
119.1
116.7
57.0
52.4
40.9
40.0
22.5
JD499.2.fid
Instrument AVF400
Group MDS
Chemist JD
Project Grant Code other
c13acq_512.crl CDCl3 {C:\NMR} mdsgrp 5
CO2Me
Me
O
N
Me
CF3O
20
220 210 200 190 180 170 160 150 140 130 120 110 100 90 80 70 60 50 40 30 20 10 0 -10 -20
f1 (ppm)

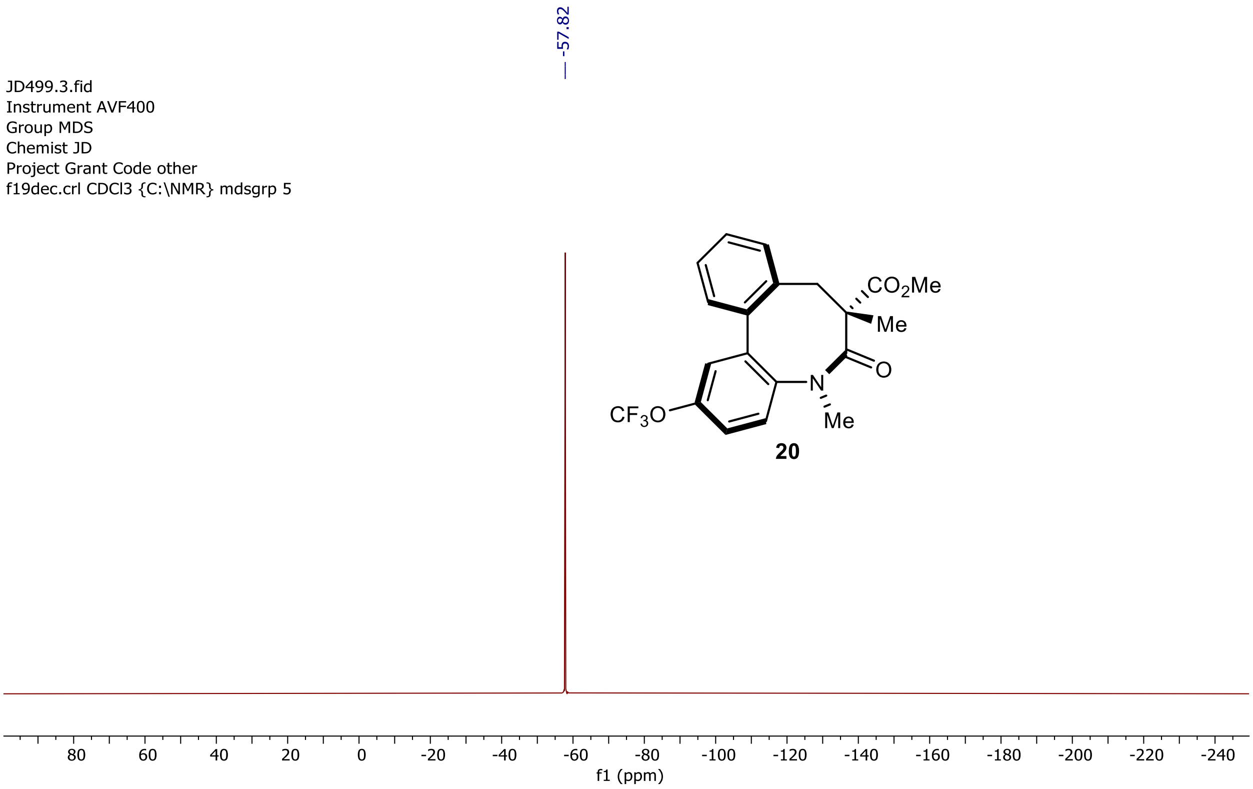
-57.82
JD499.3.fid
Instrument AVF400
Group MDS
Chemist JD
Project Grant Code other
f19dec.crl CDCl3 {C:\NMR} mdsgrp 5
CO2Me
Me
O
N
Me
CF3O
20
80 60 40 20 0 -20 -40 -60 -80 -100 -120 -140 -160 -180 -200 -220 -240
f1 (ppm)

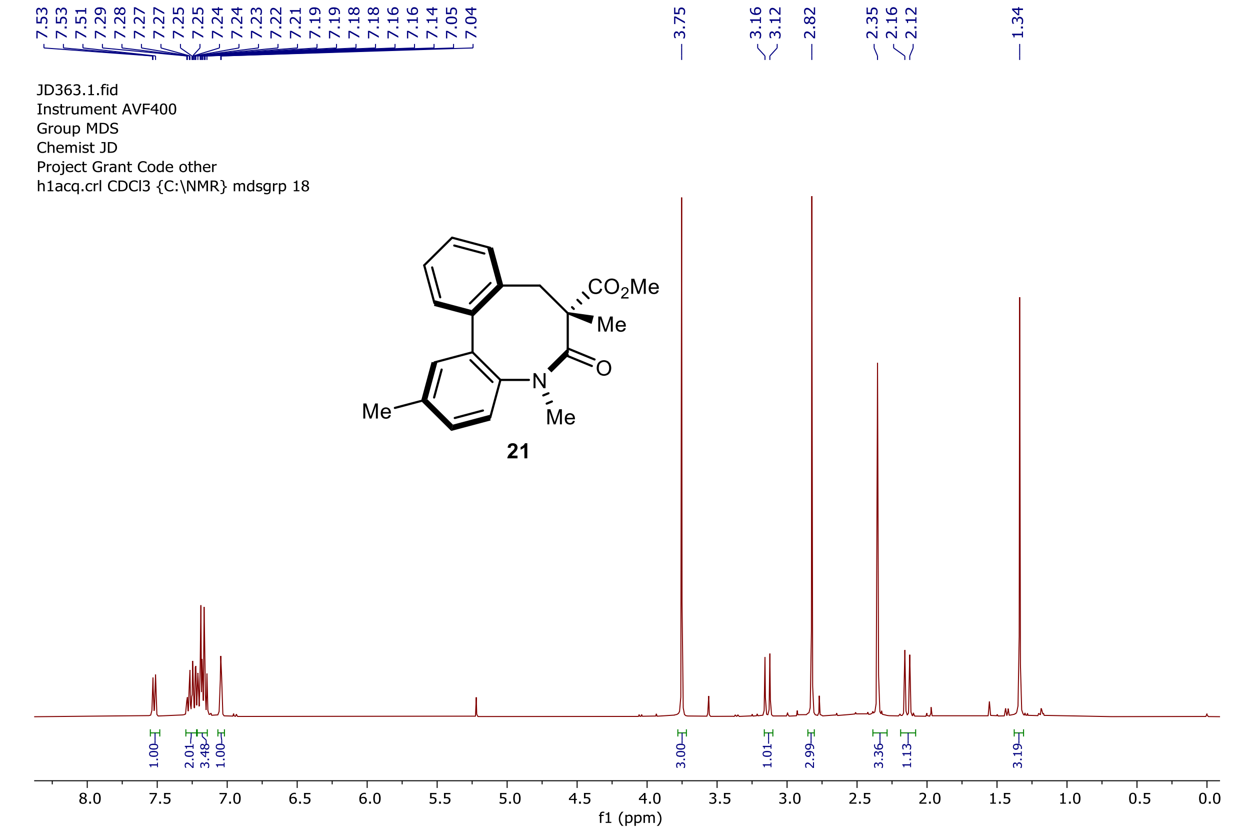
7.53 7.53 7.51 7.29 7.28 7.27 7.27 7.25 7.25 7.24 7.24 7.23 7.22 7.21 7.19 7.19 7.18 7.18 7.16 7.16 7.14 7.05 7.04
3.75
3.16 3.12
2.82
2.35 2.16 2.12
1.34
JD363.1.fid
Instrument AVF400
Group MDS
Chemist JD
Project Grant Code other
h1acq.crl CDCl3 {C:\NMR} mdsgrp 18
CO2Me
Me
O
N
Me
Me
21
1.00 2.01 3.48 1.00 3.00 1.01 2.99 3.36 1.13 3.19
8.0 7.5 7.0 6.5 6.0 5.5 5.0 4.5 4.0 3.5 3.0 2.5 2.0 1.5 1.0 0.5 0.0
f1 (ppm)

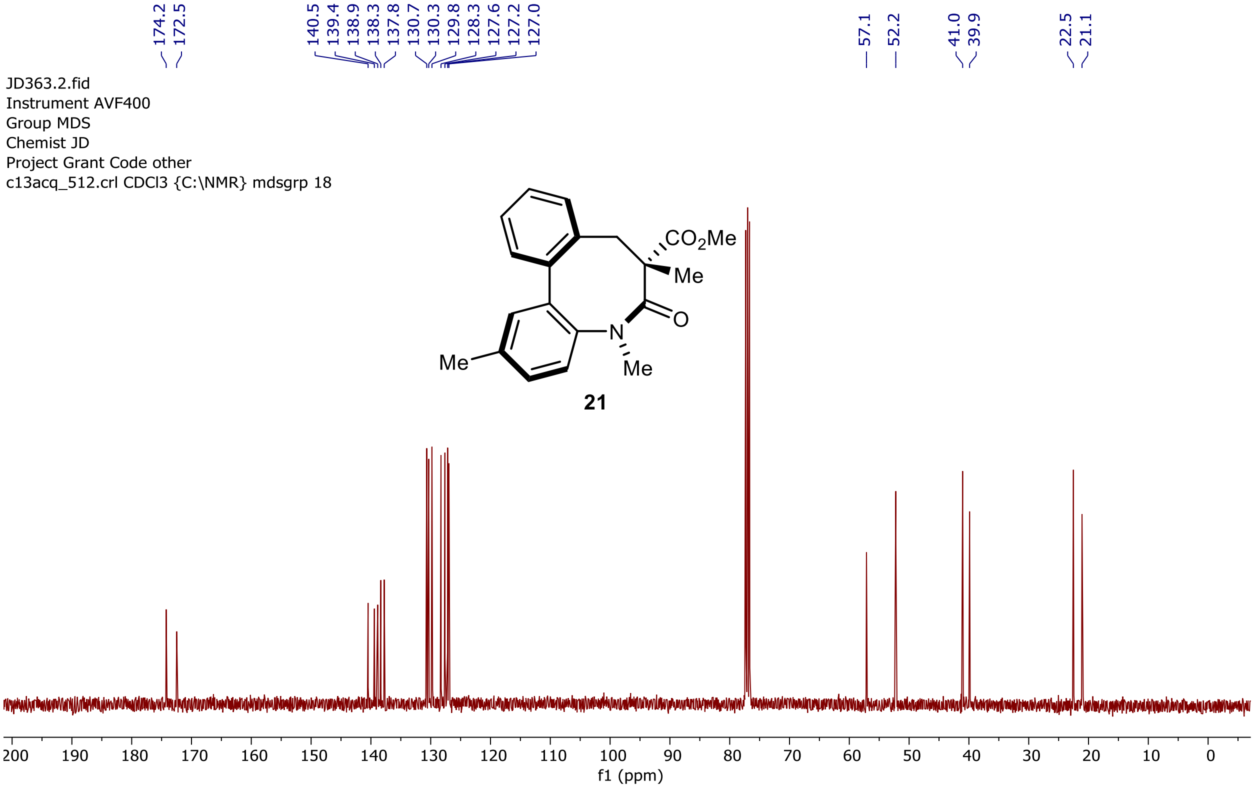
174.2 172.5
140.5 139.4 138.9 138.3 137.8 130.7 130.3 129.8 128.3 127.6 127.2 127.0
57.1
52.2
41.0 39.9
22.5 21.1
JD363.2.fid
Instrument AVF400
Group MDS
Chemist JD
Project Grant Code other
c13acq_512.crl CDCl3 {C:\NMR} mdsgrp 18
CO2Me
Me
O
N
Me
Me
21
200 190 180 170 160 150 140 130 120 110 100 90 80 70 60 50 40 30 20 10 0
f1 (ppm)

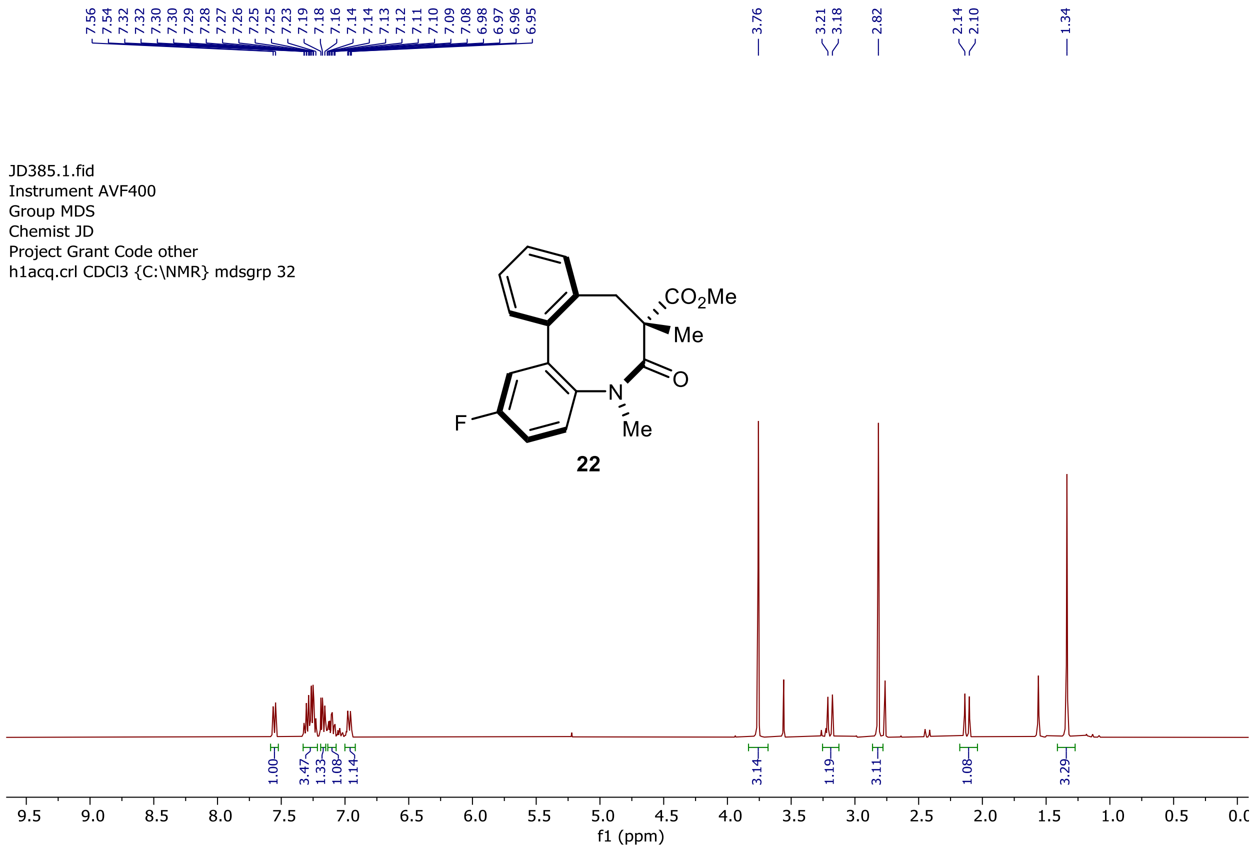
7.56 7.54 7.32 7.32 7.30 7.30 7.29 7.28 7.27 7.26 7.25 7.23 7.19 7.18 7.16 7.14 7.14 7.13 7.12 7.11 7.10 7.09 7.08 6.98 6.97 6.96 6.95
3.76
3.21 3.18
2.82
2.14 2.10
1.34
JD385.1.fid
Instrument AVF400
Group MDS
Chemist JD
Project Grant Code other
h1acq.crl CDCl3 {C:\NMR} mdsgrp 32
CO2Me
Me
O
N
Me
F
22
1.00 3.47 1.33 1.08 1.14 3.14 1.19 3.11 1.08 3.29
9.5 9.0 8.5 8.0 7.5 7.0 6.5 6.0 5.5 5.0 4.5 4.0 3.5 3.0 2.5 2.0 1.5 1.0 0.5
f1 (ppm)

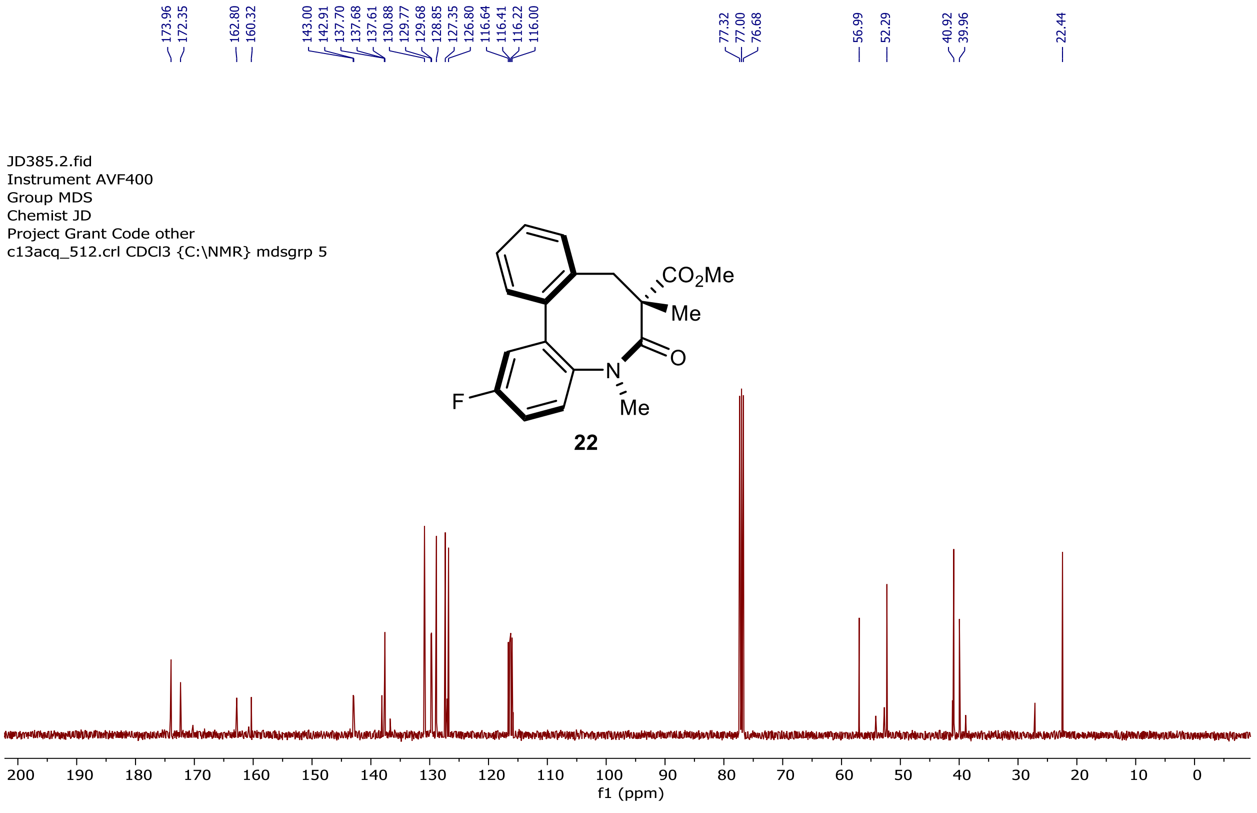
173.96 172.35
162.80 160.32
143.00 142.91 137.70 137.68 137.61 130.88 129.77 129.68 128.85 127.35 126.80 116.64 116.41 116.22 116.00
77.32 77.00 76.68
56.99
52.29
40.92 39.96
22.44
JD385.2.fid
Instrument AVF400
Group MDS
Chemist JD
Project Grant Code other
c13acq_512.crl CDCl3 {C:\NMR} mdsgrp 5
CO2Me
Me
O
N
Me
F
22
200 190 180 170 160 150 140 130 120 110 100 90 80 70 60 50 40 30 20 10 0
f1 (ppm)

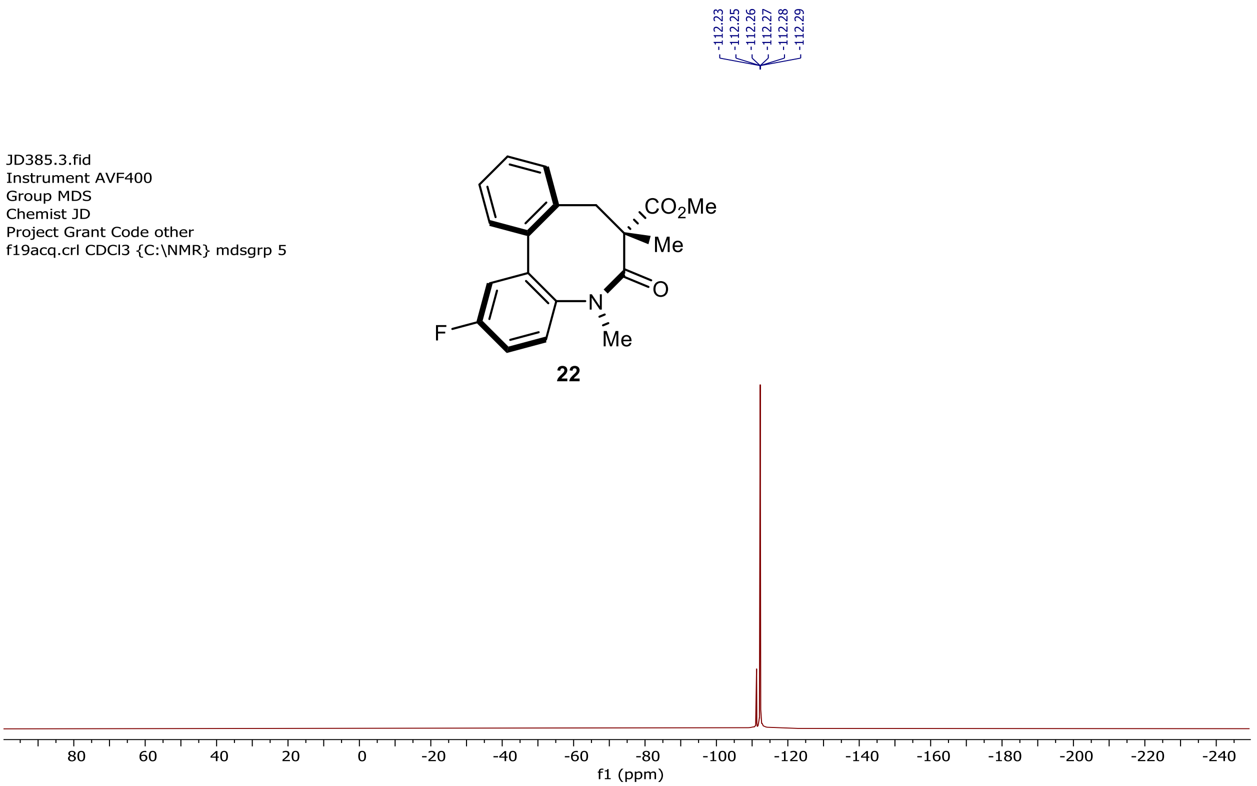
-112.23
-112.25
-112.26
-112.27
-112.28
-112.29
JD385.3.fid
Instrument AVF400
Group MDS
Chemist JD
Project Grant Code other
f19acq.crl CDCl3 {C:\NMR} mdsgrp 5
CO2Me
Me
O
N
Me
F
22
80 60 40 20 0 -20 -40 -60 -80 -100 -120 -140 -160 -180 -200 -220 -240
f1 (ppm)

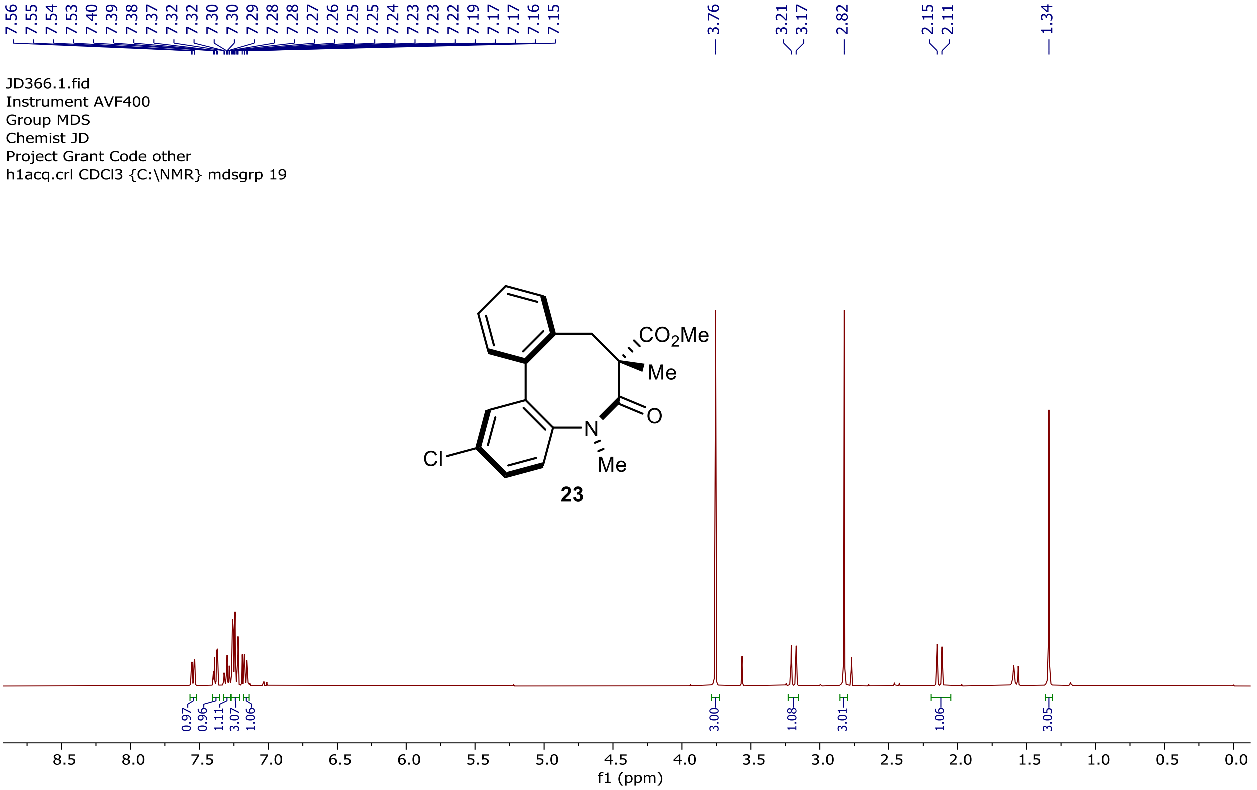
7.56 7.55 7.54 7.53 7.40 7.39 7.38 7.37 7.32 7.32 7.30 7.30 7.29 7.28 7.28 7.27 7.26 7.25 7.25 7.24 7.23 7.23 7.22 7.19 7.17 7.17 7.16 7.15
3.76
3.21
3.17
2.82
2.15
2.11
1.34
JD366.1.fid
Instrument AVF400
Group MDS
Chemist JD
Project Grant Code other
h1acq.crl CDCl3 {C:\NMR} mdsgrp 19
CO2Me
Me
O
N
Me
Cl
23
0.97 0.96 1.11 3.07 1.06
3.00
1.08
3.01
1.06
3.05
8.5 8.0 7.5 7.0 6.5 6.0 5.5 5.0 4.5 4.0 3.5 3.0 2.5 2.0 1.5 1.0 0.5 0.0
f1 (ppm)

JD366.2.fid
Instrument AVF400
Group MDS
Chemist JD
Project Grant Code other
c13acq_512.crl CDCl3 {C:\NMR} mdsgrp 19

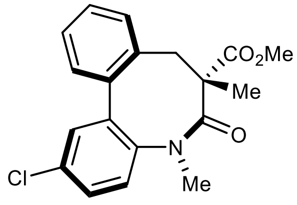

23

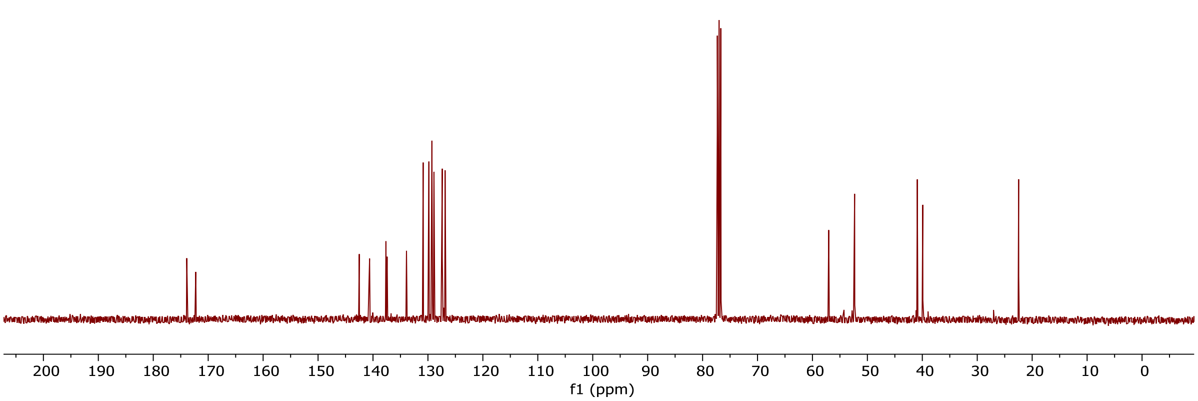

JD751-a.1.fid
Instrument AVF400
Group MDS
Chemist JD
Project Grant Code other
h1acq.crl CDCl3 {C:\NMR} mdsgrp 49

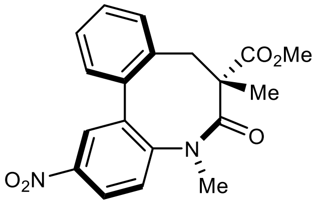

24

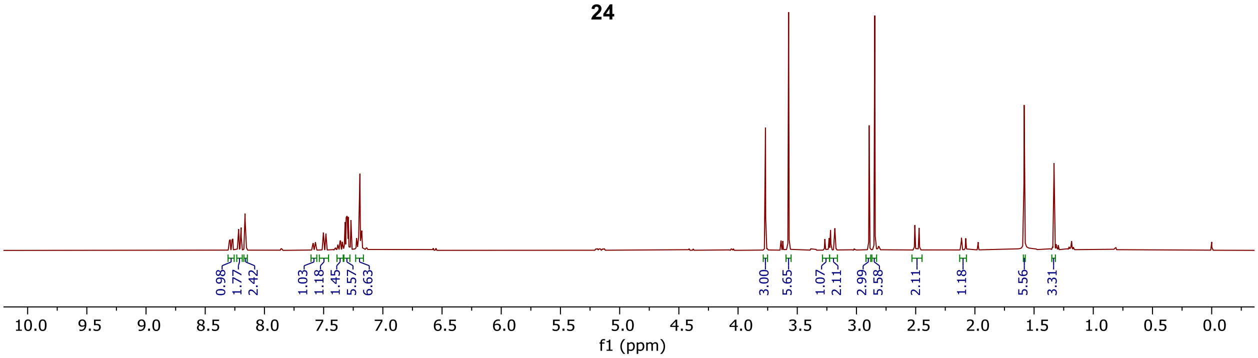

173.74, 173.49, 171.98, 169.63, 147.78, 147.41, 147.24, 146.90, 142.95, 142.49, 137.54, 136.58, 136.51, 136.47, 131.05, 130.01, 129.63, 129.52, 129.11, 129.09, 127.89, 127.74, 127.31, 127.06, 125.22, 124.64, 124.24, 124.07, 77.32, 77.00, 76.68, 57.08, 54.55, 52.97, 52.45, 41.04, 40.90, 40.06, 39.15, 26.82, 22.50

JD751-a.2.fid
Instrument AVF400
Group MDS
Chemist JD
Project Grant Code other
c13acq_512.crl CDCl3 {C:\NMR} mdsgrp 49

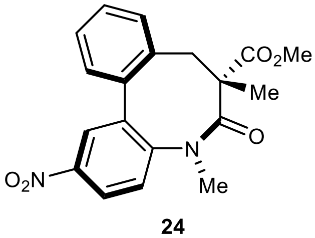

24

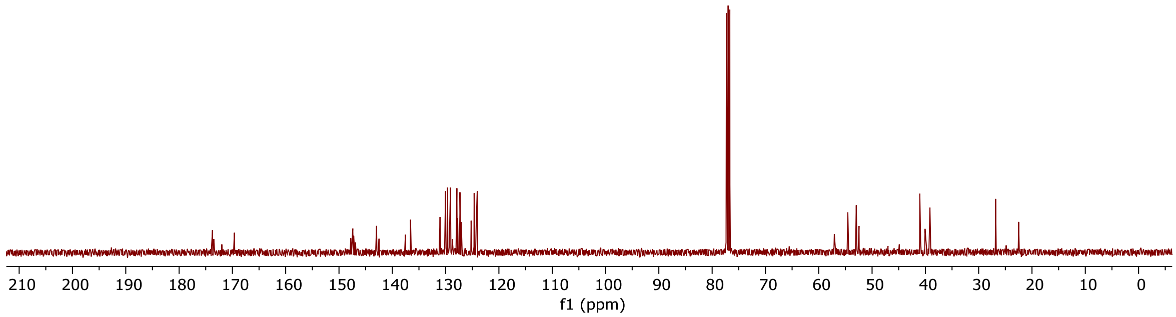

7.74, 7.73, 7.72, 7.71, 7.59, 7.59, 7.58, 7.58, 7.57, 7.56, 7.55, 7.44, 7.42, 7.36, 7.36, 7.34, 7.34, 7.32, 7.32, 7.28, 7.28, 7.28, 7.28, 7.26, 7.26, 7.17, 7.17, 7.15, 7.15, 3.76, 3.25, 3.21, 2.87, 2.09, 2.05, 1.32

JD469.1.fid
Instrument AVF400
Group MDS
Chemist JD
Project Grant Code other
h1acq.crl CDCl3 {C:\NMR} mdsgrp 58

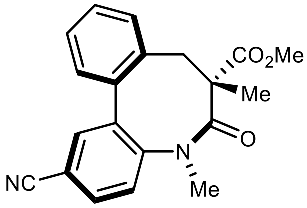

25

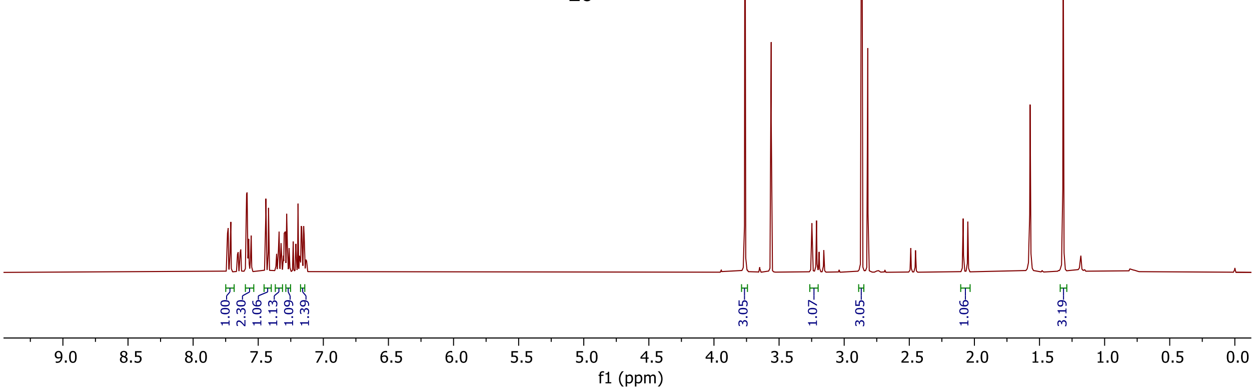

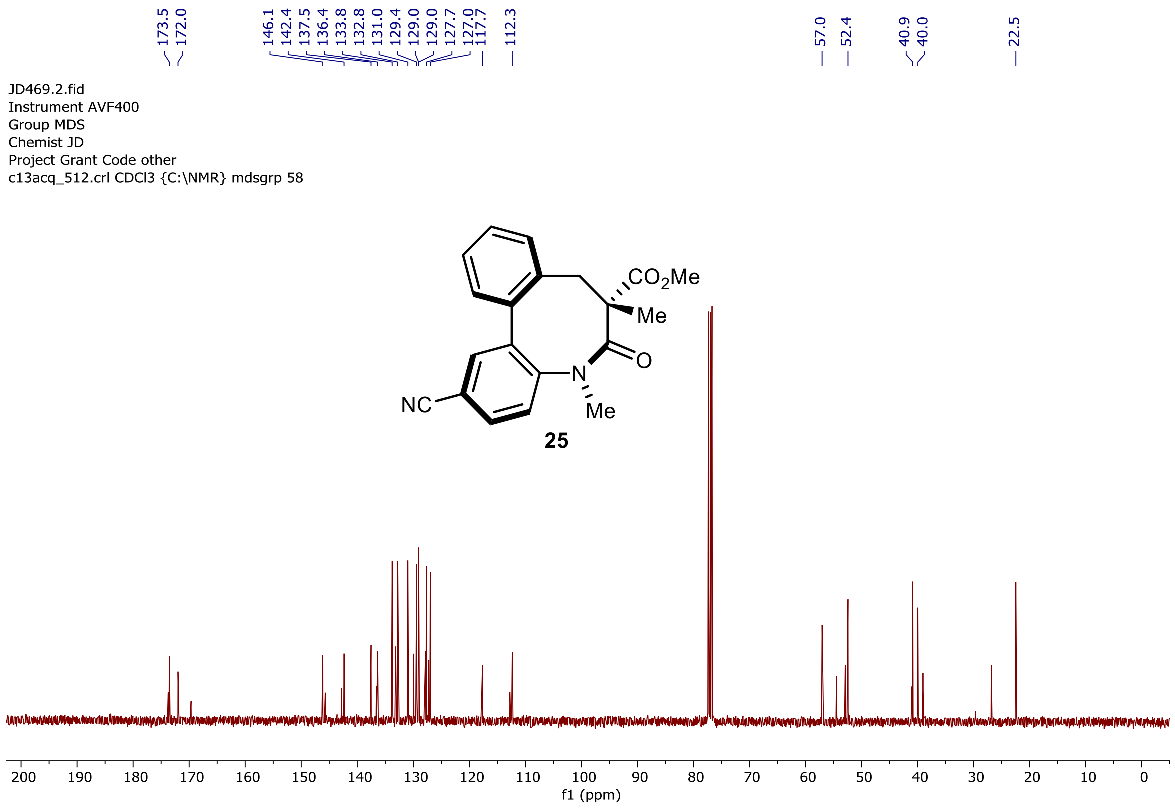
173.5
172.0
146.1
142.4
137.5
136.4
133.8
132.8
131.0
129.4
129.0
129.0
127.7
127.0
117.7
112.3
57.0
52.4
40.9
40.0
22.5
JD469.2.fid
Instrument AVF400
Group MDS
Chemist JD
Project Grant Code other
c13acq_512.crl CDCl3 {C:\NMR} mdsgrp 58
CO2Me
Me
O
N
Me
NC
25
200 190 180 170 160 150 140 130 120 110 100 90 80 70 60 50 40 30 20 10 0
f1 (ppm)

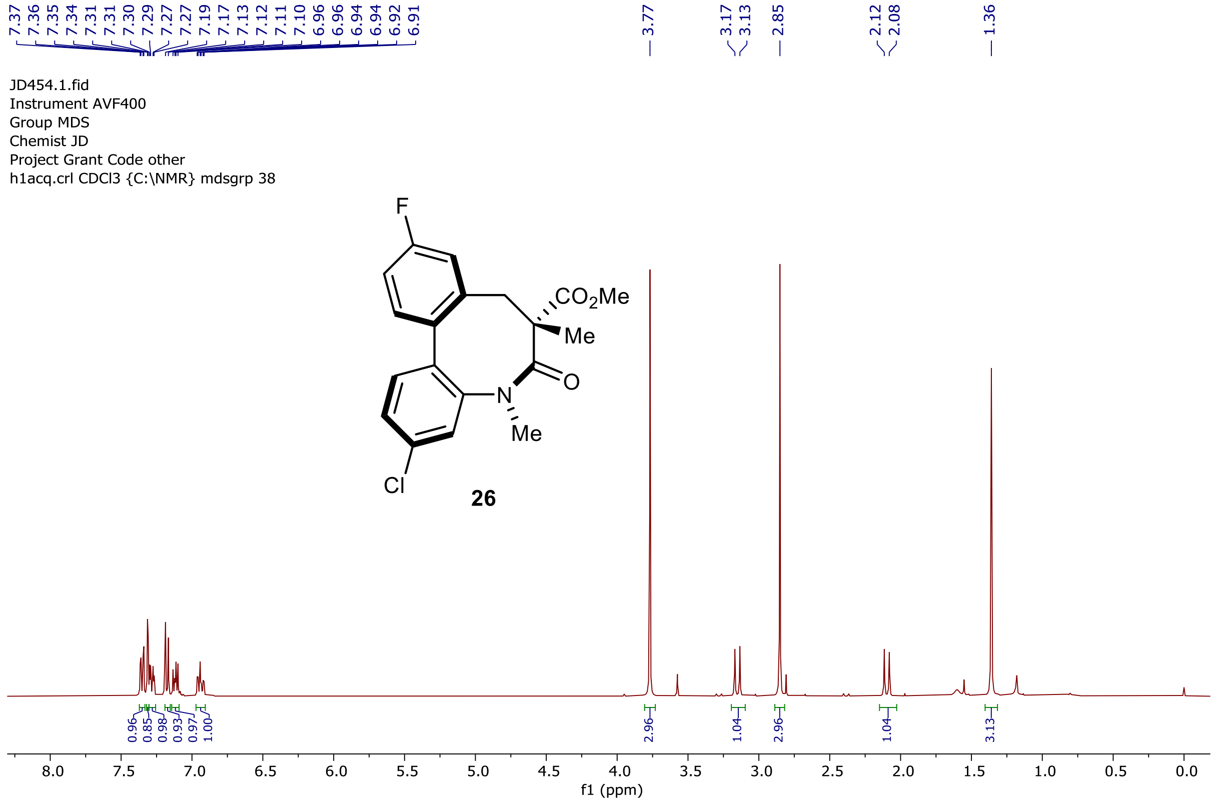
7.37
7.36
7.35
7.34
7.31
7.31
7.30
7.29
7.27
7.27
7.19
7.17
7.13
7.12
7.11
7.10
6.96
6.96
6.94
6.94
6.92
6.91
3.77
3.17
3.13
2.85
2.12
2.08
1.36
JD454.1.fid
Instrument AVF400
Group MDS
Chemist JD
Project Grant Code other
h1acq.crl CDCl3 {C:\NMR} mdsgrp 38
F
CO2Me
Me
O
N
Me
Cl
26
0.96
0.85
0.98
0.93
0.97
1.00
2.96
1.04
2.96
1.04
3.13
8.0 7.5 7.0 6.5 6.0 5.5 5.0 4.5 4.0 3.5 3.0 2.5 2.0 1.5 1.0 0.5 0.0
f1 (ppm)

JD454.2.fid
Instrument AVF400
Group MDS
Chemist JD
Project Grant Code other
c13acq_512.crl CDCl3 {C:\NMR} mdsgrp 38

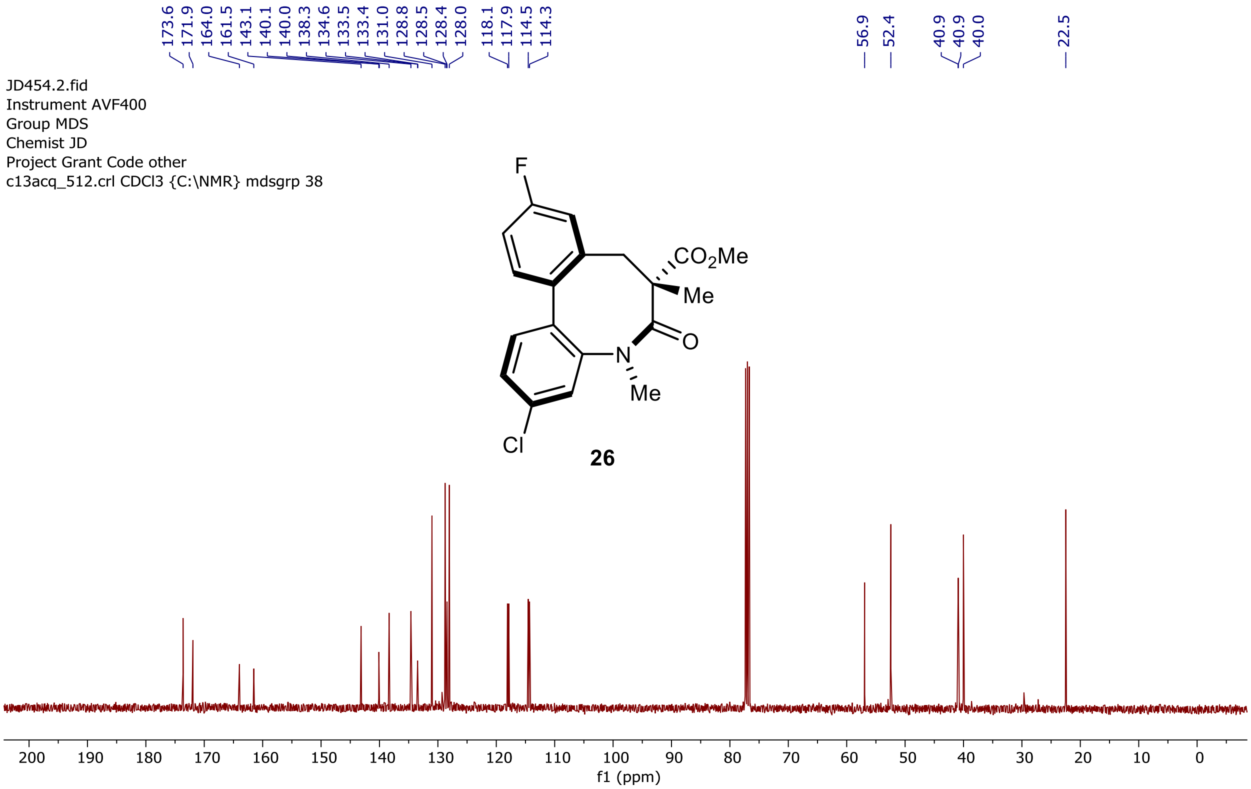

JD454.3.fid
Instrument AVF400
Group MDS
Chemist JD
Project Grant Code other
f19dec.crl CDCl3 {C:\NMR} mdsgrp 38

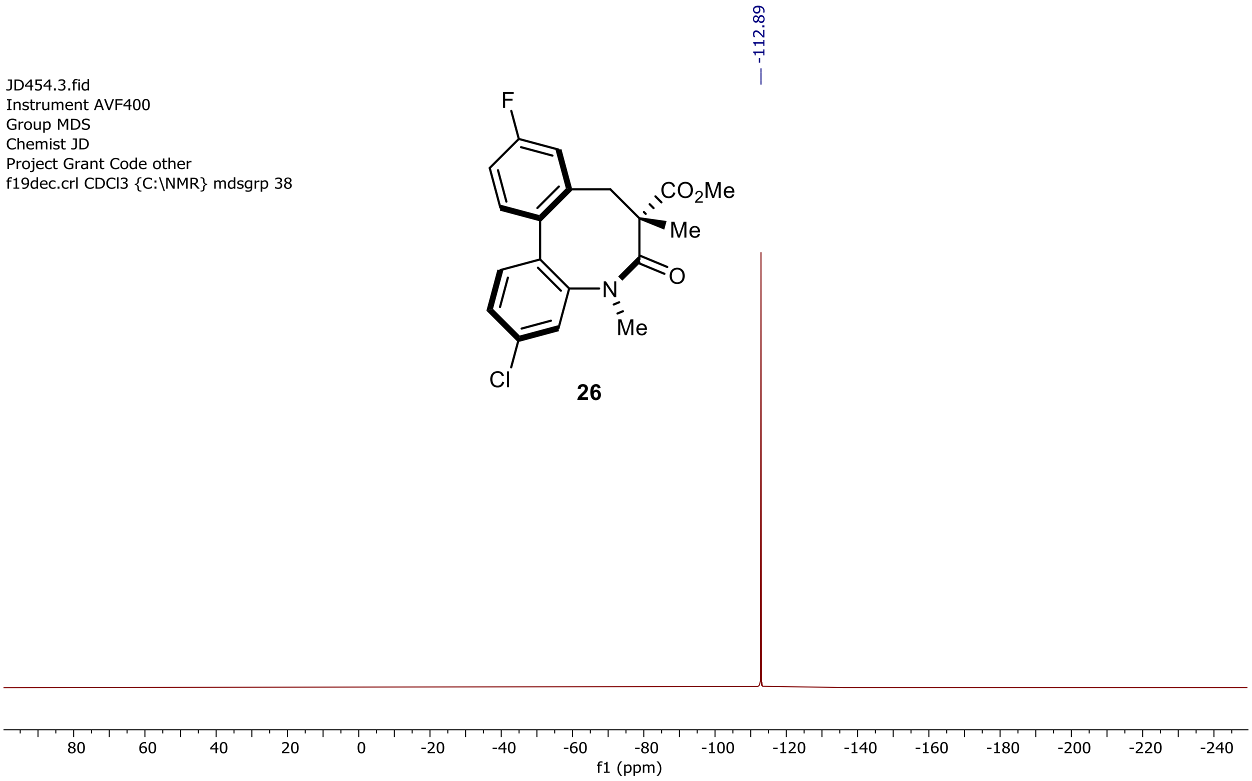

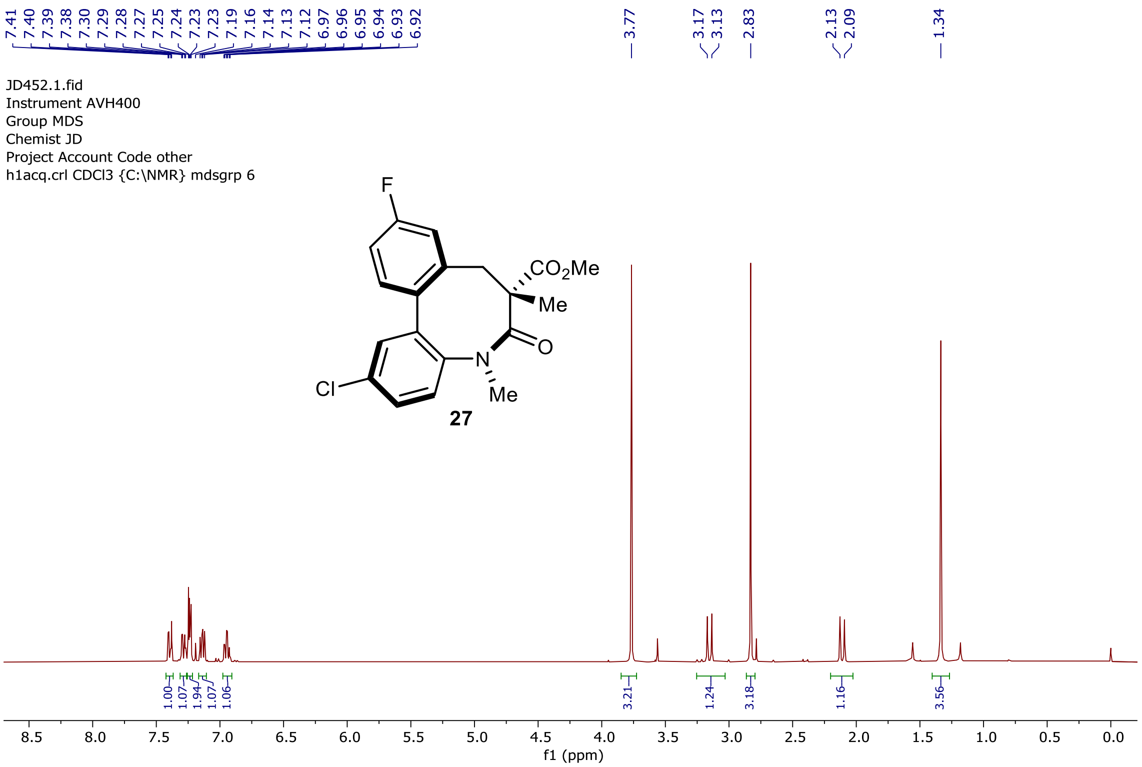
7.41 7.40 7.39 7.38 7.30 7.29 7.28 7.27 7.25 7.24 7.23 7.23 7.19 7.16 7.14 7.13 7.12 6.97 6.96 6.95 6.94 6.93 6.92
3.77
3.17 3.13
2.83
2.13 2.09
1.34
JD452.1.fid
Instrument AVH400
Group MDS
Chemist JD
Project Account Code other
h1acq.crl CDCl3 {C:\NMR} mdsgrp 6
F
CO2Me
Me
O
N
Me
Cl
27
1.00 1.07 1.94 1.07 1.06
3.21
1.24
3.18
1.16
3.56
8.5 8.0 7.5 7.0 6.5 6.0 5.5 5.0 4.5 4.0 3.5 3.0 2.5 2.0 1.5 1.0 0.5 0.0
f1 (ppm)

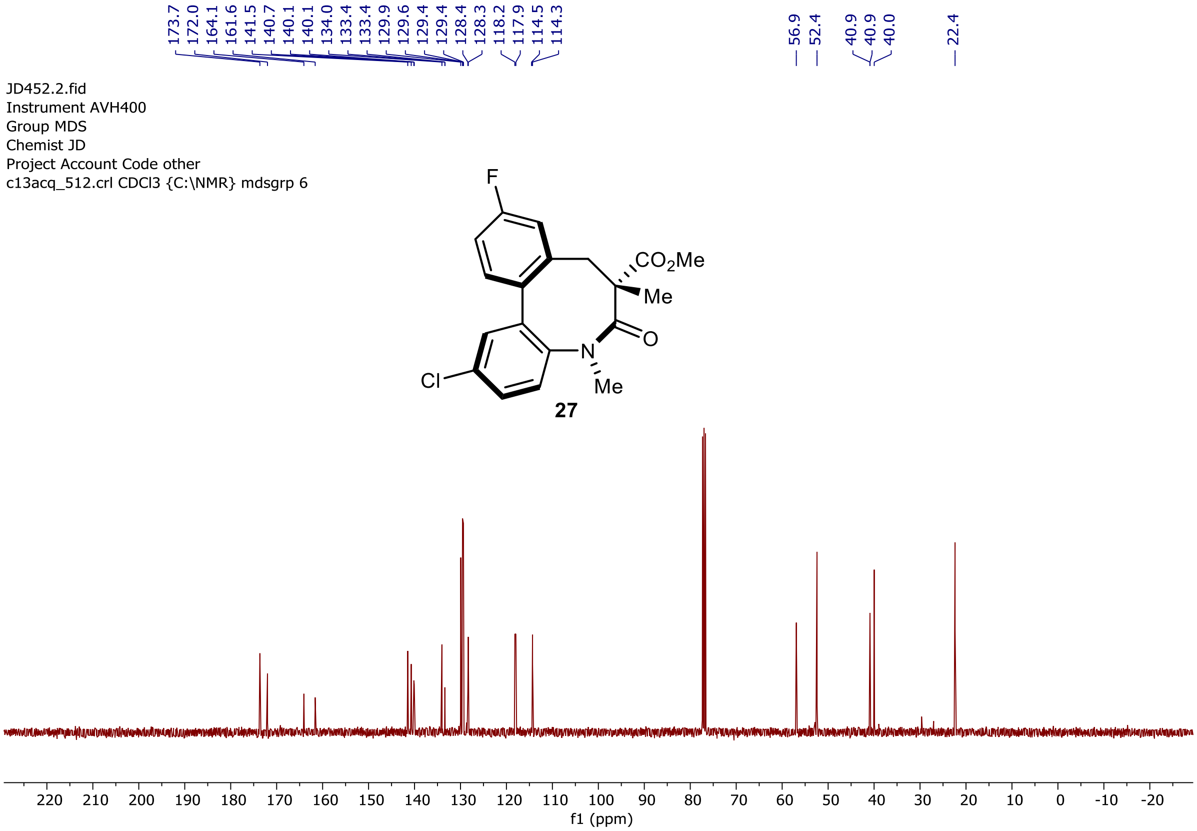
173.7 172.0 164.1 161.6 141.5 140.7 140.1 140.1 134.0 133.4 133.4 129.9 129.6 129.4 129.4 128.4 128.3 118.2 117.9 114.5 114.3
56.9 52.4
40.9 40.9 40.0
22.4
JD452.2.fid
Instrument AVH400
Group MDS
Chemist JD
Project Account Code other
c13acq_512.crl CDCl3 {C:\NMR} mdsgrp 6
F
CO2Me
Me
O
N
Me
Cl
27
220 210 200 190 180 170 160 150 140 130 120 110 100 90 80 70 60 50 40 30 20 10 0 -10 -20
f1 (ppm)

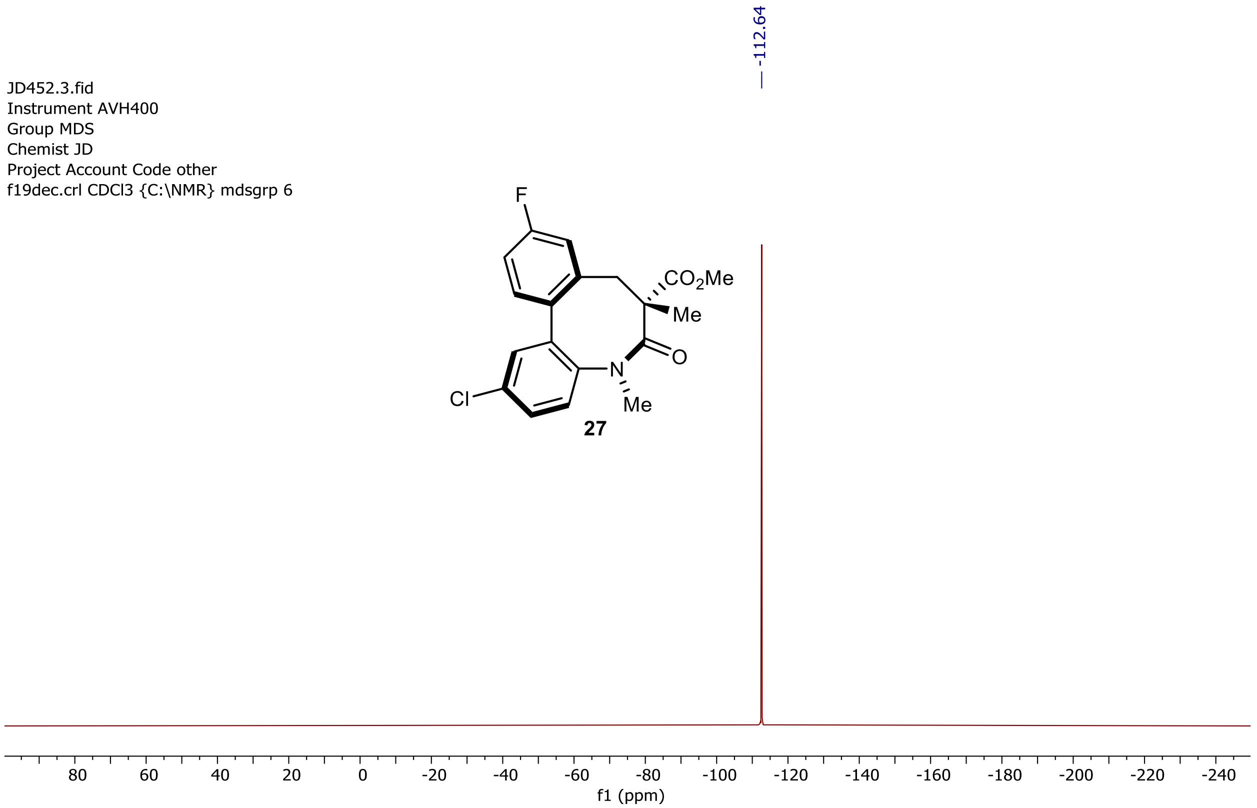
-112.64
JD452.3.fid
Instrument AVH400
Group MDS
Chemist JD
Project Account Code other
f19dec.crl CDCl3 {C:\NMR} mdsgrp 6
F
CO_2Me
Me
O
N
Me
Cl
27
80 60 40 20 0 -20 -40 -60 -80 -100 -120 -140 -160 -180 -200 -220 -240
f1 (ppm)

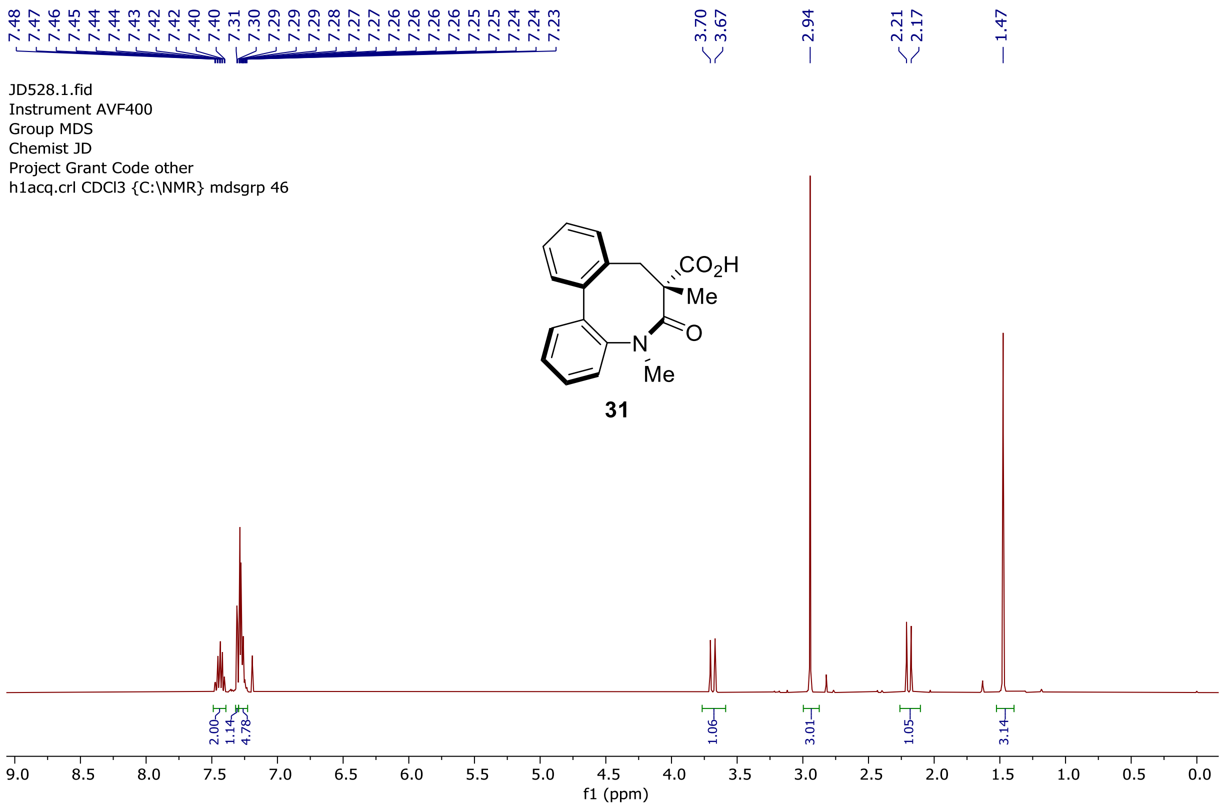
7.48 7.47 7.46 7.45 7.44 7.44 7.43 7.42 7.42 7.40 7.40 7.31 7.30 7.29 7.29 7.29 7.28 7.27 7.27 7.26 7.26 7.26 7.25 7.25 7.24 7.24 7.23
3.70 3.67
2.94
2.21 2.17
1.47
JD528.1.fid
Instrument AVF400
Group MDS
Chemist JD
Project Grant Code other
h1acq.crl CDCl3 {C:\NMR} mdsgrp 46
CO_2H
Me
O
N
Me
31
2.00 1.14 4.78
1.06
3.01
1.05
3.14
9.0 8.5 8.0 7.5 7.0 6.5 6.0 5.5 5.0 4.5 4.0 3.5 3.0 2.5 2.0 1.5 1.0 0.5 0.0
f1 (ppm)

JD528.2.fid
Instrument AVF400
Group MDS
Chemist JD
Project Grant Code other
c13acq_512.crl CDCl3 {C:\NMR} mdsgrp 46

31

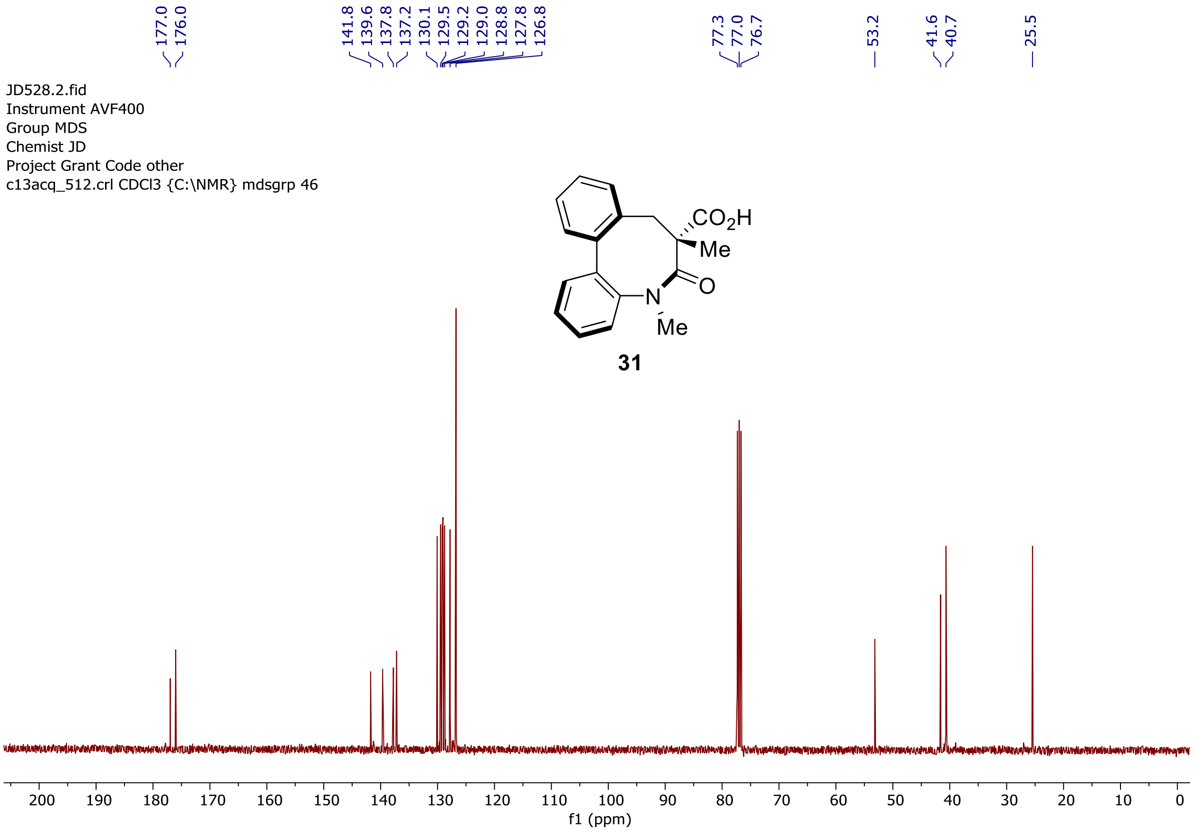

JD527.1.fid
Instrument AVF400
Group MDS
Chemist JD
Project Grant Code other
h1acq.crl CDCl3 {C:\NMR} mdsgrp 27

32

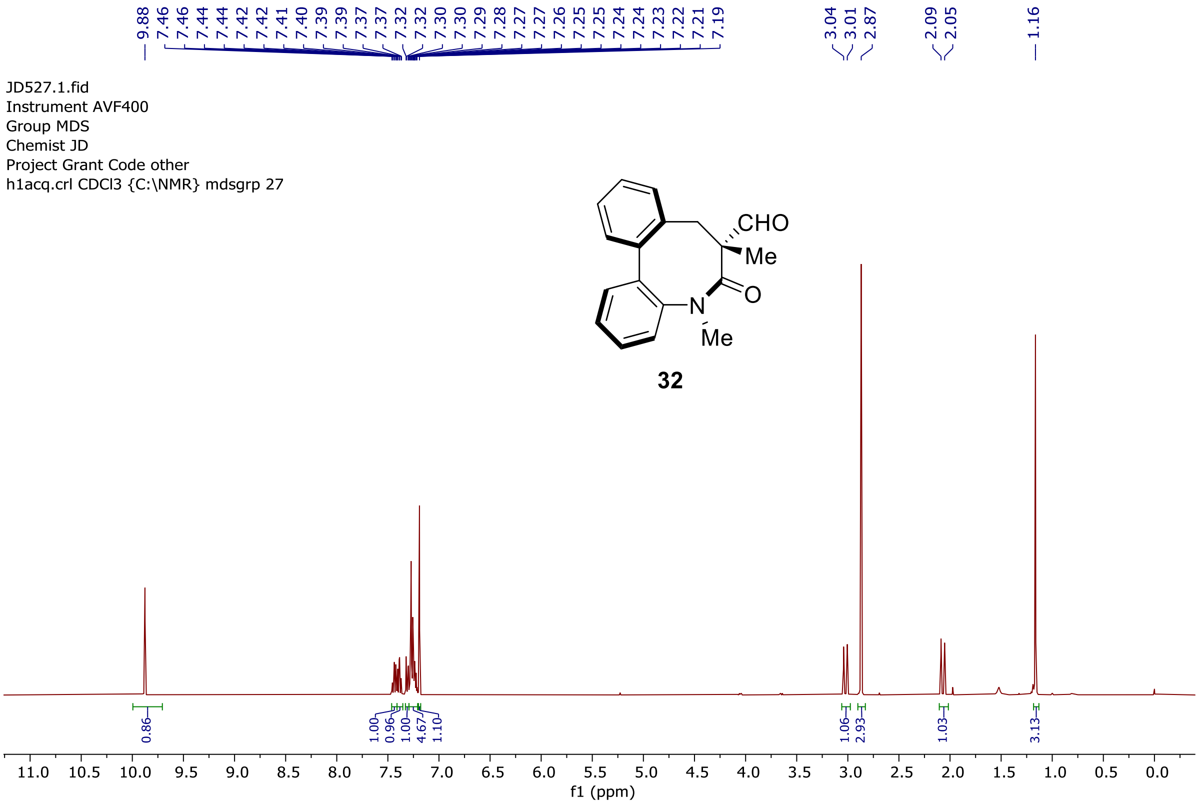

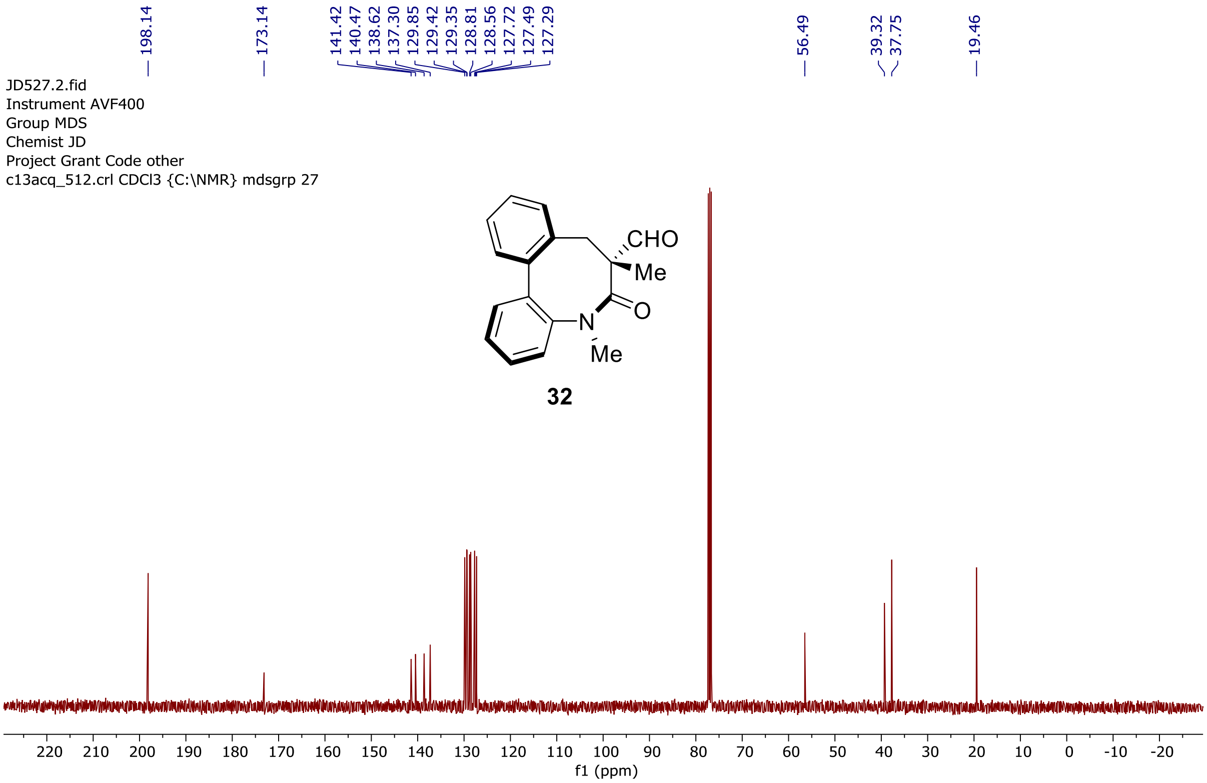
198.14
173.14
141.42
140.47
138.62
137.30
129.85
129.42
129.35
128.81
128.56
127.72
127.49
127.29
56.49
39.32
37.75
19.46
JD527.2.fid
Instrument AVF400
Group MDS
Chemist JD
Project Grant Code other
c13acq_512.crl CDCl3 {C:\NMR} mdsgrp 27
CHO
Me
O
N
Me
32
220 210 200 190 180 170 160 150 140 130 120 110 100 90 80 70 60 50 40 30 20 10 0 -10 -20
f1 (ppm)

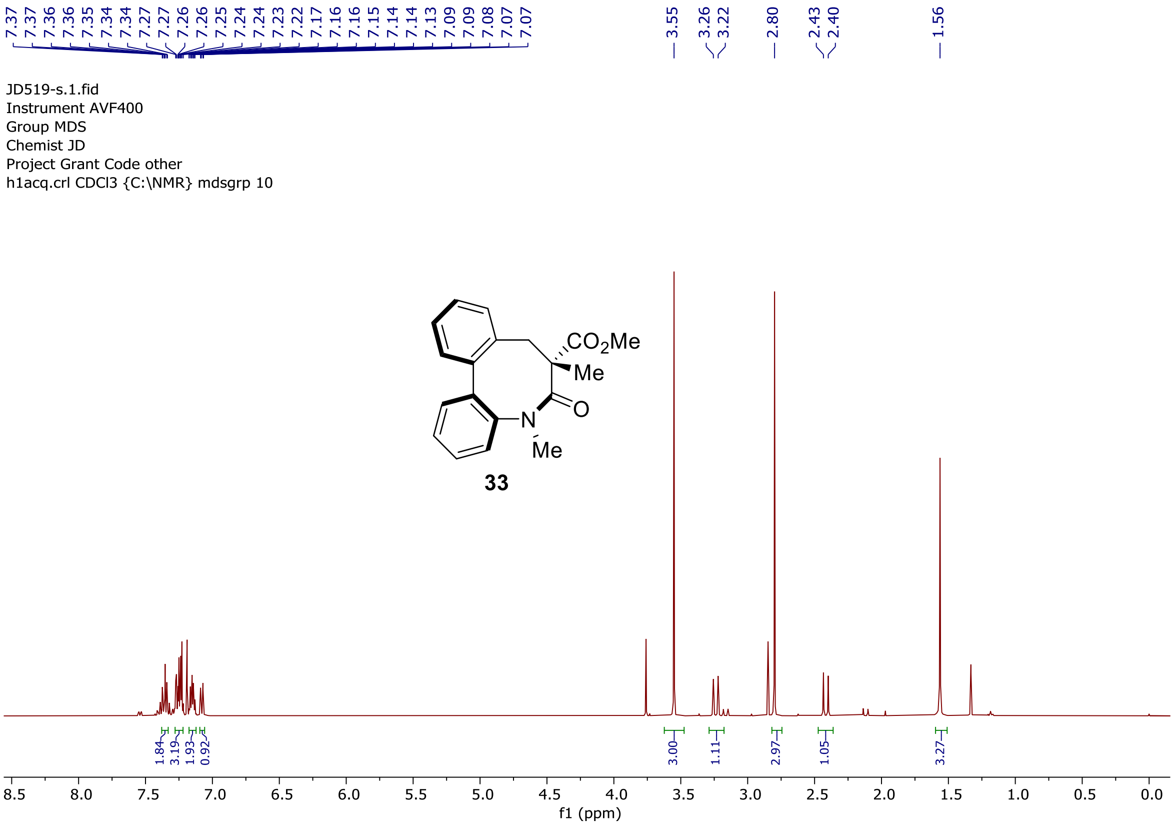
7.37
7.37
7.36
7.36
7.35
7.34
7.34
7.27
7.27
7.26
7.26
7.25
7.24
7.24
7.23
7.22
7.17
7.16
7.16
7.15
7.14
7.14
7.13
7.09
7.09
7.08
7.07
7.07
3.55
3.26
3.22
2.80
2.43
2.40
1.56
JD519-s.1.fid
Instrument AVF400
Group MDS
Chemist JD
Project Grant Code other
h1acq.crl CDCl3 {C:\NMR} mdsgrp 10
CO2Me
Me
O
N
Me
33
1.84
3.19
1.93
0.92
3.00
1.11
2.97
1.05
3.27
8.5 8.0 7.5 7.0 6.5 6.0 5.5 5.0 4.5 4.0 3.5 3.0 2.5 2.0 1.5 1.0 0.5 0.0
f1 (ppm)

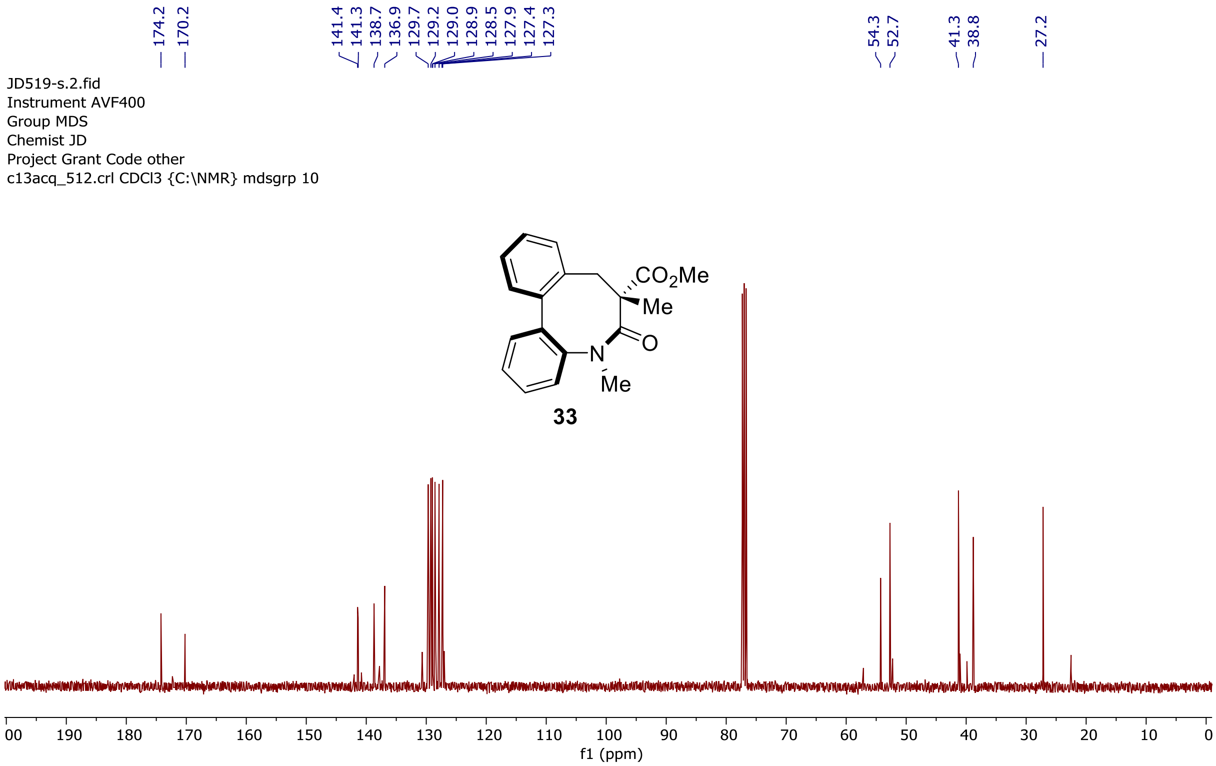
174.2
170.2
141.4
141.3
138.7
136.9
129.7
129.2
129.0
128.9
128.5
127.9
127.4
127.3
54.3
52.7
41.3
38.8
27.2
JD519-s.2.fid
Instrument AVF400
Group MDS
Chemist JD
Project Grant Code other
c13acq_512.crl CDCl3 {C:\NMR} mdsgrp 10
CO2Me
Me
O
N
Me
33
f1 (ppm)

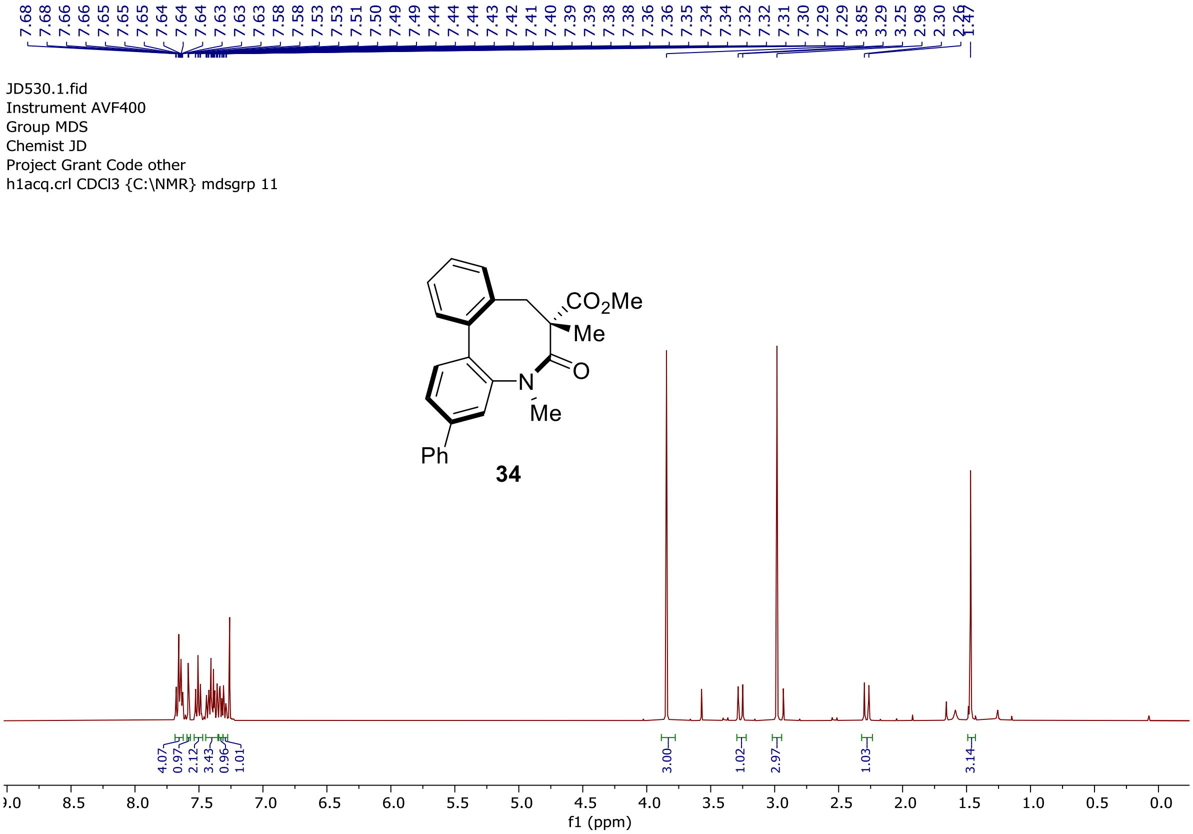
7.68
7.68
7.66
7.66
7.65
7.65
7.65
7.64
7.64
7.63
7.63
7.58
7.58
7.53
7.51
7.50
7.49
7.49
7.44
7.44
7.43
7.42
7.41
7.40
7.39
7.38
7.38
7.36
7.36
7.35
7.34
7.34
7.32
7.32
7.31
7.30
7.29
7.29
3.85
3.29
3.25
2.98
2.30
2.26
1.47
JD530.1.fid
Instrument AVF400
Group MDS
Chemist JD
Project Grant Code other
h1acq.crl CDCl3 {C:\NMR} mdsgrp 11
CO2Me
Me
O
N
Me
Ph
34
4.07
0.97
2.12
3.43
0.96
1.01
3.00
1.02
2.97
1.03
3.14
f1 (ppm)

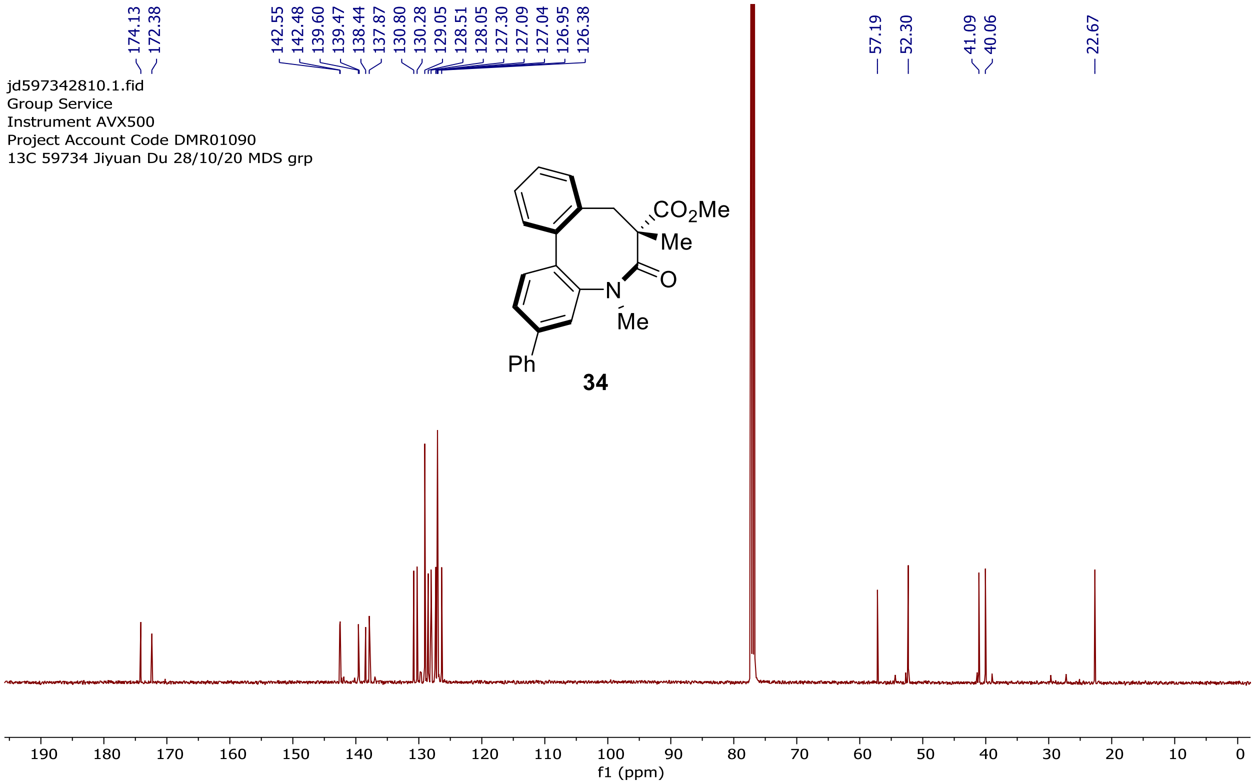
174.13
172.38
142.55
142.48
139.60
139.47
138.44
137.87
130.80
130.28
129.05
128.51
128.05
127.30
127.09
127.04
126.95
126.38
57.19
52.30
41.09
40.06
22.67
jd597342810.1.fid
Group Service
Instrument AVX500
Project Account Code DMR01090
13C 59734 Jiyuan Du 28/10/20 MDS grp
CO2Me
Me
N
O
Me
Ph
34
190 180 170 160 150 140 130 120 110 100 90 80 70 60 50 40 30 20 10 0
f1 (ppm)

Chromatogram and Results

Injection Details

Injection Name:	**JD509(rac)**	*Run Time (min):*	**33.00**
Vial Number:	**BE1**	*Injection Volume:*	**20.00**
Injection Type:	**Unknown**	*Channel:*	**UV_VIS_3**
Calibration Level:		*Wavelength:*	**222**
Instrument Method:	**50% IC 1mL IPA-pre-equ-33 min run**	*Bandwidth:*	**4**
Processing Method:	**Standard Processing Method**	*Dilution Factor:*	**1.0000**
Injection Date/Time:	**07/Oct/20 13:15**	*Sample Weight:*	**1.0000**

Chromatogram

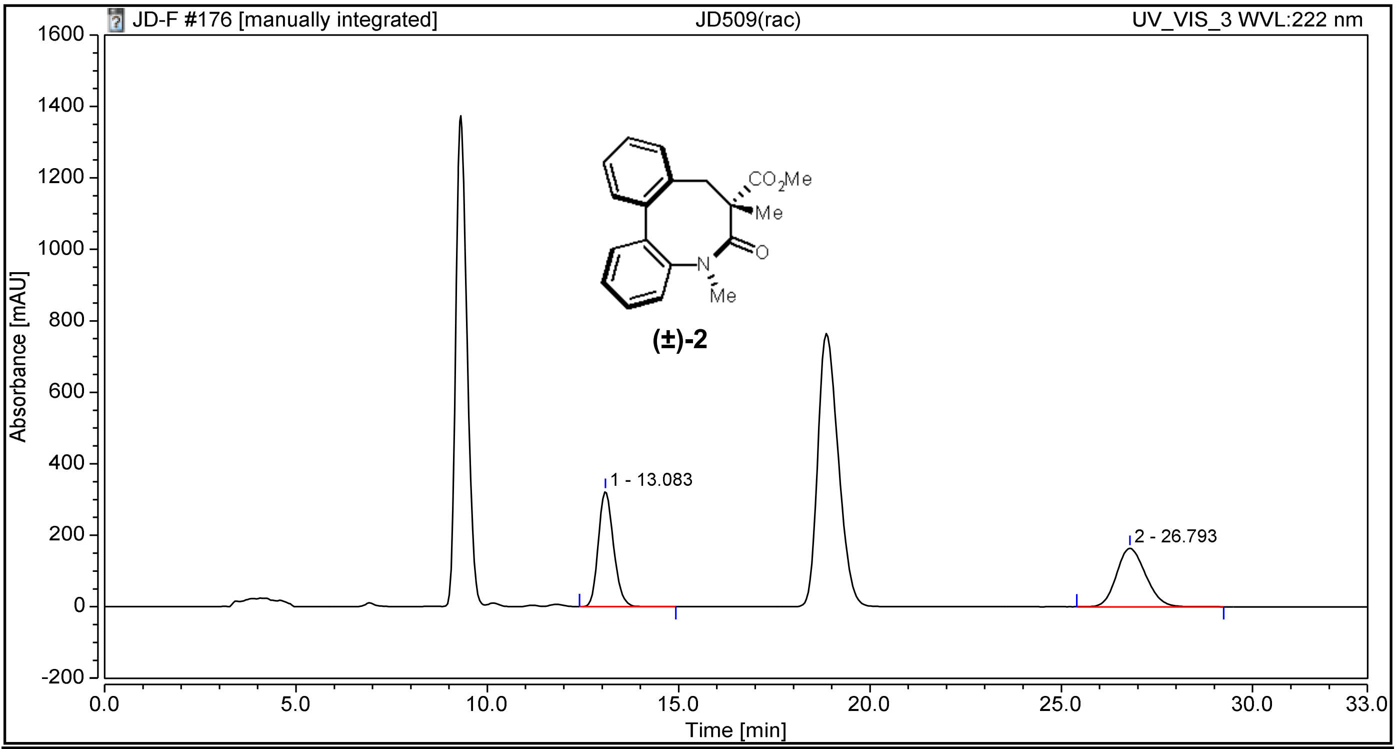

Integration Results

No.	Peak Name	Retention Time min	Area mAU*min	Height mAU	Relative Area %	Relative Height %	Amount n.a.
1		13.083	149.010	322.702	50.80	66.37	n.a.
2		26.793	144.330	163.539	49.20	33.63	n.a.
**Total:**			**293.340**	**486.241**	**100.00**	**100.00**	

Chromatogram and Results

Injection Details

Injection Name:	**JD511**	*Run Time (min):*	**33.00**
Vial Number:	**BE2**	*Injection Volume:*	**20.00**
Injection Type:	**Unknown**	*Channel:*	**UV_VIS_3**
Calibration Level:		*Wavelength:*	**222**
Instrument Method:	**50%IPA -IC 1mL 33 min run**	*Bandwidth:*	**4**
Processing Method:	**Standard Processing Method**	*Dilution Factor:*	**1.0000**
Injection Date/Time:	**07/Oct/20 13:49**	*Sample Weight:*	**1.0000**

Chromatogram

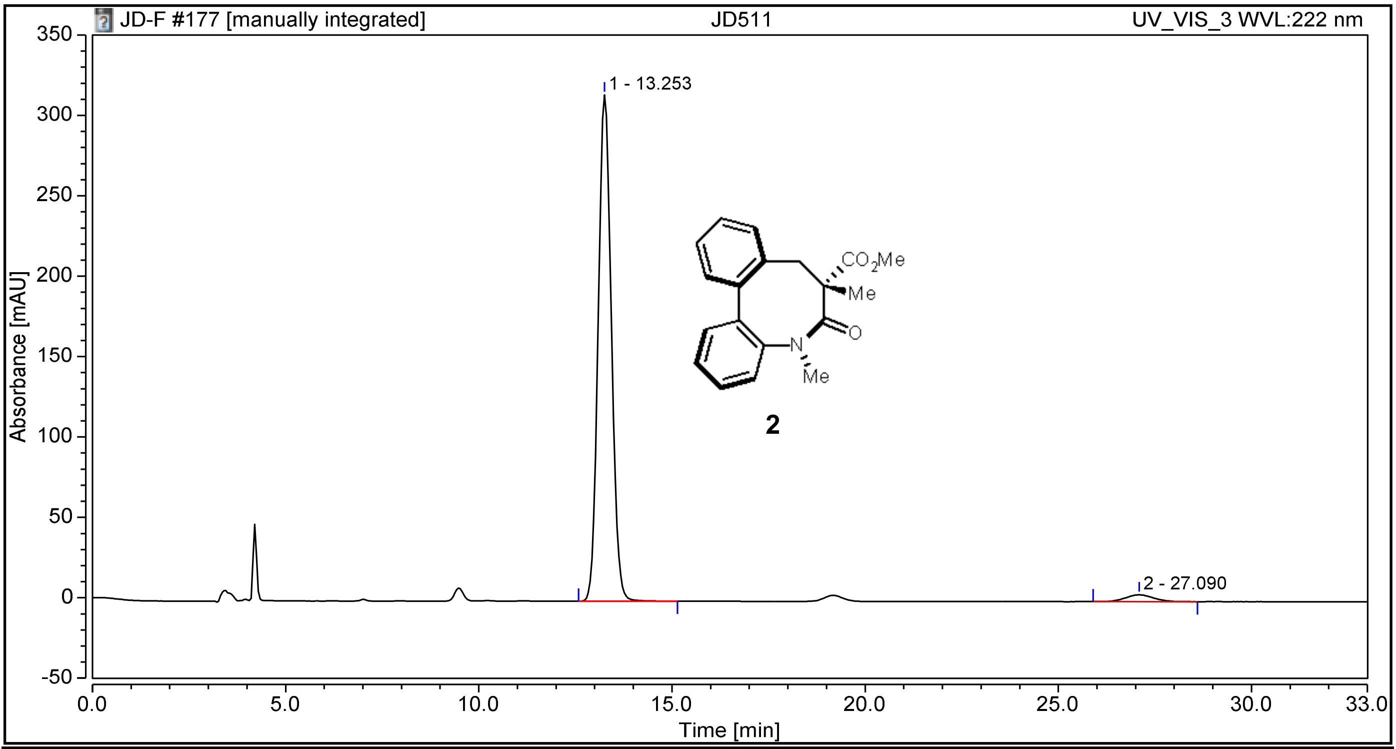

Integration Results

No.	Peak Name	Retention Time min	Area mAU*min	Height mAU	Relative Area %	Relative Height %	Amount n.a.
1		13.253	123.945	314.798	97.19	98.67	n.a.
2		27.090	3.585	4.256	2.81	1.33	n.a.
**Total:**			**127.530**	**319.054**	**100.00**	**100.00**	

Chromatogram and Results

Injection Details

Injection Name:	**JD360(rac)-s**	*Run Time (min):*	**33.00**
Vial Number:	**GA2**	*Injection Volume:*	**20.00**
Injection Type:	**Unknown**	*Channel:*	**UV_VIS_3**
Calibration Level:		*Wavelength:*	**222**
Instrument Method:	**50% IC-33min run**	*Bandwidth:*	**4**
Processing Method:	**Standard Processing Method**	*Dilution Factor:*	**1.0000**
Injection Date/Time:	**03/Jul/20 11:45**	*Sample Weight:*	**1.0000**

Chromatogram

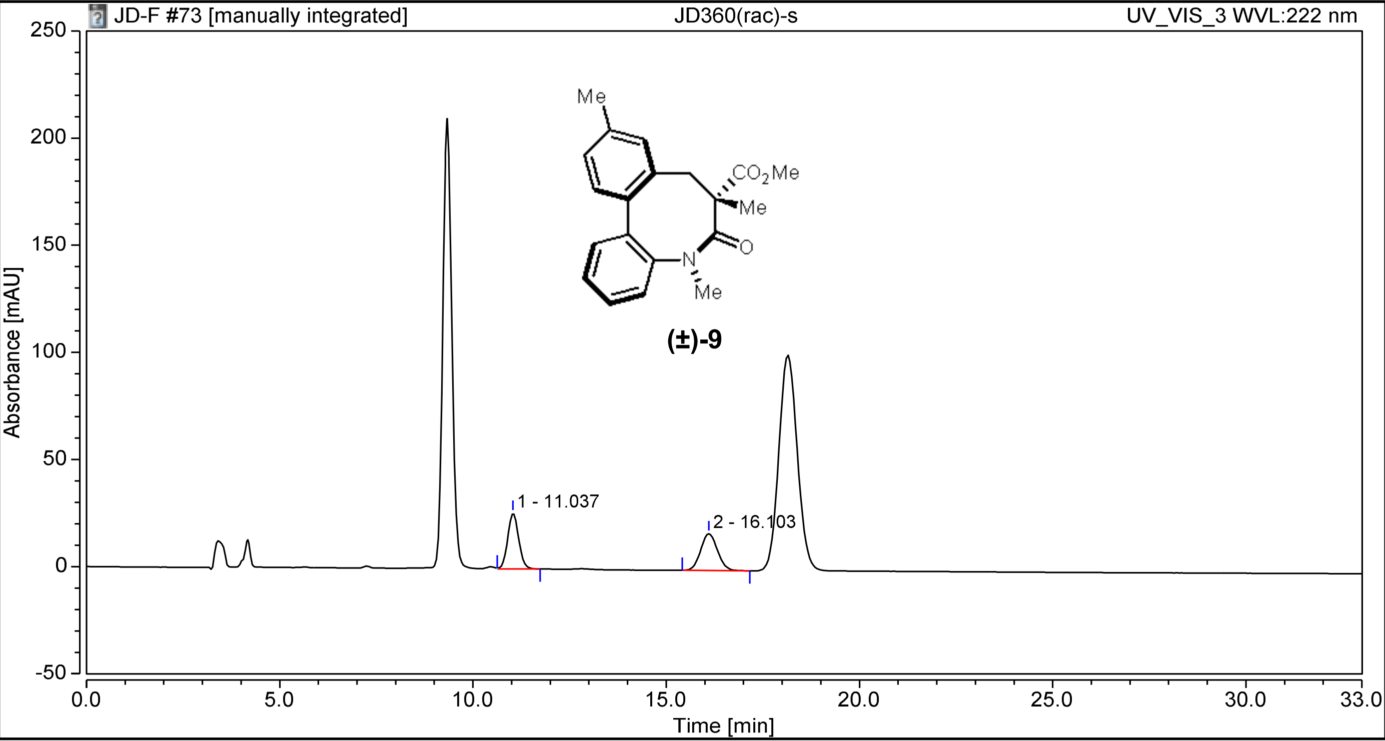

Integration Results

No.	Peak Name	Retention Time min	Area mAU*min	Height mAU	Relative Area %	Relative Height %	Amount n.a.
1		11.037	8.573	25.932	49.78	60.12	n.a.
2		16.103	8.650	17.203	50.22	39.88	n.a.
**Total:**			**17.223**	**43.136**	**100.00**	**100.00**	

Chromatogram and Results

Injection Details

Injection Name:	**JD359**	*Run Time (min):*	**33.00**
Vial Number:	**GA3**	*Injection Volume:*	**20.00**
Injection Type:	**Unknown**	*Channel:*	**UV_VIS_3**
Calibration Level:		*Wavelength:*	**222**
Instrument Method:	**50% IC-33min run**	*Bandwidth:*	**4**
Processing Method:	**Standard Processing Method**	*Dilution Factor:*	**1.0000**
Injection Date/Time:	**03/Jul/20 11:11**	*Sample Weight:*	**1.0000**

Chromatogram

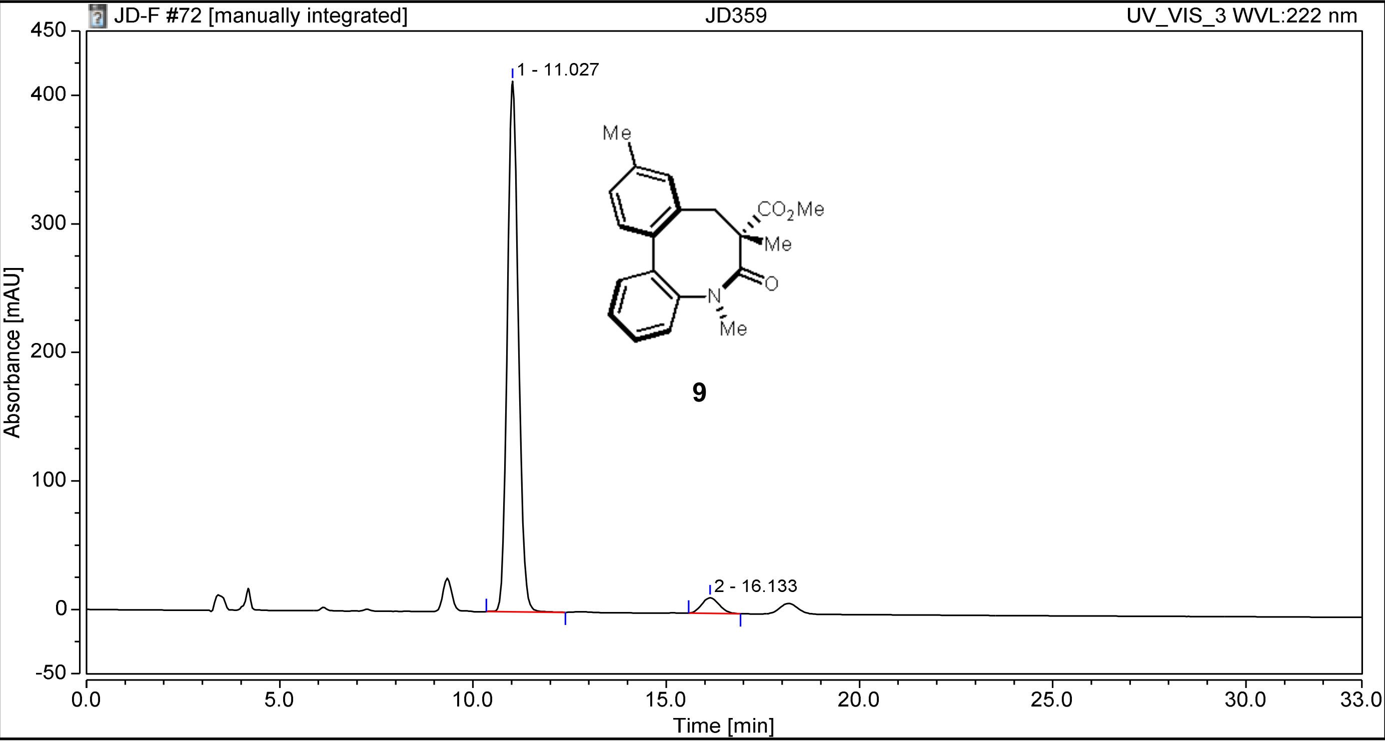

Integration Results

No.	Peak Name	Retention Time min	Area mAU*min	Height mAU	Relative Area %	Relative Height %	Amount n.a.
1		11.027	136.965	412.685	95.59	97.16	n.a.
2		16.133	6.320	12.080	4.41	2.84	n.a.
**Total:**			**143.284**	**424.765**	**100.00**	**100.00**	

Chromatogram and Results

Injection Details

Injection Name:	**JD382(rac)**	*Run Time (min):*	**40.00**
Vial Number:	**GE1**	*Injection Volume:*	**10.00**
Injection Type:	**Unknown**	*Channel:*	**UV_VIS_3**
Calibration Level:		*Wavelength:*	**222**
Instrument Method:	**3% ADH-pre-equ-40min run**	*Bandwidth:*	**4**
Processing Method:	**Standard Processing Method**	*Dilution Factor:*	**1.0000**
Injection Date/Time:	**22/Jul/20 17:18**	*Sample Weight:*	**1.0000**

Chromatogram

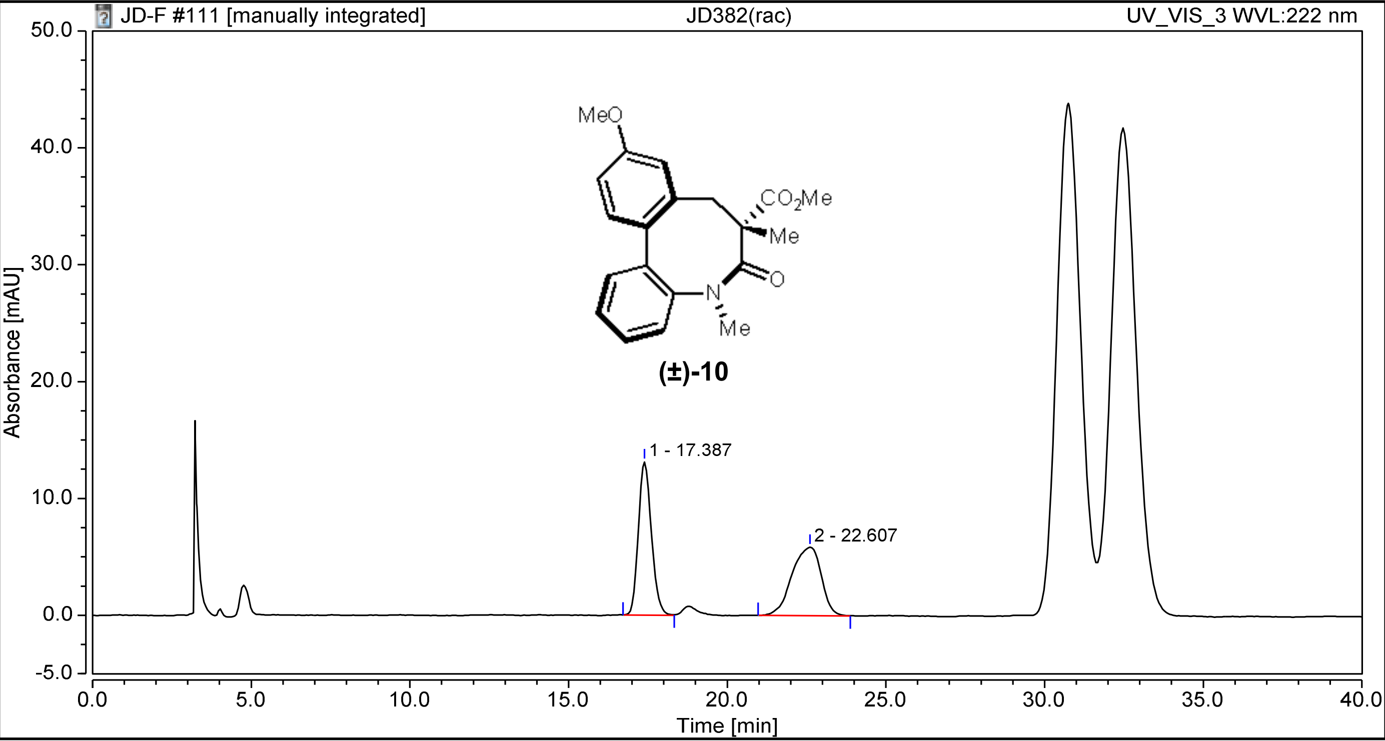

(±)-10

Integration Results

No.	Peak Name	Retention Time min	Area mAU*min	Height mAU	Relative Area %	Relative Height %	Amount n.a.
1		17.387	6.258	13.117	49.95	69.12	n.a.
2		22.607	6.271	5.860	50.05	30.88	n.a.
**Total:**			**12.529**	**18.977**	**100.00**	**100.00**	

Chromatogram and Results

Injection Details

Injection Name:	**JD381**	*Run Time (min):*	**40.00**
Vial Number:	**GE2**	*Injection Volume:*	**10.00**
Injection Type:	**Unknown**	*Channel:*	**UV_VIS_3**
Calibration Level:		*Wavelength:*	**222**
Instrument Method:	**3% ADH 40min run**	*Bandwidth:*	**4**
Processing Method:	**Standard Processing Method**	*Dilution Factor:*	**1.0000**
Injection Date/Time:	**22/Jul/20 17:59**	*Sample Weight:*	**1.0000**

Chromatogram

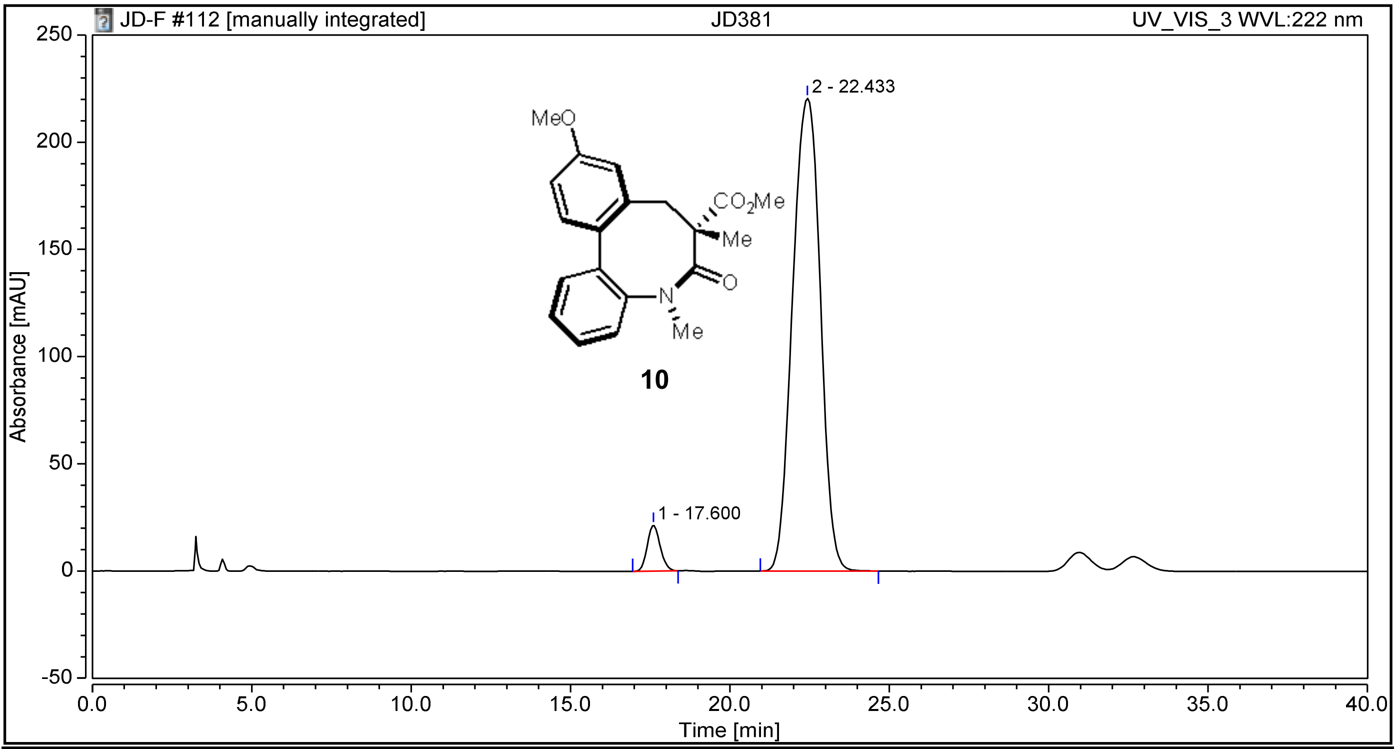

Integration Results

No.	Peak Name	Retention Time min	Area mAU*min	Height mAU	Relative Area %	Relative Height %	Amount n.a.
1		17.600	10.031	21.415	4.31	8.85	n.a.
2		22.433	222.632	220.497	95.69	91.15	n.a.
**Total:**			**232.662**	**241.912**	**100.00**	**100.00**	

Chromatogram and Results

Injection Details

Injection Name:	**JD384(rac)**	*Run Time (min):*	**40.00**
Vial Number:	**GE1**	*Injection Volume:*	**10.00**
Injection Type:	**Unknown**	*Channel:*	**UV_VIS_3**
Calibration Level:		*Wavelength:*	**222**
Instrument Method:	**IC 50% 0.7mL-pre-equ-40 min run**	*Bandwidth:*	**4**
Processing Method:	**Standard Processing Method**	*Dilution Factor:*	**1.0000**
Injection Date/Time:	**22/Jul/20 11:45**	*Sample Weight:*	**1.0000**

Chromatogram

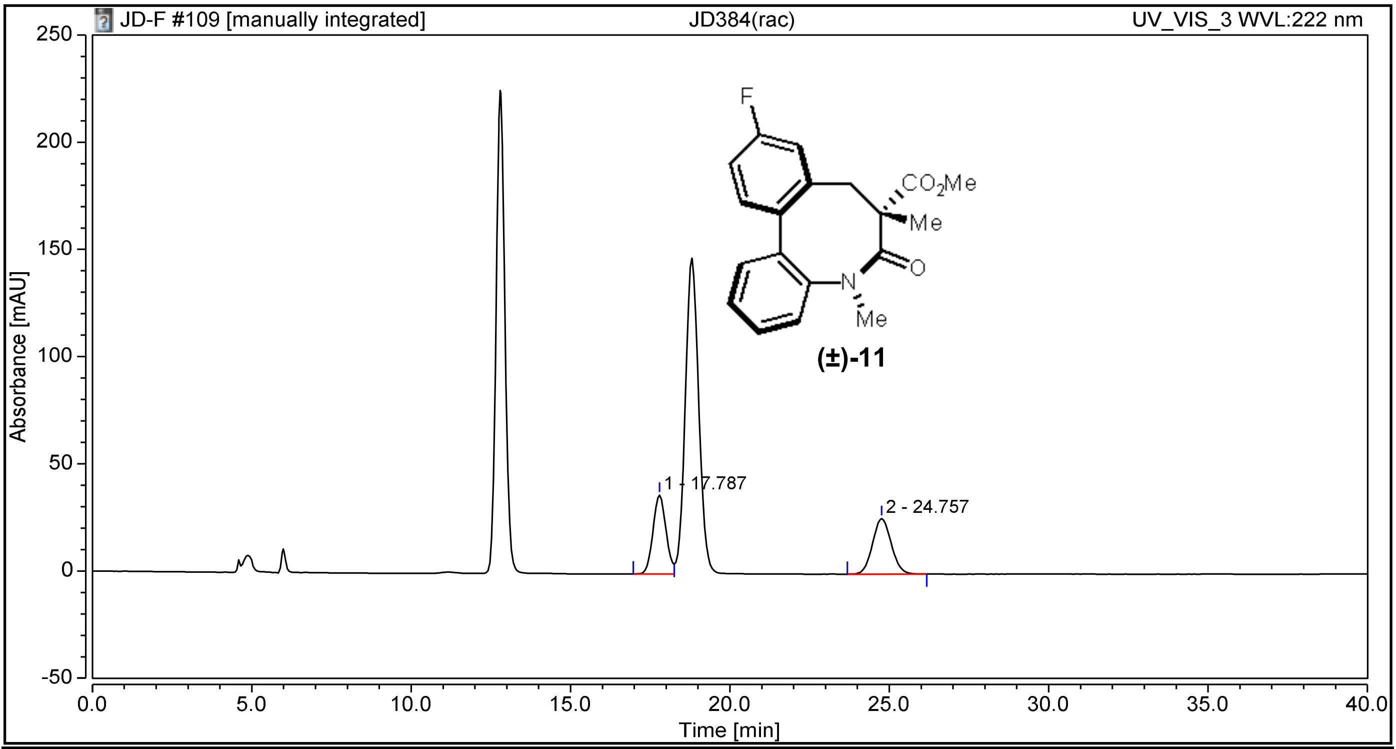

Integration Results

No.	Peak Name	Retention Time min	Area mAU*min	Height mAU	Relative Area %	Relative Height %	Amount n.a.
1		17.787	17.404	36.791	49.75	58.70	n.a.
2		24.757	17.580	25.884	50.25	41.30	n.a.
**Total:**			**34.984**	**62.675**	**100.00**	**100.00**	

Chromatogram and Results

Injection Details

Injection Name:	**JD383**	*Run Time (min):*	**40.00**
Vial Number:	**GE2**	*Injection Volume:*	**10.00**
Injection Type:	**Unknown**	*Channel:*	**UV_VIS_3**
Calibration Level:		*Wavelength:*	**222**
Instrument Method:	**IC 50% 0.7mL-40 min run**	*Bandwidth:*	**4**
Processing Method:	**Standard Processing Method**	*Dilution Factor:*	**1.0000**
Injection Date/Time:	**22/Jul/20 12:26**	*Sample Weight:*	**1.0000**

Chromatogram

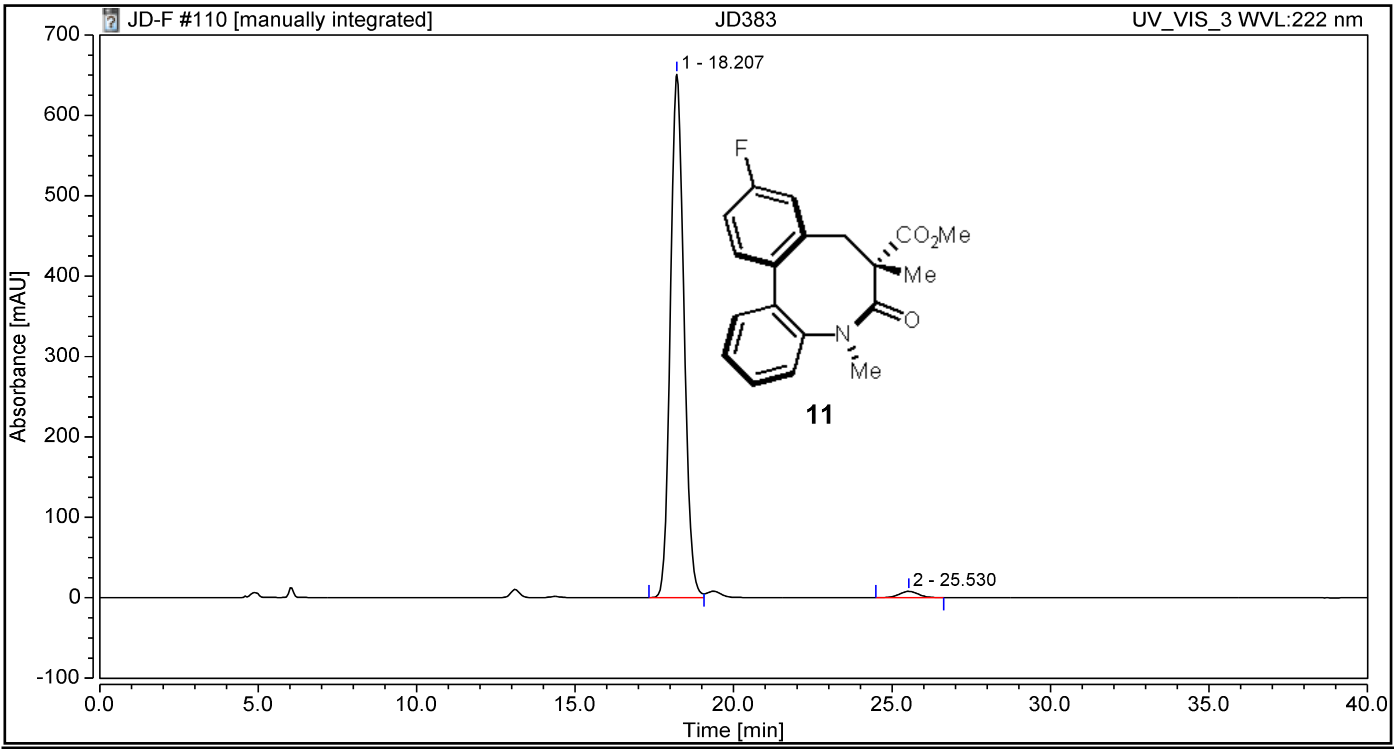

Integration Results

No.	Peak Name	Retention Time min	Area mAU*min	Height mAU	Relative Area %	Relative Height %	Amount n.a.
1		18.207	324.242	650.701	98.31	98.80	n.a.
2		25.530	5.563	7.886	1.69	1.20	n.a.
**Total:**			**329.804**	**658.586**	**100.00**	**100.00**	

Chromatogram and Results

Injection Details

Injection Name:	**JD462(rac)**	*Run Time (min):*	**33.00**
Vial Number:	**BE3**	*Injection Volume:*	**10.00**
Injection Type:	**Unknown**	*Channel:*	**UV_VIS_3**
Calibration Level:		*Wavelength:*	**222**
Instrument Method:	**50%IPA -IC 1mL 33 min run**	*Bandwidth:*	**4**
Processing Method:	**Standard Processing Method**	*Dilution Factor:*	**1.0000**
Injection Date/Time:	**04/Sep/20 15:29**	*Sample Weight:*	**1.0000**

Chromatogram

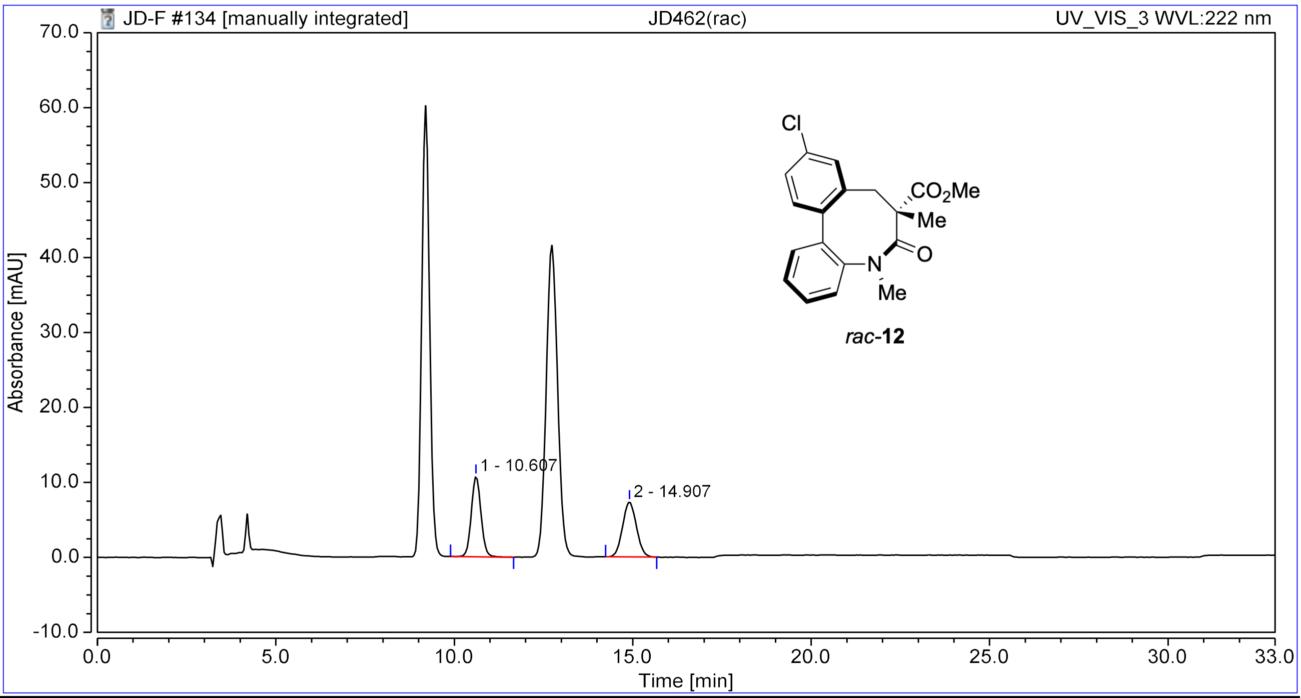

Integration Results

No.	Peak Name	Retention Time min	Area mAU*min	Height mAU	Relative Area %	Relative Height %	Amount n.a.
1		10.607	3.366	10.716	50.50	59.46	n.a.
2		14.907	3.299	7.306	49.50	40.54	n.a.
**Total:**			**6.666**	**18.022**	**100.00**	**100.00**	

Chromatogram and Results

Injection Details

Injection Name:	**JD461**	*Run Time (min):*	**33.00**
Vial Number:	**BE4**	*Injection Volume:*	**10.00**
Injection Type:	**Unknown**	*Channel:*	**UV_VIS_3**
Calibration Level:		*Wavelength:*	**222**
Instrument Method:	**50%IPA -IC 1mL 33 min run**	*Bandwidth:*	**4**
Processing Method:	**Standard Processing Method**	*Dilution Factor:*	**1.0000**
Injection Date/Time:	**04/Sep/20 16:03**	*Sample Weight:*	**1.0000**

Chromatogram

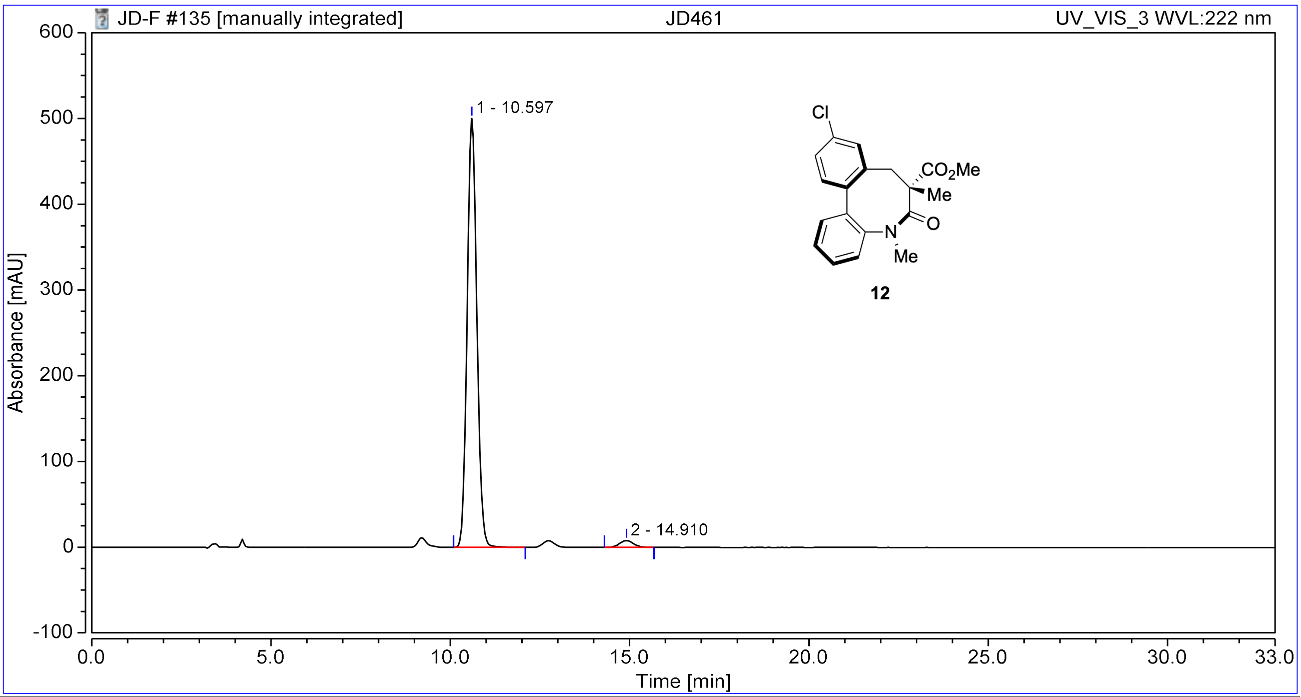

Integration Results

No.	Peak Name	Retention Time<br>min	Area<br>mAU*min	Height<br>mAU	Relative Area<br>%	Relative Height<br>%	Amount<br>n.a.
1		10.597	156.730	500.271	97.76	98.44	n.a.
2		14.910	3.588	7.929	2.24	1.56	n.a.
**Total:**			**160.317**	**508.200**	**100.00**	**100.00**	

Chromatogram and Results

Injection Details

Injection Name:	**JD457(rac)**	*Run Time (min):*	**33.00**
Vial Number:	**BE7**	*Injection Volume:*	**10.00**
Injection Type:	**Unknown**	*Channel:*	**UV_VIS_3**
Calibration Level:		*Wavelength:*	**222**
Instrument Method:	**50% IC-pre-equ-33min run**	*Bandwidth:*	**4**
Processing Method:	**Standard Processing Method**	*Dilution Factor:*	**1.0000**
Injection Date/Time:	**02/Sep/20 14:32**	*Sample Weight:*	**1.0000**

Chromatogram

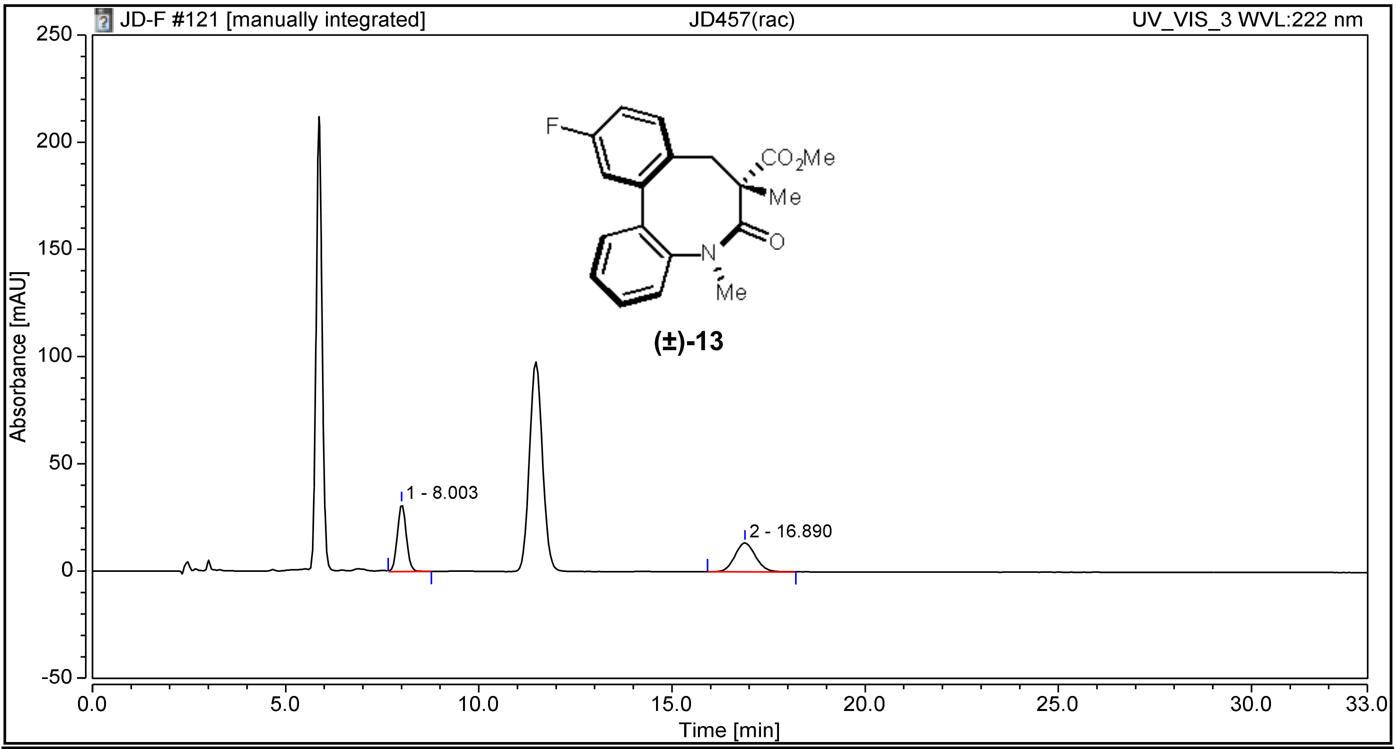

Integration Results

No.	Peak Name	Retention Time min	Area mAU*min	Height mAU	Relative Area %	Relative Height %	Amount n.a.
1		8.003	8.493	31.388	50.40	69.98	n.a.
2		16.890	8.356	13.462	49.60	30.02	n.a.
**Total:**			**16.849**	**44.851**	**100.00**	**100.00**	

Chromatogram and Results

Injection Details

Injection Name:	**JD456**	*Run Time (min):*	**33.00**
Vial Number:	**BE8**	*Injection Volume:*	**10.00**
Injection Type:	**Unknown**	*Channel:*	**UV_VIS_3**
Calibration Level:		*Wavelength:*	**222**
Instrument Method:	**50% IC-pre-equ-33min run**	*Bandwidth:*	**4**
Processing Method:	**Standard Processing Method**	*Dilution Factor:*	**1.0000**
Injection Date/Time:	**02/Sep/20 15:16**	*Sample Weight:*	**1.0000**

Chromatogram

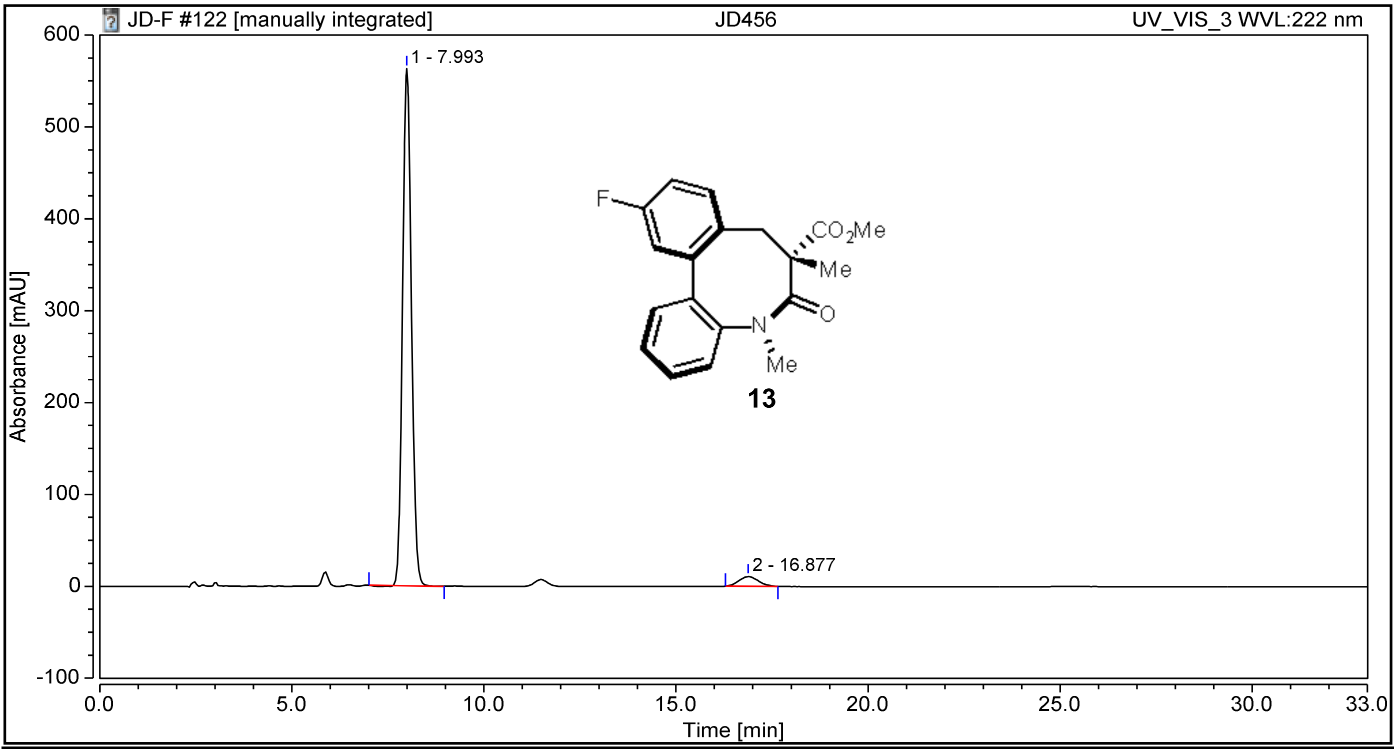

Integration Results

No.	Peak Name	Retention Time<br>min	Area<br>mAU*min	Height<br>mAU	Relative Area<br>%	Relative Height<br>%	Amount<br>n.a.
1		7.993	149.487	563.037	96.03	98.18	n.a.
2		16.877	6.185	10.462	3.97	1.82	n.a.
**Total:**			**155.671**	**573.498**	**100.00**	**100.00**	

Chromatogram and Results

Injection Details

Injection Name:	**JD466(rac)**	*Run Time (min):*	**33.00**
Vial Number:	**BE5**	*Injection Volume:*	**10.00**
Injection Type:	**Unknown**	*Channel:*	**UV_VIS_3**
Calibration Level:		*Wavelength:*	**222**
Instrument Method:	**50%IPA -IC 1mL 33 min run**	*Bandwidth:*	**4**
Processing Method:	**Standard Processing Method**	*Dilution Factor:*	**1.0000**
Injection Date/Time:	**04/Sep/20 16:37**	*Sample Weight:*	**1.0000**

Chromatogram

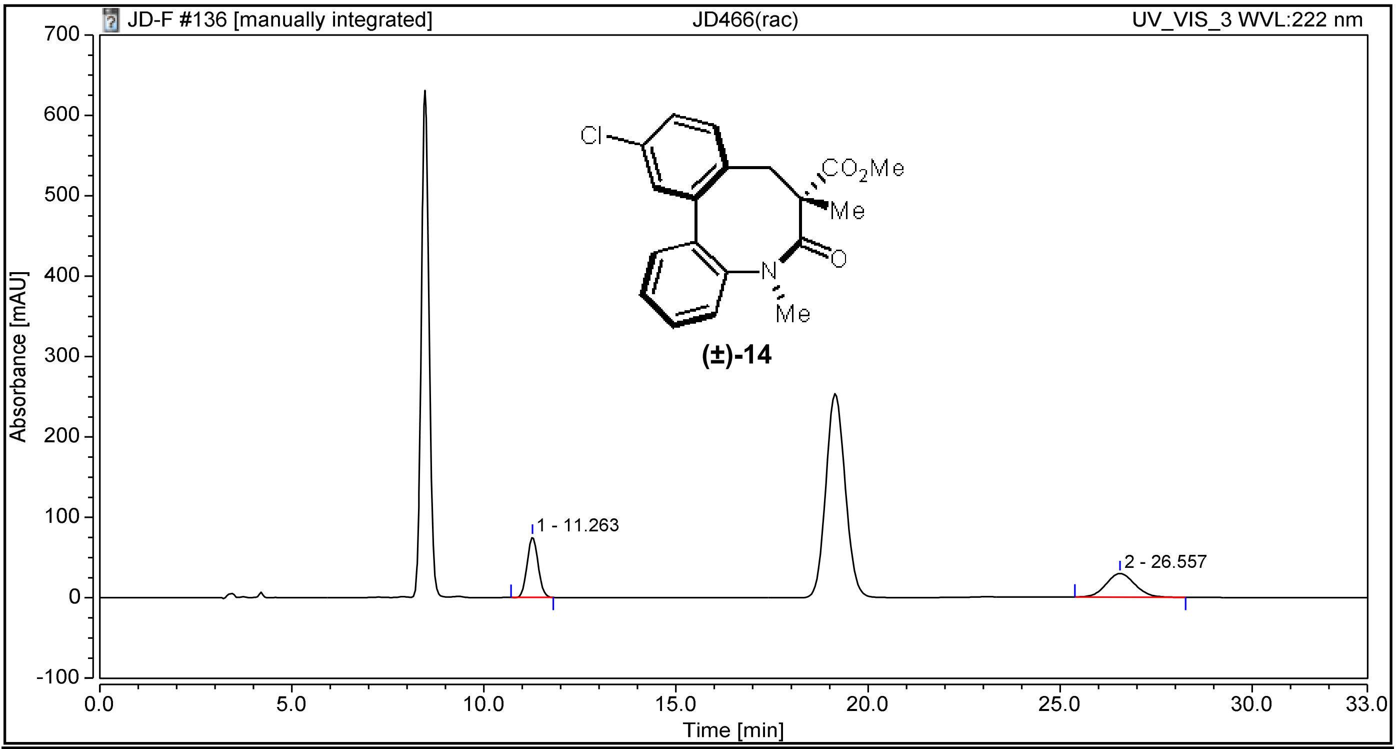

Integration Results

No.	Peak Name	Retention Time min	Area mAU*min	Height mAU	Relative Area %	Relative Height %	Amount n.a.
1		11.263	24.847	75.025	49.55	71.90	n.a.
2		26.557	25.302	29.314	50.45	28.10	n.a.
**Total:**			**50.149**	**104.339**	**100.00**	**100.00**	

Chromatogram and Results

Injection Details

Injection Name:	**JD465**	*Run Time (min):*	**33.00**
Vial Number:	**BE6**	*Injection Volume:*	**10.00**
Injection Type:	**Unknown**	*Channel:*	**UV_VIS_3**
Calibration Level:		*Wavelength:*	**222**
Instrument Method:	**50%IPA -IC 1mL 33 min run**	*Bandwidth:*	**4**
Processing Method:	**Standard Processing Method**	*Dilution Factor:*	**1.0000**
Injection Date/Time:	**04/Sep/20 17:11**	*Sample Weight:*	**1.0000**

Chromatogram

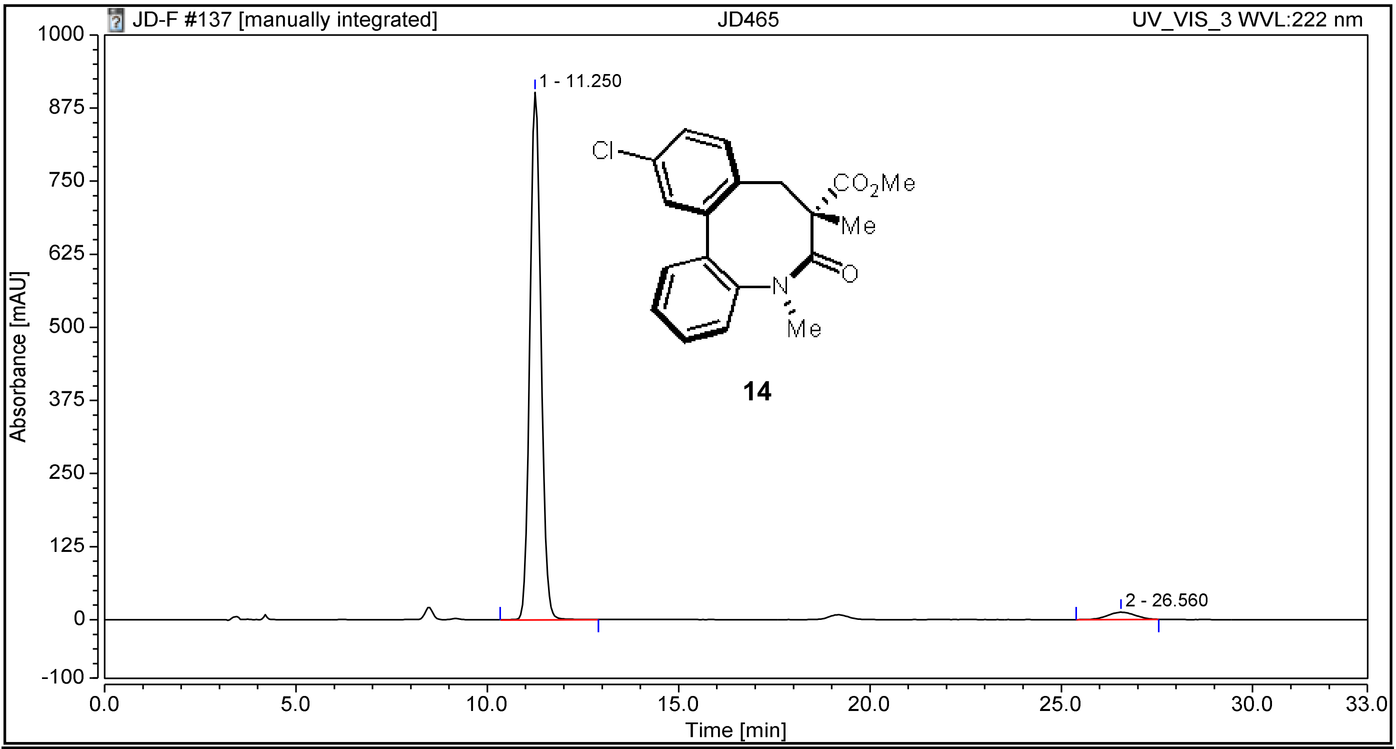

Integration Results

No.	Peak Name	Retention Time<br>min	Area<br>mAU*min	Height<br>mAU	Relative Area<br>%	Relative Height<br>%	Amount<br>n.a.
1		11.250	304.071	902.073	96.71	98.64	n.a.
2		26.560	10.357	12.417	3.29	1.36	n.a.
**Total:**			**314.428**	**914.490**	**100.00**	**100.00**	

Chromatogram and Results

Injection Details

Injection Name:	**JD451(rac)-s**	*Run Time (min):*	**33.00**
Vial Number:	**BE1**	*Injection Volume:*	**10.00**
Injection Type:	**Unknown**	*Channel:*	**UV_VIS_3**
Calibration Level:		*Wavelength:*	**222**
Instrument Method:	**50% IC-pre-equ-33min run**	*Bandwidth:*	**4**
Processing Method:	**Standard Processing Method**	*Dilution Factor:*	**1.0000**
Injection Date/Time:	**02/Sep/20 16:00**	*Sample Weight:*	**1.0000**

Chromatogram

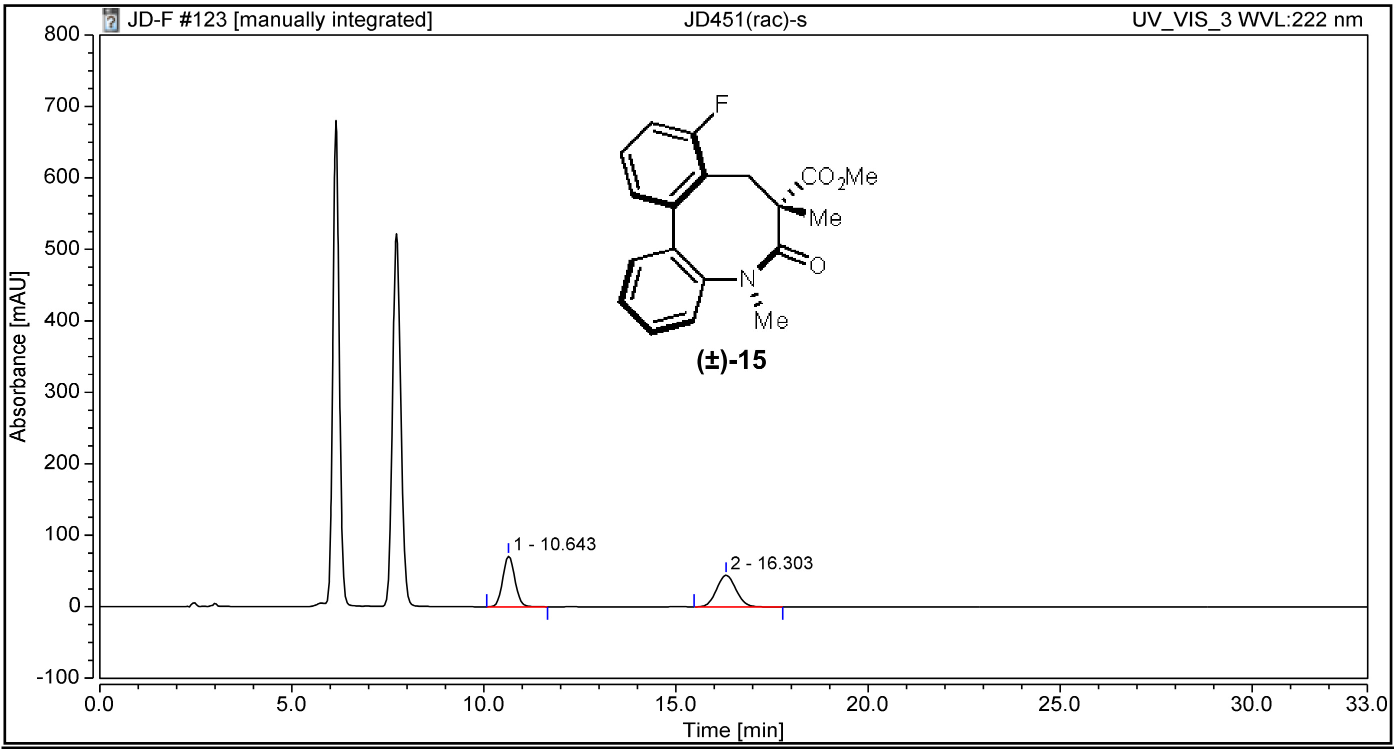

Integration Results

No.	Peak Name	Retention Time min	Area mAU*min	Height mAU	Relative Area %	Relative Height %	Amount n.a.
1		10.643	26.019	70.855	50.13	61.56	n.a.
2		16.303	25.889	44.245	49.87	38.44	n.a.
**Total:**			**51.908**	**115.101**	**100.00**	**100.00**	

Chromatogram and Results

Injection Details

Injection Name:	**JD450**	*Run Time (min):*	**33.00**
Vial Number:	**BE2**	*Injection Volume:*	**10.00**
Injection Type:	**Unknown**	*Channel:*	**UV_VIS_3**
Calibration Level:		*Wavelength:*	**222**
Instrument Method:	**50% IC-pre-equ-33min run**	*Bandwidth:*	**4**
Processing Method:	**Standard Processing Method**	*Dilution Factor:*	**1.0000**
Injection Date/Time:	**02/Sep/20 10:51**	*Sample Weight:*	**1.0000**

Chromatogram

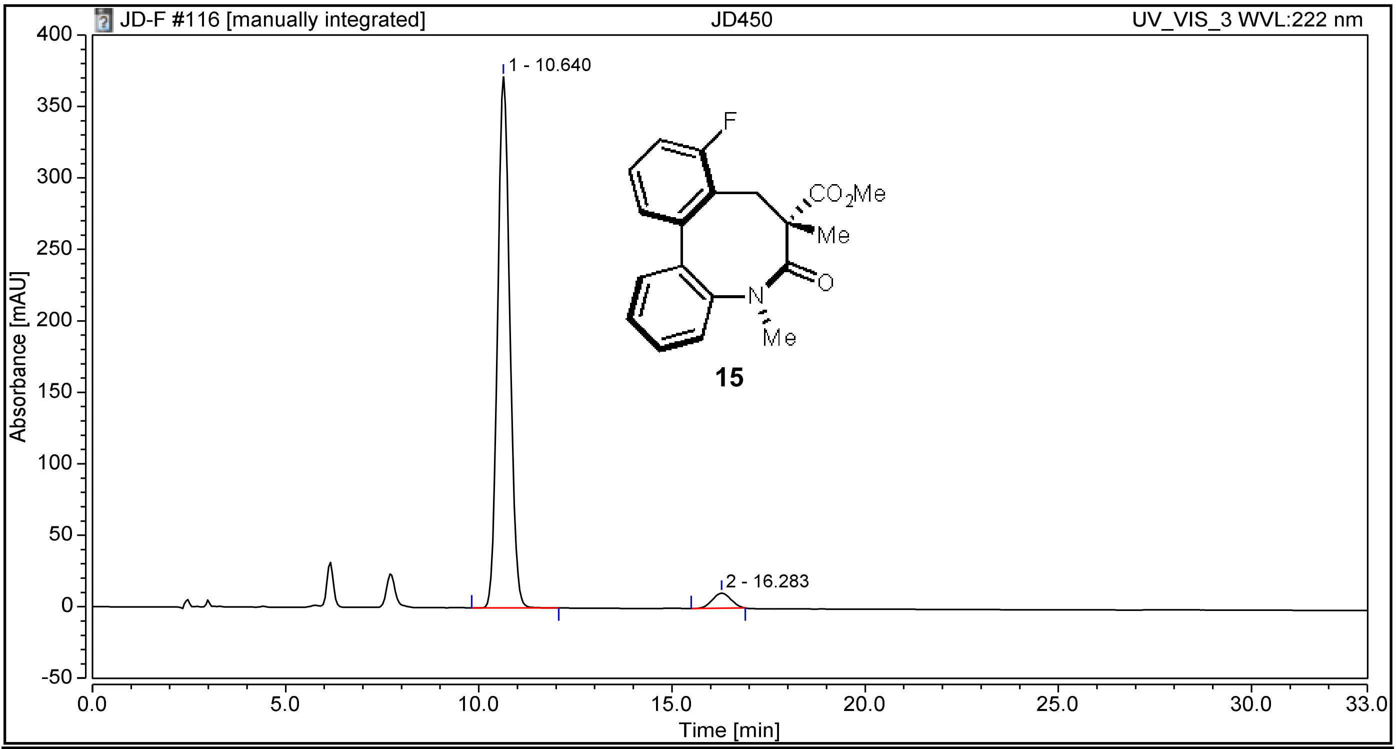

15

Integration Results

No.	Peak Name	Retention Time min	Area mAU*min	Height mAU	Relative Area %	Relative Height %	Amount n.a.
1		10.640	136.215	371.515	95.83	97.24	n.a.
2		16.283	5.924	10.562	4.17	2.76	n.a.
**Total:**			**142.138**	**382.076**	**100.00**	**100.00**	

Chromatogram and Results

Injection Details

Injection Name:	**JD481(rac)**	*Run Time (min):*	**50.00**
Vial Number:	**BE1**	*Injection Volume:*	**20.00**
Injection Type:	**Unknown**	*Channel:*	**UV_VIS_3**
Calibration Level:		*Wavelength:*	**222**
Instrument Method:	**50% IPA-IC 1mL50min run**	*Bandwidth:*	**4**
Processing Method:	**Standard Processing Method**	*Dilution Factor:*	**1.0000**
Injection Date/Time:	**10/Sep/20 17:36**	*Sample Weight:*	**1.0000**

Chromatogram

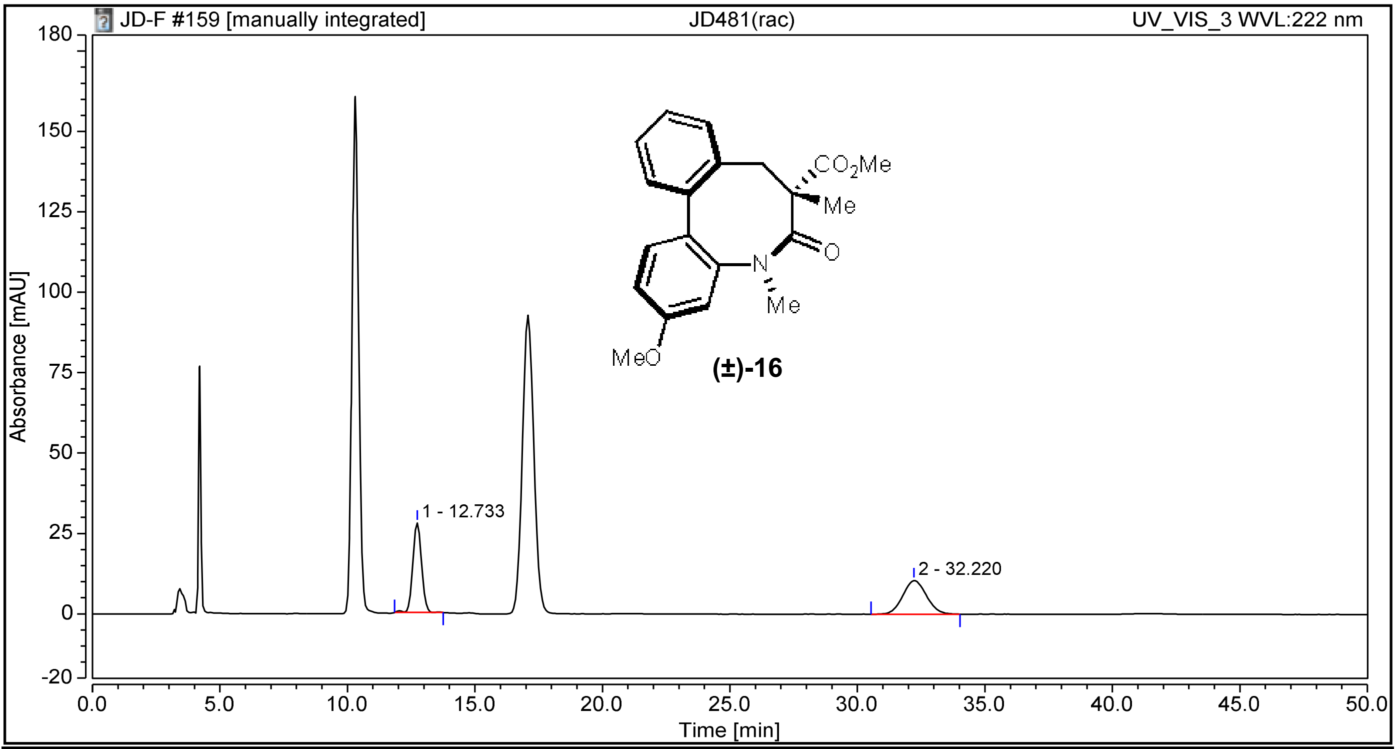

Integration Results

No.	Peak Name	Retention Time min	Area mAU*min	Height mAU	Relative Area %	Relative Height %	Amount n.a.
1		12.733	11.081	27.805	49.58	72.73	n.a.
2		32.220	11.268	10.426	50.42	27.27	n.a.
**Total:**			**22.349**	**38.232**	**100.00**	**100.00**	

Chromatogram and Results

Injection Details

Injection Name:	**JD479**	*Run Time (min):*	**50.00**
Vial Number:	**BE2**	*Injection Volume:*	**20.00**
Injection Type:	**Unknown**	*Channel:*	**UV_VIS_3**
Calibration Level:		*Wavelength:*	**222**
Instrument Method:	**50% IPA-IC 1mL50min run**	*Bandwidth:*	**4**
Processing Method:	**Standard Processing Method**	*Dilution Factor:*	**1.0000**
Injection Date/Time:	**10/Sep/20 15:03**	*Sample Weight:*	**1.0000**

Chromatogram

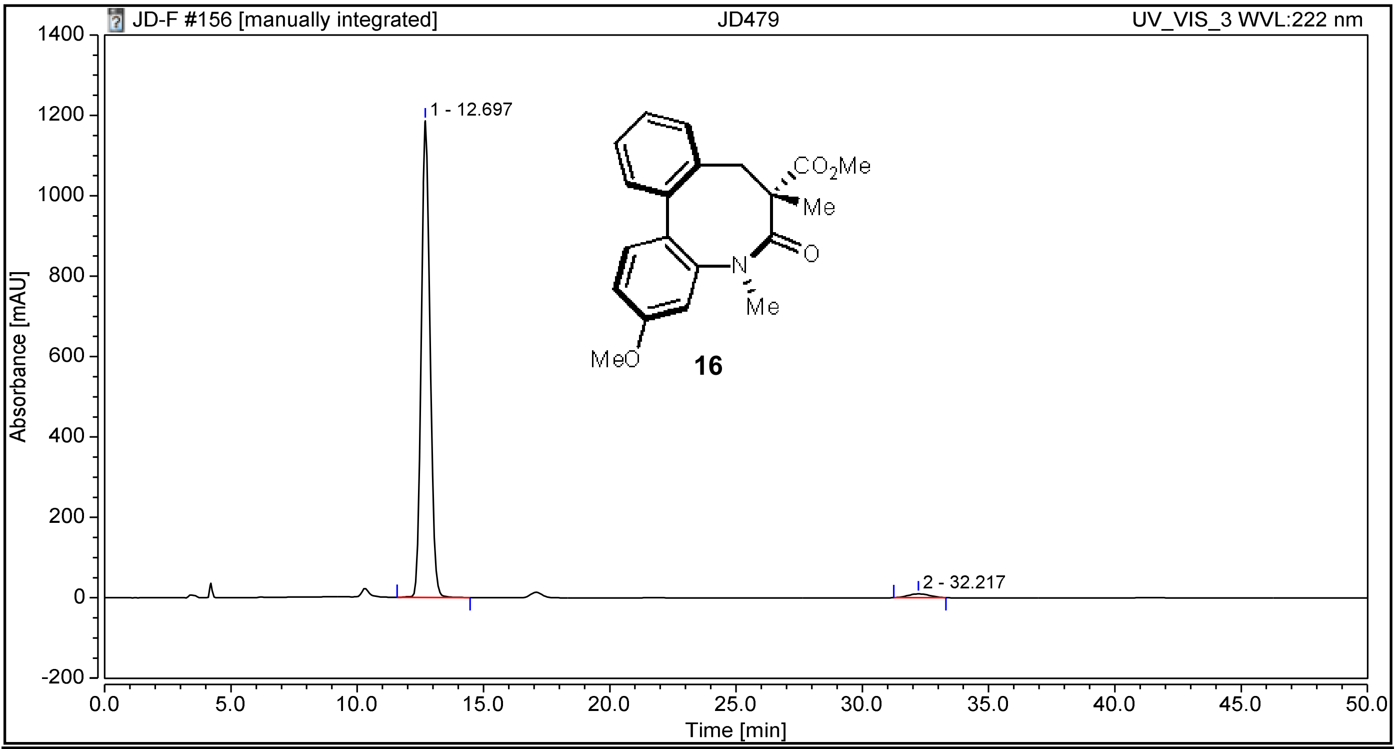

Integration Results

No.	Peak Name	Retention Time min	Area mAU*min	Height mAU	Relative Area %	Relative Height %	Amount n.a.
1		12.697	475.012	1185.172	98.07	99.21	n.a.
2		32.217	9.370	9.457	1.93	0.79	n.a.
**Total:**			**484.382**	**1194.629**	**100.00**	**100.00**	

Chromatogram and Results

Injection Details

Injection Name:	**JD482(rac)**	*Run Time (min):*	**50.00**
Vial Number:	**BE3**	*Injection Volume:*	**20.00**
Injection Type:	**Unknown**	*Channel:*	**UV_VIS_3**
Calibration Level:		*Wavelength:*	**222**
Instrument Method:	**50% IPA-IC 1mL50min run**	*Bandwidth:*	**4**
Processing Method:	**Standard Processing Method**	*Dilution Factor:*	**1.0000**
Injection Date/Time:	**10/Sep/20 15:54**	*Sample Weight:*	**1.0000**

Chromatogram

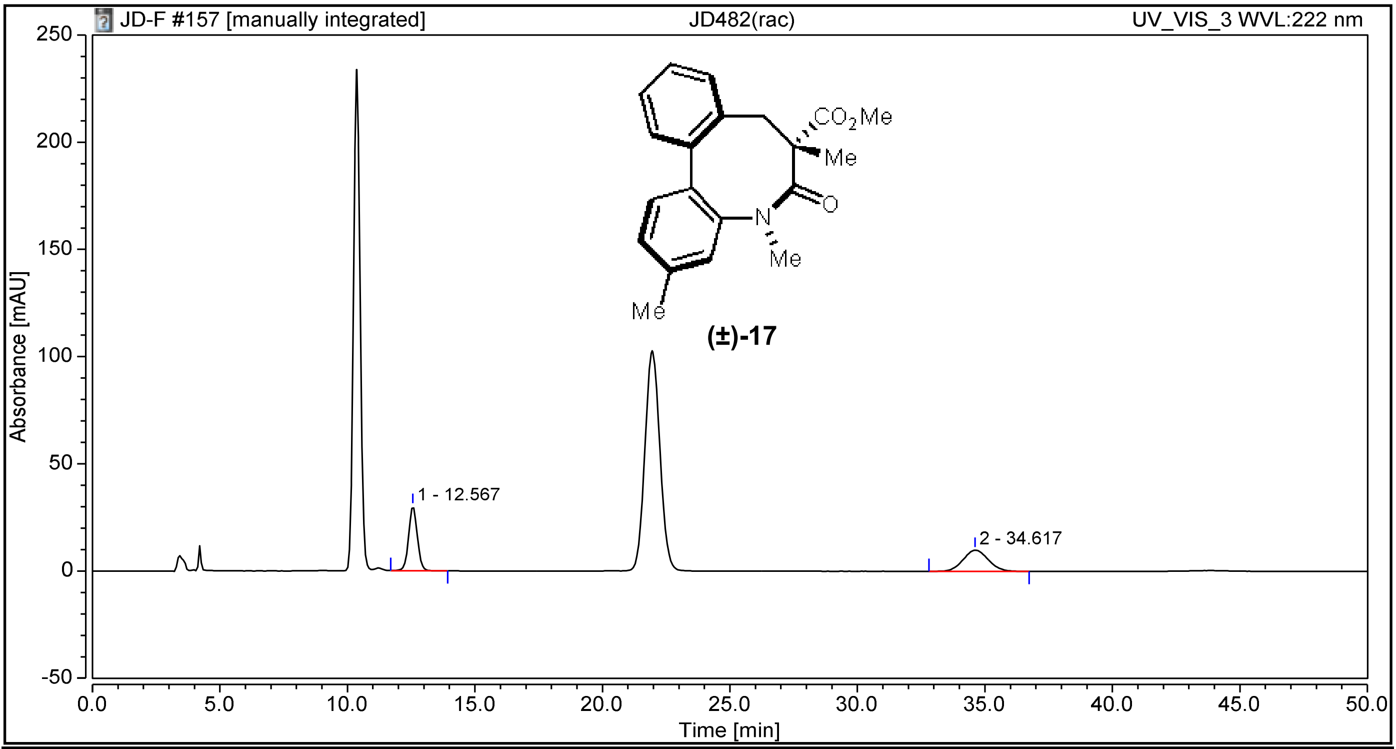

Integration Results

No.	Peak Name	Retention Time min	Area mAU*min	Height mAU	Relative Area %	Relative Height %	Amount n.a.
1		12.567	12.079	30.095	51.59	75.24	n.a.
2		34.617	11.336	9.904	48.41	24.76	n.a.
**Total:**			**23.415**	**39.999**	**100.00**	**100.00**	

Chromatogram and Results

Injection Details

Injection Name:	**JD480**	*Run Time (min):*	**50.00**
Vial Number:	**BE4**	*Injection Volume:*	**20.00**
Injection Type:	**Unknown**	*Channel:*	**UV_VIS_3**
Calibration Level:		*Wavelength:*	**222**
Instrument Method:	**50% IPA-IC 1mL50min run**	*Bandwidth:*	**4**
Processing Method:	**Standard Processing Method**	*Dilution Factor:*	**1.0000**
Injection Date/Time:	**10/Sep/20 16:45**	*Sample Weight:*	**1.0000**

Chromatogram

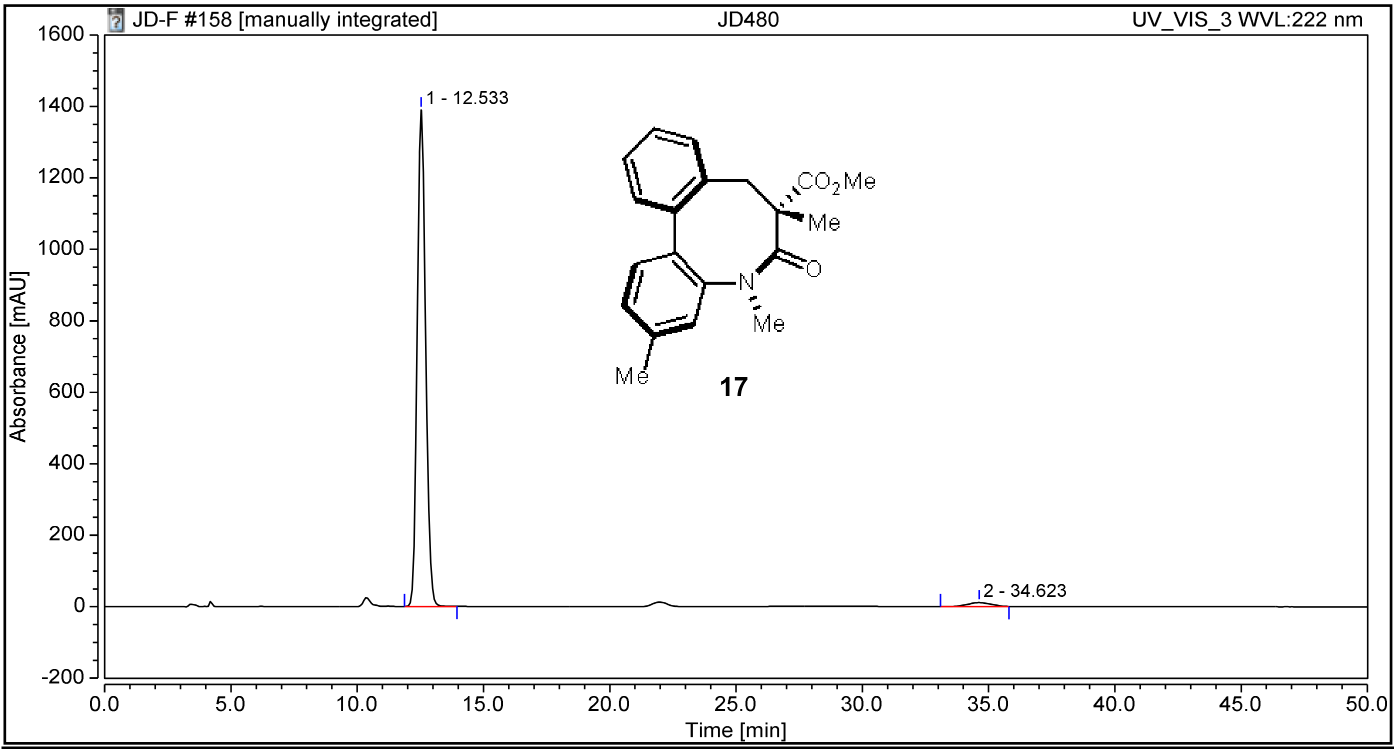

Integration Results

No.	Peak Name	Retention Time min	Area mAU*min	Height mAU	Relative Area %	Relative Height %	Amount n.a.
1		12.533	535.458	1389.794	97.80	99.21	n.a.
2		34.623	12.037	11.132	2.20	0.79	n.a.
**Total:**			**547.495**	**1400.926**	**100.00**	**100.00**	

Chromatogram and Results

Injection Details

Injection Name:	**JD468-s(rac)-s**	*Run Time (min):*	**50.00**
Vial Number:	**BD3**	*Injection Volume:*	**10.00**
Injection Type:	**Unknown**	*Channel:*	**UV_VIS_3**
Calibration Level:		*Wavelength:*	**222**
Instrument Method:	**50% IPA-IC 1mL50min run**	*Bandwidth:*	**4**
Processing Method:	**Standard Processing Method**	*Dilution Factor:*	**1.0000**
Injection Date/Time:	**05/Sep/20 10:36**	*Sample Weight:*	**1.0000**

Chromatogram

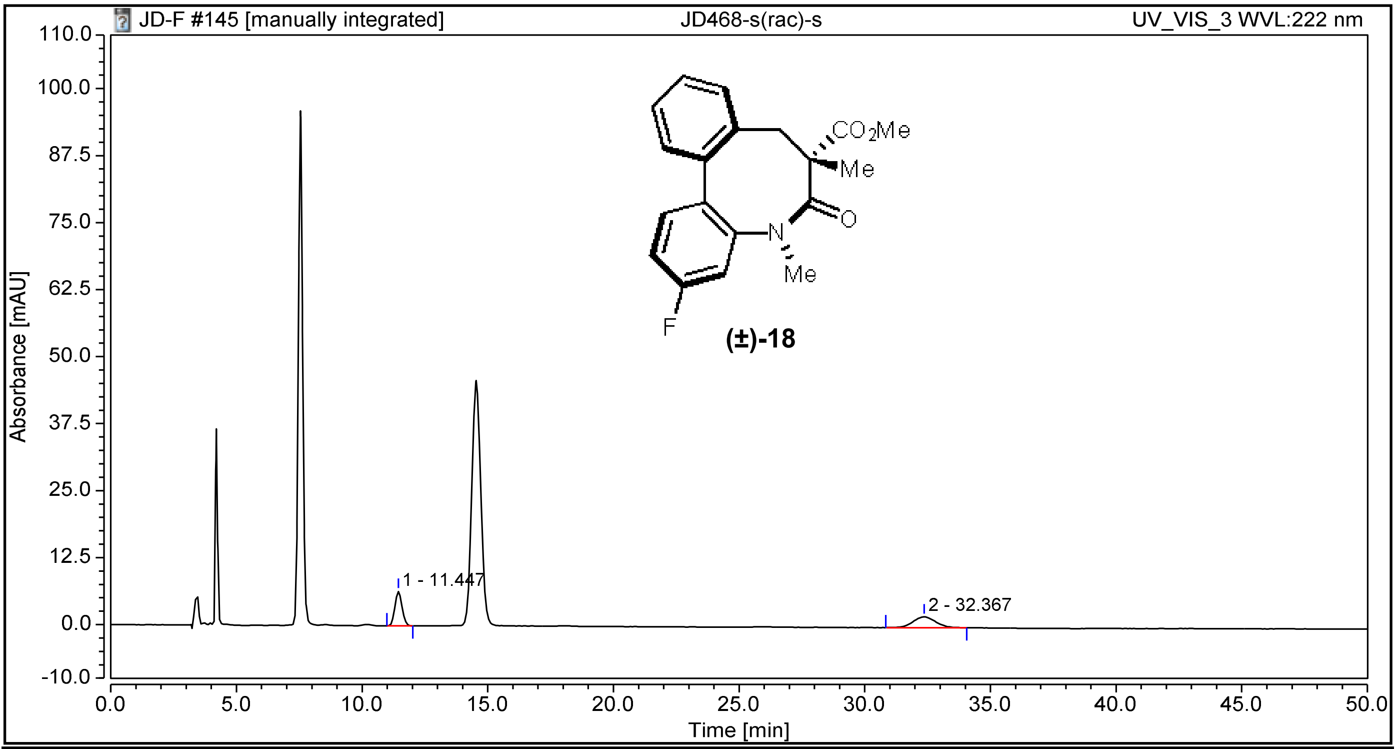

Integration Results

No.	Peak Name	Retention Time min	Area mAU*min	Height mAU	Relative Area %	Relative Height %	Amount n.a.
1		11.447	2.138	6.438	49.76	75.97	n.a.
2		32.367	2.158	2.036	50.24	24.03	n.a.
**Total:**			**4.297**	**8.474**	**100.00**	**100.00**	

Chromatogram and Results

Injection Details

Injection Name:	**JD467-s**	*Run Time (min):*	**50.00**
Vial Number:	**BE8**	*Injection Volume:*	**10.00**
Injection Type:	**Unknown**	*Channel:*	**UV_VIS_3**
Calibration Level:		*Wavelength:*	**222**
Instrument Method:	**50% IPA-IC 1mL50min run**	*Bandwidth:*	**4**
Processing Method:	**Standard Processing Method**	*Dilution Factor:*	**1.0000**
Injection Date/Time:	**05/Sep/20 11:27**	*Sample Weight:*	**1.0000**

Chromatogram

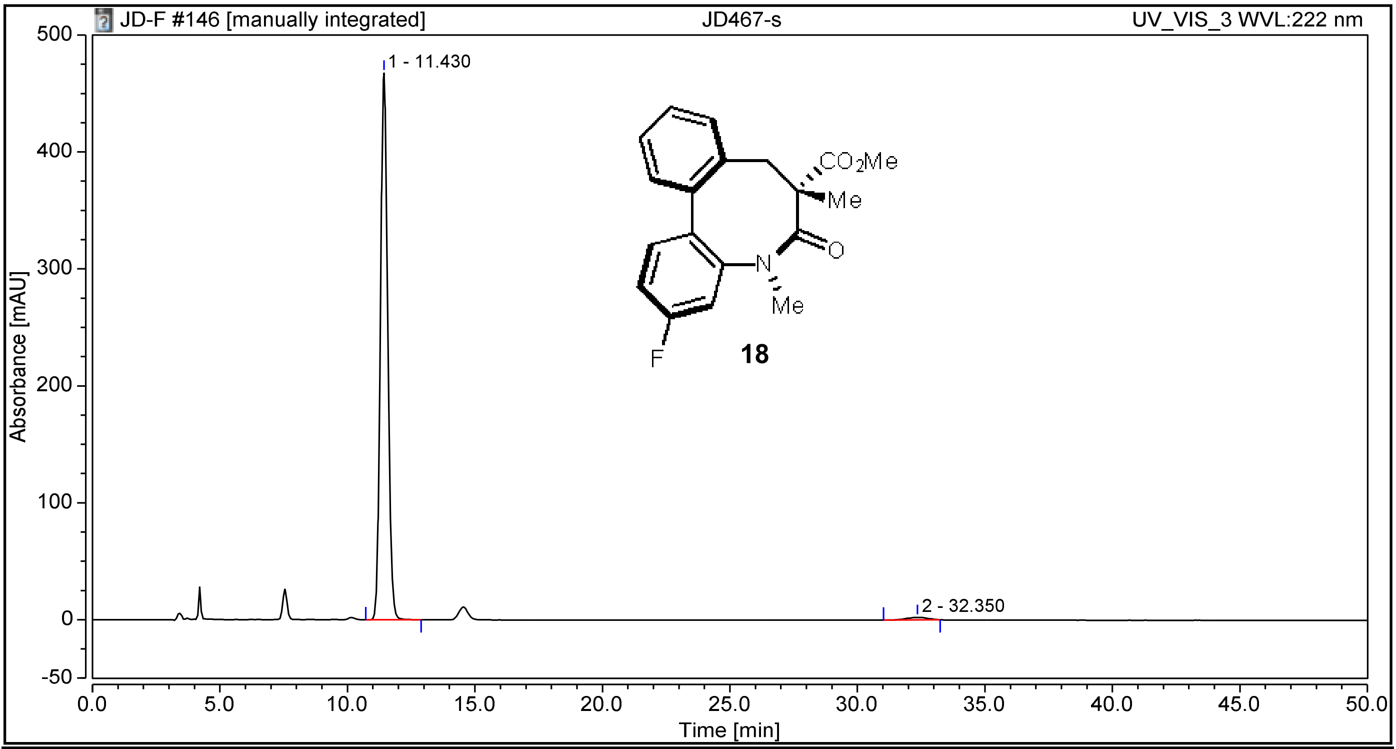

Integration Results

No.	Peak Name	Retention Time min	Area mAU*min	Height mAU	Relative Area %	Relative Height %	Amount n.a.
1		11.430	155.616	467.461	98.70	99.53	n.a.
2		32.350	2.056	2.188	1.30	0.47	n.a.
**Total:**			**157.673**	**469.649**	**100.00**	**100.00**	

Chromatogram and Results

Injection Details

Injection Name:	**JD365(rac)**	*Run Time (min):*	**70.00**
Vial Number:	**GE1**	*Injection Volume:*	**20.00**
Injection Type:	**Unknown**	*Channel:*	**UV_VIS_3**
Calibration Level:		*Wavelength:*	**222**
Instrument Method:	**3% ADH-pre-equ-70min run**	*Bandwidth:*	**4**
Processing Method:	**Standard Processing Method**	*Dilution Factor:*	**1.0000**
Injection Date/Time:	**10/Jul/20 16:53**	*Sample Weight:*	**1.0000**

Chromatogram

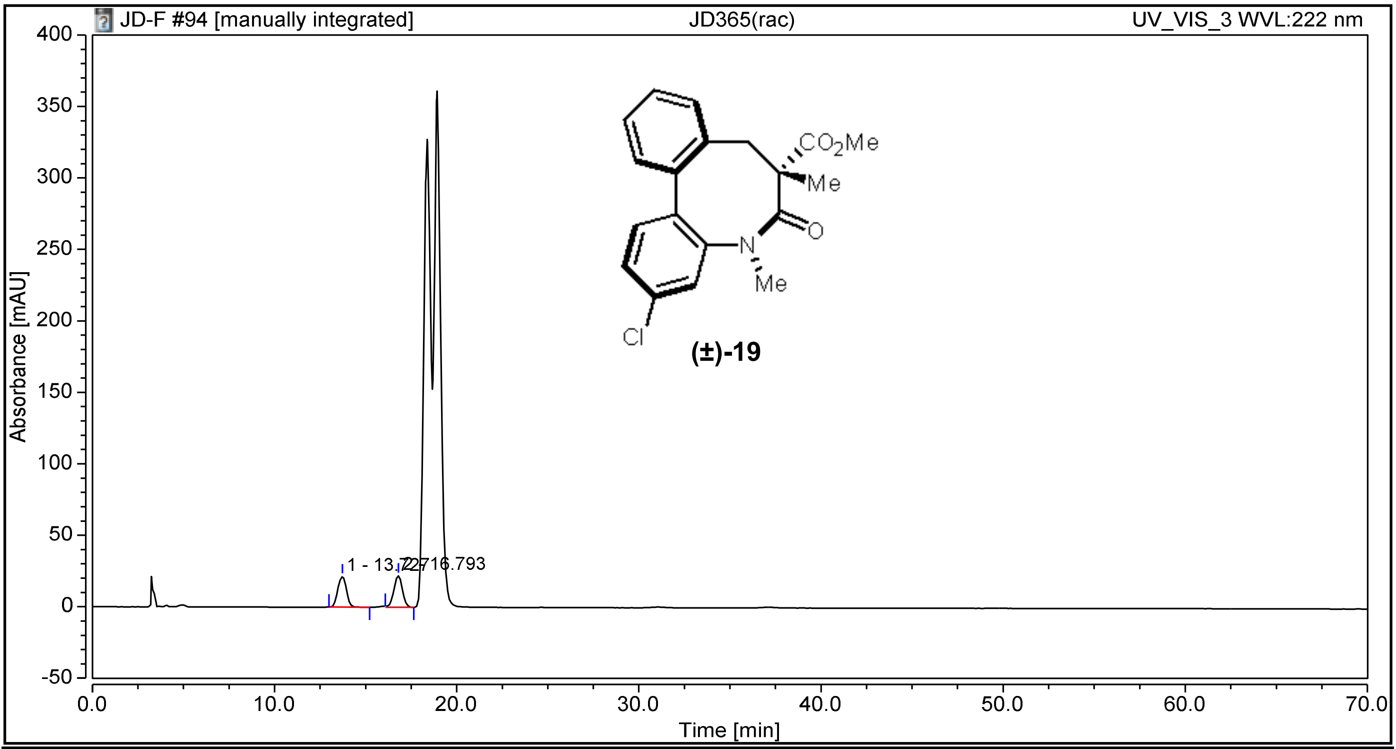

Integration Results

No.	Peak Name	Retention Time min	Area mAU*min	Height mAU	Relative Area %	Relative Height %	Amount n.a.
1		13.727	11.849	21.334	49.48	49.25	n.a.
2		16.793	12.100	21.987	50.52	50.75	n.a.
**Total:**			**23.948**	**43.321**	**100.00**	**100.00**	

Chromatogram and Results

Injection Details

Injection Name:	**JD364**	*Run Time (min):*	**40.00**
Vial Number:	**GE5**	*Injection Volume:*	**20.00**
Injection Type:	**Unknown**	*Channel:*	**UV_VIS_3**
Calibration Level:		*Wavelength:*	**222**
Instrument Method:	**3% ADH 40min run**	*Bandwidth:*	**4**
Processing Method:	**Standard Processing Method**	*Dilution Factor:*	**1.0000**
Injection Date/Time:	**10/Jul/20 19:26**	*Sample Weight:*	**1.0000**

Chromatogram

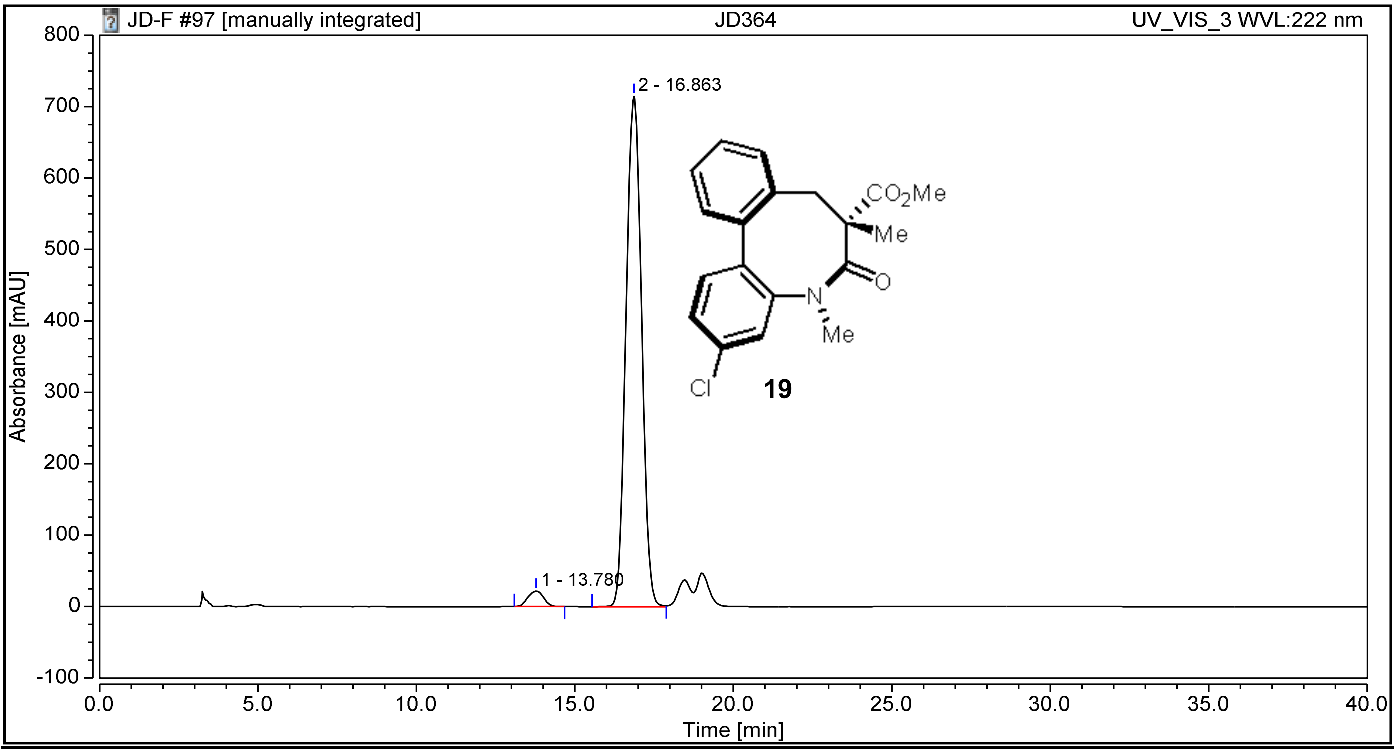

Integration Results

No.	Peak Name	Retention Time<br>min	Area<br>mAU*min	Height<br>mAU	Relative Area<br>%	Relative Height<br>%	Amount<br>n.a.
1		13.780	12.387	21.519	2.96	2.92	n.a.
2		16.863	406.640	714.753	97.04	97.08	n.a.
**Total:**			**419.027**	**736.271**	**100.00**	**100.00**	

Chromatogram and Results

Injection Details

Injection Name:	**JD500(rac)**	*Run Time (min):*	**33.00**
Vial Number:	**BE1**	*Injection Volume:*	**20.00**
Injection Type:	**Unknown**	*Channel:*	**UV_VIS_3**
Calibration Level:		*Wavelength:*	**222**
Instrument Method:	**50%IPA -IC 1mL 33 min run**	*Bandwidth:*	**4**
Processing Method:	**Standard Processing Method**	*Dilution Factor:*	**1.0000**
Injection Date/Time:	**25/Sep/20 17:45**	*Sample Weight:*	**1.0000**

Chromatogram

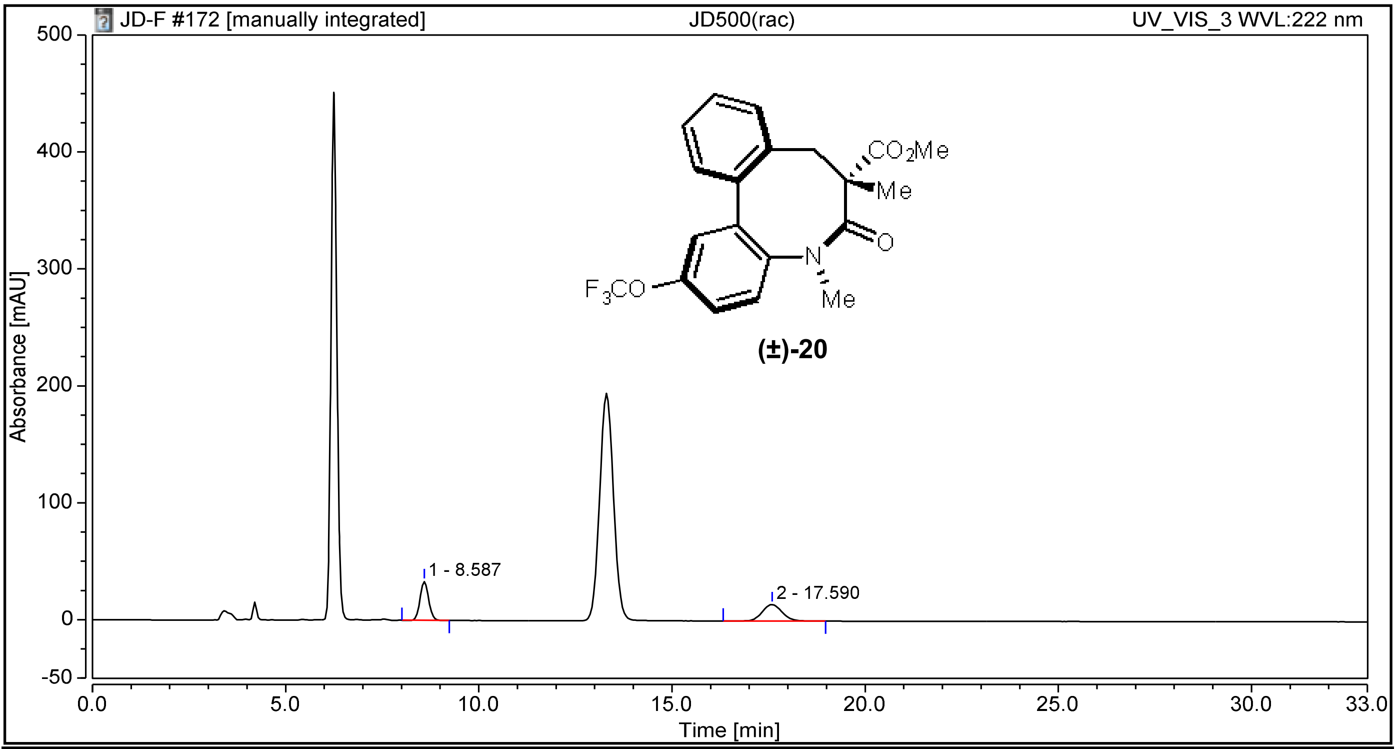

Integration Results

No.	Peak Name	Retention Time<br>min	Area<br>mAU*min	Height<br>mAU	Relative Area<br>%	Relative Height<br>%	Amount<br>n.a.
1		8.587	8.587	33.201	50.75	70.19	n.a.
2		17.590	8.334	14.101	49.25	29.81	n.a.
**Total:**			**16.920**	**47.302**	**100.00**	**100.00**	

Chromatogram and Results

Injection Details

Injection Name:	**JD499**	*Run Time (min):*	**33.00**
Vial Number:	**BE2**	*Injection Volume:*	**20.00**
Injection Type:	**Unknown**	*Channel:*	**UV_VIS_3**
Calibration Level:		*Wavelength:*	**222**
Instrument Method:	**50%IPA -IC 1mL 33 min run**	*Bandwidth:*	**4**
Processing Method:	**Standard Processing Method**	*Dilution Factor:*	**1.0000**
Injection Date/Time:	**24/Sep/20 12:58**	*Sample Weight:*	**1.0000**

Chromatogram

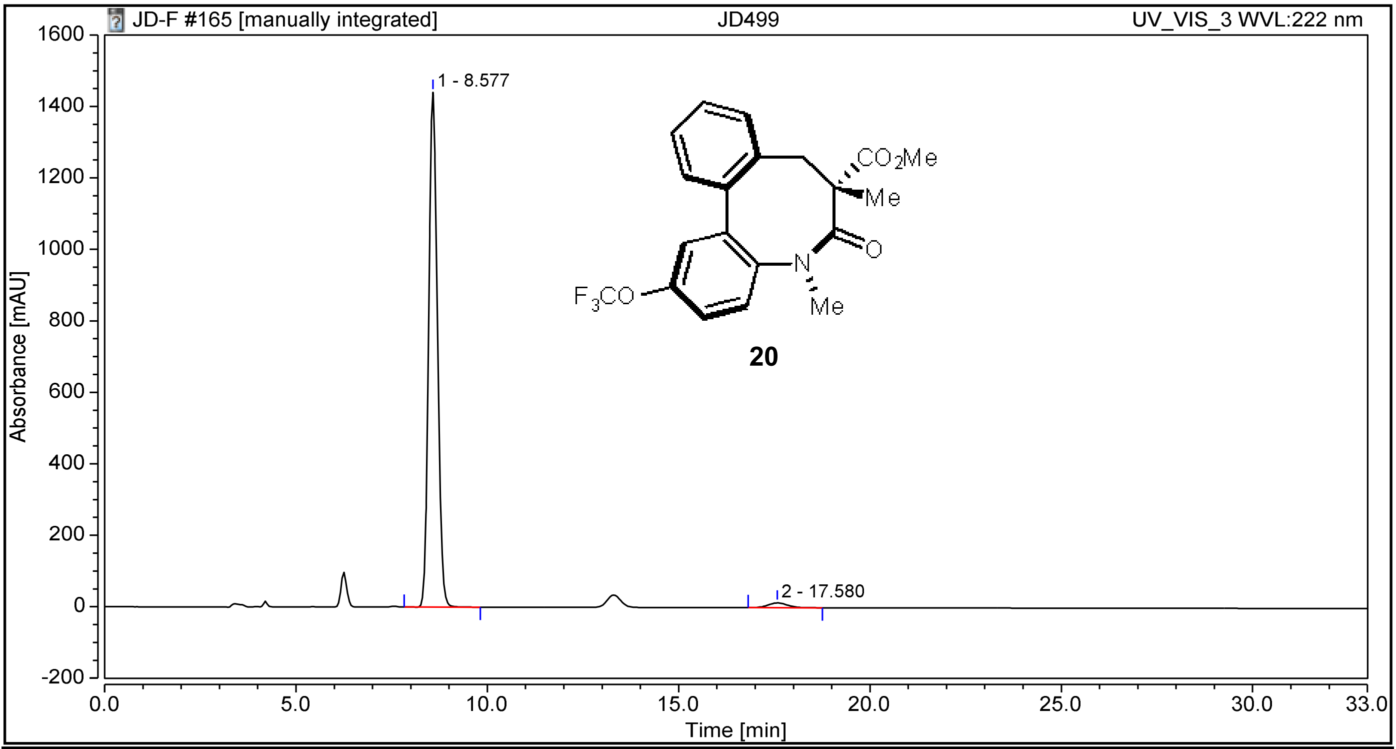

Integration Results

No.	Peak Name	Retention Time min	Area mAU*min	Height mAU	Relative Area %	Relative Height %	Amount n.a.
1		8.577	380.986	1440.610	98.00	99.08	n.a.
2		17.580	7.785	13.310	2.00	0.92	n.a.
**Total:**			**388.771**	**1453.920**	**100.00**	**100.00**	

Chromatogram and Results

Injection Details

Injection Name:	**JD322(S)**	*Run Time (min):*	**33.00**
Vial Number:	**GE3**	*Injection Volume:*	**20.00**
Injection Type:	**Unknown**	*Channel:*	**UV_VIS_3**
Calibration Level:		*Wavelength:*	**222**
Instrument Method:	**50% IC-33min run**	*Bandwidth:*	**4**
Processing Method:	**Standard Processing Method**	*Dilution Factor:*	**1.0000**
Injection Date/Time:	**07/Jul/20 12:26**	*Sample Weight:*	**1.0000**

Chromatogram

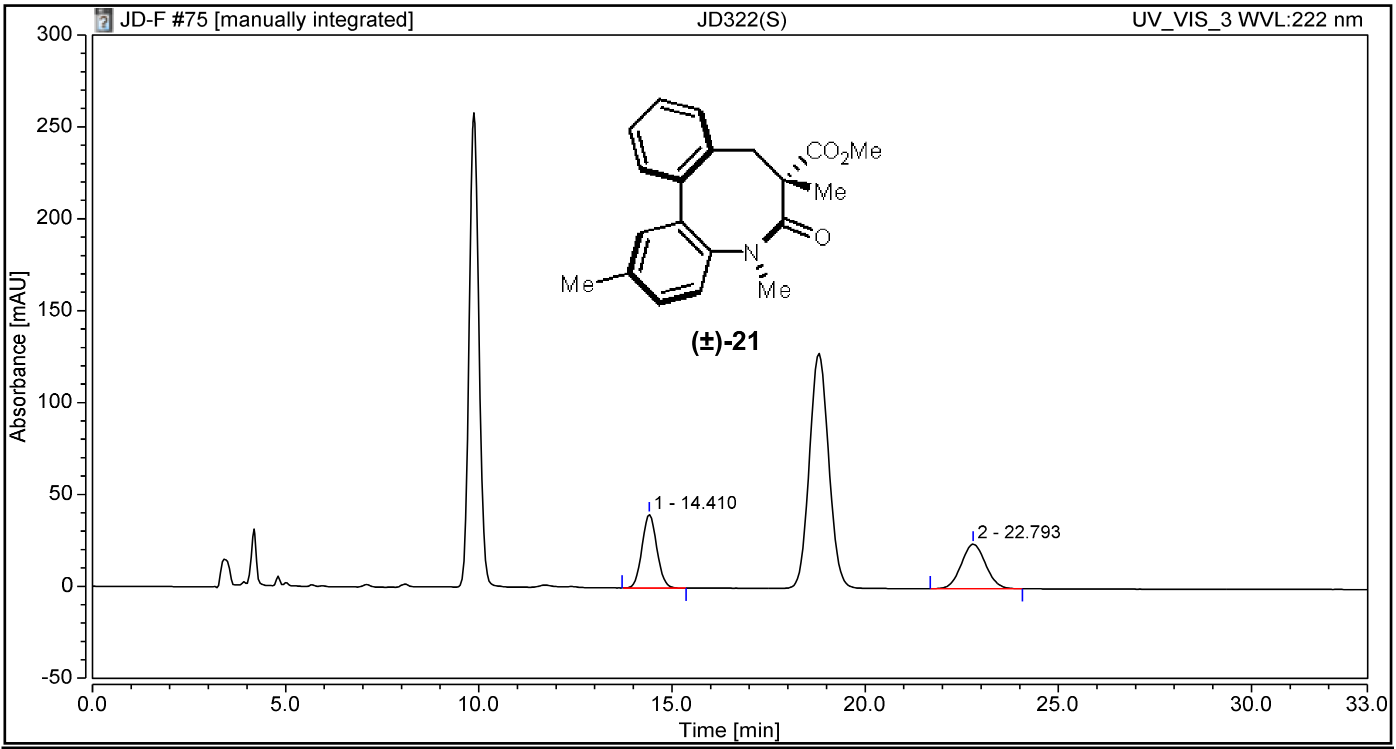

Integration Results

No.	Peak Name	Retention Time<br>min	Area<br>mAU*min	Height<br>mAU	Relative Area<br>%	Relative Height<br>%	Amount<br>n.a.
1		14.410	17.764	39.905	50.18	62.23	n.a.
2		22.793	17.635	24.219	49.82	37.77	n.a.
**Total:**			**35.399**	**64.124**	**100.00**	**100.00**	

Chromatogram and Results

Injection Details

Injection Name:	**JD363**	*Run Time (min):*	**40.00**
Vial Number:	**GE2**	*Injection Volume:*	**20.00**
Injection Type:	**Unknown**	*Channel:*	**UV_VIS_3**
Calibration Level:		*Wavelength:*	**222**
Instrument Method:	**50% IC-pre-equ-40min run**	*Bandwidth:*	**4**
Processing Method:	**Standard Processing Method**	*Dilution Factor:*	**1.0000**
Injection Date/Time:	**07/Jul/20 11:44**	*Sample Weight:*	**1.0000**

Chromatogram

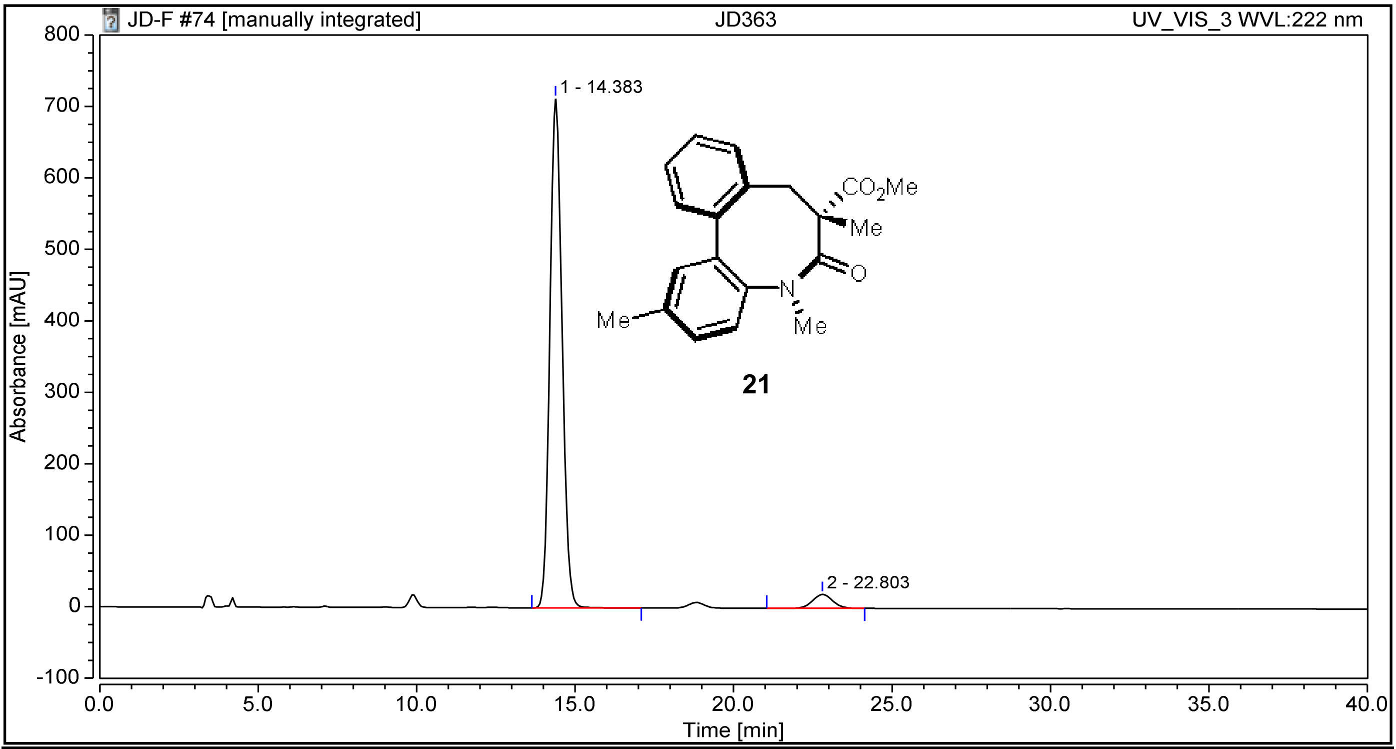

Integration Results

No.	Peak Name	Retention Time<br>min	Area<br>mAU*min	Height<br>mAU	Relative Area<br>%	Relative Height<br>%	Amount<br>n.a.
1		14.383	317.770	712.391	95.70	97.35	n.a.
2		22.803	14.270	19.402	4.30	2.65	n.a.
**Total:**			**332.040**	**731.793**	**100.00**	**100.00**	

Chromatogram and Results

Injection Details

Injection Name:	**JD386(rac)**	*Run Time (min):*	**33.00**
Vial Number:	**GE3**	*Injection Volume:*	**20.00**
Injection Type:	**Unknown**	*Channel:*	**UV_VIS_3**
Calibration Level:		*Wavelength:*	**222**
Instrument Method:	**50% IC-33min run**	*Bandwidth:*	**4**
Processing Method:	**Standard Processing Method**	*Dilution Factor:*	**1.0000**
Injection Date/Time:	**21/Jul/20 14:02**	*Sample Weight:*	**1.0000**

Chromatogram

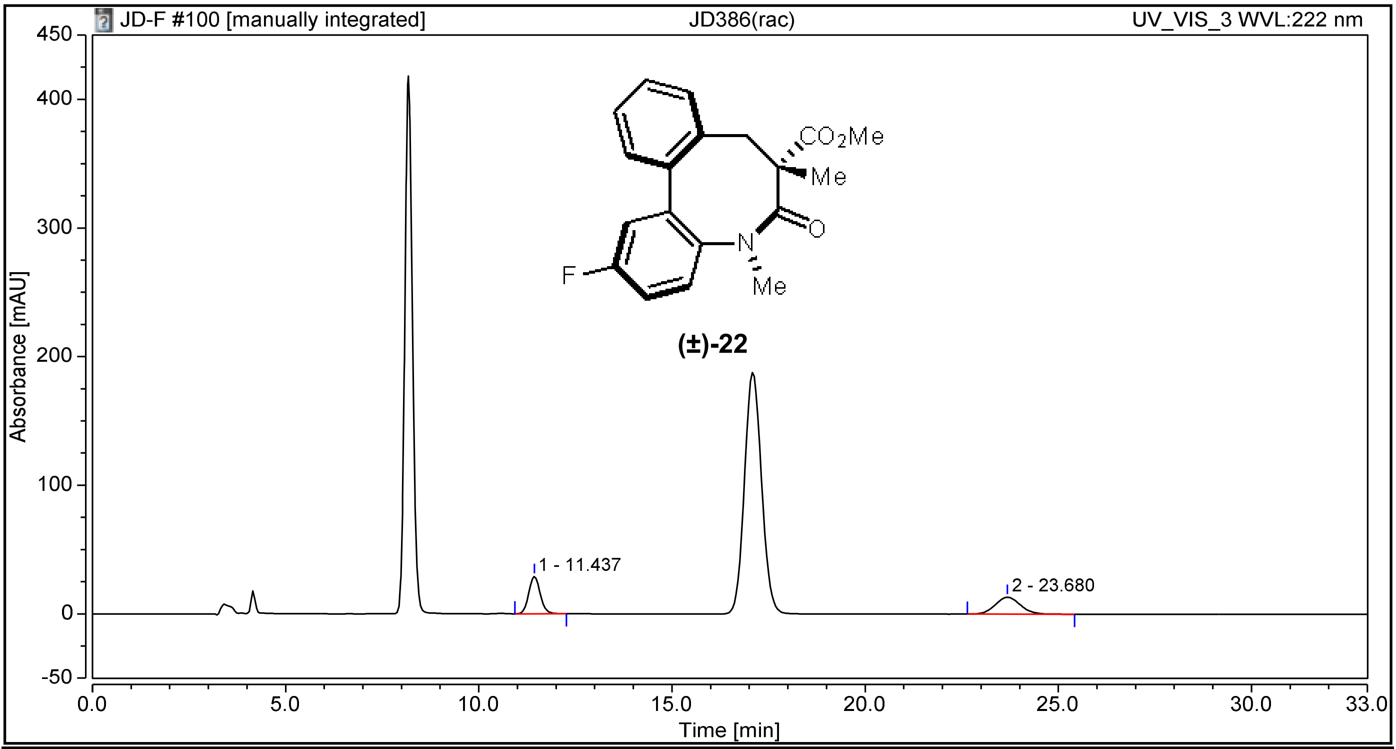

(±)-22

Integration Results

No.	Peak Name	Retention Time min	Area mAU*min	Height mAU	Relative Area %	Relative Height %	Amount n.a.
1		11.437	9.992	29.110	50.13	68.94	n.a.
2		23.680	9.942	13.118	49.87	31.06	n.a.
**Total:**			**19.934**	**42.228**	**100.00**	**100.00**	

Chromatogram and Results

Injection Details

Injection Name:	**JD385**	*Run Time (min):*	**33.00**
Vial Number:	**GE4**	*Injection Volume:*	**20.00**
Injection Type:	**Unknown**	*Channel:*	**UV_VIS_3**
Calibration Level:		*Wavelength:*	**222**
Instrument Method:	**50% IC-33min run**	*Bandwidth:*	**4**
Processing Method:	**Standard Processing Method**	*Dilution Factor:*	**1.0000**
Injection Date/Time:	**21/Jul/20 14:36**	*Sample Weight:*	**1.0000**

Chromatogram

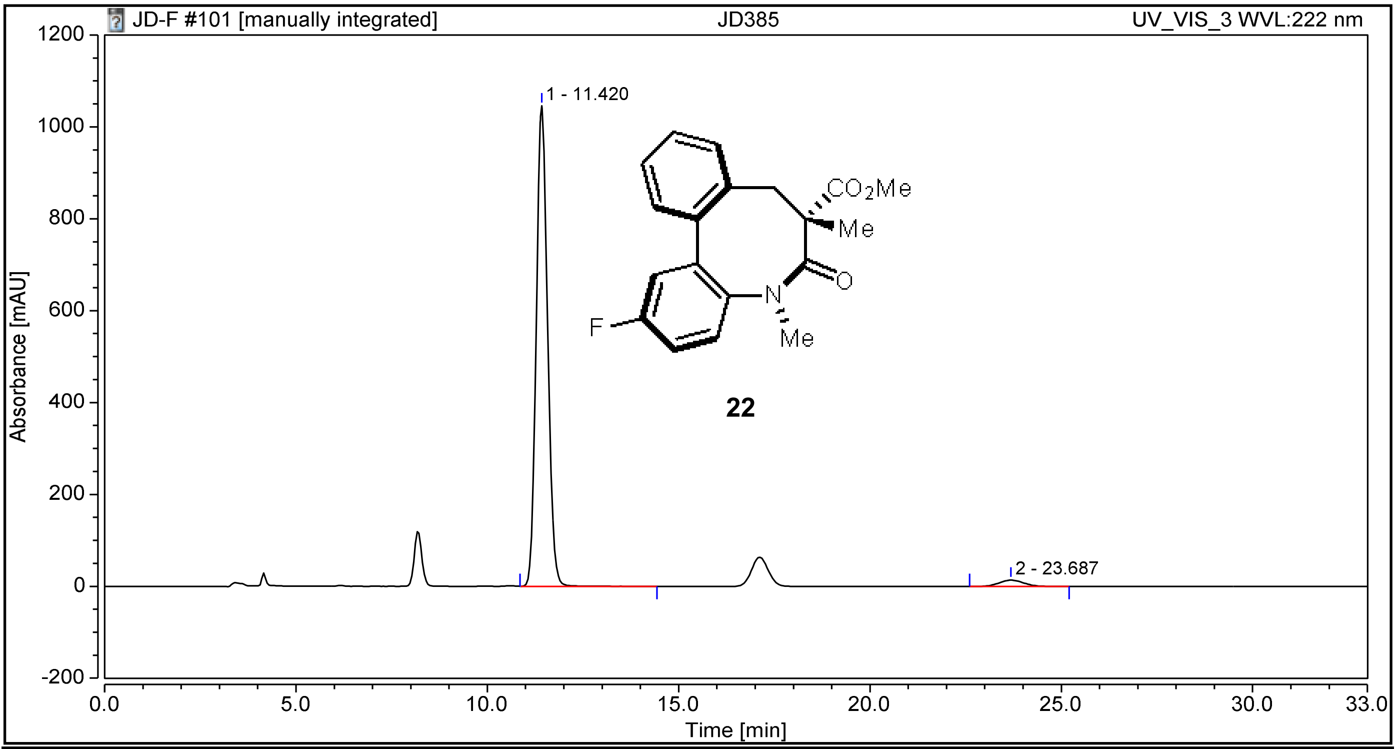

Integration Results

No.	Peak Name	Retention Time min	Area mAU*min	Height mAU	Relative Area %	Relative Height %	Amount n.a.
1		11.420	358.096	1046.303	97.16	98.69	n.a.
2		23.687	10.454	13.845	2.84	1.31	n.a.
**Total:**			**368.550**	**1060.149**	**100.00**	**100.00**	

Chromatogram and Results

Injection Details

Injection Name:	**JD367(rac)**	*Run Time (min):*	**33.00**
Vial Number:	**GE7**	*Injection Volume:*	**20.00**
Injection Type:	**Unknown**	*Channel:*	**UV_VIS_3**
Calibration Level:		*Wavelength:*	**222**
Instrument Method:	**50% IC-33min run**	*Bandwidth:*	**4**
Processing Method:	**Standard Processing Method**	*Dilution Factor:*	**1.0000**
Injection Date/Time:	**07/Jul/20 14:43**	*Sample Weight:*	**1.0000**

Chromatogram

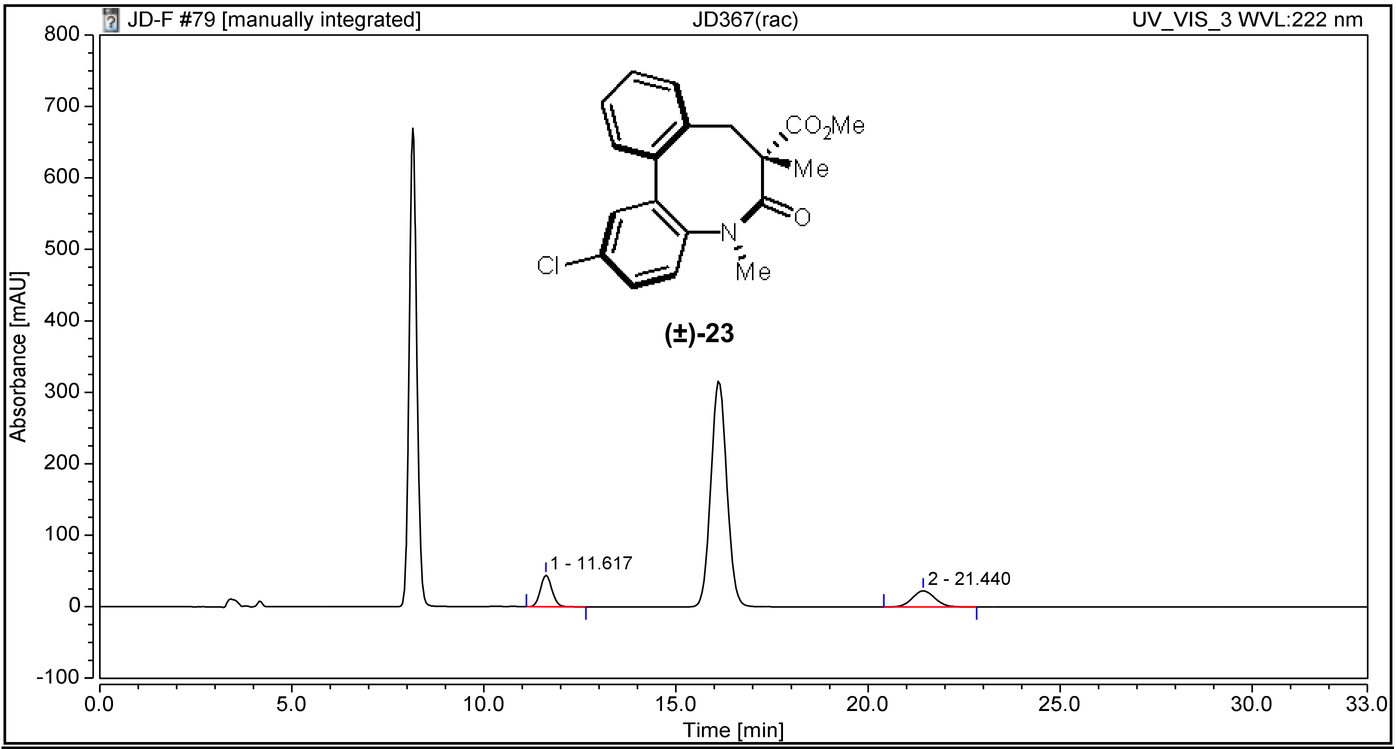

Integration Results

No.	Peak Name	Retention Time min	Area mAU*min	Height mAU	Relative Area %	Relative Height %	Amount n.a.
1		11.617	15.387	44.183	49.96	66.28	n.a.
2		21.440	15.414	22.474	50.04	33.72	n.a.
**Total:**			**30.802**	**66.657**	**100.00**	**100.00**	

Chromatogram and Results

Injection Details

Injection Name:	**JD366**	*Run Time (min):*	**33.00**
Vial Number:	**GE6**	*Injection Volume:*	**20.00**
Injection Type:	**Unknown**	*Channel:*	**UV_VIS_3**
Calibration Level:		*Wavelength:*	**222**
Instrument Method:	**50% IC-33min run**	*Bandwidth:*	**4**
Processing Method:	**Standard Processing Method**	*Dilution Factor:*	**1.0000**
Injection Date/Time:	**07/Jul/20 14:08**	*Sample Weight:*	**1.0000**

Chromatogram

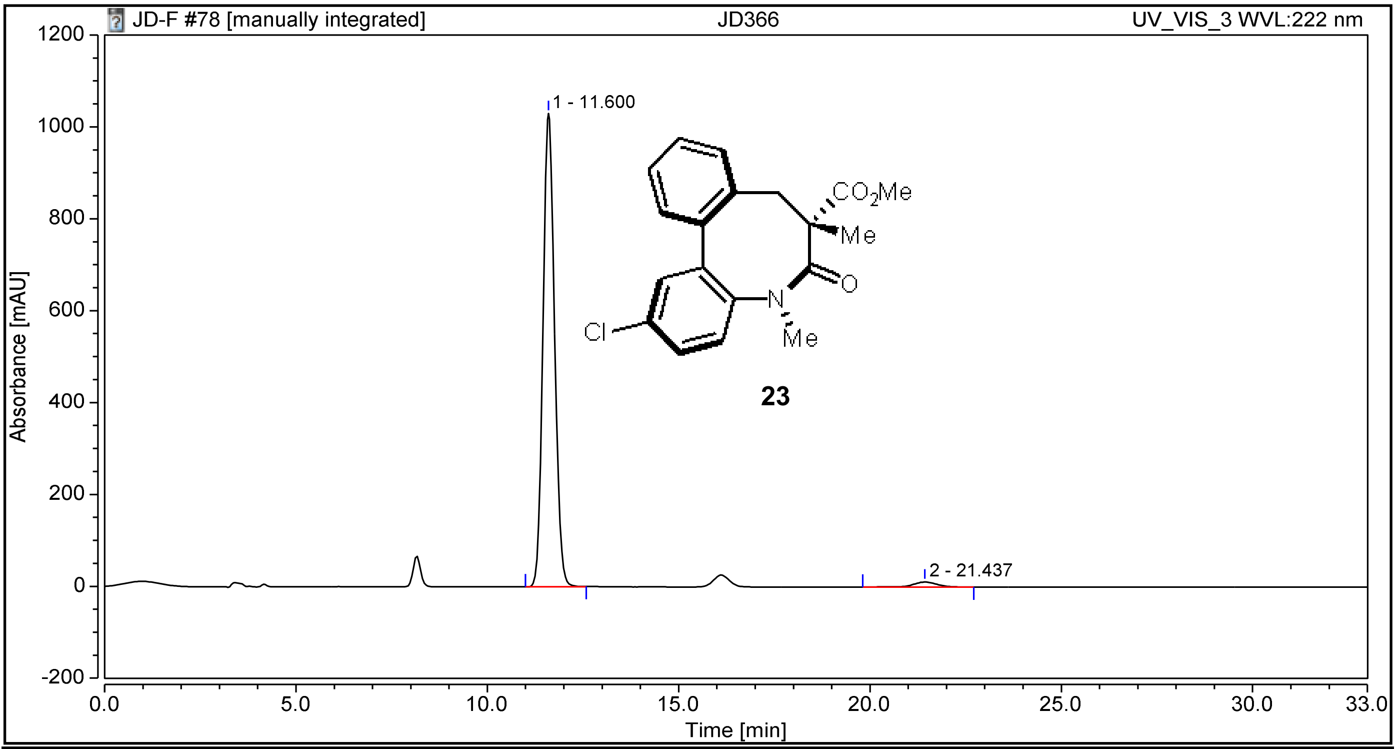

Integration Results

No.	Peak Name	Retention Time min	Area mAU*min	Height mAU	Relative Area %	Relative Height %	Amount n.a.
1		11.600	359.546	1030.221	97.93	98.96	n.a.
2		21.437	7.588	10.874	2.07	1.04	n.a.
**Total:**			**367.134**	**1041.095**	**100.00**	**100.00**	

Chromatogram and Results

Injection Details

Injection Name:	**JD473(rac)-s**	*Run Time (min):*	**50.00**
Vial Number:	**BD4**	*Injection Volume:*	**10.00**
Injection Type:	**Unknown**	*Channel:*	**UV_VIS_3**
Calibration Level:		*Wavelength:*	**222**
Instrument Method:	**50% IPA-IC 1mL50min run**	*Bandwidth:*	**4**
Processing Method:	**Standard Processing Method**	*Dilution Factor:*	**1.0000**
Injection Date/Time:	**05/Sep/20 13:10**	*Sample Weight:*	**1.0000**

Chromatogram

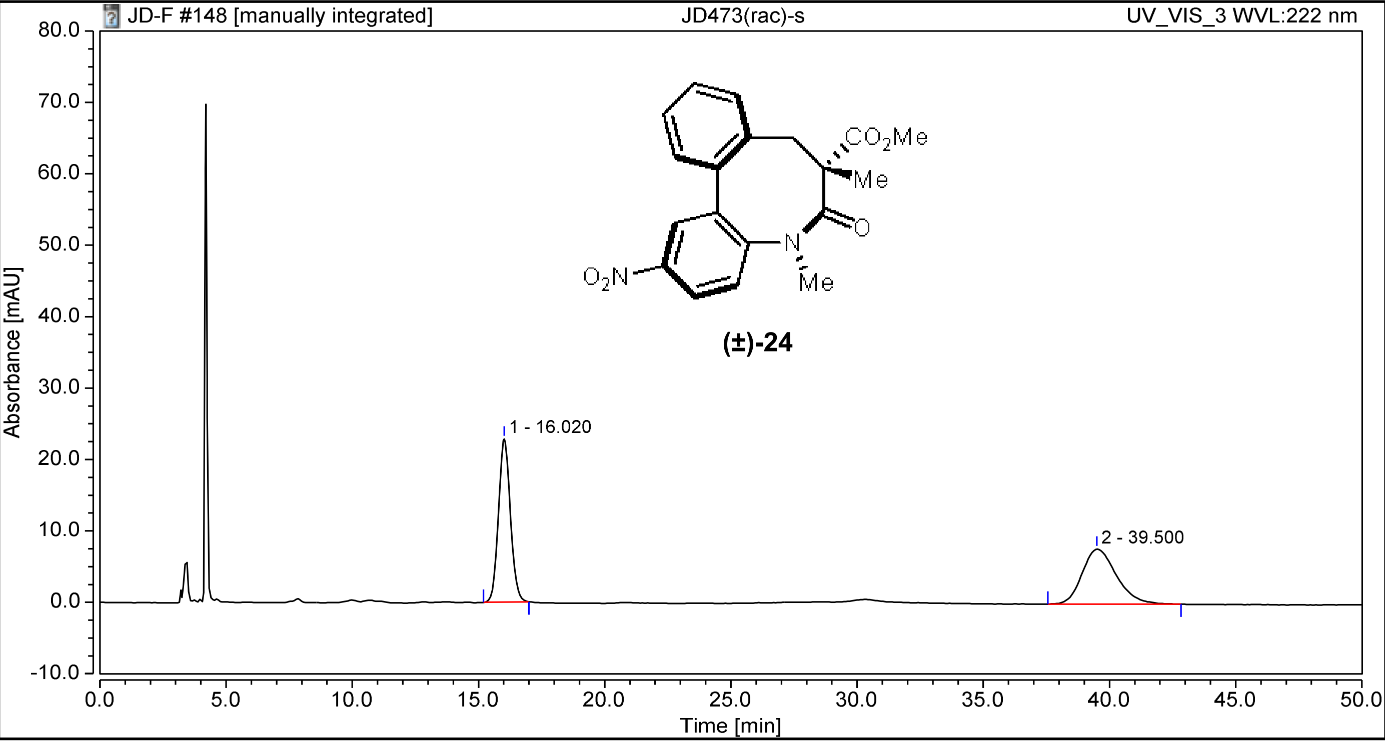

Integration Results

No.	Peak Name	Retention Time min	Area mAU*min	Height mAU	Relative Area %	Relative Height %	Amount n.a.
1		16.020	12.537	22.841	50.79	74.77	n.a.
2		39.500	12.150	7.709	49.21	25.23	n.a.
**Total:**			**24.687**	**30.550**	**100.00**	**100.00**	

Chromatogram and Results

Injection Details

Injection Name:	**JD463**	*Run Time (min):*	**50.00**
Vial Number:	**BE2**	*Injection Volume:*	**10.00**
Injection Type:	**Unknown**	*Channel:*	**UV_VIS_3**
Calibration Level:		*Wavelength:*	**222**
Instrument Method:	**50% IPA-IC 1mL50min run**	*Bandwidth:*	**4**
Processing Method:	**Standard Processing Method**	*Dilution Factor:*	**1.0000**
Injection Date/Time:	**05/Sep/20 14:01**	*Sample Weight:*	**1.0000**

Chromatogram

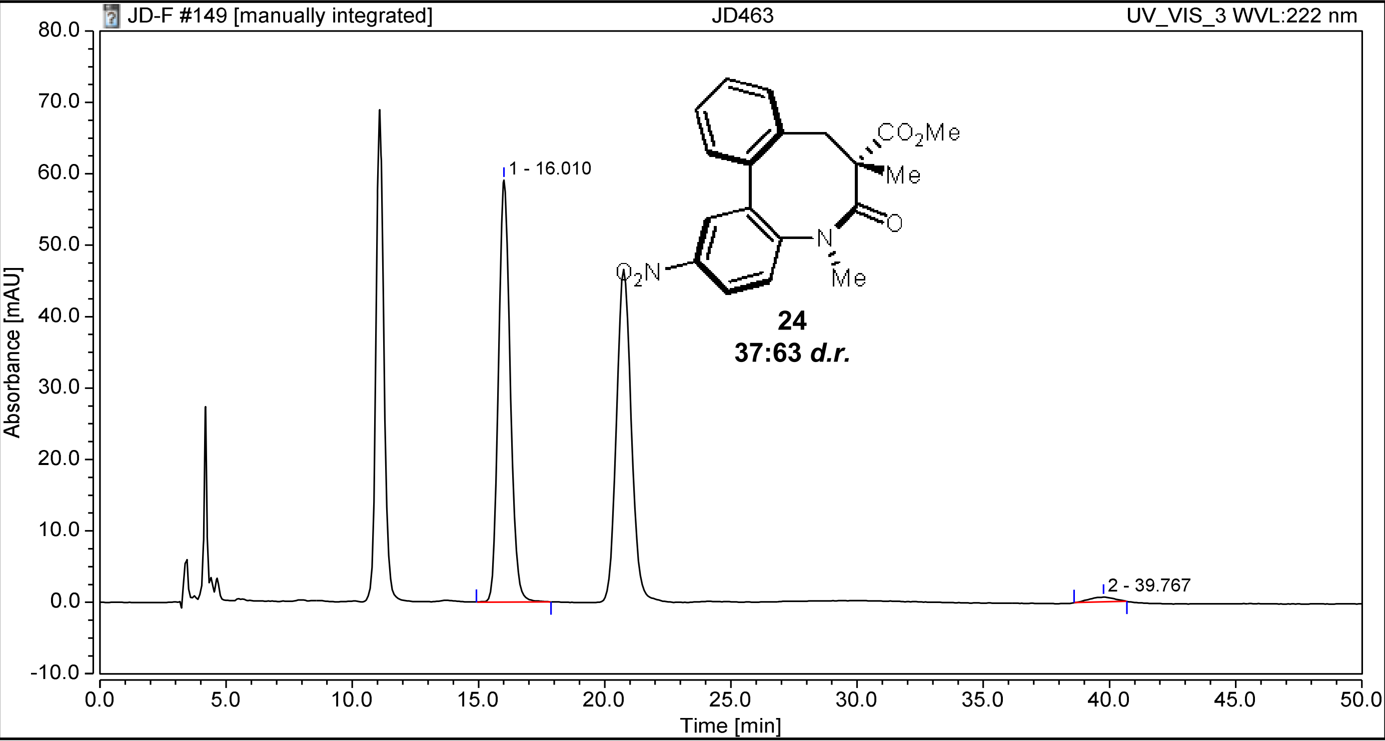

Integration Results

No.	Peak Name	Retention Time min	Area mAU*min	Height mAU	Relative Area %	Relative Height %	Amount n.a.
1		16.010	32.151	59.072	97.63	98.87	n.a.
2		39.767	0.782	0.673	2.37	1.13	n.a.
**Total:**			**32.933**	**59.745**	**100.00**	**100.00**	

Chromatogram and Results

Injection Details

Injection Name:	**JD470(rac)-s**	*Run Time (min):*	**50.00**
Vial Number:	**BD1**	*Injection Volume:*	**10.00**
Injection Type:	**Unknown**	*Channel:*	**UV_VIS_3**
Calibration Level:		*Wavelength:*	**222**
Instrument Method:	**50% IPA-IC 1mL50min run**	*Bandwidth:*	**4**
Processing Method:	**Standard Processing Method**	*Dilution Factor:*	**1.0000**
Injection Date/Time:	**05/Sep/20 09:45**	*Sample Weight:*	**1.0000**

Chromatogram

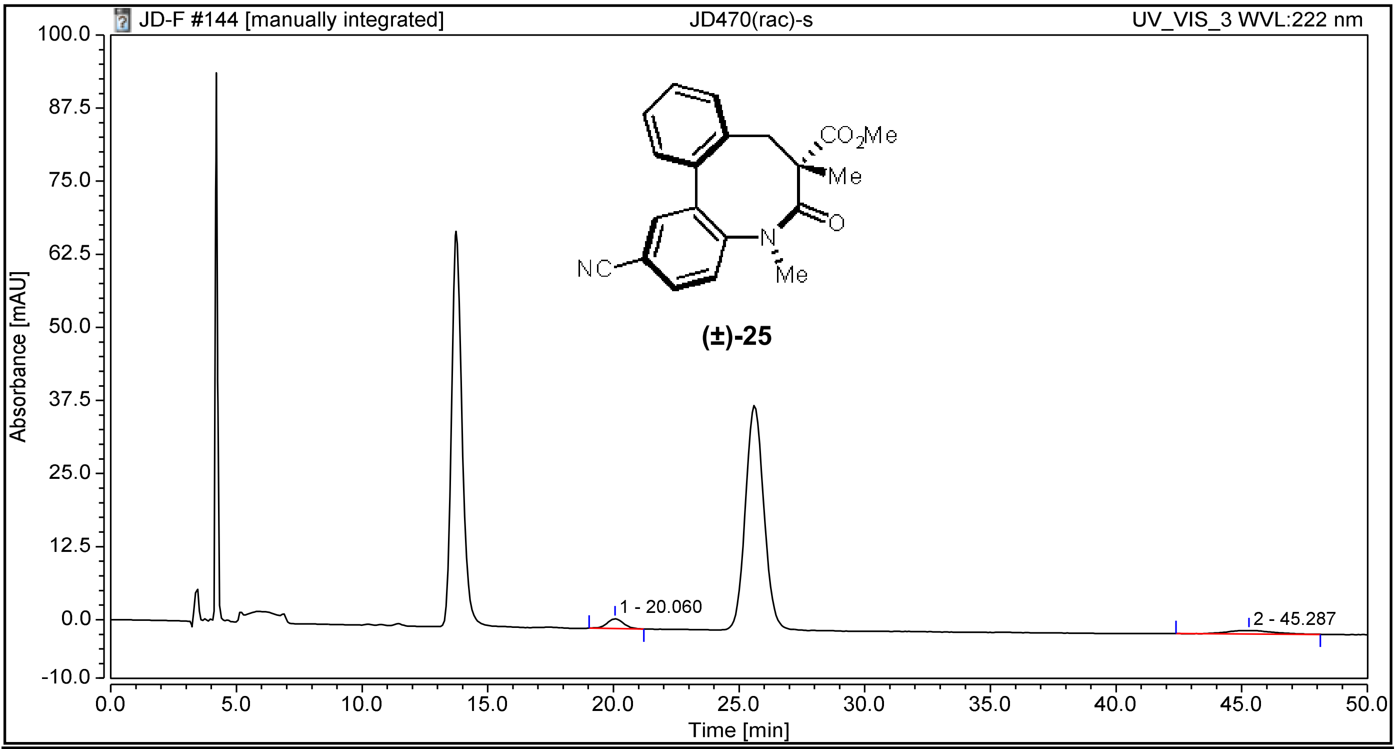

Integration Results

No.	Peak Name	Retention Time min	Area mAU*min	Height mAU	Relative Area %	Relative Height %	Amount n.a.
1		20.060	1.263	1.671	50.60	73.65	n.a.
2		45.287	1.233	0.598	49.40	26.35	n.a.
**Total:**			**2.496**	**2.269**	**100.00**	**100.00**	

Chromatogram and Results

Injection Details

Injection Name:	**JD469-s**	*Run Time (min):*	**50.00**
Vial Number:	**BD2**	*Injection Volume:*	**10.00**
Injection Type:	**Unknown**	*Channel:*	**UV_VIS_3**
Calibration Level:		*Wavelength:*	**222**
Instrument Method:	**50% IPA-IC 1mL50min run**	*Bandwidth:*	**4**
Processing Method:	**Standard Processing Method**	*Dilution Factor:*	**1.0000**
Injection Date/Time:	**05/Sep/20 12:18**	*Sample Weight:*	**1.0000**

Chromatogram

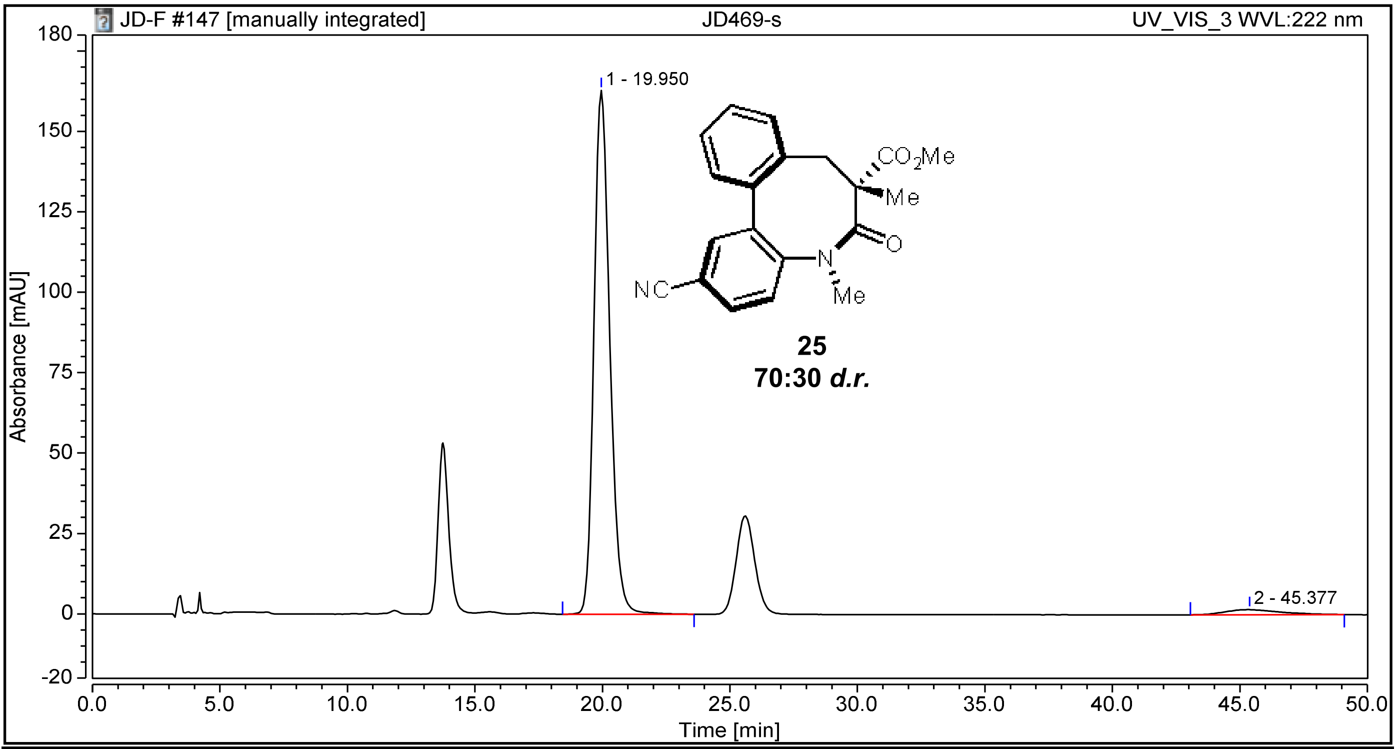

25
70:30 *d.r.*

Integration Results

No.	Peak Name	Retention Time min	Area mAU*min	Height mAU	Relative Area %	Relative Height %	Amount n.a.
1		19.950	117.744	162.858	96.70	99.02	n.a.
2		45.377	4.017	1.609	3.30	0.98	n.a.
**Total:**			**121.761**	**164.467**	**100.00**	**100.00**	

Default DAD/Integration

Chromeleon (c) Dionex
Version 7.2.5.9717

Chromatogram and Results

Injection Details

Injection Name:	**JD455(rac)**	*Run Time (min):*	**40.00**
Vial Number:	**BE3**	*Injection Volume:*	**10.00**
Injection Type:	**Unknown**	*Channel:*	**UV_VIS_3**
Calibration Level:		*Wavelength:*	**222**
Instrument Method:	**IC 50% 0.7mL-40 min run**	*Bandwidth:*	**4**
Processing Method:	**Standard Processing Method**	*Dilution Factor:*	**1.0000**
Injection Date/Time:	**03/Sep/20 10:18**	*Sample Weight:*	**1.0000**

Chromatogram

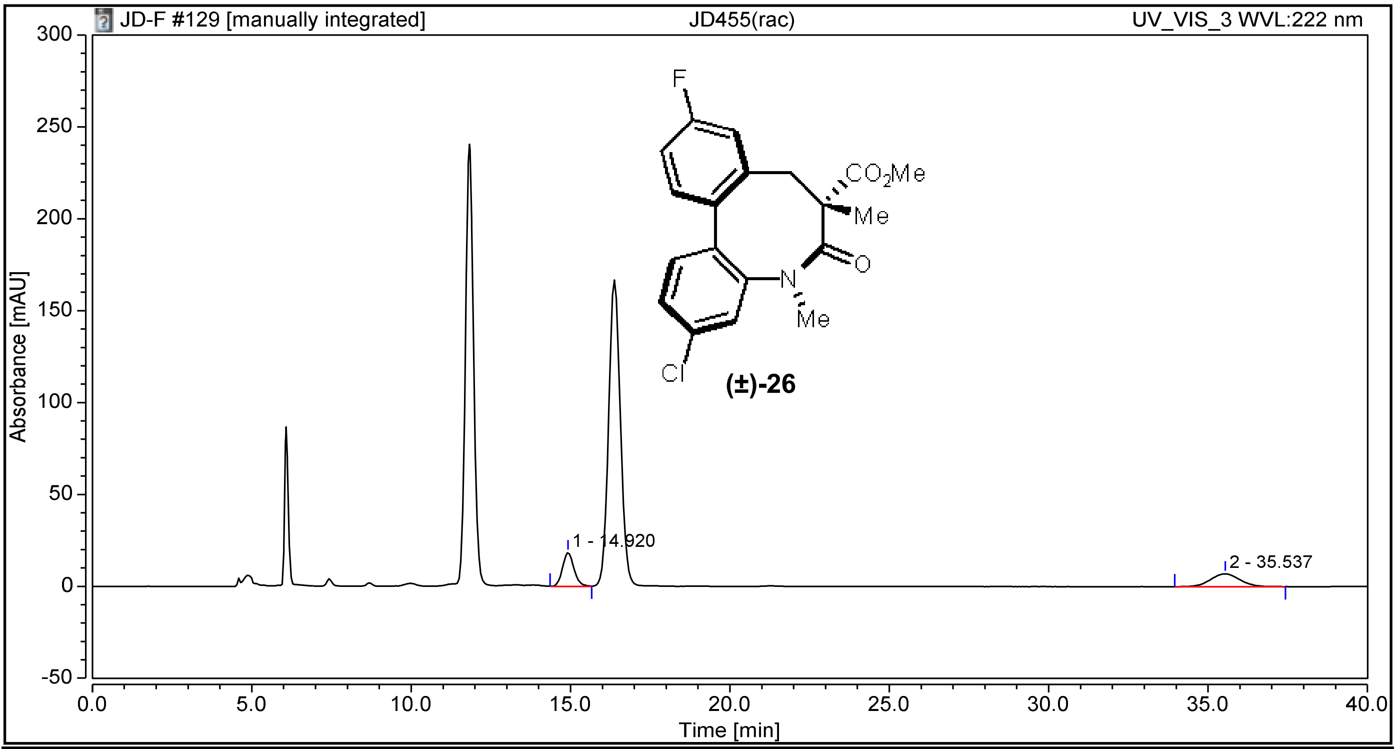

Integration Results

No.	Peak Name	Retention Time min	Area mAU*min	Height mAU	Relative Area %	Relative Height %	Amount n.a.
1		14.920	7.585	18.224	50.58	72.47	n.a.
2		35.537	7.411	6.924	49.42	27.53	n.a.
**Total:**			**14.995**	**25.148**	**100.00**	**100.00**	

Chromatogram and Results

Injection Details

Injection Name:	**JD454**	*Run Time (min):*	**40.00**
Vial Number:	**BE4**	*Injection Volume:*	**10.00**
Injection Type:	**Unknown**	*Channel:*	**UV_VIS_3**
Calibration Level:		*Wavelength:*	**222**
Instrument Method:	**IC 50% 0.7mL-40 min run**	*Bandwidth:*	**4**
Processing Method:	**Standard Processing Method**	*Dilution Factor:*	**1.0000**
Injection Date/Time:	**03/Sep/20 11:41**	*Sample Weight:*	**1.0000**

Chromatogram

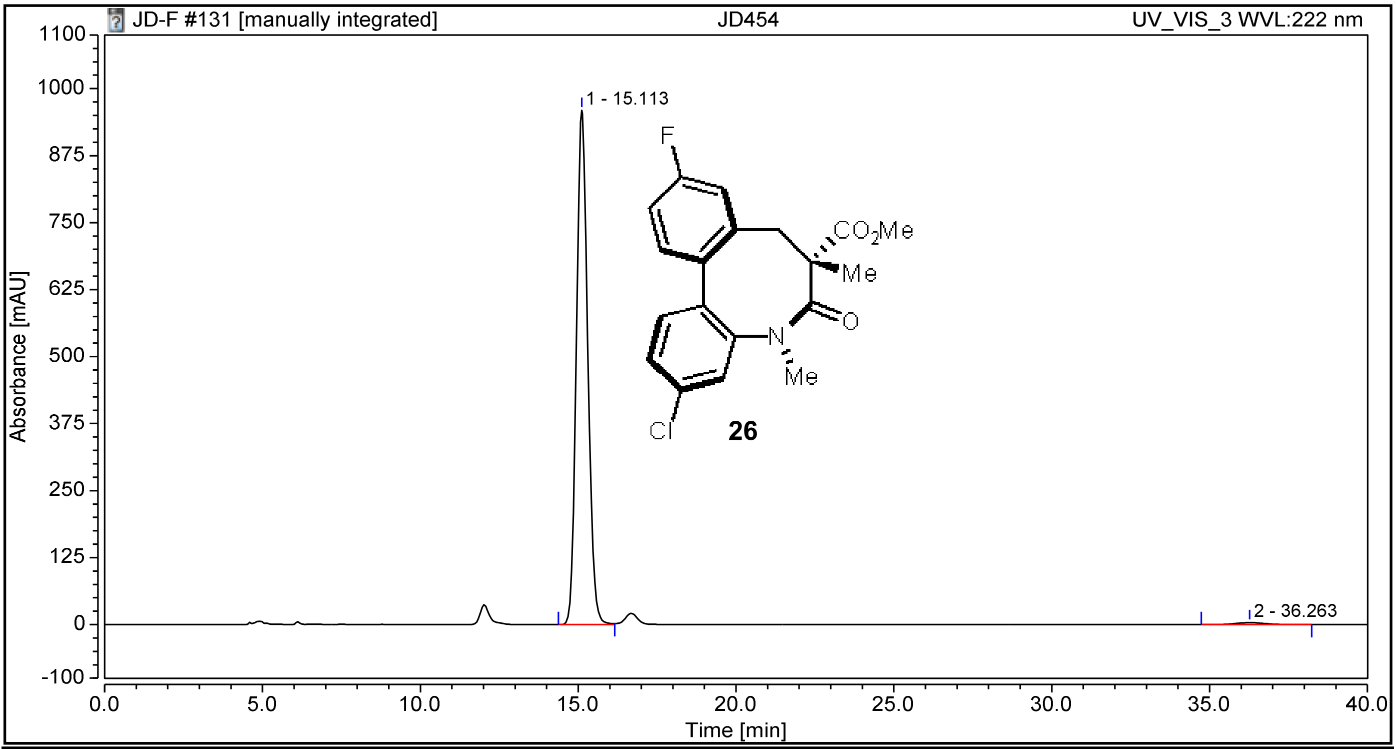

Integration Results

No.	Peak Name	Retention Time min	Area mAU*min	Height mAU	Relative Area %	Relative Height %	Amount n.a.
1		15.113	400.413	959.385	98.98	99.61	n.a.
2		36.263	4.117	3.743	1.02	0.39	n.a.
**Total:**			**404.530**	**963.128**	**100.00**	**100.00**	

Chromatogram and Results

Injection Details

Injection Name:	**JD453(rac)**	*Run Time (min):*	**40.00**
Vial Number:	**BE1**	*Injection Volume:*	**10.00**
Injection Type:	**Unknown**	*Channel:*	**UV_VIS_3**
Calibration Level:		*Wavelength:*	**222**
Instrument Method:	**IC 50% 0.7mL-pre-equ-40 min run**	*Bandwidth:*	**4**
Processing Method:	**Standard Processing Method**	*Dilution Factor:*	**1.0000**
Injection Date/Time:	**03/Sep/20 09:37**	*Sample Weight:*	**1.0000**

Chromatogram

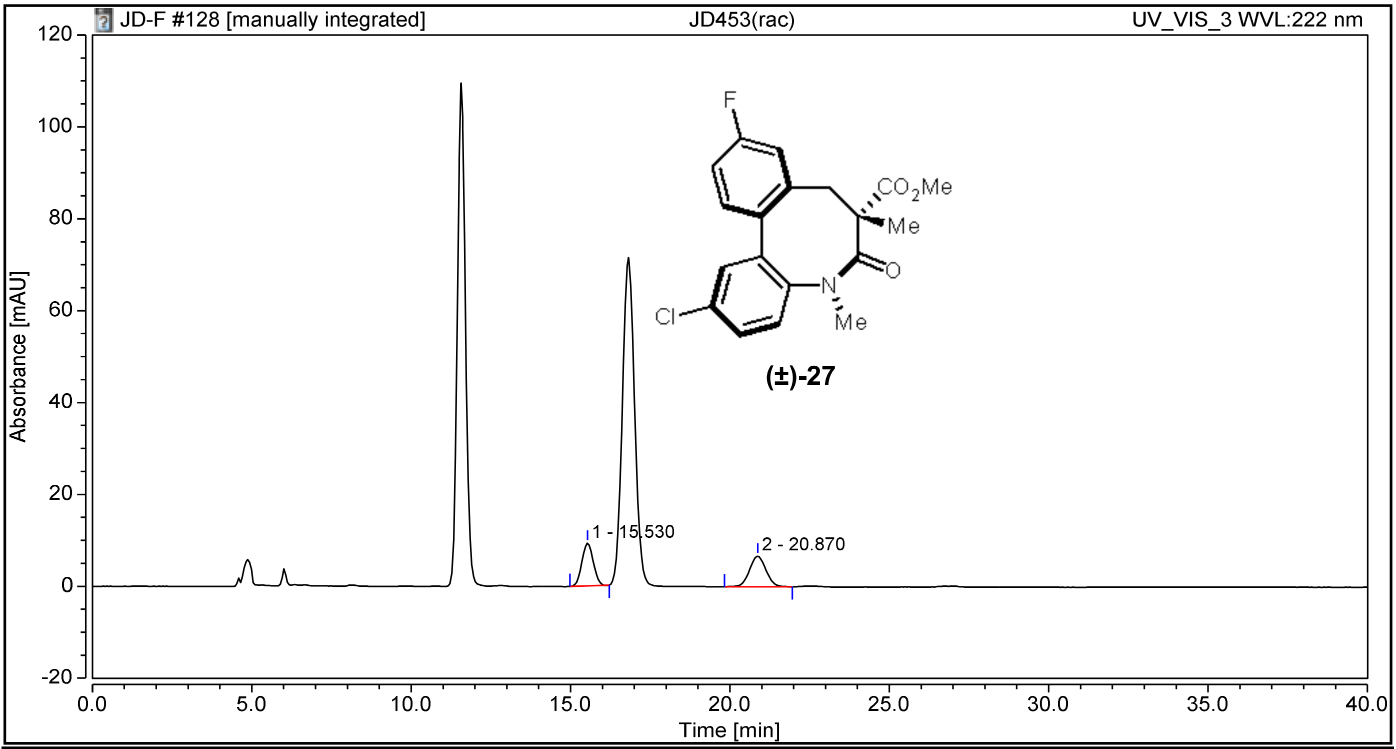

Integration Results

No.	Peak Name	Retention Time min	Area mAU*min	Height mAU	Relative Area %	Relative Height %	Amount n.a.
1		15.530	3.848	9.281	48.94	58.14	n.a.
2		20.870	4.015	6.683	51.06	41.86	n.a.
**Total:**			**7.863**	**15.965**	**100.00**	**100.00**	

Chromatogram and Results

Injection Details

Injection Name:	**JD452**	*Run Time (min):*	**40.00**
Vial Number:	**BE2**	*Injection Volume:*	**10.00**
Injection Type:	**Unknown**	*Channel:*	**UV_VIS_3**
Calibration Level:		*Wavelength:*	**222**
Instrument Method:	**IC 50% 0.7mL-40 min run**	*Bandwidth:*	**4**
Processing Method:	**Standard Processing Method**	*Dilution Factor:*	**1.0000**
Injection Date/Time:	**03/Sep/20 11:00**	*Sample Weight:*	**1.0000**

Chromatogram

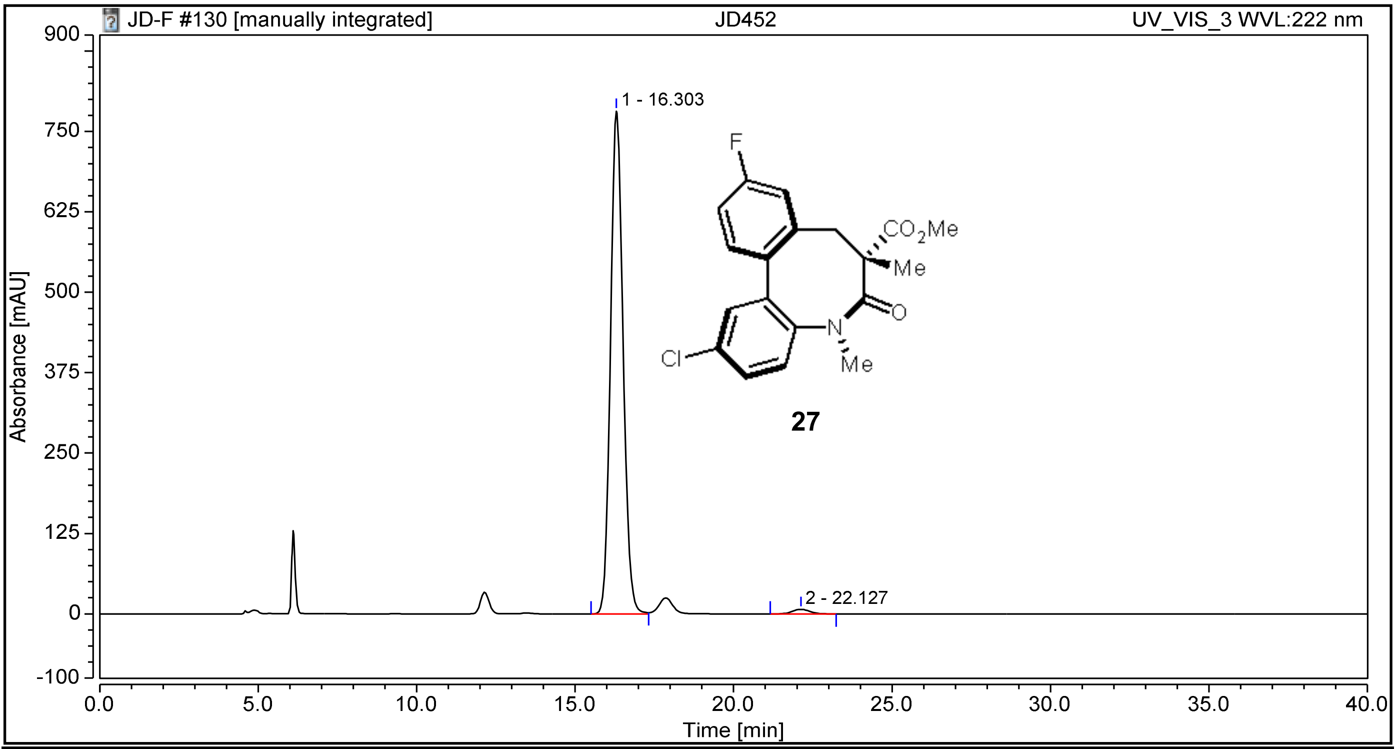

Integration Results

No.	Peak Name	Retention Time min	Area mAU*min	Height mAU	Relative Area %	Relative Height %	Amount n.a.
1		16.303	359.249	781.643	98.75	99.10	n.a.
2		22.127	4.554	7.105	1.25	0.90	n.a.
**Total:**			**363.803**	**788.748**	**100.00**	**100.00**	

Chromatogram and Results

Injection Details

Injection Name:	**JD529(rac)**	*Run Time (min):*	**50.00**
Vial Number:	**BD1**	*Injection Volume:*	**10.00**
Injection Type:	**Unknown**	*Channel:*	**UV_VIS_3**
Calibration Level:		*Wavelength:*	**222**
Instrument Method:	**50% IPA-IC 1mL-pre-equ-50min run**	*Bandwidth:*	**4**
Processing Method:	**Standard Processing Method**	*Dilution Factor:*	**1.0000**
Injection Date/Time:	**21/Oct/20 09:34**	*Sample Weight:*	**1.0000**

Chromatogram

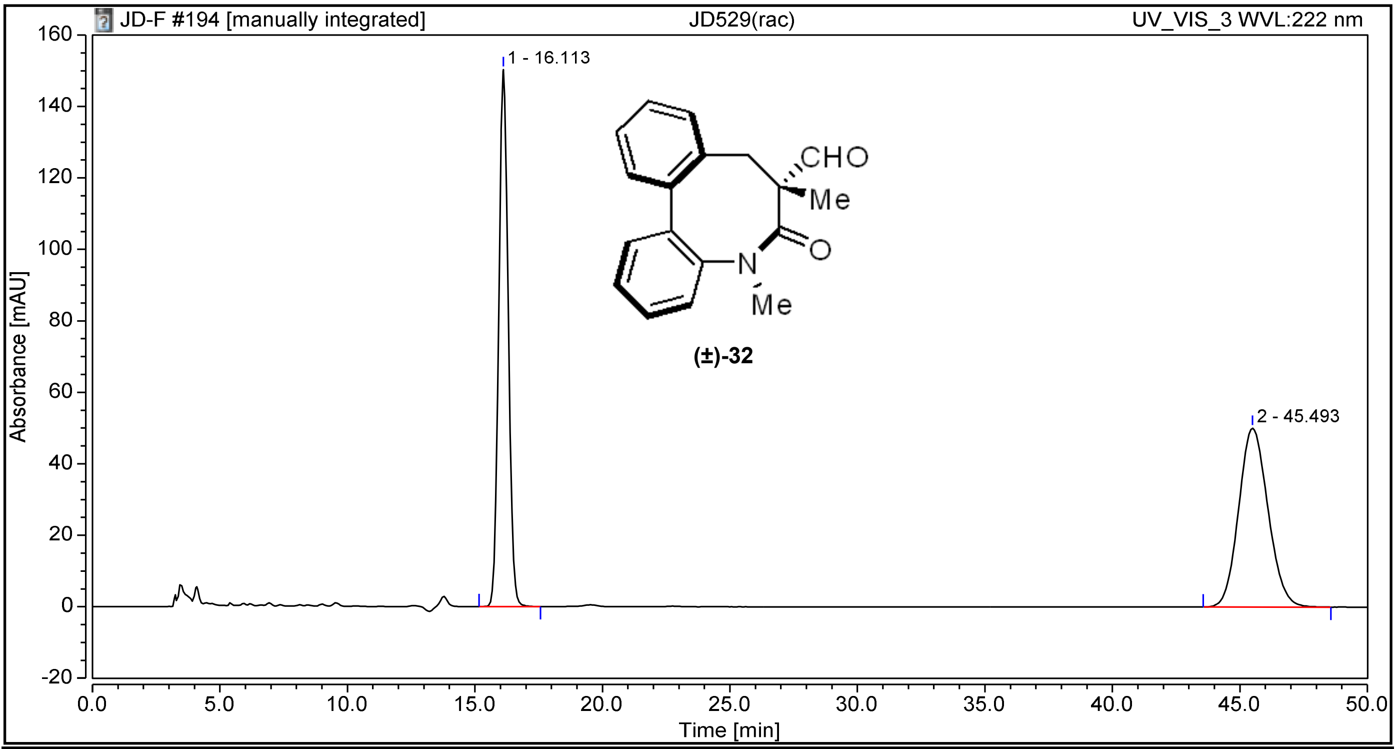

Integration Results

No.	Peak Name	Retention Time<br>min	Area<br>mAU*min	Height<br>mAU	Relative Area<br>%	Relative Height<br>%	Amount<br>n.a.
1		16.113	64.742	150.307	49.22	75.01	n.a.
2		45.493	66.802	50.071	50.78	24.99	n.a.
**Total:**			**131.545**	**200.378**	**100.00**	**100.00**	

Chromatogram and Results

Injection Details

Injection Name:	**JD527**	*Run Time (min):*	**50.00**
Vial Number:	**BD2**	*Injection Volume:*	**10.00**
Injection Type:	**Unknown**	*Channel:*	**UV_VIS_3**
Calibration Level:		*Wavelength:*	**222**
Instrument Method:	**50% IPA-IC 1mL50min run**	*Bandwidth:*	**4**
Processing Method:	**Standard Processing Method**	*Dilution Factor:*	**1.0000**
Injection Date/Time:	**21/Oct/20 10:25**	*Sample Weight:*	**1.0000**

Chromatogram

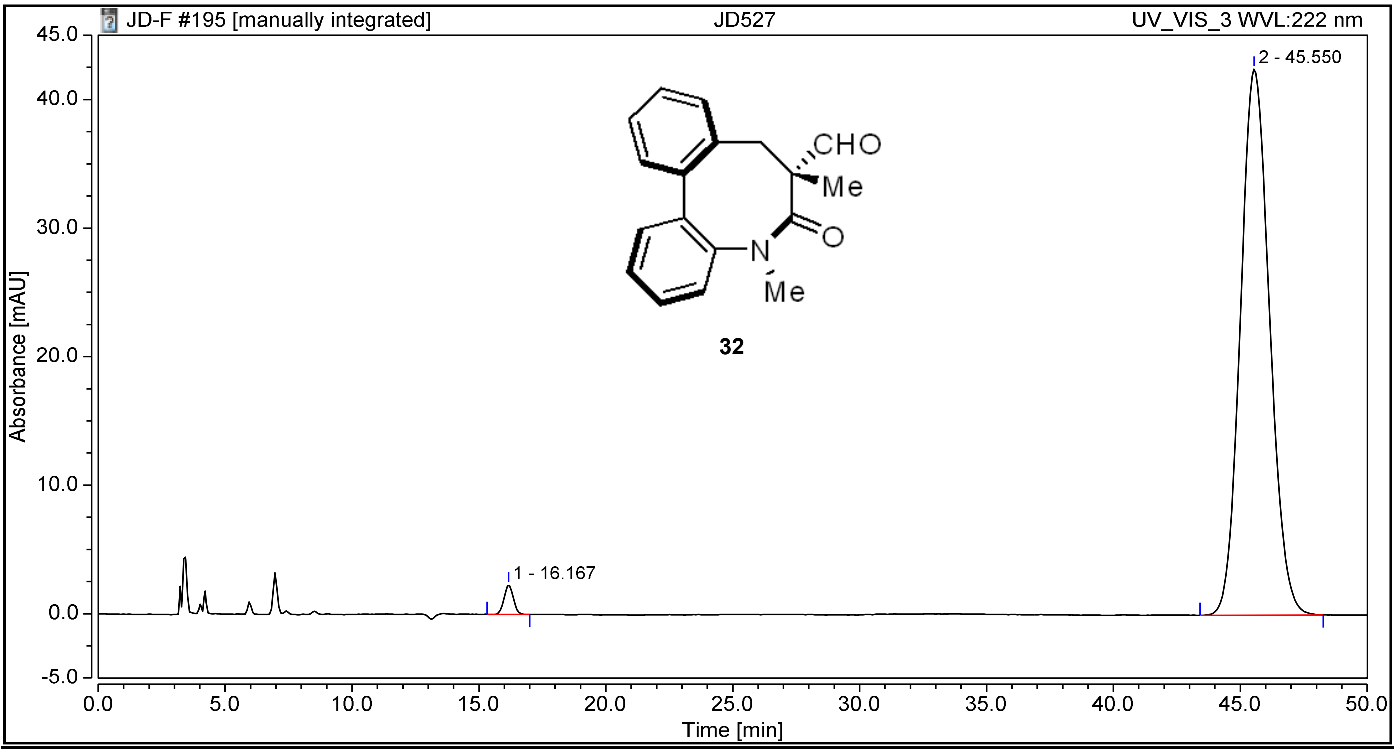

Integration Results

No.	Peak Name	Retention Time<br>min	Area<br>mAU*min	Height<br>mAU	Relative Area<br>%	Relative Height<br>%	Amount<br>n.a.
1		16.167	0.973	2.294	1.69	5.12	n.a.
2		45.550	56.504	42.488	98.31	94.88	n.a.
**Total:**			**57.477**	**44.782**	**100.00**	**100.00**	

Chromatogram and Results

Injection Details

Injection Name:	**JD509(rac)**	*Run Time (min):*	**33.00**
Vial Number:	**BE1**	*Injection Volume:*	**20.00**
Injection Type:	**Unknown**	*Channel:*	**UV_VIS_3**
Calibration Level:		*Wavelength:*	**222**
Instrument Method:	**50% IC 1mL IPA-pre-equ-33 min run**	*Bandwidth:*	**4**
Processing Method:	**Standard Processing Method**	*Dilution Factor:*	**1.0000**
Injection Date/Time:	**07/Oct/20 13:15**	*Sample Weight:*	**1.0000**

Chromatogram

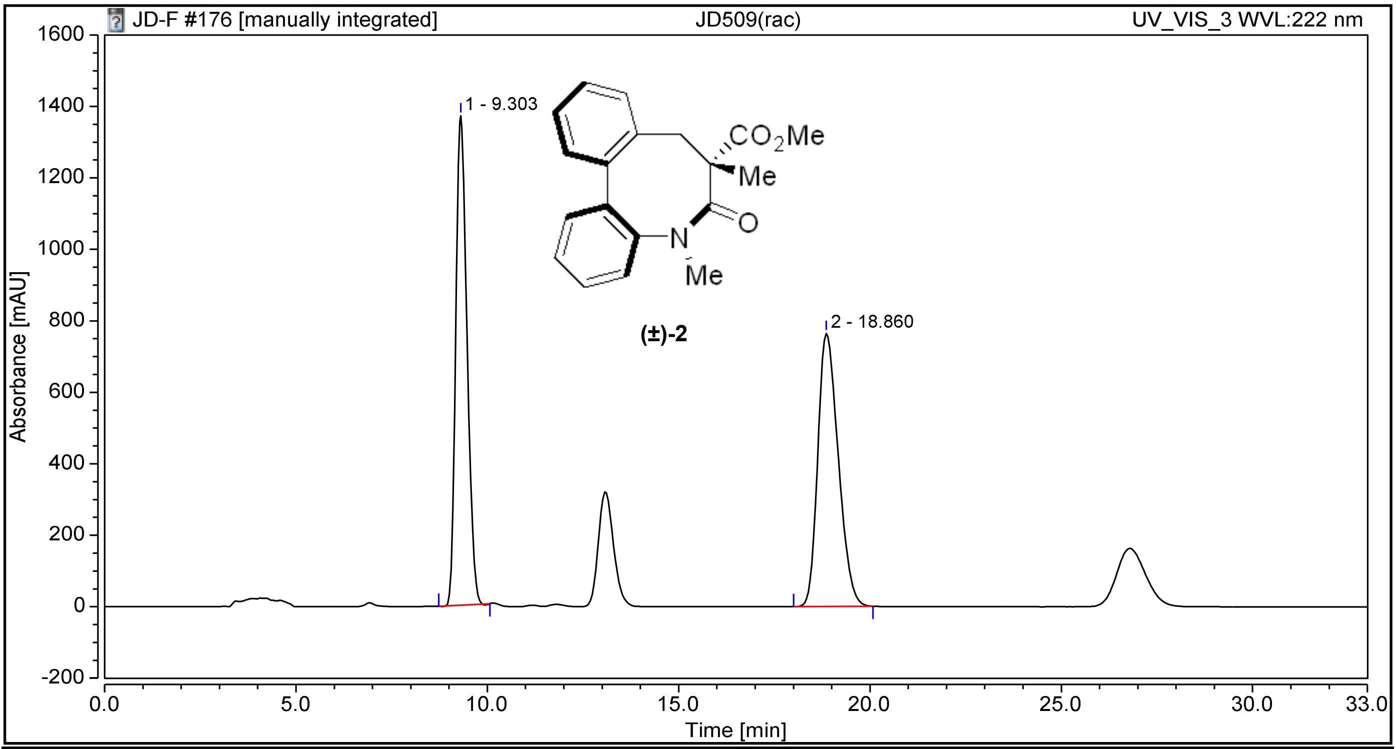

Integration Results

No.	Peak Name	Retention Time min	Area mAU*min	Height mAU	Relative Area %	Relative Height %	Amount n.a.
1		9.303	464.454	1369.956	49.48	64.18	n.a.
2		18.860	474.148	764.618	50.52	35.82	n.a.
**Total:**			**938.602**	**2134.574**	**100.00**	**100.00**	

Chromatogram and Results

Injection Details

Injection Name:	**JD519-300**	*Run Time (min):*	**33.00**
Vial Number:	**BD3**	*Injection Volume:*	**30.00**
Injection Type:	**Unknown**	*Channel:*	**UV_VIS_1**
Calibration Level:		*Wavelength:*	**254**
Instrument Method:	**IC 50% IPA 33 min run**	*Bandwidth:*	**4**
Processing Method:	**Standard Processing Method**	*Dilution Factor:*	**1.0000**
Injection Date/Time:	**10/Oct/20 20:06**	*Sample Weight:*	**1.0000**

Chromatogram

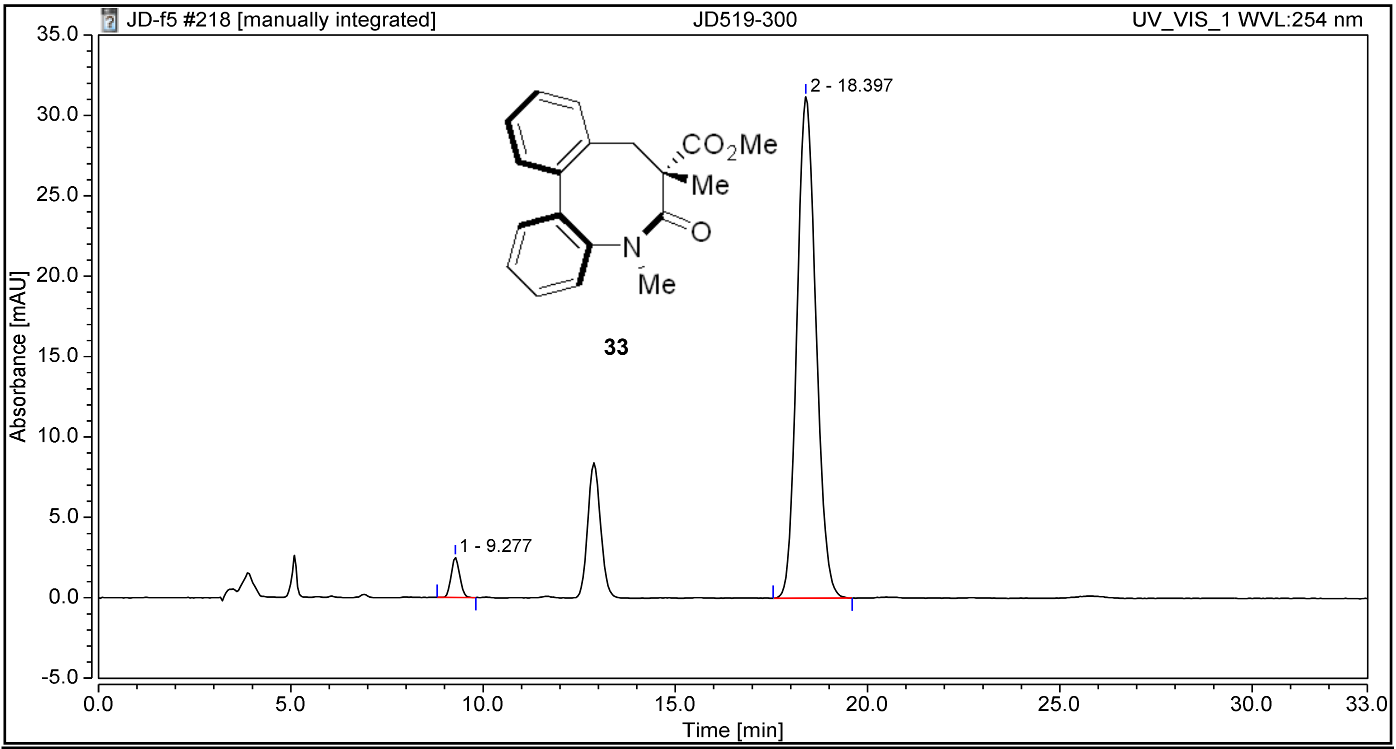

Integration Results

No.	Peak Name	Retention Time<br>min	Area<br>mAU*min	Height<br>mAU	Relative Area<br>%	Relative Height<br>%	Amount<br>n.a.
1		9.277	0.642	2.481	3.46	7.37	n.a.
2		18.397	17.891	31.174	96.54	92.63	n.a.
**Total:**			**18.532**	**33.655**	**100.00**	**100.00**	

Chromatogram and Results

Injection Details

Injection Name:	**JD532(rac)**	*Run Time (min):*	**50.00**
Vial Number:	**BD3**	*Injection Volume:*	**10.00**
Injection Type:	**Unknown**	*Channel:*	**UV_VIS_3**
Calibration Level:		*Wavelength:*	**222**
Instrument Method:	**50% IPA-IC 1mL-pre-equ-50min run**	*Bandwidth:*	**4**
Processing Method:	**Standard Processing Method**	*Dilution Factor:*	**1.0000**
Injection Date/Time:	**23/Oct/20 10:45**	*Sample Weight:*	**1.0000**

Chromatogram

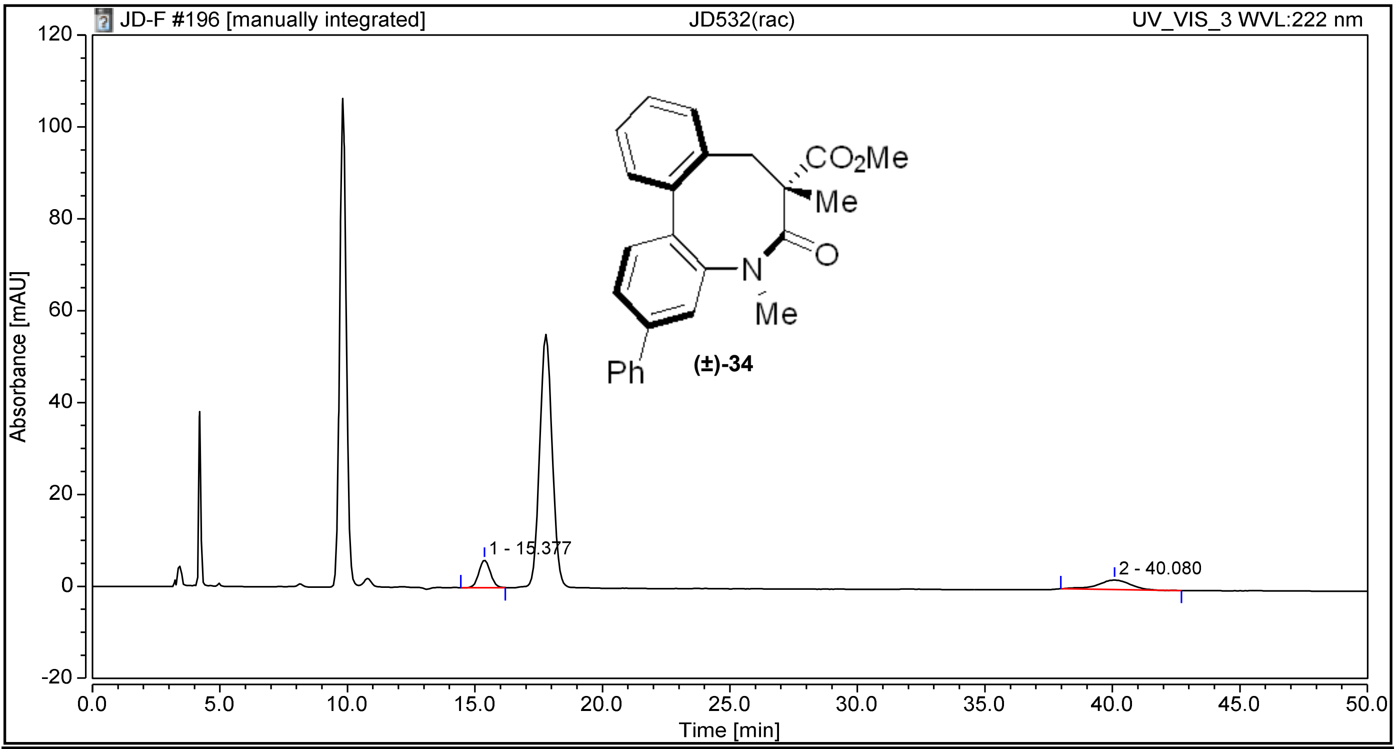

Integration Results

No.	Peak Name	Retention Time<br>min	Area<br>mAU*min	Height<br>mAU	Relative Area<br>%	Relative Height<br>%	Amount<br>n.a.
1		15.377	3.118	5.977	50.85	74.40	n.a.
2		40.080	3.013	2.057	49.15	25.60	n.a.
**Total:**			**6.131**	**8.034**	**100.00**	**100.00**	

Chromatogram and Results

Injection Details

Injection Name:	**JD530**	*Run Time (min):*	**50.00**
Vial Number:	**BD4**	*Injection Volume:*	**10.00**
Injection Type:	**Unknown**	*Channel:*	**UV_VIS_3**
Calibration Level:		*Wavelength:*	**222**
Instrument Method:	**50% IPA-IC 1mL50min run**	*Bandwidth:*	**4**
Processing Method:	**Standard Processing Method**	*Dilution Factor:*	**1.0000**
Injection Date/Time:	**23/Oct/20 11:36**	*Sample Weight:*	**1.0000**

Chromatogram

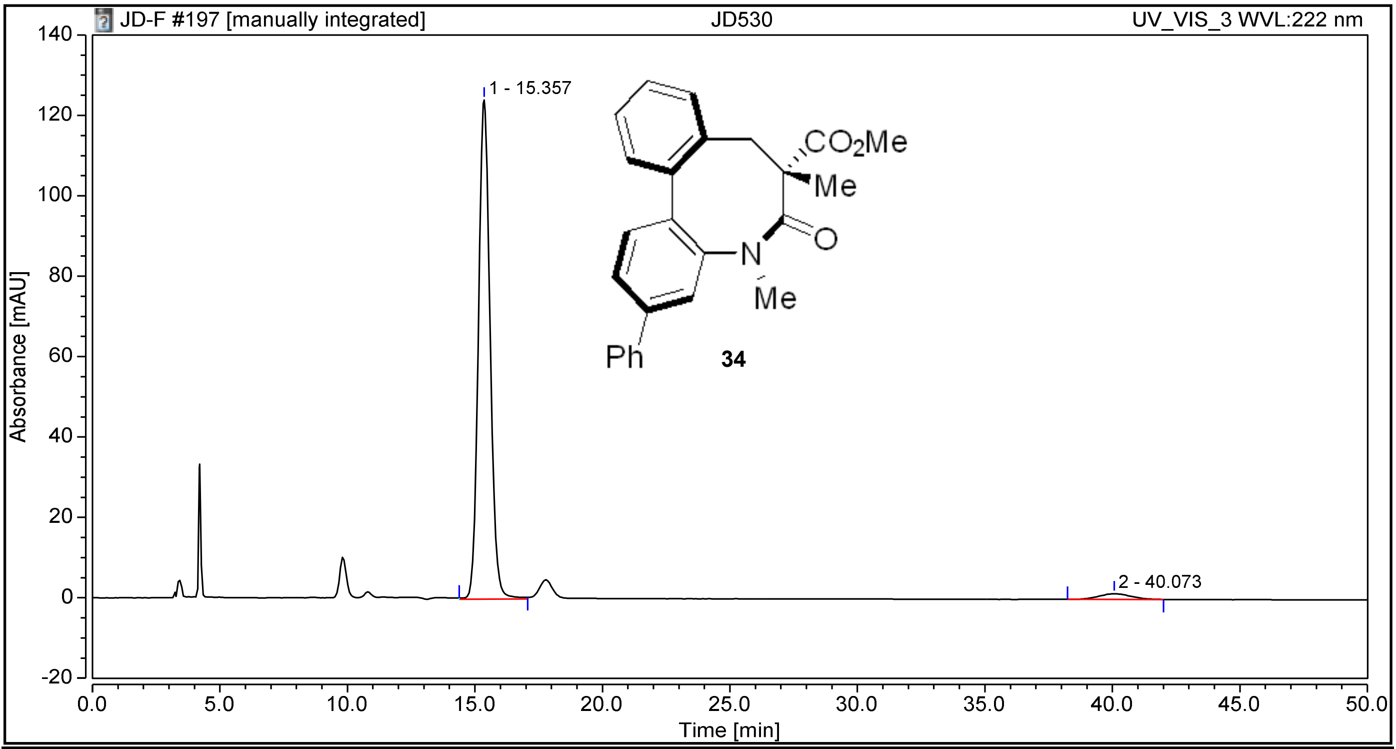

Integration Results

No.	Peak Name	Retention Time min	Area mAU*min	Height mAU	Relative Area %	Relative Height %	Amount n.a.
1		15.357	66.062	124.186	97.00	98.88	n.a.
2		40.073	2.041	1.407	3.00	1.12	n.a.
**Total:**			**68.104**	**125.592**	**100.00**	**100.00**	

9. References.

[1] B. A. Jones, T. Balan, J. D. Jolliffe, C. D. Campbell, M. D. Smith, *Angew. Chem. Int. Ed.* **2019**, *58*, 4596 – 4600.

[2] R. Ding, B. Zheng, Y. Wang, and Y. Peng *Org. Lett* **2015**, *17*, 4128–4131.

[3] (a) H. Iwasaki, T. Eguchi, N. Tsutsui, H. Ohno, and T. Tanaka *J. Org. Chem.* **2008**, *73*, 7145 –7152; (b) A. P. Kale, G. S. Kumar, A. R. K. Mangadan, and M. Kapur *Org. Lett.* **2015**, *17*, 1324 – 1327.

[4] M. A. Alam, K. Arora, S. Gurrapu, S. K. Jonnalagadda, G. L. Nelson, P. Kiprof, S. C. Jonnalagadda, V. R. Mereddy *Tetrahedron* **2016**, *72*, 3795 – 3801.

[5] R. Shen, T. Kusakabe, K. Takahashi and K. Kato *Org. Biomol. Chem.*, **2014**, *12*, 4602-4609
